# A guideline on biomarkers in the diagnosis and evaluation in axial spondyloarthritis

**DOI:** 10.3389/fimmu.2024.1394148

**Published:** 2024-10-30

**Authors:** Dong Liu, Ya Xie, Liudan Tu, Xianghui Wen, Qing Lv, Budian Liu, Mingcan Yang, Xinyu Wu, Xuqi Zheng, Xiqing Luo, Liuzhong Zhou, Jialing Wu, Bin Liu, Kun Wang, Ou Jin, Xiaohong Wang, Jie Qin, Lijun Wu, Dongbao Zhao, Dongyi He, Shanzhi He, Wenhui Huang, Shanhui Ye, Huiqiong Zhou, Jinyu Wu, Yongfu Wang, Shengyun Liu, Zhenbin Li, Zhiming Tan, Chiduo Xu, Youlian Wang, Donghui Zheng, Feng Zhan, Changsong Lin, Ya Wen, Jiayun Wu, Shenghui Wen, Zetao Liao, Yan Shen, Kehu Yang, Jieruo Gu

**Affiliations:** ^1^ Department of Rheumatology, The Third Affiliated Hospital of Sun Yat-Sen University, Guangzhou, China; ^2^ Department of Rheumatology, Shenzhen Longhua District Central Hospital, Shenzhen, China; ^3^ Shenzhen Longhua Institute of Immunology Transformation, Shenzhen, China; ^4^ Department of Rheumatology, The Seventh Affiliated Hospital of Sun Yat-Sen University, Shenzhen, China; ^5^ Department of Spine Surgery, The Third Affiliated Hospital of Sun Yat-Sen University, Guangzhou, China; ^6^ Department of Joint Surgery, The Third Affiliated Hospital of Sun Yat-Sen University, Guangzhou, China; ^7^ Department of Radiology, The Third Affiliated Hospital of Sun Yat-Sen University, Guangzhou, China; ^8^ Department of Rheumatology, People’s Hospital of Xinjiang Uygur Autonomous Region, Urumchi, China; ^9^ Department of Rheumatology, Changhai Hospital, Shanghai, China; ^10^ Department of Rheumatology, Shanghai Guanghua Hospital, Shanghai University of Traditional Chinese Medicine, Shanghai, China; ^11^ Department of Rheumatology, Zhongshan City People’s Hospital, Zhongshan, China; ^12^ Department of Rheumatology, The Second Affiliated Hospital of Guangzhou Medical University, Guangzhou, China; ^13^ Department of Rheumatology, The First Affiliated Hospital of Guangzhou Medical University, Guangzhou, China; ^14^ Department of Rheumatology, The Fourth Central Hospital Chinese PLA Medical School, Xi’an, China; ^15^ Department of Rheumatology, The First Affiliated Hospital of Guangxi University of Chinese Medicine, Nanning, China; ^16^ Department of Rheumatology, The First Affiliated Hospital of Baotou Medical College, Baotou, China; ^17^ Department of Rheumatology, The First Affiliated Hospital of Zhengzhou University, Zhengzhou, China; ^18^ Department of Rheumatology, Bethune International Peace Hospital, Shijiazhuang, China; ^19^ Department of Rheumatology, Huizhou Central Hospital, Huizhou, China; ^20^ Department of Rheumatology, The Second People‘s Hospital of Shenzhen City, Shenzhen, China; ^21^ Department of Rheumatology, Jiangxi Province People’s Hospital, Nanchang, China; ^22^ Department of Rheumatology, Sun Yat-Sen Memorial Hospital, Sun Yat-Sen University, Guangzhou, China; ^23^ Department of Nephropathy and Rheumatology, Hainan General Hospital, Hainan Affiliated Hospital of Hainan Medical University, Haikou, China; ^24^ Department of Rheumatology, The First Affiliated Hospital of Guangzhou University of Chinese Medicine, Guangzhou, China; ^25^ National Laboratory of Medical Molecular Biology, Institute of Basic Medical Sciences, Chinese Academy of Medical Science and Peking Union Medical College, Tsinghua University, Beijing, China; ^26^ Evidence-Based Medicine Center, School of Basic Medical Sciences, Lanzhou University, Lanzhou, China

**Keywords:** axial spondyloarthritis, biomarker, guideline, HLA-B27, C-reactive protein

## Abstract

**Objective:**

To develop a guideline for selecting biomarkers in the diagnosis and assessment in patients with axial spondyloarthritis (axSpA).

**Method:**

A joint effort was carried out by the core team, the literature review team and the multidisciplinary voting panel to formulate recommendations regarding biomarkers in axSpA, using an evidence-based and consensus-based strategy. Certainty of evidence and strength of recommendation were determined, and levels of agreement within the voting panel were calculated.

**Results:**

A total of 20 recommendations were formulated in this guideline, with levels of agreement ranging from 6.48 to 9.71. The two strong recommendations, HLA-B27 testing in patients suspected of axSpA and regular-interval monitoring of CRP/ESR represent the status quo of axSpA evaluation, while the 13 conditional recommendations represent the promising biomarkers with clinical utility in diagnosis, disease activity assessment, prediction of radiographic progression and therapeutic responses. This guideline does not dictate clinical choices of tests on axSpA, and decisions should be made based on comprehensive consideration of costs, accessibility, patients’ values and willingness as well as the objective of testing in the local context.

**Conclusion:**

This guideline addresses the interpretation of the clinical significance of biomarkers in axSpA, and the biomarkers endorsed in this guideline composed a clinical toolkit for healthcare professionals to choose from.

## 1 Introduction

Axial spondyloarthritis (axSpA) is a disorder predominantly involving the axial skeleton, characterized by inflammation at the sacroiliac joint and spine, often with involvements of the peripheral joints and entheses, as well as extra-articular structures such as the anterior uvea and gastrointestinal tract (1, 2). It could potentially impose significant disease burden on the patients, which could derive from the pain caused by active disease, and functional disability caused by new bone formation and joint ankylosis subsequent to persistent inflammation (3, 4). Timely institution of appropriate treatment is critical to the remission of active disease and precluding radiographic progression. An early and correct diagnosis is important in this process, which often relies on both imaging examinations and laboratory findings, notably HLA-B27. However, even the combination of MRI and HLA-B27 testing does not guarantee complete accuracy of axSpA diagnosis (5); more efforts are still made to identify biomarkers that could potentially assist in the diagnosis of axSpA. Moreover, the concept of precision medicine has put forth new requirements to the medical community (6), even more so in the context of axSpA diagnosis and treatment. The taxonomy of axSpA encompasses various groups of diseases, with differing tendencies of radiographic progression with various clinical outcomes (1). Rheumatologists have to choose wisely from the toolkit of myriad biomarkers, properly interpret their clinical significance, stratify the patients based on disease activity and tendency of radiographic progression, tailor treatment and monitor therapeutic responses. Much research has been devoted to the identification and interpretation and the biomarkers associated with axSpA (7, 8). The translation of these biomarkers from research to clinical practice is, alas, still much lacked. Based on such observations, the objective of this guideline is to examine recent advances of biomarkers in axSpA and verify their reliability, formulating recommendations for rheumatologists about what biomarkers to choose in clinical practice.

## 2 Methods

This guideline was developed using the framework of the Grading of Recommendations Assessment, Development and Evaluation (GRADE) methodology to assess the certainty of evidence and develop recommendations (9–11). The detailed description of the methodology is explained in **Supplementary Appendix 1** in **Supplementary Table 1**. The Core Team, the Literature Review Team and the Voting Panel led a joint effort to devise a preliminary set of biomarkers associated with axSpA. The Core Team and the Voting Panel comprised experts in rheumatology, orthopedic surgery and GRADE methodology. The complete list of participants could be seen in **Supplementary Appendix 2** in **Supplementary Table 2**. Biomarkers discussed in this project were defined as molecules, genetic variants or other indicators which could be measured using blood, fecal or urine sample. To explore the significance and clinical relevance of each potential biomarker, assignments were handed out to each member of the Literature Review Team to conduct systemic literature reviews (SLRs). Search strategies and study inclusion process could be seen in **Supplementary Appendices 3** and **4** in **Supplementary Tables 3, 4**. This guideline was registered under the registration number of IPGRP-2020CN093.

Moving from evidence to recommendations, a recommendation is formulated under the comprehensive consideration of costs, accessibility, clinical significance and certainty of evidence of each biomarker. The rationale of developing recommendations is that a biomarker has to provide information which is helpful in the diagnosis or stratification of axSpA patients and ultimately could assist in optimizing treatment options. To this end, the clinical significance of each biomarker is stratified to four levels: a) diagnostic utility; b) indication of disease activity; c) prediction of radiographic progression; d) prediction or evaluation of therapeutic responses. A mere up-regulation or down-regulation does not suffice to make a recommendation. The Literature Review Team has to gather evidence regarding the four levels of clinical significance and prove that a certain biomarker could provide significant incremental information which could help rheumatologists or physicians form a better understanding of the axSpA patients. The strengths of each recommendation were classified as strong or conditional. A strong recommendation indicates that this biomarker should be considered in daily clinical practice given its significance in the four aspects, while a conditional recommendation indicates that this biomarker provides potentially helpful information to a certain extent and could be considered by the clinician.

Recommendation statements were written based on the evidence reports. An online meeting was held, during which the recommendation statements and the evidence reports were presented to the Voting Panel. Having reviewed the evidence reports and the recommendation statements, each member of the Voting Panel voted for or against the recommendations and rated the level of agreement. At least a consensus of 70% of the Voting Panel was required to include the preliminary recommendations in the final guideline.

It should be clarified that biomarkers discussed in this project only applies to patients suspected of axSpA or already diagnosed as axSpA. Since there is no preventive therapy, we do not recommend any of these biomarkers in the screening of the general population, unless an individual is at great risk.

## 3 Results

The recommendations of this guideline were summarized in **Table 1**, and the clinical significance of each biomarker was stratified in **Table 2**. The process of biomarker selection was presented in **Table 3**. A brief executive summary could be seen in **Supplementary Appendix 8**.

**Table 1:** Recommendations on biomarkers pertinent to the diagnosis and evaluation of patients with axSpA.

	Recommendations	Certainty of evidence	Approval rate	Level of agreement
Biomarkers pertinent to diagnosis				
1	We strongly recommend HLA-B27 testing in patients suspected of axSpA.	High	100.00%	9.71
2	We conditionally recommend testing of HLA-B27 subtypes in patients with difficulties in diagnosis of axSpA.	Medium	100.00%	8.10
3	We conditionally recommend testing of polygenic risk score (PRS) in patients suspected of axSpA.	Low	90.48%	7.05
4	We strongly recommend against testing of antibodies in patients with axSpA in daily practice, including anti-CD74 antibodies, anti-sclerostin and anti-noggin antibodies, antibodies against microbial targets.	Low	90.48%	8.10
Biomarkers pertinent to inflammatory status				
5	We strongly recommend monitoring of CRP and ESR concentrations in patients with axSpA over usual care without CRP or ESR monitoring.	High	100.00%	9.71
6	We conditionally recommend regular-interval monitoring of SAA in patients with axSpA	Medium	95.24%	7.14
7	We conditionally recommend the testing of leptin and HMW-APN in patients with axSpA.	Low	90.48%	6.48
8	We conditionally recommended against testing of VEGF in patients with axSpA.	Very low	95.24%	7.62
9	We conditionally recommend the testing of calprotectin in patients with axSpA, especially using the fecal sample to monitor gut inflammation.	Medium	85.71%	6.95
10	We conditionally recommend the testing of IL-6, IL-17 and TNF-α in the monitoring of disease activity in patients with axSpA.	Low	90.48%	7.24
11	We conditionally recommend the analysis of peripheral lymphocyte subsets in patients with axSpA.	Medium	95.24%	7.52
12	We strongly recommend against testing of non-coding RNAs in patients with axSpA in daily practice.	Very low	71.43%	7.52
Biomarkers pertinent to bone destruction and formation				
13	We conditionally recommend testing of bone turnover markers, including CTX-I and PINP, in patients with axSpA.	Low	90.48%	7.05
14	We conditionally recommend testing of sclerostin in patients with axSpA.	Low	95.24%	7.24
15	We conditionally recommend testing of DKK-1 in patients with axSpA.	Low	85.71%	7.14
16	We conditionally recommend against testing of OPG/RANKL/RANK in patients with axSpA.	Low	85.71%	7.14
17	We conditionally recommend against testing of MMP-3 in patients with axSpA.	Very low	95.24%	7.05
Biomarkers pertinent to prediction of therapeutic safety and efficacy				
18	We conditionally recommend genotyping of CYP2C9 alleles before axSpA patients start medication of NSAIDs metabolized by CYP2C9, such as diclofenac, meloxicam and celecoxib.	Low	95.24%	7.33
19	We conditionally recommend genetyping of NAT2 alleles before axSpA patients start medication of sulfasalazine.	Low	90.48%	7.33
20	We conditionally recommend measurement of antidrug antibodies in patients receiving medication of TNF-α inhibitors at the time of clinical non-responses.	Medium	95.24%	8.57

Deep red indicates that this guideline strongly recommends against the testing of this biomarker in patients with axSpA in clinical practice, while light red indicates that this guideline conditionally recommends against testing of this biomarker. Deep green indicates that this guideline strongly recommends the testing of the biomarker for the corresponding purposes in clinical practice, while light green indicates conditional recommendation, which should also take into consideration the costs, accessibility and patients’ willingness.

**Table 2:** Stratification of the clinical significance of the biomarkers discussed in this guideline.

Biomarkers	Diagnosis	Assessment of disease activity	Prediction of radiographic progression	Prediction/monitoring of therapeutic responses
HLA-B27	√			
HLA-B27 subtypes	√			
Polygenic risk score	√			
Antibodies, including anti-CD74 antibodies, anti-sclerostin and anti-noggin antibodies, antibodies againts microbial targets				
CRP	√	√	√	√
ESR		√	√	√
SAA		√		
Leptin and HMW-APN		√	√	
VEGF				
Calprotectin		√		
Inflammatory cytokines including IL-6, IL-17 and TNF-α		√		
Peripheral lymphocyte subsets		√		
Non-coding RNAs				
Bone turnover markers, including sCTX and PINP			√	
Sclerostin			√	
DKK-1			√	
OPG/RANKL/RANK				
MMP-3				
NSAIDs-related genes				√
SSZ-related genes				√
Anti-drug antibodies				√

The symbol √ indicates that results of the systemic literature review supports that this biomarker bears clinical significance in this field.

**Table 3:** The selection process of biomarkers in this guideline.

Preliminary set		Systemic literature review	Preliminary recommendations	Final recommendations
HLA-B27	DKK-1	HLA-B27	HLA-B27	HLA-B27
HLA-B27 subtypes	OPG/RANKL/RANK	HLA-B27 subtypes	HLA-B27 subtypes	HLA-B27 subtypes
Genes	MMP-3	Genes	PRS score	PRS score
Antibodies, including anti-CD74 antibodies, anti-sclerostin and anti-noggin antibodies, antibodies againts microbial targets	MMP-8	Antibodies, including anti-CD74 antibodies, anti-sclerostin and anti-noggin antibodies, antibodies againts microbial targets	Antibodies, including anti-CD74 antibodies, anti-sclerostin and anti-noggin antibodies, antibodies againts microbial targets	Antibodies, including anti-CD74 antibodies, anti-sclerostin and anti-noggin antibodies, antibodies againts microbial targets
CRP	MMP-9	CRP	CRP and ESR	CRP and ESR
ESR	BMP-2	ESR	SAA	SAA
SAA	TNC	SAA	Leptin and HMW-APN	Leptin and HMW-APN
Adipokines, including leptin, adiponectin and resistin	Fetuin A	Adipokines, including leptin, adiponectin and resistin	VEGF	VEGF
VEGF	YKL-40	VEGF	Calprotectin	Calprotectin
CXCL8	MIF	Calprotectin	Inflammatory cytokines including IL-6, IL-17 and TNF-α	Inflammatory cytokines including IL-6, IL-17 and TNF-α
HGF	Vitamin D	non-coding RNA	Peripheral lymphocyte subsets	Peripheral lymphocyte subsets
Calprotectin	Gut microbiota	Inflammatory cytokines including IL-6, IL-17 and TNF-α	non-coding RNA	non-coding RNA
Pentraxin 3	Metabolomics signature	Peripheral lymphocyte subsets	Bone turnover markers, including β-CTX and PINP	Bone turnover markers, including β-CTX and PINP
non-coding RNA	NSAIDs-related genes	Bone turnover markers, including β-CTX and PINP	Sclerostin	Sclerostin
Inflammatory cytokines including IL-6, IL-17 and TNF-α	SSZ-related genes	C1M, C2M, C3M, C6M and VICM	DKK-1	DKK-1
TL1A	Anti-drug antibodies	Sclerostin	OPG/RANKL/RANK	OPG/RANKL/RANK
Peripheral lymphocyte subsets		DKK-1	MMP-3	MMP-3
Neutrophil-to-lymphocyte ratio		OPG/RANKL/RANK	NSAIDs-related genes (CYP2C9)	NSAIDs-related genes (CYP2C9)
Fibrinogen-to-albumin ratio		MMP-3	SSZ-related genes (NAT2)	SSZ-related genes (NAT2)
Bone turnover markers, including β-CTX and PINP		BMP-2	Anti-drug antibodies	Anti-drug antibodies
C1M, C2M, C3M, C6M and VICM		TNC		
COMP		Gut microbiota		
Aggrecan		Metabolomics signature		
Osteocalcin		NSAIDs-related genes		
RBP4		SSZ-related genes		
Sclerostin		Anti-drug antibodies		

Deep red indicates that this guideline strongly recommends against the testing of this biomarker in patients with axSpA in clinical practice, while light red indicates that this guideline conditionally recommends against testing of this biomarker. Deep green indicates that this guideline strongly recommends the testing of the biomarker for the corresponding purposes in clinical practice, while light green indicates conditional recommendation, which should also take into consideration the costs, accessibility and patients’ willingness.

### 3.1 We strongly recommend HLA-B27 testing in patients suspected of axSpA

It has long been established that HLA-B27 is of critical significance to the diagnosis of axSpA, even more so to its prototypical type, namely ankylosing spondylitis (AS) (7). About 85% of AS patients are HLA-B27 positive, while only about 8% of the general population carry this gene (12). It serves as an indispensable component of the clinical arm in the Assessment of Spondyloarthritis International Society (ASAS) classification criteria of axSpA (13). However, being HLA-B27 positive does not necessarily equate with a diagnosis of axSpA, since the majority of HLA-B27 positive individuals do not develop axSpA (14). Diagnosis of axSpA should be based on clinical characteristics, HLA-B27 status, MRI and sometimes other biomarkers. The voting panel unanimously agreed that HLA-B27 should be tested in patients suspected of axSpA, more specifically, in patients with chronic lower back pain for over 3 months and the onset is before 45 years old.

Another intriguing observation is the association between HLA-B27 and radiographic progression, especially in the sacroiliac joint. It has been observed that HLA-B27-positive patients were more likely to develop from non-radiographic axSpA (nr-axSpA) to AS (15); however, HLA-B27 positivity has no value in the prediction of the radiographic progression or syndesmophyte formation in the spine (16). One argument is that HLA-B27 positivity can merely be associated with the high probability of true inflammation, while HLA-B27 per se does not participate in the process of new bone formation (7).

Some studies reported that HLA-B27-positive patients were more likely to respond to TNF-α inhibitors (17, 18). However, we believe this finding must be interpreted with caveat. Such observation could be attributed to the fact that HLA-B27-positive patients were more likely to receive early diagnosis and appropriate treatment. On the other hand, efficacy of secukinumab seemed to be not influenced by HLA-B27 status (19).

### 3.2 We conditionally recommend testing of HLA-B27 subtypes in patients with difficulties in diagnosis of axSpA

The heterogeneity of phenotypes and clinical outcomes in axSpA arise in part from the various subtypes of HLA-B27. To date, over 200 subtypes of HLA-B27 have been identified, but only a few were proved to be associated with the increased risk of axSpA (20). Our systemic literature review and meta-analysis concluded that HLA-B27*04 and 05 were significantly associated with an increased risk of axSpA (OR=1.91, 95% CI 1.08-3.39; OR=1.65, 95% CI 1.34-2.05) (**Supplementary Appendix 6** in **Supplementary Table 6**), while carriers of HLA-B27*06 and 07 were less likely to develop axSpA. (OR=0.13, 95% CI 0.05-0.29; OR=0.30, 95% CI 0.17-0.54) Moreover, previous studies reported that peripheral arthritis was more prevalent in patients with HLA-B27*04 (21). The voting panel agreed on this recommendation that testing of HLA-B27 subtypes could increase the confidence of diagnosis, but it should only be considered in cases where imaging examinations and other laboratory tests returned ambiguous results. In terms of methodology, this guideline endorses DNA microarray or PCR-SSP in HLA-B27 subtype testing.

### 3.3 We conditionally recommend testing of polygenic risk score (PRS) in patients suspected of axSpA

Despite its significant association with axSpA, HLA-B27 only contributes to ~20% of the heritability of axSpA (22). Genomic-wide association studies have identified numerous genetic loci which were associated with the genetic risks of axSpA (22–24). Among these genetic loci, MHC genes confer more significant genetics risks than non-MHC genes (25). Our meta-analysis confirmed that HLA-DRB1, especially the allele HLA-DRB*12, as well as HLA-B60 was associated with a higher risk of axSpA. (**Supplementary Appendix 6** in **Supplementary Table 6**). Researchers aggregated from 110 to thousands of the most relevant single nucleotide polymorphisms and devised polygenic risk score (PRS) to assist in the diagnosis of axSpA. Results showed that the overall PRS (AUC=0.924), which included MHC and non-MHC single nucleotide polymorphisms, outperformed HLA-B27 (AUC=0.869), MRI (AUC=0.885) or CRP (AUC=0.700) in diagnostic utility (25). 90.48% of the voting panel agreed on the recommendation of PRS for patients suspected of axSpA.

### 3.4 We strongly recommend against testing of antibodies in patients with axSpA in daily practice, including anti-CD74 antibodies, anti-sclerostin and anti-noggin antibodies, antibodies against microbial targets

There is not sufficient evidence to prove the diagnostic values of antibodies in axSpA. Despite earlier studies showing that anti-CD74 antibodies and anti-CLIP antibodies could be detected in 69% and 85.1% of axSpA patients (26, 27), subsequent studies showed high inconsistency regarding their diagnostic capacity. The SPACE cohort showed that the positive predictive value (PPV) and negative predictive value (NPV) of anti-CD74 antibodies were only 58.8% and 59.1% (28), and its diagnostic capacity in East Asians population was also limited (29). Anti-sclerostin and anti-noggin antibodies could be implicated in signaling pathways regulating new bone formation (30), but there is no reliable evidence proving that these antibodies could be predictors of new bone formation. Antibodies against microbial targets such Klebsiella pneumonia, Salmonella, Saccharomyces cerevisiae could also be detected in axSpA (31), but their diagnostic values remained unclear. 90.48% of the voting panel agreed on the recommendation against testing of antibodies in axSpA in daily practice.

### 3.5 We strongly recommend monitoring of CRP and ESR concentrations in patients with axSpA over usual care without CRP or ESR monitoring

As an acute phase reactant, C-reactive protein (CRP) is a long-established biomarker of disease activity in axSpA patients, while erythrocyte sedimentation rate (ESR) is another important indicator of inflammation. Serum CRP level above the upper limit has only a sensitivity of 50% and a specificity of 80% (25), but it was included as a SpA feature in the ASAS classification criteria for axSpA (13). Regarding their association with radiographic progression, our meta-analyses concluded that the baseline levels of CRP and ESR were both significant predictors of radiographic progression of the spine, more specifically the mSASSS score increase. (OR=1.02, 95%CI 1.00-1.03; OR=1.02, 95% 1.01-1.03) (**Supplementary Appendix 6** in **Supplementary Table 6**) Patients with elevated CRP levels seemed to respond better to TNF-α inhibitors such as etanercept (32) and adalimumab (33), as well as IL-17 inhibitors such as bimekizumab (34) and secukinumab (19). The voting panel unanimously agreed on the recommendation of regular -interval monitoring of CRP and ESR over usual care without CRP or ESR monitoring. More specifically, CRP/ESR levels should be monitored at 0 week, 3 weeks, 6 weeks, 12 weeks and every 3 months during follow-up visits. This recommendation was in line with the 2019 ACR recommendations for the treatment of radiographic and non-radiographic axial spondyloarthritis, which conditionally recommended regular-interval use and monitoring of CRP concentrations or ESR over usual care without regular CRP or ESR monitoring (35). The recommended assay for CRP is immunoturbidimetry.

### 3.6 We conditionally recommend regular-interval monitoring of SAA in patients with axSpA

Serum amyloid A (SAA) is another acute phase reactant indicative of active inflammation, and multiple studies have established the strong positive correlation between SAA and other indices of disease activity, such as BASDAI and CRP (36–38). SAA could be an addition to other inflammatory markers, and baseline levels of CRP and SAA combined could be predictors of ASAS response for patients receiving treatment of TNF-α inhibitors (38). SAA should be tested at first visits and follow-up visits to monitor disease activity. Moreover, serum SAA levels could be a potential biomarker of amyloid A amyloidosis, a known complication in radiographic axial spondyloarthritis (36). 95.24% of the voting panel agreed on this recommendation.

### 3.7 We conditionally recommend the testing of leptin and HMW-APN in patients with axSpA

Adipokines are mostly secreted by adipocytes and participate in multiple metabolic processes. The most researched adipokines include leptin, adiponectin and resistin (39). Leptin is also considered a pro-inflammatory cytokine given its capacity of stimulating T cell proliferation and enhancing T cell activation (40), while adiponectin is considered an anti-inflammatory cytokine since it could inhibit the production of inflammatory cytokines (41). Meta-analysis showed that leptin was up-regulated in the serum of AS patients <40 years old, while AS patients ≥ 40 years old had significantly higher serum adiponectin levels (42). Several studies have investigated the association between the adipokines and radiographic progression, and results showed that both higher baseline levels of leptin and lower baseline levels of high-molecular-weight adiponectin (HMW-APN) were predictors of radiographic progression in axSpA (43, 44). Given their relevance in disease activity and radiographic progression, this guideline recommends testing of leptin and HMW-APN with an approval rate of 90.48%, but costs and accessibility should also be considered before ordering a test. We also conducted a systemic literature review on resistin (**Supplementary Appendix 6** in **Supplementary Table 6**), which also belongs in adipokines, but it was decided that resistin could not provide incremental values to leptin and HMW-APN.

### 3.8 We conditionally recommended against testing of VEGF in patients with axSpA

Vascular endothelial growth factor (VEGF) is a critical mediator in angiogenesis, and it is also implicated in the inflammatory process by increasing the vascular permeability and promoting infiltration of inflammatory cells (45). Although meta-analysis showed that serum levels of VEGF were significantly higher in patients with axSpA than healthy controls, it also showed that VEGF levels were poorly correlated with disease activity (30). Moreover, baseline levels of VEGF could predict neither spinal inflammation nor syndesmophyte formation (46). There is no sufficient evidence to build a case for the recommendation of VEGF, and 95.24% of the voting panel agreed on the recommendation against testing of VEGF in clinical practice.

### 3.9 We conditionally recommend the testing of calprotectin in patients with axSpA, especially using the fecal sample to monitor gut inflammation

Calprotectin is a cytosolic protein complex comprising S100A8 and S100A9. When excreted, it could combine with Toll-like receptor 4 (TLR4) and receptor for advanced glycation end products (RAGE), followed by activation of innate immune responses and inflammation (47). It has been acknowledged that fecal calprotectin is a sensitive biomarker of inflammatory bowel disease (IBD) and has been applied in the clinical practice (48). Meta-analysis confirmed that both serum and fecal calprotectin were significantly elevated in spondyloarthritis patients and associated with disease activity (49). Previous epidemiological study showed that 46.2% of SpA patients exhibited microscopic gut inflammation, and axSpA was often complicated with IBD (50). The value of calprotectin lies in its ability of monitoring gut inflammation, since there is still a lack of non-invasive approaches of monitoring gut inflammation apart from endoscopy. We believe that calprotectin, especially when tested with fecal sample, could close that gap and provide critical information about inflammation in the gastrointestinal tract. This is increasingly relevant since IL-17 inhibitors should be used with caution in patients with susceptibility to IBD (51).

### 3.10 We conditionally recommend the testing of IL-6, IL-17 and TNF-α in the monitoring of disease activity in patients with axSpA

1. Meta-analysis confirmed that the serum interleukin-6 (IL-6) levels were significantly elevated in patients with axSpA (52), and multiple studies have confirmed the association between IL-6 and CRP as well as ESR (53, 54). One study reported that baseline levels of IL-6 could predict changes of mSASSS.

2. Interleukin-17 (IL-17) plays an important role both in the inflammatory process and in the ossification process. IL-17 is significantly elevated in the serum of axSpA patients (52).

3. Tumor necrosis factor α (TNF-α) is also a cytokine reflecting inflammatory status, with potential correlation with other inflammatory indicators such as ESR and IL-6 (53). Evidence is still lacked regarding its capability in predicting radiographic progression and therapeutic responses.

90.48% of the voting panel agreed on this recommendation.

### 3.11 We conditionally recommend the analysis of peripheral lymphocyte subsets in patients with axSpA

Our meta-analysis showed that the proportions of Th17 cells as well as Th1/Th2 ratios in the peripheral blood is significantly elevated in patients with axSpA, while Tregs were down-regulated (**Supplementary Appendix 6** in **Supplementary Table 6**). The Th17 cells are known as an important lymphocyte subset in the pathogenesis of axSpA, notably in the skin disease as well as enthesitis (55). Tregs possess immunomodulatory traits and lower proportions of Tregs could indicate active inflammation (55). Previous studies have showed that Th17 cells were positively correlated with disease activity, while Tregs were inversely correlated with disease activity (56, 57). However, costs and accessibility should be considered before a flow cytometric analysis is ordered.

### 3.12 We strongly recommend against testing of non-coding RNAs in patients with axSpA in daily practice

There have been extensive studies investigating roles of non-coding RNAs in the pathogenesis of axSpA, including microRNA, lncRNA and circRNA. Transcriptomic analysis revealed that the altered levels of some microRNAs could be implicated in the inflammatory processs, ossification process, dysregulation of T cells in axSpA, such as miR-29a, Let-7i and miR-16 (58). miR-29a could target DKK-1 and GSK3b and interfere with the bone formation process, with some studies reporting that levels of miR-29a were significantly elevated in peripheral blood mononuclear cells of AS patients and could result in increased activity of osteoblasts (59). One study reported that serum levels of TUG1 were negatively correlated with CRP in ankylosing spondylitis patients (60). Despite the numerous studies in this field, many of the results were rarely replicated by subsequent studies, and it came to our notice that an unusual number of articles in this field of research were retracted. Members of the literature review team expressed concern regarding the reliability of the evidence, and combined with the many challenges in non-coding RNA testing, such as instability of RNA, various subtypes of mononuclear cells and lack of validation studies (7), this guideline determined to strongly recommend against testing of non-coding RNAs in patients with axSpA, unless high quality evidence is brought forward. This recommendation triggered debate within the core team and the voting panel. Some members of the voting panel argued that such categorical denial of the merits of non-coding RNAs would be inappropriate and that we should not easily dismiss the evidence as unreliable. It was reiterated to the voting panel that this recommendation was not trying to negate the significance of non-coding RNA in the pathogenesis of axSpA, but given the current evidence we did not encourage routine testing of non-coding RNA in clinical practice. This recommendation was sustained with an approval rate of 71.43%.

### 3.13 We conditionally recommend testing of bone turnover markers, including CTX-I and PINP, in patients with axSpA

In terms of the osteoinflammatory process, axSpA is characterized by the paradoxical disequilibrium between bone resorption and bone formation (61). Both osteoporosis and new bone formation are prominent features in axSpA, and the prevalence of vertebral fractures could be as high as 30% (62). Bone turnover markers include markers of bone absorption and markers of bone formation. Our meta-analysis confirmed that both C-terminal telopeptide of type I collagen (CTX-I) levels in the serum and the deoxypyridinoline(DPD)/creatinine ratio in the urine were significantly elevated in patients with axSpA, suggesting excessive bone absorption (**Supplementary Appendix 6** in **Supplementary Table 6**). Markers of bone formation, such as Procollagen I N-terminal peptide (PINP), could be indicators of therapeutic responses of anti-osteoporosis medication. Although evidence regarding the values of bone turnover markers in the management of axSpA was indirect, we still believe that such markers could help visualize which direction the balance of the osteoinflammatory process is tipping towards. However, matrix metalloproteinase-mediated degradation fragments of extracellular matrix, including C1M, C2M, C3M, C6M and VICM, was not included in this recommendation due to limited quality of evidence.

### 3.14 We conditionally recommend testing of sclerostin in patients with axSpA

Sclerostin is a glycoprotein produced and secreted mostly by mature osteocytes (63). It is an inhibitor of the Wnt signaling pathway, which could inhibit osteoblast-induced new bone formation (64). Moreover, sclerostin can stimulated RANKL secretion by osteocytes, thereby promoting osteoclastogenesis and bone resorption (65). Despite the heterogeneity observed in the studies investigating serum levels of sclerostin in axSpA patients, the majority of studies could confirm that serum sclerostin levels could be an indicator of bone formation activity, and patients with lower sclerostin levels were more likely to exhibit radiographic progression (66–68). We believe that this heterogeneity could be derived from the different ossification activity of the included patients. It should be noted that sclerostin was not correlated with disease activity (**Supplementary Appendix 6** in **Supplementary Table 6**). Based on the gathered evidence, this guideline conditionally recommended testing of sclerostin as an indicator of new bone formation, with an approval rate of 95.24%.

### 3.15 We conditionally recommend testing of DKK-1 in patients with axSpA

Dickkopf-1 (DKK-1) is another inhibitor of the Wnt/β-catenin signaling pathway, which could competitively combine with LRP5/6 and ultimately inhibit new bone formation (69). Previous meta-analysis concluded that lower serum DKK-1 levels could be observed in the subgroups of AS patients with increased CRP (CRP > 10 mg/L) and high mSASSS (mSASSS > 30), indicating an inverse correlation between DKK-1 and disease activity as well as radiographic progression (70). Lower DKK-1 levels could be interpreted as higher risks for radiographic progression and might require more advanced treatment. We conditionally recommend the testing of DKK-1 in patients with axSpA, and 85.71% of the voting panel agreed on this recommendation.

### 3.16 We conditionally recommend against testing of OPG/RANKL/RANK in patients with axSpA

Receptor activator of nuclear factor-kappa B ligand (RANKL) could combine with the receptor activator of nuclear factor-kappa B (RANK) on the cell surface of osteoclast precursors and mediate osteoclastogenesis, while osteoprotegerin is a soluble decoy RANKL receptor produced by osteoblasts and could inhibit bone resorption (71, 72). The OPG/RANKL/RANK system regulates the balance between bone resorption and bone formation, hence the speculation that these molecules could be potential biomarkers in axSpA. However, in the systemic literature review, despite pooled results that serum levels of OPG, RANKL, and RANKL/OPG ratio were significantly elevated in axSpA (73), we could not find evidence that the OPG/RANKL/RANK system could be a predictor of syndesmophyte formation, while studies investigating their correlation with disease activity were highly inconsistent (**Supplementary Appendix 6** in **Supplementary Table 6**). In light of the limited quality of evidence, we decided to recommend against routine testing of OPG/RANKL/RANK in patients with axSpA until more substantial evidence is brought forward. 85.71% of the voting panel agreed on this recommendation.

### 3.17 We conditionally recommend against testing of MMP-3 in patients with axSpA

Matrix metalloproteinase 3 (MMP-3) could degrade the ECM and is associated with the destruction of articular cartilage and bone (74). It was hypothesized that the up-regulated activity of MMP-3 is correlated with increased disease activity and the extent of articular damage in axSpA. The systemic literature review examined its role in disease activity and radiographic progression, and results showed significant heterogeneity in its correlation with disease activity. (**Supplementary Appendix 6** in **Supplementary Table 6**) Only two studies investigated its capacity as a predictor of radiographic progression (75, 76), while only one study found that baseline serum MMP-3 levels were significantly associated with 2-year progression of mSASSS, and MMP-3 was primarily contributory in patients who already had substantial baseline damage (76). Moreover, MMPs are also involved in the therapeutic implications in axSpA (77). It was unclear what incremental value MMP-3 could bring to the current panel of biomarkers. Based on this consideration, this guideline conditionally recommends against routine testing of MMP-3. The approval rate was 95.24%.

### 3.18 We conditionally recommend genotyping of CYP2C9 alleles before axSpA patients start medication of NSAIDs metabolized by CYP2C9, such as diclofenac, meloxicam and celecoxib

Multiple non-steroidal anti-inflammatory drugs (NSAIDs) were metabolized by CYP2C9, including diclofenac, meloxicam and celecoxib with the exception of aspirin (78). Dozens of alleles of the gene CYP2C9 have been identified, and most allelic variants of CYP2C9 would cause reductions in the enzymatic activity. Currently the most researched allelic variants include CYP2C9*2 and CYP2C9*3, which were slightly more common in Caucasians with prevalence of 12.68% and 6.88%, as compared with <1% and 3.38% in East Asians (79). In terms of pharmacokinetics, carriers of CYP2C9*2 or CYP2C9*3 were more likely to be slow metabolizers of NSAIDs with higher peak concentration and greater area under the curve (AUC) (79). Our meta-analysis confirmed that carriers of CYP2C9*2 or CYP2C9*3 were more likely to have gastrointestinal adverse reactions, more specifically upper gastrointestinal bleeding, compared with homozygotes of CYP2C9*1 (**Supplementary Appendix 6** in **Supplementary Table 6**). There was not enough evidence to suggest that variants of CYP2C9 were associated with other adverse reactions of NSAIDs, such as cardiovascular events, despite a few reports. It was also hypothesized that the variants of PTGS2, which encodes cyclooxygenase 2 (COX-2), could have an impact on the efficacy and safety of NSAIDs, but evidence is limited (80). We conditionally recommend genotyping of CYP2C9 alleles before medication of NSAIDs, and for carriers of CYP2C9*2 or CYP2C9*3 as well as patients identified as slow metabolizers of NSAIDs based on previous medical history, it is advised to start with half the lowest dose. It should be taken into consideration that since genetic testing could be expensive in some areas, patients’ values and willingness should also be considered.

### 3.19 We conditionally recommend genetyping of NAT2 alleles before axSpA patients start medication of sulfasalazine

Most of sulfasalazine is hydrolyzed in the colon into 5-aminosalicylic acid and sulfapyridine, and the latter is absorbed into blood and metabolized in the liver by N-acetyltransferase 2 (NAT2) (81). Individuals carrying the wild type gene of NAT2, namely NAT2*4 could be categorized as fast acetylator, while those carrying the mutated genes NAT2*5, 6, 7 could be categorized as slow acetylator (81). Our meta-analysis confirmed that the slow acetylators carrying the allelic variants NAT2*5, 6, 7 were at a significantly higher risk of dose-dependent adverse events, such as nausea, vomiting, dizziness, but slow acetylation was not associated with hypersensitivity-related adverse events, such as skin rash or granulocytopenia (**Supplementary Appendix 6** in **Supplementary Table 6**). Interestingly, mutations of NAT2 are very prevalent across the general population (~50%) (82). Apart from NAT2, ABCG2 is another gene reported to be associated with the safety and efficacy of sulfasalazine, but evidence is still limited (83). Based on the evidence above, we conditionally recommend genotyping of NAT2 genetic variants before medication of sulfasalazine. For slow acetylators determined through genotyping or based on previous medical history, it is advised to start with half the lowest dose of sulfasalazine, or choose different kinds of medication. It should be noted that since the adverse events associated with slow acetylation are not life-threatening and genetic testing could be expensive, patients’ willingness to avert possible adverse reactions through genetic testing should be considered.

### 3.20 We conditionally recommend measurement of antidrug antibodies in patients receiving medication of TNF-α inhibitors at the time of clinical non-responses

Measurement of antidrug antibodies (ADAbs) falls in the category of therapeutic drug monitoring (TDM). A recent clinical trial exhibited that among patients receiving maintenance therapy with infliximab, proactive TDM was more effective than treatment without TDM in sustaining disease control (84). EULAR also developed points-to-consider addressing the principles and clinical utility of TDM, pointing out that measurement of ADAbs should be considered to understand clinical non-response in the case of immunogenic biopharmaceuticals (85). Measurement of ADAbs should also be considered in the case of a hypersensitivity reaction, mainly related to infusions, but not injection-site reaction. Our meta-analysis concluded that ADAbs were significantly associated with lower drug concentrations of TNF-α inhibitors. (**Supplementary Appendix 6** in **Supplementary Table 6**) On the other hand, IL-17 inhibitors generally exhibited good immunogenicity. The incidence rate of ADAbs in secukinumab was less than 1% (86, 87). For ixekizumab, the general incidence rate of ADAbs was 9-19.4%, yet such ADAbs were not neutralizing antibodies and could not predict treatment outcomes (88). It is currently believed that ADAbs to secukinumab, ixekizumab and bimekizumab were not associated with adverse events (89). Based on the evidence above, we formulated a recommendation of ADAbs measurement in patients receiving medication of TNF-α inhibitors, but not IL-17 inhibitors, at the time of clinical non-responses. In line with the EULAR points-to-consider, proactive testing of ADAbs is not recommended.

## 4 Other biomarkers to consider

### 4.1 Metabolomic signature

Metabolomics studies in patients with axSpA have revealed significant alterations in the metabolism of amino acids, fatty acids and choline. Diagnostic panels were formulated based on the metabolomic signature of axSpA patients, with AUC as high as 0.998, but such diagnostic panels did not undergo external validation or were not verified by subsequent studies (90, 91). It was also unclear what incremental value it could bring to the currently established biomarkers. With considerations of costs, accessibility and certainty of evidence, we decided not to formulate a recommendation, but the metabolomic signature shall be revisited in the future to determine its clinical utility.

### 4.2 Gut microbiota

The diversity and abundance of gut microbiota could be explored by means of metagenomic shotgun sequencing and 16S rRNA gene sequencing. Current studies have revealed changes of a-diversity and elevated abundance of dialister, actinobacteria and clostridium, which could be associated with disease activity (92, 93). Diagnostic panels were devised, but were not validated by subsequent studies (94). Considering the costs, accessibility and certainty of evidence, we decided not to formulate a recommendation, but the potential of gut microbiota as a biomarker in axSpA shall be revisited in the future.

## 5 Discussion

This guideline puts forward recommendations for choosing biomarkers in the diagnosis and assessment of axSpA, using an evidence-based and consensus-based methodology. A total of 20 recommendations were formulated in this project. The only two strong recommendations endorsed the testing of HLA-B27 in patients suspected of axSpA, and CRP/ESR as indices of disease activity. These two recommendations represent the status quo of the clinical practice of axSpA, yet the intention of this guideline was not to maintain the status quo; it sought to push the clinical practice further and expedite the process from bench to bedside, by pooling all the evidence of the utility of biomarkers in the diagnosis and assessment of patients with axSpA. We conducted an exhaustive examination of the biomarkers which have been studied in axSpA, in terms of diagnostic utility, disease activity, radiographic progression and predicting/monitoring therapeutic responses. Based on the systemic literature review, these recommendations highlight the interpretations of the biomarkers in axSpA in the four dimensions mentioned above.

However, this guideline does not dictate clinical choices of tests on axSpA patients. Along with the two strong recommendations, the 13 conditional recommendations compose a toolkit for healthcare professionals to choose appropriate testing items from. In clinical practice, decisions should be made based on the pragmatic consideration of costs, accessibility, patients’ values and willingness in the local context, and most importantly, the objective of the tests. Studies focusing on the economic evaluations of the biomarkers were sparse, thus limiting the certainty of evidence in this project. Another issue is that local laboratory conditions might vary, causing difficulties in establishing the reference range for the biomarkers, affecting the generalizability of these recommendations. Joint efforts should be carried out to standardize the testing methodology and the reference range.

This guideline only examined some of the biomarkers that have been extensively studied. In light of the advances in research, we believe that more promising biomarkers will keep emerging and ultimately complement the toolkit we propose. Considering that evidence is still sparse regarding certain biomarkers, we decided not to formulate recommendations for the time being, such as the metabolomic signature, gut microbiota and TNC, but these biomarkers shall be revisited in the future, when more evidence becomes available.

In conclusion, this guideline formulated recommendations on biomarkers in the diagnosis and assessment of axSpA patients advising on whether, in whom, when to choose the laboratory tests and how to interpret the alterations of these biomarkers. The ultimate goal of this guideline is to stratify patients based on the information provided by the biomarkers and facilitate personalized care to patients with axSpA.
