# Supplementary material for: A guideline on biomarkers in the diagnosis and evaluation in axial spondyloarthritis
Source: Front Immunol. 2024 Oct 30;15:1394148. doi: 10.3389/fimmu.2024.1394148 (PMC11557325; doi:10.3389/fimmu.2024.1394148)
Supplement: Supplementary file 1 [file Table1.docx]

Supplementary appendix 1: Methods

Methodology overview

This guideline was developed following the Grading of Recommendations Assessment, Development, and Evaluation (GRADE) methodology (www.gradeworkinggroup.org).[1-3] Since this guideline mainly involves non-interventional tests of biomarkers in the evaluation of patients with axial spondyloarthritis (axSpA), the GRADE methodology was modified based on a previous publication.[4]

Teams involved

The core team was comprised of experts in the field of rheumatology and evidence-based medicine, including an expert in the GRADE methodology, whose main assignment was to provide counsel on the modification of the GRADE methodology regarding the non-interventional approaches, also on the process of evidence gathering and presentation. The core team (4 members) was in charge of this project and tasked with defining the scope, preliminary selection of the biomarkers, drafting the basic structure of this guideline, handing out assignments to the literature review team, managing the voting process and drafting of the manuscript.

The literature review team (10 members) received assignments from the core team and conducted systemic literature review on the topics assigned. The members of the literature review team were required to search the databases (Pubmed, Embase, Cochrane library) for pertinent articles, assess study quality, extract related data from the included studies, summarize the evidence, grade the quality of evidence and compile the evidence reports.

The voting panel consists of 21 members with expertise in rheumatology, orthopedics and laboratory medicine. Members of the voting panel were invited to provide opinions on the selection of biomarkers and scope definition. After the literature review team completed the systemic literature review, the evidence reports were presented to the voting panel at an online conference. Members of the voting panel were invited to examine the evidence reports and vote on each recommendation.

All members participating in the development of this guideline received training sessions on the GRADE methodology and its modification applied in this project. Rosters of each team could be seen in Supplementary Appendix 2.

Disclosures of conflicts of interest

Principal investigators of this guideline had no relevant conflicts of interest before the initiation of this project, while the majority of the team members in the development of this guideline declared no conflicts of interest throughout the period. Individuals employed by companies that manufactured or sold diagnostics or therapeutics were deemed ineligible to participate. In the meanwhile, individuals who had any relationship with such companies were considered conflicted. Intellectual conflicts, defined as previous publication or projects pertinent to the application of biomarkers in axSpA were exempt, under the condition that such conflicts were fully disclosed.

Scope and target audience

The scope of this guideline was mainly biomarkers in the diagnosis, disease activity evaluation, prediction of radiographic progression, and monitoring of therapeutic responses in the population of axSpA patients. Biomarkers that could be detected with blood sample, fecal sample and urine sample were considered. The target audience of this guideline includes rheumatologists and medical laboratory technicians, as well as other physicians that might come in contact with axSpA patients.

Development of the framework and clinical questions

The core team led a joint effort with the voting panel and the literature review team to devise the framework and select the preliminary set of biomarkers awaiting examination. After a round of discussion within the core team, biomarkers bearing certain clinical significance were selected from the preliminary set and entered the next stage of systemic literature review. Since this guideline mainly involved non-interventional approaches, the PICO structure does not apply to this project. This guideline was mostly concerned about the clinical significance of the biomarkers investigated, which could be stratified to the four levels: a) diagnostic utility; b) indication of disease activity; c) prediction of radiographic progression; d) monitoring of therapeutic responses.

The core team held weekly meetings with the literature review team to discuss the developments of this guideline. An online conference was convened among all the members of the core team, the voting panel and the literature review team to determine the framework of this guideline. The preliminary set of biomarkers was shown in Table 1, while biomarkers selected for systemic literature review were also shown. Clinical questions were then raised regarding each biomarker, concerning the four levels of clinical significance. Additional questions were discussed by email after this meeting.

Systemic literature review

Identifying eligible studies

In order to gather all the articles pertinent to the clinical questions, each member of the literature review team performed systemic literature searches based on the assignments each member received. All the articles were searched in the databases including Embase, Pubmed and Cochrane Library. Details of the search strategies could be seen in Supplementary Appendix 3.

Study selection

Having retrieved articles identified as potentially eligible with full text, two independent members of the literature review team screened through these articles to rule out duplicates. Another two independent reviewers went through all the eligible articles and matched each article to the clinical questions. All the manuscripts were subject to evaluation of study quality, employing the Newcastle-Ottawa Quality Assessment Scale.[4]

Data synthesis

Having selected the pertinent articles and matched the articles to each clinical question, members of the literature review team were required to conduct tabulation of study characteristics, quality and effects. Reviewers also extracted data from the included studies when applicable, and used statistical methods for exploring differences between studies and combining the effects. The data extracted were stratified into the four levels: a) diagnostic utility; b) indication of disease activity; c) prediction of radiographic progression; d) monitoring of therapeutic responses. The R platform (The R Project for Statistical Computing, Vienna, Austria), version 3.6.3 was employed to calculate the pooled estimates of the statistics, using the R package “meta”. All the pooled estimates were reported with 95% confidence intervals. The majority of the included studies were observational studies, and data pooling was conducted mostly on case-control studies investigating the differences of a certain biomarker between axSpA patients and healthy controls, but also the power of a certain biomarker in the prediction of radiographic progression.

Evidence report drafting

Two independent reviewers were tasked with evaluating the quality of evidence for each biomarker, by means of the GRADE quality assessment criteria.[1] Once disagreements occurred between the two reviewers, the rest of the literature review team reviewed the evidence and settle the disagreements. The summary of findings tables were presented in the evidence reports listed in Supplementary Appendix 6. After the compilation, the evidence reports were submitted to the core team for further evaluation. One member of the literature review team collected comments from the core team, and revised the evidence reports.

Moving from evidence to recommendations

Since this guideline mainly addressed non-interventional approaches, namely the non-invasive testing of the biomarkers, the GRADE methodology was not fully applicable to this guideline. Instead, certain modifications were applied to the GRADE methodology under the advice of Professor Kehu Yang. A recommendation should be made based on the comprehensive consideration of cost, accessibility and the clinical significance of the biomarkers, together with the quality of evidence. The overarching principle is that a recommendation should only be made when a certain biomarker bears clinical information, which could assist in the evaluation of diagnosis, disease activity, radiographic progression and therapeutic responses, with relatively low costs and easy accessibility.

Even if a certain biomarker proves to be significantly up-regulated or down-regulated in axSpA patients, it does not necessarily equate with a recommendation. A recommendation is only formulated when this biomarker could inform rheumatologists about diagnosis, disease activity, tendencies of radiographic progression or therapeutic responses. For biomarkers with potential applications in diagnosis, it is more relevant to investigate its incremental value to the routine HLA-B27 and MRI examination. A mere increase or decrease of the biomarker is not sufficient to convince the panels, unless it could provide additional sensitivity or specificity to the routine procedure. For biomarkers as potential indicators of disease activity, not only did the literature review team have to prove its reliability and robustness, they had to prove that this biomarker is a valuable addition to the routine CRP/ESR tests. For biomarkers associated with radiographic progression, it had to be proved that the the baseline level of this biomarker could be a predictor of radiographic progression, which could educate treatment options. For biomarkers related to therapeutic responses, this guideline is interested in how this biomarker could reflect safety or efficacy or the medication.

Moreover, the literature review team was also tasked with the exploration of heterogeneity of the studies. Members had to assess the risk of publication bias as well as related biases, and determine whether the evidence should be trusted. If the evidence exhibited conspicuous inconsistency across different studies, the reliability of the evidence could be questioned and such heterogeneity could affect the strength of the evidence and even the strength of the recommendation. The recommendations will be formulated based on comprehensive consideration of all the issues above.

Consensus building

Once all the evidence reports had been compiled, the evidence reports were sent to the voting panel through email. After careful examination of the evidence reports, each member of the voting panel received a ballot through email and was invited to fill out the ballot, regarding the direction and strength of each recommendation. One member of the literature review team collected the ballots and calculated the approval ratings and strengths of each recommendation. Only one round of voting was held, and a 70% consensus was regarded necessary for a recommendation to be included in the guideline; if the 70% threshold was not fulfilled, this recommendation was discarded from the guideline.
