# Supplementary material for: A guideline on biomarkers in the diagnosis and evaluation in axial spondyloarthritis
Source: Front Immunol. 2024 Oct 30;15:1394148. doi: 10.3389/fimmu.2024.1394148 (PMC11557325; doi:10.3389/fimmu.2024.1394148)
Supplement: Supplementary file 2 [file Table2.docx]

**SUPPLEMENTARY APPENDIX 2: Panel/Teams Involved**

**Core Leadership Team**

Jieruo Gu, MD, PhD (the Third Affiliated Hospital of Sun Yat-Sen University, Guangzhou, China; Project Co-PI), Kehu Yang, MD, PhD (Lanzhou University, Lanzhou, China; Project Co-PI), Yan Shen, MD, PhD (National Laboratory of Medical Molecular Biology, Institute of Basic Medical Sciences, Chinese Academy of Medical Science and Peking Union Medical College, Tsinghua University, Beijing, China; Project Co-PI)

**Voting Panel**

Ou Jin (the Third Affiliated Hospital of Sun Yat-Sen University, Guangzhou, China), Lijun Wu (People’s Hospital of Xinjiang Uygur Autonomous Region, Urumchi, China), Dongbao Zhao (Changhai Hospital, Shanghai, China), Dongyi He (Shanghai Guanghua hospital of intergrated traditional Chinese and western medicine, Shanghai, China), Shanzhi He (Zhongshan City People’s Hospital, Zhongshan, China), Wenhui Huang (the Second Affiliated Hospital of Guangzhou Medical University, Guangzhou, China), Shanhui Ye (the First Affiliated Hospital of Guangzhou Medical University, Guangzhou, China), Huiqiong Zhou (the Fourth Central Hospital Chinese PLA Medical School, Xi’an, China), Qing Lyu (the Seventh Affiliated Hospital of Sun Yat-Sen University, Guangzhou, China), Jinyu Wu (the First Affiliated Hospital of Guangxi university of Chinese medicine, Nanning, China), Yongfu Wang (the First Affiliated Hospital of Baotou medical college, Baotou, China), Shengyun Liu (the First Affiliated Hospital of Zhengzhou University, Zhengzhou, China), Zhenbin Li (Bethune International Peace Hospital, Shijiazhuang, China), Zhiming Tan (Huizhou central hospital, Huizhou, China), Chiduo Xu (the Second People‘s Hospital of Shenzhen city, Shenzhen, China), Youlian Wang (Jiangxi Province People’s Hospital, Nanchang, China), Donghui Zheng (Sun Yat-Sen Memorial Hospital, Sun Yat-Sen University, Guangzhou, China), Feng Zhan (Hainan General Hospital, Hainan affiliated Hospital of Hainan Medical University, Haikou, China), Kun Wang (the Third Affiliated Hospital of Sun Yat-Sen University, Guangzhou, China), Bin Liu ((the Third Affiliated Hospital of Sun Yat-Sen University, Guangzhou, China), Changsong Lin (the First Affiliated Hospital of Guangzhou university of Chinese medicine, Guangzhou, China)

**Literature Review Team**

Dong Liu (the Third Affiliated Hospital of Sun Yat-Sen University, Guangzhou, China), Ya Xie (the Third Affiliated Hospital of Sun Yat-Sen University, Guangzhou, China), Liudan Tu (the Third Affiliated Hospital of Sun Yat-Sen University, Guangzhou, China), Xianghui Wen (Shenzhen Immunological medicine translational research institute (Longhua), Shenzhen, China), Budian Liu (the Third Affiliated Hospital of Sun Yat-Sen University, Guangzhou, China), Mingcan Yang (the Third Affiliated Hospital of Sun Yat-Sen University, Guangzhou, China), Xinyu Wu (the Third Affiliated Hospital of Sun Yat-Sen University, Guangzhou, China), Xiqing Luo (the Third Affiliated Hospital of Sun Yat-Sen University, Guangzhou, China), Liuzhong Zhou (the Third Affiliated Hospital of Sun Yat-Sen University, Guangzhou, China), Shenghui Wen (the Seventh Affiliated Hospital of Sun Yat-Sen University, Shenzhen, China), Ya Wen (the Seventh Affiliated Hospital of Sun Yat-Sen University, Guangzhou, China), Jiayun Wu (the Seventh Affiliated Hospital of Sun Yat-Sen University, Guangzhou, China), Zetao Liao (the Third Affiliated Hospital of Sun Yat-Sen University, Guangzhou, China)
