# Supplementary material for: A guideline on biomarkers in the diagnosis and evaluation in axial spondyloarthritis
Source: Front Immunol. 2024 Oct 30;15:1394148. doi: 10.3389/fimmu.2024.1394148 (PMC11557325; doi:10.3389/fimmu.2024.1394148)
Supplement: Supplementary file 3 [file Table3.docx]

**SUPPLEMENTARY APPENDIX 3: Search strategies**

**Search Strategies**

| **Syntax Guide for PubMed** |
| --- |
| MeSH = Medical Subject Heading |
| All Fields = Word appears in title, abstract, keyword, author, editor, publisher, journal, etc. |
| Supplementary Concept = Includes chemical, protocol, disease or organism terms. Synonyms to the supplementary concepts will automatically map when tagged with [nm]. This field was implemented in mid-1980; however, many chemical names are searchable as MeSH terms before that date. |
| **Additional Syntax Guide for Embase** |
| Exp = A command to retrieve all narrower subject headings (MeSH) |
| **Additional Syntax Guide for Cochrane Library** |
| ti,ab,kw = Word appears in title, abstract or keyword field of record; word variations are searched in title, abstract, keyword search |
| **Boolean Operators** |
| AND = retrieves results that include all the search terms |
| OR = retrieves results that include at least one of the search terms |
| NOT = excludes the retrieval of terms from the search |

**Question 1: HLA-B27**

**Database: PubMed**

Search strategies:

| #1 | ((((((((((((((((((((((((((((((Low Back Pain[MeSH Terms]) OR (Back Pain, Low)) OR (Back Pains, Low)) OR (Low Back Pains)) OR (Pain, Low Back)) OR (Pains, Low Back)) OR (Lumbago)) OR (Lower Back Pain)) OR (Back Pain, Lower)) OR (Back Pains, Lower)) OR (Lower Back Pains)) OR (Pain, Lower Back)) OR (Pains, Lower Back)) OR (Low Back Ache)) OR (Ache, Low Back)) OR (Aches, Low Back)) OR (Back Ache, Low)) OR (Back Aches, Low)) OR (Low Back Aches)) OR (Low Backache)) OR (Backache, Low)) OR (Backaches, Low)) OR (Low Backaches)) OR (Low Back Pain, Postural)) OR (Postural Low Back Pain)) OR (Low Back Pain, Posterior Compartment)) OR (Low Back Pain, Recurrent)) OR (Recurrent Low Back Pain)) OR (Low Back Pain, Mechanical)) OR (Mechanical Low Back Pain) |  |
| --- | --- | --- |
| #2 | (((((((HLA-B27 Antigen[MeSH Terms]) ) OR (HLA B27 Antigen)) OR (Antigen, HLA-B27)) OR (HLA-B27)) OR (HLA Class I Histocompatibility Antigen, B-27 alpha Chain)) OR (HLA Class I Histocompatibility Antigen, B 27 alpha Chain) |  |
| #3 | #1 AND #2 |  |
| #4 | "spondylarthritis"[MeSH Terms] OR "spondylarthritis"[All Fields] OR "spondyloarthritis"[All Fields] |  |
| #5 | "spondylitis, ankylosing"[MeSH Terms] OR ("spondylitis"[All Fields] AND "ankylosing"[All Fields]) OR "ankylosing spondylitis"[All Fields] OR ("ankylosing"[All Fields] AND "spondylitis"[All Fields]) |  |
| #6 | #4 OR #5 |  |
| #7 | #3 AND #6 |  |
| #8 | #7 AND 1976/01/01:2022/11/21[Date - Publication] |  |
| #9 | #8 AND "english"[Language] |  |
| #10 | ((((Diagnosis[MeSH Terms]) ) OR (Diagnoses)) OR (Diagnose) |  |
| #11 | #9 AND #10 |  |
| #12 | (disease activity[MeSH Terms]) OR (BASDAI) |  |
| #13 | #9 AND #14 |  |
| #14 | #9 AND (radiographic progression[MeSH Terms]) |  |
| #15 | ((((((therapeutic response[MeSH Terms]) OR (treatment response[MeSH Terms])) OR (treament effects[MeSH Terms])) OR (Therapeutic Effects)) OR (Effects, Therapeutic)) OR (Therapeutic Effect)) OR (Effect, Therapeutic) |  |
| #16 | #9 AND #17 |  |
| #17 | #11 OR #13 or #16 OR #18 | **Q1：**  **179 results** |

**Database: Embase**

Search strategies:

| #1 | 'low back pain'/exp OR 'low back pain' |  |
| --- | --- | --- |
| #2 | 'hla b27 antigen'/exp OR 'hla b27 antigen' |  |
| #3 | #1 and #2 |  |
| #4 | 'spondylarthritis'/exp OR spondylarthritis |  |
| #5 | 'ankylosing spondylitis'/exp OR 'ankylosing spondylitis' |  |
| #6 | #4 OR #5 |  |
| #7 | #3 AND #6 |  |
| #8 | #5 AND #6 AND [01-01-1976]/sd NOT [22-11-2022]/sd AND [english]/lim |  |
| #9 | 'diagnosis'/exp OR diagnosis |  |
| #10 | 'disease activity'/exp OR 'disease activity' |  |
| #11 | 'bath ankylosing spondylitis disease activity index'/exp OR 'bath ankylosing spondylitis disease activity index' |  |
| #12 | 'radiographic progression'/exp OR 'radiographic progression' OR (radiographic AND ('progression'/exp OR progression)) |  |
| #13 | 'treatment response'/exp OR 'treatment response' |  |
| #14 | 'therapy effect'/exp OR 'therapy effect' |  |
| #15 | #9 OR #10 OR #11 OR #12 OR #13 OR #14 |  |
| #16 | #8 AND #15 | **Q1：**  **377 results** |

**Database: Cochrane Library**

Search strategies:

| #1 | MeSH descriptor: [Low Back Pain] explode all trees |  |
| --- | --- | --- |
| #2 | (Back Pain, Low or Back Pains, Low or Low Back Pains or Pain, Low Back or Pains, Low Back or Lumbago or Lower Back Pain or Back Pain, Lower or Back Pains, Lower or Lower Back Pains or Pain, Lower Back or Pains, Lower Back or Low Back Ache or Ache, Low BackAches, Low Back or Back Ache, Low or Back Aches, Low or Low Back Aches or Low Backache or Backache, Low or Backaches, Low or Low Backaches or Low Back Pain, Postural or Postural Low Back Pain or Low Back Pain, Posterior Compartment or Low Back Pain, Recurrent or Recurrent Low Back Pain or Low Back Pain, Mechanical or Mechanical Low Back Pain):ti,ab,kw |  |
| #3 | #1 OR #2 |  |
| #4 | MeSH descriptor: [HLA-B27 Antigen] explode all trees |  |
| #5 | (Antigen, HLA-B27 or HLA B27 Antigen or HLA-B27 or HLA Class I Histocompatibility or Antigen, B-27 alpha Chain or HLA Class I Histocompatibility Antigen, B 27 alpha Chain):ti,ab,kw |  |
| #6 | #4 or #5 |  |
| #7 | #3 and #6 |  |
| #8 | #7 with Cochrane Library publication date from Jan 1976 to Nov 2022 |  |
| #9 | MeSH descriptor: [Spondylitis, Ankylosing] explode all trees |  |
| #10 | (Spondyloarthritis Ankylopoietica or Spondyloarthritides, Ankylosing or Spondylitis Ankylopoietica or Ankylosing Spondyloarthritides or Ankylosing Spondylitis or Spondyloarthritis, Ankylosing or Spondylarthritis, Ankylosing or Marie-Struempell Disease or Bechterew Disease or Spondylarthritides, Ankylosing or Ankylosing Spondylarthritides or Ankylosing Spondyloarthritis or Bechterews Disease or Rheumatoid Spondylitis or Marie Struempell Disease or Bechterew's Disease or Ankylosing Spondylarthritis or Spondylarthritis Ankylopoietica or Spondylitis, Rheumatoid):ti,ab,kw |  |
| #11 | #9 or #10 |  |
| #12 | MeSH descriptor: [Spondylarthritis] explode all trees |  |
| #13 | (Spinal Arthritis or Spinal Arthritides or Spondylarthritides or Arthritis, Spinal):ti,ab,kw |  |
| #14 | #12 or #13 |  |
| #15 | #11 or #14 |  |
| #16 | #8 and #15 |  |
| #17 | MeSH descriptor: [Diagnosis] explode all trees |  |
| #18 | (Diagnoses or Diagnose or Diagnoses, Postmortem or Postmortem Diagnoses or Postmortem Diagnosis or Diagnosis, Postmortem or Examinations and Diagnoses or Examination and Diagnoses or Diagnoses and Examination or Diagnoses and Examinations or Antemortem Diagnosis or Diagnoses, Antemortem or Diagnosis, Antemortem or Antemortem Diagnoses):ti,ab,kw |  |
| #19 | #17 or #18 |  |
| #20 | (disease activity):ti,ab,kw |  |
| #21 | (BASDAI):ti,ab,kw |  |
| #22 | (radiographic progression):ti,ab,kw |  |
| #23 | (therapeutic response):ti,ab,kw |  |
| #24 | (treatment response):ti,ab,kw |  |
| #25 | (treament effect):ti,ab,kw |  |
| #26 | #19 or #20 or #21 or #22 or #23 or #24 or #25 |  |
| #27 | #16 and #26 | **Q1:**  **13 results** |

**Question 2 : HLA-B27 subtypes**

**Database: PubMed**

Search strategies:

| #1 | ((((((((((((((((((("Spondylitis, Ankylosing"[Mesh]) OR (Bechterew's Disease[All Fields])) OR (Bechterews Disease[All Fields])) OR (Marie-Struempell Disease[All Fields])) OR (Marie Struempell Disease[All Fields])) OR (Spondylarthritis Ankylopoietica[All Fields])) OR (Spondyloarthritis Ankylopoietica[All Fields])) OR (Ankylosing Spondylitis[All Fields])) OR (Ankylosing Spondylarthritis[All Fields])) OR (Ankylosing Spondylarthritides[All Fields])) OR (Spondylarthritides, Ankylosing[All Fields])) OR (Spondylarthritis, Ankylosing[All Fields])) OR (Ankylosing Spondyloarthritis[All Fields])) OR (Ankylosing Spondyloarthritides[All Fields])) OR (Spondyloarthritides, Ankylosing[All Fields])) OR (Spondyloarthritis, Ankylosing[All Fields])) OR (Spondylitis Ankylopoietica[All Fields])) OR (Bechterew Disease[All Fields])) OR (Rheumatoid Spondylitis[All Fields])) OR (Spondylitis, Rheumatoid[All Fields]) |  |
| --- | --- | --- |
| #2 | (((("Spondylarthritis"[Mesh]) OR (Spondylarthritides)) OR (Spinal Arthritis)) OR (Spinal Arthritides)) OR (Arthritis, Spinal) |  |
| #3 | #1 OR #2 |  |
| #4 | ((((("HLA-B27 Antigen"[Mesh]) OR (Antigen, HLA-B27[All Fields])) OR (HLA B27 Antigen[All Fields])) OR (HLA-B27[All Fields])) OR (HLA Class I Histocompatibility Antigen, B-27 alpha Chain[All Fields])) OR (HLA Class I Histocompatibility Antigen, B 27 alpha Chain[All Fields]) |  |
| #5 | (((subtype) OR (subtyping)) OR (subtypes)) OR ((alleles) OR (allele)) |  |
| #6 | #3 AND #4 AND #5 | **Q2:**  **730 results** |

**Database: Embase**

Search strategies:

| #1 | 'spondylarthritis'/exp OR spondylarthritis |  |
| --- | --- | --- |
| #2 | 'ankylosing spondylitis'/exp OR 'ankylosing spondylitis' |  |
| #3 | #1 OR #2 |  |
| #4 | 'hla b27 antigen'/exp OR 'antigen b 27' OR 'antigen hla 27b' OR 'hl a 27b antigen' OR 'hl a b27' OR 'hla 27b antigen' OR 'hla b27' OR 'hla-b27 antigen' OR 'human lymphocyte antigen 27b' |  |
| #5 | subtype OR subtyes OR subtyping |  |
| #6 | #3 AND #4 AND #5 | **Q2:**  **301 results** |

**Database: Cochrane Library**

Search strategies:

| #1 | ("spondylarthritis"):ti,ab,kw |  |
| --- | --- | --- |
| #2 | ("ankylosing spondylitis"):ti,ab,kw |  |
| #3 | #1 OR #2 |  |
| #4 | ("hla b27 "):ti,ab,kw |  |
| #5 | (subtype or subtypes or subtyping):ti,ab,kw |  |
| #6 | #3 AND #4 AND #5 | **Q2:**  **3 results** |

**Question 3: Genes**

**Database: PubMed**

Search strategies:

| #1 | ((((((((((((((((((("Spondylitis, Ankylosing"[Mesh]) OR (Bechterew's Disease[All Fields])) OR (Bechterews Disease[All Fields])) OR (Marie-Struempell Disease[All Fields])) OR (Marie Struempell Disease[All Fields])) OR (Spondylarthritis Ankylopoietica[All Fields])) OR (Spondyloarthritis Ankylopoietica[All Fields])) OR (Ankylosing Spondylitis[All Fields])) OR (Ankylosing Spondylarthritis[All Fields])) OR (Ankylosing Spondylarthritides[All Fields])) OR (Spondylarthritides, Ankylosing[All Fields])) OR (Spondylarthritis, Ankylosing[All Fields])) OR (Ankylosing Spondyloarthritis[All Fields])) OR (Ankylosing Spondyloarthritides[All Fields])) OR (Spondyloarthritides, Ankylosing[All Fields])) OR (Spondyloarthritis, Ankylosing[All Fields])) OR (Spondylitis Ankylopoietica[All Fields])) OR (Bechterew Disease[All Fields])) OR (Rheumatoid Spondylitis[All Fields])) OR (Spondylitis, Rheumatoid[All Fields]) |  |
| --- | --- | --- |
| #2 | (((("Spondylarthritis"[Mesh]) OR (Spondylarthritides)) OR (Spinal Arthritis)) OR (Spinal Arthritides)) OR (Arthritis, Spinal) |  |
| #3 | #1 OR #2 |  |
| #4 | ((((((((((Polymorphism) OR (Polymorphisms, Genetic)) OR (Genetic Polymorphism)) OR (Genetic Polymorphisms)) OR (Gene Polymorphism)) OR (Gene Polymorphisms)) OR (Polymorphism, Gene)) OR (Polymorphisms, Gene)) OR (Polymorphism (Genetics))) OR (Polymorphisms (Genetics))) OR (Polymorphism[MeSH Terms]) |  |
| #5 | (((((((((("Major Histocompatibility Complex"[Mesh]) OR (Complex, Major Histocompatibility)) OR (Complices, Major Histocompatibility)) OR (Histocompatibility Complex, Major)) OR (Histocompatibility Complices, Major)) OR (Major Histocompatibility Complices)) OR (Histocompatibility Complex)) OR (Complex, Histocompatibility)) OR (Complices, Histocompatibility)) OR (Histocompatibility Complices)) OR (mhc) |  |
| #6 | HLA allele |  |
| #7 | ((heredity) OR (genetics)) OR (genetic) |  |
| #8 | #4 OR #5 OR #6 OR #7 |  |
| #9 | #3 AND #8 | **Q3:**  **5,371 results** |

**Database: Embase**

Search strategies:

| #1 | 'spondylarthritis'/exp OR spondylarthritis |  |
| --- | --- | --- |
| #2 | 'ankylosing spondylitis'/exp OR 'ankylosing spondylitis' |  |
| #3 | #1 OR #2 |  |
| #4 | ('hla'/exp OR hla) AND ('allele'/exp OR allele) |  |
| #5 | 'major histocompatibility complex'/exp OR 'major histocompatibility complex' OR 'mhc' |  |
| #6 | #4 OR #5 |  |
| #7 | #3 AND #6 | **Q3:**  **1,899 results** |

**Database: Cochrane Library**

Search strategies:

| #1 | ("spondylarthritis"):ti,ab,kw |  |
| --- | --- | --- |
| #2 | ("ankylosing spondylitis"):ti,ab,kw |  |
| #3 | #1 OR #2 |  |
| #4 | (mhc):ti,ab,kw OR (hla):ti,ab,kw" |  |
| #5 | #3 AND #4 | **Q3:**  **165 results** |

**Database: CKNI**

Search strategies:

| #1 | 关键词=强直性脊柱炎 |  |
| --- | --- | --- |
| #2 | 关键词=HLA-B |  |
| #3 | 关键词=HLA-DRB1 |  |
| #4 | #2 OR #3 |  |
| #5 | #1 AND #4 | **Q3:**  **248 results** |

**Question 4: Antibodies, including anti-CD74 antibodies, anti-sclerostin and anti-noggin antibodies, antibodies againts microbial targets**

**Database: PubMed**

Search strategies:

| #1 | "spondylitis, ankylosing"[MeSH Terms] OR ("spondylitis"[All Fields] AND "ankylosing"[All Fields]) OR "ankylosing spondylitis"[All Fields] OR ("ankylosing"[All Fields] AND "spondylitis"[All Fields]) |  |
| --- | --- | --- |
| #2 | "spondylarthritis"[MeSH Terms] OR "spondylarthritis"[All Fields] OR "spondyloarthritis"[All Fields] |  |
| #3 | #1 OR #2 |  |
| #4 | "CD74"[All Fields] |  |
| #5 | "anti-protein"[All Fields] AND ("phosphatase s"[All Fields] OR "phosphoric monoester hydrolases"[MeSH Terms] OR ("phosphoric"[All Fields] AND "monoester"[All Fields] AND "hydrolases"[All Fields]) OR "phosphoric monoester hydrolases"[All Fields] OR "phosphatase"[All Fields] OR "phosphatases"[All Fields]) AND "magnesium-dependent"[All Fields] AND "1A"[All Fields] |  |
| #6 | "noggin"[All Fields] AND ("sclerostin"[All Fields] OR "sclerostin s"[All Fields]) |  |
| #7 | "antibodies, bacterial"[MeSH Terms] OR ("antibodies"[All Fields] AND "bacterial"[All Fields]) OR "bacterial antibodies"[All Fields] OR ("bacterial"[All Fields] AND "antibodies"[All Fields]) |  |
| #8 | "antibodies, antinuclear"[MeSH Terms] OR ("antibodies"[All Fields] AND "antinuclear"[All Fields]) OR "antinuclear antibodies"[All Fields] OR ("antinuclear"[All Fields] AND "antibodies"[All Fields]) |  |
| #9 | "antibodies, antineutrophil cytoplasmic"[MeSH Terms] OR ("antibodies"[All Fields] AND "antineutrophil"[All Fields] AND "cytoplasmic"[All Fields]) OR "antineutrophil cytoplasmic antibodies"[All Fields] OR ("anti"[All Fields] AND "neutrophil"[All Fields] AND "cytoplasmic"[All Fields] AND "antibodies"[All Fields]) OR "anti neutrophil cytoplasmic antibodies"[All Fields]) |  |
| #10 | #4 OR #5 OR #6 OR #7 OR #8 OR #9 |  |
| #11 | #3 OAND #10 | **Q4:**  **381 results** |

**Database: Embase**

Search strategies:

| #1 | 'spondylarthritis'/exp OR spondylarthritis |  |
| --- | --- | --- |
| #2 | 'ankylosing spondylitis'/exp OR 'ankylosing spondylitis' |  |
| #3 | #1 OR #2 |  |
| #4 | 'bacterial antibodies' OR (bacterial AND ('antibodies'/exp OR antibodies)) |  |
| #5 | 'anti-neutrophil cytoplasmic antibodies' OR ('anti neutrophil' AND cytoplasmic AND ('antibodies'/exp OR antibodies)) |  |
| #6 | ('noggin'/exp OR noggin) AND ('sclerostin'/exp OR sclerostin) |  |
| #7 | CD74 |  |
| #8 | 'protein phosphatase magnesium-dependent 1a' OR (('protein'/exp OR protein) AND ('phosphatase'/exp OR phosphatase) AND 'magnesium dependent' AND 1a) |  |
| #9 | 'antinuclear antibodies' OR (antinuclear AND ('antibodies'/exp OR antibodies)) |  |
| #10 | #4 OR #5 OR #6 OR #7 OR #8 OR #9 |  |
| #11 | #3 AND #10 |  |
| #12 | #3 AND #10 AND [article]/lim |  |
| #13 | #3 AND #10 AND [article]/lim AND [english]/lim | **Q4:**  **765 results** |

**Question 5: CRP**

**Database: PubMed**

Search strategies:

| #1 | ((((((((((((((((((("Spondylitis, Ankylosing"[Mesh]) OR (Bechterew's Disease[All Fields])) OR (Bechterews Disease[All Fields])) OR (Marie-Struempell Disease[All Fields])) OR (Marie Struempell Disease[All Fields])) OR (Spondylarthritis Ankylopoietica[All Fields])) OR (Spondyloarthritis Ankylopoietica[All Fields])) OR (Ankylosing Spondylitis[All Fields])) OR (Ankylosing Spondylarthritis[All Fields])) OR (Ankylosing Spondylarthritides[All Fields])) OR (Spondylarthritides, Ankylosing[All Fields])) OR (Spondylarthritis, Ankylosing[All Fields])) OR (Ankylosing Spondyloarthritis[All Fields])) OR (Ankylosing Spondyloarthritides[All Fields])) OR (Spondyloarthritides, Ankylosing[All Fields])) OR (Spondyloarthritis, Ankylosing[All Fields])) OR (Spondylitis Ankylopoietica[All Fields])) OR (Bechterew Disease[All Fields])) OR (Rheumatoid Spondylitis[All Fields])) OR (Spondylitis, Rheumatoid[All Fields]) |  |
| --- | --- | --- |
| #2 | (((("Spondylarthritis"[Mesh]) OR (Spondylarthritides)) OR (Spinal Arthritis)) OR (Spinal Arthritides)) OR (Arthritis, Spinal) |  |
| #3 | #1 OR #2 |  |
| #4 | (((C-reactive protein) OR (CRP)) OR (C-reactive protein,CRP))OR (C-reactive protein[MeSH Terms]) |  |
| #5 | #3 AND #4 | **Q5:**  **2,342 results** |

**Database: Embase**

Search strategies:

| #1 | ankylosing spondylitis.mp. or ankylosing spondylitis/ |  |
| --- | --- | --- |
| #2 | ankylosing spondylitis/ or spondylarthritis/ |  |
| #3 | Spondylitis, Ankylosing.mp. or ankylosing spondylitis/ |  |
| #4 | #1 or #2 or #3 |  |
| #5 | C-reactive protein.mp. or C reactive protein/ |  |
| #6 | CRP.mp. or C reactive protein/ |  |
| #7 | #5 or #6 |  |
| #8 | #4 and #7 | **Q5:**  **6,994 results** |

**Database: Cochrane Library**

Search strategies:

| #1 | (spondylarthritis):ti,ab,kw (Word variations have been searched) |  |
| --- | --- | --- |
| #2 | ankylosing spondylitis |  |
| #3 | #1 OR #2 |  |
| #4 | C-reactive protein |  |
| #5 | CRP |  |
| #6 | #4 OR #5 |  |
| #7 | #3 AND #6 | **Q5:**  **783 results** |

**Question 6: ESR**

**Database: PubMed**

Search strategies:

| #1 | "spondylarthritis"[MeSH Terms] OR "spondylarthritis"[All Fields] OR "spondyloarthritis"[All Fields] |  |
| --- | --- | --- |
| #2 | "spondylitis, ankylosing"[MeSH Terms] OR ("spondylitis"[All Fields] AND "ankylosing"[All Fields]) OR "ankylosing spondylitis"[All Fields] OR ("ankylosing"[All Fields] AND "spondylitis"[All Fields]) |  |
| #3 | #1 OR #2 |  |
| #4 | "esr"[All Fields] OR "erythrocyte sedimentation rate"[All Fields] |  |
| #5 | #3 AND #4 |  |
| #6 | "disease activity"[All Fields] OR "radiographic progression"[All Fields] OR "adalimumab"[All Fields] OR "etanercept"[All Fields] OR "infliximab"[All Fields] OR "golimumab"[All Fields] OR "certolizumab"[All Fields] OR "secukinumab"[All Fields] |  |
| #7 | #5 AND #6 | **Q6:**  **908 results** |

**Database: Embase**

Search strategies:

| #1 | 'spondylarthritis' |  |
| --- | --- | --- |
| #2 | 'ankylosing spondylitis' |  |
| #3 | #1 OR #2 |  |
| #4 | 'ESR' OR 'erythrocyte sedimentation rate' |  |
| #5 | #3 AND #4 |  |
| #6 | 'disease activity' OR 'radiographic progression' OR 'adalimumab' OR 'etanercept' OR 'infliximab' OR 'golimumab' OR 'certolizumab' OR 'secukinumab' |  |
| #7 | #5 AND #6 | **Q6:**  **3,146 results** |

**Database: Cochrane Library**

Search strategies:

| #1 | ("spondyloarthritis"):ti,ab,kw |  |
| --- | --- | --- |
| #2 | ("ankylosing spondylitis"):ti,ab,kw |  |
| #3 | #1 OR #2 |  |
| #4 | (ESR):ti,ab,kw |  |
| #5 | ("erythrocyte sedimentation rate"):ti,ab,kw |  |
| #6 | #4 OR #5 |  |
| #7 | #3 AND #6 | **Q6:**  **259 results** |

**Question 7**: **SAA**

**Database: PubMed**

Search strategies:

| #1 | ((((((((((((((((((("Spondylitis, Ankylosing"[Mesh]) OR (Bechterew's Disease[All Fields])) OR (Bechterews Disease[All Fields])) OR (Marie-Struempell Disease[All Fields])) OR (Marie Struempell Disease[All Fields])) OR (Spondylarthritis Ankylopoietica[All Fields])) OR (Spondyloarthritis Ankylopoietica[All Fields])) OR (Ankylosing Spondylitis[All Fields])) OR (Ankylosing Spondylarthritis[All Fields])) OR (Ankylosing Spondylarthritides[All Fields])) OR (Spondylarthritides, Ankylosing[All Fields])) OR (Spondylarthritis, Ankylosing[All Fields])) OR (Ankylosing Spondyloarthritis[All Fields])) OR (Ankylosing Spondyloarthritides[All Fields])) OR (Spondyloarthritides, Ankylosing[All Fields])) OR (Spondyloarthritis, Ankylosing[All Fields])) OR (Spondylitis Ankylopoietica[All Fields])) OR (Bechterew Disease[All Fields])) OR (Rheumatoid Spondylitis[All Fields])) OR (Spondylitis, Rheumatoid[All Fields]) |  |
| --- | --- | --- |
| #2 | (((("Spondylarthritis"[Mesh]) OR (Spondylarthritides)) OR (Spinal Arthritis)) OR (Spinal Arthritides)) OR (Arthritis, Spinal) |  |
| #3 | #1 OR #2 |  |
| #4 | "serum amyloid a protein"[MeSH Terms] OR "serum amyloid a protein"[All Fields] |  |
| #5 | #3 AND #4 | **Q7:**  **44 results** |

**Database: Embase**

Search strategies:

| #1 | 'spondylarthritis'/exp OR spondylarthritis |  |
| --- | --- | --- |
| #2 | 'ankylosing spondylitis'/exp OR 'ankylosing spondylitis' |  |
| #3 | #1 OR #2 |  |
| #4 | 'serum amyloid a' |  |
| #5 | #3 AND #4 | **Q7:**  **144 results** |

**Database: Cochrane Library**

Search strategies:

| #1 | (spondylarthritis) OR ("ankylosing spondylitis") OR ("ankylosing spondylarthritis") OR ("spondylitis ankylopoietica") OR ("ankylosing spondyloarthritides") |  |
| --- | --- | --- |
| #2 | serum amyloid a protein |  |
| #3 | #1 and #2 | **Q7:**  **4 results** |

**Question 8:** **Adipokines, including leptin, adiponectin and resistin**

**Database: PubMed**

Search strategies:

| #1 | ((((((((((((((((((("Spondylitis, Ankylosing"[Mesh]) OR (Bechterew's Disease[All Fields])) OR (Bechterews Disease[All Fields])) OR (Marie-Struempell Disease[All Fields])) OR (Marie Struempell Disease[All Fields])) OR (Spondylarthritis Ankylopoietica[All Fields])) OR (Spondyloarthritis Ankylopoietica[All Fields])) OR (Ankylosing Spondylitis[All Fields])) OR (Ankylosing Spondylarthritis[All Fields])) OR (Ankylosing Spondylarthritides[All Fields])) OR (Spondylarthritides, Ankylosing[All Fields])) OR (Spondylarthritis, Ankylosing[All Fields])) OR (Ankylosing Spondyloarthritis[All Fields])) OR (Ankylosing Spondyloarthritides[All Fields])) OR (Spondyloarthritides, Ankylosing[All Fields])) OR (Spondyloarthritis, Ankylosing[All Fields])) OR (Spondylitis Ankylopoietica[All Fields])) OR (Bechterew Disease[All Fields])) OR (Rheumatoid Spondylitis[All Fields])) OR (Spondylitis, Rheumatoid[All Fields]) |  |
| --- | --- | --- |
| #2 | (((("Spondylarthritis"[Mesh]) OR (Spondylarthritides)) OR (Spinal Arthritis)) OR (Spinal Arthritides)) OR (Arthritis, Spinal) |  |
| #3 | #1 OR #2 |  |
| #4 | ((((((("Adipokines"[Mesh]) OR (Adipokine)) OR (Adipocytokine)) OR (Adipocytokines)) OR (Adiponectin)) OR (Leptin)) OR (Resistin)) OR (Zn-Alpha-2-Glycoprotein) |  |
| #5 | #3 AND #4 | **Q8:**  **102 resutls** |

**Database: Embase**

Search strategies:

| #1 | 'ankylosing spondylitis'/exp |  |
| --- | --- | --- |
| #2 | 'ankylating spondylitis' OR 'ankylopoietic spondylarthritis' OR 'ankylopoietic spondylitis' OR 'ankylosing spine' OR 'ankylosing spondilitis' OR 'ankylosing spondylarthritis' OR 'ankylosing spondylarthrosis' OR 'ankylosis spondylitis' OR 'ankylotic spondylitis' OR 'bechterew disease' OR 'bekhterev disease' OR 'morbus bechterew' OR 'spinal ankylosis' OR 'spine ankylosis' OR 'spondylarthritis ankylopoietica' OR 'spondylarthritis ankylosans' OR 'spondylarthrosis ankylopoietica' OR 'spondylitis ankylopoetica' OR 'spondylitis ankylopoietica' OR 'spondylitis, ankylosing' OR 'spondyloarthritis ankylopoietica' OR 'vertebral ankylosis' |  |
| #3 | #1 OR #2 |  |
| #4 | 'spondylarthritis'/exp |  |
| #5 | 'arthritis, spine' OR 'spine arthritis' OR 'spondyloarthritis' OR 'vertebral arthritis' OR 'vertebral osteo-arthritis' OR 'vertebral osteoarthritis' |  |
| #6 | #4 OR #5 |  |
| #7 | #3 OR #6 |  |
| #8 | 'adipocytokine'/exp |  |
| #9 | 'adipocytokines' OR 'adipokine' OR 'adipokines' OR 'adipose tissue derived cytokine' |  |
| #10 | 'adiponectin' OR 'leptin' OR 'zn-alpha-2-glycoprotein' OR 'resistin' |  |
| #11 | #8 OR #9 OR #10 |  |
| #12 | #7 AND #11 | **Q8:**  **335 results** |

**Database: Cochrane Library**

Search strategies:

| #1 | MeSH descriptor: [Spondylitis, Ankylosing] explode all trees |  |
| --- | --- | --- |
| #2 | (Spondyloarthritis Ankylopoietica or Spondyloarthritides, Ankylosing or Spondylitis Ankylopoietica or Ankylosing Spondyloarthritides or Ankylosing Spondylitis or Spondyloarthritis, Ankylosing or Spondylarthritis, Ankylosing or Marie-Struempell Disease or Bechterew Disease or Spondylarthritides, Ankylosing or Ankylosing Spondylarthritides or Ankylosing Spondyloarthritis or Bechterews Disease or Rheumatoid Spondylitis or Marie Struempell Disease or Bechterew's Disease or Ankylosing Spondylarthritis or Spondylarthritis Ankylopoietica or Spondylitis, Rheumatoid):ti,ab,kw |  |
| #3 | MeSH descriptor: [Spondylarthritis] explode all trees |  |
| #4 | (Spinal Arthritis or Spinal Arthritides or Spondylarthritides or Arthritis, Spinal):ti,ab,kw |  |
| #5 | #1 OR #2 |  |
| #6 | #3 OR #4 |  |
| #7 | #5 OR #6 |  |
| #8 | MeSH descriptor: [Adipokines] explode all trees |  |
| #9 | (Adipokine or Adipocytokine or Adipocytokines or Adiponectin or Leptin or Resistin or Zn Alpha 2 Glycoprotein):ti,ab,kw |  |
| #10 | #8 OR #9 |  |
| #11 | #7 AND #10 | **Q8:**  **10 results** |

**Question 9**: **VEGF**

**Database: PubMed**

Search strategies:

| #1 | "spondylarthritis"[MeSH Terms] OR "spondylarthritis"[All Fields] OR "spondyloarthritis"[All Fields] |  |
| --- | --- | --- |
| #2 | "spondylitis, ankylosing"[MeSH Terms] OR ("spondylitis"[All Fields] AND "ankylosing"[All Fields]) OR "ankylosing spondylitis"[All Fields] OR ("ankylosing"[All Fields] AND "spondylitis"[All Fields]) |  |
| #3 | #1 OR #2 |  |
| #4 | VEGF[MeSH Terms] |  |
| #5 | Vascular endothelial growth factor[MeSH Terms] |  |
| #6 | #4 OR #5 |  |
| #7 | #3 AND #6 | **Q9:**  **57 results** |

**Database: Embase**

Search strategies:

| #1 | 'spondylarthritis'/exp OR spondylarthritis |  |
| --- | --- | --- |
| #2 | 'ankylosing spondylitis'/exp OR 'ankylosing spondylitis' |  |
| #3 | #1 OR #2 |  |
| #4 | 'vascular endothelial growth factor'/exp OR 'vascular endothelial growth factor' OR (vascular AND endothelial AND ('growth'/exp OR growth) AND factor) |  |
| #5 | 'vegf'/exp OR vegf |  |
| #6 | #4 OR #5 |  |
| #7 | #3 AND #6 AND ([article]/lim OR [article in press]/lim) | **Q9:**  **163 results** |

**Database: Cochrane Library**

Search strategies:

| #1 | MeSH descriptor: [Spondylitis, Ankylosing] explode all trees |  |
| --- | --- | --- |
| #2 | (Spondyloarthritis Ankylopoietica or Spondyloarthritides, Ankylosing or Spondylitis Ankylopoietica or Ankylosing Spondyloarthritides or Ankylosing Spondylitis or Spondyloarthritis, Ankylosing or Spondylarthritis, Ankylosing or Marie-Struempell Disease or Bechterew Disease or Spondylarthritides, Ankylosing or Ankylosing Spondylarthritides or Ankylosing Spondyloarthritis or Bechterews Disease or Rheumatoid Spondylitis or Marie Struempell Disease or Bechterew's Disease or Ankylosing Spondylarthritis or Spondylarthritis Ankylopoietica or Spondylitis, Rheumatoid):ti,ab,kw |  |
| #3 | #1 OR #2 |  |
| #4 | MeSH descriptor: [Spondylarthritis] explode all trees |  |
| #5 | (Spinal Arthritis or Spinal Arthritides or Spondylarthritides or Arthritis, Spinal):ti,ab,kw |  |
| #6 | #4 OR #5 |  |
| #7 | #3 OR #6 |  |
| #8 | (Vascular endothelial growth factor):kw |  |
| #9 | (VEGF):kw |  |
| #10 | #8 OR #9 |  |
| #11 | #7 AND #10 in Trials | **Q9:**  **2,022 results** |

**Question 10**: **Calprotectin**

**Database: PubMed**

Search strategies:

| #1 | ((((((((((((((((((("Spondylitis, Ankylosing"[Mesh]) OR (Bechterew's Disease[All Fields])) OR (Bechterews Disease[All Fields])) OR (Marie-Struempell Disease[All Fields])) OR (Marie Struempell Disease[All Fields])) OR (Spondylarthritis Ankylopoietica[All Fields])) OR (Spondyloarthritis Ankylopoietica[All Fields])) OR (Ankylosing Spondylitis[All Fields])) OR (Ankylosing Spondylarthritis[All Fields])) OR (Ankylosing Spondylarthritides[All Fields])) OR (Spondylarthritides, Ankylosing[All Fields])) OR (Spondylarthritis, Ankylosing[All Fields])) OR (Ankylosing Spondyloarthritis[All Fields])) OR (Ankylosing Spondyloarthritides[All Fields])) OR (Spondyloarthritides, Ankylosing[All Fields])) OR (Spondyloarthritis, Ankylosing[All Fields])) OR (Spondylitis Ankylopoietica[All Fields])) OR (Bechterew Disease[All Fields])) OR (Rheumatoid Spondylitis[All Fields])) OR (Spondylitis, Rheumatoid[All Fields]) |  |
| --- | --- | --- |
| #2 | (((("Spondylarthritis"[Mesh]) OR (Spondylarthritides)) OR (Spinal Arthritis)) OR (Spinal Arthritides)) OR (Arthritis, Spinal) |  |
| #3 | #1 OR #2 |  |
| #4 | ("Leukocyte L1 Antigen Complex"[Mesh]) OR (Calcium-Binding Myeloid Protein P8,14) OR (Calcium Binding Myeloid Protein P8,14) OR (Calgranulin) OR (Calprotectin) OR (Migratory Inhibitory Factor-Related Protein MRP) OR (Migratory Inhibitory Factor Related Protein MRP) OR (Myelomonocytic Antigen L1) OR (Antigen L1, Myelomonocytic) OR (L1 Antigen) OR (Antigen, L1) OR (27E10 Antigen) OR (Antigen, 27E10) OR (Leukocyte L1 Protein) OR (L1 Protein, Leukocyte)) |  |
| #6 | #3 AND #4 | **Q10:**  **99 results** |

**Database: Embase**

Search strategies:

| #1 | 'spondylarthritis'/exp OR spondylarthritis |  |
| --- | --- | --- |
| #2 | 'ankylosing spondylitis'/exp OR 'ankylosing spondylitis' |  |
| #3 | #1 OR #2 |  |
| #4 | 'calprotectin'/exp OR calprotectin |  |
| #6 | #3 AND #4 | **Q10:**  **354 results** |

**Database: Cochrane Library**

Search strategies:

| #1 | ("spondylarthritis"):ti,ab,kw |  |
| --- | --- | --- |
| #2 | ("ankylosing spondylitis"):ti,ab,kw |  |
| #3 | #1 OR #2 |  |
| #5 | (calprotectin):ti,ab,kw |  |
| #6 | #3 AND #4 AND #5 | **Q10:**  **15 results** |

**Question 11****: non-coding RNA**

**Database: PubMed**

Search strategies:

| #1 | ((((((((((((((((((("Spondylitis, Ankylosing"[Mesh]) OR (Bechterew's Disease[All Fields])) OR (Bechterews Disease[All Fields])) OR (Marie-Struempell Disease[All Fields])) OR (Marie Struempell Disease[All Fields])) OR (Spondylarthritis Ankylopoietica[All Fields])) OR (Spondyloarthritis Ankylopoietica[All Fields])) OR (Ankylosing Spondylitis[All Fields])) OR (Ankylosing Spondylarthritis[All Fields])) OR (Ankylosing Spondylarthritides[All Fields])) OR (Spondylarthritides, Ankylosing[All Fields])) OR (Spondylarthritis, Ankylosing[All Fields])) OR (Ankylosing Spondyloarthritis[All Fields])) OR (Ankylosing Spondyloarthritides[All Fields])) OR (Spondyloarthritides, Ankylosing[All Fields])) OR (Spondyloarthritis, Ankylosing[All Fields])) OR (Spondylitis Ankylopoietica[All Fields])) OR (Bechterew Disease[All Fields])) OR (Rheumatoid Spondylitis[All Fields])) OR (Spondylitis, Rheumatoid[All Fields]) |  |
| --- | --- | --- |
| #2 | (((("Spondyloarthritis"[Mesh]) OR (Spondyloarthritides)) OR (Spinal Arthritis)) OR (Spinal Arthritides)) OR (Arthritis, Spinal) |  |
| #3 | #1 OR #2 |  |
| #4 | (transcriptomic) OR (non-coding RNA) OR (microRNA) OR (lncRNA) OR (circRNA) |  |
| #5 | #3 AND #4 | **Q11:**  **332 results** |

**Database: Embase**

Search strategies:

| #1 | 'spondyloarthritis'/exp OR spondyloarthritis |  |
| --- | --- | --- |
| #2 | 'ankylosing spondylitis'/exp OR 'ankylosing spondylitis' |  |
| #3 | #1 OR #2 |  |
| #4 | 'transcriptomic'/exp OR 'non-coding RNA' OR 'microRNA'/exp OR 'lncRNA' OR 'circRNA' |  |
| #5 | #3 AND #4 | **Q11:**  **94 results** |

**Database: Cochrane Library**

Search strategies:

| #1 | ("spondyloarthritis"):ti,ab,kw |  |
| --- | --- | --- |
| #2 | ("ankylosing spondylitis"):ti,ab,kw |  |
| #3 | #1 OR #2 |  |
| #4 | (transcriptome):ti,ab,kw OR (non-coding RNA):ti,ab,kw OR (microRNA):ti,ab,kw OR (lncRNA):ti,ab,kw OR (circRNA):ti,ab,kw |  |
| #5 | #3 AND #4 | **Q11:**  **1 result** |

**Question 12**: **Inflammatory cytokines including IL-6, IL-17 and TNF-α**

**Database: PubMed**

Search strategies:

| #1 | (cytokines[MeSH Terms]) OR (cytokine) |  |
| --- | --- | --- |
| #2 | (Interleukin-17[MeSH Terms]) |  |
| #3 | (Interleukin-6[MeSH Terms]) |  |
| #4 | (Tumor Necrosis Factor-alpha[MeSH Terms] |  |
| #5 | #2 OR #3 OR #4 |  |
| #6 | #1 AND #5 |  |
| #7 | "spondylitis, ankylosing"[MeSH Terms] OR ("spondylitis"[All Fields] AND "ankylosing"[All Fields]) OR "ankylosing spondylitis"[All Fields] OR ("ankylosing"[All Fields] AND "spondylitis"[All Fields]) |  |
| #8 | #6 AND #7 |  |
| #9 | #8 AND (("1974/1/1"[Date - Publication] : "2022/11/21"[Date - Publication])) |  |
| #10 | #9 AND (english[Language]) | **Q12:**  **1,899 results** |

**Database: Embase**

Search strategies:

| #1 | 'cytokine'/exp OR cytokine OR cytokines |  |
| --- | --- | --- |
| #2 | 'interleukin 6'/exp OR 'interleukin 6' |  |
| #3 | 'interleukin 17'/exp OR 'interleukin 17' |  |
| #4 | 'tumor necrosis factor-alpha'/exp OR 'tumor necrosis factor-alpha' OR (('tumor'/exp OR tumor) AND ('necrosis'/exp OR necrosis) AND 'factor alpha') |  |
| #5 | #2 OR #3 OR #4 |  |
| #6 | #1 AND #5 |  |
| #7 | 'ankylosing spondylitis'/exp OR 'ankylosing spondylitis' OR (ankylosing AND ('spondylitis'/exp OR spondylitis)) |  |
| #8 | #6 AND #7 |  |
| #9 | #6 AND #8 AND [01-01-1974]/sd NOT [22-11-2022]/sd |  |
| #10 | #6 AND #8 AND [01-01-1974]/sd NOT [22-11-2022]/sd AND [english]/lim |  |
| #11 | #6 AND #8 AND [01-01-1974]/sd NOT [22-11-2022]/sd AND [english]/lim AND [article]/lim | **Q12:**  **1,233 results** |

**Database: Cochrane Library**

Search strategies:

| #1 | MeSH descriptor: [Cytokines] explode all trees |  |
| --- | --- | --- |
| #2 | **MeSH descriptor: [Interleukin-17] explode all trees** |  |
| #3 | MeSH descriptor: [Interleukin-6] explode all trees |  |
| #4 | MeSH descriptor: [Tumor Necrosis Factor-alpha] explode all trees |  |
| #5 | #2 OR #3 OR #4 |  |
| #6 | #1 AND #5 |  |
| #7 | MeSH descriptor: [Spondylitis, Ankylosing] explode all trees |  |
| #8 | #6 AND #7 |  |
| #9 | #8 with Cochrane Library publication date from Jan 1974 to Nov 2022, in Trials | **Q12:**  **83 resutls** |

**Question 13**: **Peripheral lymphocyte subsets**

**Database: PubMed,Cochrane, Medline and China National Knowledge Infrastructure(CNKI)**

Search strategies:

| #1 | “ankylosing spondylitis” |  |
| --- | --- | --- |
| #2 | (“lymphocyte subsets” OR “T cell” OR “B cell” OR “Th1” OR “Th2” OR “Th17” OR ”Treg” OR “NK cell” OR “NKT cell” OR “gamma delta T cell”  OR “flow cytometry”) |  |
| #3 | #1 AND #2 | **Q13:**  **2,972 resutls** |

**Question 14**: **Bone turnover markers**

**Database: PubMed**

Search strategies:

| #1 | "spondylarthritis"[MeSH Terms] OR "spondylarthritis"[All Fields] OR "spondyloarthritis"[All Fields] |  |
| --- | --- | --- |
| #2 | "spondylitis, ankylosing"[MeSH Terms] OR ("spondylitis"[All Fields] AND "ankylosing"[All Fields]) OR "ankylosing spondylitis"[All Fields] OR ("ankylosing"[All Fields] AND "spondylitis"[All Fields]) |  |
| #3 | #1 OR #2 |  |
| #4 | (((bone metabolic markers) OR (bone metabolism markers)) OR (bone turnover marker)) OR (marker of bone metabolism) |  |
| #5 | #3 AND #4 |  |
| #6 | #5 AND (("1992/01/01"[Date - Publication] : "2022/11/21"[Date - Publication])) |  |
| #7 | #6 AND (English[Language]) | **Q14:**  **398 results** |

**Database: Embase**

Search strategies:

| #1 | 'spondylarthritis'/exp OR spondylarthritis |  |
| --- | --- | --- |
| #2 | 'ankylosing spondylitis'/exp OR 'ankylosing spondylitis' |  |
| #3 | #1 OR #2 |  |
| #4 | 'bone metabolic markers' OR (('bone'/exp OR bone) AND metabolic AND markers) |  |
| #5 | 'bone metabolism markers' OR (('bone'/exp OR bone) AND ('metabolism'/exp OR metabolism) AND markers) |  |
| #6 | 'bone turnover marker'/exp OR 'bone turnover marker' OR (('bone'/exp OR bone) AND ('turnover'/exp OR turnover) AND ('marker'/exp OR marker)) |  |
| #7 | 'marker of bone metabolism' OR (('marker'/exp OR marker) AND of AND ('bone'/exp OR bone) AND ('metabolism'/exp OR metabolism)) |  |
| #8 | #4 OR #5 OR #6 OR #7 |  |
| #9 | #3 AND #8 |  |
| #10 | #3 AND #8 AND [01-01-1992]/sd NOT [22-11-2022]/sd |  |
| #11 | #3 AND #8 AND [01-01-1992]/sd NOT [21-11-2022]/sd AND [english]/lim | **Q14:**  **1,095 results** |

**Database: Cochrane Library**

Search strategies:

| #1 | MeSH descriptor: [Spondylitis, Ankylosing] explode all trees |  |
| --- | --- | --- |
| #2 | (Spondyloarthritis Ankylopoietica or Spondyloarthritides, Ankylosing or Spondylitis Ankylopoietica or Ankylosing Spondyloarthritides or Ankylosing Spondylitis or Spondyloarthritis, Ankylosing or Spondylarthritis, Ankylosing or Marie-Struempell Disease or Bechterew Disease or Spondylarthritides, Ankylosing or Ankylosing Spondylarthritides or Ankylosing Spondyloarthritis or Bechterews Disease or Rheumatoid Spondylitis or Marie Struempell Disease or Bechterew's Disease or Ankylosing Spondylarthritis or Spondylarthritis Ankylopoietica or Spondylitis, Rheumatoid):ti,ab,kw |  |
| #3 | #1 OR #2 |  |
| #4 | MeSH descriptor: [Spondylarthritis] explode all trees |  |
| #5 | (Spinal Arthritis or Spinal Arthritides or Spondylarthritides or Arthritis, Spinal):ti,ab,kw |  |
| #6 | #4 OR #5 |  |
| #7 | #3 OR #6 |  |
| #8 | (bone metabolic markers):ti,ab,kw |  |
| #9 | (bone turnover marker):ti,ab,kw |  |
| #10 | (marker of bone metabolism):ti,ab,kw |  |
| #11 | (bone metabolism markers):ti,ab,kw |  |
| #12 | #8 OR #9 OR #10 OR #11 |  |
| #13 | #7 AND #12 |  |
| #14 | #13 with Cochrane Library publication date from Jan 1992 to Nov 2022 | **Q14:**  **16 results** |

**Database: CKNI**

Search strategies:

| #1 | 关键词=强直性脊柱炎 |  |
| --- | --- | --- |
| #2 | 关键词=骨密度 |  |
| #3 | 关键词=骨代谢 |  |
| #4 | #1 AND #2 OR #3 | **Q14:**  **186 results** |

**Question 15:** **C1M, C2M, C3M, C6M and VICM**

**Database: PubMed**

Search strategies:

| #1 | ((((((((((((((((((("Spondylitis, Ankylosing"[Mesh]) OR (Bechterew's Disease[All Fields])) OR (Bechterews Disease[All Fields])) OR (Marie-Struempell Disease[All Fields])) OR (Marie Struempell Disease[All Fields])) OR (Spondylarthritis Ankylopoietica[All Fields])) OR (Spondyloarthritis Ankylopoietica[All Fields])) OR (Ankylosing Spondylitis[All Fields])) OR (Ankylosing Spondylarthritis[All Fields])) OR (Ankylosing Spondylarthritides[All Fields])) OR (Spondylarthritides, Ankylosing[All Fields])) OR (Spondylarthritis, Ankylosing[All Fields])) OR (Ankylosing Spondyloarthritis[All Fields])) OR (Ankylosing Spondyloarthritides[All Fields])) OR (Spondyloarthritides, Ankylosing[All Fields])) OR (Spondyloarthritis, Ankylosing[All Fields])) OR (Spondylitis Ankylopoietica[All Fields])) OR (Bechterew Disease[All Fields])) OR (Rheumatoid Spondylitis[All Fields])) OR (Spondylitis, Rheumatoid[All Fields]) |  |
| --- | --- | --- |
| #2 | (((("Spondylarthritis"[Mesh]) OR (Spondylarthritides)) OR (Spinal Arthritis)) OR (Spinal Arthritides)) OR (Arthritis, Spinal) |  |
| #3 | #1 OR #2 |  |
| #4 | (Bone remodeling) OR (Bone turnover) OR (C1M) OR (C3M) OR (C6M) OR (VICM) |  |
| #5 | #3 AND #4 | **Q15:**  **20 results** |

**Database: Embase**

Search strategies:

| #1 | 'spondylarthritis'/exp OR spondylarthritis |  |
| --- | --- | --- |
| #2 | 'ankylosing spondylitis'/exp OR 'ankylosing spondylitis' |  |
| #3 | #1 OR #2 |  |
| #4 | 'Bone turnover' OR 'bone remodeling' OR 'VICM' OR 'C1M' OR 'C3M' OR 'C6M' |  |
| #5 | #3 AND #4 | **Q15:**  **58 results** |

**Database: Cochrane Library**

Search strategies:

| #1 | ("spondylarthritis"):ti,ab,kw |  |
| --- | --- | --- |
| #2 | ("ankylosing spondylitis"):ti,ab,kw |  |
| #3 | #1 OR #2 |  |
| #4 | (bone turnover):ti,ab,kw |  |
| #5 | #3 AND #4 | **Q15:**  **3 results** |

**Question 16**: **Sclerostin**

**Database: PubMed**

Search strategies:

| #1 | "spondylarthritis"[MeSH Terms] OR "spondylarthritis"[All Fields] OR "spondyloarthritis"[All Fields] |  |
| --- | --- | --- |
| #2 | "spondylitis, ankylosing"[MeSH Terms] OR ("spondylitis"[All Fields] AND "ankylosing"[All Fields]) OR "ankylosing spondylitis"[All Fields] OR ("ankylosing"[All Fields] AND "spondylitis"[All Fields]) |  |
| #3 | #1 OR #2 |  |
| #4 | (sclerostin[Title/Abstract]) |  |
| #5 | #3 AND #4 Filters: English | **Q16:**  **72 results** |

**Database: Embase**

Search strategies:

| #1 | 'spondylarthritis'/exp OR spondylarthritis |  |
| --- | --- | --- |
| #2 | 'ankylosing spondylitis'/exp OR 'ankylosing spondylitis' |  |
| #3 | #1 OR #2 |  |
| #4 | sclerostin:ab,ti |  |
| #5 | #3 AND #7 AND ([article]/lim OR [article in press]/lim) AND [english]/lim | **Q16:**  **60 results** |

**Database: Cochrane Library**

Search strategies:

| #1 | MeSH descriptor: [Spondylitis, Ankylosing] explode all trees |  |
| --- | --- | --- |
| #2 | (Spondyloarthritis Ankylopoietica or Spondyloarthritides, Ankylosing or Spondylitis Ankylopoietica or Ankylosing Spondyloarthritides or Ankylosing Spondylitis or Spondyloarthritis, Ankylosing or Spondylarthritis, Ankylosing or Marie-Struempell Disease or Bechterew Disease or Spondylarthritides, Ankylosing or Ankylosing Spondylarthritides or Ankylosing Spondyloarthritis or Bechterews Disease or Rheumatoid Spondylitis or Marie Struempell Disease or Bechterew's Disease or Ankylosing Spondylarthritis or Spondylarthritis Ankylopoietica or Spondylitis, Rheumatoid):ti,ab,kw |  |
| #3 | #1 OR #2 |  |
| #4 | (sclerostin):kw |  |
| #5 | #4 AND #5 | **Q16:**  **84 results** |

**Question 17**: **DKK-1**

**Database: PubMed**

Search strategies:

| #1 | ((((((((((((((((((("Spondylitis, Ankylosing"[Mesh]) OR (Bechterew's Disease[All Fields])) OR (Bechterews Disease[All Fields])) OR (Marie-Struempell Disease[All Fields])) OR (Marie Struempell Disease[All Fields])) OR (Spondylarthritis Ankylopoietica[All Fields])) OR (Spondyloarthritis Ankylopoietica[All Fields])) OR (Ankylosing Spondylitis[All Fields])) OR (Ankylosing Spondylarthritis[All Fields])) OR (Ankylosing Spondylarthritides[All Fields])) OR (Spondylarthritides, Ankylosing[All Fields])) OR (Spondylarthritis, Ankylosing[All Fields])) OR (Ankylosing Spondyloarthritis[All Fields])) OR (Ankylosing Spondyloarthritides[All Fields])) OR (Spondyloarthritides, Ankylosing[All Fields])) OR (Spondyloarthritis, Ankylosing[All Fields])) OR (Spondylitis Ankylopoietica[All Fields])) OR (Bechterew Disease[All Fields])) OR (Rheumatoid Spondylitis[All Fields])) OR (Spondylitis, Rheumatoid[All Fields]) |  |
| --- | --- | --- |
| #2 | (((("Spondylarthritis"[Mesh]) OR (Spondylarthritides)) OR (Spinal Arthritis)) OR (Spinal Arthritides)) OR (Arthritis, Spinal) |  |
| #3 | #1 OR #2 |  |
| #4 | (DDK1) OR (Dickkopf1) OR (DDK-1) OR (Dickkopf-1) |  |
| #5 | #3 AND #4 | **Q17:**  **65 results** |

**Database: Embase**

Search strategies:

| #1 | 'spondylarthritis'/exp OR spondylarthritis |  |
| --- | --- | --- |
| #2 | 'ankylosing spondylitis'/exp OR 'ankylosing spondylitis' |  |
| #3 | #1 OR #2 |  |
| #4 | 'dickkopf 1 protein'/exp OR 'dickkopf 1' OR 'ddk1' |  |
| #5 | #3 AND #4 | **Q17:**  **281 results** |

**Database: Cochrane Library**

Search strategies:

| #1 | ("spondylarthritis"):ti,ab,kw |  |
| --- | --- | --- |
| #2 | ("ankylosing spondylitis"):ti,ab,kw |  |
| #3 | #1 OR #2 |  |
| #4 | (Dickkopf-1):ti,ab,kw |  |
| #5 | #3 AND #4 | **Q17:**  **3 results** |

**Question 18: OPG/RANKL/RANK**

**Database: PubMed**

Search strategies:

| #1 | ((((((((((((((((((("Spondylitis, Ankylosing"[Mesh]) OR (Bechterew's Disease[All Fields])) OR (Bechterews Disease[All Fields])) OR (Marie-Struempell Disease[All Fields])) OR (Marie Struempell Disease[All Fields])) OR (Spondylarthritis Ankylopoietica[All Fields])) OR (Spondyloarthritis Ankylopoietica[All Fields])) OR (Ankylosing Spondylitis[All Fields])) OR (Ankylosing Spondylarthritis[All Fields])) OR (Ankylosing Spondylarthritides[All Fields])) OR (Spondylarthritides, Ankylosing[All Fields])) OR (Spondylarthritis, Ankylosing[All Fields])) OR (Ankylosing Spondyloarthritis[All Fields])) OR (Ankylosing Spondyloarthritides[All Fields])) OR (Spondyloarthritides, Ankylosing[All Fields])) OR (Spondyloarthritis, Ankylosing[All Fields])) OR (Spondylitis Ankylopoietica[All Fields])) OR (Bechterew Disease[All Fields])) OR (Rheumatoid Spondylitis[All Fields])) OR (Spondylitis, Rheumatoid[All Fields]) |  |
| --- | --- | --- |
| #2 | (((("Spondylarthritis"[Mesh]) OR (Spondylarthritides)) OR (Spinal Arthritis)) OR (Spinal Arthritides)) OR (Arthritis, Spinal) |  |
| #3 | #1 OR #2 |  |
| #4 | (RANKL) OR (RANK) OR (receptor activator of nuclear factor-κB ligand) OR (receptor activator of nuclear factor-κB) OR (OPG) OR (osteoprotegerin) |  |
| #5 | #3 AND #4 | **Q18:**  **309 results** |

**Database: Embase**

Search strategies:

| #1 | 'spondylarthritis'/exp OR spondylarthritis |  |
| --- | --- | --- |
| #2 | 'ankylosing spondylitis'/exp OR 'ankylosing spondylitis' |  |
| #3 | #1 OR #2 |  |
| #4 | 'rankl'/exp OR 'rankl' OR 'rank' OR 'receptor activator of nuclear factor-κb ligand' OR 'receptor activator of nuclear factor-κb' OR 'opg' OR 'osteoprotegerin'/exp OR 'osteoprotegerin' |  |
| #5 | #3 AND #4 | **Q18:**  **526 results** |

**Database: Cochrane Library**

Search strategies:

| #1 | ("spondylarthritis"):ti,ab,kw |  |
| --- | --- | --- |
| #2 | ("ankylosing spondylitis"):ti,ab,kw |  |
| #3 | #1 OR #2 |  |
| #4 | (RANKL):ti,ab,kw OR (RANK):ti,ab,kw OR (OPG):ti,ab,kw |  |
| #5 | #3 AND #4 | **Q18:**  **2 results** |

**Question 19**: **MMP3**

**Database: PubMed**

Search strategies:

| #1 | "spondylarthritis"[MeSH Terms] OR "spondylarthritis"[All Fields] OR "spondyloarthritis"[All Fields] |  |
| --- | --- | --- |
| #2 | "spondylitis, ankylosing"[MeSH Terms] OR ("spondylitis"[All Fields] AND "ankylosing"[All Fields]) OR "ankylosing spondylitis"[All Fields] OR ("ankylosing"[All Fields] AND "spondylitis"[All Fields]) |  |
| #3 | #1 OR #2 |  |
| #4 | "mmp3 protein human"[Supplementary Concept] OR "mmp3 protein human"[All Fields] OR "matrix metalloproteinase 3"[All Fields] OR "matrix metalloproteinase 3"[Supplementary Concept] OR "matrix metalloproteinase 3"[MeSH Terms] |  |
| #5 | #3 AND #4 | **Q19:**  **92 results** |

**Database: Embase**

Search strategies:

| #1 | 'spondylarthritis'/exp OR spondylarthritis |  |
| --- | --- | --- |
| #2 | 'ankylosing spondylitis'/exp OR 'ankylosing spondylitis' |  |
| #3 | #1 OR #2 |  |
| #4 | ' matrix metalloproteinase 3' |  |
| #5 | #3 AND #4 | **Q19:**  **116 results** |

**Database: Cochrane Library**

Search strategies:

| #1 | ("spondyloarthritis"):ti,ab,kw |  |
| --- | --- | --- |
| #2 | ("ankylosing spondylitis"):ti,ab,kw |  |
| #3 | #1 OR #2 |  |
| #4 | (matrix metalloproteinase 3):ti,ab,kw |  |
| #9 | #3 AND #4 | **Q19:**  **6 results** |

**Question 20**: **BMP2**

**Database: PubMed**

Search strategies:

| #1 | "spondylarthritis"[MeSH Terms] OR "spondylarthritis"[All Fields] OR "spondyloarthritis"[All Fields] |  |
| --- | --- | --- |
| #2 | "spondylitis, ankylosing"[MeSH Terms] OR ("spondylitis"[All Fields] AND "ankylosing"[All Fields]) OR "ankylosing spondylitis"[All Fields] OR ("ankylosing"[All Fields] AND "spondylitis"[All Fields]) |  |
| #3 | #1 OR #2 |  |
| #4 | "bone morphogenetic protein 2"[Supplementary Concept] OR "bone morphogenetic protein 2"[All Fields] OR "bmp2 protein human"[Supplementary Concept] OR "bmp2 protein human"[All Fields] OR "bone morphogenetic protein 2"[MeSH Terms] |  |
| #5 | #3 AND #4 | **Q20:**  **27 results** |

**Database: Embase**

Search strategies:

| #1 | 'spondylarthritis'/exp OR spondylarthritis |  |
| --- | --- | --- |
| #2 | 'ankylosing spondylitis'/exp OR 'ankylosing spondylitis' |  |
| #3 | #1 OR #2 |  |
| #4 | 'bone morphogenetic protein 2' |  |
| #5 | #3 AND #4 | **Q20:**  **126 results** |

**Database: Cochrane Library**

Search strategies:

| #1 | ("spondyloarthritis"):ti,ab,kw |  |
| --- | --- | --- |
| #2 | ("ankylosing spondylitis"):ti,ab,kw |  |
| #3 | #1 OR #2 |  |
| #4 | (bone morphogenetic protein 2):ti,ab,kw |  |
| #9 | #3 AND #4 | **Q20：**  **5 results** |

**Question 21: TNC**

**Database: PubMed**

Search strategies:

| #1 | ((((((((((((((((((("Spondylitis, Ankylosing"[Mesh]) OR (Bechterew's Disease[All Fields])) OR (Bechterews Disease[All Fields])) OR (Marie-Struempell Disease[All Fields])) OR (Marie Struempell Disease[All Fields])) OR (Spondylarthritis Ankylopoietica[All Fields])) OR (Spondyloarthritis Ankylopoietica[All Fields])) OR (Ankylosing Spondylitis[All Fields])) OR (Ankylosing Spondylarthritis[All Fields])) OR (Ankylosing Spondylarthritides[All Fields])) OR (Spondylarthritides, Ankylosing[All Fields])) OR (Spondylarthritis, Ankylosing[All Fields])) OR (Ankylosing Spondyloarthritis[All Fields])) OR (Ankylosing Spondyloarthritides[All Fields])) OR (Spondyloarthritides, Ankylosing[All Fields])) OR (Spondyloarthritis, Ankylosing[All Fields])) OR (Spondylitis Ankylopoietica[All Fields])) OR (Bechterew Disease[All Fields])) OR (Rheumatoid Spondylitis[All Fields])) OR (Spondylitis, Rheumatoid[All Fields]) |  |
| --- | --- | --- |
| #2 | (((("Spondylarthritis"[Mesh]) OR (Spondylarthritides)) OR (Spinal Arthritis)) OR (Spinal Arthritides)) OR (Arthritis, Spinal) |  |
| #3 | #1 OR #2 |  |
| #4 | ("Tenascin"[Mesh]) OR (((((Hexabrachion) OR (Tenascin-C)) OR (Tenascin C)) OR (Cytotactin)) OR (J1-200-220)) |  |
| #5 | #3 AND #4 | **Q21:**  **8 results** |

**Database: Embase**

Search strategies:

| #1 | 'ankylosing spondylitis'/exp |  |
| --- | --- | --- |
| #2 | 'ankylating spondylitis' OR 'ankylopoietic spondylarthritis' OR 'ankylopoietic spondylitis' OR 'ankylosing spine' OR 'ankylosing spondilitis' OR 'ankylosing spondylarthritis' OR 'ankylosing spondylarthrosis' OR 'ankylosis spondylitis' OR 'ankylotic spondylitis' OR 'bechterew disease' OR 'bekhterev disease' OR 'morbus bechterew' OR 'spinal ankylosis' OR 'spine ankylosis' OR 'spondylarthritis ankylopoietica' OR 'spondylarthritis ankylosans' OR 'spondylarthrosis ankylopoietica' OR 'spondylitis ankylopoetica' OR 'spondylitis ankylopoietica' OR 'spondylitis, ankylosing' OR 'spondyloarthritis ankylopoietica' OR 'vertebral ankylosis' |  |
| #3 | #1 OR #2 |  |
| #4 | 'spondylarthritis'/exp |  |
| #5 | 'arthritis, spine' OR 'spine arthritis' OR 'spondyloarthritis' OR 'vertebral arthritis' OR 'vertebral osteo-arthritis' OR 'vertebral osteoarthritis' |  |
| #6 | #4 OR #5 |  |
| #7 | #3 OR #6 |  |
| #8 | 'tenascin'/exp |  |
| #9 | 'cytotactin' OR 'glioma mesenchymal extracellular matrix antigen' OR 'glycoprotein j1' OR 'hexabrachion' OR 'j1 glycoprotein' OR 'myotendinous antigen' OR 'tenascin c' OR 'tenascin' |  |
| #10 | #8 OR #9 |  |
| #12 | #7 AND #10 | **Q21:**  **16 results** |

**Database: Cochrane Library**

Search strategies:

| #1 | MeSH descriptor: [Spondylitis, Ankylosing] explode all trees |  |
| --- | --- | --- |
| #2 | (Spondyloarthritis Ankylopoietica or Spondyloarthritides, Ankylosing or Spondylitis Ankylopoietica or Ankylosing Spondyloarthritides or Ankylosing Spondylitis or Spondyloarthritis, Ankylosing or Spondylarthritis, Ankylosing or Marie-Struempell Disease or Bechterew Disease or Spondylarthritides, Ankylosing or Ankylosing Spondylarthritides or Ankylosing Spondyloarthritis or Bechterews Disease or Rheumatoid Spondylitis or Marie Struempell Disease or Bechterew's Disease or Ankylosing Spondylarthritis or Spondylarthritis Ankylopoietica or Spondylitis, Rheumatoid):ti,ab,kw |  |
| #3 | MeSH descriptor: [Spondylarthritis] explode all trees |  |
| #4 | (Spinal Arthritis or Spinal Arthritides or Spondylarthritides or Arthritis, Spinal):ti,ab,kw |  |
| #5 | #1 OR #2 |  |
| #6 | #3 OR #4 |  |
| #7 | #5 OR #6 |  |
| #8 | MeSH descriptor: [Tenascin] explode all trees |  |
| #9 | (Hexabrachion or Cytotactin or Tenascin C ):ti,ab,kw |  |
| #10 | #8 OR #9 |  |
| #11 | #7 AND #10 | **Q21:**  **0 result** |

**Question 22: Gut microbiota**

**Database: PubMed**

Search strategies:

| #1 | ((((((((((((((((((("Spondylitis, Ankylosing"[Mesh]) OR (Bechterew's Disease[All Fields])) OR (Bechterews Disease[All Fields])) OR (Marie-Struempell Disease[All Fields])) OR (Marie Struempell Disease[All Fields])) OR (Spondylarthritis Ankylopoietica[All Fields])) OR (Spondyloarthritis Ankylopoietica[All Fields])) OR (Ankylosing Spondylitis[All Fields])) OR (Ankylosing Spondylarthritis[All Fields])) OR (Ankylosing Spondylarthritides[All Fields])) OR (Spondylarthritides, Ankylosing[All Fields])) OR (Spondylarthritis, Ankylosing[All Fields])) OR (Ankylosing Spondyloarthritis[All Fields])) OR (Ankylosing Spondyloarthritides[All Fields])) OR (Spondyloarthritides, Ankylosing[All Fields])) OR (Spondyloarthritis, Ankylosing[All Fields])) OR (Spondylitis Ankylopoietica[All Fields])) OR (Bechterew Disease[All Fields])) OR (Rheumatoid Spondylitis[All Fields])) OR (Spondylitis, Rheumatoid[All Fields]) |  |
| --- | --- | --- |
| #2 | (((("Spondyloarthritis"[Mesh]) OR (Spondyloarthritides)) OR (Spinal Arthritis)) OR (Spinal Arthritides)) OR (Arthritis, Spinal) |  |
| #3 | #1 OR #2 |  |
| #4 | (gut microbiome) OR (gut microbiota) OR (metagenomic sequencing) OR (16S rRNA) |  |
| #5 | #3 AND #4 | **Q22:**  **218 results** |

**Database: Embase**

Search strategies:

| #1 | 'spondyloarthritis'/exp OR spondyloarthritis |  |
| --- | --- | --- |
| #2 | 'ankylosing spondylitis'/exp OR 'ankylosing spondylitis' |  |
| #3 | #1 OR #2 |  |
| #4 | 'gut microbiome'/exp OR 'gut microbiota' OR 'metagenomic sequencing'/exp OR '16S rRNA' |  |
| #5 | #3 AND #4 | **Q22:**  **97 results** |

**Database: Cochrane Library**

Search strategies:

| #1 | ("spondyloarthritis"):ti,ab,kw |  |
| --- | --- | --- |
| #2 | ("ankylosing spondylitis"):ti,ab,kw |  |
| #3 | #1 OR #2 |  |
| #4 | (gut microbiota):ti,ab,kw OR (metagenomic sequencing):ti,ab,kw OR (16S rRNA):ti,ab,kw |  |
| #5 | #3 AND #4 | **Q22:**  **3 results** |

**Question 23: Metabonomics**

**Database: PubMed**

Search strategies:

| #1 | ((((((((((((((((((("Spondylitis, Ankylosing"[Mesh]) OR (Bechterew's Disease[All Fields])) OR (Bechterews Disease[All Fields])) OR (Marie-Struempell Disease[All Fields])) OR (Marie Struempell Disease[All Fields])) OR (Spondylarthritis Ankylopoietica[All Fields])) OR (Spondyloarthritis Ankylopoietica[All Fields])) OR (Ankylosing Spondylitis[All Fields])) OR (Ankylosing Spondylarthritis[All Fields])) OR (Ankylosing Spondylarthritides[All Fields])) OR (Spondylarthritides, Ankylosing[All Fields])) OR (Spondylarthritis, Ankylosing[All Fields])) OR (Ankylosing Spondyloarthritis[All Fields])) OR (Ankylosing Spondyloarthritides[All Fields])) OR (Spondyloarthritides, Ankylosing[All Fields])) OR (Spondyloarthritis, Ankylosing[All Fields])) OR (Spondylitis Ankylopoietica[All Fields])) OR (Bechterew Disease[All Fields])) OR (Rheumatoid Spondylitis[All Fields])) OR (Spondylitis, Rheumatoid[All Fields]) |  |
| --- | --- | --- |
| #2 | (((("Spondyloarthritis"[Mesh]) OR (Spondyloarthritides)) OR (Spinal Arthritis)) OR (Spinal Arthritides)) OR (Arthritis, Spinal) |  |
| #3 | #1 OR #2 |  |
| #4 | (metabolomics) OR (metabolites) |  |
| #5 | #3 AND #4 | **Q23:**  **114 results** |

**Database: Embase**

Search strategies:

| #1 | 'spondyloarthritis'/exp OR spondyloarthritis |  |
| --- | --- | --- |
| #2 | 'ankylosing spondylitis'/exp OR 'ankylosing spondylitis' |  |
| #3 | #1 OR #2 |  |
| #4 | 'metabolomics'/exp OR 'metabolomics' OR 'metabolites'/exp OR 'metabolites' |  |
| #5 | #3 AND #4 | **Q23:**  **61 results** |

**Database: Cochrane Library**

Search strategies:

| #1 | ("spondyloarthritis"):ti,ab,kw |  |
| --- | --- | --- |
| #2 | ("ankylosing spondylitis"):ti,ab,kw |  |
| #3 | #1 OR #2 |  |
| #4 | (metabolimics):ti,ab,kw OR (metabolites):ti,ab,kw |  |
| #5 | #3 AND #4 | **Q23:**  **1 result** |

**Question 24: NSAIDs-related genes**

**Database: PubMed**

Search strategies:

| #1 | "anti inflammatory agents non steroidal"[Pharmacological Action] OR "anti inflammatory agents, non steroidal"[MeSH Terms] OR "non-steroidal anti-inflammatory agents"[All Fields] OR "nsaid"[All Fields] OR "nsaids"[All Fields] |  |
| --- | --- | --- |
| #2 | "cytochrome p 450 cyp2c9"[MeSH Terms] OR "cytochrome p 450 cyp2c9"[All Fields] OR "cyp2c9"[All Fields] OR "p450 2c9"[All Fields] |  |
| #3 | "polymorphism, genetic"[MeSH Terms] OR "genetic polymorphism" [All Fields] OR "gene polymorphism"[All Fields] |  |
| #4 | #2 OR #3 |  |
| #5 | #1 AND #4 | **Q24:**  **1,464 results** |

**Database: Embase**

Search strategies:

| #1 | 'nonsteroid antiinflammatory agent'/exp OR 'nonsteroid antiinflammatory agent' |  |
| --- | --- | --- |
| #2 | 'cytochrome p450 2c9' |  |
| #3 | 'genetic polymorphism' |  |
| #4 | #2 OR #3 |  |
| #5 | #1 AND #4 | **Q24:**  **7,215 results** |

**Database: Cochrane Library**

Search strategies:

| #1 | (NSAID):ti,ab,kw |  |
| --- | --- | --- |
| #2 | (non-steroidal anti-inflammatory drugs):ti,ab,kw |  |
| #3 | #1 OR #2 |  |
| #4 | (CYP2C9):ti,ab,kw |  |
| #5 | (P450 2C9):ti,ab,kw |  |
| #6 | (cytochrome p450 2c9):ti,ab,kw |  |
| #7 | (genetic polymorphism):ti,ab,kw |  |
| #8 | #4 OR #5 OR #6 OR #7 |  |
| #9 | #3 AND #8 | **Q24:**  **25 results** |

**Question 25: SSZ-related genes**

**Database: PubMed**

Search strategies:

| #1 | (("sulfasalazine"[MeSH Terms] OR "sulfasalazine"[All Fields] OR "sulfasalazin"[All Fields] OR "sulphasalazine"[All Fields]) AND ("polymorphic"[All Fields] OR "polymorphics"[All Fields] OR "polymorphism s"[All Fields] OR "polymorphism, genetic"[MeSH Terms] OR ("polymorphism"[All Fields] AND "genetic"[All Fields]) OR "genetic polymorphism"[All Fields] OR "polymorphism"[All Fields] OR "polymorphisms"[All Fields])) OR (("sulfasalazine"[MeSH Terms] OR "sulfasalazine"[All Fields] OR "sulfasalazin"[All Fields] OR "sulphasalazine"[All Fields]) AND ("pharmacogenetically"[All Fields] OR "pharmacogenetics"[MeSH Terms] OR "pharmacogenetics"[All Fields] OR "pharmacogenetic"[All Fields])) OR (("sulfasalazine"[MeSH Terms] OR "sulfasalazine"[All Fields] OR "sulfasalazin"[All Fields] OR "sulphasalazine"[All Fields]) AND "N-Acetyltransferase"[All Fields]) | **Q25:**  **94 results** |
| --- | --- | --- |

**Database: Embase**

Search strategies:

| #1 | 'salazosulfapyridine'/exp |  |
| --- | --- | --- |
| #2 | 'n acetyltransferase'/exp OR 'n acetyltransferase' OR nat2 |  |
| #3 | 'polymorphism'/exp |  |
| #4 | 'pharmacogenetics'/exp |  |
| #5 | #1 AND #2 |  |
| #6 | #1 AND #3 |  |
| #7 | #1 AND #4 |  |
| #8 | #5 OR #6 OR #7 | **Q25:**  **221 results** |

**Database: Cochrane Library**

Search strategies:

| #1 | (sulfasalazine):ti,ab,kw AND (polymorphism):ti,ab,kw |  |
| --- | --- | --- |
| #2 | (sulfasalazine):ti,ab,kw AND (pharmacogenetically):ti,ab,kw |  |
| #3 | (sulfasalazine):ti,ab,kw AND (N-Acetyltransferase):ti,ab,kw |  |
| #4 | #2 OR #4 OR #5 | **Q25:**  **7 results** |

**Question 26: Anti-drug antibodies**

**Database: PubMed**

Search strategies:

| #1 | "Spondylitis, Ankylosing "[Mesh] OR ankylosing[Title/Abstract] OR spondyloarthritis[Title/Abstract] |  |
| --- | --- | --- |
| #2 | (etanercept[Title/Abstract] OR enbrel[Title/Abstract] OR benepali[Title/Abstract] OR erelzi[Title/Abstract] OR etanercept[MeSH Terms] OR adalimumab[Title/Abstract] OR humira[Title/Abstract] OR adalimumab[MeSH Terms] OR infliximab[Title/Abstract] OR remicade[Title/Abstract] OR inflectra[Title/Abstract] OR remsima[Title/Abstract] OR flixabi[Title/Abstract] OR infliximab[MeSH Terms] OR golimumab[Title/Abstract] OR simponi[Title/Abstract] OR certolizumab[Title/Abstract] OR certolizumab pegol[Title/Abstract] OR cimzia[Title/Abstract] OR certolizumab pegol[MeSH Terms] OR biologic*[Title/Abstract] OR bDMARD[Title/Abstract] OR anti-TNF[Title/Abstract] OR TNFi[Title/Abstract] OR TNF inhibitor*[Title/Abstract] OR biosimilar*[Title/Abstract] OR biosimilar pharmaceuticals[MeSH Terms] OR antibodies, monoclonal[MeSH Terms]) |  |
| #3 | #1 AND #2 |  |
| #4 | immunogenicity [all fields] OR antibody formation [all fields] OR Antibody Formation [MeSH terms] OR “response failure”[all fields] OR bioavailability OR biological availability OR “Biological Availability”[MeSH Terms] OR “drug tolerance”[all fields] OR Drug Tolerance [MeSH Terms] OR“treatment outcome” [all fields] OR Treatment Outcome [MeSH Terms] OR anti-drug antibodies OR anti-drug antibody[Title/Abstract] OR ADAs[Title/Abstract] OR antibodies[Title/Abstract] |  |
| #5 | #3 AND #4 | **Q26:**  **1822 results** |

**Database: Embase**

Search strategies:

| #1 | 'ankylosing spondylitis'/exp OR spondyloarthritis:ab,ti OR ankylosing:ab,ti |  |
| --- | --- | --- |
| #2 | (etanercept OR enbrel:ab,ti OR benepali:ab,ti OR erelzi:ab,ti OR etanercept/exp OR adalimumab:ab,ti OR humira:ab,ti OR adalimumab/exp OR infliximab:ab,ti OR remicade:ab,ti OR inflectra:ab,ti OR remsima:ab,ti OR flixabi:ab,ti OR infliximab/exp OR golimumab:ab,ti OR simponi:ab,ti OR bDMARD:ab,ti OR anti-TNF:ab,ti OR TNFi:ab,ti OR TNF inhibitor*:ab,ti OR biosimilar*:ab,ti OR biosimilar pharmaceuticals OR antibodies, monoclonal/exp) |  |
| #3 | #1 AND #2 |  |
| #4 | (immunogenic* OR antibody formation:ab,ti OR Antibody Formation/exp OR response failure:ab,ti OR bioavailability OR biological availability OR Biological Availability/exp OR drug tolerance:ab,ti OR Drug Tolerance/exp OR treatment outcome:ab,ti OR Treatment Outcome/exp OR anti-drug antibodies OR anti-drug antibody:ab,ti OR ADAs:ab,ti) |  |
| #5 | #3 AND #4 | **Q26:**  **18 results** |

**Database: Cochrane Library**

Search strategies:

| #1 | MeSH descriptor: [Spondylitis, Ankylosing] explode all trees |  |
| --- | --- | --- |
| #2 | (spondyloarthritis):ti,ab,kw |  |
| #3 | #1 OR #2 |  |
| #4 | (anti-drug antibodies):ti,ab,kw |  |
| #5 | #3 AND #4 | **Q26:**  **35 results** |
