# Supplementary material for: A guideline on biomarkers in the diagnosis and evaluation in axial spondyloarthritis
Source: Front Immunol. 2024 Oct 30;15:1394148. doi: 10.3389/fimmu.2024.1394148 (PMC11557325; doi:10.3389/fimmu.2024.1394148)
Supplement: Supplementary file 4 [file Table4.docx]

**SUPPLEMENTARY APPENDIX 4: Flowchart of the study selection process**

**Question 1: HLA-B27**

Records identified through searching of PubMed (n=179), Embase (n=377) and Cochrane Library (n = 13)

Total records screened after duplicates publications removed
(n = 366)

Total excluded after title and abstract screening (n = 270)

*Citations did not match study designs of interest, did not examine populations or interventions of interest, or did not report outcome measures of interest* (n = 46)

Review or Meta-analysis or Case report or Conferrence abstract or letter or Animal model (n = 180)

No full-text((n = 44)

Full-text articles assessed for eligibility
(n = 96)

Full-text articles excluded (n = 54)

*Citations did not provide evidence matching questions of interest*

Studies considered
for evidence report
(n = 42)

**Question 2: HLA-B27 subtypes**

Records identified through searching of PubMed (n=730), Embase (n=301) and Cochrane Library (n = 3)

Total records screened after duplicates and
non-English publications removed
(n = 864)

Total excluded after title and abstract screening (n = 766)

*Citations did not match study designs of interest, did not examine populations or interventions of interest, or did not report outcome measures of interest*

Full-text articles assessed for eligibility
(n = 98)

Full-text articles excluded (n = 56)

*Citations did not provide evidence matching a question of interest*

Studies considered
for evidence report
(n = 42)

Studies excluded during data extraction
(n = 2)

Studies matched

to this part
(n = 40)

**Question 3: Genes**

Records identified through searching of PubMed (n=5371), Embase (n=1899), Cochrane Library (n =165) and CNKI (n=248)

Total records screened after duplicates and
non-English publications removed
(n = 6309)

Total excluded after title and abstract screening (n = 5679)

*Citations did not match study designs of interest, did not examine populations or interventions of interest, or did not report outcome measures of interest*

Full-text articles assessed for eligibility
(n = 630)

Full-text articles excluded (n = 375)

*Citations did not provide evidence matching a question of interest*

Studies considered
for evidence report
(n = 255)

Studies excluded during data extraction
(n = 221)

Studies matched

to this part
(n = 34)

**Question 4**: **Antibodies**

Records identified through searching of PubMed (n=381), Embase (n=765)

Total records screened after duplicates publications removed
(n = 1019)

Total excluded after title and abstract screening (n = 103)

*Citations did not match study designs of interest, did not examine populations or interventions of interest, or did not report outcome measures of interest* (n = 185)

Review or Meta-analysis or Case report or Conferrence abstract or letter or Animal model (n = 388)

No full-text(n = 252)

Full-text articles assessed for eligibility
(n = 91)

Full-text articles excluded (n = 55)

*Citations did not provide evidence matching questions of interest*

Studies considered
for evidence report
(n = 36)

**Question 5**: **CRP**

Records identified through searching of PubMed (n=2342), Embase (n=6994) and Cochrane Library (n =783)

Total records screened after duplicates（n=3307） and non-English publications （n=0） removed （n=6812）
(n = 6812)

Total excluded after title and abstract screening (n =4330)

*Citations did not match study designs of interest, did not examine populations or interventions of interest, or did not report outcome measures of interest*

Full-text articles assessed for eligibility
(n = 2482)

Full-text articles excluded (n = 2166)

*Citations did not provide evidence matching a question of interest*

Studies considered
for evidence report
(n = 316)

Studies excluded during data extraction
(n = 258)

Studies matched

to this part
(n =58)

**Question 6: ESR**

Records identified through searching of PubMed (n=908), Embase (n=3146) and Cochrane Library (n = 259)

Total records screened after duplicates and
non-English publications removed
(n = 3390)

Total excluded after title and abstract screening (n = 3132)

*Citations did not match study designs of interest, did not examine populations or interventions of interest, or did not report outcome measures of interest*

Full-text articles assessed for eligibility
(n = 258)

Full-text articles excluded (n = 224)

*Citations did not provide evidence matching questions of interest*

Studies considered
for evidence report
(n = 34)

Studies excluded during data extraction
(n = 3)

Studies matched

to this part
(n = 31)

**Question 7: SAA**

Records identified through searching of PubMed (n=44), Embase (n=144), Cochrane Library (n = 4) and CKNI(n=7)

Total records screened after duplicates and
non-English or non-Chinese publications removed
(n = 174)

Total excluded after title and abstract screening (n = 139)

*Citations did not match study designs of interest, did not examine populations or interventions of interest, or did not report outcome measures of interest* (n = 68)

Type of work,including review or meta-analysis or case report or conferrence abstract or letter or animal model or editorial or notes (n = 71)

Full-text articles assessed for eligibility
(n = 35)

Full-text articles excluded (n =27)

*Citations did not provide evidence matching questions of interest*

Studies considered
for evidence report
(n = 8)

**Question 8: Adipokines, including leptin, adiponectin and resistin**

Records identified through searching of PubMed (n=102), Embase (n=335) ,Cochrane Library (n = 10)

Total records screened after duplicates publications removed
(n =358)

Total excluded after title and abstract screening (n = 126)

Review or Meta-analysis or Case report or Conferrence abstract or letter or Animal model (n = 154)

Citations did not match study designs of interest, did not examine populations or interventions of interest, or did not report outcome measures of interest (n = 41)

No full-text((n = 4)

Full-text articles assessed for eligibility
(n = 33)

Full-text articles excluded (n = 5)

*Citations did not provide evidence matching questions of interest*

Studies considered
for evidence report
(n = 28)

**Question 9**: **VEGF**

Records identified through searching of PubMed (n=57), Embase (n=163), Cochrane Library (n = 2022)

Total records screened after duplicates and
non-English or non-Chinese publications removed
(n = 2180)

Total excluded after title and abstract screening (n = 60)

*Citations did not match study designs of interest, did not examine populations or interventions of interest, or did not report outcome measures of interest* (n = 1952)

Type of work,including review or meta-analysis or case report or conferrence abstract or letter or animal model or editorial or notes (n = 144)

*No full-text*(n = 1)

Full-text articles assessed for eligibility
(n = 23)

Full-text articles excluded (n =7)

*Citations did not provide evidence matching questions of interest*

Studies considered
for evidence report
(n = 16)

**Question 10**: **Calprotectin**

Records identified through searching of PubMed (n=99), Embase (n=354) and Cochrane Library (n = 15)

Total records screened after duplicates and
non-English publications removed
(n = 360)

Total excluded after title and abstract screening (n = 272)

*Citations did not match study designs of interest, did not examine populations or interventions of interest, or did not report outcome measures of interest*

Full-text articles assessed for eligibility
(n = 88)

Full-text articles excluded (n = 56)

*Citations did not provide evidence matching a question of interest*

Studies considered
for evidence report
(n =7)

Studies excluded during data extraction
(n =0)

Studies matched

to this part
(n = 7)

**Question 11: non-coding RNA**

Records identified through searching of PubMed (n=332), Embase (n=94) and Cochrane Library (n=1)

Total records screened after duplicates and
non-English publications removed
(n = 341)

Total excluded after title and abstract screening (n = 273)

*Citations did not match study designs of interest, did not examine populations or interventions of interest, or did not report outcome measures of interest*

Full-text articles assessed for eligibility
(n = 68)

Full-text articles excluded (n = 31)

*Citations did not provide evidence matching a question of interest*

Studies considered
for evidence report
(n = 37)

Studies excluded during data extraction
(n = 9)

Studies matched

to this part
(n = 28)

**Question 12**: **Inflammatory cytokines including IL-6, IL-17 and TNF-α**

Records identified through searching of PubMed (n=1899), Embase (n=1233) and Cochrane Library (n = 83)

Total records screened after duplicates publications removed
(n = 2660)

Total excluded after title and abstract screening (n = 111)

*Citations did not match study designs of interest, did not examine populations or interventions of interest, or did not report outcome measures of interest* (n = 624)

Review or Meta-analysis or Case report or Conferrence abstract or letter or Animal model (n = 962)

No full-text(n = 685)

Full-text articles assessed for eligibility
(n = 278)

Full-text articles excluded (n = 228)

*Citations did not provide evidence matching questions of interest*

Studies considered
for evidence report
(n = 50)

**Question 13**: **Peripheral lymphocyte subsets**

Additional records identified

through other sources(n=10)

Records identified through

database searching (n = 2972)

Records after duplicates

removed (n=2282)

Records excluded on the basis of title a

abstract (n = 1,898)

Records screened

(n = 2,282)

Full texts excluded (n = 289), with reasons

(i) Not original articles (reviews) (n = 22)

(ii) Not human experiment (n =28)

(iii)Not research about AS patient (n=50)

(iv) Not research about peripheral blood (n =9)

(v) Not report the proportion of lymphocyte subsets (n = 133)

(vi) No comparison group (n = 47)

Full texts assessed

For Eligibility

(n = 384)

Studies included in this meta-analysis (n = 95)

**Question 14**: **Bone turnover markers**

Records identified through searching of PubMed (n=398), Embase (n=1095) ,Cochrane Library (n = 16) and CNKI (n=186)

Total records screened after duplicates and
non-English or non-Chinese publications removed
(n = 1495)

Total excluded after title and abstract screening (n = 1387)

*Citations did not match study designs of interest, did not examine populations or interventions of interest, or did not report outcome measures of interest* (n = 363)

Type of work,including review or meta-analysis or case report or conferrence abstract or letter or animal model or editorial or notes (n = 857)

*No full-text*(n = 167)

Full-text articles assessed for eligibility
(n = 108)

Full-text articles excluded (n =49)

*Citations did not provide evidence matching questions of interest*

Studies considered
for evidence report
(n = 59)

**Question 15**: **C1M, C2M, C3M, C6M and VICM**

Records identified through searching of PubMed (n=20), Embase (n=58) and Cochrane Library (n =3)

Total records screened after duplicates and
non-English publications removed
(n = 72)

Total articles excluded after title and abstract screening (n = 52)

*Citations did not match study designs of interest, did not examine populations or interventions of interest, or did not report outcome measures of interest*

Full-text articles assessed for eligibility
(n =20)

Full-text articles excluded (n = 9)

*Citations did not provide evidence matching a question of interest*

Studies considered
for evidence report
(n =11)

Studies excluded during data extraction
(n = 1)

Studies matched

to this part
(n = 10)

**Question 16**: **Sclerostin**

Records identified through searching of PubMed (n=72), Embase (n=60), Cochrane Library (n = 84)

Total records screened after duplicates and
non-English or non-Chinese publications removed
(n =54 )

Total excluded after title and abstract screening (n = 7)

*Citations did not match study designs of interest, did not examine populations or interventions of interest, or did not report outcome measures of interest* (n =98 )

Type of work,including review or meta-analysis or case report or conferrence abstract or letter or animal model or editorial or notes (n =24 )

*No full-text*(n =3 )

Full-text articles assessed for eligibility
(n =30 )

Full-text articles excluded (n =10)

*Citations did not provide evidence matching questions of interest*

Studies considered
for evidence report
(n = 20)

**Question 17**: **DKK-1**

Records identified through searching of PubMed (n=65), Embase (n=281) and Cochrane Library (n =3)

Total records screened after duplicates and
non-English publications removed
(n = 291)

Total excluded after title and abstract screening (n = 171)

*Citations did not match study designs of interest, did not examine populations or interventions of interest, or did not report outcome measures of interest*

Full-text articles assessed for eligibility
(n =118)

Full-text articles excluded (n = 98)

*Citations did not provide evidence matching a question of interest*

Studies considered
for evidence report
(n =20)

Studies excluded during data extraction
(n = 0)

Studies matched

to this part
(n = 20)

**Question 18: OPG/RANKL/RANK**

Records identified through searching of PubMed (n=309), Embase (n=526) and Cochrane Library (n=2)

Total records screened after duplicates and
non-English publications removed
(n = 311)

Total excluded after title and abstract screening (n = 193)

*Citations did not match study designs of interest, did not examine populations or interventions of interest, or did not report outcome measures of interest*

Full-text articles assessed for eligibility
(n = 94)

Full-text articles excluded (n = 71)

*Citations did not provide evidence matching a question of interest*

Studies considered
for evidence report
(n = 25)

Studies excluded during data extraction
(n = 2)

Studies matched

to this part
(n = 23)

**Question 19**: **MMP3**

Records identified through searching of PubMed (n=92), Embase (n=116) and Cochrane Library (n = 6)

Total records screened after duplicates and
non-English publications removed
(n = 160)

Total excluded after title and abstract screening (n = 75)

*Citations did not match study designs of interest, did not examine populations or interventions of interest, or did not report outcome measures of interest*

Full-text articles assessed for eligibility
(n = 85)

Full-text articles excluded (n = 61)

*Citations did not provide evidence matching questions of interest*

Studies considered
for evidence report
(n = 24)

Studies excluded during data extraction
(n = 4)

Studies matched

to this part
(n = 20)

**Question 20**: **BMP2**

Records identified through searching of PubMed (n=27), Embase (n=126) and Cochrane Library (n = 5)

Total records screened after duplicates and
non-English publications removed
(n = 129)

Total excluded after title and abstract screening (n = 76)

*Citations did not match study designs of interest, did not examine populations or interventions of interest, or did not report outcome measures of interest*

Full-text articles assessed for eligibility
(n = 53)

Full-text articles excluded (n = 45)

*Citations did not provide evidence matching questions of interest*

Studies considered
for evidence report
(n = 8)

Studies excluded during data extraction
(n = 1)

Studies matched

to this part
(n = 7)

**Question 21: TNC**

**Flowchart of the Study Selection Process**

Records identified through searching of PubMed (n=8), Embase (n=16), Cochrane Library (n = 0)

Total records screened after duplicates and

non-English publications removed
(n =17)

Total excluded after title and abstract screening (n = 7)

Review or Meta-analysis or Case report or Conferrence abstract or letter or Animal model (n = 3)

Full-text articles assessed for eligibility
(n =7)

Full-text articles excluded (n =3)

*Citations did not provide evidence matching questions of interest*

Studies considered
for evidence report
(n = 4)

**Question 22:** **Gut microbiota**

Records identified through searching of PubMed (n=218), Embase (n=97) and Cochrane Library (n=3)

Total records screened after duplicates and
non-English publications removed
(n = 264)

Total excluded after title and abstract screening (n = 187)

*Citations did not match study designs of interest, did not examine populations or interventions of interest, or did not report outcome measures of interest*

Full-text articles assessed for eligibility
(n = 57)

Full-text articles excluded (n = 28)

*Citations did not provide evidence matching a question of interest*

Studies considered
for evidence report
(n = 29)

Studies excluded during data extraction
(n = 5)

Studies matched

to this part
(n = 24)

**Question 23: Metabonomics signature**

Records identified through searching of PubMed (n=114), Embase (n=61) and Cochrane Library (n=1)

Total records screened after duplicates and
non-English publications removed
(n = 131)

Total excluded after title and abstract screening (n = 84)

*Citations did not match study designs of interest, did not examine populations or interventions of interest, or did not report outcome measures of interest*

Full-text articles assessed for eligibility
(n = 47)

Full-text articles excluded (n = 27)

*Citations did not provide evidence matching a question of interest*

Studies considered
for evidence report
(n = 20)

Studies excluded during data extraction
(n = 1)

Studies matched

to this part
(n = 19)

**Question 24: NSAIDs-related genes**

Records identified through searching of PubMed (n=1464), Embase (n=7215) and Cochrane Library (n = 25)

Total records screened after duplicates and
non-English publications removed
(n = 7705)

Total excluded after title and abstract screening (n = 7488)

*Citations did not match study designs of interest, did not examine populations or interventions of interest, or did not report outcome measures of interest*

Full-text articles assessed for eligibility
(n = 217)

Full-text articles excluded (n = 202)

*Citations did not provide evidence matching questions of interest*

Studies considered
for evidence report
(n = 15)

Studies excluded during data extraction
(n = 1)

Studies matched

to this part
(n = 14)

**Question 25: SSZ-related genes**

Records identified through searching of PubMed (n=94), Embase (n=221) and Cochrane Library (n =7)

Total records screened after duplicates and
non-English publications removed
(n = 89)

Total excluded after title and abstract screening (n =70)

*Citations did not match study designs of interest, did not examine populations or interventions of interest, or did not report outcome measures of interest*

Full-text articles assessed for eligibility
(n = 19)

Full-text articles excluded (n = 6)

*Citations did not provide evidence matching questions of interest*

Studies considered
for evidence report
(n = 13)

Studies excluded during data extraction
(n =4)

Studies matched

to this part
(n = 9)

**Question 26: Anti-drug antibodies**

Records identified through searching of PubMed (n=1822), Embase (n=18), Cochrane Library (n = 35)

Total records screened after duplicates removed
(n =1855 )

Total excluded after title and abstract screening (n =116)

*Citations did not match study designs of interest, did not examine populations or interventions of interest, or did not report outcome measures of interest* (n =924)

Type of work,including review or meta-analysis or case report or conferrence abstract or letter or animal model or editorial or notes (n =680)

*No full-text*(n =1)

Full-text articles assessed for eligibility
(n =134)

Full-text articles excluded (n =114)

*Citations did not provide evidence matching questions of interest*

Studies considered
for evidence report
(n = 20)
