# Supplementary material for: A guideline on biomarkers in the diagnosis and evaluation in axial spondyloarthritis
Source: Front Immunol. 2024 Oct 30;15:1394148. doi: 10.3389/fimmu.2024.1394148 (PMC11557325; doi:10.3389/fimmu.2024.1394148)
Supplement: Supplementary file 5 [file Table5.docx]

**SUPPLEMENTARY APPENDIX 5: Characteristics of the included studies**

**Question 1**: **HLA-B27**

| Author | Publish year | Design | Country | Patient diagnosis | Patient number | Control type | Control number | Age (year) Patient/control | NOS score | Database | Reference number |
| --- | --- | --- | --- | --- | --- | --- | --- | --- | --- | --- | --- |
| Jajić | 1979 | Cross-sectional | Yugoslavia | LBP | 652 | no-IBP | 302 | 15-70 year | 3 | PubMed | [1] |
| Sadowska-Wróblewska | 1983 | cohort | Poland | eAS | 70 | - | - | - | 3 | PubMed | [2] |
| Deesomchok | 1985 | cohort | Thailand | AS | 61 | - | - | - | 3 | PubMed | [3] |
| Sampaio-Barros | 2001 | cohort | Brazil | uSpA | 68 | - | - | - | 4 | PubMed, Embase | [4] |
| Brandt | 2007 | Cohort | Germany | IBP | 350 | - | - | 40(16,75) | 3 | PubMed | [5] |
| Dincer | 2008 | cohort | Turkey | AS | 111 | - | - | 33.58±11.96 | 4 | PubMed | [6] |
| Bennett | 2008 | cohort | Spain | IBP | 55 | - | - | - | 5 | PubMed | [7] |
| Nazarinia | 2009 | Cross-sectional | Iran | HLA-B27(+) | 72 | HLA-B27(-) | 26 | - | 3 | PubMed | [8] |
| Liao | 2009 | cohort | China | local residents | 10921 | - | - | - | 5 | PubMed | [9] |
| Aggarwal | 2009 | cohort | India | AS | 70 | - | - | - |  | PubMed | [10] |
| Liu | 2010 | Cohort | China | suspected AS | 1016 | HC | 167 | 38.9±9.1 and 25.1±8.6 | 6 | PubMed | [11] |
| van Onna | 2011 | cohort | Netherlands | IBP | 68 | - | - | 34.9(10.3) | 5 | PubMed | [12] |
| Liao | 2011 | Cross-sectional | China | HLA-B27(+)uSpA | 135 | HLA-B27(-)uSpA | 57 | 22.7±6.5/26.5± 8.3 | 5 | PubMed | [13] |
| Chung | 2011 | cohort | France | IBP | 708 | - | - | 33.6 | 4 | PubMed | [14] |
| De Carvalho | 2012 | cohort | Brazil | SpA | 1050 | - | - | - | 4 | PubMed, Embase | [15] |
| van den Berg | 2013 | cohort | Netherlands | CBP | 157 | - | - | 31.2(12.6) | 6 | PubMed | [16] |
| Qi | 2013 | Cross-sectional | China | AS | 846 | HC | 959 | 28.3±8.8/48.5±6.8 | 5 | PubMed | [17] |
| Peláez-Ballestas | 2013 | cohort | Mexican | Community individual | 758 | - | - | - | 4 | PubMed | [18] |
| Tomero | 2014 | cross-sectional | Spain | SpA | 538 | Non-SpA/Unlikely SpA | 182/55 | 33(7)/33(7)/34(7) | 6 | PubMed | [19] |
| Kassimos | 2014 | cohort | Greece | young Greek males | 347184 | - | - | 25.3(19.2-31.3) | 5 | PubMed | [20] |
| Nakashima | 2016 | Cohort | Japan | AS | 72 | - | - | - |  | PubMed, Embase | [21] |
| Burgos-Vargas | 2016 | Cross-sectional | 19 countries in Latin America | nr-axSpA/AS | 266/491 | Other IBP | 157 | - | 5 | PubMed, Embase | [22] |
| Bautista-Molano | 2016 | cohort | Colombian | SpA | 581 | - | - | 35± 13.5 | 3 | PubMed | [23] |
| Bandinelli | 2016 | cohort | Italy | SpA | 135 | - | - | - | 3 | PubMed | [24] |
| Arnbak | 2016 | cohort | Southern Denmark | IBP | 759 | - | - | - | 5 | PubMed | [25] |
| Fırat | 2017 | cross-sectional | Turkey | HLA-B27(+)AS | 115 | HLA-B27(-)AS | 48 | 27.6±9.0/- | 2 | PubMed | [26] |
| Ez-Zaitouni | 2017 | cohort | Netherlands, Norway and Italy | CBP | 500 | - | - | 29.3 (8.3) | 4 | PubMed | [27] |
| Tong | 2018 | cohort | China | students | 3770 | - | - | 19.48±2.80 | 4 | PubMed | [28] |
| Endo | 2019 | cohort | Japan | SpA | 114 | - | - | 57(48-72) | 4 | PubMed | [29] |
| Passalent | 2022 | cohort | Canada | LBP | 405 | - | - | 37 (29-44)/- | 6 | PubMed | [30] |
| Edara | 2022 | Cross-sectional | India | rSpA/nrSpA | 16/12 | nrSpA | 12 | 28.5±6.3 | 3 | PubMed | [31] |
| Chung | 2022 | cross-sectional | China | axSpA/no-IBP | 447/122 | no-IBP | 122 | 44.9±14.0/48.1±15.0 | 6 | Embase | [32] |
| Puhakka | 2004 | Cross-sectional | Denmark | HLA-B27(+) SpA | 26 | HLA-B27(-) SpA | 15 | - | 3 | PubMed | [33] |
| Ma | 2012 | Cross-sectional | China | AS in Southern of China | 109 | AS in Northern of China | 101 | 28.6±9.7/28.3±12.8 | 5 | PubMed | [34] |
| Londono | 2012 | Cross-sectional | Spain | SpA | 62 | HC | 46 | 31.9±9.9/- | 4 | PubMed, Embase | [35] |
| Weiss | 2016 | Cross-sectional | American | ERA/PSe | 40 | HC | 14 | 14.1(2.7)/13.9 (2.4) | 4 | PubMed | [36] |
| Huerta-Sil | 2006 | Cohort | Mexican | uSpA | 62 | - | - | - | 4 | PubMed | [37] |
| Sampaio-Barros | 2010 | Cohort | Brazil | uSpA | 111 | Remission | - | 53.2±12.5 and - | 5 | PubMed | [38] |
| Bakker | 2019 | Cohort | Europe | IBP | 188 | - | - | 31.0±8.2 | 6 | PubMed | [39] |
| Lorenzin | 2020 | Cohort | Italy | LBP | 75 | - | - | 28.51±8.05 | 7 | PubMed | [40] |
| Baraliakos | 2014 | Cohort | United States | AS | 867 | - | - | 43(36-51) | 6 | Embase | [41] |
| Al-Saleh | 2022 | Cohort | Middle Eastern Countries | axSpA | 309 | - | - | - | 7 | Cochrane Library | [42] |

**Question 2**: **HLA-B27 subtypes**

| Author | Publish year | Design | Country | Patient diagnosis | Patient number | Control type | Control number | Age (year) Patient/control | NOS score | Database | Reference number |
| --- | --- | --- | --- | --- | --- | --- | --- | --- | --- | --- | --- |
| Yi | 2013 | Cross-sectional | China | AS | 360 | non rheumatic disease | 350 | - | 4 | PubMed, Embase | [43] |
| Siala | 2009 | Cross-sectional | Tunisia | AS | 42 | HC | 100 | - | 5 | PubMed, Embase | [44] |
| Ren | 1997 | Cross-sectional | Singapore | AS | 50 | B27+ HC | 45 | - | 3 | PubMed | [45] |
| Qi | 2013 | Cross-sectional | China | AS | 846 | HC | 959 | 28.3 ± 8.8/48.5±6.8 | 6 | PubMed | [17] |
| Pazár | 2010 | Cross-sectional | Hungary | AS | 297 | HC | 200 | 39.2±5.6/45.2±4.1 | 6 | PubMed | [46] |
| Park | 2009 | Cross-sectional | Korea | AS | 143 | HLA-B27(+) HC | 32 | 35.11 (9.27)/ 33.69 (14.50) | 6 | PubMed | [47] |
| Mou | 2010 | Cross-sectional | China | AS | 505 | HC | 1368 | JAS 21.3±7.6, AAS 30.5±7.6/? | 6 | PubMed, Embase | [48] |
| Ma | 2006 | Cross-sectional | China | AS  uSpA | 111  17 | HLA-B27(+) HC | 14  169 | - | 6 | PubMed, Embase | [49] |
| Luo | 2019 | Cross-sectional | China | AS | 235 | HC | 261 | - | 5 | PubMed, Embase | [50] |
| Lopez-Larrea | 1995 | Cross-sectional | Spain | AS | 64 | HC | 9 | - | 5 | PubMed, Embase | [51] |
| Lodhi | 2019 | Cross-sectional | Pakistan | AS | 49 | HC | 18 | 32.57 ±9.0/ 15.33 years ±11.59 | 5 | PubMed | [52] |
| Liu | 2010 | Cross-sectional | China | AS | 172 | HC | 145 | 18-57/20-56 | 6 | PubMed | [53] |
| Kchir | 2010 | Cross-sectional | Tunisia | AS | 100 | HC | 100 | 38.4 ± 12.3 | 6 | PubMed, Embase | [54] |
| Hou | 2007 | Cross-sectional | China | AS | 314 | HLA-B27(+) non AS | 71 | - | 4 | PubMed, Embase | [55] |
| Harfouch | 2011 | Cross-sectional | Syrian | AS | 50 | HC | 217 | - | 5 | PubMed, Embase | [56] |
| Gonzalez-Roces | 1997 | Cross-sectional | worldwide | AS | 746 | HC | 235 | - | 3 | PubMed, Embase | [57] |
| Diyarbakir | 2012 | Cross-sectional | Turkey | AS | 43 | HC | 39 | - | 4 | PubMed, Embase | [58] |
| Cipriani | 2003 | Cross-sectional | Venezuela | AS | 48 | HC | 55 | - | 6 | PubMed, Embase | [59] |
| Chou | 2003 | Cross-sectional | China | AS | 82 | HLA-B27(+) HC | 47 | - | 4 | PubMed, Embase | [60] |
| Chavan | 2011 | Cross-sectional | India | AS | 81 | HLA-B27(+) HC | 29 | - | 5 | PubMed, Embase | [61] |
| Birinci | 2006 | Cross-sectional | Turkey | AS | 38 | HLA-B27(+) HC | 47 | - | 5 | PubMed, Embase | [62] |
| Ben Radhia | 2008 | Cross-sectional | Tunisia | AS | 121 | HLA-B27(+) HC | 39 | 36.5±15.2/- | 4 | PubMed | [63] |
| Alaez | 2007 | Cross-sectional | israel | AS | 24 | HC | 51 | 25 (20–40)/ 28 (19–41) | 5 | PubMed, Embase | [64] |
| Acar | 2012 | Cross-sectional | Turkey | AS | 51 | HLA-B27(+) non rheumatic disease | 948 | - | 4 | PubMed, Embase | [65] |
| Varnavidou-Nicolaidou | 2004 | Cross-sectional | Greek Cypriots | AS | 31 | HLA-B27(+) HC | 60 | - | 3 | PubMed | [66] |
| Lopez-Larrea | 2006 | Cross-sectional | Spain, Portugal | AS  AS | 71  55 | HLA-B27(+) HC | 105  57 | - | 5 | PubMed | [67] |
| Gonzalez | 2002 | Cross-sectional | Spain | AS | 89 | HLA-B27(+) HC | 97 | - | 5 | PubMed, Embase | [68] |
| Díaz-Peña | 2016 | Cross-sectional | Spain | AS | 367 | HLA-B27(-) HC  HLA-B27(+) HC | 462  549 | - | 3 | PubMed | [69] |
| Chen | 2002 | Cross-sectional | China | AS | 184 | HC | 20000 | - | 5 | PubMed | [70] |
| Yamaguchi | 1996 | Cross-sectional | Japan | AS | 20 | HC | 11 | - | 3 | PubMed, Embase | [71] |
| Wu | 2020 | Cross-sectional | China | AS | 318 | HC | 89 | 29.55 ± 8.83/ 39.86 ± 18.03 | 5 | PubMed, Embase | [72] |
| Van Gaalen | 2012 | Cross-sectional | worldwide | AS | 929 | HLA-B27(+) HC | 5990 | - | - | PubMed | [73] |
| Oguz | 2004 | Cross-sectional | Turkey | SpA | 49 | HC | 55 | - | 4 | PubMed, Embase | [74] |
| Mou | 2015 | Cross-sectional | China | JAS | 145 | AAS | 360 | - | 4 | PubMed, Embase | [75] |
| Lopez-Larrea | 1995 | Cross-sectional | India  Thailand | AS | 45  45 | HC | 17  17 | - | 3 | PubMed, Embase | [76] |
| Kanga | 1996 | Cross-sectional | India | AS | 24 | HC | 17 | - | 6 | PubMed | [77] |
| Fouladi | 2009 | Cross-sectional | Iran | AS | 40 | HC | 17 | - | 4 | PubMed | [78] |
| Liu | 2010 | Cross-sectional | China | AS | 130 | HC | 61 | - | 3 | PubMed | [79] |
| Yang | 2014 | Meta-analysis | China | AS | 3410 | HC | 1735 | - | 5 | PubMed, Embase | [80] |
| Lin | 2017 | Meta-analysis | China | AS | - | HC | - | - | 5 | PubMed, Embase | [81] |

**Question 3**: **Genes**

| Author | Publish year | Design | Country | Patient diagnosis | Patient number | Control type | Control number | Age (year) Patient/control | NOS score | Database | reference number |
| --- | --- | --- | --- | --- | --- | --- | --- | --- | --- | --- | --- |
| Cortes | 2013 | cross-sectional | Europe, East Asia | AS | 9069/1550 | - | 13578/1567 | - | 3 | Embase; PubMed | [82] |
| Ellinghaus | 2016 | cross-sectional | Europe | AS | 8726 | HC | 34213 | - | 4 | Embase; PubMed | [83] |
| Robinson | 2016 | cross-sectional | Europe | AS | 5040 | HC | 21133 | - | 4 | Embase; PubMed | [84] |
| Soomro | 2022 | cross-sectional | Europe | PSA | 4072 | HC | 9965 | - | 3 | Embase; PubMed | [85] |
| Bettencourt | 2013 | cross-sectional | Portugal | AS | 200 | HC | 559 | ＞35/- | 3 | Embase; PubMed | [86] |
| Lin | 2012 | cross-sectional | China | AS | 1837 | HC | 4231 | - | 3 | Embase; PubMed | [87] |
| Zvyagin | 2010 | cross-sectional | China | HLA-B27(+) AS | - | HLA-B27(+) HC | - | 21~63 / 21~63 | 6 | Embase; PubMed | [88] |
| Zhang | 2014 | cross-sectional | China | AS | 602 | HC | 619 | 30.4±10.4/ 31.4±6.7 | 4 | Embase; PubMed | [89] |
| Zhang | 2015 | cross-sectional | China | AS | - | HC | - | 28.9 ± 8.9 / 27.9 ± 7.9 | 5 | Embase; PubMed | [90] |
| Wiśniewski | 2019 | cross-sectional | Poland | AS | 180 | HC | 506 | 52.9 (19–80) / 33.9 (14–63) | 5 | Embase; PubMed | [91] |
| Daryabor | 2014 | cross-sectional | Iran | AS | 294 | HC | 352 | 38.17 / 37.42 | 3 | PubMed | [92] |
| Burton | 2007 | cross-sectional | UK, US | AS | 2000 | HC | 3000 | - | 3 | Embase; PubMed | [93] |
| Reveille | 2010 | cross-sectional | Australia, UK, US | AS | 2053 | HC | 5140 | - | 4 | Embase; PubMed | [94] |
| Rostami | 2020 | crosss-sectional | Norway | AS | 164 | HC | 49032 | 41.9±11.1/47.4±17.2 | 5 | Embase; PubMed | [95] |
| Jung | 2016 | cross-sectional | Korea | AS | 861 | HC | 1043 | 23.4±8.6/ 32.4±10.3 | 5 | Embase; PubMed | [96] |
| Li | 2021 | cross-sectional | European, East Asian | AS | 15585 | HC | 20452 | - | 6 | Embase; PubMed | [97] |
| Huang | 2020 | cross-sectional | UK | AS with acute anterior uveitis | 2752 | AS without acute anterior uveitis | 3836 | - | 5 | Embase; PubMed | [98] |
| Thomas | 2017 | cross-sectional | UK, Germany, Taiwan (China), Canada, Columbia, Turkey | axSpA | 4428 | HC | 9638 | - | 6 | Embase; PubMed | [99] |
| van Gaalen | 2013 | cross-sectional | Netherlands | AS | 154 | HC | 5584 | 43(16-66)/- | 5 | Embase; PubMed | [100] |
| Wei | 2015 | cross-sectional | China | AS | 471 | HC | 557 | 39.0 ± 11.3/39.0 ± 12.2 | 5 | Embase; PubMed | [101] |
| Wei | 2004 | cross-sectional | China | HLA-B27(-) AS | 41 | HLA-B27(-) HC | 11383 | (25-52)/- | 5 | Embase; PubMed | [102] |
| Atouf | 2012 | cross-sectional | Morocco | AS | 46 | HC | 183 | (20-65)/- | 5 | Embase; PubMed | [103] |
| Kchir | 2010 | cross-sectional | Tunisia | AS | 100 | HC | 100 | 38.4 ± 12.3/- | 6 | Embase; PubMed | [54] |
| Reveille | 2019 | cross-sectional | European, Asian, Africa | AS | 2457 | HC | 1581 | - | 6 | Embase; PubMed | [104] |
| Santos | 2017 | cross-sectional | Colombia | AS | 87 | HC | 100 | 35.91 ± 12.7/- | 5 | Embase; PubMed | [105] |
| Helenius | 2004 | cross-sectional | Finland | AS/SpA | 19/21 | RA/MCTD | 24/19 | 41.4 ± 11.3/38.9± 9.6/48.9 ± 11.4/45.1 ± 15.1 | 5 | Embase; PubMed | [106] |
| Jaakkola | 2006 | cross-sectional | Finland | AS | 237 | family members without AS | 383 | - | 6 | Embase; PubMed | [107] |
| La Nasa | 1993 | cross-sectional | Italy | AS | 25 | HC | 44 | 39(22-64)/35(21-46) | 5 | Embase; PubMed | [108] |
| Madhavan | 2006 | cross-sectional | India | AS | 65 | HC | 124 | - | 4 | Embase; PubMed | [109] |
| Monowarul Islam | 1995 | cross-sectional | Japan | AS with acute anterior uveitis | 20 | AS without acute anterior uveitis | 22 | - | 4 | Embase; PubMed | [110] |
| Perez-Guijo | 2002 | cross-sectional | Spain | AS | 115 | HC | 748 | 44.8 ± 12.1/- | 5 | Embase; PubMed | [111] |
| Ploski | 1995 | cross-sectional | Norway | juvenile AS | 63 | adult AS | 44 | 32.8 ± 13.3/58.0 ± 12.3 | 5 | Embase; PubMed | [112] |
| Yang | 2010 | cross-sectional | China | AS | 63 | HC | 106 | - | 4 | CNKI | [113] |
| Cai | 2005 | cross-sectional | China | AS | 110 | HC | 134 | 30 ± 12/33 ± 9 | 5 | CNKI | [114] |

**Question 4: Antibodies**

| Author | Publish year | Design | Country | Patient diagnosis | Patient number | Control type | Control number | Age (year) Patient/control | NOS score | Database | Reference number |
| --- | --- | --- | --- | --- | --- | --- | --- | --- | --- | --- | --- |
| Baraliakos | 2014 | cross-sectional | Germany | axSpA | 94 | non-SpA | 51 | 43.0±11.0/57.7 ±14.5 | 3 | Pubmed | [115] |
| Baerlecken | 2014 | cross-sectional | Germany | axSpA | 216 | non-SpA | 285 | 46±29/- | 4 | Pubmed | [116] |
| Abdelaziz | 2021 | cross-sectional | Egypt | SpA | 50 | non-SpA | 30 | 38.2±12.20/ - | 4 | Pubmed | [117] |
| Do | 2021 | cross-sectional | Germany | r-axSpA | 155 | non-SpA | 151 | 55.5 ±11.4/- | 5 | Pubmed | [118] |
| de Winter | 2018 | cross-sectional | Netherlands | axSpA | 274 | CBP | 319 | 30.7±7.9/31.4 ±8.5 | 5 | Pubmed,  Embase | [119] |
| Riechers | 2019 | cross-sectional | Germany | axSpA | 100 | IBP | 149 | 28.8/42.7 | 3 | Pubmed, Embase | [120] |
| Hu | 2020 | cross-sectional | China | axSpA | 97 | HC | 60 | -/32.2±6.6 | 4 | Pubmed | [121] |
| Ziade | 2019 | cross-sectional | Lebanon | axSpA | 49 | Blood Donors | 102 | 34/30 | 3 | Pubmed, Embase | [122] |
| Çolak | 2021 | cross-sectional | Turkey | AS | 111 | IBD | 108 | 43.17±11.96/39.5±11.5 | 3 | Pubmed | [123] |
| Liu | 2019 | cross-sectional | China | SpA | 71 | HC | 70 | - | 3 | Pubmed, Embase | [124] |
| Tsui | 2014 | cross-sectional | Canada | AS | 56 | MBP | 28 | - | 3 | Pubmed, Embase | [125] |
| Rosenberg | 1979 | cross-sectional | London | AS | 88 | PsA | 52 | - | 3 | Pubmed | [126] |
| de Vries | 2010 | cross-sectional | Netherlands | AS with IBD | 130 | AS without IBD | 52 | - | 3 | Pubmed | [127] |
| Matzkies | 2012 | cross-sectional | United States | AS | 47 | HC | 42 | 47 (20-85)/39 (27-70) | 4 | Pubmed | [128] |
| Stone | 2004 | cross-sectional | Canada | AS | 94 | HC | 33 | - | 4 | Pubmed | [129] |
| Stebbings | 2002 | cross-sectional | United Kingdom | AS | 15 | Blood Donors | 15 | - | 3 | Pubmeb | [130] |
| Tiwana | 1997 | cross-sectional | United Kingdom | active AS | 35 | Blood Donors | 60 | 47(31-80)/35(19-57) | 3 | Pubmed | [131] |
| Collado | 1994 | cross-sectional | Spain | AS | 84 | NIA and RA | 41/22 | 41±12/42 ±12、57±9 | 3 | Pubmed, Embase | [132] |
| Cooper | 1988 | cross-sectional | Glasgow | AS | 64 | HC | 30 | - | 4 | Pubmed | [133] |
| Csángó | 1987 | cross-sectional | Norway | HLA-B27(+) AS and healthy individuals | 151 | HC | 37 | - | 3 | Pubmed | [134] |
| Kihlström. | 1989 | cross-sectional | Swden | AS | 15 | Blood Donors | 37 | 35 (24-58)/32 (18-54) | 3 | Pubmed | [135] |
| Kumar | 2014 | cross-sectional | India | AS | - | ReA | - | - | 3 | Pubmed, Embase | [136] |
| Tsuchiya | 1990 | cross-sectional | Florida | AS | 75 | HC | 59 | 39.8±11.1/38.3+11 .4 | 3 | Pubmed | [137] |
| Zambrano-Zaragoza | 2009 | cross-sectional | Mexico | AS | 28 | HC | 28 | 31.9±10.1/26.6±6.4 | 3 | Pubmed | [138] |
| Andretta | 2012 | cross-sectional | Brazil | SpA | 70 | HC | 57 | 34.7 ± 12.50/35.47 ± 12.47 | 5 | Pubmed | [139] |
| Aydin | 2008 | cross-sectional | Turkey | AS, uSpA | 175、47 | HC | 103 | - | 4 | Pubmed | [140] |
| Hoffman | 2003 | cross-sectional | Belgium | SpA | 108 | HC | 45 | - | 4 | Pubmed | [141] |
| Mundwiler | 2009 | cross-sectional | United States | AS | 79 | HC | 79 | 36.5(19-61)/30.8(18-10.4) | 3 | Pubmed | [142] |
| Rodrigues | 2015 | cross-sectional | Brazil | SpA | 91 | Crohn disease | 40 | - | 4 | Pubmed | [143] |
| Wallis. | 2013 | cross-sectional | United States | AS | 76 | AS-IBD | 77 | 39.5 (28.5-50.5)/39.0 (31.0-50.0) | 4 | Pubmed | [144] |
| Tani | 1997 | cross-sectional | Japanese | AS | 56 | HC | 60 | 42(20–69)/45(31–60) | 4 | Pubmed | [145] |
| Dominguez-López | 2002 | cross-sectional | Mexico | HLA-B27+ AS | 49 | HLA-B27- HC | 101 | - | 3 | Pubmed | [146] |
| Mäki-Ikola | 1997 | cross-sectional | Finland | AS | 25 | Blood Donors | 100 | - | 3 | Pubmed | [147] |
| Lee | 2020 | Cohort | Korea | AS | 153 | - | - | 37.8 ± 10.9 | 5 | Pubmed | [148] |
| Kim | 2014 | Cohort | United States | AS | 45 | - | - | 31.31 ± 10.77 | 4 | Pubmed | [149] |
| Torok | 2004 | cross-sectional | Germany | SpA | 87 | HC | 145 | 38.5B11.8/44.6B12.5 | 4 | Pubmed | [150] |

**Question 5**: **CRP**

| Author | Publish year | Design | Country | Patient diagnosis | Patient number | Control type | Control number | Age (year) Patient/control | NOS score | Database | Reference number |
| --- | --- | --- | --- | --- | --- | --- | --- | --- | --- | --- | --- |
| Wu | 2021 | case-control | China | AS | 136 | HC | 63 | 33 (27-42) /34 (28-44) | 6 | PubMed, Embase | [151] |
| Kwan | 2019 | cross-sectional | Singapore | axSpA | 280 | - | - | 39 (18‐78) | 6 | PubMed, Embase | [152] |
| Akbal | 2016 | case-control | Turkey | AS | 40 | HC | 40 | 40±0.9/ 40± 0.8 | 5 | PubMed, Embase | [153] |
| Sundaram | 2020 | Cohort | India | AS | 107 | - | - | mean age-29 years | 5 | PubMed, Embase | [154] |
| Navarini | 2022 | Cohort | Italy | AxSpA | 295 | -- | -- | 47 (40–56) | 6 | PubMed, Embase | [155] |
| Ben-Shabat | 2022 | case-control | Israel | AS | 5930 | HC | 29018 | 49.8 (±16.6)  same for AS patients and controls | 5 | PubMed, Embase | [156] |
| Su | 2019 | Cohort | China | AS | 129681 | - | - | Plasma concentration of hs-CRP (mg/L)：<1.00 49.3 ± 12.4；1.00–2.99 51.5 ± 13.0；3.00–9.99 54.4 ± 12.9；≥10.00 55.4 ± 13.5 | 6 | PubMed, Embase | [157] |
| Cowling | 1980 | Cohort | London | AS | 469 | - | - | 34.5 (range 13-72), | 5 | PubMed, Embase | [158] |
| Bedaiwi | 2021 | cross-sectional | Saudi Arabia | SpA | 106 | - | - | 39 ± 14.89 | 6 | PubMed, Embase | [159] |
| Chen | 2015 | Cohort | China | AS | 156 |  |  | 35.55 (12.17) | 5 | PubMed, Embase | [160] |
| Kilic | 2015 | cohort | Turkey | axSpA | 287 |  |  | nr-axSpA 33.40 ± 9.30; AS 37.99 ± 9.03 | 5 | PubMed, Embase | [161] |
| Benhamou | 2010 | RCT | France | axial AS | 851 | - | - | 42.9 (11.8) | 4 | PubMed, Embase | [162] |
| de Vries | 2009 | cohort | Netherlands | AS | 155 | - | - | 42±11 | 5 | PubMed, Embase | [163] |
| Siebuhr | 2019 | case-control | Denmark | AxSpA | 193 | HC | 100 | AS (n=72) 34.5 years;  nr-axSpA(n=121)37.5 years; HC(n=100) 37 years | 4 | PubMed, Embase | [164] |
| Li | 2021 | case-control | China | JoAS | 53 | Nr-axSpA | 53 | JoAS 16.0 (1.0)  Nr-axSpA 17.0 (3.0) | 4 | PubMed, Embase | [165] |
| Huang | 2022 | case-control | China | axSpA | 297 | HC | 71 | - | 5 | PubMed, Embase | [166] |
| Senna | 2012 | case-control | Egypt | AS | 40 | HC | 40 | 33.2 ± 6.4 /31.8 ±5.6 | 5 | PubMed, Embase | [167] |
| Tang | 2018 | case-control | China | AS | 250 | HC | 250 | 34.32 ± 11.25/ 33.28 ± 9.35 | 6 | PubMed, Embase | [168] |
| Sun | 2023 | cohort | China | AS | 196 | - | - | 30.75 ± 9.92 | 4 | PubMed, Embase | [169] |
| Toldi | 2013 | case-control | Hungary | AS | 33 | HC | 29 | 41 (35-45) /55 (46-69) | 5 | PubMed, Embase | [170] |
| Ozgocmen | 2007 | cohort | Turkey | AS | 27 | - | - | 36.41±11.79 | 4 | PubMed, Embase | [171] |
| Mlcoch | 2017 | cohort | Czech Republic | AS | 313 | - | - | 42 (36-53) | 4 | PubMed, Embase | [172] |
| Kang | 2015 | cohort | South Korea | AS | 67 | - | - | 35.3 ± 10.2 | 5 | PubMed, Embase | [173] |
| Ho | 2000 | case-control | China | AS | 24 | HC | 21 | 35.6 (19–54) / 30.2 (22-47) | 4 | PubMed, Embase | [174] |
| Wang | 2008 | case-control | China | AS | 35 | HC | 15 | 35 (16-48) / 39 (22-56) | 4 | PubMed, Embase | [175] |
| Kang | 2014 | cohort | South Korea | AS | 298 | - | - | 33.9±10.9 | 5 | PubMed, Embase | [176] |
| Komsalova | 2020 | case-control | España | SpA | 47 | Not SpA | 86 | 39.7±9.9/ 38.5±9.3 | 4 | PubMed, Embase | [177] |
| Nazıroğlu | 2011 | case-control | Turkey | AS | 13 | HC | 13 | 33.46± 9.14/ 33.38± 9.35 | 5 | PubMed, Embase | [178] |
| Zwolak | 2019 | cohort | Poland | AS | 82 | - | - | 40,7±10,2 (18-76) | 4 | PubMed | [179] |
| Seng | 2018 | cross-sectional | Singapore | AxSpA | 122 |  |  | 37.0 ± 12.5 | 4 | PubMed, Embase | [180] |
| Sahli | 2019 | cross sectional | Tunisia | SpA | 60 | - | - | 44±14 (5–82) | 4 | PubMed, Embase | [181] |
| Hirano | 2022 | cohort | Netherlands | SpA | 708 | - | - | 33.7 (8.6) | 5 | PubMed, Embase | [182] |
| Poddubnyy | 2011 | cohort | Germany | axSpA | 210 | - | - | 37.1±10.6 | 5 | PubMed, Embase | [183] |
| Iervolino | 2012 | cohort | Italy | PsA | 146 | - | - | 45.62 ± 11.82 | 4 | PubMed, Embase | [184] |
| Londono | 2012 | case-control | Navarra | SpA | 62 | HC | 46 | 31.9 ± 9.9 | 3 | PubMed, Embase | [185] |
| Li | 2020 | cross-sectional | China | AS | 167 | - | - | 35.8 (11.2) | 5 | PubMed, Embase | [186] |
| Bansal | 2017 | cohort | India | AS | 254 | - | - | 32.98±11.82 (16 – 74) | 5 | PubMed, Embase | [187] |
| Navarini | 2020 | cohort | Italy | AS | 133 | - | - | 46 (39–54) | 4 | PubMed, Embase | [188] |
| Sebastian | 2017 | cohort | Poland | axSpA | 65 | - | - | 45 (22–71). | 5 | PubMed, Embase | [189] |
| Wendling | 2017 | cohort | France | SpA | 708 | - | - | 33.8 (8.6) | 5 | PubMed, Embase | [190] |
| Weiss | 2016 | cross-sectional | Pennsylvania | JSpA | 40 | HC | 14 | 14.1 (2.7)/ 13.9 (2.4) | 6 | PubMed, Embase | [36] |
| Zong | 2022 | RCT | China | axSpA | 125 |  |  | - | 4 | PubMed, Embase | [191] |
| Kim | 2021 | cohort | South Korea | axSpA | 43 | - | - | 17.9 ± 1.6 | 5 | PubMed, Embase | [192] |
| Braga | 2020 | cohort | Brazil | PsA | 45 | - | - | 50.1 ± 11.5 | 4 | PubMed, Embase | [193] |
| Chen | 2011 | cohort | China | AS | 531 | - | - | No Spinal  Involvement  (n =291) 30.6 ± 9.1  Spinal Involvement  without Fusion  (n = 142) 34.5 ±10.6  Spinal Involvement  with Fusion  (n = 98) 42.4 ± 9.9 | 5 | PubMed, Embase | [194] |
| Yildirim | 2004 | cohort | Turkey | AS | 20 | - | - | - | 5 | PubMed, Embase | [195] |
| Webers | 2015 | cohort | Netherlands | AS | 216 | - | - | 43.6±12.7/- | 7 | Pubmed, Embase | [196] |
| Syrbe | 2015 | cohort | Germany | AS | 86 | HC | 25 | 38.1±11.7 / 40.2±12.2 | 5 | Pubmed, Embase | [197] |
| Kang | 2015 | cohort | Korea | axSpA | 110 | - | - | 31.6±10.6 | 5 | Pubmed, Embase | [198] |
| Poddubnyy | 2016 | cohort | Germany | axSpA | 178 | - | - | 38.1±10.7 | 6 | Pubmed, Embase | [199] |
| Kim | 2016 | cohort | Korea | AS | 610 | - | - | 37.94±8.78 | 5 | Pubmed, Embase | [200] |
| Sohn | 2018 | cross-sectional | Korea | AS | 55 | HC | 26 | 37.8±10.8/35.6±6.8 | 6 | Pubmed, Embase | [201] |
| Deminger | 2018 | cohort | Sweden | AS | 204 | - | - | 50±13/- | 7 | Pubmed, Embase | [202] |
| Pedersen | 2018 | cohort | Denmark | AS | 33 | - | - | 40.3±10.9 | 5 | Pubmed, Embase | [203] |
| Jeong | 2015 | cohort | Korea | AS | 47 | - | - | 25.7±8.6/- | 5 | Pubmed, Embase | [204] |
| Huerta-sil | 2006 | cohort | Mexico | undifferentiated spA | 50 | - | - | 27.6±7.5 | 6 | Pubmed, Embase | [205] |
| Braun | 2016 | cohort | USA | AS | 356 | - | - | - | 5 | Pubmed, Embase | [206] |
| Poddubnyy | 2012 | cohort | Germany | axSpa | 210 | - | - | 37.1±10.6/- | 6 | Pubmed, Embase | [207] |

**Question 6**: **ESR**

| Author | Publish year | Design | Country | Patient diagnosis | Patient number | Control type | Control number | Age (year) Patient/control | NOS score | Database | Reference number |
| --- | --- | --- | --- | --- | --- | --- | --- | --- | --- | --- | --- |
| Wu | 2021 | case-control | China | AS | 136 | HC | 63 | 33.00 (range 27-42)/ 34 (range 28-44) | 5 | Pubmed, Embase | [151] |
| Kim | 2020 | cross-sectional | Korea | AS | 200 | OA | 130 | 31.9±12.5/ 57.7±7.3 | 5 | Pubmed, Embase | [208] |
| de Vires | 2009 | cohort | Netherlands | AS | 155 | - | - | 42±11/- | 6 | Pubmed, Embase | [163] |
| Jung | 2007 | case-control | Korea | AS | 38 | HC | 38 | 29.6±10.7/ 30.1±8.7 | 6 | Pubmed, Embase | [209] |
| Borman | 2001 | case-control | Turkey | AS | 32 | HC | 30 | 35.1±11.4/ 36.3±12.7 | 6 | Pubmed, Embase | [210] |
| Hussein | 1987 | cohort | Germany | JSpA | 12 | - | - | 12.5 (range 2.5-17.5)/- | 5 | Pubmed, Embase | [211] |
| Sheehan | 1986 | cross-sectional | UK | AS | 65 | - | - | 39.8 (range 21-71)/- | 4 | Pubmed, Embase | [212] |
| Cowling | 1980 | case-control | UK | AS | 149 | HC | 60 | 13.5 (range 13-72)/- | 4 | Pubmed, Embase | [158] |
| Wang | 2022 | clinical trial | China | AS | 180 | - | - | -/- | 6 | Embase, Cochrane | [213] |
| Alegre-Sancho | 2021 | cohort | Spain | axSpA/PsA | 131/79 | - | - | 48.0±12.3/50.0±11.7 | 6 | Pubmed, Embase | [214] |
| Zhang | 2021 | cohort | China | SpA treated with TNFi | 165 | SpA received basic treatment | 74 | 28.67±10.21/ 27.3±7.11 | 6 | Pubmed, Embase | [215] |
| You | 2020 | clinical trial | China | AS | 90 | - | - | 20.33±4.56/- | 7 | Pubmed, Embase, Cochrane | [216] |
| Xu | 2019 | case-control | China | AS | 232 | HC | 314 | 62.35±8.21/ 62.02±7.83 | 6 | Pubmed, Embase | [217] |
| Dong | 2019 | cohort | China | AS | 60 | HC | 24 | 30.5±6.67/ 31.28±8.62 | 5 | Pubmed, Embase | [218] |
| Gentileschi | 2018 | cohort | Italy | axSpA | 21 | - | - | 56.57±9.62/- | 6 | Pubmed, Embase | [219] |
| Wei | 2018 | clinical trial | China | nr-axSpA treated with etanercept | 54 | nr-axSpA treated with Placebo | 57 | 32.0±6.8/ 32.2±8.7 | 8 | Pubmed, Embase, Cochrane | [220] |
| Sebastian | 2017 | cohort | Poland | axSpA | 65 | - | - | 45 (range 22-71)/- | 6 | Pubmed, Embase | [189] |
| Lubrano | 2016 | cohort | Italy | axSpA | 174 | - | - | 43.2±12.7/- | 6 | Pubmed, Embase | [221] |
| Mok | 2015 | clinical trial | China | axSpA treated with golimumab | 20 | axSpA treated with pamidronate | 10 | 30.1±10.7/ 36.3±11.4 | 8 | Pubmed, Embase, Cochrane | [222] |
| Kneepkens | 2015 | cohort | Netherlands | AS | 115 | - | - | 42±11/- | 6 | Pubmed, Embase | [223] |
| Korkosz | 2013 | cohort | Poland | AS | 40 | - | - | 38.0±11.4/- | 7 | Pubmed, Embase | [224] |
| Paramarta | 2012 | clinical trial | Netherlands | SpA treated with adalimumab | 20 | SpA treated with placebo | 20 | 41.5±12.8/ 44.4±11.1 | 8 | Pubmed, Embase, Cochrane | [225] |
| Sandhya | 2011 | cohort | India | SpA | 24 | - | - | 31 (range 18-53)/- | 5 | Pubmed | [226] |
| Kim | 2018 | cohort | Korea | axSpa | 119 | - | - | 35±9/- | 6 | Pubmed, Embase | [227] |
| Sohn | 2018 | cross-sectional | Korea | AS | 55 | HC | 26 | 37.8±10.8/35.6±6.8 | 6 | Pubmed, Embase | [201] |
| Deminger | 2018 | cohort | Sweden | AS | 204 | - | - | 50±13/- | 7 | Pubmed, Embase | [202] |
| Jeong | 2015 | cohort | Korea | AS | 47 | - | - | 25.7±8.6/- | 5 | Pubmed, Embase | [204] |
| Webers | 2015 | cohort | Netherlands | AS | 216 | - | - | 43.6±12.7/- | 7 | Pubmed, Embase | [196] |
| Haroon | 2013 | cohort | Canada | AS | 241 | - | - | 41.0±13.0/- | 6 | Pubmed, Embase | [228] |
| Poddubnyy | 2012 | cohort | Germany | axSpa | 210 | - | - | 37.1±10.6/- | 6 | Pubmed, Embase | [207] |
| Poddubnyy | 2011 | cohort | Germany | axSpa | 210 | - | - | 37.1±10.6/- | 7 | Pubmed, Embase | [183] |

**Question 7: SAA**

| Author | Publish year | Design | Country | Patient diagnosis | Patient number | Control type | Control number | Age (year) Patient/ control | NOS score | Database | Reference number |
| --- | --- | --- | --- | --- | --- | --- | --- | --- | --- | --- | --- |
| de Vries | 2009 | Cohort | Netherlands | AS (infliximab) | 155 | - | - | 42±11 | 6 | Pubmed, Embase | [163] |
| van Eijk | 2009 | Cohort | Netherlands | AS (etanercept or infliximab) | 92 | - | - | 43±11.2 | 6 | Pubmed, Embase | [229] |
| Lange | 2000 | Cross-sectional | Germany | AS | 72 | - | - | - | 4 | Pubmed, Embase | [230] |
| Ostensen | 1985 | Cross-sectional/ Cohort | Norway | AS (pregnancy+ post partum) | 13 | HC | 28 | 22~34/18~35 | 6 | pubmed | [231] |
| Hu | 2021 | Cross-sectional | china | AS | 78 | HC | 80 | 32.64±13.93/31.33 ± 12.16 | 6 | Embase | [232] |
| Liu | 2020 | Cross-sectional | China | AS | 36 | HC | 36 | 43±12/43±15 | 6 | Embase | [233] |
|  |  |  |  | AS | 62 | HC | 114 | 33±11/31±8 |  |  |  |
|  |  |  |  | AS | 40 | HC | 40 | 43±12/42±12 |  |  |  |
|  |  |  |  | Stable AS | 28 | HC | 28 | 47±14/43±19 |  |  |  |
|  |  |  |  | Active AS | 26 |  |  | 45±15/43±19 |  |  |  |
| Rademacher | 2019 | Cross-sectional | Berlin | axSpA | 117 | - | - | 42.9±10.2 | 5 | Embase | [234] |
| Londono | 2012 | Cross-sectional | Sabana | SPA | 62 | HC | 46 | 31.9±9.9/- | 6 | Embase | [235] |

**Question 8: Adipokines**

| Author | Publish year | Design | Country | Patient diagnosis | Patient number | Control type | Control number | Age (year) Patient/ control | NOS score | Database | Reference number |
| --- | --- | --- | --- | --- | --- | --- | --- | --- | --- | --- | --- |
| Toussirot | 2007 | case-control | France | AS | 53 | HC | 35 | 44.1 ± 0.6 | 7 | pubmed, Embase | [236] |
| Elolemy | 2013 | case-control | Egypt | AS | 25 | HC | 20 | 29.11 ± 7.86/ 32.12 ± 6.41 | 2 | Embase | [237] |
| Park | 2007 | case-control | Korea | AS | 42 | HC | 42 | 31.0±9.3 | 3 | pubmed, Embase | [238] |
| Miranda-Filloy | 2013 | Cross-sectional study | Spain | AS | 29 | - | - | 50.8 ± 15.0 | 4 | pubmed, Embase | [239] |
| Gonzalez-Lopez | 2017 | case-control | México | AS | 48 | HC | 41 | 44.3 ± 11.4 | 2 | pubmed, Embase | [240] |
| Pishgahi | 2020 | case-control | Iran | AS | 31 | HC | 35 | 42.93 ± 10.48 | 3 | pubmed, Embase | [241] |
| Xie | 2022 | Randomization Study | China | AS | 2252 | HC | 227,338 | -/- | 4 | pubmed, Embase | [242] |
| Park | 2009 | case-control | Korea | AS | 20 | HC | 20 | 30.2 ± 7 | 5 | pubmed, Embase | [243] |
| Kocabas | 2012 | case-control | Turkey | AS | 30 | HC | 30 | 43.93 ± 10.85 | 3 | pubmed, Embase | [244] |
| Mustafa Güler. | 2013 | case-control | Turkey | AS | 108 | HC | 65 | 36.4±11.2/38.2±13.0 | 2 | Embase | [245] |
| Kononoff | 2021 | population-based longitudinal epidemiological study | Finland | SpA | 58 | RA/UA | 47/71 | 44 (13) | 3 | Embase | [246] |
| Rademacher | 2019 | RCT | Germany | AS | 117 | - | - | 42.9 (10.2) | 6 | pubmed, Embase | [234] |
| Sari | 2007 | case-control | Turkey | AS | 28 | HC | 17 | 31 (20–51) | 3 | pubmed, Embase | [247] |
| Wang | 2016 | case-control | China | AS | 120 | HC | 100 | 60 ± 13 | 3 | pubmed, Embase | [248] |
| Genre | 2020 | case-control | Spain | axSpA | 385 | HC | 84 | 46.3 ± 12.1 | 4 | pubmed, Embase | [249] |
| Syrbe | 2015 | case-control | Germany | AS | 86 | HC | 25 | 38.1 ± 11.7 | 7 | pubmed, Embase | [197] |
| Toussirot | 2013 | case-control | France | AS/RA | 31/30 | HC | 51 | 43.8 ± 2.4 | 7 | pubmed, Embase | [250] |
| Kim | 2012 | case-control | Korea | AS | 72 | HC | 20 | 35.1 ± 1.1 | 3 | pubmed, Embase | [251] |
| Hartl | 2017 | ENRADAS trial | Germany | AS | 120 | - | - | 42.9 ± 10.3 | 5 | pubmed, Embase | [252] |
| Toussirot | 2014 | case-control | France | AS/RA | 12/8 | - | - | - | 4 | pubmed, Embase | [253] |
| Rueda-Gotor | 2021 | Cross-sectional study | Spain | axSpA | 510 | - | - | 48.8 ± 12.4 | 5 | pubmed, Embase | [254] |
| Rademacher | 2022 | cohort study | Germany | AS | 137 | - | - | 42.0 ± 10.8 | 5 | pubmed, Embase | [255] |
| Czókolyová | 2021 | cohort study | Hungary | AS | 27 | RA | 36 | 43.6 ± 12.4 (24–72) | 6 | pubmed, Embase | [256] |
| Derdemezis | 2011 | cohort study | Greece | AS | 30 | HC | 30 | 40.6 ± 13.7 | 4 | pubmed, Embase | [257] |
| Miranda-Filloy | 2013 | cohort study | Spain | AS | 30 | - | - | 50.5 ± 14.8 | 4 | pubmed, Embase | [258] |
| Hulejová | 2011 | cohort study | Prague | AS | 26 | - | - | 36 (22–48) | 3 | pubmed, Embase | [259] |
| Inman | 2016 | GO-RAISE | Canada | AS | 356 | - | - | - | 5 | pubmed, Embase | [260] |
| Wagner | 2012 | GO-RAISE | United States | AS | 356 | - | - | - | 7 | Embase | [261] |

**Question 9: VEGF**

| Author | Publish year | Design | Country | Patient diagnosis | Patient number | Control type | Control number | Age (year) Patient/ control | NOS score | Database | Reference number |
| --- | --- | --- | --- | --- | --- | --- | --- | --- | --- | --- | --- |
| Goldberger | 2002 | cross-sectional | Innsbruck | AS | 16 | HC | 8 | 50.4±2.7/- | 3 | PubMed | [262] |
| Drouart | 2003 | cross-sectional | France | SpA | 105 | HC | 64 | 42.7±14.7/59.8±12.8 | 3 | PubMed | [263] |
| Wang | 2016 | cross-sectional | China | AS | 208 | HC | 412 | 32.40 (±9.99)/33.54 (±8.00) | 4 | PubMed | [264] |
| Pedersen | 2011 | Cohort | Denmark | SpA | 60 | - | - | 40 (21–62) | 5 | PubMed | [265] |
| Lin | 2015 | cross-sectional | China | AS | 140 | HC | 90 | 31.8±9.3/30.2±8.2 | 4 | PubMed | [266] |
| Przepiera-Będzak | 2016 | cross-sectional | Poland | AS | 80 | HC | 21 | 50.9±12.8/48.2±13.5 | 4 | PubMed | [267] |
| Sakellariou | 2017 | cross-sectional | Greece | AS | 57 | HC | 34 | 39.1±1.4/38.8±1.0 | 4 | PubMed | [268] |
| Torres | 2019 | cross-sectional | Sweden | AS | 204 | HC | 80 | 49(41–62)/48.5 (41–57) | 5 | PubMed | [269] |
| Seo | 2005 | cross-sectional | Korea | AS | 157 | HC | 140 | 29.8/28.8 | 3 | PubMed | [270] |
| Poddubnyy | 2013 | Cohort | Hindenburgda | axSpA | 172 | - | - | 37.0±10.6 | 5 | PubMed, Embase | [271] |
| Braun | 2016 | Cohort | Germany | AS | 140 | - | - | - | 6 | PubMed, Embase | [272] |
| Rademacher | 2019 | Cohort | Germany | SpA with radiographic progression | 28 | SpA without radiographic progression | 89 | 44.6 (7.6)/42.4 (10.8) | 4 | PubMed | [234] |
| Appel | 2008 | Cohort | Germany | SpA | 71 | - | - | 37.75±10.67 | 5 | PubMed | [273] |
| Visvanathan | 2008 | cross-sectional | USA | AS with infliximab | 201 | AS with placebo | 78 | - | 5 | PubMed | [274] |
| Pedersen | 2010 | Cohort | Denmark | SpA | 42 |  |  |  | 5 | PubMed | [275] |
| Tošovský | 2014 | cross-sectional | Czech | AS with TNF-α | 21 | AS without TNF-α | 34 | 42(42.8)/47.5 (49.4) | 3 | PubMed | [276] |

**Question 10**: **Calprotectin**

| Author | Publish year | Design | Country | Patient diagnosis | Patient number | Control type | Control number | Age (year) Patient/ control | NOS score | Database | Reference number |
| --- | --- | --- | --- | --- | --- | --- | --- | --- | --- | --- | --- |
| Ma | 2020 | Meta-analysis | China | SpA | - | HC | - | - | 5 | Pubmed, Embase | [277] |
| Ercalik | 2021 | cross-sectional | Turkey | AS | 97 | non-inflammatory rheumatic diseases | 49 | 43.90 ± 13.42 | 3 | Pubmed, Embase | [278] |
| Genre | 2018 | case-control | Genre | AS | 163 | HC | 63 | 43.7±11.6/ 50.9±15.3 | 6 | Pubmed, Embase | [279] |
| Huang | 2017 | case-control | China | AS  nr-axSpA | 53  59 | HC | 47 | 32.3 ± 8.21/ 34.4 ± 7.79 | 5 | Pubmed, Embase | [280] |
| Klingberg | 2012 | case-control | Sweden | AS | 205 | HC | 80 | 50 (17-78) | 6 | Pubmed, Embase | [281] |
| Oktayoglu | 2014 | case-control | Turkey | AS | 31 | HC | 45 | 29.7 ± 8.1/ 31.7 ± 7.8) | 6 | Pubmed, Embase | [282] |
| Olofsson | 2019 | case-control | Sweden | AS  nr-axSpA | 33  78 | HC | 35 | 56 ± 14/ 46 ± 11 | 6 | Pubmed, Embase | [283] |

**Question 11: non-coding RNA**

| Author | Year | Design | Country | Patient diagnosis | Patient number | Controls type | Patient number | RNA type | NOS score | Database | Reference number |
| --- | --- | --- | --- | --- | --- | --- | --- | --- | --- | --- | --- |
| Qian | 2016 | Case-control | China | AS | 80 | HC | 78 | miR-155 | 5 | Pubmed;  Embase | [284] |
| Wang | 2017 | Case-control | China | AS | 40 | HC | 40 | miR-31  miR-155  miR-16 | 5 | Pubmed;  Embase | [285] |
| Wang | 2017 | Case-control | China | AS | 41 | HC | 36 | miRNA-199a-5p | 4 | Pubmed;  Embase | [286] |
| Wei | 2017 | Case-control | China | AS | 45 | HC | 30 | miR-146a | 4 | Pubmed;  Embase | [287] |
| Yildirim | 2021 | Case-control | Turkey | r-axSpA | 15 | HC | 9 | miR-145-5p | 4 | Pubmed;  Embase | [288] |
| Zhang | 2018 | Case-control | China | AS | 50 | HC | 25 | miRNA16a | 4 | Pubmed;  Embase | [289] |
| Zou | 2019 | Case-control | China | AS | 69 | HC | 69 | miR-21 | 6 | Pubmed;  Embase | [290] |
| Guo | 2018 | Case-control | China | AS | 219 | HC | 113 | miR-132 | 6 | Pubmed;  Embase | [291] |
| Ciechomska | 2018 | Case-control | Poland | AS | 13 | HC | 12 | miR-5196 | 4 | Pubmed;  Embase | [292] |
| Fotoh | 2020 | cross-sectional | Egypt | AS | 55 | HC | 55 | iRNA-451a  miRNA-125a | 5 | Pubmed;  Embase | [293] |
| Lv | 2014 | Case-control | China | AS | 40 | HC | 50 | hsa-miR-126-3p  hsa-miR-29a | 4 | Pubmed;  Embase | [294] |
| RAJZLEROVÁ | 2020 | Cross-sectional | Czech | AS | 19 | / | / | miR-145 | 3 | Pubmed;  Embase | [295] |
| Liu | 2020 | Case-control | China | AS | 32 | HC | 24 | miR-214 | 5 | Pubmed;  Embase | [296] |
| Ni | 2020 | Case-control | China | AS | 150 | HC | 150 | miR-495 | 6 | Pubmed;  Embase | [297] |
| Perez-Sanchez | 2017 | Case-control | Spain | AS | 53 | HC | 57 | miR-146a-5p  miR-125a-5p  miR-151a–3p  miR-22–3p  miR-451a | 5 | Pubmed;  Embase | [298] |
| PrajzlerovaÂ | 2017 | Case-control | Switzerland | axSpA | 68 | HC | 29 | miR-29a-3p  miR-146a-5p  miR-222-3p  miR-625-3p | 6 | Pubmed;  Embase | [299] |
| Reyes-Loyola | 2019 | cross-sectional | Mexico | AS | 15 | HC | 13 | miR-16 | 5 | Pubmed;  Embase | [300] |
| Tan | 2021 | Case-control | China | AS | 44 | HC | 56 | miR-146a  miR-125a-5p  miR-125b-5p  miR-499a  miR-155a | 4 | Pubmed;  Embase | [301] |
| Li | 2017 | Case-control | China | axSpA | 59 | HC | 39 | miR-27a | 5 | Pubmed;  Embase | [302] |
| Huang | 2014 | Case-control | China | AS | 122 | HC | 122 | miR-21 | 6 | Pubmed;  Embase | [303] |
| Lan | 2018 | Case-control | China | AS | 82 | HC | 32 | TUG1 | 4 | Pubmed;  Embase | [304] |
| Zhong | 2019 | Case-control | China | AS  LBP | 80  22 | HC | 20 | LINC00311 | 4 | Pubmed;  Embase | [305] |
| Han | 2022 | Case-control | China | AS | 60 | HC | 60 | lncRNA‐NEF | 4 | Pubmed;  Embase | [306] |
| Wang | 2022 | Case-control | China | AS | 20 | HC | 20 | linc00304  linc00926  MIAT | 5 | Pubmed;  Embase | [307] |
| Wang | 2021 | Case-control | China | AS | 9 | HC | 12 | circPTPN22  hsa_circ_0005918 | 3 | Pubmed;  Embase | [308] |
| Zou | 2023 | Case-control | China | AS | 3 | FNF | 3 | circRNA | 3 | Pubmed;  Embase | [309] |
| Tang | 2021 | Case-control | China | AS | 60 | HC | 30 | circRNA | 5 | Pubmed;  Embase | [310] |
| Luo | 2020 | Case-control | China | axSpA  SLE | 46  46 | HC | 25 | hsa_circ_0079787 | 4 | Pubmed;  Embase | [311] |

**Question 12: Inflammatory cytokines including IL-6, IL-17 and TNF-α**

| Author | Publish year | Design | Country | Patient diagnosis | Patient number | Control type | Control number | Age (year) Patient/ control | NOS score | Database | Reference number |
| --- | --- | --- | --- | --- | --- | --- | --- | --- | --- | --- | --- |
| Romero-Sanchez | 2011 | cross-sectional | China | AS | 50 | mechanical LBP | 27 | - | 3 | Pubmed, Embase | [312] |
| Gratacós | 1999 | Cohort | Spain | active AS | 14 | inactive AS | 20 | 33±1/ 31±1 | 5 | Pubmed | [313] |
| Bal | 2007 | cross-sectional | Turkey | AS | 70 | HC | 39 | 38.79±11.45 | 5 | Pubmed | [314] |
| Elolemy | 2013 | cross-sectional | Egypt | AS | 25 | HC | 20 | 29.11±7.86/ 32.12±6.41 | 5 | Embase | [237] |
| Sharma | 2014 | cross-sectional | India | AS | 62 | HC | 60 | 32.9±8.33/ 33.0±5.2 | 5 | Embase | [315] |
| Przepiera-Będzak | 2015 | cross-sectional | Poland | SpA | 152 | HC | 29 | 43.3±13.2/ 48.2±13.5 | 4 | Pubmed | [316] |
| Li | 2016 | cross-sectional | China | AS | 30 | HC | 30 | - | 4 | Pubmed | [317] |
| Rabelo | 2018 | cross-sectional | Brazil | AS | 32 | HC | 32 | 46.9±10.7/ 46.5±9.7 |  | Embase | [318] |
| Park | 2007 | cross-sectional | Korea | AS | 42 | HC | 42 | 31.0±9.3/ 31.3±9.3 | 5 | Pubmed | [238] |
| Park | 2009 | cross-sectional | Korea | AS | 20 | HC | 20 | 30.2±7.0/ 30.5±6.7 | 4 | Pubmed | [243] |
| Mattey | 2012 | Cohort | United Kingdom | AS | 157 | HC | - | - | 4 | Pubmed,  Embase | [319] |
| Taylan | 2012 | cross-sectional | Turkey | active AS | 28 | inactive AS | 28 | - | 3 | Pubmed,  Embase | [320] |
| Taylan | 2012 | cross-sectional | Turkey | AS | 127 | HC | 38 | - | 5 | Pubmed | [321] |
| Sveaas | 2015 | cross-sectional | Norway | AS | 143 | HC | 125 | 49.3±11.0/ 53.2±11.3 | 3 | Pubmed,  Embase | [322] |
| Liu | 2015 | cross-sectional | China | AS | 60 | HC | 60 | - | 5 | Pubmed | [323] |
| He | 2017 | Cohort | United States | AS | 24 | - | - | - | 5 | Pubmed,  Embase | [324] |
| Korkosz | 2018 | cross-sectional | Poland | SpA | 27 | HC | 23 | 32.9±7.7/ 35.1±5.4 | 4 | Pubmed,  Embase | [325] |
| Falkenbach | 1998 | Cohort | Austria | AS | 261 | - | - | - | 3 | Pubmed,  Embase | [326] |
| Falkenbach | 2000 | Cohort | Austria | AS | 128 | - | - | - | 5 | Pubmed | [327] |
| Korczowska | 2011 | cross-sectional | Poland | AS | 66 | HC | 63 | 51.6±13.2/ 17.4±12.1 | 5 | Pubmed | [328] |
| Gonzalez-Lopez | 2017 | cross-sectional | Mexico | AS | 48 | HC | 41 | 44.3±11.4/ 46.2±10.7 | 4 | Pubmed | [240] |
| Dong | 2019 | Cohort | China | AS | 60 | HC | 24 | - | 6 | Pubmed | [218] |
| Brandt | 2000 | Cohort | Germany | AS | 11 |  |  | - | 3 | Pubmed,  Embase | [329] |
| Tarner | 2009 | cross-sectional | Germany | AS | 12 | HC | 12 | - | 4 | Pubmed | [330] |
| Visvanathan | 2008 | Cohort | United States | AS | 279 | - | - | 39.6±10.6)/ 40.3±9.4 | 6 | Embase,  Medline | [274] |
| Pedersen | 2010 | Cohort | Denmark | SpA | 49 | - | - | - | 5 | Pubmed | [275] |
| Pedersen | 2011 | Cohort | Denmark | SpA | 60 | - | - | - | 6 | Pubmed | [331] |
| Capkin | 2012 | cross-sectional | Turkey | AS | 108 | HC | 65 | 36.4±11.2/ 38.2±13.0 | 4 | Pubmed | [332] |
| Limón-Camacho | 2012 | cross-sectional | Mexico | AS | 46 | HC | 25 | 32±13/ 32±8 | 3 | Pubmed,  Embase | [333] |
| Wagner | 2012 | Cohort | United States | AS | 100 | - | - | - | 6 | Embase | [261] |
| Schulz | 2014 | Cohort | Germany | AS | 50 | - | - | - | 4 | Pubmed | [334] |
| Inman | 2016 | Cohort | Canada | AS | 278 | - | - | - | 6 | Pubmed,  Embase | [260] |
| Levitova | 2016 | Cohort | Czech Republic | AS | 46 | - | - | - | 5 | Pubmed,  Embase | [335] |
| Zhang | 2021 | Cohort | China | active AS | 103 | - | - | - | 6 | Pubmed | [336] |
| Eggert | 2007 | Cohort | Germany | AS | 17 | HC | 24 | - | 4 | Pubmed | [337] |
| Yang | 2018 | cross-sectional | China | AS | 486 | HC | 480 | 32.75±11.18/ 37.84±14.31 | 4 | Embase | [338] |
| Aghaei | 2020 | cross-sectional | Iran | AS | 455 | HC | 450 | 38.10±10.46/ 36.60±8.25 | 5 | Pubmed | [339] |
| Chen | 2012 | cross-sectional | China | AS | 49 | HC | 25 | 39.0±12.3 | 4 | Pubmeb | [340] |
| Londono | 2012 | cross-sectional | Colombia | SpA | 62 | HC | 46 | - | 4 | Pubmed,  Embase | [235] |
| Mei | 2011 | cross-sectional | China | AS | 50 | HC | 45 | 28.1±8.9/ 25.3±6.7 | 4 | Pubmed | [341] |
| Wendling | 2007 | cross-sectional | France | AS | 28 | HC | - | - | 3 | Pubmed | [342] |
| Wielińska | 2021 | cross-sectional | Poland | AS | 138 | HC | 190 | - | 4 | Pubmed | [343] |
| Tan | 2022 | cross-sectional | China | AS | 60 | HC | 60 | - | 5 | Embase | [344] |
| Jansen | 2015 | cross-sectional | Netherlands | AS | 31 | HC | 21 | - | 4 | Pubmed | [345] |
| Xueyi | 2013 | Cohort | China | AS | 222 | - | - | - | 6 | Pubmed, Embase | [346] |
| Perpétuo | 2015 | Cohort | Finland | AS | 13 | - | - | - | 3 | Pubmed | [347] |
| Milanez | 2016 | cross-sectional | Brazil | AS | 86 | HC | 47 | 38.0±11.1/ 49.5±10.5 | 4 | Pubmed | [348] |
| Du | 2022 | cross-sectional | China | AS | 80 | HC | 46 | 32.6±10.8/ 32.7±10.9 | 5 | Pubmed | [349] |
| Wen | 2017 | cross-sectional | China | AS | 60 | HC | 30 | 21.0±4.1/ 20.0±5.4 | 4 | Pubmed | [350] |
| Dong | 2019 | Cohort | China | AS | 60 | HC | 21 | 29.5±6.96/ 31.80±10.53 | 5 | Pubmed | [351] |

**Question 13: Peripheral lymphocyte subsets**

| Author | Publish year | Design | Country | Patient diagnosis | Patient number | Control type | Control number | Age (year) AS/HC | NOS score | Database | Reference number |
| --- | --- | --- | --- | --- | --- | --- | --- | --- | --- | --- | --- |
| An | 2019 | cross-sectional | China | AS | 73 | HC | 85 | nr | 5 | Medline; PubMed | [352] |
| Appel | 2011 | cross-sectional | Germany | AS | 19 | HC | 20 | 40.9±13.8/nr | 4 | Medline; PubMed | [353] |
| Bautista-Caro | 2014 | cross-sectional | Norway | AS | 25 | HC | 50 | 56±14.8/nr | 5 | Medline; PubMed | [354] |
| Bidad | 2013 | cross-sectional | Iran | AS | 18 | HC | 18 | 34±2/33±1 | 6 | Medline; PubMed | [355] |
| Brand | 1997 | cross-sectional | Germany | AS | 21 | HC | 29 | 42±14/47±16 | 5 | Medline; PubMed | [356] |
| Cai | 2013 | cross-sectional | China | AS | 40 | HC | 20 | 29±9.4/28.4±10.3 | 7 | CNKI | [357] |
| Cai | 2005 | cross-sectional | China | AS | 30 | HC | 20 | nr | 4 | CNKI | [358] |
| Cao | 2004 | cross-sectional | Sweden | AS | 10 | HC | 29 | nr | 4 | PubMed | [359] |
| Chen | 2013 | cross-sectional | China | AS | 61 | HC | 36 | 25±8.2/25±7 | 7 | CNKI | [360] |
| Chen | 2011 | cross-sectional | China | AS | 23 | HC | 25 | nr | 6 | Medline; PubMed | [361] |
| Cheng | 2007 | cross-sectional | China | AS | 25 | HC | 21 | 2B±9/27±6 | 6 | CNKI | [362] |
| Dejaco | 2010 | cross-sectional | Austria | AS | 22 | HC | 17 | 40.9±12,7/40.3± 23.4 | 3 | Medline; PubMed | [363] |
| Deng | 2019 | cross-sectional | China | AS | 49 | HC | 100 | 28.31±6.72/27.38±6.39 | 7 | CNKI | [364] |
| Deng | 2018 | cross-sectional | China | AS | 91 | HC | 50 | nr | 6 | CNKI | [365] |
| Dong | 2006 | cross-sectional | China | AS | 30 | HC | 30 | nr | 5 | CNKI | [366] |
| Duan | 2017 | cross-sectional | China | AS | 21 | HC | 16 | 37±9.8/34.6±10.1 | 6 | Medline; PubMe | [367] |
| Dulic | 2017 | cross-sectional | Hungary | AS | 7 | HC | 10 | nr | 6 | Medline; PubMe | [368] |
| Fattahi | 2018 | cohort | Iran | AS | 30 | HC | 15 | 31.4±9.1/32.1±8.2 | 6 | Medline: PubMe | [369] |
| Forger | 2009 | cross-sectional | Switzerland | AS | 15 | HC | 18 | nr | 5 | PubMed | [370] |
| Gao | 2012 | cross-sectional | China | AS | 40 | HC | 37 | 29.1±8.6/26.7±6.9 | 6 | CNKI | [371] |
| Guo | 2012 | cross-sectional | China | AS | 98 | HC | 76 | nr | 5 | CNKI | [372] |
| Hajialilo | 2019 | cross-sectional | Iran | AS | 24 | HC | 35 | nr | 6 | Cochrane, Medline,  PubMed | [373] |
| Han | 2006 | cross-sectional | China | AS | 69 | HC | 50 | nr | 5 | CNKI | [374] |
| He | 2012 | cross-sectional | China | AS | 32 | HC | 50 | nr | 4 | CNKI | [375] |
| Hu | 2019 | cross-sectional | China | AS | 60 | HC | 40 | nr | 5 | CNKI | [376] |
| Hu | 2013 | cross-sectional | China | AS | 32 | HC | 30 | 34±3.89/36±3.76 | 4 | CNKI | [377] |
| Huang | 2009 | cross-sectional | China | AS | 20 | HC | 9 | nr | 4 | CNKI | [378] |
| Huang | 1990 | cross-sectional | China | AS | 9 | HC | 9 | nr | 4 | CNKI | [379] |
| Ji | 2014 | cohort | China | AS | 20 | HC | 20 | nr | 7 | Medline; PubMed | [380] |
| Kenna | 2012 | cross-sectional | Austra]ia | AS | 17 | HC | 20 | 39.47±13.6/nr | 6 | Medline: PubMed | [381] |
| Kim | 2012 | cross-sectional | South Korea | AS | 49 | HC | 53 | 36.4±10.8/34.9±9 | 6 | Medline; PubMed | [382] |
| Klasen | 2019 | cross-sectional | Germany | AS | 14 | HC | 5 | 42.7±3.15/nr | 5 | Medline; PubMed | [383] |
| Li | 2019 | cross-sectional | China | AS | 64 | HC | 60 | 33.26±5.74/35.84±6.19 | 7 | CNKI | [384] |
| Li | 2013 | cohort | China | AS | 222 | HC | 68 | 33.6±8/34.1±10.6 | 6 | Medline; PubMed | [385] |
| Li | 2009 | cross-sectional | China | AS | 30 | HC | 10 | nr | 5 | CNKI | [386] |
| Li | 2008 | cross-sectional | China | AS | 50 | HC | 21 | 25±8/25±5 | 6 | CNKI | [387] |
| Liao | 2015 | cohort | China | AS | 69 | HC | 30 | 39.6±12.7/44.3± 10.5 | 7 | Medline; PubMed | [388] |
| Limon-Camacho | 2012 | cross-sectional | Mexico | AS | 39 | HC | 25 | 32±13/32±8 | 4 | PubMed | [389] |
| Lin | 2008 | cohort | China | AS | 66 | HC | 30 | 29.7±9.6/26.7±6.7 | 6 | CNKI | [390] |
| Lin | 2009 | cohort | China | AS | 66 | HC | 30 | 29.7±9.6/26.7±6.7 | 6 | Cochrane; Medline; PubMed | [391] |
| Liu | 2017 | cross-sectional | China | AS | 38 | HC | 38 | 39.3±3.4/40.4± 3.9 | 6 | CNKI | [392] |
| Liu | 2016 | cross-sectional | China | AS | 60 | HC | 20 | 35±10.7/41.9± 11.7 | 5 | CNKI | [393] |
| Liu | 2012 | cross-sectional | China | AS | 60 | HC | 30 | 31.5±9.1/nr | 6 | CNKI | [394] |
| Liu | 2010 | cross-sectional | China | AS | 30 | HC | 20 | 26±3.69/25.15±3.79 | 6 | CNKI | [395] |
| Long | 2018 | cross-sectional | China | AS | 65 | HC | 20 | 27.8±8.5/31.4±7.4 | 6 | Medline; PubMed | [396] |
| Ma | 2011 | cross-sectional | China | AS | 36 | HC | 32 | 23.1±4.8/25.8±3.6 | 5 | CNKI | [397] |
| Ma | 2011 | cross-sectional | China | AS | 43 | HC | 20 | nr | 4 | CNKI | [398] |
| Ma | 2004 | cross-sectional | China | AS | 25 | HC | 30 | nr | 5 | CNKI | [399] |
| Meng | 2015 | cross-sectional | China | AS | 42 | HC | 20 | 32.4±9.3/29.5±8.4 | 6 | CNKI | [400] |
| Mo | 2019 | cross-sectional | China | AS | 30 | HC | 23 | 40.7±3.18/45.71±2.6 | 7 | CNKI | [401] |
| Pishgahi | 2020 | cross-sectional | Iran | AS | 31 | HC | 35 | nr/41.89±11.29 | 5 | Medline; PubMed | [241] |
| Shan | 2015 | cross-sectional | China | AS | 20 | HC | 10 | nr | 6 | Medline: PubMed | [402] |
| Shen | 2009 | cross-sectional | China | AS | 10 | HC | 16 | 46.05±11.51/nr | 3 | Medline; PubMed | [403] |
| Suen | 2008 | cross-sectional | China | AS | 23 | HC | 26 | 43±12/37±12 | 4 | Medline; PubMed | [404] |
| Szalay | 2012 | cohort | Hungary | AS | 13 | HC | 9 | 43.7± 9.2/nr | 4 | Medline; PubMed | [405] |
| Szanto | 2008 | cross-sectional | Hungary | AS | 42 | HC | 52 | nr | 5 | Medline; PubMed | [406] |
| Thoen | 1987 | cross-sectional | Norway | AS | 31 | HC | 15 | 32± 1.8/nr | 3 | Medline; PubMed | [407] |
| Toussirot | 2009 | cross-sectional | France | AS | 32 | HC | 15 | 42.9±1.1/44.4±0.8 | 4 | PubMed | [408] |
| Wang | 2020 | cross-sectional | China | AS | 90 | HC | 90 | 43.27±8.19/43.55±8.6 | 5 | CNKI | [409] |
| Wang | 2018 | cross-sectional | China | AS | 30 | HC | 30 | 31.2±4.1/nr | 6 | CNKI | [410] |
| Wang | 2018 | cross-sectional | China | AS | 26 | HC | 26 | 33.5±8.4/31.5±10.2 | 4 | Medline; PubMed | [411] |
| Wang | 2016 | Cross-sectional | China | AS | 50 | HC | 50 | 28.53 ± 8.15/27.93 ± 8.52 | 7 | Medline; PubMed | [412] |
| Wang | 2015 | cross-sectional | China | AS | 78 | HC | 30 | 26±7,8/25±8 | 6 | CNKI | [413] |
| Wang | 2015 | cross-sectional | China | AS | 45 | HC | 20 | nr/55.05±6.42 | 5 | Medline; PubMed | [414] |
| Wang | 2012 | cross-sectional | China | AS | 60 | HC | 44 | nr | 6 | CNKI | [415] |
| Wang | 2008 | cross-sectional | China | AS | 30 | HC | 20 | 26 +3.69/25.15±3.79 | 6 | CNKI | [416] |
| Wei | 2017 | cross-sectional | China | AS | 131 | HC | 127 | 27±8/26±9 | 6 | CNKI | [417] |
| Wu | 2014 | cross-sectional | China | AS | 60 | HC | 60 | 26.9±7.8/24.3±5.6 | 6 | CNKI | [418] |
| Wu | 2011 | cross-sectional | China | AS | 51 | HC | 49 | nr | 6 | Medline; PubMed | [419] |
| Wu | 2011 | cross-sectional | China | AS | 24 | HC | 30 | 35±14/33±12 | 6 | CNKI | [420] |
| Xu | 2019 | cross-sectional | China | AS | 18 | HC | 9 | 39.4±2.3/42.6±4.3 | 6 | Medline; PubMed | [421] |
| Xu | 2018 | cross-sectional | China | AS | 69 | HC | 22 | nr | 6 | CNKI | [422] |
| Xu | 2013 | cross-sectional | China | AS | 24 | HC | 22 | 24.3±8.5/27.9±8.6 | 6 | CNKI | [423] |
| Xu | 2011 | cross-sectional | China | AS | 78 | HC | 50 | nr | 5 | CNKI | [424] |
| Xue | 2015 | cross-sectional | China | AS | 38 | HC | 30 | 29.93 ±9,82/30.58±8.39 | 6 | CNKI | [425] |
| Xue | 2008 | cross-sectional | China | AS | 89 | HC | 42 | nr | 5 | CNKI | [426] |
| Yang | 2020 | cohort | China | AS | 67 | HC | 50 | nr | 6 | Medline; PubMed | [427] |
| Yang | 2018 | cross-sectional | China | AS | 30 | HC | 30 | 29.3±5.9/31.1±6.7 | 6 | CNKI | [428] |
| Yang | 2017 | cross-sectional | China | AS | 40 | HC | 40 | 32.53±9.76/33.7±10.06 | 6 | CNKI | [429] |
| Yang | 2016 | cross-sectional | China | AS | 38 | HC | 31 | 28.9±10.8/29.1± 8.1 | 7 | CNKI | [430] |
| Yang | 2007 | cross-sectional | China | AS | 60 | HC | 30 | nr | 6 | CNKI | [431] |
| Ye | 2013 | cross-sectional | China | AS | 21 | HC | 27 | 36.6±10.2/37.9± 9.1 | 3 | Medline: PubMed | [432] |
| Zhang | 2019 | cross-sectional | China | AS | 60 | HC | 30 | 43±11/32±12 | 5 | CNKI | [433] |
| Zhang | 2019 | cross-sectional | China | AS | 39 | HC | 41 | 28.87±8.31/27.05±6.63 | 6 | CNKI | [434] |
| Zhang | 2014 | cross-sectional | China | AS | 60 | HC | 60 | 39±3.2/39.2±3.1 | 6 | CNKI | [435] |
| Zhang | 2014 | cross-sectional | China | AS | 10 | HC | 10 | nr | 4 | CNKI | [436] |
| Zhang | 2012 | cross-sectional | China | AS | 32 | HC | 20 | 36.6±10.2/37.9±9.1 | 6 | Medline; PubMed | [437] |
| Zhang | 2008 | cross-sectional | China | AS | 78 | HC | 50 | 26.1±6.8/25.5±3.8 | 7 | CNKI | [438] |
| Zhao | 2013 | cross-sectional | China | AS | 21 | HC | 20 | nr/26±8 | 5 | CNKI | [439] |
| Zhao | 2011 | Cross-sectional | China | AS | 14 | HC | 18 | 26.4 ± 6.1/28.2 ± 9.4 | 5 | Medline; PubMed | [440] |
| Zhao | 2009 | cross-sectional | China | AS | 30 | HC | 30 | nr | 5 | CNKI | [441] |
| Zhong | 2014 | cross-sectional | China | AS | 78 | HC | 30 | nr | 6 | CNKI | [442] |
| Zhu | 2017 | cross-sectional | China | AS | 42 | HC | 42 | nr | 3 | CNKI | [443] |
| Zhu | 2016 | cross-sectional | China | AS | 30 | HC | 30 | nr | 5 | CNKI | [444] |
| Zhu | 2000 | cross-sectional | China | AS | 14 | HC | 7 | nr | 4 | CNKI | [445] |

**Question 14: Bone turnover markers, including β-CTX and PINP**

| Author | Publish year | Design | Country | Patient diagnosis | Patient number | Control type | Control number | Age (year) Patient/control | NOS score | Database | Reference number |
| --- | --- | --- | --- | --- | --- | --- | --- | --- | --- | --- | --- |
| Marhoffer | 1995 | cross-sectional | Germany | AS | 62 | HC | 50 | - | 4 | Embase, PubMed | [446] |
| Toussirot | 1999 | cross-sectional | France | AS | 32 | HC | 25 | 36.7±13.9/35.04±9.5 | 6 | Embase, PubMed | [447] |
| Yilmaz | 2000 | cross-sectional | Turkey | AS | 44 | HC | 41 | 47±2.7/44±3.1 | 7 | PubMed | [448] |
| Lange | 2005 | cross-sectional | Germany | AS | 58 | HC | 58 | 38.4(17-76)/- | 7 | Embase, PubMed | [449] |
| Muntean | 2011 | cross-sectional | Romania | AS | 44 | HC | 39 | 41±10.2)/39.5±9.3) | 5 | Embase, PubMed | [450] |
| Taylan | 2012 | cross-sectional | Turkey | AS | 55 | HC | 33 | 36 (19–61)/39 (23–48) | 6 | Embase, PubMed | [451] |
| Korkosz | 2013 | cross-sectional | Poland | AS | 78 | HC | 23 | 37.8±11.6/ 32.0±6.6/ 32.3±7.5 | 4 | PubMed | [452] |
| Almodóvar | 2014 | Cohort | Spain | SpA | 60 | - | - | 33.5 (27.8–36.9) | 3 | Embase, PubMed | [453] |
| Klingberg | 2014 | cross-sectional | Sweden | AS | 204 | HC | 80 | 49 (17, 78)/48 (19, 71) | 6 | Embase, PubMed | [454] |
| Nocturne | 2015 | cross-sectional | France | SpA | 479 | HC | 80 | -/32 ±9.1 | 4 | PubMed | [455] |
| Wang | 2015 | cross-sectional | China | AS | 102 | HC | 102 | 35.5±2.9/35.7±2.8 | 5 | Embase, PubMed | [456] |
| Zhang | 2015 | cross-sectional | China | AS | 150 | HC | 168 | 29.19 ± 8.94/31.83 ± 9.97 | 5 | Embase, PubMed | [457] |
| Guła | 2018 | cross-sectional | Poland | axSpA | 40 | perSpA | 23 | - | 3 | PubMed | [458] |
| Descamps | 2021 | Cohort | Belgium | SpA | 708 | - | - | - | 3 | PubMed | [459] |
| Speden | 2002 | cross-sectional | United Kingdom | AS | 40 | HC | 74 | 41.6±10.1/44.6±12.1 | 4 | Embase, PubMed | [460] |
| Torres | 2019 | cross-sectional | Sweden | AS | 204 | HC | 80 | 49(41–62)/ 48.5 (41–57) | 5 | PubMed | [269] |
| Franck | 2004 | cross-sectional | Germany | AS | 264 | HC | 240 | 50.4±10.4/50.4±16.4 | 5 | Embase, PubMed | [461] |
| Grisar | 2002 | cross-sectional | Australia | AS | 30 | HC | 41 | 44.2±12.7/41.7 ± 11.1 | 5 | Embase, PubMed | [462] |
| Acebes | 1999 | cross-sectional | Spain | AS | 18 | HC | 18 | 44.7(21–75)/- | 4 | PubMed | [463] |
| Huang | 2016 | cross-sectional | China | AS | 43 | HC | 42 | 36.8±9.3/36.1±9.7 | 5 | Embase | [464] |
| Borman | 2001 | cross-sectional | Turkey | AS | 32 | HC | 30 | 39.1±11.4/- | 4 | Embase | [210] |
| Park | 2008 | cross-sectional | South Korea | AS | 35 | HC | 70 | 28.8±8.6/29.2±8.8 | 6 | Embase, PubMed | [465] |
| Vosse | 2008 | Cohort | Vosse, D. | AS | 83 | - | - | 50±12 | 5 | PubMed | [466] |
| Arends | 2014 | cross-sectional | France | AS with bridging | 52 | AS without bridging | 99 | - | 3 | Embase, PubMed | [467] |
| Gamez-Nava | 2016 | cross-sectional | Mexico | AS | 78 | HC | 58 | - | 4 | Embase, PubMed | [468] |
| Sun | 2019 | cross-sectional | China | AS | 88 | HC | 26 | - | 5 | PubMed | [469] |
| Iaremenko | 2020 | cross-sectional | Ukraine | SpA | 102 | HC | 15 | 38.1±11.2 | 5 | Embase, PubMed | [470] |
| Liu | 2021 | cross-sectional | China | RHI-AS | 177 | WORHI-AS | 162 | 27(23, 37)/37(28, 48) | 4 | Embase | [471] |
| Kim | 2012 | cross-sectional | Korea | AS | 72 | HC | 20 | 35.1±1.1/29.9±1.0 | 3 | PubMed | [251] |
| Briot | 2005 | Cohort | France | SpA | 19 |  |  | 21-71 | 4 | PubMed | [472] |
| Appel | 2008 | Cohort | Germany | SpA | 71 | - | - | 37.75±10.67 | 5 | PubMed | [273] |
| Visvanathan | 2009 | Cohort | United States | AS | 279 | - | - | 39.6±10.6/40.3±9.4 | 6 | Embase, PubMed | [473] |
| Arends | 2012 | Cohort | Netherlands | AS | 111 | - | - | 42.2 ± 10.3 | 5 | Embase, PubMed | [474] |
| Kwon | 2012 | Cohort | South Korea | AS | 56 | HC | 40 | 34.6±9.0/34.6±9.0 | 4 | Embase, PubMed | [475] |
| Saad | 2012 | Cohort | England | AS | 30 | HC | - | - | 5 | PubMed | [476] |
| Korkosz | 2014 | Cohort | Poland | AS | 40 | - | - | - | 3 | PubMed | [224] |
| De Andrade | 2014 | cross-sectional | Brazil | SpA | 52 | HC | 26 | 42.09±11.95/37.35± 12.73 | 5 | Embase, PubMed | [477] |
| Li | 2015 | Cohort | China | AS | - | - | - | - | 4 | Embase, PubMed | [478] |
| Sharma | 2017 | Cohort | India | AS | 23 | - | - | - | 3 | Embase | [479] |
| Gulyás | 2020 | Cohort | Hungary | AS | 17 | - | - | 43.6 ± 12.4 | 4 | Embase, PubMed | [480] |
| Braun | 2021 | Cohort | England | AS | - | - | - | - | 5 | PubMed | [481] |
| Choi | 2008 | Cross-sectional | Korea | AS | 30 | HC | 23 | 33.4±12.7/- | 6 | Embase, PubMed | [482] |
| Li | 2014 | cross-sectional | China | AS | 120 | HC | 80 | 29 ± 8/30±10 | 4 | CNKI | [483] |
| Zou | 2013 | cross-sectional | China | AS | 40 | HC | 40 | 32±7/32±7 | 3 | CNKI | [484] |
| Liu | 2015 | cross-sectional | China | AS | 37 | HC | 40 | 31.4±11.6/31.3±10.9 | 3 | CNKI | [485] |
| Kang | 2016 | cross-sectional | China | AS | 52 | HC | 40 | 32.33±13.51/31.52±11.87 | 4 | CNKI | [486] |
| Wei | 2013 | cross-sectional | China | AS | 40 | HC | 40 | 33.67±6.88/30.95±6.04 | 4 | CNKI | [487] |
| Kong | 2005 | cross-sectional | China | AS | 97 | HC | 102 | 29.20±6.27/29.51±7.29 | 4 | CNKI | [488] |
| Kang | 2018 | cross-sectional | China | AS | 50 | HC | 50 | 27.1±7.8/26.1±6.4 | 4 | CNKI | [489] |
| Wang | 2010 | cross-sectional | China | AS | 30 | HC | 20 | 28.30±7.65/30.20±5.20 | 3 | CNKI | [490] |
| Kong | 2010 | cross-sectional | China | AS | 100 | HC | 100 | 31.04±8.72/30.42±8.27 | 4 | CNKI | [491] |
| Xie | 2018 | cross-sectional | China | AS | 60 | HC | 20 | 26±11/30±10 | 4 | CNKI | [492] |
| Chen | 2014 | cross-sectional | China | AS | 46 | HC | 25 | 32±9/33±4 | 5 | CNKI | [493] |
| Lu | 2012 | cross-sectional | China | AS | 189 | HC | 37 | 29.83±5.80 | 4 | CNKI | [494] |
| Yang | 2016 | cross-sectional | China | SpA | 77 | HC | 30 | 30.01±8.5/29.1±7.7 | 4 | CNKI | [495] |
| Fang | 2014 | cross-sectional | China | AS | 150 | HC | 50 | 46±19/- | 4 | CNKI | [496] |
| Pan | 2014 | Cross-sectional | China | AS | 120 | HC | 120 | 31.85±8.76/31.79±8.64 | 5 | CNKI | [497] |
| Xu | 2009 | Cross-sectional | China | AS | 24 | HC | 8 | 29.5±12.1/31.4±4.9 | 4 | CNKI | [498] |
| Tan | 2017 | Cross-sectional | China | AS | 94 | HC | 80 | 35.43(20-68)/38.47(21-63) | 5 | CNKI | [499] |

**Question 15**: **C1M, C2M, C3M, C6M and VICM**

| Author | Publish year | Design | Country | Patient diagnosis | Patient number | Control type | Control number | Age (year) Patient/control | NOS score | Database | Reference number |
| --- | --- | --- | --- | --- | --- | --- | --- | --- | --- | --- | --- |
| Bay-Jensson | 2011 | Case-control | Germany | AS | 40 | HC | 40 | 42.9 (29-63)/43 (18-66) | 6 | Embase, PubMed | [500] |
| Holm Nielsen | 2022 | Cohort | Denmark | axSpA | 55 | - | - | 40 (32-48) | 5 | Embase, PubMed | [501] |
| Husakova | 2017 | Cohort | Denmark | axSpA | 46 | - | - | 36.4 ±5.9 | 4 | Embase, PubMed | [502] |
| Port | 2022 | Case-control | Denmark | axSpA | 102 | HC;  Postpartum women;  Disc herniation | 92;  46;  25 | 30.9±6.41/ 34±6.42/ 32.6±3.25/ 35.2 ±5.70 | 6 | Embase, PubMed | [503] |
| Siebuhr | 2018 | Cohort | Netherlands | AS | 122 | - | - | 45 ±12 | 5 | Embase, PubMed | [504] |
| Siebhur | 2016 | Cohort | Denmark | AS | 22 | - | - | 42.4 (37.7-46.8) | 4 | Embase, PubMed | [505] |
| Gudmann | 2017 | Case-control | Denmark | axSpA | 110 | HC | 120 | 36.6 (35.3-38.0)/37.0 (35.6-38.5) | 4 | Embase, PubMed | [506] |
| Bay-Jensen | 2013 | Cohort | Denmark | AS | 201 | HC | 35 | 43.4 ±12.8 / 4.25 ±8.95 | 6 | Embase, PubMed | [507] |
| Siebhur | 2019 | Cross-sectional | Czech | axSpA | 193 | HC | 100 | 34.5 (32.3-36.8)/ 37.0 (35.4-39.6) | 4 | Embase, PubMed | [508] |
| Husakova | 2019 | Cross-sectional | Czech | axSpA | 193 | HC | 100 | 37.5(35.5-39.6)/38.1 (36.3-39.9) | 4 | Embase, PubMed | [509] |

**Question 16: Sclerostin**

| Author | Publish year | Design | Country | Patient diagnosis | Patient number | Control type | Control number | Age (year) Patient/control | NOS score | Database | Reference number |
| --- | --- | --- | --- | --- | --- | --- | --- | --- | --- | --- | --- |
| Aschermann | 2016 | cross-sectional | Germany | SpA | 31 | HC | 30 | 47.8±16.3/50.9±15.6 | 4 | PubMed | [510] |
| Saad | 2012 | Cohort | England | AS | 30 | HC | 30 | 35.7 ± 11.0 | 5 | PubMed | [476] |
| Taylan | 2012 | cross-sectional | Turkey | AS | 55 | HC | 33 | 36 (19–61)/39 (23–48) | 6 | Embase, PubMed | [451] |
| Korkosz | 2013 | cross-sectional | Poland | AS | 78 | HC | 23 | 37.8 (11.6)/32.0 (6.6)/32.3 (7.5) | 4 | PubMed | [452] |
| Klingberg | 2014 | cross-sectional | Sweden | AS | 204 | HC | 80 | 49 (17, 78)/48 (19, 71) | 6 | Embase, PubMed | [454] |
| Ustun | 2014 | cross-sectional | Turkey | AS | 44 | HC | 41 | 40.06±9.51/- | 4 | PubMed | [511] |
| Rossini | 2016 | cross-sectional | Italy | AS | 71 | HC | 71 | - | 5 | PubMed | [512] |
| Sakellariou | 2017 | cross-sectional | Greece | AS | 57 | HC | 34 | 39.1±1.4/38.8±1.0 | 4 | PubMed | [268] |
| Solmaz | 2018 | cross-sectional | Turkey | AS | 97 | HC | 48 | 38 (14.0)/41 (6.8) | 5 | Embase, PubMed | [513] |
| Iaremenko | 2020 | cross-sectional | Ukraine | SpA | 102 | HC | 15 | 38.1±11.2/- | 4 | PubMed | [470] |
| Appel | 2008 | cross-sectional | Germany | AS | 46 | HC | 50 | - | 4 | Pubmed | [514] |
| Tuylu | 2014 | cross-sectional | Turkey | AS | 94 | HC | 68 | 40.7±8.7/43.9±9.9 /44.2±10.6 | 4 | PubMed | [515] |
| Perrotta | 2018 | cross-sectional | Italy | AS | 40 | HC | 20 | 50 (40.5–56.75)/- | 4 | PubMed | [516] |
| Rademacher, J. | 2019 | Cohort | Germany | SpA with radiographic progression | 28 | SpA without radiographic progression | 89 | 44.6 (7.6)/42.4 (10.8) | 4 | PubMed | [234] |
| Sun | 2019 | cross-sectional | China | AS | 88 | HC | 26 | 36.5±13.5/- | 4 | PubMed | [469] |
| Rademacher | 2022 | Cohort | Germany | AS | 137 | - | - | 42±11 | 6 | PubMed | [255] |
| Pathan | 2012 | Cohort | UK | AS | 38 | - | - | - | 3 | PubMed | [517] |
| Atas | 2022 | Cohort | Turkey | axSpA | 30 | HC | 30 | 38.1±13.3/37.7±7.7 | 4 | PubMed | [518] |
| Guo | 2023 | Cohort | China | AS | 116 | - | - | - | 4 | PubMed | [519] |
| Özdemirel | 2023 | Cohort | Turkey | AS | 53 | HC | 50 | - | 4 | Embase | [520] |

**Question 17**: **DKK-1**

| Author | Publish year | Design | Country | Patient diagnosis | Patient number | Control type | Control number | Age (year) Patient/control | NOS score | Database | Reference number |
| --- | --- | --- | --- | --- | --- | --- | --- | --- | --- | --- | --- |
| Kwon | 2012 | cross-sectional | Korea | AS | 56 | HC | 40 | 34.6 ± 9.0 / 43.5 ± 8.6 | 3 | PubMed | [475] |
| Fassio | 2017 | cross-sectional | Italy | PSA | 28 | HC | 43 | 57 ± 10 / 61 ± 5.8 | 3 | Embase; PubMed | [521] |
| Iaremenko | 2017 | cross-sectional | Ukraine | SPA | 102 | HC | 15 | 38.1 ± 11.2 / - | 5 | Embase; PubMed | [470] |
| Rubio Vargas | 2017 | cross-sectional | Spain | Early axSpA | 68 | Established axSpA | 22 | 34.6 ±9.7 / 43.2 ± 11 | 6 | Embase; PubMed | [522] |
| Noctrune | 2015 | cross-sectional | France | axSpA | 486 | HC | 80 | 32.5±8.6 / 32±9.1 | 5 | Embase; PubMed | [455] |
| Özdemirel | 2023 | cross-sectional | Turkey | AS | 53 | HC | 50 | 38 (20-52) / 36 (18-55) | 6 | PubMed | [520] |
| Guo | 2023 | RCT | China | axSpA  before  COX-2  inhibitors | 116 | axSpA after COX-2 inhibitors | 116 | 29.98 ± 8.72 / 30.1 ± 7.67 | 7 | Embase; PubMed | [519] |
| Papagoras | 2022 | Case-control | Greece | r-axSpA | 12 | HC | 16 | 37 (22-52) / 37 (22-55) | 5 | Embase; PubMed | [523] |
| Atas | 2022 | Case-control | Turkey | axSpA | 30 | HC | 30 | 38.1 ± 13.3 / 37.7 ± 7.7 | 6 | Embase; PubMed | [518] |
| Wu | 2018 | Meta-analysis | China | AS | 1348 | HC | 909 | - | 5 | Embase; PubMed | [524] |
| Sakellariou | 2017 | Cross-sectional | Greece | AS | 57 | HC | 34 | 39.1 ± 1.4 / 38.8 ± 1.0 | 8 | Embase; PubMed | [268] |
| Niu | 2017 | Case-control | China | AS | 6 | HC | 9 | 54.0 ± 19.1 / 55.9 ± 15.9 | 7 | Embase; PubMed | [525] |
| Liao | 2017 | Case-control | China | AS | 72 | HC | 30 | 36.82 ± 11.67/- | 7 | PubMed | [526] |
| Rossini | 2016 | Case-control | Italy | AS | 71 | HC | 71 | 43 ± 12 / 47 ± 12 | 8 | PubMed | [512] |
| Huang | 2016 | Case-control | China | AS | 43 | HC | 42 | 36.8±9.3 / 36.1±9.7 | 7 | PubMed | [464] |
| Ustun | 2014 | Cross-sectional | Turkey | AS | 44 | HC | 41 | - | 8 | Embase; PubMed | [511] |
| Tuylu | 2014 | Case-control | Turkey | AS | 94 | HC | 68 | 43.9±9.9 / 44.2±10.6 | 7 | Embase; PubMed | [515] |
| Korkosz | 2013 | Case-control | Poland | AS | 78 | HC | 23 | 37.8 ± 11.6 / 32.3 ± 7.5 | 6 | Embase; PubMed | [452] |
| Kim | 2012 | Case-control | Korea | AS | 40 | HC | 53 | - | 7 | Embase; PubMed | [382] |
| Daoussiss | 2010 | Case control | Greece | AS | 45 | HC | 50 | 42.3 ± 8.7 / - | 6 | Embase; PubMed | [527] |

**Question 18: OPG/RANKL/RANK**

| Author | Publish year | Design | Country | Patient diagnosis | Patient number | Control type | Control number | Age (year) Patient/control | NOS score | Database | Reference number |
| --- | --- | --- | --- | --- | --- | --- | --- | --- | --- | --- | --- |
| Beyazal | 2016 | Case-control | Turkey | AS | 60 | HC | 50 | 40.9 ± 10.9 / 42.8 ± 7.9 | 7 | Embase, PubMed | [528] |
| Caparbo | 2018 | Case-control | Brazil | AS | 85 | HC | 59 | 42.6 ± 9.0 / 40.7 ± 10.0 | 4 | Embase, PubMed | [529] |
| Chen | 2010 | Case-control | China | AS | 42 | HC | 26 | 33.9 ± 12.14 | 5 | Embase, PubMed | [530] |
| Dhir | 2013 | Case-control | India | AS | 85 | HC | 20 | 33.0 ± 10.0 | 3 | Embase, PubMed | [531] |
| Franck | 2004 | Case-control | Germany | AS | 264 | HC | 240 | 50.4 ± 10.4 / 50.4 ± 14.6 | 4 | Embase, PubMed | [461] |
| Genre | 2014 | Cohort | Spain | AS | 30 | - | - | 50.47 ± 14.85 | 4 | Embase, PubMed | [532] |
| Genre | 2018 | Case-control | Spain | axSpA | 163 | HC | 63 | 43.7 ± 11.6 / 50.9 ± 15.3 | 5 | Embase, PubMed | [533] |
| Grisar | 2002 | Case-control | Austria | AS | 30 | HC | 41 | 44.2 ± 12.7 / 41.7 ± 11.1 | 6 | Embase, PubMed | [462] |
| Guo | 2023 | Cohort | China | axSpA | 116 | - | - | - | 4 | Embase, PubMed | [519] |
| Hou | 2018 | Case-control | China | AS | 40 | HC | 40 | - | 3 | Embase, PubMed | [534] |
| Jadon | 2017 | Case-control | UK | AS | 157 | HC | 50 | 54.7 (44.52-3.41) / 59.93 (51.44-67.56) | 3 | Embase, PubMed | [535] |
| Kim | 2006 | Case-control | South Korea | AS | 60 | HC | NA | 32.1 ± 1.2 | 4 | Embase, PubMed | [536] |
| Klingberg | 2014 | Case-control | Sweden | AS | 204 | HC | 80 | 49 (17-78) | 4 | Embase, PubMed | [454] |
| Korkosz | 2018 | Case-control | Poland | axSpA | 27 | HC | 23 | 32.9 ± 7.7 / 35.1 ± 5.4 | 3 | Embase, PubMed | [325] |
| Kwon | 2012 | Cohort | South Korea | AS | 56 | HC | 40 | 34.6 ± 9.0 / 43.5 ± 8.6 | 3 | Embase, PubMed | [475] |
| Liu | 2021 | Cohort | China | AS | 23 | HC | 15 | 32.44 ± 8.00 | 3 | Embase, PubMed | [537] |
| Mou | 2015 | Case-control | China | juvenile-onset AS | 68 | HC | 32 | 18.66 ± 5.33 | 5 | Embase, PubMed | [538] |
| Niu | 2017 | Case-control | China | AS | 6 | HC | 9 | 54.0 ± 19.1 / 55.9 ± 15.9 | 3 | Embase, PubMed | [525] |
| Stupphann | 2008 | Case-control | Austria | AS | 21 | HC | 15 | 51.3 ± 3.3 / 49 ± 4 | 3 | Embase, PubMed | [539] |
| Sveaas | 2015 | Case-control | Norway | AS | 143 | HC | 124 | 43.9 ± 11.0 / 53.2 ± 11.3 | 5 | Embase, PubMed | [322] |
| Taylan | 2012 | Cohort | Turkey | AS | 55 | HC | 33 | 36 (19-61) / 39 (23-48) | 6 | Embase, PubMed | [451] |
| Wang | 2019 | Case-control | China | AS | 22 | HC | 22 | 42.89 ± 12.81 | 4 | Embase, PubMed | [540] |
| Woo | 2007 | Cohort | South Korea | AS | 26 | - | - | 34.5 ± 7.5 | 5 | Embase, PubMed | [541] |

**Question 19**: **MMP3**

| Author | Publish year | Design | Country | Patient diagnosis | Patient number | Control type | Control number | Age (year) Patient/control | NOS score | Database | Reference number |
| --- | --- | --- | --- | --- | --- | --- | --- | --- | --- | --- | --- |
| Lorenzin | 2019 | cross-sectional | Italy | SpA | 75 | - | - | - | 4 | Pubmed, Embase | [542] |
| Torres | 2019 | case-control | Sweden | AS | 204 | HC | 80 | 49 (41-28.2)/ 48.5 (41-57) | 5 | Pubmed, Embase | [269] |
| Mou | 2015 | case-control | China | JAS | 56 | HC | 32 | 18.66±5.33/ - | 6 | Pubmed, Embase | [538] |
| Almodovar | 2014 | cross-sectional | Spain | early SpA | 60 | - | - | 32.4±6.7/ - | 5 | Pubmed, Embase | [453] |
| Ramonda | 2013 | cohort | Italy | PsA | 43 | - | - | 49.9±8.5/ - | 6 | Pubmed, Embase | [543] |
| Soliman | 2012 | case-control | Egypt | AS | 30 | HC | 10 | 29.1±9.8/ - | 6 | Pubmed, Embase | [544] |
| Mattey | 2012 | cross-sectional | UK | AS | 157 | - | - | 49.05 (38.3-57.7)/ - | 5 | Pubmed, Embase | [319] |
| Ardend | 2011 | cohort | Netherlands | AS | 92 | - | - | 41.2±9.9/ - | 6 | Pubmed, Embase | [545] |
| Wendling | 2008 | case-control | France | AS | 23 | HC | 21 | 39.9±2.1/ 41.2±2.7 | 5 | Pubmed, Embase | [546] |
| Appel | 2008 | cohort | Germany | AS | 71 | - | - | 37.75±10.67/ - | 6 | Pubmed | [273] |
| Maksymowych | 2008 | cohort | Canada | AS | 82 | - | - | 40.0±10.78/ - | 7 | Pubmed, Embase, Cochrane | [547] |
| Chen | 2006 | case-control | China | AS | 42 | HC | 20 | 36.6±12.0/ - | 5 | Pubmed, Embase | [548] |
| Yang | 2004 | case-control | China | AS | 41 | HC | 28 | 25±9.9/ range 15-49 | 5 | Pubmed, Embase | [549] |
| Kaaij | 2020 | cohort | Netherlands | pSpA | 20 | - | - | 45.8±13.1/ - | 5 | Pubmed, Embase | [550] |
| Turina | 2014 | case-control | Netherlands | SpA | 78 | HC | 20 | -/- | 4 | Pubmed, Embase, Cochrane | [551] |
| He | 2017 | case-control | China | AS | 24 | HC | 10 | 37.2±12.2/ 29.5±5.98 | 5 | Pubmed, Embase | [324] |
| Chandran | 2013 | cohort | Canada | PsA | 40 | - | - | 44.3±10.9/ - | 5 | Pubmed, Embase | [552] |
| Pedersen | 2011 | cohort | Denmark | SpA | 60 | HC | 333 | 40 (range 21-62)/ - | 5 | Pubmed, Embase | [331] |
| Woo | 2007 | cohort | Korea | AS | 26 | - | - | 34.5±7.5/ - | 5 | Pubmed, Embase | [541] |
| Maksymowych | 2007 | cohort | Canada | AS | 97 | - | - | 42.9±12.5/ - | 6 | Pubmed, Embase | [553] |

**Question 20**: **BMP2**

| Author | Publish year | Design | Country | Patient diagnosis | Patient number | Control type | Control number | Age (year) Patient/control | NOS score | Database | Reference number |
| --- | --- | --- | --- | --- | --- | --- | --- | --- | --- | --- | --- |
| Guo | 2023 | cohort | China | axSpA | 116 | - | - | 29.98±8.72/- | 6 | Pubmed | [519] |
| Ozdemirel | 2022 | case-control | Turkiye | AS | 100 | HC | 102 | median 40 (range 18-62)/ median 38 (range 18-55) | 6 | Pubmed, Embase | [554] |
| Liao | 2017 | case-control | China | AS | 72 | HC | 30 | 36.82±11.76/- | 6 | Pubmed, Embase | [526] |
| Tosovsky | 2014 | case-control | Czech | AS patients treated with TNFi | 21 | AS patients untreated with TNFi | 42 | median 42 (37, 46)/ median 47.5 (38, 57) | 5 | Pubmed, Embase | [276] |
| Park | 2008 | case-control | Korea | AS | 40 | HC | 40 | 31.4±10.1/31.9±8.8 | 5 | Pubmed, Embase | [555] |
| Ozdemirel | 2023 | case-control | Turkiye | AS | 53 | HC | 50 | median 38 (range 20-52) / median 36 (range 18-55) | 6 | Pubmed | [520] |
| Chen | 2010 | case-control | China | AS | 120 | HC | 40 | 35.63±10.67/34.8±9.4 | 6 | Pubmed, Embase | [556] |

**Question 21: TNC**

| Author | Publish year | Design | Country | Patient diagnosis | Patient number | Control type | Control number | Age (year) Patient/control | NOS score | Database | Reference number |
| --- | --- | --- | --- | --- | --- | --- | --- | --- | --- | --- | --- |
| Bubová | 2020 | case-control | Czech | axSpA | 45 | HC | 20 | 37.8 (13.3)/ 37.3 (10.8) | 2 | Pubmed, Embase | [557] |
| Gupta | 2018 | case-control | India | AS | 36 | HC | 39 | 30 (20–35)/ 26 (18–36) | 3 | Embase | [558] |
| Al-Hindawi | 2023 | case-control | Iraq | axSpA | 74 | HC | 28 | 35.6 ± 0.9/- | 2 | Embase | [559] |
| Hulejova | 2019 | case-control | Czech Republic | AS | 61 | HC | 20 | -/- | 9 | Embase | [560] |

**Question 22: Gut microbiota**

| Author | Publish year | Design | Country | Patient diagnosis | Patient number | Control type | Control number | Measurement type | NOS score | Database | Reference number |
| --- | --- | --- | --- | --- | --- | --- | --- | --- | --- | --- | --- |
| Berland | 2023 | Case-control | France | SpA | 102 | HC | 63 | metagenomic shot- gun sequencing | 6 | Embase, PubMed | [561] |
| Berlinberg | 2021 | Case-control | USA | axSpA  CD  CD-axSpA | 21  27  12 | HC | 24 | metagenomic shotgun sequencing | 6 | Embase, PubMed | [562] |
| Breban | 2017 | Case-control | France | SpA  RA | 87  38 | HC | 69 | 16S rRNA gene V3-V4 region sequencing | 7 | Embase, PubMed | [563] |
| Cardoneanu | 2020 | Case-control | Romania | AS  UC  CD  IBD+AS | 28  27  29  17 | HC | 32 | 16S rRNA gene V3-V4 region sequencing | 5 | Embase, PubMed | [564] |
| Cardoneanu | 2021 | Case-control | Romania | AS | 28 | HC | 32 | 16S rRNA gene V3-V4 region sequencing | 5 | Embase, PubMed | [565] |
| Chen | 2019 | Case-control | China | AS | 41 | HC | 19 | 16S rRNA gene V4 region sequencing | 6 | Embase, PubMed | [566] |
| Chen | 2021 | Cohort | China | AS | 30 | HC | 24 | 16S rRNA gene V4 region sequencing | 5 | Embase, PubMed | [567] |
| Costello | 2015 | Case-control | Australia | AS | 9 | HC | 9 | culture-independent 16S rRNA gene V4 region sequencing | 6 | Embase, PubMed | [568] |
| Dai | 2022 | Cohort | China | AS | 24 | HC | 11 | 16S rRNA gene V3-V4 region sequencing | 4 | Embase, PubMed | [569] |
| Huang | 2019 | Cohort | China | AS | 29 | HC | 37 | metagenomic shotgun sequencing | 3 | Embase, PubMed | [570] |
| Klingberg | 2019 | Case-control | Sweden | AS  HC | 150  18 | HC | 17 | GA-mapTM Dysbiosis Test | 5 | Embase, PubMed | [571] |
| Li | 2023 | Case-control | China | AS | 193 | HC | 59 | metagenomic shotgun sequencing | 3 | Embase, PubMed | [572] |
| Liu | 2020 | Case-control | China | AS | 10 | HC | 12 | 16S rRNA gene V3-V4 region sequencing | 4 | Embase, PubMed | [573] |
| Min | 2023 | Case-control | South Korea | axSpA | 33 | HC | 20 | 16S rRNA gene V3-V4 region sequencing | 6 | Embase, PubMed | [574] |
| Sternes | 2022 | Case-control | Australia, Italy, Sweden | AS | 185 | HC | 105 | 16S rRNA gene V3-V4 region sequencing | 7 | Embase, PubMed | [575] |
| Thompson | 2023 | Case-control | USA | axSpA  RA  PsA  NIJP | 67  119  35  54 | HC | 165 | metagenomic shotgun sequencing | 6 | Embase, PubMed | [576] |
| Tito | 2016 | Case-control | Belgium | SpA | 27 | HC | 15 | 16S rRNA gene V4 region sequencing | 6 | Embase, PubMed | [577] |
| Wen | 2017 | Case-control | China | AS | 97 | HC | 114 | metagenomic shotgun sequencing | 5 | Embase, PubMed | [578] |
| Yin | 2019 | Cohort | China | AS | 127 | HC | 123 | metagenomic shotgun sequencing | 7 | Embase, PubMed | [579] |
| You | 2021 | Case-control | China | AS | 40 | HC | 20 | 16S rRNA gene V3-V4 region sequencing | 4 | Embase, PubMed | [580] |
| Zhang | 2019 | Case-control | China | AS | 103 | HC | 104 | 16S rRNA gene V3-V4 region sequencing | 6 | Embase, PubMed | [581] |
| Zhang | 2020 | Cohort | China | AS | 20 | HC | 20 | 16S rRNA gene V3-V4 region sequencing | 5 | Embase, PubMed | [582] |
| Zhang | 2020 | Cohort | China | AS | 78 | HC | 19 | 16S rRNA gene V3-V4 region sequencing | 5 | Embase, PubMed | [583] |
| Zhou | 2019 | Case-control | China | AS | 85 | HC | 62 | metagenomic shotgun sequencing | 6 | Embase, PubMed | [584] |

**Question 23: Metabonomics signature**

| Author | Publish year | Design | Country | Patient diagnosis | Patient number | Control type | Control number | Measurement type | NOS score | Database | Reference number |
| --- | --- | --- | --- | --- | --- | --- | --- | --- | --- | --- | --- |
| Berlinberg | 2021 | Case-control | USA | axSpA | 21 | HC  CD | 24  27 | LC-MS | 6 | Embase, PubMed | [562] |
| Chen | 2014 | Case-control | China | AS | 33 | HC | 33 | GC-MS | 5 | Embase, PubMed | [585] |
| Dogan | 2022 | Case-control | Turkey | AS | 18 | HC | 20 | LC-MS | 3 | Embase, PubMed | [586] |
| Fischer | 2011 | Case-control | UK | AS | 18 | HC | 9 | nLC-MS/MS | 4 | Embase, PubMed | [587] |
| Gao | 2009 | Case-control | China | AS | 15 | HC | 24 | GC-MS+LC-MS | 5 | Embase, PubMed | [588] |
| He | 2019 | Case-control | China | AS | 49 | HC | 38 | GC-MS | 6 | Embase, PubMed | [589] |
| Jiang | 2013 | Case-control | China | AS  GA  RA  OA | 27  33  27  27 | HC | 60 | GC−TOF MS  UPLC−QTOF MS | 4 | Embase, PubMed | [590] |
| Bogunia-Kubik | 2021 | Cohort | Poland | AS  RA  PsA | 29  26  23 |  |  | 1H NMR | 4 | Embase, PubMed | [591] |
| Li | 2022 | Case-control | China | AS | 20 | HC | 20 | UPLC–MS/MS | 3 | Embase, PubMed | [592] |
| Lv | 2021 | Case-control | China | AS | 37 | HC | 41 | GC-MS | 6 | Embase, PubMed | [593] |
| Onmaz | 2021 | Case-control | Turkey | AS | 85 | HC | 50 | tandem mass spectrometry | 4 | Embase, PubMed | [594] |
| Onmaz | 2021 | Cohort | Turkey | AS | 60 | HC | 60 | tandem mass spectrometry | 5 | Embase, PubMed | [595] |
| Ou | 2021 | Cohort | China | AS | 32 | HC | 40 | LC-MS | 7 | Embase, PubMed | [596] |
| Shao | 2016 | Case-control | China | AS  RA | 40  35 | HC | 34 | H1 NMR spectroscopy | 4 | Embase, PubMed | [597] |
| Stoll | 2016 | Case-control | USA | ERA | 14;  10 | HC | 9;  10 | LC-MS | 4 | Embase, PubMed | [598] |
| Wang | 2016 | Case-control | China | AS | 44 | HC | 44 | NMR spectroscopy | 4 | Embase, PubMed | [599] |
| Zhang | 2023 | Case-control | China | AS | NA | HC | NA | LC-MS | 3 | Embase, PubMed | [600] |
| Zhou | 2020 | Case-control | China | AS  RA | 30  32 | HC | 30 | UPLC-TQ-MS | 5 | Embase, PubMed | [601] |
| Du | 2023 | Case-control | China | AS  RA  SLE  SS  SSc  CTD | 36  186  84  39  12  6 | HC | 186 | LC-MS | 3 | Embase, PubMed | [602] |

**Question 24: NSAIDs-related genes**

| Author | Publish year | Design | Country | Patient diagnosis | Patient number | Control type | Control number | Age (year) Patient/control | NOS score | Database | Reference number |
| --- | --- | --- | --- | --- | --- | --- | --- | --- | --- | --- | --- |
| Forgerini | 2023 | Case-control | Brazil | gastrointestinal bleeding | 200 | HC | 706 | 60.2±16.3/59.8±15.8 | 6 | PubMed,  Embase | [603] |
| Forgerini | 2021 | Case-control | Brazil | gastrointestinal bleeding | 200 | HC | 706 | 60.2±16.3/59.8±15.8 | 6 | PubMed, | [604] |
| Groza | 2017 | Case-control | Romania | gastrointestinal bleeding | 163 | without gastrointestinal bleeding | 178 | 65(range 55-71)/64(range 52-73) | 6 | PubMed, | [605] |
| Figueiras | 2016 | Case-control | Spain and Italy | gastrointestinal bleeding | 557 | HC | 1343 | 62.77±16.856/63.45±15.585 | 6 | PubMed, Embase | [606] |
| Ishihara | 2014 | Case-control | Japan | NSAIDs user | 156 | HC | 422 | 68.2±12.2/53.1±10.0 | 5 | PubMed, Embase | [607] |
| Musumba | 2012 | Case-control | UK | peptic ulcer disease | 835 | without peptic ulcer disease | 404 | 65.2±13.6/57.6±14.7 | 4 | PubMed, Embase | [608] |
| Carbonell | 2010 | Case-control | France | gastrointestinal bleeding | 188 | HC | 263 | 65±17 | 4 | PubMed | [609] |
| Blanco | 2008 | Case-control | Spain | gastrointestinal bleeding | 134 | without gastrointestinal bleeding | 177 | 61.5±16.8/68.0±12.0 | 5 | PubMed, Embase | [610] |
| Ma | 2008 | Cross-sectional | China | NSAIDs user | 109 | - | - | 48±17 | 4 | PubMed, Embase | [611] |
| Pilotto | 2007 | Case-control | Italy | gastrointestinal bleeding | 26 | without gastrointestinal bleeding | 52 | 74.2±7.8/74.4±10.9 | 6 | PubMed, Embase | [612] |
| Vonkeman | 2006 | Case-control | Netherlands | NSAID-related ulcer | 26 | coumarin anticoagulants user | 87 | 74.5(range 32-96)/69(range 48-81) | 5 | PubMed, Embase | [613] |
| Skarke | 2006 | Case-control | Germany | healthy -765GG genotype carrier | 10 | healthy -765CC genotype carrier | 10 | 26.5±3.8/28.7±6.2 | 6 | PubMed | [614] |
| Martinez | 2004 | Case-control | Spain | gastrointestinal bleeding | 94 | without gastrointestinal bleeding | 124 | 62.2±19.9/63.8±12.7 | 5 | PubMed, Embase | [615] |
| Martin | 2001 | Case-control | New Zealand | NSAID-related ulcer | 23 | NSAIDs user without gastric ulcer | 31 | 68(range 28-87)/52(range 28-72) | 5 | PubMed | [616] |

**Question 25: SSZ-related genes**

| Author | Publish year | Design | Country | Patient diagnosis | Patient number | Control type | Control number | Age (year) Patient/control | NOS score | Database | Reference number |
| --- | --- | --- | --- | --- | --- | --- | --- | --- | --- | --- | --- |
| Ricart | 2002 | Retrospective cohort study | United States | UC | 64 | - | - | 47 (28–78) | 6 | PubMed, Embase | [617] |
| Chen | 2007 | Prospective  cohort study | China | IBD | 68 | HC | 109 | 39.19±11.74(with adverse effects)  42.36±17.81(without adverse effects) | 6 | PubMed, Embase | [618] |
| Hou | 2014 | Prospective  cohort study | China | AS | 266 | HC | 280 | 27.8± 9.1/35.1±10.0 | 8 | PubMed, Embase | [619] |
| Tanaka | 2002 | Retrospective cohort study | Japan | RA | 144 | - | - | 49.9 ± 13.4 | 6 | PubMed, Embase | [620] |
| Sabbagh | 1997 | Prospective  cohort study | France | CDLE | 11 | - | - | 38 (27-64) | 6 | PubMed, Embase | [621] |
| Tanigawara | 2002 | Retrospective cohort study | Japan | IBD | 13 | HC | 8 | 26—63 /23—32 | 4 | PubMed, Embase | [622] |
| Kumagai | 2004 | Retrospective cohort study | Japan | RA | 96 | HC | 180 | 58.0 ± 12.7/- | 5 | PubMed, Embase | [623] |
| Taniguchi | 2007 | Retrospective cohort study | Japan | RA | 186 | - | - | 55.1±12.9 (NAT2*4)  56.0±14.0 (without NAT2*4) | 4 | PubMed, Embase | [624] |
| Wiese | 2014 | Prospective  cohort study | Australia | RA | 229 | - | - | 56.4 (44.3–68.6) | 8 | PubMed, Embase | [625] |

**Question 26: Anti-drug antibodies**

| Author | Publish year | Design | Country | Patient diagnosis | Patient number | Control type | Control number | Age (year) Patient/control | NOS score | Database | Reference number |
| --- | --- | --- | --- | --- | --- | --- | --- | --- | --- | --- | --- |
| Ducourau | 2011 | Cohort | France | SpA | 91 | - | - | - | 6 | PubMed | [626] |
| Plasencia | 2013 | Cohort | Spain | SpA | 42 | - | - | 49.6±10.4 | 5 | PubMed | [627] |
| Paramarta | 2014 | Cohort | Netherlands | SpA | 26 | - | - | - | 4 | PubMed | [628] |
| Park | 2017 | Cohort | Korea | AS | 174 | - | - | - | 6 | PubMed | [629] |
| Gehin | 2019 | Cohort | Norway | SpA | 116 | - | - | - | 6 | PubMed | [630] |
| Ducourau | 2020 | Cohort | France | SpA | 107 | - | - | - | 7 | PubMed | [631] |
| Park | 2013 | Cohort | Korea | AS | 250 | - | - | 38.0 (18–69) | 6 | pubMed | [632] |
| Kneepkens | 2015 | Cohort | Netherlands | AS | 115 | - | - | 42±11 | 5 | PubMed | [633] |
| Jung | 2014 | Cohort | Korea | AS | 177 | - | - | 34.8±11.3 | 4 | PubMed | [634] |
| Hoxha | 2016 | Cohort | Italy | AS | 22 | - | - | 40.23 ± 10.87 | 3 | PubMed | [635] |
| de Vries | 2007 | Cohort | Netherlands | AS | 38 | - | - | 40±10 | 4 | pubMed | [636] |
| Braun | 2008 | Cohort | Germany | AS | 277 | - | - | - | 5 | pubMed | [637] |
| Arends | 2010 | Cohort | Netherlands | AS | 60 | - | - | - | 5 | PubMed | [638] |
| Su | 2020 | Cohort | Chinese | AS | 648 | - | - |  | 6 | PubMed | [639] |
| Mahmoud | 2020 | Cross-sectional | Tunisia | SpA | 71 | - | - | 43.05±11.2 | 5 | PubMed | [640] |
| de Vries | 2009 | Cohort | Netherlands | AS | 53 | - | - | 41±11 | 3 | PubMed | [641] |
| Balsa | 2018 | Cross-sectional | Spain | SpA | 293 | RA | 275 | 47.9 (11.5)/56.3 (12.1) | 4 | PubMed | [642] |
| Arstikyte | 2015 | Cross-sectional | Lithuania | SpA | 81 | - | - | 41.85 ± 11.23 | 4 | PubMed | [643] |
| Plasencia | 2012 | Cohort | Spain | SpA | 94 | - | - | 50±11 | 5 | PubMed | [644] |
| Méric | 2011 | Cohort | France | SpA | 32 | - | - | 48(23–73) | 4 | PubMed | [645] |

**REFERENCE**

1. Jajić I. The role of HLA-B27 in the diagnosis of low back pain. Acta Orthop Scand. 1979 Aug; 50(4):411-413.

2. Sadowska-Wróblewska M, Filipowicz A, Garwolinska H, Michalski J, Rusiniak B, Wróblewska T. Clinical symptoms and signs useful in the early diagnosis of ankylosing spondylitis. Clin Rheumatol. 1983 Mar; 2(1):37-43.

3. Deesomchok U, Tumrasvin T. Clinical study of Thai patients with ankylosing spondylitis. Clin Rheumatol. 1985 Mar; 4(1):76-82.

4. Sampaio-Barros PD, Bertolo MB, Kraemer MHS, Marques-Neto JF, Samara AM. Undifferentiated spondyloarthropathies: A 2-year follow-up study. Clinical Rheumatology. 2001; 20(3):201-206.

5. Brandt HC, Spiller I, Song IH, Vahldiek JL, Rudwaleit M, Sieper J. Performance of referral recommendations in patients with chronic back pain and suspected axial spondyloarthritis. Ann Rheum Dis. 2007 Nov; 66(11):1479-1484.

6. Dincer U, Cakar E, Kiralp MZ, Dursun H. Diagnosis delay in patients with ankylosing spondylitis: possible reasons and proposals for new diagnostic criteria. Clin Rheumatol. 2008 Apr; 27(4):457-462.

7. Bennett AN, McGonagle D, O'Connor P, Hensor EM, Sivera F, Coates LC, et al. Severity of baseline magnetic resonance imaging-evident sacroiliitis and HLA-B27 status in early inflammatory back pain predict radiographically evident ankylosing spondylitis at eight years. Arthritis Rheum. 2008 Nov; 58(11):3413-3418.

8. Nazarinia MA, Ghaffarpasand F, Heiran HR, Habibagahi Z. Pattern of ankylosing spondylitis in an Iranian population of 98 patients. Mod Rheumatol. 2009; 19(3):309-315.

9. Liao ZT, Pan YF, Huang JL, Huang F, Chi WJ, Zhang KX, et al. An epidemiological survey of low back pain and axial spondyloarthritis in a Chinese Han population. Scand J Rheumatol. 2009 Nov-Dec; 38(6):455-459.

10. Aggarwal R, Malaviya AN. Clinical characteristics of patients with ankylosing spondylitis in India. Clin Rheumatol. 2009 Oct; 28(10):1199-1205.

11. Liu X, Li YR, Hu LH, Zhou ZM, Chen FH, Ning Y, et al. High frequencies of HLA-B27 in Chinese patients with suspected of ankylosing spondylitis. Rheumatol Int. 2010 Aug; 30(10):1305-1309.

12. van Onna M, Jurik AG, van der Heijde D, van Tubergen A, Heuft-Dorenbosch L, Landewé R. HLA-B27 and gender independently determine the likelihood of a positive MRI of the sacroiliac joints in patients with early inflammatory back pain: a 2-year MRI follow-up study. Ann Rheum Dis. 2011 Nov; 70(11):1981-1985.

13. Liao Z, Lin Z, Xu M, Hu Z, Li T, Wei Q, et al. Clinical features of axial undifferentiated spondyloarthritis (USpA) in China: HLA-B27 is more useful for classification than MRI of the sacroiliac joint. Scand J Rheumatol. 2011 Nov; 40(6):439-443.

14. Chung HY, Machado P, van der Heijde D, D'Agostino MA, Dougados M. HLA-B27 positive patients differ from HLA-B27 negative patients in clinical presentation and imaging: results from the DESIR cohort of patients with recent onset axial spondyloarthritis. Ann Rheum Dis. 2011 Nov; 70(11):1930-1936.

15. De Carvalho HMS, Bortoluzzo AB, Gonçalves CR, Da Silva JAB, Ximenes AC, Bértolo MB, et al. Gender characterization in a large series of Brazilian patients with spondyloarthritis. Clinical Rheumatology. 2012; 31(4):687-695.

16. van den Berg R, de Hooge M, van Gaalen F, Reijnierse M, Huizinga T, van der Heijde D. Percentage of patients with spondyloarthritis in patients referred because of chronic back pain and performance of classification criteria: experience from the Spondyloarthritis Caught Early (SPACE) cohort. Rheumatology (Oxford). 2013 Aug; 52(8):1492-1499.

17. Qi J, Li Q, Lin Z, Liao Z, Wei Q, Cao S, et al. Higher risk of uveitis and dactylitis and older age of onset among ankylosing spondylitis patients with HLA-B*2705 than patients with HLA-B*2704 in the Chinese population. Tissue Antigens. 2013 Dec; 82(6):380-386.

18. Peláez-Ballestas I, Navarro-Zarza JE, Julian B, Lopez A, Flores-Camacho R, Casasola-Vargas JC, et al. A community-based study on the prevalence of spondyloarthritis and inflammatory back pain in Mexicans. J Clin Rheumatol. 2013 Mar; 19(2):57-61.

19. Tomero E, Mulero J, de Miguel E, Fernández-Espartero C, Gobbo M, Descalzo MA, et al. Performance of the Assessment of Spondyloarthritis International Society criteria for the classification of spondyloarthritis in early spondyloarthritis clinics participating in the ESPERANZA programme. Rheumatology (Oxford). 2014 Feb; 53(2):353-360.

20. Kassimos DG, Vassilakos J, Magiorkinis G, Garyfallos A. Prevalence and clinical manifestations of ankylosing spondylitis in young Greek males. Clin Rheumatol. 2014 Sep; 33(9):1303-1306.

21. Nakashima Y, Ohishi M, Okazaki K, Fukushi JI, Oyamada A, Hara D, et al. Delayed diagnosis of ankylosing spondylitis in a Japanese population. Modern Rheumatology. 2016; 26(3):421-425.

22. Burgos-Vargas R, Wei JCC, Rahman MU, Akkoc N, Haq SA, Hammoudeh M, et al. The prevalence and clinical characteristics of nonradiographic axial spondyloarthritis among patients with inflammatory back pain in rheumatology practices: A multinational, multicenter study. Arthritis Research and Therapy. 2016; 18(1).

23. Bautista-Molano W, Landewé RB, Londoño J, Romero-Sanchez C, Valle-Oñate R, van der Heijde D. Analysis and performance of various classification criteria sets in a Colombian cohort of patients with spondyloarthritis. Clin Rheumatol. 2016 Jul; 35(7):1759-1767.

24. Bandinelli F, Salvadorini G, Delle Sedie A, Riente L, Bombardieri S, Matucci-Cerinic M. Impact of gender, work, and clinical presentation on diagnostic delay in Italian patients with primary ankylosing spondylitis. Clin Rheumatol. 2016 Feb; 35(2):473-478.

25. Arnbak B, Grethe Jurik A, Hørslev-Petersen K, Hendricks O, Hermansen LT, Loft AG, et al. Associations Between Spondyloarthritis Features and Magnetic Resonance Imaging Findings: A Cross-Sectional Analysis of 1,020 Patients With Persistent Low Back Pain. Arthritis Rheumatol. 2016 Apr; 68(4):892-900.

26. Fırat SN, Yazıcı A, Yılmazer B, Coşan F, Savlı H, Cefle A. Low frequency of HLA-B27 in ankylosing spondylitis and its relationship with clinical findings in patients from Turkey. Eur J Rheumatol. 2017 Dec; 4(4):268-271.

27. Ez-Zaitouni Z, Bakker PAC, van Lunteren M, Berg IJ, Landewé R, van Oosterhout M, et al. Presence of multiple spondyloarthritis (SpA) features is important but not sufficient for a diagnosis of axial spondyloarthritis: data from the SPondyloArthritis Caught Early (SPACE) cohort. Ann Rheum Dis. 2017 Jun; 76(6):1086-1092.

28. Tong F, Lv Q, Li A, Fang L, Luo Z, Feng J, et al. An epidemiological study of the prevalence rate of inflammatory back pain and axial spondyloarthritis in a university in the south of China. Clin Rheumatol. 2018 Nov; 37(11):3087-3091.

29. Endo Y, Fujikawa K, Koga T, Mizokami A, Mine M, Tsukada T, et al. Characteristics of late-onset spondyloarthritis in Japan: A retrospective cohort study. Medicine (Baltimore). 2019 Feb; 98(7):e14431.

30. Passalent L, Sundararajan K, Perruccio AV, Hawke C, Coyte PC, Bombardier C, et al. Bridging the Gap Between Symptom Onset and Diagnosis in Axial Spondyloarthritis. Arthritis Care Res (Hoboken). 2022 Jun; 74(6):997-1005.

31. Edara M, Bhatt V, Zanwar A, Koneru K, Patel AM, Jawade P. A study of clinical, radiological features and HLA-B27 serology of axial spondyloarthropathy with comparison of radiographic and non-radiographic disease. J Family Med Prim Care. 2022 Aug; 11(8):4417-4423.

32. Chung HY, Huang JX, Lee KH, Tsang HHL, Lau CS, Chan SCW. MRI lesions in SpA: a comparison with noninflammatory back pain using propensity score adjustment method. Therapeutic Advances in Musculoskeletal Disease. 2022; 14.

33. Puhakka KB, Jurik AG, Schiottz-Christensen B, Hansen GV, Egund N, Christiansen JV, et al. Magnetic resonance imaging of sacroiliitis in early seronegative spondylarthropathy. Abnormalities correlated to clinical and laboratory findings. Rheumatology (Oxford). 2004 Feb; 43(2):234-237.

34. Ma HJ, Yin QF, Hu FP, Guo MH, Liu XD, Liu Y, et al. Different clinical features in patients with ankylosing spondylitis from southern and northern China. Int J Rheum Dis. 2012 Apr; 15(2):154-162.

35. Londono J, Romero-Sanchez MC, Torres VG, Bautista WA, Fernandez DJ, Quiroga JA, et al. The association between serum levels of potential biomarkers with the presence of factors related to the clinical activity and poor prognosis in spondyloarthritis. Revista Brasileira de Reumatologia. 2012; 52(4):536-544.

36. Weiss PF, Xiao R, Biko DM, Chauvin NA. Assessment of Sacroiliitis at Diagnosis of Juvenile Spondyloarthritis by Radiography, Magnetic Resonance Imaging, and Clinical Examination. Arthritis Care Res (Hoboken). 2016 Feb; 68(2):187-194.

37. Huerta-Sil G, Casasola-Vargas JC, Londoño JD, Rivas-Ruíz R, Chávez J, Pacheco-Tena C, et al. Low grade radiographic sacroiliitis as prognostic factor in patients with undifferentiated spondyloarthritis fulfilling diagnostic criteria for ankylosing spondylitis throughout follow up. Ann Rheum Dis. 2006 May; 65(5):642-646.

38. Sampaio-Barros PD, Bortoluzzo AB, Conde RA, Costallat LT, Samara AM, Bértolo MB. Undifferentiated spondyloarthritis: a longterm followup. J Rheumatol. 2010 Jun; 37(6):1195-1199.

39. Bakker PAC, Ramiro S, Ez-Zaitouni Z, van Lunteren M, Berg IJ, Landewé R, et al. Is it Useful to Repeat Magnetic Resonance Imaging of the Sacroiliac Joints After Three Months or One Year in the Diagnosis of Patients With Chronic Back Pain and Suspected Axial Spondyloarthritis? Arthritis Rheumatol. 2019 Mar; 71(3):382-391.

40. Lorenzin M, Ortolan A, Felicetti M, Vio S, Favero M, Polito P, et al. Spine and Sacroiliac Joints Lesions on Magnetic Resonance Imaging in Early Axial-Spondyloarthritis During 24-Months Follow-Up (Italian Arm of SPACE Study). Front Immunol. 2020; 11:936.

41. Baraliakos X, Szumski A, Koenig A, Jones H. C-reactive protein as a predictor of treatment response in patients with ankylosing spondylitis. Clinical and experimental rheumatology. 2014; 32(5):787.

42. Al-Saleh JA, Saab MA, Negm A, Balushi F, Namas R, Ziade N. Predictors of not Achieving Remission or Low Disease Activity in Axial Spondyloarthritis Patients from Middle Eastern Countries: A Prospective, Multicenter, Real-world Study. Oman Medical Journal. 2022; 37(3).

43. Yi L, Wang J, Guo X, Espitia MG, Chen E, Assassi S, et al. Profiling of hla-B alleles for association studies with ankylosing spondylitis in the chinese population. Open Rheumatol J. 2013; 7:51-54.

44. Siala M, Mahfoudh N, Gdoura R, Younes M, Fourati H, Kammoun A, et al. Distribution of HLA-B27 and its alleles in patients with reactive arthritis and with ankylosing spondylitis in Tunisia. Rheumatol Int. 2009 Aug; 29(10):1193-1196.

45. Ren EC, Koh WH, Sim D, Boey ML, Wee GB, Chan SH. Possible protective role of HLA-B*2706 for ankylosing spondylitis. Tissue Antigens. 1997 Jan; 49(1):67-69.

46. Pazar B, Safrany E, Gergely P, Szanto S, Szekanecz Z, Poor G. Association of ARTS1 gene polymorphisms with ankylosing spondylitis in the Hungarian population: the rs27044 variant is associated with HLA-B*2705 subtype in Hungarian patients with ankylosing spondylitis. J Rheumatol. 2010 Feb; 37(2):379-384.

47. Park SH, Kim J, Kim SG, Kim SK, Chung WT, Choe JY. Human leucocyte antigen-B27 subtypes in Korean patients with ankylosing spondylitis: higher B*2705 in the patient group. Int J Rheum Dis. 2009 Apr; 12(1):34-38.

48. Mou Y, Wu Z, Gu J, Liao Z, Lin Z, Wei Q, et al. HLA-B27 polymorphism in patients with juvenile and adult-onset ankylosing spondylitis in Southern China. Tissue Antigens. 2010 Jan; 75(1):56-60.

49. Ma HJ, Hu FP. Diversity of human leukocyte antigen-B27 alleles in Han population of Hunan province, southern China. Tissue Antigens. 2006 Aug; 68(2):163-166.

50. Luo F, Zhao Z, Zhang J, Leng J. Comparison of HLA-B*27 subtypes between Chinese patients with ankylosing spondylitis and non-ankylosing spondylitis carriers. J Int Med Res. 2019 Jul; 47(7):3171-3178.

51. Lopez-Larrea C, Gonzalez-Roces S, Pena M, Dominguez O, Coto E, Alvarez V, et al. Characterization of B27 haplotypes by oligotyping and genomic sequencing in the Mexican Mestizo population with ankylosing spondylitis: juvenile and adult onset. Hum Immunol. 1995 Jul; 43(3):174-180.

52. Lodhi NA, Bashir MM, Tipu HN, Hussain M. Distribution of HLA-B*27 Subtypes in Patients with Ankylosing Spondylitis in Local Population. J Coll Physicians Surg Pak. 2019 May; 29(5):418-421.

53. Liu X, Hu LH, Li YR, Chen FH, Ning Y, Yao QF. The association of HLA-B*27 subtypes with ankylosing spondylitis in Wuhan population of China. Rheumatol Int. 2010 Mar; 30(5):587-590.

54. Kchir MM, Hamdi W, Laadhar L, Kochbati S, Kaffel D, Saadellaoui K, et al. HLA-B, DR and DQ antigens polymorphism in Tunisian patients with ankylosing spondylitis (a case-control study). Rheumatol Int. 2010 May; 30(7):933-939.

55. Hou TY, Chen HC, Chen CH, Chang DM, Liu FC, Lai JH. Usefulness of human leucocyte antigen-B27 subtypes in predicting ankylosing spondylitis: Taiwan experience. Intern Med J. 2007 Nov; 37(11):749-752.

56. Harfouch EI, Al-Cheikh SA. HLA-B27 and its subtypes in Syrian patients with ankylosing spondylitis. Saudi Med J. 2011 Apr; 32(4):364-368.

57. Gonzalez-Roces S, Alvarez MV, Gonzalez S, Dieye A, Makni H, Woodfield DG, et al. HLA-B27 polymorphism and worldwide susceptibility to ankylosing spondylitis. Tissue Antigens. 1997 Feb; 49(2):116-123.

58. Diyarbakir E, Eyerci N, Melikoglu M, Topcu A, Pirim I. HLA B27 subtype distribution among patients with ankylosing spondylitis in eastern Turkey. Genet Test Mol Biomarkers. 2012 May; 16(5):456-458.

59. Cipriani A, Rivera S, Hassanhi M, Marquez G, Hernandez R, Villalobos C, et al. HLA-B27 subtypes determination in patients with ankylosing spondylitis from Zulia, Venezuela. Hum Immunol. 2003 Jul; 64(7):745-749.

60. Chou CT, Chen JM, Hsu CM, Chen SJ. HLA-B27 and its subtypes in 4 Taiwanese Aborigine tribes: a comparison to Han Chinese patients with ankylosing spondylitis. J Rheumatol. 2003 Feb; 30(2):321-325.

61. Chavan H, Samant R, Deshpande A, Mankeshwar R. Correlation of HLA B27 subtypes with clinical features of ankylosing spondylitis. Int J Rheum Dis. 2011 Oct; 14(4):369-374.

62. Birinci A, Bilgici A, Kuru O, Durupinar B. HLA-B27 polymorphism in Turkish patients with ankylosing spondylitis. Rheumatol Int. 2006 Feb; 26(4):285-287.

63. Ben Radhia K, Ayed-Jendoubi S, Sfar I, Ben Romdhane T, Makhlouf M, Gorgi Y, et al. Distribution of HLA-B*27 subtypes in Tunisians and their association with ankylosing spondylitis. Joint Bone Spine. 2008 Mar; 75(2):172-175.

64. Alaez C, Gazit E, Ibarrola B, Yaron M, Livneh A, Avishai O, et al. Distribution of HLA-B27 subtypes in ankylosing spondylitis in an Israeli population. Arch Med Res. 2007 May; 38(4):452-455.

65. Acar M, Cora T, Tunc R, Acar H. HLA-B27 subtypes in Turkish patients with ankylosing spondylitis and healthy controls. Rheumatol Int. 2012 Oct; 32(10):3103-3105.

66. Varnavidou-Nicolaidou A, Karpasitou K, Georgiou D, Stylianou G, Kokkofitou A, Michalis C, et al. HLA-B27 in the Greek Cypriot population: distribution of subtypes in patients with ankylosing spondylitis and other HLA-B27-related diseases. The possible protective role of B*2707. Hum Immunol. 2004 Dec; 65(12):1451-1454.

67. Lopez-Larrea C, Blanco-Gelaz MA, Torre-Alonso JC, Bruges Armas J, Suarez-Alvarez B, Pruneda L, et al. Contribution of KIR3DL1/3DS1 to ankylosing spondylitis in human leukocyte antigen-B27 Caucasian populations. Arthritis Res Ther. 2006; 8(4):R101.

68. Gonzalez S, Garcia-Fernandez S, Martinez-Borra J, Blanco-Gelaz MA, Rodrigo L, Sanchez del Rio J, et al. High variability of HLA-B27 alleles in ankylosing spondylitis and related spondyloarthropathies in the population of northern Spain. Hum Immunol. 2002 Aug; 63(8):673-676.

69. Diaz-Pena R, Vidal-Castineira JR, Lopez-Vazquez A, Lopez-Larrea C. HLA-B*40:01 Is Associated with Ankylosing Spondylitis in HLA-B27-positive Populations. J Rheumatol. 2016 Jun; 43(6):1255-1256.

70. Chen IH, Yang KL, Lee A, Huang HH, Lin PY, Lee TD. Low frequency of HLA-B*2706 in Taiwanese patients with ankylosing spondylitis. Eur J Immunogenet. 2002 Oct; 29(5):435-438.

71. Yamaguchi A, Ogawa A, Tsuchiya N, Shiota M, Mitsui H, Tokunaga K, et al. HLA-B27 subtypes in Japanese with seronegative spondyloarthropathies and healthy controls. J Rheumatol. 1996 Jul; 23(7):1189-1193.

72. Wu X, Wu J, Li X, Wei Q, Lv Q, Zhang P, et al. The Clinical Characteristics of Other HLA-B Types in Chinese Ankylosing Spondylitis Patients. Front Med (Lausanne). 2020; 7:568790.

73. Van Gaalen FA. Does HLA-B*2706 protect against ankylosing spondylitis? A meta-analysis. Int J Rheum Dis. 2012 Feb; 15(1):8-12.

74. Oguz FS, Ocal L, Diler AS, Ozkul H, Asicioglu F, Kasapoglu E, et al. HLA B-27 subtypes in Turkish patients with spondyloarthropathy and healthy controls. Dis Markers. 2004; 20(6):309-312.

75. Mou Y, Zhang P, Li Q, Lin Z, Liao Z, Wei Q, et al. Clinical Features in Juvenile-Onset Ankylosing Spondylitis Patients Carrying Different B27 Subtypes. Biomed Res Int. 2015; 2015:594878.

76. Lopez-Larrea C, Sujirachato K, Mehra NK, Chiewsilp P, Isarangkura D, Kanga U, et al. HLA-B27 subtypes in Asian patients with ankylosing spondylitis. Evidence for new associations. Tissue Antigens. 1995 Mar; 45(3):169-176.

77. Kanga U, Mehra NK, Larrea CL, Lardy NM, Kumar A, Feltkamp TE. Seronegative spondyloarthropathies and HLA-B27 subtyes: a study in Asian Indians. Clin Rheumatol. 1996 Jan; 15 Suppl 1:13-18.

78. Fouladi S, Adib M, Salehi M, Karimzadeh H, Bakhshiani Z, Ostadi V. Distribution of HLA-B*27 alleles in patients with ankylosing spondylitis in Iran. Iran J Immunol. 2009 Mar; 6(1):49-54.

79. Liu Y, Jiang L, Cai Q, Danoy P, Barnardo MC, Brown MA, et al. Predominant association of HLA-B*2704 with ankylosing spondylitis in Chinese Han patients. Tissue Antigens. 2010 Jan; 75(1):61-64.

80. Yang T, Duan Z, Wu S, Liu S, Zeng Z, Li G, et al. Association of HLA-B27 genetic polymorphisms with ankylosing spondylitis susceptibility worldwide: a meta-analysis. Mod Rheumatol. 2014 Jan; 24(1):150-161.

81. Lin H, Gong YZ. Association of HLA-B27 with ankylosing spondylitis and clinical features of the HLA-B27-associated ankylosing spondylitis: a meta-analysis. Rheumatol Int. 2017 Aug; 37(8):1267-1280.

82. International Genetics of Ankylosing Spondylitis C, Cortes A, Hadler J, Pointon JP, Robinson PC, Karaderi T, et al. Identification of multiple risk variants for ankylosing spondylitis through high-density genotyping of immune-related loci. Nat Genet. 2013 Jul; 45(7):730-738.

83. Ellinghaus D, Jostins L, Spain SL, Cortes A, Bethune J, Han B, et al. Analysis of five chronic inflammatory diseases identifies 27 new associations and highlights disease-specific patterns at shared loci. Nat Genet. 2016 May; 48(5):510-518.

84. Robinson PC, Leo PJ, Pointon JJ, Harris J, Cremin K, Bradbury LA, et al. Exome-wide study of ankylosing spondylitis demonstrates additional shared genetic background with inflammatory bowel disease. NPJ Genom Med. 2016; 1:16008.

85. Soomro M, Stadler M, Dand N, Bluett J, Jadon D, Jalali-Najafabadi F, et al. Comparative Genetic Analysis of Psoriatic Arthritis and Psoriasis for the Discovery of Genetic Risk Factors and Risk Prediction Modeling. Arthritis Rheumatol. 2022 Sep; 74(9):1535-1543.

86. Bettencourt BF, Rocha FL, Alves H, Amorim R, Caetano-Lopes J, Vieira-Sousa E, et al. Protective effect of an ERAP1 haplotype in ankylosing spondylitis: investigating non-MHC genes in HLA-B27-positive individuals. Rheumatology (Oxford). 2013 Dec; 52(12):2168-2176.

87. Lin Z, Bei JX, Shen M, Li Q, Liao Z, Zhang Y, et al. A genome-wide association study in Han Chinese identifies new susceptibility loci for ankylosing spondylitis. Nat Genet. 2011 Dec 4; 44(1):73-77.

88. Zvyagin IV, Dorodnykh VY, Mamedov IZ, Staroverov DB, Bochkova AG, Rebrikov DV, et al. Association of ERAP1 Allelic Variants with Risk of Ankylosing Spondylitis. Acta Naturae. 2010 Jul; 2(3):72-77.

89. Zhang Z, Dai D, Yu K, Yuan F, Jin J, Ding L, et al. Association of HLA-B27 and ERAP1 with ankylosing spondylitis susceptibility in Beijing Han Chinese. Tissue Antigens. 2014 May; 83(5):324-329.

90. Zhang L, Fan D, Liu L, Yang T, Ding N, Hu Y, et al. Association Study of IL-12B Polymorphisms Susceptibility with Ankylosing Spondylitis in Mainland Han Population. PLoS One. 2015; 10(6):e0130982.

91. Wisniewski A, Kasprzyk S, Majorczyk E, Nowak I, Wilczynska K, Chlebicki A, et al. ERAP1-ERAP2 haplotypes are associated with ankylosing spondylitis in Polish patients. Hum Immunol. 2019 May; 80(5):339-343.

92. Daryabor G, Mahmoudi M, Jamshidi A, Nourijelyani K, Amirzargar A, Ahmadzadeh N, et al. Determination of IL-23 receptor gene polymorphism in Iranian patients with ankylosing spondylitis. Eur Cytokine Netw. 2014 Mar 1; 25(1):24-29.

93. Wellcome Trust Case Control C, Australo-Anglo-American Spondylitis C, Burton PR, Clayton DG, Cardon LR, Craddock N, et al. Association scan of 14,500 nonsynonymous SNPs in four diseases identifies autoimmunity variants. Nat Genet. 2007 Nov; 39(11):1329-1337.

94. Australo-Anglo-American Spondyloarthritis C, Reveille JD, Sims AM, Danoy P, Evans DM, Leo P, et al. Genome-wide association study of ankylosing spondylitis identifies non-MHC susceptibility loci. Nat Genet. 2010 Feb; 42(2):123-127.

95. Rostami S, Hoff M, Brown MA, Hveem K, Holmen OL, Fritsche LG, et al. Prediction of Ankylosing Spondylitis in the HUNT Study by a Genetic Risk Score Combining 110 Single-nucleotide Polymorphisms of Genome-wide Significance. J Rheumatol. 2020 Feb; 47(2):204-210.

96. Jung SH, Cho SM, Yim SH, Kim SH, Park HC, Cho ML, et al. Developing a Risk-scoring Model for Ankylosing Spondylitis Based on a Combination of HLA-B27, Single-nucleotide Polymorphism, and Copy Number Variant Markers. J Rheumatol. 2016 Dec; 43(12):2136-2141.

97. Li Z, Wu X, Leo PJ, De Guzman E, Akkoc N, Breban M, et al. Polygenic Risk Scores have high diagnostic capacity in ankylosing spondylitis. Ann Rheum Dis. 2021 Sep; 80(9):1168-1174.

98. Huang XF, Li Z, De Guzman E, Robinson P, Gensler L, Ward MM, et al. Genomewide Association Study of Acute Anterior Uveitis Identifies New Susceptibility Loci. Invest Ophthalmol Vis Sci. 2020 Jun 3; 61(6):3.

99. Thomas GP, Willner D, Robinson PC, Cortes A, Duan R, Rudwaleit M, et al. Genetic diagnostic profiling in axial spondyloarthritis: a real world study. Clin Exp Rheumatol. 2017 Mar-Apr; 35(2):229-233.

100. van Gaalen FA, Verduijn W, Roelen DL, Bohringer S, Huizinga TW, van der Heijde DM, et al. Epistasis between two HLA antigens defines a subset of individuals at a very high risk for ankylosing spondylitis. Ann Rheum Dis. 2013 Jun; 72(6):974-978.

101. Wei JC, Sung-Ching HW, Hsu YW, Wen YF, Wang WC, Wong RH, et al. Interaction between HLA-B60 and HLA-B27 as a Better Predictor of Ankylosing Spondylitis in a Taiwanese Population. PLoS One. 2015; 10(10):e0137189.

102. Wei JC, Tsai WC, Lin HS, Tsai CY, Chou CT. HLA-B60 and B61 are strongly associated with ankylosing spondylitis in HLA-B27-negative Taiwan Chinese patients. Rheumatology (Oxford). 2004 Jul; 43(7):839-842.

103. Atouf O, Benbouazza K, Brick C, Saoud B, Benseffaj N, Amine B, et al. Distribution of HLA class I and II genes in ankylosing spondylitis patients from Morocco. Pathol Biol (Paris). 2012 Dec; 60(6):e80-83.

104. Reveille JD, Zhou X, Lee M, Weisman MH, Yi L, Gensler LS, et al. HLA class I and II alleles in susceptibility to ankylosing spondylitis. Ann Rheum Dis. 2019 Jan; 78(1):66-73.

105. Santos AM, Pena P, Avila M, Briceno I, Jaramillo C, Vargas-Alarcon G, et al. Association of human leukocyte A, B, and DR antigens in Colombian patients with diagnosis of spondyloarthritis. Clin Rheumatol. 2017 Apr; 36(4):953-958.

106. Helenius LM, Hallikainen D, Helenius I, Meurman JH, Koskimies S, Tervahartiala P, et al. HLA-DRB1* alleles and temporomandibular joint erosion in patients with various rheumatic diseases. Scand J Rheumatol. 2004; 33(1):24-29.

107. Jaakkola E, Herzberg I, Laiho K, Barnardo MC, Pointon JJ, Kauppi M, et al. Finnish HLA studies confirm the increased risk conferred by HLA-B27 homozygosity in ankylosing spondylitis. Ann Rheum Dis. 2006 Jun; 65(6):775-780.

108. La Nasa G, Mathieu A, Mulargia M, Carcassi C, Vacca A, Ledda A, et al. Association of the HLA-A2, CW2, B27, S31, DR2 haplotype with ankylosing spondylitis. A possible role of non-B27 factors in the disease. Dis Markers. 1993 Nov; 11(4):191-203.

109. Madhavan R, Parthiban M, Rajendran CP, Chandrasekaran AN, Zake L, Sanjeevi CB. HLA class I and class II association with ankylosing spondylitis in a southern Indian population. Ann N Y Acad Sci. 2002 Apr; 958:403-407.

110. Monowarul Islam SM, Numaga J, Fujino Y, Masuda K, Ohda H, Hirata R, et al. HLA-DR8 and acute anterior uveitis in ankylosing spondylitis. Arthritis Rheum. 1995 Apr; 38(4):547-550.

111. Perez-Guijo V, Munoz E, Escudero A, Veroz R, Sanchez M, Munoz-Villanueva MC, et al. Distribution of HLA-DRB1 genes in patients with sporadic ankylosing spondylitis in the south of Spain. Joint Bone Spine. 2002 Oct; 69(5):458-462.

112. Ploski R, Flato B, Vinje O, Maksymowych W, Forre O, Thorsby E. Association to HLA-DRB1*08, HLA-DPB1*0301 and homozygosity for an HLA-linked proteasome gene in juvenile ankylosing spondylitis. Hum Immunol. 1995 Oct; 44(2):88-96.

113. Yang C, Zhu C, Du F, Song Y, Jiang M, Zhang Y, et al. [Association of HLA-B27 subtype, HLA-B, HLA-DRB1 and HLA-DQB1 alleles with ankylosing spondylitis]. Chinese Journal of Blood Transfusion. 2010; 23(8):620-623.

114. Cai Q, Han X, Jiang R, Yang B, Lv J. [Study on the correlation between ankylosing spondylitis and HLA-B and HLA-DRB1]. National Medical Journal of China. 2005; 85(14):992-994.

115. Baraliakos X, Baerlecken N, Witte T, Heldmann F, Braun J. High prevalence of anti-CD74 antibodies specific for the HLA class II-associated invariant chain peptide (CLIP) in patients with axial spondyloarthritis. Ann Rheum Dis. 2014 Jun; 73(6):1079-1082.

116. Baerlecken NT, Nothdorft S, Stummvoll GH, Sieper J, Rudwaleit M, Reuter S, et al. Autoantibodies against CD74 in spondyloarthritis. Ann Rheum Dis. 2014 Jun; 73(6):1211-1214.

117. Abdelaziz MM, Gamal RM, Ismail NM, Lafy RA, Hetta HF. Diagnostic value of anti-CD74 antibodies in early and late axial spondyloarthritis and its relationship to disease activity. Rheumatology (Oxford). 2021 Jan 5; 60(1):263-268.

118. Do L, Granasen G, Hellman U, Lejon K, Geijer M, Baraliakos X, et al. Anti-CD74 IgA autoantibodies in radiographic axial spondyloarthritis: a longitudinal Swedish study. Rheumatology (Oxford). 2021 Sep 1; 60(9):4085-4093.

119. de Winter JJ, van de Sande MG, Baerlecken N, Berg I, Ramonda R, van der Heijde D, et al. Anti-CD74 antibodies have no diagnostic value in early axial spondyloarthritis: data from the spondyloarthritis caught early (SPACE) cohort. Arthritis Res Ther. 2018 Mar 1; 20(1):38.

120. Riechers E, Baerlecken N, Baraliakos X, Achilles-Mehr Bakhsh K, Aries P, Bannert B, et al. Sensitivity and Specificity of Autoantibodies Against CD74 in Nonradiographic Axial Spondyloarthritis. Arthritis Rheumatol. 2019 May; 71(5):729-735.

121. Hu CJ, Li MT, Li X, Peng LY, Zhang SZ, Leng XM, et al. CD74 auto-antibodies display little clinical value in Chinese Han population with axial spondyloarthritis. Medicine (Baltimore). 2020 Dec 11; 99(50):e23433.

122. Ziade NR, Mallak I, Merheb G, Ghorra P, Baerlecken N, Witte T, et al. Added Value of Anti-CD74 Autoantibodies in Axial SpondyloArthritis in a Population With Low HLA-B27 Prevalence. Front Immunol. 2019; 10:574.

123. Colak S, Turgay TM, Kucuksahin O, Duman MT, Cetinkaya H, Toruner M. The association of anti-CD74 antibody with spondyloarthropathies. Eur J Rheumatol. 2021 Oct; 8(4):207-210.

124. Liu Y, Liao X, Shi G. Autoantibodies in Spondyloarthritis, Focusing on Anti-CD74 Antibodies. Front Immunol. 2019; 10:5.

125. Tsui FW, Tsui HW, Las Heras F, Pritzker KP, Inman RD. Serum levels of novel noggin and sclerostin-immune complexes are elevated in ankylosing spondylitis. Ann Rheum Dis. 2014 Oct; 73(10):1873-1879.

126. Rosenberg JN, Johnson GD, Holborow EJ. Antinuclear antibodies in ankylosing spondylitis, psoriatic arthritis, and psoriasis. Ann Rheum Dis. 1979 Dec; 38(6):526-528.

127. de Vries M, van der Horst-Bruinsma I, van Hoogstraten I, van Bodegraven A, von Blomberg BM, Ratnawati H, et al. pANCA, ASCA, and OmpC antibodies in patients with ankylosing spondylitis without inflammatory bowel disease. J Rheumatol. 2010 Nov; 37(11):2340-2344.

128. Matzkies FG, Targan SR, Berel D, Landers CJ, Reveille JD, McGovern DP, et al. Markers of intestinal inflammation in patients with ankylosing spondylitis: a pilot study. Arthritis Res Ther. 2012 Nov 29; 14(6):R261.

129. Stone MA, Payne U, Schentag C, Rahman P, Pacheco-Tena C, Inman RD. Comparative immune responses to candidate arthritogenic bacteria do not confirm a dominant role for Klebsiella pneumonia in the pathogenesis of familial ankylosing spondylitis. Rheumatology (Oxford). 2004 Feb; 43(2):148-155.

130. Stebbings S, Munro K, Simon MA, Tannock G, Highton J, Harmsen H, et al. Comparison of the faecal microflora of patients with ankylosing spondylitis and controls using molecular methods of analysis. Rheumatology (Oxford). 2002 Dec; 41(12):1395-1401.

131. Tiwana H, Wilson C, Walmsley RS, Wakefield AJ, Smith MS, Cox NL, et al. Antibody responses to gut bacteria in ankylosing spondylitis, rheumatoid arthritis, Crohn's disease and ulcerative colitis. Rheumatol Int. 1997; 17(1):11-16.

132. Collado A, Gratacos J, Ebringer A, Rashid T, Marti A, Sanmarti R, et al. Serum IgA anti-Klebsiella antibodies in ankylosing spondylitis patients from Catalonia. Scand J Rheumatol. 1994; 23(3):119-123.

133. Cooper R, Fraser SM, Sturrock RD, Gemmell CG. Raised titres of anti-klebsiella IgA in ankylosing spondylitis, rheumatoid arthritis, and inflammatory bowel disease. Br Med J (Clin Res Ed). 1988 May 21; 296(6634):1432-1434.

134. Csango PA, Upsahl MT, Romberg O, Kornstad L, Sarov I. Chlamydia trachomatis serology in ankylosing spondylitis. Clin Rheumatol. 1987 Sep; 6(3):384-390.

135. Kihlstrom E, Gronberg A, Bengtsson A. Immunoblot analysis of antibody response to Chlamydia trachomatis in patients with reactive arthritis and ankylosing spondylitis. Scand J Rheumatol. 1989; 18(6):377-383.

136. Kumar P, Bhakuni DS, Rastogi S. Diagnosis of Chlamydia trachomatis in patients with reactive arthritis and undifferentiated spondyloarthropathy. J Infect Dev Ctries. 2014 May 14; 8(5):648-654.

137. Tsuchiya N, Husby G, Williams RC, Jr. Antibodies to the peptide from the plasmid-coded Yersinia outer membrane protein (YOP1) in patients with ankylosing spondylitis. Clin Exp Immunol. 1990 Dec; 82(3):493-498.

138. Zambrano-Zaragoza JF, de Jesus Duran-Avelar M, Rodriguez-Ocampo AN, Garcia-Latorre E, Burgos-Vargas R, Dominguez-Lopez ML, et al. The 30-kDa band from Salmonella typhimurium: IgM, IgA and IgG antibody response in patients with ankylosing spondylitis. Rheumatology (Oxford). 2009 Jul; 48(7):748-754.

139. Andretta MA, Vieira TD, Nishiara R, Skare TL. Anti-Saccharomyces cerevisiae (ASCA) and anti-endomysial antibodies in spondyloarthritis. Rheumatol Int. 2012 Feb; 32(2):551-554.

140. Aydin SZ, Atagunduz P, Temel M, Bicakcigil M, Tasan D, Direskeneli H. Anti-Saccharomyces cerevisiae antibodies (ASCA) in spondyloarthropathies: a reassessment. Rheumatology (Oxford). 2008 Feb; 47(2):142-144.

141. Hoffman IE, Demetter P, Peeters M, De Vos M, Mielants H, Veys EM, et al. Anti-saccharomyces cerevisiae IgA antibodies are raised in ankylosing spondylitis and undifferentiated spondyloarthropathy. Ann Rheum Dis. 2003 May; 62(5):455-459.

142. Mundwiler ML, Mei L, Landers CJ, Reveille JD, Targan S, Weisman MH. Inflammatory bowel disease serologies in ankylosing spondylitis patients: a pilot study. Arthritis Res Ther. 2009; 11(6):R177.

143. Rodrigues IK, Andrigueti M, de Oliveira Gil ID, de Lucca Schiavon L, de Andrade KR, Pereira IA, et al. An investigation into the relationship between anti-Helicobacter pylori and anti-Saccharomyces cerevisiae antibodies in patients with axial spondyloarthritis and Crohn disease. Rheumatol Int. 2015 Feb; 35(2):359-366.

144. Wallis D, Asaduzzaman A, Weisman M, Haroon N, Anton A, McGovern D, et al. Elevated serum anti-flagellin antibodies implicate subclinical bowel inflammation in ankylosing spondylitis: an observational study. Arthritis Research & Therapy. 2013; 15(5).

145. Tani Y, Sato H, Tanaka N, Hukuda S. Antibodies against bacterial lipopolysaccharides in Japanese patients with ankylosing spondylitis. Br J Rheumatol. 1997 Apr; 36(4):491-493.

146. Dominguez-Lopez ML, Burgos-Vargas R, Galicia-Serrano H, Bonilla-Sanchez MT, Rangel-Acosta HH, Cancino-Diaz ME, et al. IgG antibodies to enterobacteria 60 kDa heat shock proteins in the sera of HLA-B27 positive ankylosing spondylitis patients. Scand J Rheumatol. 2002; 31(5):260-265.

147. Maki-Ikola O, Leirisalo-Repo M, Turunen U, Granfors K. Association of gut inflammation with increased serum IgA class Klebsiella antibody concentrations in patients with axial ankylosing spondylitis (AS): implication for different aetiopathogenetic mechanisms for axial and peripheral AS? Ann Rheum Dis. 1997 Mar; 56(3):180-183.

148. Lee JS, Lee EJ, Lee JH, Hong SC, Lee CK, Yoo B, et al. Autoantibodies against Protein Phosphatase Magnesium-Dependent 1A as a Biomarker for Predicting Radiographic Progression in Ankylosing Spondylitis Treated with Anti-Tumor Necrosis Factor Agents. J Clin Med. 2020 Dec 7; 9(12).

149. Kim YG, Sohn DH, Zhao X, Sokolove J, Lindstrom TM, Yoo B, et al. Role of protein phosphatase magnesium-dependent 1A and anti-protein phosphatase magnesium-dependent 1A autoantibodies in ankylosing spondylitis. Arthritis Rheumatol. 2014 Oct; 66(10):2793-2803.

150. Torok HP, Glas J, Gruber R, Brumberger V, Strasser C, Kellner H, et al. Inflammatory bowel disease-specific autoantibodies in HLA-B27-associated spondyloarthropathies: increased prevalence of ASCA and pANCA. Digestion. 2004; 70(1):49-54.

151. Wu J, Yan L, Chai K. Systemic immune-inflammation index is associated with disease activity in patients with ankylosing spondylitis. J Clin Lab Anal. 2021 Sep; 35(9):e23964.

152. Kwan YH, Tan JJ, Phang JK, Fong W, Lim KK, Koh HL, et al. Validity and reliability of the Ankylosing Spondylitis Disease Activity Score with C-reactive protein (ASDAS-CRP) and Bath Ankylosing Spondylitis Disease Activity Index (BASDAI) in patients with axial spondyloarthritis (axSpA) in Singapore. Int J Rheum Dis. 2019 Dec; 22(12):2206-2212.

153. Akbal A, Resorlu H, Gokmen F, Savas Y, Zateri C, Sargin B, et al. The relationship between C-reactive protein rs3091244 polymorphism and ankylosing spondylitis. Int J Rheum Dis. 2016 Jan; 19(1):43-48.

154. Sundaram TG, Muhammed H, Aggarwal A, Gupta L. A prospective study of novel disease activity indices for ankylosing spondylitis. Rheumatol Int. 2020 Nov; 40(11):1843-1849.

155. Navarini L, Currado D, Marino A, Di Donato S, Biaggi A, Caso F, et al. Persistence of C-reactive protein increased levels and high disease activity are predictors of cardiovascular disease in patients with axial spondyloarthritis. Sci Rep. 2022 May 7; 12(1):7498.

156. Ben-Shabat N, Shabat A, Watad A, Kridin K, Bragazzi NL, McGonagle D, et al. Mortality in Ankylosing Spondylitis According to Treatment: A Nationwide Retrospective Cohort Study of 5,900 Patients From Israel. Arthritis Care Res (Hoboken). 2022 Oct; 74(10):1614-1622.

157. Su J, Cui L, Yang W, Shi H, Jin C, Shu R, et al. Baseline high-sensitivity C-reactive protein predicts the risk of incident ankylosing spondylitis: Results of a community-based prospective study. PLoS One. 2019; 14(2):e0211946.

158. Cowling P, Ebringer R, Cawdell D, Ishii M, Ebringer A. C-reactive protein, ESR, and klebsiella in ankylosing spondylitis. Ann Rheum Dis. 1980 Feb; 39(1):45-49.

159. Bedaiwi MK, AlRasheed RF, Bin Zuair A, Alqurtas EM, Baeshen MO, Omair MA. A cross-sectional study on clinical characteristics of Saudi axial spondylarthritis: preliminary results. Eur Rev Med Pharmacol Sci. 2021 Aug; 25(16):5241-5247.

160. Chen CH, Chen HA, Liao HT, Liu CH, Tsai CY, Chou CT. The clinical usefulness of ESR, CRP, and disease duration in ankylosing spondylitis: the product of these acute-phase reactants and disease duration is associated with patient's poor physical mobility. Rheumatol Int. 2015 Jul; 35(7):1263-1267.

161. Kilic E, Kilic G, Akgul O, Ozgocmen S. Discriminant validity of the Ankylosing Spondylitis Disease Activity Score (ASDAS) in patients with non-radiographic axial spondyloarthritis and ankylosing spondylitis: a cohort study. Rheumatol Int. 2015 Jun; 35(6):981-989.

162. Benhamou M, Gossec L, Dougados M. Clinical relevance of C-reactive protein in ankylosing spondylitis and evaluation of the NSAIDs/coxibs' treatment effect on C-reactive protein. Rheumatology (Oxford). 2010 Mar; 49(3):536-541.

163. de Vries MK, van Eijk IC, van der Horst-Bruinsma IE, Peters MJ, Nurmohamed MT, Dijkmans BA, et al. Erythrocyte sedimentation rate, C-reactive protein level, and serum amyloid a protein for patient selection and monitoring of anti-tumor necrosis factor treatment in ankylosing spondylitis. Arthritis Rheum. 2009 Nov 15; 61(11):1484-1490.

164. Siebuhr AS, Husakova M, Forejtova S, Zegzulkova K, Tomcik M, Urbanova M, et al. Metabolites of C-reactive protein and vimentin are associated with disease activity of axial spondyloarthritis. Clin Exp Rheumatol. 2019 May-Jun; 37(3):358-366.

165. Li HG, Wang DM, Shen FC, Huang SX, Hou ZD, Lin L, et al. Risk factors for progression of juvenile-onset non-radiographic axial spondyloarthritis to juvenile-onset ankylosing spondylitis: A nested case-control study. RMD Open. 2021; 7(3).

166. Huang Y, Deng W, Pan X, Liu M, Zhong Z, Huang Q, et al. The relationship between platelet to albumin ratio and disease activity in axial spondyloarthritis patients. Mod Rheumatol. 2022 Aug 20; 32(5):974-979.

167. Senna MK, Olama SM, El-Arman M. Serum melatonin level in ankylosing spondylitis: is it increased in active disease? Rheumatol Int. 2012 Nov; 32(11):3429-3433.

168. Tang Y, Yang P, Wang F, Xu H, Zong SY. Association of polymorphisms in ERAP1 and risk of ankylosing spondylitis in a Chinese population. Gene. 2018 Mar 10; 646:8-11.

169. Sun X, Zhou C, Zhu J, Wu S, Liang T, Jiang J, et al. Identification of clinical heterogeneity and construction of a novel subtype predictive model in patients with ankylosing spondylitis: An unsupervised machine learning study. Int Immunopharmacol. 2023 Apr; 117:109879.

170. Toldi G, Szalay B, Beko G, Kovacs L, Vasarhelyi B, Balog A. Plasma soluble urokinase plasminogen activator receptor (suPAR) levels in ankylosing spondylitis. Joint Bone Spine. 2013 Jan; 80(1):96-98.

171. Ozgocmen S, Godekmerdan A, Ozkurt-Zengin F. Acute-phase response, clinical measures and disease activity in ankylosing spondylitis. Joint Bone Spine. 2007 May; 74(3):249-253.

172. Mlcoch T, Sedova L, Stolfa J, Urbanova M, Suchy D, Smrzova A, et al. Mapping the relationship between clinical and quality-of-life outcomes in patients with ankylosing spondylitis. Expert Rev Pharmacoecon Outcomes Res. 2017 Apr; 17(2):203-211.

173. Kang KY, Kwok SK, Ju JH, Park KS, Park SH, Hong YS. The predictors of development of new syndesmophytes in female patients with ankylosing spondylitis. Scand J Rheumatol. 2015; 44(2):125-128.

174. Ho KJ, Chen PQ, Chang CY, Lu FJ. The oxidative metabolism of circulating phagocytes in ankylosing spondylitis: determination by whole blood chemiluminescence. Ann Rheum Dis. 2000 May; 59(5):338-341.

175. Wang F, Yan CG, Xiang HY, Xing T, Wang NS. The significance of platelet activation in ankylosing spondylitis. Clin Rheumatol. 2008 Jun; 27(6):767-769.

176. Kang KY, Kim IJ, Jung SM, Kwok SK, Ju JH, Park KS, et al. Incidence and predictors of morphometric vertebral fractures in patients with ankylosing spondylitis. Arthritis Res Ther. 2014 Jun 16; 16(3):R124.

177. Komsalova LY, Martinez Salinas MP, Jimenez JFG. Predictive values of inflammatory back pain, positive HLA B27 antigen and acute and chronic magnetic resonance changes in early diagnosis of Spondyloarthritis. A study of 133 patients. PLoS One. 2020; 15(12):e0244184.

178. Naziroglu M, Akkus S, Celik H. Levels of lipid peroxidation and antioxidant vitamins in plasma and erythrocytes of patients with ankylosing spondylitis. Clin Biochem. 2011 Dec; 44(17-18):1412-1415.

179. Zwolak R, Suszek D, Graca A, Mazurek M, Majdan M. Reasons for diagnostic delays of axial spondyloarthritis. Wiad Lek. 2019; 72(9 cz 1):1607-1610.

180. Seng JJB, Kwan YH, Low LL, Thumboo J, Fong WSW. Role of neutrophil to lymphocyte ratio (NLR), platelet to lymphocyte ratio (PLR) and mean platelet volume (MPV) in assessing disease control in Asian patients with axial spondyloarthritis. Biomarkers. 2018 May-Jun; 23(4):335-338.

181. Sahli H, Bachali A, Tekaya R, Mahmoud I, Sedki Y, Saidane O, et al. Involvement of foot in patients with spondyloarthritis: Prevalence and clinical features. Foot Ankle Surg. 2019 Apr; 25(2):226-230.

182. Hirano F, Landewe RBM, van Gaalen FA, van der Heijde D, Gaujoux-Viala C, Ramiro S. Determinants of the Physician Global Assessment of Disease Activity and Influence of Contextual Factors in Early Axial Spondyloarthritis. Arthritis Care Res (Hoboken). 2022 Feb; 74(2):268-273.

183. Poddubnyy D, Rudwaleit M, Haibel H, Listing J, Marker-Hermann E, Zeidler H, et al. Rates and predictors of radiographic sacroiliitis progression over 2 years in patients with axial spondyloarthritis. Ann Rheum Dis. 2011 Aug; 70(8):1369-1374.

184. Iervolino S, Di Minno MN, Peluso R, Lofrano M, Russolillo A, Di Minno G, et al. Predictors of early minimal disease activity in patients with psoriatic arthritis treated with tumor necrosis factor-alpha blockers. J Rheumatol. 2012 Mar; 39(3):568-573.

185. Londono J, Romero-Sanchez MC, Torres VG, Bautista WA, Fernandez DJ, Quiroga JdA, et al. The association between serum levels of potential biomarkers with the presence of factors related to the clinical activity and poor prognosis in spondyloarthritis. Rev Bras Reumatol. 2012 Aug; 52(4):536-544.

186. Li T, Liu Y, Sheng R, Yin J, Wu X, Xu H. Correlation Between Chronic Pain Acceptance and Clinical Variables in Ankylosing Spondylitis and Its Prediction Role for Biologics Treatment. Frontiers in Medicine. 2020; 7.

187. Bansal N, Duggal L, Jain N. Validity of Simplified Ankylosing Spondylitis Disease Activity Scores (SASDAS) in Indian Ankylosing Spondylitis Patients. J Clin Diagn Res. 2017 Sep; 11(9):OC06-OC09.

188. Navarini L, Caso F, Costa L, Currado D, Stola L, Perrotta F, et al. Cardiovascular Risk Prediction in Ankylosing Spondylitis: From Traditional Scores to Machine Learning Assessment. Rheumatol Ther. 2020 Dec; 7(4):867-882.

189. Sebastian A, Wojtala P, Lubinski L, Mimier M, Chlebicki A, Wiland P. Disease activity in axial spondyloarthritis after discontinuation of TNF inhibitors therapy. Reumatologia. 2017; 55(4):157-162.

190. Wendling D, Guillot X, Gossec L, Prati C, Saraux A, Dougados M. Remission is related to CRP and smoking in early axial spondyloarthritis. The DESIR cohort. Joint Bone Spine. 2017 Jul; 84(4):473-476.

191. Zong HX, Xu SQ, Wang JX, Chu YR, Chen KM, Wang C, et al. Presence of subclinical inflammation in axial spondyloarthritis patients with NSAID/anti-TNF-alpha drug-induced clinical remission. Clin Rheumatol. 2022 May; 41(5):1403-1412.

192. Kim SH, Kim KH, Kim MY, Hong YS, Kang KY. A 2-year longitudinal study of bone health in adolescent patients with axial spondyloarthritis. Arch Osteoporos. 2021 Jan 8; 16(1):12.

193. Braga MV, de Oliveira SC, Vasconcelos AHC, Lopes JR, de Macedo Filho CL, Ramos LMA, et al. Prevalence of sacroiliitis and acute and structural changes on MRI in patients with psoriatic arthritis. Sci Rep. 2020 Jul 14; 10(1):11580.

194. Chen HA, Chen CH, Liao HT, Lin YJ, Chen PC, Chen WS, et al. Factors associated with radiographic spinal involvement and hip involvement in ankylosing spondylitis. Semin Arthritis Rheum. 2011 Jun; 40(6):552-558.

195. Yildirim K, Erdal A, Karatay S, Melikoglu MA, Ugur M, Senel K. Relationship between some acute phase reactants and the Bath Ankylosing Spondylitis Disease Activity Index in patients with ankylosing spondylitis. South Med J. 2004 Apr; 97(4):350-353.

196. Webers C, Essers I, Ramiro S, Stolwijk C, Landewe R, van der Heijde D, et al. Gender-attributable differences in outcome of ankylosing spondylitis: long-term results from the Outcome in Ankylosing Spondylitis International Study. Rheumatology (Oxford). 2016 Mar; 55(3):419-428.

197. Syrbe U, Callhoff J, Conrad K, Poddubnyy D, Haibel H, Junker S, et al. Serum adipokine levels in patients with ankylosing spondylitis and their relationship to clinical parameters and radiographic spinal progression. Arthritis Rheumatol. 2015 Mar; 67(3):678-685.

198. Kang KY, Kim IJ, Yoon MA, Hong YS, Park SH, Ju JH. Fat Metaplasia on Sacroiliac Joint Magnetic Resonance Imaging at Baseline Is Associated with Spinal Radiographic Progression in Patients with Axial Spondyloarthritis. PLoS One. 2015; 10(8):e0135206.

199. Poddubnyy D, Protopopov M, Haibel H, Braun J, Rudwaleit M, Sieper J. High disease activity according to the Ankylosing Spondylitis Disease Activity Score is associated with accelerated radiographic spinal progression in patients with early axial spondyloarthritis: results from the GErman SPondyloarthritis Inception Cohort. Ann Rheum Dis. 2016 Dec; 75(12):2114-2118.

200. Kim TJ, Shin JH, Kim S, Sung IH, Lee S, Song Y, et al. Radiographic progression in patients with ankylosing spondylitis according to tumor necrosis factor blocker exposure: Observation Study of Korean Spondyloarthropathy Registry (OSKAR) data. Joint Bone Spine. 2016 Oct; 83(5):569-572.

201. Sohn DH, Jeong H, Roh JS, Lee HN, Kim E, Koh JH, et al. Serum CCL11 level is associated with radiographic spinal damage in patients with ankylosing spondylitis. Rheumatol Int. 2018 Aug; 38(8):1455-1464.

202. Deminger A, Klingberg E, Geijer M, Gothlin J, Hedberg M, Rehnberg E, et al. A five-year prospective study of spinal radiographic progression and its predictors in men and women with ankylosing spondylitis. Arthritis Res Ther. 2018 Aug 3; 20(1):162.

203. Pedersen SJ, Weber U, Said-Nahal R, Sorensen IJ, Loft AG, Kollerup G, et al. Structural progression rate decreases over time on serial radiography and magnetic resonance imaging of sacroiliac joints and spine in a five-year follow-up study of patients with ankylosing spondylitis treated with tumour necrosis factor inhibitor. Scand J Rheumatol. 2019 May; 48(3):185-197.

204. Jeong H, Bea EK, Lee J, Koh EM, Cha HS. Body mass index and estrogen predict radiographic progression in the spine in ankylosing spondylitis. Joint Bone Spine. 2015 Dec; 82(6):473-474.

205. Huerta-Sil G, Casasola-Vargas JC, Londono JD, Rivas-Ruiz R, Chavez J, Pacheco-Tena C, et al. Low grade radiographic sacroiliitis as prognostic factor in patients with undifferentiated spondyloarthritis fulfilling diagnostic criteria for ankylosing spondylitis throughout follow up. Ann Rheum Dis. 2006 May; 65(5):642-646.

206. Braun J, Baraliakos X, Hermann KG, Xu S, Hsu B. Serum C-reactive Protein Levels Demonstrate Predictive Value for Radiographic and Magnetic Resonance Imaging Outcomes in Patients with Active Ankylosing Spondylitis Treated with Golimumab. J Rheumatol. 2016 Sep; 43(9):1704-1712.

207. Poddubnyy D, Haibel H, Listing J, Marker-Hermann E, Zeidler H, Braun J, et al. Baseline radiographic damage, elevated acute-phase reactant levels, and cigarette smoking status predict spinal radiographic progression in early axial spondylarthritis. Arthritis Rheum. 2012 May; 64(5):1388-1398.

208. Kim MJ, Lee EB, Song YW, Park JK. Profile of common inflammatory markers in treatment-naive patients with systemic rheumatic diseases. Clin Rheumatol. 2020 Oct; 39(10):2899-2906.

209. Jung SY, Park MC, Park YB, Lee SK. Serum amyloid a as a useful indicator of disease activity in patients with ankylosing spondylitis. Yonsei Med J. 2007 Apr 30; 48(2):218-224.

210. Borman P, Bodur H, Bingol N, Bingol S, Bostan EE. Bone mineral density and bone turnover markers in a group of male ankylosing spondylitis patients: relationship to disease activity. J Clin Rheumatol. 2001 Oct; 7(5):315-321.

211. Hussein A, Stein J, Ehrich JH. C-reactive protein in the assessment of disease activity in juvenile rheumatoid arthritis and juvenile spondyloarthritis. Scand J Rheumatol. 1987; 16(2):101-105.

212. Sheehan NJ, Slavin BM, Donovan MP, Mount JN, Mathews JA. Lack of correlation between clinical disease activity and erythrocyte sedimentation rate, acute phase proteins or protease inhibitors in ankylosing spondylitis. Br J Rheumatol. 1986 May; 25(2):171-174.

213. Wang Z, Hu Y, Long HB. To Compare the Early Efficacy of Infliximab and Adalimumab for the Treatment of Ankylosing Spondylitis and their Impacts on Inflammatory Markers. Indian Journal of Pharmaceutical Sciences. 2022; 84(S1).

214. Alegre-Sancho JJ, Juanola X, Rodriguez-Heredia JM, Manero J, Villa-Blanco I, Laiz A, et al. Effectiveness and persistence of golimumab as a second biological drug in patients with spondyloarthritis: A retrospective study. Medicine (Baltimore). 2021 Apr 2; 100(13):e25223.

215. Zhang K, Zheng Y, Han Q, Liu Y, Wang W, Ding J, et al. The Clinical and MRI Effect of TNF-alpha Inhibitors in Spondyloarthritis Patients With Hip Involvement: A Real-World Observational Clinical Study. Front Immunol. 2021; 12:740980.

216. You Y, Cai M, Lin J, Liu L, Chen C, Wang Y, et al. Efficacy of needle-knife combined with etanercept treatment regarding disease activity and hip joint function in ankylosing spondylitis patients with hip joint involvement: A randomized controlled study. Medicine (Baltimore). 2020 May; 99(19):e20019.

217. Xu Y, Jiang W, Zhang H. Association between C-reactive protein gene variant and treatment efficacy of etanercept in ankylosing spondylitis patients receiving hip arthroplasty. J Clin Lab Anal. 2020 Aug; 34(8):e23343.

218. Dong Y, Guo J, Bi L. Baseline Interleukin-6 and Erythrocyte Sedimentation Rate Can Predict Clinical Response of TNF Inhibitor Treatment in Patients with Ankylosing Spondylitis. Ann Clin Lab Sci. 2019 Sep; 49(5):611-618.

219. Gentileschi S, Vitale A, Rigante D, Lopalco G, Emmi G, Orlando I, et al. Prompt Clinical Response to Secukinumab in Patients with Axial Spondyloarthritis: Real Life Observational Data from Three Italian Referral Centers. Isr Med Assoc J. 2018 Jul; 20(7):438-441.

220. Wei JC, Tsai WC, Citera G, Kotak S, Llamado L. Efficacy and safety of etanercept in patients from Latin America, Central Europe and Asia with early non-radiographic axial spondyloarthritis. Int J Rheum Dis. 2018 Jul; 21(7):1443-1451.

221. Lubrano E, Massimo Perrotta F, Manara M, D'Angelo S, Addimanda O, Ramonda R, et al. Predictors of Loss of Remission and Disease Flares in Patients with Axial Spondyloarthritis Receiving Antitumor Necrosis Factor Treatment: A Retrospective Study. J Rheumatol. 2016 Aug; 43(8):1541-1546.

222. Mok CC, Li OC, Chan KL, Ho LY, Hui PK. Effect of golimumab and pamidronate on clinical efficacy and MRI inflammation in axial spondyloarthritis: a 48-week open randomized trial. Scand J Rheumatol. 2015; 44(6):480-486.

223. Kneepkens EL, Wei JC, Nurmohamed MT, Yeo KJ, Chen CY, van der Horst-Bruinsma IE, et al. Immunogenicity, adalimumab levels and clinical response in ankylosing spondylitis patients during 24 weeks of follow-up. Ann Rheum Dis. 2015 Feb; 74(2):396-401.

224. Korkosz M, Gasowski J, Leszczynski P, Pawlak-Bus K, Jeka S, Siedlar M, et al. Effect of tumour necrosis factor-alpha inhibitor on serum level of dickkopf-1 protein and bone morphogenetic protein-7 in ankylosing spondylitis patients with high disease activity. Scand J Rheumatol. 2014; 43(1):43-48.

225. Paramarta JE, De Rycke L, Heijda TF, Ambarus CA, Vos K, Dinant HJ, et al. Efficacy and safety of adalimumab for the treatment of peripheral arthritis in spondyloarthritis patients without ankylosing spondylitis or psoriatic arthritis. Ann Rheum Dis. 2013 Nov; 72(11):1793-1799.

226. Sandhya P, Danda D, Mathew J, Gattani A. Outcome of patients with seronegative spondyloarthritis continuing sulphasalazine and methotrexate after a short course of infliximab therapy--experience from a tertiary care teaching hospital in South India. Clin Rheumatol. 2011 Jul; 30(7):997-1001.

227. Kim HR, Hong YS, Park SH, Ju JH, Kang KY. Low bone mineral density predicts the formation of new syndesmophytes in patients with axial spondyloarthritis. Arthritis Res Ther. 2018 Oct 16; 20(1):231.

228. Haroon N, Maksymowych WP, Rahman P, Tsui FW, O'Shea FD, Inman RD. Radiographic severity of ankylosing spondylitis is associated with polymorphism of the large multifunctional peptidase 2 gene in the Spondyloarthritis Research Consortium of Canada cohort. Arthritis Rheum. 2012 Apr; 64(4):1119-1126.

229. van Eijk IC, de Vries MK, Levels JH, Peters MJ, Huizer EE, Dijkmans BA, et al. Improvement of lipid profile is accompanied by atheroprotective alterations in high-density lipoprotein composition upon tumor necrosis factor blockade: a prospective cohort study in ankylosing spondylitis. Arthritis Rheum. 2009 May; 60(5):1324-1330.

230. Lange U, Boss B, Teichmann J, Klor HU, Neeck G. Serum amyloid A--an indicator of inflammation in ankylosing spondylitis. Rheumatol Int. 2000; 19(4):119-122.

231. Ostensen M, Marhaug G, Husby G. Amyloid-related serum protein (SAA) during and after pregnancy in healthy women and women with rheumatic disease. Acta Pathol Microbiol Immunol Scand C. 1985 Feb; 93(1):1-5.

232. Hu QL, Fu S, Huang R, Zhang L, Wu LF, Lv YJ. The Value of Serum Amyloid A in the Diagnosis and Management of Ankylosing Spondylitis. Int J Gen Med. 2021; 14:2715-2719.

233. Liu S, Ji W, Lu J, Tang X, Guo Y, Ji M, et al. Discovery of Potential Serum Protein Biomarkers in Ankylosing Spondylitis Using Tandem Mass Tag-Based Quantitative Proteomics. J Proteome Res. 2020 Feb 7; 19(2):864-872.

234. Rademacher J, Tietz LM, Le L, Hartl A, Hermann KA, Sieper J, et al. Added value of biomarkers compared with clinical parameters for the prediction of radiographic spinal progression in axial spondyloarthritis. Rheumatology (Oxford). 2019 Sep 1; 58(9):1556-1564.

235. Londono J, Romero-Sanchez MC, Torres VG, Bautista WA, Fernandez DJ, Quiroga Jde A, et al. The association between serum levels of potential biomarkers with the presence of factors related to the clinical activity and poor prognosis in spondyloarthritis. Rev Bras Reumatol. 2012 Aug; 52(4):536-544.

236. Toussirot E, Streit G, Nguyen NU, Dumoulin G, Le Huede G, Saas P, et al. Adipose tissue, serum adipokines, and ghrelin in patients with ankylosing spondylitis. Metabolism. 2007 Oct; 56(10):1383-1389.

237. Elolemy GG, Ganeb SS, Ghanima ATA, Abdelgwad ER. Influence of adipocytokines and IL-6 on ankylosing spondylitis disease activity and functional status. The Egyptian Rheumatologist. 2013; 35(2):65-70.

238. Park MC, Lee SW, Choi ST, Park YB, Lee SK. Serum leptin levels correlate with interleukin-6 levels and disease activity in patients with ankylosing spondylitis. Scand J Rheumatol. 2007 Mar-Apr; 36(2):101-106.

239. Miranda-Filloy JA, López-Mejias R, Genre F, Carnero-López B, Ochoa R, Diaz de Terán T, et al. Adiponectin and resistin serum levels in non-diabetic ankylosing spondylitis patients undergoing TNF-α antagonist therapy. Clin Exp Rheumatol. 2013 May-Jun; 31(3):365-371.

240. Gonzalez-Lopez L, Fajardo-Robledo NS, Miriam Saldana-Cruz A, Moreno-Sandoval IV, Bonilla-Lara D, Zavaleta-Muniz S, et al. Association of adipokines, interleukin-6, and tumor necrosis factor-alpha concentrations with clinical characteristics and presence of spinal syndesmophytes in patients with ankylosing spondylitis: A cross-sectional study. J Int Med Res. 2017 Jun; 45(3):1024-1035.

241. Pishgahi A, Abolhasan R, Danaii S, Amanifar B, Soltani-Zangbar MS, Zamani M, et al. Immunological and oxidative stress biomarkers in Ankylosing Spondylitis patients with or without metabolic syndrome. Cytokine. 2020 Apr; 128:155002.

242. Xie J, Yang M, Yu H, Xu K, Wan X, Wang J, et al. No Evidence to Support a Causal Relationship between Circulating Adiponectin Levels and Ankylosing Spondylitis: A Bidirectional Two-Sample Mendelian Randomization Study. Genes (Basel). 2022 Dec 2; 13(12).

243. Park MC, Chung SJ, Park YB, Lee SK. Pro-inflammatory effect of leptin on peripheral blood mononuclear cells of patients with ankylosing spondylitis. Joint Bone Spine. 2009 Mar; 76(2):170-175.

244. Kocabas H, Kocabas V, Buyukbas S, Melikoglu MA, Sezer I, Butun B. The serum levels of resistin in ankylosing spondylitis patients: a pilot study. Rheumatol Int. 2012 Mar; 32(3):699-702.

245. Güler M, Çapkin E, Karkucak M, Aydin T, Türkyilmaz AK, Karaca A, et al. Plasma leptin status and the relationship between different medical treatments used in ankylosing spondylitis. Nobel Medicus. 2013; 9(2):109-113.

246. Kononoff A, Vuolteenaho K, Hamalainen M, Kautiainen H, Elfving P, Savolainen E, et al. Metabolic Syndrome, Disease Activity, and Adipokines in Patients With Newly Diagnosed Inflammatory Joint Diseases. J Clin Rheumatol. 2021 Dec 1; 27(8):e349-e356.

247. Sari I, Demir T, Kozaci LD, Akar S, Kavak T, Birlik M, et al. Body composition, insulin, and leptin levels in patients with ankylosing spondylitis. Clin Rheumatol. 2007 Sep; 26(9):1427-1432.

248. Wang HH, Wang QF. Low vaspin levels are related to endothelial dysfunction in patients with ankylosing spondylitis. Braz J Med Biol Res. 2016 Jul 4; 49(7).

249. Genre F, Rueda-Gotor J, Remuzgo-Martinez S, Pulito-Cueto V, Corrales A, Mijares V, et al. Omentin: a biomarker of cardiovascular risk in individuals with axial spondyloarthritis. Sci Rep. 2020 Jun 15; 10(1):9636.

250. Toussirot E, Grandclement E, Gaugler B, Michel F, Wendling D, Saas P, et al. Serum adipokines and adipose tissue distribution in rheumatoid arthritis and ankylosing spondylitis. A comparative study. Front Immunol. 2013; 4:453.

251. Kim KJ, Kim JY, Park SJ, Yoon H, Yoon CH, Kim WU, et al. Serum leptin levels are associated with the presence of syndesmophytes in male patients with ankylosing spondylitis. Clin Rheumatol. 2012 Aug; 31(8):1231-1238.

252. Hartl A, Sieper J, Syrbe U, Listing J, Hermann KG, Rudwaleit M, et al. Serum levels of leptin and high molecular weight adiponectin are inversely associated with radiographic spinal progression in patients with ankylosing spondylitis: results from the ENRADAS trial. Arthritis Res Ther. 2017 Jun 15; 19(1):140.

253. Toussirot E, Mourot L, Dehecq B, Wendling D, Grandclement E, Dumoulin G, et al. TNFalpha blockade for inflammatory rheumatic diseases is associated with a significant gain in android fat mass and has varying effects on adipokines: a 2-year prospective study. Eur J Nutr. 2014 Apr; 53(3):951-961.

254. Rueda-Gotor J, Lopez-Mejias R, Remuzgo-Martinez S, Pulito-Cueto V, Corrales A, Lera-Gomez L, et al. Vaspin in atherosclerotic disease and cardiovascular risk in axial spondyloarthritis: a genetic and serological study. Arthritis Res Ther. 2021 Apr 13; 23(1):111.

255. Rademacher J, Siderius M, Gellert L, Wink FR, Verba M, Maas F, et al. Baseline serum biomarkers of inflammation, bone turnover and adipokines predict spinal radiographic progression in ankylosing spondylitis patients on TNF inhibitor therapy. Semin Arthritis Rheum. 2022 Apr; 53:151974.

256. Czokolyova M, Pusztai A, Vegh E, Horvath A, Szentpeteri A, Hamar A, et al. Changes of Metabolic Biomarker Levels upon One-Year Anti-TNF-alpha Therapy in Rheumatoid Arthritis and Ankylosing Spondylitis: Associations with Vascular Pathophysiology. Biomolecules. 2021 Oct 18; 11(10).

257. Derdemezis CS, Filippatos TD, Voulgari PV, Tselepis AD, Drosos AA, Kiortsis DN. Leptin and adiponectin levels in patients with ankylosing spondylitis. The effect of infliximab treatment. Clin Exp Rheumatol. 2010 Nov-Dec; 28(6):880-883.

258. Miranda-Filloy JA, López-Mejias R, Genre F, Carnero-López B, Ochoa R, Diaz de Terán T, et al. Leptin and visfatin serum levels in non-diabetic ankylosing spondylitis patients undergoing TNF-α antagonist therapy. Clin Exp Rheumatol. 2013 Jul-Aug; 31(4):538-545.

259. Hulejova H, Levitova A, Kuklova M, Stochl J, Haluzik M, Pavelka K, et al. No effect of physiotherapy on the serum levels of adipocytokines in patients with ankylosing spondylitis. Clin Rheumatol. 2012 Jan; 31(1):67-71.

260. Inman RD, Baraliakos X, Hermann KA, Braun J, Deodhar A, van der Heijde D, et al. Serum biomarkers and changes in clinical/MRI evidence of golimumab-treated patients with ankylosing spondylitis: results of the randomized, placebo-controlled GO-RAISE study. Arthritis Res Ther. 2016 Dec 28; 18(1):304.

261. Wagner C, Visvanathan S, Braun J, van der Heijde D, Deodhar A, Hsu B, et al. Serum markers associated with clinical improvement in patients with ankylosing spondylitis treated with golimumab. Ann Rheum Dis. 2012 May; 71(5):674-680.

262. Goldberger C, Dulak J, Duftner C, Weidinger F, Falkenbach A, Schirmer M. Vascular endothelial growth factor (VEGF) in ankylosing spondylitis--a pilot study. Wien Med Wochenschr. 2002; 152(9-10):223-225.

263. Drouart M, Saas P, Billot M, Cedoz JP, Tiberghien P, Wendling D, et al. High serum vascular endothelial growth factor correlates with disease activity of spondylarthropathies. Clin Exp Immunol. 2003 Apr; 132(1):158-162.

264. Wang M, Zhou X, Zhang H, Liu R, Xu N. Associations of the VEGF level, VEGF rs2010963 G/C gene polymorphism and ankylosing spondylitis risk in a Chinese Han population. Immunol Lett. 2016 Nov; 179:56-60.

265. Pedersen SJ, Sorensen IJ, Garnero P, Johansen JS, Madsen OR, Tvede N, et al. ASDAS, BASDAI and different treatment responses and their relation to biomarkers of inflammation, cartilage and bone turnover in patients with axial spondyloarthritis treated with TNFalpha inhibitors. Ann Rheum Dis. 2011 Aug; 70(8):1375-1381.

266. Lin TT, Lu J, Qi CY, Yuan L, Li XL, Xia LP, et al. Elevated serum level of IL-27 and VEGF in patients with ankylosing spondylitis and associate with disease activity. Clin Exp Med. 2015 May; 15(2):227-231.

267. Przepiera-Bedzak H, Fischer K, Brzosko M. Serum VEGF, EGF, basic FGF, and acidic FGF levels and their association with disease activity and extra‑articular symptoms in ankylosing spondylitis. Pol Arch Med Wewn. 2016 Mar 24; 126(4):290-292.

268. Sakellariou GT, Iliopoulos A, Konsta M, Kenanidis E, Potoupnis M, Tsiridis E, et al. Serum levels of Dkk-1, sclerostin and VEGF in patients with ankylosing spondylitis and their association with smoking, and clinical, inflammatory and radiographic parameters. Joint Bone Spine. 2017 May; 84(3):309-315.

269. Torres L, Klingberg E, Nurkkala M, Carlsten H, Forsblad-d'Elia H. Hepatocyte growth factor is a potential biomarker for osteoproliferation and osteoporosis in ankylosing spondylitis. Osteoporos Int. 2019 Feb; 30(2):441-449.

270. Seo JS, Lee SS, Kim SI, Ryu WH, Sa KH, Kim SU, et al. Influence of VEGF gene polymorphisms on the severity of ankylosing spondylitis. Rheumatology (Oxford). 2005 Oct; 44(10):1299-1302.

271. Poddubnyy D, Conrad K, Haibel H, Syrbe U, Appel H, Braun J, et al. Elevated serum level of the vascular endothelial growth factor predicts radiographic spinal progression in patients with axial spondyloarthritis. Ann Rheum Dis. 2014 Dec; 73(12):2137-2143.

272. Braun J, Baraliakos X, Hermann KG, Xu S, Hsu B. Serum Vascular Endothelial Growth Factor Levels Lack Predictive Value in Patients with Active Ankylosing Spondylitis Treated with Golimumab. J Rheumatol. 2016 May; 43(5):901-906.

273. Appel H, Janssen L, Listing J, Heydrich R, Rudwaleit M, Sieper J. Serum levels of biomarkers of bone and cartilage destruction and new bone formation in different cohorts of patients with axial spondyloarthritis with and without tumor necrosis factor-alpha blocker treatment. Arthritis Res Ther. 2008; 10(5):R125.

274. Visvanathan S, Wagner C, Marini JC, Baker D, Gathany T, Han J, et al. Inflammatory biomarkers, disease activity and spinal disease measures in patients with ankylosing spondylitis after treatment with infliximab. Ann Rheum Dis. 2008 Apr; 67(4):511-517.

275. Pedersen SJ, Hetland ML, Sorensen IJ, Ostergaard M, Nielsen HJ, Johansen JS. Circulating levels of interleukin-6, vascular endothelial growth factor, YKL-40, matrix metalloproteinase-3, and total aggrecan in spondyloarthritis patients during 3 years of treatment with TNFalpha inhibitors. Clin Rheumatol. 2010 Nov; 29(11):1301-1309.

276. Tosovsky M, Bradna P, Andrys C, Andrysova K, Cermakova E, Soukup T. The VEGF and BMP-2 levels in patients with ankylosing spondylitis and the relationship to treatment with tumour necrosis factor alpha inhibitors. Acta Medica (Hradec Kralove). 2014; 57(2):56-61.

277. Ma Y, Fan D, Xu S, Deng J, Gao X, Guan S, et al. Calprotectin in spondyloarthritis: A systematic review and meta-analysis. Int Immunopharmacol. 2020 Nov; 88:106948.

278. Ercalik C, Baskaya MC, Ozdem S, Butun B. Investigation of asymptomatic intestinal inflammation in ankylosing spondylitis by fecal calprotectin. Arab J Gastroenterol. 2021 Dec; 22(4):272-277.

279. Genre F, Rueda-Gotor J, Remuzgo-Martinez S, Corrales A, Mijares V, Exposito R, et al. Association of circulating calprotectin with lipid profile in axial spondyloarthritis. Sci Rep. 2018 Sep 13; 8(1):13728.

280. Huang J, Yin Z, Song G, Cui S, Jiang J, Zhang L. Discriminating Value of Calprotectin in Disease Activity and Progression of Nonradiographic Axial Spondyloarthritis and Ankylosing Spondylitis. Dis Markers. 2017; 2017:7574147.

281. Klingberg E, Carlsten H, Hilme E, Hedberg M, Forsblad-d'Elia H. Calprotectin in ankylosing spondylitis--frequently elevated in feces, but normal in serum. Scand J Gastroenterol. 2012 Apr; 47(4):435-444.

282. Oktayoglu P, Bozkurt M, Mete N, Caglayan M, Em S, Nas K. Elevated serum levels of calprotectin (myeloid-related protein 8/14) in patients with ankylosing spondylitis and its association with disease activity and quality of life. J Investig Med. 2014 Aug; 62(6):880-884.

283. Olofsson T, Lindqvist E, Mogard E, Andreasson K, Marsal J, Geijer M, et al. Elevated faecal calprotectin is linked to worse disease status in axial spondyloarthritis: results from the SPARTAKUS cohort. Rheumatology (Oxford). 2019 Jul 1; 58(7):1176-1187.

284. Qian BP, Ji ML, Qiu Y, Wang B, Yu Y, Shi W, et al. Identification of Serum miR-146a and miR-155 as Novel Noninvasive Complementary Biomarkers for Ankylosing Spondylitis. Spine (Phila Pa 1976). 2016 May; 41(9):735-742.

285. Wang M, Wang L, Zhang X, Yang X, Li X, Xia Q, et al. Overexpression of miR-31 in Peripheral Blood Mononuclear Cells (PBMC) from Patients with Ankylosing Spondylitis. Med Sci Monit. 2017 Nov 18; 23:5488-5494.

286. Wang Y, Luo J, Wang X, Yang B, Cui L. MicroRNA-199a-5p Induced Autophagy and Inhibits the Pathogenesis of Ankylosing Spondylitis by Modulating the mTOR Signaling via Directly Targeting Ras Homolog Enriched in Brain (Rheb). Cell Physiol Biochem. 2017; 42(6):2481-2491.

287. Wei C, Zhang H, Wei C, Mao Y. Correlation of the expression of miR-146a in peripheral blood mononuclear cells of patients with ankylosing spondylitis and inflammatory factors. Exp Ther Med. 2017 Nov; 14(5):5027-5031.

288. Yildirim T, Yesilada E, Eren F, Apaydin H, Gulbay G. Assessment of plasma microRNA potentials as a non-invasive biomarker in patients with axial spondyloarthropathy. Eur Rev Med Pharmacol Sci. 2021 Jan; 25(2):620-625.

289. Zhang CL, Li YC, Wu JW, Zhu BL. Expression and function of peripheral blood miRNA16a in patients with ankylosing spondylitis. Eur Rev Med Pharmacol Sci. 2018 Aug; 22(16):5106-5113.

290. Zou YC, Gao YP, Yin HD, Liu G. Serum miR-21 expression correlates with radiographic progression but also low bone mineral density in patients with ankylosing spondylitis: a cross-sectional study. Innate Immun. 2019 Jul; 25(5):314-321.

291. Guo TM, Yan Y, Cao WN, Liu Q, Zhu HY, Yang L, et al. Predictive value of microRNA-132 and its target gene NAG-1 in evaluating therapeutic efficacy of non-steroidal anti-inflammatory drugs treatment in patients with ankylosing spondylitis. Clin Rheumatol. 2018 May; 37(5):1281-1293.

292. Ciechomska M, Bonek K, Merdas M, Zarecki P, Swierkot J, Gluszko P, et al. Changes in MiRNA-5196 Expression as a Potential Biomarker of Anti-TNF-alpha Therapy in Rheumatoid Arthritis and Ankylosing Spondylitis Patients. Arch Immunol Ther Exp (Warsz). 2018 Oct; 66(5):389-397.

293. Fotoh DS, Noreldin RI, Rizk MS, Elsabaawy MM, Esaily HA. miRNA-451a and miRNA-125a Expression Levels in Ankylosing Spondylitis: Impact on Disease Diagnosis, Prognosis, and Outcomes. J Immunol Res. 2020; 2020:2180913.

294. Lv Q, Li Q, Zhang P, Jiang Y, Wang X, Wei Q, et al. Disorders of MicroRNAs in Peripheral Blood Mononuclear Cells: As Novel Biomarkers of Ankylosing Spondylitis and Provocative Therapeutic Targets. Biomed Res Int. 2015; 2015:504208.

295. Prajzlerova K, Komarc M, Forejtova S, Pavelka K, Vencovsky J, Senolt L, et al. Circulating miR-145 as a marker of therapeutic response to anti-TNF therapy in patients with ankylosing spondylitis. Physiol Res. 2021 Apr 30; 70(2):255-264.

296. Liu Z, Huang F, Luo G, Wang Y, Du R, Sun W, et al. miR-214 stimulated by IL-17A regulates bone loss in patients with ankylosing spondylitis. Rheumatology (Oxford). 2020 May 1; 59(5):1159-1169.

297. Ni WJ, Leng XM. Down-regulated miR-495 can target programmed cell death 10 in ankylosing spondylitis. Mol Med. 2020 May 25; 26(1):50.

298. Perez-Sanchez C, Font-Ugalde P, Ruiz-Limon P, Lopez-Pedrera C, Castro-Villegas MC, Abalos-Aguilera MC, et al. Circulating microRNAs as potential biomarkers of disease activity and structural damage in ankylosing spondylitis patients. Hum Mol Genet. 2018 Mar 1; 27(5):875-890.

299. Prajzlerova K, Grobelna K, Husakova M, Forejtova S, Jungel A, Gay S, et al. Association between circulating miRNAs and spinal involvement in patients with axial spondyloarthritis. PLoS One. 2017; 12(9):e0185323.

300. Reyes-Loyola P, Rodriguez-Henriquez P, Ballinas-Verdugo MA, Amezcua-Castillo LM, Juarez-Vicuna Y, Jimenez-Rojas V, et al. Plasma let-7i, miR-16, and miR-221 levels as candidate biomarkers for the assessment of ankylosing spondylitis in Mexican patients naive to anti-TNF therapy. Clin Rheumatol. 2019 May; 38(5):1367-1373.

301. Tan H, Ren R, Zhang J, Huang Z, Niu Q, Yang B. Analysis of inflammation-related microRNA expression in patients with ankylosing spondylitis. Immunol Res. 2022 Feb; 70(1):23-32.

302. Li X, Lv Q, Tu L, Zhao M, Zhang P, Li Q, et al. Aberrant expression of microRNAs in peripheral blood mononuclear cells as candidate biomarkers in patients with axial spondyloarthritis. Int J Rheum Dis. 2019 Jul; 22(7):1188-1195.

303. Huang CH, Wei JC, Chang WC, Chiou SY, Chou CH, Lin YJ, et al. Higher expression of whole blood microRNA-21 in patients with ankylosing spondylitis associated with programmed cell death 4 mRNA expression and collagen cross-linked C-telopeptide concentration. J Rheumatol. 2014 Jun; 41(6):1104-1111.

304. Lan X, Ma H, Zhang Z, Ye D, Min J, Cai F, et al. Downregulation of lncRNA TUG1 is involved in ankylosing spondylitis and is related to disease activity and course of treatment. Biosci Trends. 2018 Sep 19; 12(4):389-394.

305. Zhong H, Zhong M. LINC00311 is overexpressed in ankylosing spondylitis and predict treatment outcomes and recurrence. BMC Musculoskelet Disord. 2019 Jun 7; 20(1):278.

306. Han D, Ouyang G, Pan P, Yuan Y. Upregulated lncRNA-NEF predicts recurrence and poor treatment outcomes of ankylosing spondylitis. Immun Inflamm Dis. 2022 Aug; 10(8):e627.

307. Wang JX, Zhao X, Xu SQ. Screening Key lncRNAs of Ankylosing Spondylitis Using Bioinformatics Analysis. J Inflamm Res. 2022; 15:6087-6096.

308. Wang T, Meng S, Chen P, Wei L, Liu C, Tang D, et al. Comprehensive analysis of differentially expressed mRNA and circRNA in Ankylosing spondylitis patients' platelets. Exp Cell Res. 2021 Dec 1; 409(1):112895.

309. Zou YC, Wu J, Zhao C, Luo ZR. Analysis of circular RNA expression profile of pathological bone formation in ankylosing spondylitis. Int J Rheum Dis. 2023 Jul; 26(7):1403-1406.

310. Tang YP, Zhang QB, Dai F, Liao X, Dong ZR, Yi T, et al. Circular RNAs in peripheral blood mononuclear cells from ankylosing spondylitis. Chin Med J (Engl). 2021 Oct 19; 134(21):2573-2582.

311. Luo Q, Fu B, Zhang L, Guo Y, Huang Z, Li J. Expression and clinical significance of circular RNA hsa_circ_0079787 in the peripheral blood of patients with axial spondyloarthritis. Mol Med Rep. 2020 Nov; 22(5):4197-4206.

312. Romero-Sanchez C, Tsou HK, Jan MS, Wong RH, Chang IC, Londono J, et al. Serum monocyte chemotactic protein-1 concentrations distinguish patients with ankylosing spondylitis from patients with mechanical low back pain. J Spinal Disord Tech. 2011 May; 24(3):202-207.

313. Gratacos J, Collado A, Pons F, Osaba M, Sanmarti R, Roque M, et al. Significant loss of bone mass in patients with early, active ankylosing spondylitis: a followup study. Arthritis Rheum. 1999 Nov; 42(11):2319-2324.

314. Bal A, Unlu E, Bahar G, Aydog E, Eksioglu E, Yorgancioglu R. Comparison of serum IL-1 beta, sIL-2R, IL-6, and TNF-alpha levels with disease activity parameters in ankylosing spondylitis. Clin Rheumatol. 2007 Feb; 26(2):211-215.

315. Sharma SK, Ahmad S, Sharma SK. Serum IL-6 level as a marker of disease activity in ankylosing spondylitis patients with pure axial involvement. Indian Journal of Rheumatology. 2014; 9(3):115-119.

316. Przepiera-Bedzak H, Fischer K, Brzosko M. Serum IL-6 and IL-23 Levels and Their Correlation with Angiogenic Cytokines and Disease Activity in Ankylosing Spondylitis, Psoriatic Arthritis, and SAPHO Syndrome. Mediators Inflamm. 2015; 2015:785705.

317. Li DH, He CR, Liu FP, Li J, Gao JW, Li Y, et al. Annexin A2, up-regulated by IL-6, promotes the ossification of ligament fibroblasts from ankylosing spondylitis patients. Biomed Pharmacother. 2016 Dec; 84:674-679.

318. Rabelo CF, Baptista TSA, Petersen LE, Bauer ME, Keiserman MW, Staub HL. Serum IL-6 correlates with axial mobility index (Bath Ankylosing Spondylitis Metrology Index) in Brazilian patients with ankylosing spondylitis. Open Access Rheumatol. 2018; 10:21-25.

319. Mattey DL, Packham JC, Nixon NB, Coates L, Creamer P, Hailwood S, et al. Association of cytokine and matrix metalloproteinase profiles with disease activity and function in ankylosing spondylitis. Arthritis Res Ther. 2012 May 28; 14(3):R127.

320. Taylan A, Sari I, Kozaci DL, Yildiz Y, Bilge S, Coker I, et al. Evaluation of various endothelial biomarkers in ankylosing spondylitis. Clin Rheumatol. 2012 Jan; 31(1):23-28.

321. Taylan A, Sari I, Kozaci DL, Yuksel A, Bilge S, Yildiz Y, et al. Evaluation of the T helper 17 axis in ankylosing spondylitis. Rheumatol Int. 2012 Aug; 32(8):2511-2515.

322. Sveaas SH, Berg IJ, Provan SA, Semb AG, Olsen IC, Ueland T, et al. Circulating levels of inflammatory cytokines and cytokine receptors in patients with ankylosing spondylitis: a cross-sectional comparative study. Scand J Rheumatol. 2015; 44(2):118-124.

323. Liu R, Yue Z, Peng X, Wang X, Feng Z, Wan L. Association Between Intercellular Adhesion Molecule-1, -2, -3 Plasma Levels and Disease Activity of Ankylosing Spondylitis in the Chinese Han Population. Spine (Phila Pa 1976). 2016 May; 41(10):E618-624.

324. He D, Zhu Q, Zhou Q, Qi Q, Sun H, Zachariah LM, et al. Correlation of serum MMP3 and other biomarkers with clinical outcomes in patients with ankylosing spondylitis: a pilot study. Clin Rheumatol. 2017 Aug; 36(8):1819-1826.

325. Korkosz M, Czepiel M, Gula Z, Stec M, Weglarczyk K, Rutkowska-Zapala M, et al. Sera of patients with axial spondyloarthritis (axSpA) enhance osteoclastogenic potential of monocytes isolated from healthy individuals. BMC Musculoskelet Disord. 2018 Dec 6; 19(1):434.

326. Falkenbach A, Herold M. In ankylosing spondylitis serum interleukin-6 correlates with the degree of mobility restriction, but not with short-term changes in the variables for mobility. Rheumatol Int. 1998; 18(3):103-106.

327. Falkenbach A, Herold M, Wigand R. Interleukin-6 serum concentration in ankylosing spondylitis: a reliable predictor of disease progression in the subsequent year? Rheumatol Int. 2000; 19(4):149-151.

328. Korczowska I, Przepiera-Bedzak H, Brzosko M, Lacki JK, Trefler J, Hrycaj P. Bone tissue metabolism in men with ankylosing spondylitis. Adv Med Sci. 2011; 56(2):264-269.

329. Brandt J, Haibel H, Cornely D, Golder W, Gonzalez J, Reddig J, et al. Successful treatment of active ankylosing spondylitis with the anti-tumor necrosis factor alpha monoclonal antibody infliximab. Arthritis Rheum. 2000 Jun; 43(6):1346-1352.

330. Tarner IH, Muller-Ladner U, Uhlemann C, Lange U. The effect of mild whole-body hyperthermia on systemic levels of TNF-alpha, IL-1beta, and IL-6 in patients with ankylosing spondylitis. Clin Rheumatol. 2009 Apr; 28(4):397-402.

331. Pedersen SJ, Sorensen IJ, Lambert RG, Hermann KG, Garnero P, Johansen JS, et al. Radiographic progression is associated with resolution of systemic inflammation in patients with axial spondylarthritis treated with tumor necrosis factor alpha inhibitors: a study of radiographic progression, inflammation on magnetic resonance imaging, and circulating biomarkers of inflammation, angiogenesis, and cartilage and bone turnover. Arthritis Rheum. 2011 Dec; 63(12):3789-3800.

332. Capkin E, Karkucak M, Akyuz A, Alver A, Turkyilmaz AK, Zengin E. The relationship between plasma homocysteine level and different treatment modalities in patients with ankylosing spondylitis. Rheumatol Int. 2012 Aug; 32(8):2349-2353.

333. Limon-Camacho L, Vargas-Rojas MI, Vazquez-Mellado J, Casasola-Vargas J, Moctezuma JF, Burgos-Vargas R, et al. In vivo peripheral blood proinflammatory T cells in patients with ankylosing spondylitis. J Rheumatol. 2012 Apr; 39(4):830-835.

334. Schulz M, Dotzlaw H, Neeck G. Ankylosing spondylitis and rheumatoid arthritis: serum levels of TNF-alpha and Its soluble receptors during the course of therapy with etanercept and infliximab. Biomed Res Int. 2014; 2014:675108.

335. Levitova A, Hulejova H, Spiritovic M, Pavelka K, Senolt L, Husakova M. Clinical improvement and reduction in serum calprotectin levels after an intensive exercise programme for patients with ankylosing spondylitis and non-radiographic axial spondyloarthritis. Arthritis Res Ther. 2016 Nov 25; 18(1):275.

336. Zhang Y, Ning C, Zhou H, Yan Y, Liu F, Huang Y. Interleukin-1beta, interleukin-6, and interleukin-17A as indicators reflecting clinical response to celecoxib in ankylosing spondylitis patients. Ir J Med Sci. 2021 May; 190(2):631-638.

337. Eggert M, Seeck U, Semmler M, Maass U, Dietmann S, Schulz M, et al. An evaluation of anti-TNF-alpha-therapy in patients with ankylosing spondylitis: imbalanced activation of NF kappa B subunits in lymphocytes and modulation of serum cortisol concentration. Rheumatol Int. 2007 Jul; 27(9):841-846.

338. Yang B, Xu Y, Liu X, Huang Z, Wang L. IL-23R and IL-17A polymorphisms correlate with susceptibility of ankylosing spondylitis in a Southwest Chinese population. Oncotarget. 2017 Sep 19; 8(41):70310-70316.

339. Aghaei H, Farhadi E, Akhtari M, Shahba S, Mostafaei S, Jamshidi A, et al. Copy number variation of IL17RA gene and its association with the ankylosing spondylitis risk in Iranian patients: a case-control study. BMC Med Genet. 2020 Jul 10; 21(1):147.

340. Chen WS, Chang YS, Lin KC, Lai CC, Wang SH, Hsiao KH, et al. Association of serum interleukin-17 and interleukin-23 levels with disease activity in Chinese patients with ankylosing spondylitis. J Chin Med Assoc. 2012 Jul; 75(7):303-308.

341. Mei Y, Pan F, Gao J, Ge R, Duan Z, Zeng Z, et al. Increased serum IL-17 and IL-23 in the patient with ankylosing spondylitis. Clin Rheumatol. 2011 Feb; 30(2):269-273.

342. Wendling D, Cedoz JP, Racadot E, Dumoulin G. Serum IL-17, BMP-7, and bone turnover markers in patients with ankylosing spondylitis. Joint Bone Spine. 2007 May; 74(3):304-305.

343. Wielinska J, Swierkot J, Kolossa K, Bugaj B, Chaszczewska-Markowska M, Jeka S, et al. Polymorphisms within Genes Coding for IL-17A and F and Their Receptor as Clinical Hallmarks in Ankylosing Spondylitis. Mediators Inflamm. 2021; 2021:3125922.

344. Tan H, Huang S, Wang T. Clinical Significance of Peripheral Blood Th1 and Th17 Cell Content and Serum IL-35 and IL-17 Expression in Patients with Ankylosing Spondylitis. Evid Based Complement Alternat Med. 2022; 2022:6540557.

345. Jansen DT, Hameetman M, van Bergen J, Huizinga TW, van der Heijde D, Toes RE, et al. IL-17-producing CD4+ T cells are increased in early, active axial spondyloarthritis including patients without imaging abnormalities. Rheumatology (Oxford). 2015 Apr; 54(4):728-735.

346. Xueyi L, Lina C, Zhenbiao W, Qing H, Qiang L, Zhu P. Levels of circulating Th17 cells and regulatory T cells in ankylosing spondylitis patients with an inadequate response to anti-TNF-alpha therapy. J Clin Immunol. 2013 Jan; 33(1):151-161.

347. Perpetuo IP, Raposeiro R, Caetano-Lopes J, Vieira-Sousa E, Campanilho-Marques R, Ponte C, et al. Effect of Tumor Necrosis Factor Inhibitor Therapy on Osteoclasts Precursors in Ankylosing Spondylitis. PLoS One. 2015; 10(12):e0144655.

348. Milanez FM, Saad CG, Viana VT, Moraes JC, Perico GV, Sampaio-Barros PD, et al. IL-23/Th17 axis is not influenced by TNF-blocking agents in ankylosing spondylitis patients. Arthritis Res Ther. 2016 Feb 24; 18:52.

349. Du J, Sun J, Wen Z, Wu Z, Li Q, Xia Y, et al. Serum IL-6 and TNF-alpha Levels Are Correlated with Disease Severity in Patients with Ankylosing Spondylitis. Lab Med. 2022 Mar 7; 53(2):149-155.

350. Wen JT, Zhang DH, Fang PF, Li MH, Wang RJ, Li SH. Role of Th1/Th2 cytokines in the diagnosis and prognostic evaluation of ankylosing spondylitis. Genet Mol Res. 2017 Mar 16; 16(1).

351. Dong Y, Li P, Xu T, Bi L. Effective serum level of etanercept biosimilar and effect of antidrug antibodies on drug levels and clinical efficacy in Chinese patients with ankylosing spondylitis. Clin Rheumatol. 2019 Jun; 38(6):1587-1594.

352. An H, Li X, Li F, Gao C, Li X, Luo J. The absolute counts of peripheral T lymphocyte subsets in patient with ankylosing spondylitis and the effect of low-dose interleukin-2. Medicine (Baltimore). 2019 Apr; 98(15):e15094.

353. Appel H, Wu P, Scheer R, Kedor C, Sawitzki B, Thiel A, et al. Synovial and peripheral blood CD4+FoxP3+ T cells in spondyloarthritis. J Rheumatol. 2011 Nov; 38(11):2445-2451.

354. Bautista-Caro MB, Arroyo-Villa I, Castillo-Gallego C, de Miguel E, Peiteado D, Plasencia-Rodríguez C, et al. Decreased frequencies of circulating follicular helper T cell counterparts and plasmablasts in ankylosing spondylitis patients Naïve for TNF blockers. PLoS One. 2014; 9(9):e107086.

355. Bidad K, Salehi E, Jamshidi A, Saboor-Yaraghi AA, Oraei M, Meysamie A, et al. Effect of all-transretinoic acid on Th17 and T regulatory cell subsets in patients with ankylosing spondylitis. J Rheumatol. 2013 Apr; 40(4):476-483.

356. Brand JM, Neustock P, Kruse A, Alvarez-Ossorio L, Schnabel A, Kirchner H. Stimulation of whole blood cultures in patients with ankylosing spondylitis by a mitogen derived from Mycoplasma arthritidis (MAS) and other mitogens. Rheumatol Int. 1997; 16(5):207-211.

357. Cai CS, Xiao P. [Expression of regulatory T cells in the peripheral blood of patients with ankylosing spondylitis.]. Journal of Chinese Practical Diagnosis and Therapy. 2013; 27(12):1192-1194.

358. Cai PW, Lin Y, Dou M, Chen JH, Lin Y. [Expression of CD40-CD40L on peripheral blood lymphocytes of patients with Ankylosing spondylitis.]. Immunological Journal. 2005; 21(06):507-508+513.

359. Cao D, van Vollenhoven R, Klareskog L, Trollmo C, Malmstrom V. CD25brightCD4+ regulatory T cells are enriched in inflamed joints of patients with chronic rheumatic disease. Arthritis Res Ther. 2004; 6(4):R335-346.

360. Chen SZ, Bai JP, Xie YH, You YQ, Su ML, Xu XX, et al. [Expression of transcription factor Th17，Treg and Th1 in peripheral blood from patients with ankylosing spondylitis and its correlation with disease activity.]. Chinese Journal of Immunology. 2013; 29(08):834-838+847.

361. Chen MH, Chen WS, Lee HT, Tsai CY, Chou CT. Inverse correlation of programmed death 1 (PD-1) expression in T cells to the spinal radiologic changes in Taiwanese patients with ankylosing spondylitis. Clin Rheumatol. 2011 Sep; 30(9):1181-1187.

362. Cheng F. [CD4+CD25+regulatory T cells in peripheral blood of patients with ankylosing spondylitis.] [doctoral thesis]: The Second Military Medical University; 2007.

363. Dejaco C, Duftner C, Klauser A, Schirmer M. Altered T-cell subtypes in spondyloarthritis, rheumatoid arthritis and polymyalgia rheumatica. Rheumatol Int. 2010 Jan; 30(3):297-303.

364. Deng L, Chen YP, Sun YL. [Expression of miR⁃138 in peripheral blood mononuclear cells of patients with ankylosing spondylitis and its relationship with Th1/Th2 imbalance.]. Journal of Tropical Medicine. 2019; 19(08):1008-1011+1051.

365. Deng JH, Li ZQ, Zhang R, Li YM. [The Correlation Ananlysis of HLA-B27 Expression and Lymphocytes Subsets, Cytokines in the Patients with Ankylosing Spondylitis.]. International Journal of Laboratory Medicine. 2018; 39(16):1976-1979.

366. Dong Q, Yang DR, Liu H. [The Test of Immune Function and Hemorheologic Changes in HLA-B27 Positive Patients with Ankylosing Spondylitis.]. Chinese Journal of Hemorheology. 2006; 16(02):273-274+324.

367. Duan Z, Gui Y, Li C, Lin J, Gober HJ, Qin J, et al. The immune dysfunction in ankylosing spondylitis patients. Biosci Trends. 2017 Mar 22; 11(1):69-76.

368. Dulic S, Vasarhelyi Z, Bajnok A, Szalay B, Toldi G, Kovacs L, et al. The Impact of Anti-TNF Therapy on CD4+ and CD8+ Cell Subsets in Ankylosing Spondylitis. Pathobiology. 2018; 85(3):201-210.

369. Fattahi MJ, Ahmadi H, Jafarnezhad-Ansariha F, Mortazavi-Jahromi SS, Rehm BHA, Cuzzocrea S, et al. Oral administration effects of beta-d-mannuronic acid (M2000) on Th17 and regulatory T cells in patients with ankylosing spondylitis. Biomed Pharmacother. 2018 Apr; 100:495-500.

370. Förger F, Villiger PM, Ostensen M. Pregnancy in patients with ankylosing spondylitis: do regulatory T cells play a role? Arthritis Rheum. 2009 Feb 15; 61(2):279-283.

371. Gao Y, Song Y, Fan YX, Chen M, Xiao N, Pan LZ, et al. [The alteration of TH17cells and CD4+CD25+FoxP3+ regulatory T cell in patients with ankylosing spondylitis.]. Chinese Journal of Microbiology and Immunology. 2012; 32(04):318-322.

372. Guo L, Hou Q, Kou R. [Application of Combined Detection of T Lymphocyte Subsets and Ferritin in Patients with Ankylosing Spondylitis.]. International Journal of Laboratory Medicine. 2012; 33(12):1436-1437.

373. Hajialilo M, Dolati S, Abdolmohammadi-Vahid S, Ahmadi M, Kamrani A, Eghbal-Fard S, et al. Nanocurcumin: A novel strategy in treating ankylosing spondylitis by modulating Th17 cells frequency and function. J Cell Biochem. 2019 Feb 25; 120(07):12027-12038.

374. Han YX, Zhang SH, Wu JB. [Expressions of B7 and CD28 in Peripheral Blood Lymphocytes of Patients with Ankylosing Spondylitis and their Significance.]. Journal of Wenzhou Medical University. 2006; 36(04):356-358.

375. He YH, Wu XM, Wu LJ. [Analysis of Lymphocyte Subsets in Patients with Ankylosing Spondylitis.]. International Journal of Laboratory Medicine. 2012; 33(02):141-142+145.

376. Hu W, Wang ML, Qiu W, Chen SM, Liu DS. [Preliminary study on immunological indicators of patients with ankylosing spondylitis in suqian.]. Contemporary Medicine. 2019; 25(10):1-3.

377. Hu B, Cheng J, Liu SQ. [Analysis of T cell subsets in peripheral blood in patients with ankylosing spondylitis.]. Modern Medical Journal. 2013; 41(08):543-545.

378. Huang H, Wang YD, Sun YY, Sui WG. [Expression regulatory T cells in peripheral blood of ankylosing spondyligis patients.]. China Tropical Medicine. 2009; 9(10):1992-1993.

379. Huang F, Cai XH, Shi GY, Chen XM, Cheng QL, Dong K, et al. [A study of cellular immune function in patients with ankylosing spondylitis.]. Academic Journal of Chinese PLA Medical School. 1991; 12(02):102-105.

380. Ji W, Li H, Gao F, Chen Y, Zhong L, Wang D. Effects of Tripterygium glycosides on interleukin-17 and CD4(+)CD25(+)CD127(low) regulatory T-cell expression in the peripheral blood of patients with ankylosing spondylitis. Biomed Rep. 2014 Jul; 2(4):517-520.

381. Kenna TJ, Davidson SI, Duan R, Bradbury LA, McFarlane J, Smith M, et al. Enrichment of circulating interleukin-17-secreting interleukin-23 receptor-positive γ/δ T cells in patients with active ankylosing spondylitis. Arthritis Rheum. 2012 May; 64(5):1420-1429.

382. Kim TJ, Lee SJ, Cho YN, Park SC, Jin HM, Kim MJ, et al. Immune cells and bone formation in ankylosing spondylitis. Clin Exp Rheumatol. 2012 Jul-Aug; 30(4):469-475.

383. Klasen C, Meyer A, Wittekind PS, Waque I, Nabhani S, Kofler DM. Prostaglandin receptor EP4 expression by Th17 cells is associated with high disease activity in ankylosing spondylitis. Arthritis Res Ther. 2019 Jun 28; 21(1):159.

384. Li WQ. [Expression of miＲ-155 in peripheral blood of patients with ankylosing spondylitis and its relationship with Th17 /Treg balance.]. Journal of Shanxi Medical University. 2019; 50(02):235-240.

385. Xueyi L, Lina C, Zhenbiao W, Qing H, Qiang L, Zhu P. Levels of circulating Th17 cells and regulatory T cells in ankylosing spondylitis patients with an inadequate response to anti-TNF-α therapy. J Clin Immunol. 2013 Jan; 33(1):151-161.

386. Li JX, Zhang LY, Huo YH, Li XF. [Effect of Methylprednisolone on the Th1/Th2 Balance and Cytokines in Patients with Refractong Ankylosing Spondylitis.]. Chinese Journal of Allergy and Clinical Immunology. 2009; 3(01):28-33.

387. Li HX, Sun GR, Cao YX, Wang JB. [Expression and significance of CD8+CD28-T cells in the peripheral blood of patients with AS.]. Chinese Journal of Rheumatology. 2008; 12(05):333-335+361.

388. Liao HT, Lin YF, Tsai CY, Chou CT. Regulatory T cells in ankylosing spondylitis and the response after adalimumab treatment. Joint Bone Spine. 2015 Dec; 82(6):423-427.

389. Limón-Camacho L, Vargas-Rojas MI, Vázquez-Mellado J, Casasola-Vargas J, Moctezuma JF, Burgos-Vargas R, et al. In vivo peripheral blood proinflammatory T cells in patients with ankylosing spondylitis. J Rheumatol. 2012 Apr; 39(4):830-835.

390. Lin Q, Lin ZM, Gu JR, Huang F, Li TW, Wei QJ, et al. [Changes of T lymphocyte subsets and expression of costimulatory molecule CD154 on T-cells in peripheral blood from patients with ankylosing spondylitis.]. Chinese Journal of Rheumatology. 2008; 12(05):309-313.

391. Lin Q, Gu JR, Li TW, Zhang FC, Lin ZM, Liao ZT, et al. Value of the peripheral blood B-cells subsets in patients with ankylosing spondylitis. Chin Med J (Engl). 2009 Aug 5; 122(15):1784-1789.

392. Liu EC, Feng YX. [Relationship of the Balance between Leptin and Th17 and Th1 with Ankylosing Spondycitis Patients.]. Medical Recapitulate. 2017; 23(01):187-189.

393. Liu L, Liu J, Wan L. [The changes of platelet parameters，BTLA and Treg in peripheral blood in patients with ankylosing spondylitis.]. Chinese Journal of Clinical Healthcare. 2016; 19(01):8-11.

394. Liu J, Wang SH, Wan L, Zhang JS, Yang J, Zong RK, et al. [Changes of regulatory T cells in peripheral blood in ankylosing spondylitis patients and the influence of chinese medicine spleen-strengthening unit therapy.]. Chinese Journal of Clinical Healthcare. 2012; 15(01):1-4+113.

395. Liu XC, Wang JX, Wei P. [The Function of Helper T Lymphocytes in Ankylosing Spondylitis.]. Tianjin Medical Journal. 2010; 38(12):1047-1049.

396. Long S, Ma L, Wang D, Shang X. High frequency of circulating follicular helper T cells is correlated with B cell subtypes in patients with ankylosing spondylitis. Exp Ther Med. 2018 May; 15(5):4578-4586.

397. Ma XH, Zhang X, Zhang N, Zhao Y, Song LT. [Detection of lymphocyte subsets in peripheral blood in patients with ankylosing spondylitis and its clinical meaning.]. Chinese Journal of Laboratory Diagnosis. 2011; 15(10):1765-1766.

398. Ma L, Zhang Y, Wang ZQ, Gu J. [A study of subsets and activation of lymphocytes in patients with ankylosing spondylitis.]. Journal of Clinical Research. 2011; 28(10):1963-1964.

399. Ma L, Yang J, Li H. [Study of the activated state of TH1/TH2 cytokines on ankylosing spondylitis.]. Chinese Journal of Immunology. 2004; 20(08):572-574.

400. Meng JH, Wei P, Chen HY, Wang JX, Xie JL, Zhang Y. [Change of cytotoxic T-lymphocytes in patients with ankylosing spondylitis.]. Journal of Hebei Medical University. 2015; 36(05):543-546.

401. Mo JF, Shan DP, Bao Y, Ye Q, Yan WH. [Proportion of several subsets of immune cells and expression of CXCR6 on CD3+ , CD4+ and γδT cells in peripheral blood of patients with ankylosing spondylitis.]. Current Immunology. 2019; 39(03):217-221.

402. Shan Y, Qi C, Zhao J, Liu Y, Gao H, Zhao D, et al. Higher frequency of peripheral blood follicular regulatory T cells in patients with new onset ankylosing spondylitis. Clin Exp Pharmacol Physiol. 2015 Feb; 42(2):154-161.

403. Shen H, Goodall JC, Hill Gaston JS. Frequency and phenotype of peripheral blood Th17 cells in ankylosing spondylitis and rheumatoid arthritis. Arthritis Rheum. 2009 Jun; 60(6):1647-1656.

404. Suen JL, Li HT, Jong YJ, Chiang BL, Yen JH. Altered homeostasis of CD4(+) FoxP3(+) regulatory T-cell subpopulations in systemic lupus erythematosus. Immunology. 2009 Jun; 127(2):196-205.

405. Szalay B, Meszaros G, Cseh A, Acs L, Deak M, Kovacs L, et al. Adaptive immunity in ankylosing spondylitis: phenotype and functional alterations of T-cells before and during infliximab therapy. Clin Dev Immunol. 2012; 2012:808724.

406. Szántó S, Aleksza M, Mihály E, Lakos G, Szabó Z, Végvári A, et al. Intracytoplasmic cytokine expression and T cell subset distribution in the peripheral blood of patients with ankylosing spondylitis. J Rheumatol. 2008 Dec; 35(12):2372-2375.

407. Thoen J, Førre O, Waalen K, Pahle J. Phenotypes and spontaneous cell cytotoxicity of mononuclear cells from patients with seronegative spondyloarthropathies: ankylosing spondylitis, psoriatic arthropathy and pauciarticular juvenile chronic arthritis--analysis of mononuclear cells from peripheral blood, synovial fluid and synovial membranes. Clin Rheumatol. 1988 Mar; 7(1):95-106.

408. Toussirot E, Saas P, Deschamps M, Pouthier F, Perrot L, Perruche S, et al. Increased production of soluble CTLA-4 in patients with spondylarthropathies correlates with disease activity. Arthritis Res Ther. 2009; 11(4):R101.

409. Wang YF, Wang M, Song AF. [Evaluation value of peripheral Th17/Treg balance in patients with ankylosing spondylitis.]. International Journal of Laboratory Medicine. 2020; 41(07):842-845.

410. Wang CL, Li KZ, Cui W. [Study on expression ofTh1, Th17, Treg cells and related cytokines in ankylosing spondylitis patients.]. Chronic Pathematology Journal. 2018; 19(09):1154-1156+1160.

411. Wang M, Liu C, Bond A, Yang J, Zhou X, Wang J, et al. Dysfunction of regulatory T cells in patients with ankylosing spondylitis is associated with a loss of Tim-3. International Immunopharmacology. 2018; 59:53-60.

412. Wang H, Sun N, Li K, Tian J, Li J. Assay of Peripheral Regulatory Vδ1 T Cells in Ankylosing Spondylitis and its Significance. Med Sci Monit. 2016 Sep 6; 22:3163-3168.

413. Wang ZL, Zhong NF, Ma L. [A Study on the Clinical Value and Correlation of Treg and Th17 Cells among Different Active Stages of Ankylosing Spondylitis.]. Journal of Guizhou Medical University. 2015; 40(01):68-71+75.

414. Wang C, Liao Q, Hu Y, Zhong D. T lymphocyte subset imbalances in patients contribute to ankylosing spondylitis. Exp Ther Med. 2015 Jan; 9(1):250-256.

415. Wang YF, Xu LH, Jiang LX, Qi CP, Wang Y. [Clinical Significance of Detecting Immune Functions on Patients with Ankylosing Spondylitis.]. Journal of Modern Laboratory Medicine. 2012; 27(06):132-134.

416. Wang JX, Wei P, Meng JH, Liu XC, Liu YJ, Gu G, et al. [Expression and significance of Thl／Th2 cytokines in ankylosing spondylitis.]. Clinical Medicine of China. 2008; 24(10):989-990.

417. Wei YY, Han ZJ, Huang HY, Du W, Ren TL, Gao MZ. [Analysis of Treg cells and lymphocyte subgroup in 131 patients with ankylosing spondylitis.]. China Medical Herald. 2017; 14(28):46-48.

418. Wu SS. [Association of Follicular Helper T Cells and Ankylosing Spondylitis.] [master’s thesis]: Anhui Medical University; 2014.

419. Wu Y, Ren M, Yang R, Liang X, Ma Y, Tang Y, et al. Reduced immunomodulation potential of bone marrow-derived mesenchymal stem cells induced CCR4+CCR6+ Th/Treg cell subset imbalance in ankylosing spondylitis. Arthritis Res Ther. 2011 Feb 21; 13(1):R29.

420. Wu HK, Zhou L, Zhang LZ, Zhong RQ. [The expression research of B lymphocyte subsets，B-cell activating factor and its receptor BR3 in peripheral blood from patients with ankylosing spondylitis.]. Laboratory Medicine. 2011; 26(12):818-822.

421. Xu F, Guanghao C, Liang Y, Jun W, Wei W, Baorong H. Treg-promoted New Bone Formation Through Suppressing TH17 by Secreting Interleukin-10 in Ankylosing Spondylitis. Spine (Phila Pa 1976). 2019 Dec 1; 44(23):E1349-E1355.

422. Xu WL, Luo Y, Li K, Liao CZ, Lin YH, Zhang HD. [Eepression and significance of T lymphocyte subgroup and natural killer T cells in peripheral blood of ankylosing spondylitis patients.]. Laboratory Medicine and Clinic. 2018; 15(02):192-194.

423. Xu XX. [The differentiation of Th1/Th17/Treg cells and their expression of associated transcription factors and cytokines in patients with ankylosing spondylitis.] [master’s thesis]: Fujian Medical University; 2013.

424. Xu XF, Jiang LH, Gao WH, Tao L, Huang LJ, Xu QB. [Detection of HLA-B27 and T lymphocyte subsets in patients with ankylosing spondylitis and its meaning.]. Laboratory Medicine and Clinic. 2011; 8(19):2366-2368.

425. Xue GH, Hua L, Liu XF, Chen XL, Dong L, Pan J, et al. [Frequencies of human regulatory B cells in PBMC in ankylosing spondylitis patients and its clinical significance.]. Chinese Journal of Clinical Laboratory Science. 2015; 33(09):662-667.

426. Xue YH, Cai YT, Xie KC. [Expression of CD28/CD152 : CD80/CD86 on peripheral blood lymphocytes of patients with ankylosing spondylitis.]. Laboratory Medicine. 2008; 23(05):478-480.

427. Yang M, Lv Q, Wei Q, Jiang Y, Qi J, Xiao M, et al. TNF-alpha inhibitor therapy can improve the immune imbalance of CD4+ T cells and negative regulatory cells but not CD8+ T cells in ankylosing spondylitis. Arthritis Res Ther. 2020 Jun 19; 22(1):149.

428. Yang WH, Shu R, Han YX, Yuan W, Yu P, Li N, et al. [Levels of natural killer cells in patients with ankylosing spondylitis and its clinical meaning.]. Guangxi Medical Journal. 2018; 40(10):1241-1242+1245.

429. Yang X. [Changes and Clinical Significance of CD8+ Regulatory T Cells in the Peripheral Blood of Patients with Ankylosing Spondylitis.] [master’s thesis]: Anhui Medical University; 2017.

430. Yang FF, Zhang X, Zhu P. [Percentage of peripheral effector T cells in active phase patients with ankylosing spondylitis is increased while level of PD-L1 is decreased.]. Chinese Journal of Cellular and Molecular Immunology. 2016; 32(05):676-679.

431. Yang GM, Wang YF, Ma YF, Li SQ, Tan Y. [Study on lymphocytes and CD28CD40 in peripheral blood of patients with Ankylosing Spondylitis.]. Chinese Journal of Laboratory Diagnosis. 2007; 11(11):1486-1489.

432. Ye L, Zhang L, Goodall J, Gaston H, Xu H. Altered frequencies of regulatory t-cell subsets in ankylosing spondylitis and rheumatoid arthritis patients and their response to anti-TNF therapy. Rheumatology (United Kingdom). 2013; 52:i135-i136.

433. Zhang CQ, Fang LH, Liu XP, Li R, Cui LP, Wang J, et al. [Differential expression and meaning of Th17 and Tregs in patients with ankylosing spondylitis and psoriatic arthritis.]. Chinese Remedies & Clinics. 2019; 19(01):34-36.

434. Zhang Y, Ma L, Shen X, Fang ZY, Lin J. [An observation of changes in counts and percentages of peripheral lymphocyte subsets in patients with ankylosing spondylitis.]. Shandong Medical Journal. 2019; 59(24):82-85.

435. Zhang HL, Zhang JY, Jin XY, Niu JX, Li ZJ, Zhang FL. [Study on the Correlation of the Imbalance of Th17 Cells，Th1 Cell，Ｒegulatory T Cells with ankylosing spondylitis Disease Activity Score.]. Medical Recapitulate. 2014; 20(24):4545-4546+4555.

436. Zhang X, Wang P, Wu YF, Yang R, Huang L, Tang Y, et al. [Allogeneic blood transfusion alleviates hip joint pain induced by ankylosing spondylitis.]. Chinese Journal of Tissue Engineering Research. 2014; 18(09):1465-1470.

437. Zhang L, Li YG, Li YH, Qi L, Liu XG, Yuan CZ, et al. Increased frequencies of Th22 cells as well as Th17 cells in the peripheral blood of patients with ankylosing spondylitis and rheumatoid arthritis. PLoS One. 2012; 7(4):e31000.

438. Zhang SH, Han YX, Wu JB, Hu XX, Chen D. [The alteration of CD4+ regulatory T cells in patients with ankylosing spondylitis.]. Chinese Journal of Microbiology and Immunology. 2008; 28(05):445-449.

439. Zhao JT, Li YJ. [The comparison of the ratio of Thl7／Treg cells in patients with ankylosing spondytitis and the normal controls.]. Chinese Journal of Rheumatology. 2013; 17(07):481-484.

440. Zhao SS, Hu JW, Wang J, Lou XJ, Zhou LL. Inverse correlation between CD4+CD25highCD127low/- regulatory T-cells and serum immunoglobulin A in patients with new-onset ankylosing spondylitis. Journal of International Medical Research. 2011; 39(5):1968-1974.

441. Zhao XZ, Song HC, Cui LF. [Clinical significance and expression of T cell subsets in peripheral blood from patients witb ankylosing spondylitis.]. Clinical Medicine of China. 2009; 25(03):286-288.

442. Zhong NF, Ma L. [Clinical significance of Th1，Th2 and Th17 cell determinations among different active stage ankylosing spondylitis patients.]. Laboratory Medicine. 2014; 29(05):477-482.

443. Zhu YY, Zhang L, Sun R, Zhao X, Xu YZ, Wu T, et al. [Changes in T lymphocyte subsets and related immune molecules in peripheral blood in patients with ankylosing spondylitis.]. Current Immunology. 2017; 37(01):14-19.

444. Zhu LY, Yang WJ, Yu P, Wang J, Li N, Wang JR, et al. [Expression of natural killer cell in patients with ankylosing spondylitis and its significance.]. Chinese Journal of Coal Industry Medicine. 2016; 19(07):989-992.

445. Zhu J, Liu XY, Huang F. [Th1/Th2 balance and ankylosing spondylitis.]. Chinese Journal of Rheumatology. 2000; 4(04):202-205.

446. Marhoffer W, Stracke H, Masoud I, Scheja M, Graef V, Bolten W, et al. Evidence of impaired cartilage/bone turnover in patients with active ankylosing spondylitis. Ann Rheum Dis. 1995 Jul; 54(7):556-559.

447. Toussirot E, Ricard-Blum S, Dumoulin G, Cedoz JP, Wendling D. Relationship between urinary pyridinium cross-links, disease activity and disease subsets of ankylosing spondylitis. Rheumatology (Oxford). 1999 Jan; 38(1):21-27.

448. Yilmaz N, Ozaslan J. Biochemical bone turnover markers in patients with ankylosing spondylitis. Clin Rheumatol. 2000; 19(2):92-98.

449. Lange U, Teichmann J, Strunk J, Muller-Ladner U, Schmidt KL. Association of 1.25 vitamin D3 deficiency, disease activity and low bone mass in ankylosing spondylitis. Osteoporos Int. 2005 Dec; 16(12):1999-2004.

450. Muntean L, Rojas-Vargas M, Font P, Simon SP, Rednic S, Schiotis R, et al. Relative value of the lumbar spine and hip bone mineral density and bone turnover markers in men with ankylosing spondylitis. Clin Rheumatol. 2011 May; 30(5):691-695.

451. Taylan A, Sari I, Akinci B, Bilge S, Kozaci D, Akar S, et al. Biomarkers and cytokines of bone turnover: extensive evaluation in a cohort of patients with ankylosing spondylitis. BMC Musculoskelet Disord. 2012 Oct 2; 13:191.

452. Korkosz M, Gasowski J, Leszczynski P, Pawlak-Bus K, Jeka S, Kucharska E, et al. High disease activity in ankylosing spondylitis is associated with increased serum sclerostin level and decreased wingless protein-3a signaling but is not linked with greater structural damage. BMC Musculoskelet Disord. 2013 Mar 19; 14:99.

453. Almodovar R, Rios V, Ocana S, Gobbo M, Casas ML, Zarco-Montejo P, et al. Association of biomarkers of inflammation, cartilage and bone turnover with gender, disease activity, radiological damage and sacroiliitis by magnetic resonance imaging in patients with early spondyloarthritis. Clin Rheumatol. 2014 Feb; 33(2):237-241.

454. Klingberg E, Nurkkala M, Carlsten H, Forsblad-d'Elia H. Biomarkers of bone metabolism in ankylosing spondylitis in relation to osteoproliferation and osteoporosis. J Rheumatol. 2014 Jul; 41(7):1349-1356.

455. Nocturne G, Pavy S, Boudaoud S, Seror R, Goupille P, Chanson P, et al. Increase in Dickkopf-1 Serum Level in Recent Spondyloarthritis. Data from the DESIR Cohort. PLoS One. 2015; 10(8):e0134974.

456. Wang L, Gao L, Jin D, Wang P, Yang B, Deng W, et al. The Relationship of Bone Mineral Density to Oxidant/Antioxidant Status and Inflammatory and Bone Turnover Markers in a Multicenter Cross-Sectional Study of Young Men with Ankylosing Spondylitis. Calcif Tissue Int. 2015 Jul; 97(1):12-22.

457. Zhang P, Li Q, Wei Q, Liao Z, Lin Z, Fang L, et al. Serum Vitamin D and Pyridinoline Cross-Linked Carboxyterminal Telopeptide of Type I Collagen in Patients with Ankylosing Spondylitis. Biomed Res Int. 2015; 2015:543806.

458. Gula Z, Kopczynska A, Hanska K, Slomski M, Nowakowski J, Kwasny-Krochin B, et al. Vitamin D serum concentration is not related to the activity of spondyloarthritis - preliminary study. Reumatologia. 2018; 56(6):388-391.

459. Descamps E, Molto A, Borderie D, Lories R, Richard CM, Pons M, et al. Changes in bone formation regulator biomarkers in early axial spondyloarthritis. Rheumatology (Oxford). 2021 Mar 2; 60(3):1185-1194.

460. Speden DJ, Calin AI, Ring FJ, Bhalla AK. Bone mineral density, calcaneal ultrasound, and bone turnover markers in women with ankylosing spondylitis. J Rheumatol. 2002 Mar; 29(3):516-521.

461. Franck H, Meurer T, Hofbauer LC. Evaluation of bone mineral density, hormones, biochemical markers of bone metabolism, and osteoprotegerin serum levels in patients with ankylosing spondylitis. J Rheumatol. 2004 Nov; 31(11):2236-2241.

462. Grisar J, Bernecker PM, Aringer M, Redlich K, Sedlak M, Wolozcszuk W, et al. Ankylosing spondylitis, psoriatic arthritis, and reactive arthritis show increased bone resorption, but differ with regard to bone formation. J Rheumatol. 2002 Jul; 29(7):1430-1436.

463. Acebes C, de la Piedra C, Traba ML, Seibel MJ, Garcia Martin C, Armas J, et al. Biochemical markers of bone remodeling and bone sialoprotein in ankylosing spondylitis. Clin Chim Acta. 1999 Nov; 289(1-2):99-110.

464. Huang J, Song G, Yin Z, Fu Z, Ye Z. Alteration of Bone Turnover Markers in Canonical Wingless Pathway in Patients With Ankylosing Spondylitis. Arch Rheumatol. 2016 Sep; 31(3):221-228.

465. Park MC, Chung SJ, Park YB, Lee SK. Bone and cartilage turnover markers, bone mineral density, and radiographic damage in men with ankylosing spondylitis. Yonsei Med J. 2008 Apr 30; 49(2):288-294.

466. Vosse D, Landewe R, Garnero P, van der Heijde D, van der Linden S, Geusens P. Association of markers of bone- and cartilage-degradation with radiological changes at baseline and after 2 years follow-up in patients with ankylosing spondylitis. Rheumatology (Oxford). 2008 Aug; 47(8):1219-1222.

467. Arends S, Spoorenberg A, Efde M, Bos R, Leijsma MK, Bootsma H, et al. Higher bone turnover is related to spinal radiographic damage and low bone mineral density in ankylosing spondylitis patients with active disease: a cross-sectional analysis. PLoS One. 2014; 9(6):e99685.

468. Gamez-Nava JI, de la Cerda-Trujillo LF, Vazquez-Villegas ML, Cons-Molina F, Alcaraz-Lopez MF, Zavaleta-Muniz SA, et al. Association between bone turnover markers, clinical variables, spinal syndesmophytes and bone mineral density in Mexican patients with ankylosing spondylitis. Scand J Rheumatol. 2016 Nov; 45(6):480-490.

469. Sun W, Tian L, Jiang L, Zhang S, Zhou M, Zhu J, et al. Sclerostin rather than Dickkopf-1 is associated with mSASSS but not with disease activity score in patients with ankylosing spondylitis. Clin Rheumatol. 2019 Apr; 38(4):989-995.

470. Iaremenko O, Shynkaruk I, Fedkov D, Iaremenko K, Petelytska L. Bone turnover biomarkers, disease activity, and MRI changes of sacroiliac joints in patients with spondyloarthritis. Rheumatol Int. 2020 Dec; 40(12):2057-2063.

471. Liu W, Song H, Man S, Li H, Zhang L. Analysis of Bone Strength and Bone Turnover Markers in Ankylosing Spondylitis with Radiological Hip Involvement. Med Sci Monit. 2021 Jun 27; 27:e932992.

472. Briot K, Garnero P, Le Henanff A, Dougados M, Roux C. Body weight, body composition, and bone turnover changes in patients with spondyloarthropathy receiving anti-tumour necrosis factor alpha treatment. Ann Rheum Dis. 2005 Aug; 64(8):1137-1140.

473. Visvanathan S, van der Heijde D, Deodhar A, Wagner C, Baker DG, Han J, et al. Effects of infliximab on markers of inflammation and bone turnover and associations with bone mineral density in patients with ankylosing spondylitis. Ann Rheum Dis. 2009 Feb; 68(2):175-182.

474. Arends S, Spoorenberg A, Houtman PM, Leijsma MK, Bos R, Kallenberg CG, et al. The effect of three years of TNFalpha blocking therapy on markers of bone turnover and their predictive value for treatment discontinuation in patients with ankylosing spondylitis: a prospective longitudinal observational cohort study. Arthritis Res Ther. 2012 Apr 30; 14(2):R98.

475. Kwon SR, Lim MJ, Suh CH, Park SG, Hong YS, Yoon BY, et al. Dickkopf-1 level is lower in patients with ankylosing spondylitis than in healthy people and is not influenced by anti-tumor necrosis factor therapy. Rheumatol Int. 2012 Aug; 32(8):2523-2527.

476. Saad CG, Ribeiro AC, Moraes JC, Takayama L, Goncalves CR, Rodrigues MB, et al. Low sclerostin levels: a predictive marker of persistent inflammation in ankylosing spondylitis during anti-tumor necrosis factor therapy? Arthritis Res Ther. 2012 Oct 12; 14(5):R216.

477. de Andrade KR, de Castro GR, Vicente G, da Rosa JS, Nader M, Pereira IA, et al. Evaluation of circulating levels of inflammatory and bone formation markers in axial spondyloarthritis. Int Immunopharmacol. 2014 Aug; 21(2):481-486.

478. Li H, Li Q, Chen X, Ji C, Gu J. Anti-tumor Necrosis Factor Therapy Increased Spine and Femoral Neck Bone Mineral Density of Patients with Active Ankylosing Spondylitis with Low Bone Mineral Density. J Rheumatol. 2015 Aug; 42(8):1413-1417.

479. Sharma S, Mohanan S, Sharma S. Effect of anti-tumor necrosis factor alpha therapy on bone health and biomarkers of bone turnover in Indian patients with ankylosing spondylitis. Indian Journal of Rheumatology. 2017; 12(1).

480. Gulyas K, Horvath A, Vegh E, Pusztai A, Szentpetery A, Petho Z, et al. Effects of 1-year anti-TNF-alpha therapies on bone mineral density and bone biomarkers in rheumatoid arthritis and ankylosing spondylitis. Clin Rheumatol. 2020 Jan; 39(1):167-175.

481. Braun J, Buehring B, Baraliakos X, Gensler LS, Porter B, Quebe-Fehling E, et al. Effects of secukinumab on bone mineral density and bone turnover biomarkers in patients with ankylosing spondylitis: 2-year data from a phase 3 study, MEASURE 1. BMC Musculoskelet Disord. 2021 Dec 13; 22(1):1037.

482. Choi ST, Kim JH, Kang EJ, Lee SW, Park MC, Park YB, et al. Osteopontin might be involved in bone remodelling rather than in inflammation in ankylosing spondylitis. Rheumatology (Oxford). 2008 Dec; 47(12):1775-1779.

483. Li R, Zhang G, Li Y, Li Y, Ren Z, Zhang Y, et al. [The Prevalences of Osteoporosis in Patients with Ankylosing Spondylitis and the Changes of Bone Mineral Density and Bone Metabolic Biochemical Markers among Patients]. Chinese General Practice. 2014; 17(9):1049-1051.

484. Zou H, Ji H, Lu F, Lin J, Guo W. [The clinical analysis of osteoporosis secondary to ankylosing spondylitis]. Hebei Medical Journal. 2013; 35(21):3258-3259.

485. Liu J. [The clinical characteristics, bone mineral density and bone metabolism indexes in patients with ankylosing spondylitis combined with osteoporosis]. World Latest Medicine Information. 2015; 15(65):154.

486. Kang R, Chen J, Zheng L, Zheng Q, Zeng X. [Study of the changes of bone mineral density and bone metabolic markers in patients with ankylosing spondylitis]. Chinese Journal of Osteoporosis. 2016; 22(1):61-66.

487. Wei R, Dong L, Wu H, Xu Y, Zhang J. [The correlation of bone metabolic indexes and levels of RANKL-RANK-OPG system in ankylosing spondylitis patients]. Progress in Modern Biomedicine. 2013; 13(13):2528-2531.

488. Kong W, Yan X, Qin L, Zhang W, Ma H, Ge C, et al. [Osteoporosis in ankylosing spondylitis: a clinical observation of 97 cases]. The Journal of Traditional Chinese Orthopedics and Traumatology. 2005; 17(11):3-6.

489. Kang R, Wu X, Wu L, Zhan Y, Li W. [The study of the correlation between biochemical markers of bone metabolism and gene expression of BMP-2 and RUNX-2 in patients with ankylosing spondylitis]. Fujian Medical Journal. 2018; 40(4):35-38.

490. Wang F. [Expression of M-CSF, IL-6, CT and its correlation with bone metabolism of ankylosing spondylitis] [Master's Thesis]: Hebei Medical University; 2010.

491. Kong W, Yan X, Zhang W, Ge C, Yan J. [Bone mineral density(BMD),osteoprotegerin(OPG) and biochemical makers in patients with ankylosing spondylitis(AS)]. Chinese Journal of Osteoporosis. 2010; 16(1).

492. Xie JL, Chen H, Wei P, Wang J. [The effect of active vitamin D on bone metabolism and disease activity in ankylosing spondylitis patients with low bone mineral density]. Chinese Journal of Osteoporosis. 2018; 24(3):324-327.

493. Chen Z, Li X, Li X, Wang G, Xiang N, Ma Q, et al. [The relation between bone m ineraldensity and bone turnoverm arkersin ankylosing spondylitis patient]. Chinese Journal of Rheumatology. 2014; 18(6):413-417.

494. Lu P, Xiaoping. Y. [The study of clinical characteristics, bone mineral density and bone metabolic markers in 189 patients with ankylosing spondylitis]. Chinese Journal of Osteoporosis and Bone Mineral Research. 2012; 5(1):12-19.

495. Yang Y. [The clinical study of axial and peripheral spondyloarthritis with osteoporosis] [Master's Thesis]: Jilin University; 2016.

496. Fang Z, Li Y, Jiang Y, Yang F. [Significance of dynamic monitoring of bone metabolites in patients with ankylosing spondylitis]. Chinese Remedies & Clinics. 2014; 14(4):447-449.

497. Pan X, Fang X, Fan T. [A clinical study of 120 cases of osteoporosis secondary to ankylosing spondylitis]. Jilin Medical Journal. 2014; 35(15):3230-3231.

498. Xu L, Lin J, Xu D, Xu B, Cao H, Sun D. [Preliminary observation on changes of bone metabolism markers in patients with ankylosing spondylitis]. *2009 Zhejiang Provincial Rheumatology Conference*. Ningbo, Zhejiang, China 2009.

499. Tan L, Feng X, Jiao A, Chen J, Tan F, He S, et al. [Potential clinical value of detecting markers of bone metabolism in patients with ankylosing spondylitis]. Chinese Journal of Immunology. 2017; 33(10):1543-1546.

500. Bay-Jensen AC, Leeming DJ, Kleyer A, Veidal SS, Schett G, Karsdal MA. Ankylosing spondylitis is characterized by an increased turnover of several different metalloproteinase-derived collagen species: a cross-sectional study. Rheumatol Int. 2012 Nov; 32(11):3565-3572.

501. Holm Nielsen S, Sun S, Bay-Jensen AC, Karsdal M, Sorensen IJ, Weber U, et al. Levels of extracellular matrix metabolites are associated with changes in Ankylosing Spondylitis Disease Activity Score and MRI inflammation scores in patients with axial spondyloarthritis during TNF inhibitor therapy. Arthritis Res Ther. 2022 Dec 23; 24(1):279.

502. Husakova M, Siebuhr AS, Pavelka K, Spiritovic M, Bay-Jensen AC, Levitova A. Changes of patient-reported outcomes and protein fingerprint biomarkers after exercise therapy for axial spondyloarthritis. Clin Rheumatol. 2019 Jan; 38(1):173-179.

503. Port H, Nielsen SH, Madsen SF, Bay-Jensen AC, Karsdal M, Seven S, et al. Extracellular matrix protein turnover markers are associated with axial spondyloarthritis-a comparison with postpartum women and other non-axial spondyloarthritis controls with or without back pain. Arthritis Res Ther. 2022 Jun 23; 24(1):152.

504. Siebuhr AS, van der Heijde D, Bay-Jensen AC, Karsdal MA, Landewe R, van Tubergen A, et al. Is radiographic progression in radiographic axial spondyloarthritis related to matrix metalloproteinase degradation of extracellular matrix? RMD Open. 2018; 4(1):e000648.

505. Siebuhr AS, Bay-Jensen AC, Karsdal MA, Lories RJ, de Vlam K. CRP and a biomarker of type I collagen degradation, C1M, can differentiate anti-inflammatory treatment response in ankylosing spondylitis. Biomark Med. 2016; 10(2):197-208.

506. Gudmann NS, Siebuhr AS, Christensen AF, Ejstrup L, Sørensen GL, Loft AG, et al. Type I and III collagen turnover is increased in axial spondyloarthritis and psoriatic arthritis. Associations with disease activity and diagnostic capacity. Clin Exp Rheumatol. 2017 Jul-Aug; 35(4):653-659.

507. Bay-Jensen AC, Karsdal MA, Vassiliadis E, Wichuk S, Marcher-Mikkelsen K, Lories R, et al. Circulating citrullinated vimentin fragments reflect disease burden in ankylosing spondylitis and have prognostic capacity for radiographic progression. Arthritis Rheum. 2013 Apr; 65(4):972-980.

508. Siebuhr AS, Hušaková M, Forejtová S, Zegzulková K, Tomčik M, Urbanová M, et al. Metabolites of C-reactive protein and vimentin are associated with disease activity of axial spondyloarthritis. Clin Exp Rheumatol. 2019 May-Jun; 37(3):358-366.

509. Husakova M, Bay-Jensen AC, Forejtova S, Zegzulkova K, Tomcik M, Gregova M, et al. Metabolites of type I, II, III, and IV collagen may serve as markers of disease activity in axial spondyloarthritis. Sci Rep. 2019 Aug 2; 9(1):11218.

510. Aschermann S, Englbrecht M, Bergua A, Spriewald BM, Said-Nahal R, Breban M, et al. Presence of HLA-B27 is associated with changes of serum levels of mediators of the Wnt and hedgehog pathway. Joint Bone Spine. 2016 Jan; 83(1):43-46.

511. Ustun N, Tok F, Kalyoncu U, Motor S, Yuksel R, Yagiz AE, et al. Sclerostin and Dkk-1 in patients with ankylosing spondylitis. Acta Reumatol Port. 2014 Apr-Jun; 39(2):146-151.

512. Rossini M, Viapiana O, Idolazzi L, Ghellere F, Fracassi E, Troplini S, et al. Higher Level of Dickkopf-1 is Associated with Low Bone Mineral Density and Higher Prevalence of Vertebral Fractures in Patients with Ankylosing Spondylitis. Calcif Tissue Int. 2016 May; 98(5):438-445.

513. Solmaz D, Uslu S, Kozaci D, Karaca N, Bulbul H, Tarhan EF, et al. Evaluation of periostin and factors associated with new bone formation in ankylosing spondylitis: Periostin may be associated with the Wnt pathway. Int J Rheum Dis. 2018 Feb; 21(2):502-509.

514. Appel H, Ruiz-Heiland G, Listing J, Zwerina J, Herrmann M, Mueller R, et al. Altered skeletal expression of sclerostin and its link to radiographic progression in ankylosing spondylitis. Arthritis Rheum. 2009 Nov; 60(11):3257-3262.

515. Tuylu T, Sari I, Solmaz D, Kozaci DL, Akar S, Gunay N, et al. Fetuin-A is related to syndesmophytes in patients with ankylosing spondylitis: a case control study. Clinics (Sao Paulo). 2014 Dec; 69(10):688-693.

516. Perrotta FM, Ceccarelli F, Barbati C, Colasanti T, De Socio A, Scriffignano S, et al. Serum Sclerostin as a Possible Biomarker in Ankylosing Spondylitis: A Case-Control Study. J Immunol Res. 2018; 2018:9101964.

517. Pathan E, Abraham S, Van Rossen E, Withrington R, Keat A, Charles PJ, et al. Efficacy and safety of apremilast, an oral phosphodiesterase 4 inhibitor, in ankylosing spondylitis. Ann Rheum Dis. 2013 Sep 1; 72(9):1475-1480.

518. Atas N, Cakir B, Bakir F, Ucar M, Satis H, Guz GT, et al. The impact of anti-TNF treatment on Wnt signaling, noggin, and cytokine levels in axial spondyloarthritis. Clin Rheumatol. 2022 May; 41(5):1381-1389.

519. Guo Y, Jiang D, Mai Z, Chen Y, Li T, Gao G. Imrecoxib and celecoxib affect sacroiliac joint inflammation in axSpA by regulating bone metabolism and angiogenesis. Clin Rheumatol. 2023 Jun; 42(6):1585-1592.

520. Ozdemirel AE, Guven SC, Doganci A, Sari Surmeli Z, Ozyuvali A, Kurt M, et al. Anti-tumor necrosis factor alpha treatment does not influence serum levels of the markers associated with radiographic progression in ankylosing spondylitis. Arch Rheumatol. 2023 Mar; 38(1):148-155.

521. Fassio A, Gatti D, Rossini M, Idolazzi L, Giollo A, Adami G, et al. Secukinumab produces a quick increase in WNT signalling antagonists in patients with psoriatic arthritis. Clin Exp Rheumatol. 2019 Jan-Feb; 37(1):133-136.

522. Rubio Vargas R, Melguizo Madrid E, Gonzalez Rodriguez C, Navarro Sarabia F, Dominguez Quesada C, Ariza Ariza R, et al. Association between serum dickkopf-1 levels and disease duration in axial spondyloarthritis. Reumatol Clin. 2017 Jul-Aug; 13(4):197-200.

523. Papagoras C, Tsiami S, Chrysanthopoulou A, Mitroulis I, Baraliakos X. Serum granulocyte-macrophage colony-stimulating factor (GM-CSF) is increased in patients with active radiographic axial spondyloarthritis and persists despite anti-TNF treatment. Arthritis Res Ther. 2022 Aug 16; 24(1):195.

524. Wu M, Chen M, Ma Y, Yang J, Han R, Yuan Y, et al. Dickkopf-1 in ankylosing spondylitis: Review and meta-analysis. Clin Chim Acta. 2018 Jun; 481:177-183.

525. Niu CC, Lin SS, Yuan LJ, Chen LH, Yang CY, Chung AN, et al. Correlation of blood bone turnover biomarkers and Wnt signaling antagonists with AS, DISH, OPLL, and OYL. BMC Musculoskelet Disord. 2017 Feb 2; 18(1):61.

526. Liao HT, Lin YF, Tsai CY, Chou TC. Bone morphogenetic proteins and Dickkopf-1 in ankylosing spondylitis. Scand J Rheumatol. 2018 Jan; 47(1):56-61.

527. Daoussis D, Liossis SN, Solomou EE, Tsanaktsi A, Bounia K, Karampetsou M, et al. Evidence that Dkk-1 is dysfunctional in ankylosing spondylitis. Arthritis Rheum. 2010 Jan; 62(1):150-158.

528. Serdaroglu Beyazal M, Erdogan T, Turkyilmaz AK, Devrimsel G, Cure MC, Beyazal M, et al. Relationship of serum osteoprotegerin with arterial stiffness, preclinical atherosclerosis, and disease activity in patients with ankylosing spondylitis. Clin Rheumatol. 2016 Sep; 35(9):2235-2241.

529. Caparbo VF, Saad CGS, Moraes JC, de Brum-Fernandes AJ, Pereira RMR. Monocytes from male patients with ankylosing spondylitis display decreased osteoclastogenesis and decreased RANKL/OPG ratio. Osteoporos Int. 2018 Nov; 29(11):2565-2573.

530. Chen CH, Chen HA, Liao HT, Liu CH, Tsai CY, Chou CT. Soluble receptor activator of nuclear factor-kappaB ligand (RANKL) and osteoprotegerin in ankylosing spondylitis: OPG is associated with poor physical mobility and reflects systemic inflammation. Clin Rheumatol. 2010 Oct; 29(10):1155-1161.

531. Dhir V, Srivastava R, Aggarwal A. Circulating Levels of Soluble Receptor Activator of NF- kappa B Ligand and Matrix Metalloproteinase 3 (and Their Antagonists) in Asian Indian Patients with Ankylosing Spondylitis. Int J Rheumatol. 2013; 2013:814350.

532. Genre F, López-Mejías R, Miranda-Filloy JA, Ubilla B, Carnero-López B, Palmou-Fontana N, et al. Osteoprotegerin correlates with disease activity and endothelial activation in non-diabetic ankylosing spondylitis patients undergoing TNF-α antagonist therapy. Clin Exp Rheumatol. 2014 Sep-Oct; 32(5):640-646.

533. Genre F, Rueda-Gotor J, Remuzgo-Martínez S, Corrales A, Ubilla B, Mijares V, et al. Implication of osteoprotegerin and sclerostin in axial spondyloarthritis cardiovascular disease: study of 163 Spanish patients. Clin Exp Rheumatol. 2018 Mar-Apr; 36(2):302-309.

534. Hou C, Luan L, Ren C. Oxidized low-density lipoprotein promotes osteoclast differentiation from CD68 positive mononuclear cells by regulating HMGB1 release. Biochem Biophys Res Commun. 2018 Jan 1; 495(1):1356-1362.

535. Jadon DR, Sengupta R, Nightingale A, Lu H, Dunphy J, Green A, et al. Serum bone-turnover biomarkers are associated with the occurrence of peripheral and axial arthritis in psoriatic disease: a prospective cross-sectional comparative study. Arthritis Res Ther. 2017 Sep 21; 19(1):210.

536. Kim HR, Lee SH, Kim HY. Elevated serum levels of soluble receptor activator of nuclear factors-kappaB ligand (sRANKL) and reduced bone mineral density in patients with ankylosing spondylitis (AS). Rheumatology (Oxford). 2006 Oct; 45(10):1197-1200.

537. Liu J, Zhao L, Yang X, Liu C, Kong N, Yu Y, et al. Bone mineral density, bone metabolism-related factors, and microRNA-218 are correlated with disease activities in Chinese ankylosing spondylitis patients. J Clin Lab Anal. 2022 Feb; 36(2):e24223.

538. Mou YK, Zhang PP, Li QX, Lin ZM, Liao ZT, Wei QJ, et al. Changes of serum levels of MMP-3, sRANKL, and OPG in juvenile-onset ankylosing spondylitis patients carrying different HLA-B27 subtypes. Clin Rheumatol. 2015 Jun; 34(6):1085-1089.

539. Stupphann D, Rauner M, Krenbek D, Patsch J, Pirker T, Muschitz C, et al. Intracellular and surface RANKL are differentially regulated in patients with ankylosing spondylitis. Rheumatol Int. 2008 Aug; 28(10):987-993.

540. Wang CM, Tsai SC, Lin JC, Wu YJ, Wu J, Chen JY. Association of Genetic Variants of RANK, RANKL, and OPG with Ankylosing Spondylitis Clinical Features in Taiwanese. Mediators Inflamm. 2019; 2019:8029863.

541. Woo JH, Lee HJ, Sung IH, Kim TH. Changes of clinical response and bone biochemical markers in patients with ankylosing spondylitis taking etanercept. J Rheumatol. 2007 Aug; 34(8):1753-1759.

542. Lorenzin M, Ortolan A, Felicetti M, Favero M, Vio S, Zaninotto M, et al. Serological Biomarkers in Early Axial Spondyloarthritis During 24-Months Follow Up (Italian Arm of Space Study). Front Med (Lausanne). 2019; 6:177.

543. Ramonda R, Modesti V, Ortolan A, Scanu A, Bassi N, Oliviero F, et al. Serological markers in psoriatic arthritis: promising tools. Exp Biol Med (Maywood). 2013 Dec; 238(12):1431-1436.

544. Soliman E, Labib W, el-Tantawi G, Hamimy A, Alhadidy A, Aldawoudy A. Role of matrix metalloproteinase-3 (MMP-3) and magnetic resonance imaging of sacroiliitis in assessing disease activity in ankylosing spondylitis. Rheumatol Int. 2012 Jun; 32(6):1711-1720.

545. Arends S, van der Veer E, Groen H, Houtman PM, Jansen TL, Leijsma MK, et al. Serum MMP-3 level as a biomarker for monitoring and predicting response to etanercept treatment in ankylosing spondylitis. J Rheumatol. 2011 Aug; 38(8):1644-1650.

546. Wendling D, Cedoz JP, Racadot E. Serum levels of MMP-3 and cathepsin K in patients with ankylosing spondylitis: effect of TNFalpha antagonist therapy. Joint Bone Spine. 2008 Oct; 75(5):559-562.

547. Maksymowych WP, Rahman P, Shojania K, Olszynski WP, Thomson GT, Ballal S, et al. Beneficial effects of adalimumab on biomarkers reflecting structural damage in patients with ankylosing spondylitis. J Rheumatol. 2008 Oct; 35(10):2030-2037.

548. Chen CH, Lin KC, Yu DT, Yang C, Huang F, Chen HA, et al. Serum matrix metalloproteinases and tissue inhibitors of metalloproteinases in ankylosing spondylitis: MMP-3 is a reproducibly sensitive and specific biomarker of disease activity. Rheumatology (Oxford). 2006 Apr; 45(4):414-420.

549. Yang C, Gu J, Rihl M, Baeten D, Huang F, Zhao M, et al. Serum levels of matrix metalloproteinase 3 and macrophage colony-stimulating factor 1 correlate with disease activity in ankylosing spondylitis. Arthritis Rheum. 2004 Oct 15; 51(5):691-699.

550. Kaaij MH, Helder B, van Mens LJJ, van de Sande MGH, Baeten DLP, Tas SW. Anti-IL-17A treatment reduces serum inflammatory, angiogenic and tissue remodeling biomarkers accompanied by less synovial high endothelial venules in peripheral spondyloarthritis. Sci Rep. 2020 Dec 3; 10(1):21094.

551. Turina MC, Yeremenko N, Paramarta JE, De Rycke L, Baeten D. Calprotectin (S100A8/9) as serum biomarker for clinical response in proof-of-concept trials in axial and peripheral spondyloarthritis. Arthritis Res Ther. 2014 Aug 19; 16(4):413.

552. Chandran V, Shen H, Pollock RA, Pellett FJ, Carty A, Cook RJ, et al. Soluble biomarkers associated with response to treatment with tumor necrosis factor inhibitors in psoriatic arthritis. J Rheumatol. 2013 Jun; 40(6):866-871.

553. Maksymowych WP, Landewe R, Conner-Spady B, Dougados M, Mielants H, van der Tempel H, et al. Serum matrix metalloproteinase 3 is an independent predictor of structural damage progression in patients with ankylosing spondylitis. Arthritis Rheum. 2007 Jun; 56(6):1846-1853.

554. Ozdemirel AE, Guven SC, Sari Surmeli Z, Ozyuvali A, Kurt M, Rustemova D, et al. Serum BMP-2 and BMP-4 levels and their relationship with disease activity in patients with rheumatoid arthritis and ankylosing spondylitis. Arch Rheumatol. 2022 Sep; 37(3):466-474.

555. Park MC, Park YB, Lee SK. Relationship of bone morphogenetic proteins to disease activity and radiographic damage in patients with ankylosing spondylitis. Scand J Rheumatol. 2008 May-Jun; 37(3):200-204.

556. Chen HA, Chen CH, Lin YJ, Chen PC, Chen WS, Lu CL, et al. Association of bone morphogenetic proteins with spinal fusion in ankylosing spondylitis. J Rheumatol. 2010 Oct; 37(10):2126-2132.

557. Bubova K, Prajzlerova K, Hulejova H, Gregova M, Mintalova K, Husakova M, et al. Elevated Tenascin-C Serum Levels in Patients With Axial Spondyloarthritis. Physiol Res. 2020 Aug 31; 69(4):653-660.

558. Gupta L, Bhattacharya S, Aggarwal A. Tenascin-C, a biomarker of disease activity in early ankylosing spondylitis. Clin Rheumatol. 2018 May; 37(5):1401-1405.

559. Alosami MHM, Al-Gebori AM, Al-Hindawi MS. Tenascin-C and Interleukin-17 Up-regulation in Axial Spondyloarthritis Patients. Rheumatology (Bulgaria). 2023; 30(4):3-11.

560. Hulejova H, Bubova K, Prajzlerová K, Husakova M, Filková M, Tomcík M, et al. Serum tenascin-C levels are elevated in patients with axial spondyloarthritis. Annals of the Rheumatic Diseases. 2019; 78:1496.

561. Berland M, Meslier V, Berreira Ibraim S, Le Chatelier E, Pons N, Maziers N, et al. Both Disease Activity and HLA-B27 Status Are Associated With Gut Microbiome Dysbiosis in Spondyloarthritis Patients. Arthritis Rheumatol. 2023 Jan; 75(1):41-52.

562. Berlinberg AJ, Regner EH, Stahly A, Brar A, Reisz JA, Gerich ME, et al. Multi 'Omics Analysis of Intestinal Tissue in Ankylosing Spondylitis Identifies Alterations in the Tryptophan Metabolism Pathway. Front Immunol. 2021; 12:587119.

563. Breban M, Tap J, Leboime A, Said-Nahal R, Langella P, Chiocchia G, et al. Faecal microbiota study reveals specific dysbiosis in spondyloarthritis. Ann Rheum Dis. 2017 Sep; 76(9):1614-1622.

564. Cardoneanu A, Mihai C, Rezus E, Burlui A, Popa I, Cijevschi Prelipcean C. Gut microbiota changes in inflammatory bowel diseases and ankylosing spondilytis. J Gastrointestin Liver Dis. 2021 Mar 12; 30(1):46-54.

565. Cardoneanu A, Cozma S, Rezus C, Petrariu F, Burlui AM, Rezus E. Characteristics of the intestinal microbiome in ankylosing spondylitis. Exp Ther Med. 2021 Jul; 22(1):676.

566. Chen Z, Qi J, Wei Q, Zheng X, Wu X, Li X, et al. Variations in gut microbial profiles in ankylosing spondylitis: disease phenotype-related dysbiosis. Ann Transl Med. 2019 Oct; 7(20):571.

567. Chen Z, Zheng X, Wu X, Wu J, Li X, Wei Q, et al. Adalimumab Therapy Restores the Gut Microbiota in Patients With Ankylosing Spondylitis. Front Immunol. 2021; 12:700570.

568. Costello ME, Ciccia F, Willner D, Warrington N, Robinson PC, Gardiner B, et al. Brief Report: Intestinal Dysbiosis in Ankylosing Spondylitis. Arthritis Rheumatol. 2015 Mar; 67(3):686-691.

569. Dai Q, Xia X, He C, Huang Y, Chen Y, Wu Y, et al. Association of anti-TNF-alpha treatment with gut microbiota of patients with ankylosing spondylitis. Pharmacogenet Genomics. 2022 Sep 1; 32(7):247-256.

570. Huang R, Li F, Zhou Y, Zeng Z, He X, Fang L, et al. Metagenome-wide association study of the alterations in the intestinal microbiome composition of ankylosing spondylitis patients and the effect of traditional and herbal treatment. J Med Microbiol. 2020 Jun; 69(6):797-805.

571. Klingberg E, Magnusson MK, Strid H, Deminger A, Ståhl A, Sundin J, et al. A distinct gut microbiota composition in patients with ankylosing spondylitis is associated with increased levels of fecal calprotectin. Arthritis Res Ther. 2019 Nov 27; 21(1):248.

572. Li C, Zhang Y, Yan Q, Guo R, Chen C, Li S, et al. Alterations in the gut virome in patients with ankylosing spondylitis. Front Immunol. 2023; 14:1154380.

573. Liu G, Hao Y, Yang Q, Deng S. The Association of Fecal Microbiota in Ankylosing Spondylitis Cases with C-Reactive Protein and Erythrocyte Sedimentation Rate. Mediators Inflamm. 2020; 2020:8884324.

574. Min HK, Na HS, Jhun J, Lee SY, Choi SS, Park GE, et al. Identification of gut dysbiosis in axial spondyloarthritis patients and improvement of experimental ankylosing spondyloarthritis by microbiome-derived butyrate with immune-modulating function. Frontiers in Immunology. 2023; 14.

575. Sternes PR, Brett L, Phipps J, Ciccia F, Kenna T, de Guzman E, et al. Distinctive gut microbiomes of ankylosing spondylitis and inflammatory bowel disease patients suggest differing roles in pathogenesis and correlate with disease activity. Arthritis Res Ther. 2022 Jul 7; 24(1):163.

576. Thompson KN, Bonham KS, Ilott NE, Britton GJ, Colmenero P, Bullers SJ, et al. Alterations in the gut microbiome implicate key taxa and metabolic pathways across inflammatory arthritis phenotypes. Sci Transl Med. 2023 Jul 26; 15(706):eabn4722.

577. Tito RY, Cypers H, Joossens M, Varkas G, Van Praet L, Glorieus E, et al. Brief Report: Dialister as a Microbial Marker of Disease Activity in Spondyloarthritis. Arthritis Rheumatol. 2017 Jan; 69(1):114-121.

578. Wen C, Zheng Z, Shao T, Liu L, Xie Z, Le Chatelier E, et al. Quantitative metagenomics reveals unique gut microbiome biomarkers in ankylosing spondylitis. Genome Biol. 2017 Jul 27; 18(1):142.

579. Yin J, Sternes PR, Wang M, Song J, Morrison M, Li T, et al. Shotgun metagenomics reveals an enrichment of potentially cross-reactive bacterial epitopes in ankylosing spondylitis patients, as well as the effects of TNFi therapy upon microbiome composition. Ann Rheum Dis. 2020 Jan; 79(1):132-140.

580. You Y, Cai M, Zhang W, Lin JS. Altered bacterial profiles at genus level in the gut of ankylosing spondylitis patients with different ASDAS scores. Int J Rheum Dis. 2022 Feb; 25(2):239-241.

581. Zhang L, Han R, Zhang X, Fang G, Chen J, Li J, et al. Fecal microbiota in patients with ankylosing spondylitis: Correlation with dietary factors and disease activity. Clin Chim Acta. 2019 Oct; 497:189-196.

582. Zhang F, Ma C, Zhang B, Bi L. Dynamic changes in gut microbiota under the influence of smoking and TNF-alpha-blocker in patients with ankylosing spondylitis. Clin Rheumatol. 2020 Sep; 39(9):2653-2661.

583. Zhang F, Ma C, Zhang B. Dynamic Variations in Gut Microbiota in Ankylosing Spondylitis Patients Treated with Anti-TNF-alpha for Six Months. Ann Clin Lab Sci. 2020 Jan; 50(1):99-106.

584. Zhou C, Zhao H, Xiao XY, Chen BD, Guo RJ, Wang Q, et al. Metagenomic profiling of the pro-inflammatory gut microbiota in ankylosing spondylitis. J Autoimmun. 2020 Feb; 107:102360.

585. Chen R, Han S, Dong D, Wang Y, Liu Q, Xie W, et al. Serum fatty acid profiles and potential biomarkers of ankylosing spondylitis determined by gas chromatography-mass spectrometry and multivariate statistical analysis. Biomed Chromatogr. 2015 Apr; 29(4):604-611.

586. Doğan HO, Şenol O, Karadağ A, Yıldız SN. Metabolomic profiling in ankylosing spondylitis using time-of-flight mass spectrometry. Clinical Nutrition ESPEN. 2022; 50:124-132.

587. Fischer R, Trudgian DC, Wright C, Thomas G, Bradbury LA, Brown MA, et al. Discovery of candidate serum proteomic and metabolomic biomarkers in ankylosing spondylitis. Mol Cell Proteomics. 2012 Feb; 11(2):M111 013904.

588. Gao P, Lu C, Zhang F, Sang P, Yang D, Li X, et al. Integrated GC-MS and LC-MS plasma metabonomics analysis of ankylosing spondylitis. Analyst. 2008 Sep; 133(9):1214-1220.

589. He Z, Wang M, Li H, Wen C. GC-MS-based fecal metabolomics reveals gender-attributed fecal signatures in ankylosing spondylitis. Sci Rep. 2019 Mar 7; 9(1):3872.

590. Jiang M, Chen T, Feng H, Zhang Y, Li L, Zhao A, et al. Serum metabolic signatures of four types of human arthritis. J Proteome Res. 2013 Aug 2; 12(8):3769-3779.

591. Bogunia‐kubik K, Wojtowicz W, Swierkot J, Mielko KA, Qasem B, Wielińska J, et al. Disease differentiation and monitoring of anti‐tnf treatment in rheumatoid arthritis and spondyloarthropathies. International Journal of Molecular Sciences. 2021; 22(14).

592. Li H, Wang L, Zhu J, Xiao J, Yang H, Hai H, et al. Diagnostic serum biomarkers associated with ankylosing spondylitis. Clin Exp Med. 2023 Sep; 23(5):1729-1739.

593. Lv L, Jiang H, Yan R, Xu D, Wang K, Wang Q, et al. The Salivary Microbiota, Cytokines, and Metabolome in Patients with Ankylosing Spondylitis Are Altered and More Proinflammatory than Those in Healthy Controls. mSystems. 2021 Jun 29; 6(3):e0117320.

594. Eryavuz Onmaz D, Sivrikaya A, Isik K, Abusoglu S, Albayrak Gezer I, Humeyra Yerlikaya F, et al. Altered kynurenine pathway metabolism in patients with ankylosing spondylitis. Int Immunopharmacol. 2021 Oct; 99:108018.

595. Onmaz DE, Isik K, Sivrikaya A, Abusoglu S, Gezer İA, Abusoglu G, et al. Determination of serum methylarginine levels by tandem mass spectrometric method in patients with ankylosing spondylitis. Amino Acids. 2021; 53(9):1329-1338.

596. Ou J, Xiao M, Huang Y, Tu L, Chen Z, Cao S, et al. Serum Metabolomics Signatures Associated With Ankylosing Spondylitis and TNF Inhibitor Therapy. Frontiers in Immunology. 2021; 12.

597. Shao T-j, He Z-x, Xie Z-j, Li H-c, Wang M-j, Wen C-p. Characterization of ankylosing spondylitis and rheumatoid arthritis using 1H NMR-based metabolomics of human fecal extracts. Metabolomics. 2016; 12(4).

598. Stoll ML, Kumar R, Lefkowitz EJ, Cron RQ, Morrow CD, Barnes S. Fecal metabolomics in pediatric spondyloarthritis implicate decreased metabolic diversity and altered tryptophan metabolism as pathogenic factors. Genes Immun. 2016 Dec; 17(7):400-405.

599. Wang W, Yang GJ, Zhang J, Chen C, Jia ZY, Li J, et al. Plasma, urine and ligament tissue metabolite profiling reveals potential biomarkers of ankylosing spondylitis using NMR-based metabolic profiles. Arthritis Res Ther. 2016 Oct 22; 18(1):244.

600. Zhang S, Fan Z, Ouyang Z, Sun H, Song Y, Yu H, et al. Purine metabolites promote ectopic new bone formation in ankylosing spondylitis. Int Immunopharmacol. 2023 Mar; 116:109810.

601. Zhou Y, Zhang X, Chen R, Han S, Liu Y, Liu X, et al. Serum amino acid metabolic profiles of ankylosing spondylitis by targeted metabolomics analysis. Clin Rheumatol. 2020 Aug; 39(8):2325-2336.

602. Du Q, Wang X, Chen J, Wang Y, Liu W, Wang L, et al. Machine learning encodes urine and serum metabolic patterns for autoimmune disease discrimination, classification and metabolic dysregulation analysis. Analyst. 2023 Sep 11; 148(18):4318-4330.

603. Forgerini M, Urbano G, De Nadai TR, Batah SS, Fabro AT, De Carvalho Mastroianni P. The role of CYP2C9*2, CYP2C9*3 and VKORC1-1639 variants on the susceptibility of upper gastrointestinal bleeding: A full case-control study. J Pharm Pharm Sci. 2023; 26:11136.

604. Forgerini M, Urbano G, de Nadai TR, Batah SS, Fabro AT, Mastroianni PC. Genetic Variants in PTGS1 and NOS3 Genes Increase the Risk of Upper Gastrointestinal Bleeding: A Case-Control Study. Front Pharmacol. 2021; 12:671835.

605. Groza I, Matei D, Tantau M, Trifa AP, Crisan S, Vesa SC, et al. VKORC1-1639 G>A Polymorphism and the Risk of Non-Variceal Upper Gastrointestinal Bleeding. J Gastrointestin Liver Dis. 2017 Mar; 26(1):13-18.

606. Figueiras A, Estany-Gestal A, Aguirre C, Ruiz B, Vidal X, Carvajal A, et al. CYP2C9 variants as a risk modifier of NSAID-related gastrointestinal bleeding: a case-control study. Pharmacogenet Genomics. 2016 Feb; 26(2):66-73.

607. Ishihara M, Ohmiya N, Nakamura M, Funasaka K, Miyahara R, Ohno E, et al. Risk factors of symptomatic NSAID-induced small intestinal injury and diaphragm disease. Aliment Pharmacol Ther. 2014 Sep; 40(5):538-547.

608. Musumba CO, Jorgensen A, Sutton L, Van Eker D, Zhang E, O'Hara N, et al. CYP2C19*17 gain-of-function polymorphism is associated with peptic ulcer disease. Clin Pharmacol Ther. 2013 Feb; 93(2):195-203.

609. Carbonell N, Verstuyft C, Massard J, Letierce A, Cellier C, Deforges L, et al. CYP2C9*3 Loss-of-Function Allele Is Associated With Acute Upper Gastrointestinal Bleeding Related to the Use of NSAIDs Other Than Aspirin. Clin Pharmacol Ther. 2010 Jun; 87(6):693-698.

610. Blanco G, Martinez C, Ladero JM, Garcia-Martin E, Taxonera C, Gamito FG, et al. Interaction of CYP2C8 and CYP2C9 genotypes modifies the risk for nonsteroidal anti-inflammatory drugs-related acute gastrointestinal bleeding. Pharmacogenet Genomics. 2008 Jan; 18(1):37-43.

611. Ma J, Yang XY, Qiao L, Liang LQ, Chen MH. CYP2C9 polymorphism in non-steroidal anti-inflammatory drugs-induced gastropathy. J Dig Dis. 2008 May; 9(2):79-83.

612. Pilotto A, Seripa D, Franceschi M, Scarcelli C, Colaizzo D, Grandone E, et al. Genetic susceptibility to nonsteroidal anti-inflammatory drug-related gastroduodenal bleeding: role of cytochrome P450 2C9 polymorphisms. Gastroenterology. 2007 Aug; 133(2):465-471.

613. Vonkeman HE, van de Laar MA, van der Palen J, Brouwers JR, Vermes I. Allele variants of the cytochrome P450 2C9 genotype in white subjects from The Netherlands with serious gastroduodenal ulcers attributable to the use of NSAIDs. Clin Ther. 2006 Oct; 28(10):1670-1676.

614. Skarke C, Reus M, Schmidt R, Grundei I, Schuss P, Geisslinger G, et al. The cyclooxygenase 2 genetic variant -765G>C does not modulate the effects of celecoxib on prostaglandin E2 production. Clin Pharmacol Ther. 2006 Dec; 80(6):621-632.

615. Martinez C, Blanco G, Ladero JM, Garcia-Martin E, Taxonera C, Gamito FG, et al. Genetic predisposition to acute gastrointestinal bleeding after NSAIDs use. Br J Pharmacol. 2004 Jan; 141(2):205-208.

616. Martin JH, Begg EJ, Kennedy MA, Roberts R, Barclay ML. Is cytochrome P450 2C9 genotype associated with NSAID gastric ulceration? Br J Clin Pharmacol. 2001 Jun; 51(6):627-630.

617. Ricart E, Taylor WR, Loftus EV, O'Kane D, Weinshilboum RM, Tremaine WJ, et al. N-acetyltransferase 1 and 2 genotypes do not predict response or toxicity to treatment with mesalamine and sulfasalazine in patients with ulcerative colitis. Am J Gastroenterol. 2002 Jul; 97(7):1763-1768.

618. Chen M, Xia B, Chen B, Guo Q, Li J, Ye M, et al. N-acetyltransferase 2 slow acetylator genotype associated with adverse effects of sulphasalazine in the treatment of inflammatory bowel disease. Can J Gastroenterol. 2007 Mar; 21(3):155-158.

619. Hou ZD, Xiao ZY, Gong Y, Zhang YP, Zeng QY. Arylamine N-acetyltransferase polymorphisms in Han Chinese patients with ankylosing spondylitis and their correlation to the adverse drug reactions to sulfasalazine. BMC Pharmacol Toxicol. 2014 Nov 21; 15:64.

620. Tanaka E, Taniguchi A, Urano W, Nakajima H, Matsuda Y, Kitamura Y, et al. Adverse effects of sulfasalazine in patients with rheumatoid arthritis are associated with diplotype configuration at the N-acetyltransferase 2 gene. J Rheumatol. 2002 Dec; 29(12):2492-2499.

621. Sabbagh N, Delaporte E, Marez D, Lo-Guidice JM, Piette F, Broly F. NAT2 genotyping and efficacy of sulfasalazine in patients with chronic discoid lupus erythematosus. Pharmacogenetics. 1997 Apr; 7(2):131-135.

622. Tanigawara Y, Kita T, Aoyama N, Gobara M, Komada F, Sakai T, et al. N-acetyltransferase 2 genotype-related sulfapyridine acetylation and its adverse events. Biol Pharm Bull. 2002 Aug; 25(8):1058-1062.

623. Kumagai S, Komada F, Kita T, Morinobu A, Ozaki S, Ishida H, et al. N-acetyltransferase 2 genotype-related efficacy of sulfasalazine in patients with rheumatoid arthritis. Pharm Res. 2004 Feb; 21(2):324-329.

624. Taniguchi A, Urano W, Tanaka E, Furihata S, Kamitsuji S, Inoue E, et al. Validation of the associations between single nucleotide polymorphisms or haplotypes and responses to disease-modifying antirheumatic drugs in patients with rheumatoid arthritis: a proposal for prospective pharmacogenomic study in clinical practice. Pharmacogenet Genomics. 2007 Jun; 17(6):383-390.

625. Wiese MD, Alotaibi N, O'Doherty C, Sorich MJ, Suppiah V, Cleland LG, et al. Pharmacogenomics of NAT2 and ABCG2 influence the toxicity and efficacy of sulphasalazine containing DMARD regimens in early rheumatoid arthritis. Pharmacogenomics J. 2014 Aug; 14(4):350-355.

626. Ducourau E, Mulleman D, Paintaud G, Miow Lin DC, Lauferon F, Ternant D, et al. Antibodies toward infliximab are associated with low infliximab concentration at treatment initiation and poor infliximab maintenance in rheumatic diseases. Arthritis Res Ther. 2011 Jun 27; 13(3):R105.

627. Plasencia C, Pascual-Salcedo D, Garcia-Carazo S, Lojo L, Nuno L, Villalba A, et al. The immunogenicity to the first anti-TNF therapy determines the outcome of switching to a second anti-TNF therapy in spondyloarthritis patients. Arthritis Res Ther. 2013 Jul 26; 15(4):R79.

628. Paramarta JE, Baeten DL. Adalimumab serum levels and antidrug antibodies towards adalimumab in peripheral spondyloarthritis: no association with clinical response to treatment or with disease relapse upon treatment discontinuation. Arthritis Res Ther. 2014 Jul 29; 16(4):R160.

629. Park W, Yoo DH, Miranda P, Brzosko M, Wiland P, Gutierrez-Urena S, et al. Efficacy and safety of switching from reference infliximab to CT-P13 compared with maintenance of CT-P13 in ankylosing spondylitis: 102-week data from the PLANETAS extension study. Ann Rheum Dis. 2017 Feb; 76(2):346-354.

630. Gehin JE, Goll GL, Warren DJ, Syversen SW, Sexton J, Strand EK, et al. Associations between certolizumab pegol serum levels, anti-drug antibodies and treatment response in patients with inflammatory joint diseases: data from the NOR-DMARD study. Arthritis Res Ther. 2019 Nov 29; 21(1):256.

631. Ducourau E, Rispens T, Samain M, Dernis E, Le Guilchard F, Andras L, et al. Methotrexate effect on immunogenicity and long-term maintenance of adalimumab in axial spondyloarthritis: a multicentric randomised trial. RMD Open. 2020 Jan; 6(1).

632. Park W, Hrycaj P, Jeka S, Kovalenko V, Lysenko G, Miranda P, et al. A randomised, double-blind, multicentre, parallel-group, prospective study comparing the pharmacokinetics, safety, and efficacy of CT-P13 and innovator infliximab in patients with ankylosing spondylitis: the PLANETAS study. Ann Rheum Dis. 2013 Oct; 72(10):1605-1612.

633. Kneepkens EL, Wei JC, Nurmohamed MT, Yeo KJ, Chen CY, van der Horst-Bruinsma IE, et al. Immunogenicity, adalimumab levels and clinical response in ankylosing spondylitis patients during 24 weeks of follow-up. Ann Rheum Dis. 2015 Feb; 74(2):396-401.

634. Jung SM, Kim HS, Kim HR, Kim NY, Lee JH, Kim J, et al. Immunogenicity of anti-tumour necrosis factor therapy in Korean patients with rheumatoid arthritis and ankylosing spondylitis. Int Immunopharmacol. 2014 Jul; 21(1):20-25.

635. Hoxha A, Calligaro A, Tonello M, Ramonda R, Carletto A, Paolazzi G, et al. The clinical relevance of early anti-adalimumab antibodies detection in rheumatoid arthritis, ankylosing spondylitis and psoriatic arthritis: A prospective multicentre study. Joint Bone Spine. 2016 Mar; 83(2):167-171.

636. de Vries MK, Wolbink GJ, Stapel SO, de Vrieze H, van Denderen JC, Dijkmans BA, et al. Decreased clinical response to infliximab in ankylosing spondylitis is correlated with anti-infliximab formation. Ann Rheum Dis. 2007 Sep; 66(9):1252-1254.

637. Braun J, Deodhar A, Dijkmans B, Geusens P, Sieper J, Williamson P, et al. Efficacy and safety of infliximab in patients with ankylosing spondylitis over a two-year period. Arthritis Rheum. 2008 Sep 15; 59(9):1270-1278.

638. Arends S, Lebbink HR, Spoorenberg A, Bungener LB, Roozendaal C, van der Veer E, et al. The formation of autoantibodies and antibodies to TNF-alpha blocking agents in relation to clinical response in patients with ankylosing spondylitis. Clin Exp Rheumatol. 2010 Sep-Oct; 28(5):661-668.

639. Su J, Li M, He L, Zhao D, Wan W, Liu Y, et al. Comparison of the Efficacy and Safety of Adalimumab (Humira) and the Adalimumab Biosimilar Candidate (HS016) in Chinese Patients with Active Ankylosing Spondylitis: A Multicenter, Randomized, Double-Blind, Parallel, Phase III Clinical Trial. BioDrugs. 2020 Jun; 34(3):381-393.

640. Mahmoud I, Rouached L, Ben Tekaya A, Saidane O, Bouden S, Jradi S, et al. Immunogenicity of antitumor necrosis factor therapy in patients with spondyloarthritis. Drug Metabolism and Drug Interactions. 2021; 36(1):25-32.

641. de Vries MK, van der Horst-Bruinsma IE, Nurmohamed MT, Aarden LA, Stapel SO, Peters MJ, et al. Immunogenicity does not influence treatment with etanercept in patients with ankylosing spondylitis. Ann Rheum Dis. 2009 Apr; 68(4):531-535.

642. Balsa A, Sanmarti R, Rosas J, Martin V, Cabez A, Gomez S, et al. Drug immunogenicity in patients with inflammatory arthritis and secondary failure to tumour necrosis factor inhibitor therapies: the REASON study. Rheumatology (Oxford). 2018 Apr 1; 57(4):688-693.

643. Arstikyte I, Kapleryte G, Butrimiene I, Venalis A, Shi G. Influence of immunogenicity on the efficacy of long-term treatment with TNF α blockers in rheumatoid arthritis and spondyloarthritis patients. BioMed Research International. 2015; 2015.

644. Plasencia C, Pascual-Salcedo D, Nuno L, Bonilla G, Villalba A, Peiteado D, et al. Influence of immunogenicity on the efficacy of longterm treatment of spondyloarthritis with infliximab. Ann Rheum Dis. 2012 Dec; 71(12):1955-1960.

645. Meric JC, Mulleman D, Ducourau E, Lauferon F, Miow Lin DC, Watier H, et al. Therapeutic drug monitoring of infliximab in spondyloarthritis: an observational open-label study. Ther Drug Monit. 2011 Aug; 33(4):411-416.
