# Supplementary material for: A guideline on biomarkers in the diagnosis and evaluation in axial spondyloarthritis
Source: Front Immunol. 2024 Oct 30;15:1394148. doi: 10.3389/fimmu.2024.1394148 (PMC11557325; doi:10.3389/fimmu.2024.1394148)
Supplement: Supplementary file 6 [file Table6.docx]

**SUPPLEMENTARY APPENDIX 6: Evidence Report**

**Question 1**: **HLA-B27**

We found 13 cross-sectional studies and 29 cohort studies addressing this question.

The evidence shows:

1.HLA-B27 contributed approximately 20% of the overall genetic risk to axial spondyloarthritis.[1]

2.HLA-B27 testing is still a fundamental test in the diagnostic pathway of axial spondyloarthritis. Individuals with HLA-B27 positivity had a significantly higher risk of developing ankylosing spondylitis (OR=8.19), and 82.1% of patients with ankylosing spondylitis were HLA-B27 positive.[2] The clinical arm of Assessment in SpondyloArthritis international Society (ASAS) classification criteria for axial spondyloarthritis deemed HLA-B27 a prerequisite.[3]

3.HLA-B27 positive patients are more likely to develop to radiographic axial spondyloarthritis[4], but HLA-B27 positivity per se does not predict syndesmophyte formation or the worsening of mSASSS score.

4.HLA-B27 positivity is associated with the phenotype of disease. HLA-B27 patients are more likely to develop uveitis, peripheral arthritis and hip involvement.[2]

5.Patients without HLA-B27 positivity might be less responsive to treatment of TNF-a inhibitors than HLA-B27 positive patients.[5, 6] However, this statement must be interpreted with caveat, since this phenomenon could be attributed to the fact that HLA-B27 positive patients could get an earlier diagnosis. However, efficacy of IL-17 inhibitors seems to be independent of HLA-B27 positivity.

The quality of evidence is HIGH.

**Table: Evidence profile**

| Certainty assessment | | | | | | | Summary of findings | |
| --- | --- | --- | --- | --- | --- | --- | --- | --- |
| No of participants  (studies)  Follow-up | Risk of bias | Inconsistency | Indirectness | Imprecision | Publication bias | Overall certainty of evidence | Pooled Result (95%CI) | Brief Summary |
| Question 1: | | | | | | | | |
| 42 studies (13 cross-sectional studies and 29 cohort studies) | Not Serious | Not serious | Not serious | Not serious | Not serious | ⨁⨁⨁⨁  HIGH | \ | HLA-B27 contributed approximately 20% of the overall genetic risk to axial spondyloarthritis. The clinical arm of Assessment in ASAS classification criteria for axial spondyloarthritis deemed HLA-B27 a prerequisite. HLA-B27 positive patients are more likely to develop to radiographic axial spondyloarthritis and more likely to develop uveitis, peripheral arthritis and hip involvement. |

**Table: Studies addressing the value of HLA-B27 as biomarkers of AS**

| Study | Year | Design | Population | Result |
| --- | --- | --- | --- | --- |
| Jajić[7] | 1979 | Cross-sectional | 652 LBP patients | The HLA-B27 antigen was positive in 276(42.4%) of whole 652 patients with low back pain of various origins and was significantly higher than control group(p<0.001).128 HLA-B27(+) patients are diagosed as AS. |
| Sadowska-Wróblewska[8] | 1983 | cohort | 70 eAS | Back pain had high sensitivity index in eAS(sensitivity:93%,specificity:19%).HLA B27 antigen was a parameter both highly sensitive and specific in eAS and PDD group(sensitivity:83%,specificity:95%). |
| Deesomchok[9] | 1985 | cohort | 61 SpA | 51(91.7%) had HLA-B27(+). |
| Sampaio-Barros[10] | 2001 | cohort | 68 uSpA | HLA-B27 was positive in 37 patients (54%). The first disease manifestations were ILBP (49%). |
| Brandt[11] | 2007 | Cohort | 350 BP patients | 159 back patin patients whose duration >3months or age <45 year old are diagosed as axSpA,including 50.3% AS and 49.7% preradiographic axial SpA.HLA-B27 was positive in 35.4% of all(n=57) , 36.6% of IBP(n=59).The mean symptom duration from onset of back pain to the time diagnosis of definite axSpA ,AS,preradiographic axSpA and possible SpA was 7.7 (range 0.1–45, median 5) years, 10.7 (range 0.3–45, median 8) years,4.6 (range 0.1–35, median 2) years and 6.6 (range 0.1–20, median 6) years. |
| Dincer[12] | 2008 | cohort | 111 AS | The average diagnosis delay was 5. 3±3.5 in HLA B 27+ AS patients, whereas it was 9.2±7.7 in HLA B 27(-)AS patients ( p= 0.037). HLA B27 status may diminish the average diagnosis time and also diagnosis delay.We found that diagnosis delay in patients having IBP at disease onset was lower than those not having IBP (3.28±3.32, 8.57±8.54, respectively,P=0.001). |
| Bennett[4] | 2008 | cohort | 55 IBP | Combination of severe sacroiliitis and HLA–B27 positivity has a high specificity for development of AS,Testing for HLA-B27 among patients (of European descent) with IBP who do not show unequivocal radiological sacroiliitis can play a central part in the diagnostic investigation of axial SpA. |
| Nazarinia[13] | 2009 | Cross-sectional | 98 AS, including 72 HLA-B27(+) and 26HLA-B27(-) | Positive HLA-B27 was associated with hip involvement (P = 0.042) and adult onset (P = 0.035). Adult-onset AS was associated with HLA-B27 (P = 0.005). |
| Liao[14] | 2009 | cohort | 10921 | Prevalence of LBP is 7.21%.The prevalence of axial SpA is 0.782%. HLA-B27 positive rate was significant higher in the SpA patients (82.67%) than in the other LBP patients (11.65%). |
| Aggarwal[15] | 2009 | cohort | 70 AS | HLA B27 was present in 65(92.9%) of patients.There was no significant difference in the laboratory of HLA B27 characteristics between the genders. |
| Liu[16] | 2010 | Cohort | 1016 suspected of AS | HLA-B27(+) was detected in 113 (46.7%) patients of whom 242 (23.8%) had low back pain and stiffness>3 months. During 1 year follow-up,69 patients(67.6%) were diagnosed as AS.Compared with other symptoms in total 1016 suspected of ankylosing spondylitis,low back pain and stiffness patients with HLA-B27(+) had higher incidence of AS( p<0.01) |
| van Onna[17] | 2011 | cohort | 68 IBP | HLA-B27 positivity (OR 5.1, 95% CI 1.9 to 13.6, p<0.001) independently determined the likelihood of a positive MRI at any time point. |
| Liao[18] | 2011 | Cross-sectional | 135 HLA-B27(+)/57 HLA-B27(-) | More HLA-B27-positive than HLA-B27-negative patients fulfilled the ESSG(68.1 vs52.6,p<0.05,OR:1.93), Amor(78.5 vs 33.8,p<0.001,OR:7.31), and ASAS criteria(77.8 vs 52.6,p<0.001,OR:3.55).HLA-B27 is more useful for classification than MRI of the sacroiliac joint. |
| Chung[19] | 2011 | cohort | 708 IBP | HLA-B27 was positive in 61.5% of the patients.In early axial SpA, HLA-B27 is associated with earlier onset of IBP, less delay in diagnosis, axial infl ammation (spine and SIJ), radiographic damage of the SIJ, decreased disease activity and lower frequency of psoriasis. |
| De Carvalho[20] | 2012 | cohort | 1505 SpA | HLA-B27 (tested in 728 patients) was significantly more frequent in males than female(72.4% vs. 59.3%; p=0.001) |
| van den Berg[21] | 2013 | cohort | 157 CBP | The finding showed HLA-B27(+) and IBP both are belpful to screen for most axSpA patients with the entry criteria for the ASAS axSpA criteria |
| Qi[22] | 2013 | Cross-sectional | 846 AS/959 HC | 741 AS patients (87.6%) and 39 healthy subjects (4.1%) tested positive for HLA-B27.Inflammatory low back pain was the most prevalent symptom (n =788, 93.1%).The study showed that compared with HLA-B*2704-positive AS patients, HLA-B*2705-positive AS patients had an older age of onset and higher risk of developing uveitis and dactylitis. |
| Peláez-Ballestas[23] | 2013 | cohort | 758 individuals | The prevalence rates of Nontraumatic BP,IBP,SpA and AS were 14.6%,1.3% ,0.6%and 0.1%, respectively.HLA-B27 positive(p=0.009)and pain NRS(p=0.004) had significant differences between probable and definite IBP. |
| Tomero[24] | 2014 | cross-sectional | 775 patients | 75% Of 775 patients which included 538 SpA had IBP.299(56%) in SpA group were HLA-B27(+).HLA-B27 positive( (p<0.001, OR 5.9, 95% CI 3.6-9.5)and ILBP (p<0.001, OR 3.8, 95% CI 2.4-6.0) both had a major influence on the ASAS classification of SpA |
| Kassimos[25] | 2014 | cohort | 347184 | A total of285 AS cases were diagnosed certain AS(179) and probable AS(106).HLA-B27 antigen was found in 257 patients (90 %, 95 % C.I. 86–93 %). |
| Nakashima[26] | 2016 | Cohort | 72 AS | Of 80 delayed diagnosis patients,low back pain is the most common initial sympton.HLA B27-positive patients had significantly earlier symptom onset than those with negative patients (22.1 years old versus 32.0 years old, p=0.0263). |
| Burgos-Vargas[27] | 2016 | cross-sectional | nr-axSpA (n = 266)  AS (n = 491)  Other IBP (n = 157) | HLA-B27 test results were available for 71.05 % of those with nr-axSpA, and 82.78 % of AS had a positive test result. |
| Bautista-Molano[28] | 2016 | cohort | 581 SpA | 439(75.6%) had symptom of back pain.The rate ofHLA-B27 positivity in the group ofpatients with a clinical diagnosis ofSpAwho did not fulfill the ASAS SpA criteria was low and was lower than in those patients with a clinical diagnosis ofSpA not fulfilling other criteria sets for SpA.rheumatologists did not pay too much attention to the presence or absence of HLA-B27 when considering a clinical diagnosis of SpA, |
| Bandinelli[29] | 2016 | cohort | 135 AS | the inflammatory low back pain (118/135 [87.4 %]) was the most frequent manifestation at onset and had a higher delayed diaginosis (p=0.0021).112/135 (82.9 %) had HLA-B27(+).Between HLA B27-positive AS patients, women had a lower delayed diaginosis than men. |
| Arnbak[30] | 2016 | cohort | 759 IBP | 86(11%) patients included met ASAS criteria for diagnosis of axial SpA.SpA group(47%) had higher rate of HLA-B27 positive than non-SpA(6%). |
| Fırat[31] | 2017 | Cross-sectional | 163 AS | 150(92%) of all AS patients had IBP,including 115(93%) HLA-B27-positive and 43(89.5%) HLA-B27-negetive(0.528).presence of syndesmophytes was significantly associated with HLA-B27 positivity (22.6%, 8.33%, respectively; p=0.044). |
| Ez-Zaitouni[32] | 2017 | cohort | 500 CBP | 250(50%) was diagnosed as axSpA.329(66%) were classified as IBP and 198(40%) were HLA-B27(+).HLA-B27 positivity and positive imaging were both independent determinants of axSpA diagnosis |
| Tong[33] | 2018 | cohort | 3770 students | Prevalences of CLBP and IBP among students were 19.39 and 2.94%, respectively.9 of 111 students with IBP had HLA-B27(+).Prevalence ofaxial SpA was 0.34%. |
| Endo[34] | 2019 | cohort | 114 SpA | 50(43.9%) of all SpA had IBP, especially in EOSpA(58.2%). IBP is an independent risk factor for early-onset SpA (p=0.021, OR 0.288, 95% CI 0.097-0.857). |
| Passalent[35] | 2022 | cohort | 405 LBP | 57(14.1%) patients were HLA-B27(+).This study considered HLA-B27 alone is not a useful second screening tool for axSpA(sensitivity 28d,specificity 94,PPV 70,NPV 71). |
| Edara[36] | 2022 | Cross-sectional | 16 rSpA/12 nrSpA | HLA‑B27 positivity was seen in 24 patients (85.7%).HLA-B27(+) is no significant difference between the radiographic axSpA and the non-radiographic group. |
| Chung[37] | 2022 | cross-sectional | 447 axSpA  122 without IBP | Compared with those non-IBP,447 axSpA had earlier age of back pain onset(32.8±13 vs 40.0±14.8,p<0.001),longer duration of back pain(12.1±11.5 vs 8.1±9.0,p<0.001) and higher rate of HLA-B27 positive(81.9% vs 8.9%,p<0.001). |

**Table: Studies addressing the value of HLA-B27 as a biomarker for indicating disease activity**

| Study | Year | Design | Population | Result |
| --- | --- | --- | --- | --- |
| Puhakka[38] | 2004 | Cross-sectional | 26 HLA-B27(+) SpA/15 HLA-B27(-) SpA | HLA-B27(+) SpA had significantly higher joint destruction scores (7.8 vs 4.3, P<0.05) and activity scores (13.4 vs 5.0, P<0.01) than HLA-B27(-). |
| Ma[39] | 2012 | Cross-sectional | 109 AS (Southern) 101 AS (Northern) | HLA-B27 positive was higher in the southern group than in northern(96.5% vs. 83.5%,p=0.0011) |
| Londono[40] | 2012 | Cross-sectional | 62 SpA/46HC | 26(41.9%) of 62 SpA had HLA-B27(+).US-CRP (P = 0.04), IL-6 (P = 0.003), IL-1α (P = 0.03), and LBP (P = 0.03) levels were associated with presence of HLA-B27, infl ammatory back pain, and arthritis. |
| Weiss[41] | 2016 | Cross-sectional | 40 JSpA/15 HLA-B27(-) SpA | Patients with positive findings on MRI were more likely to be HLA-B27 positive (p=0.01).There was a significantly higher prevalence of HLA-B27 positivity in those children with sacroiliitis(sensitivity:0.88 (0.65, 1.00),specificity:0.69 (0.52, 0.86)). |

**Table: Studies addressing the value of HLA-B27 as a biomarker for predicting therapeutic effect of bDMARDs**

| Study | Year | Design | Population | Result |
| --- | --- | --- | --- | --- |
| Baraliakos[42] | 2014 | Cohort | 867 ETN-treated AS | HLA-B27(+) at baseline was associated with significantly higher odds of responding to enacept-treatment at week 12. That means HLA-B27 positive status may be a predictor of better treatment response. |
| Al-Saleh[43] | 2022 | Cohort | 309 axSpA | 212(68.6%) of total had IBP and 95(30.8%) are HLA-B27 positive.HLA-B27 positive patients are more likely hard to achieve remission or LDA( p = 0.03, OR 2.0, 95% CI 1.1-3.7). |

**Table: Studies addressing the value of HLA-B27 as a biomarker for predicting radiological progression**

| Study | Year | Design | Population | Result |
| --- | --- | --- | --- | --- |
| Huerta-Sil[44] | 2006 | Cohort | 62 uSpA | The study showed that at follow up of 3-5 years,21 patients (42%) had developed ankylosing spondylitis.Low grade radiographic sacroiliitis is a prognostic factor for AS originally classified as uSpA.HLA-B27(+) at baseline showed an increased trend to linkage with AS(79.8% vs 59.6%，p=0.14). |
| Sampaio-Barros[45] | 2010 | Cohort | 111 uSpA | In 7 years follow-up,75(67.6%) uSpA patients had ILBP,only 28.8% at onset. HLA-B27 positive (p = 0.035, OR 6.720, 95% CI 11.45-39.43) was one of best predictors of progression to AS,compared to 25 patients of remission (p = 0.035, OR 6.720, 95% CI 11.45-39.43) |
| Bakker[46] | 2019 | Cohort | 188 IBP | 91 patients of all had HLA-B27(+).HLA-B27 positivity determines the likelihood of a positive MRI result.In patients who had a negative baseline MRI result, HLA-B27 status had a significant effect on the likelihood of a positive MRI at follow- up. |
| Lorenzin[47] | 2020 | Cohort | 75 LBP | HLA-B27 positivity appeared one of predictors of radiological damage and activity. |

**Question 2**: **HLA-B27 subtypes**

We found 38 Cross-sectional studies and 2 meta-analysis addressing this question.

The evidence shows:

1.There are approximately 240 subtypes of HLA-B27 currently known.

2.Association with disease has not been established in most subtypes of HLA. However, according our meta-analysis, it could be confirmed that the alleles of HLA-B27:04 and 05 are risk factors of ankylosing spondylitis, while the alleles of HLA-B27:06 and 07 could be protective factors. Association with disease could not be established in the subtype of HLA-B27:09 and 10.

The quality of evidence is MEDIUM.

**Table: Evidence profile**

| Certainty assessment | | | | | | | Summary of findings | |
| --- | --- | --- | --- | --- | --- | --- | --- | --- |
| No of participants  (studies)  Follow-up | Risk of bias | Inconsistency | Indirectness | Imprecision | Publication bias | Overall certainty of evidence | Pooled Result (95%CI) | Brief Summary |
| Question 2: | | | | | | | | |
| 40 studies (38 Cross-sectional studies and 2 meta-analysis) | Not Serious | Not serious | Not serious | Not serious | Not serious | ⨁⨁⨁◯  MEDIUM | \ | The alleles of HLA-B27:04 and 05 are risk factors of ankylosing spondylitis, while the alleles of HLA-B27:06 and 07 could be protective factors. Association with disease could not be established in the subtype of HLA-B27:09 and 10. |

**Table: Studies addressing the value of HLA-B27 subtypes as biomarkers of AS**

| Study | Year | Design | Population | Result |
| --- | --- | --- | --- | --- |
| Yi[48] | 2013 | Cross-sectional | 360 AS, 350 non rheumatic disease | the HLA-B alleles, and identified HLA-B*27:04:01 and B*27:05:02 as two major susceptibility suballeles of HLA-B*27 to AS in Han Chinese population. |
| Siala[49] | 2009 | Cross-sectional | 42AS,100HC | B*2702 and B*2705 subtypes are the unique alleles expressed by AS patients |
| Ren[50] | 1997 | Cross-sectional | 50AS, 45 B27+HC | only 3 subtypes of B27 are found in the Singapore Chinese population: B*2704, B*2705 and B*2706, B*2706 allele has a significant negative association with disease (p = 0.047). Together with recent data indicating the existence of AS "protective" B27 alleles |
| Qi[22] | 2013 | Cross-sectional | 846 AS,959 HC | HLA-B*2706 was more frequent in healthy controls than in AS patients;  Age of onset of disease was significantly earlier in HLA-B*2704-positive patients than in HLA-B*2705-positive patients (20.7 ± 6.7 vs 22.9 ± 8 years, P = 0.028)  compared with HLA-B*2704-positive AS patients, HLA-B*2705-positive AS patients had an older age of onset and higher risk of developing uveitis and dactylitis |
| Pazár[51] | 2010 | Cross-sectional | 297 AS, 200 HC | the B*2705 subtype was significantly associated with AS when we compared the data with HLA-B27 subtypes of healthy individuals |
| Park[52] | 2009 | Cross-sectional | 143 AS,32 B27+ HC | The proportion of B*2705 subtypes was significantly higher in the AS group than the control group (P < 0.01).  The clinical manifestations of this disease do not correlate with subtype distribution. |
| Mou[53] | 2010 | Cross-sectional | 505 AS, 1368 HC | B*2704 was the dominant subtype and was significantly higher in AS patients than that in healthy controls (odds ratio = 3.91, 95% CI: 2.322–6.564).  The rate of B*2704 homozygotes in AS patients was significantly higher than that in controls.  The mean age of onset in patients carrying B*2704 was significantly earlier than that in patients carrying B*2705 in AAS group. |
| Ma[54] | 2006 | Cross-sectional | AS+uSpA: 111+17  HC+( B27+ HC): 169+14 | B*2704 is the predominant subtype in AS patients in Hunan. B*2706 and B*2707 were solely detected in healthy controls. |
| Luo[55] | 2019 | Cross-sectional | 235 AS 261 HC | The frequencies of the main subtypes (HLA-B*2704 and HLA-B*2705) were similar between AS patients and controls  lower back pain was significantly more common in AS patients with HLA-B*2704 and HLA-B*2705 subtypes compared with the HLA-B*27-negative group (P <0.05). Uveitis was significantly less common in HLA-B*2704- and HLAB*2705-positive patients with AS than in HLA-B*27-negative patients with AS (P <0.05) |
| Lopez-Larrea[56] | 1995 | Cross-sectional | 64 AS,9 HC | B*2705, 02, and 04 occur in the AS Mestizo population. B*2705 was the predominant subtype found in this group (93.8%) |
| Lodhi[57] | 2019 | Cross-sectional | 49 AS,18 HC | HLA-B*27:06 was found in control group only. Ther were no significant differences for the distribution of B*27 subtypes between patients and controls (p >0.05). |
| Liu[58] | 2010 | Cross-sectional | 172 AS,142 HC | The frequency of B*2704 in AS group was significantly higher than in the control individuals (P < 0.01). |
| Kchir[59] | 2010 | Cross-sectional | 100 AS,100 HC | B*2702 and B*2705 were the dominant subtypes (32 and 24% respectively) |
| Hou[60] | 2007 | Cross-sectional | 314 AS,71 B27+non AS | the HLA-B*2704 was the predominant subtype with positive association to AS in the Taiwanese population. |
| Harfouch[61] | 2011 | Cross-sectional | 50 AS,217 HC | HLA-B*27 allele was found in 1.4% healthy Syrians and 60% in patients with AS (OR=107, p=0.0001, corrected p=0.003).  The most common HLA-B*27 variants in patients were B*2705,, followed by B*2702. |
| Gonzalez-Roces[62] | 1997 | Cross-sectional | 746 AS,235 HC | B*2705 was the predominant subtype in circumpolar and subarctic areas.  B*2702 was found to be practically restricted to Caucasian populations, showing a higher frequency in Middle-East (Jews) and North Africa (Arabsmerbers) groups. |
| Diyarbakir[63] | 2012 | Cross-sectional | 43 AS,39 HC | Among B27 subtypes, the most common B27 alleles found in the patients and the controls were B*2702 and B*2705.  B*2702 subtype was found predominantly in both patients (48.8%) and controls (46.2%). |
| Cipriani[64] | 2003 | Cross-sectional | 48 AS,55 HC | B*2705 subtype showed significant association with patients being male.  In the healthy controls, the most common subtype was B*2708. |
| Chou[65] | 2003 | Cross-sectional | 55 AS,46 b27+ HC | the Aborigines without AS carrying B2704 showed a significant difference from the Chinese without AS carrying B2704 (p = 0.041). |
| Chavan[66] | 2011 | Cross-sectional | 81 AS,29 b27+ HC | occurrence of AS-associated uveitis was more prevalent in B*2704-positive patients (34.78%, 8/23) compared to patients with allele B*2705 (16.36%, 9/55). (*P* = 0.130). |
| Birinci[67] | 2006 | Cross-sectional | 38 AS,47 b27+ HC | B*2705 was the predominant subtype among both of the patients (71.1%) and controls (68.0%). B*2702 was observed in 26.3% and 32.0% of the patients and controls, respectively.  B*2708 subtype was found in 2.6% of the patients but not among the control group |
| Ben Radhia[68] | 2008 | Cross-sectional | 121 AS,39 b27+ HC | B*2702 and B*2705 were the dominant subtypes followed by B*2707 and B*2703.  No statistically significant differences in the distribution of any allele were found among AS patients compared with controls. |
| Alaez[69] | 2007 | Cross-sectional | 24 AS,51 HC | The only allele conferring risk to AS expression in Israeli Jews was B∗2702 |
| Acar[70] | 2012 | Cross-sectional | 51 AS,948 b27+ non rheumatic disease | B*2705 and B*2702 are the most common HLA-B27 subtypes in AS patients and Turkish population, and the frequency of B*2705 is higher than that of B*2702. These subtypes are not different between patients and controls. |
| Varnavidou-Nicolaidou[71] | 2004 | Cross-sectional | 31 AS，60 b27+ HC | the B*2702 allele has an elevated frequency in the AS patients. The allele B*2707 seems to have a protective role in the population studied |
| Lopez-Larrea[72] | 2006 | Cross-sectional | Spain:71AS,105HC  Portugal:55AS,57HC | No differences are found in the distribution of B27 subtypes among patients with AS and B27-positive matched controls in either the Spanish or Azorean populations |
| Gonzalez[73] | 2002 | Cross-sectional | 89 AS，97 b27+ HC | B*2705 was the most common allele, this being followed by B*2702. No statistically significant differences in the distribution of any allele were found among the different SpA. |
| Díaz-Peña[74] | 2016 | Cross-sectional | 367 AS，462 b27- HC ,549 b27+ HC | B*07:02 allele frequencies were significantly decreased in patients with AS compared to healthy controls (9.5% vs 18.8%, P_BONF_ < 0.01, OR 0.45, 95% CI 0.30–0.70). |
| Chen[75] | 2002 | Cross-sectional | 184 AS,20000 HC | B*2704, B*2705 and B*2707 are the three major B27 alleles positively associated with AS in Taiwanese patients. B*2706 (OR = 0.08, 95% CI = 0.01–0.72) is decreased in AS patients compared to B*2704 (OR = 4.62, 95% CI = 1.84–11.61), B*2705 (OR = 0.38, 95% CI = 0.12–1.20), and B*2707 (OR = 0.29, 95% CI = 0.04–1.92). |
| Yamaguchi[76] | 1996 | Cross-sectional | 20 AS,11HC | HLA-B*2704 and B*2705 are present in the Japanese population, the former being the predominant subtype of HLA-B27. Both subtypes are associated with SpA. |
| Wu[77] | 2020 | Cross-sectional | 318 AS,89HC | the genotype HLA-B27:04 can improve diagnostic accuracy, and patients with HLA-B27:04 had a high risk of arthritis and enthesitis. |
| Van Gaalen[78] | 2012 | Cross-sectional | 929 AS，5990 b27+ HC | Meta-analysis shows a protective effect of HLA-B*2706 on development of AS in HLA-B27 individuals (odds ratio = 0.128, 95% CI = 0.043–0.378, *P*< 0.001). |
| Oguz[79] | 2004 | Cross-sectional | 49 SpA，55HC | The proportion of B*2705 among B27-positive patients and controls was significantly different (P=0.02).  B*2705 and B*2702 were more frequent in Caucasian patients with SpA. |
| Mou[80] | 2015 | Cross-sectional | 145 JAS ，360 AAS | Peripheral arthritis, enthesitis, BASDAI, ESR, and CRP were significantly higher in JAS patients with HLA-B(*)2704 than those with B27-negative.  Enthesitis and ESR were significantly higher in patients with HLA-B(*)2705 than those with B27-negative.  The onset age of HLA-B(*)2715 group was much earlier than the other groups.  The peripheral arthritis, enthesitis, and hip joint involvement in JAS with HLA-B(*)2704 were significantly higher than those in AAS with HLA-B(*)2704. |
| Lopez-Larrea[81] | 1995 | Cross-sectional | India: 45 AS, 17 HC  Thailand: 45 AS, 17 HC | B*2704 is associated with AS in the Thai patients (91% in AS vs. 47% in C; RR = 11.5; EF = 0.83). B*2704 is found with similar frequency in Asian Indians AS patients and controls (41% in AS vs. 41% in C.).  B*2706 is found overrepresented in control populations and absent in AS patients (0% in AS vs. 47% in C.; pc < 10(-6)). The B*2706 could indicate a protective effect of this subtype on AS susceptibility. |
| Kanga[82] | 1996 | Cross-sectional | 24 AS, 17HC | B*2704 is the predominant subtype in the AS group (70.8%) compared to its frequency of 47% in healthy controls (RR = 2.73)  in the undiff SpA group, B*2705 occurred most frequently (73.1%, RR = 3.05). |
| Fouladi[83] | 2009 | Cross-sectional | 40 AS, 17HC | B*2702 and B*2705 were predominant alleles followed by B*2704 and B*2707 |
| Liu[84] | 2010 | Cross-sectional | 130AS，61HC | B*2704 is the predominant and more strongly associated subtype with AS disease.  B*2710 is identified for the first time in the Chinese Han population, although its association with AS could not be determined. |
| Yang[85] | 2014 | meta-analysis | - | B2704 is strongly related to AS as a risk factor.  B2703, B2706 and B2707 might be potential protective factors of AS, especially in Asian populations |
| Lin[2] | 2017 | meta-analysis | - | the prevalence of HLA-B27*02 and HLA-B27*04 could increase the risk of AS |


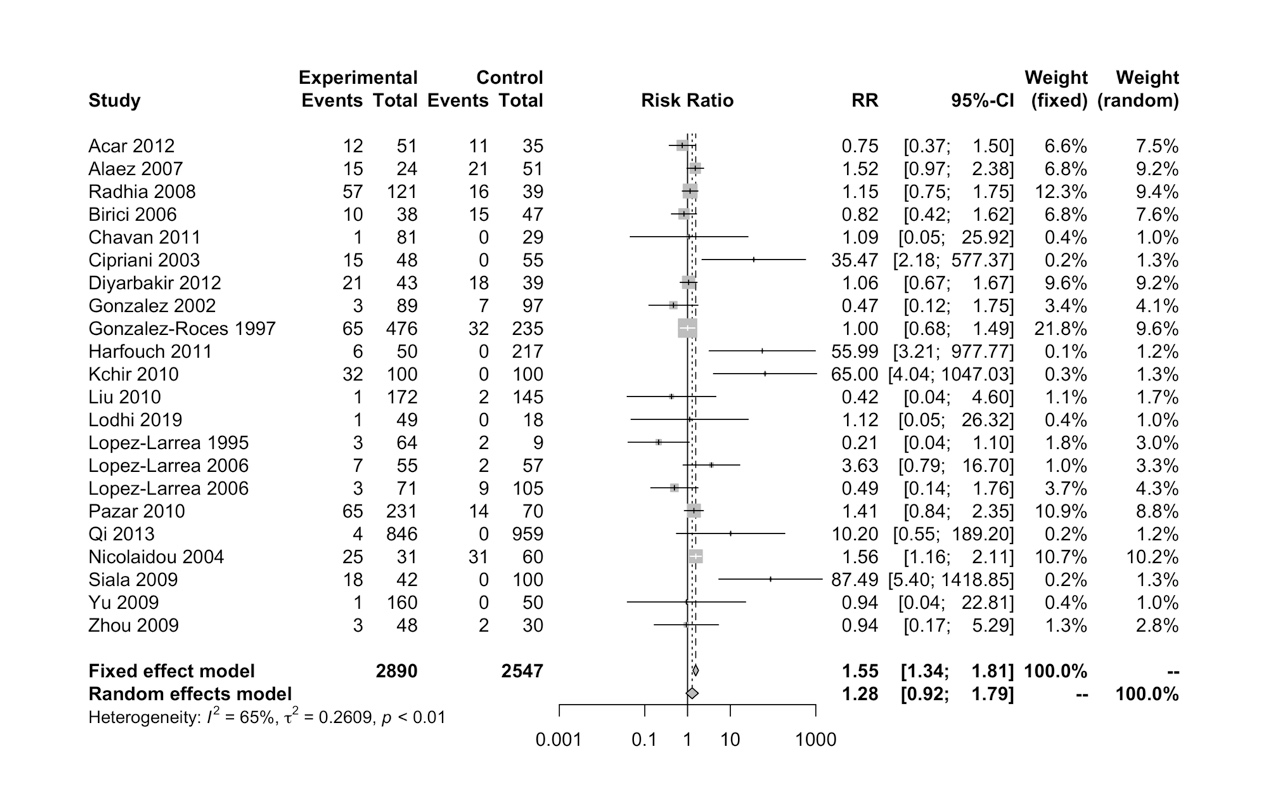


**Figure: Risk of AS in carriers of HLA-B27:02 compared with non-carriers**


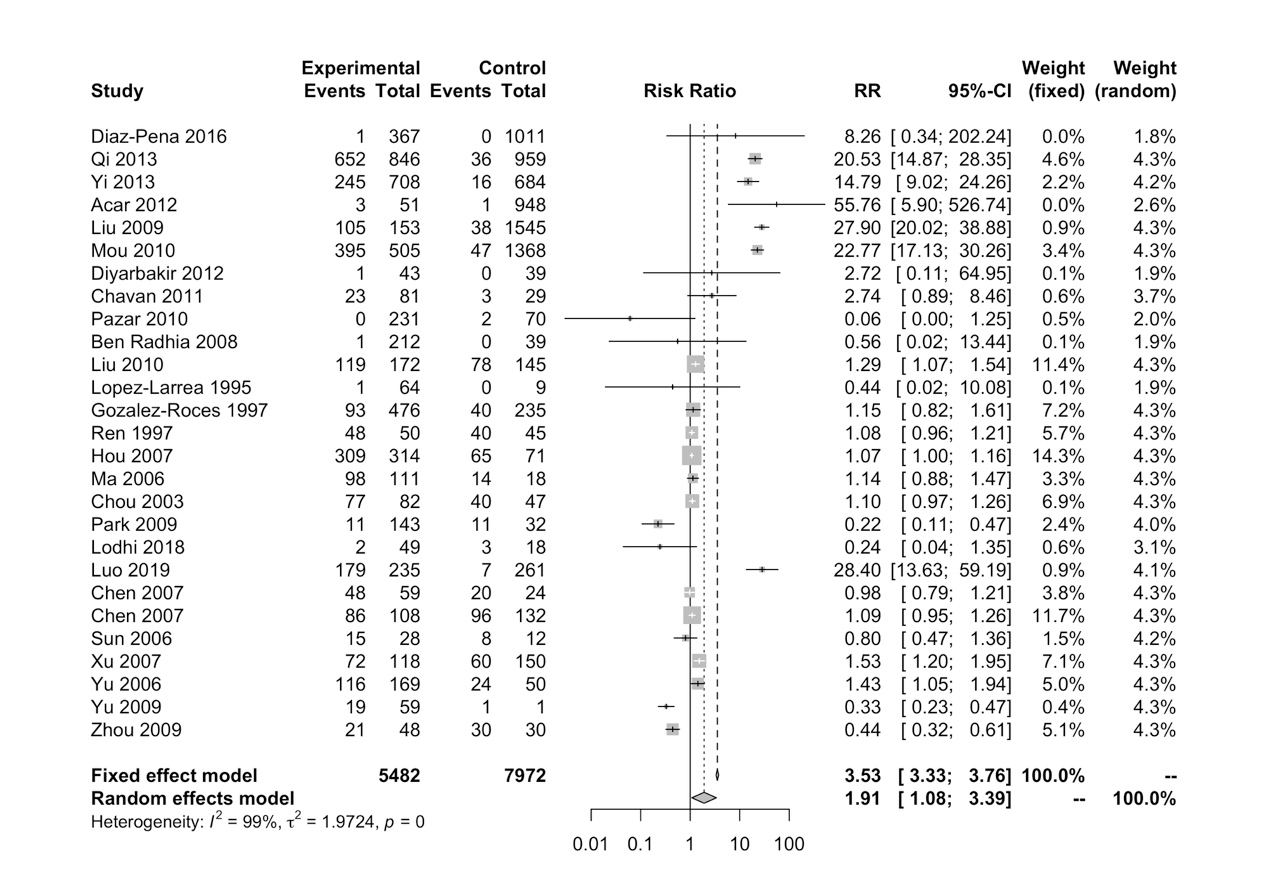


**Figure: Risk of AS in carriers of HLA-B27:04 compared with non-carriers**
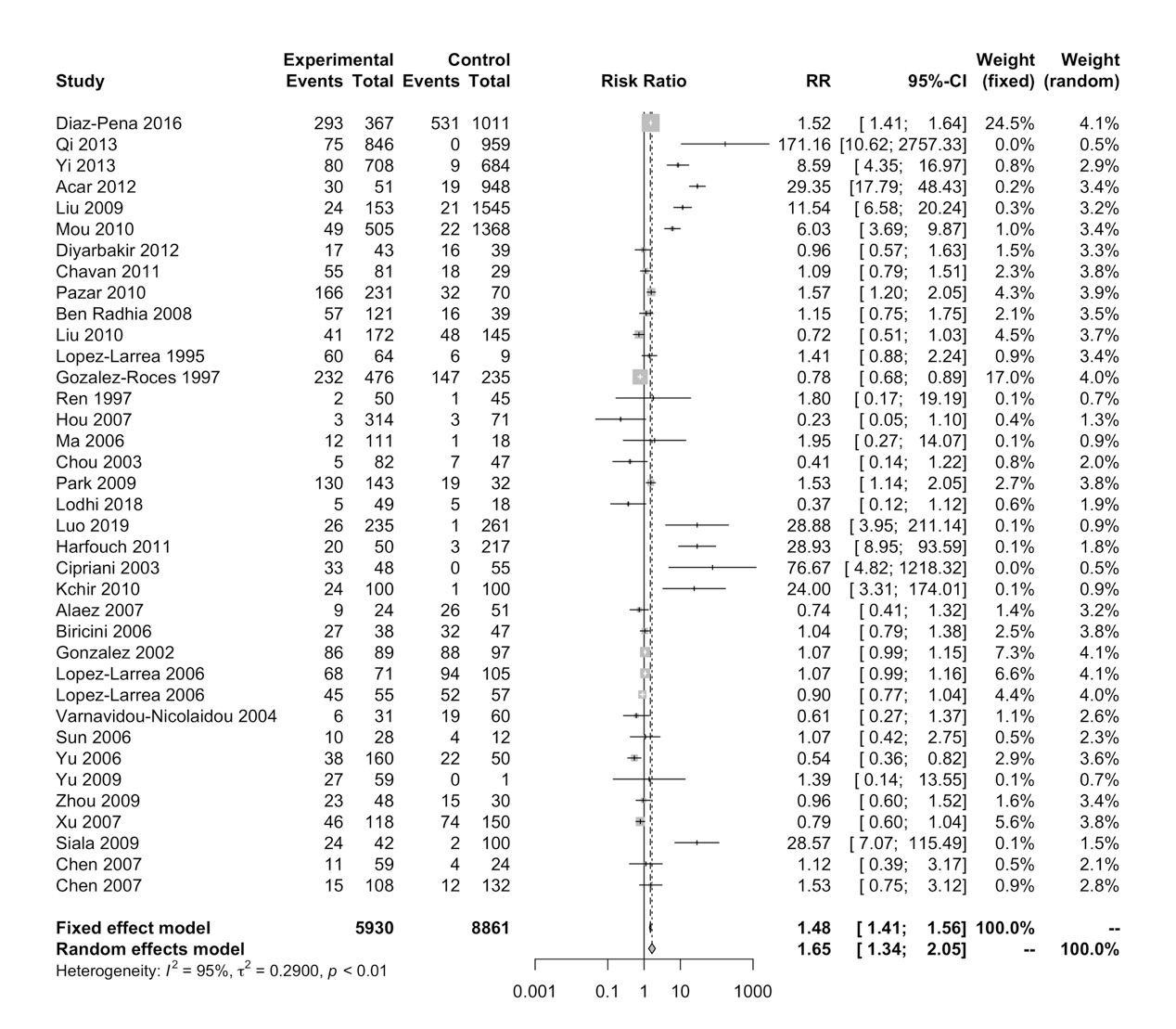


**Figure: Risk of AS in carriers of HLA-B27:05 compared with non-carriers**


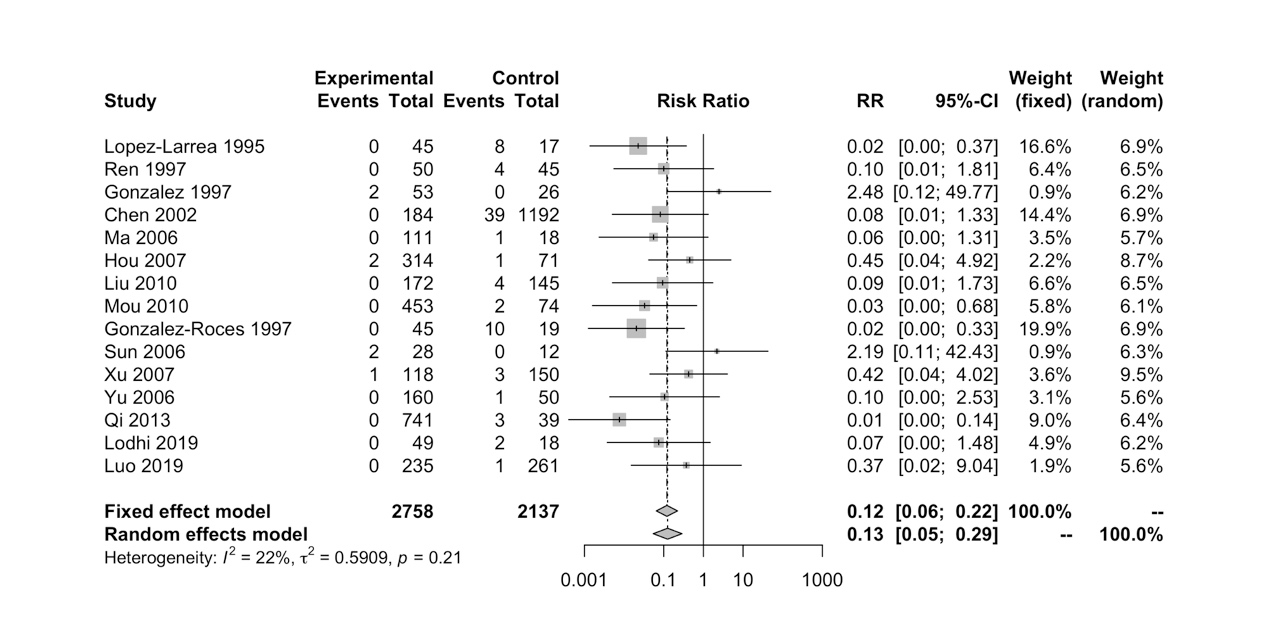


**Figure: Risk of AS in carriers of HLA-B27:06 compared with non-carriers**


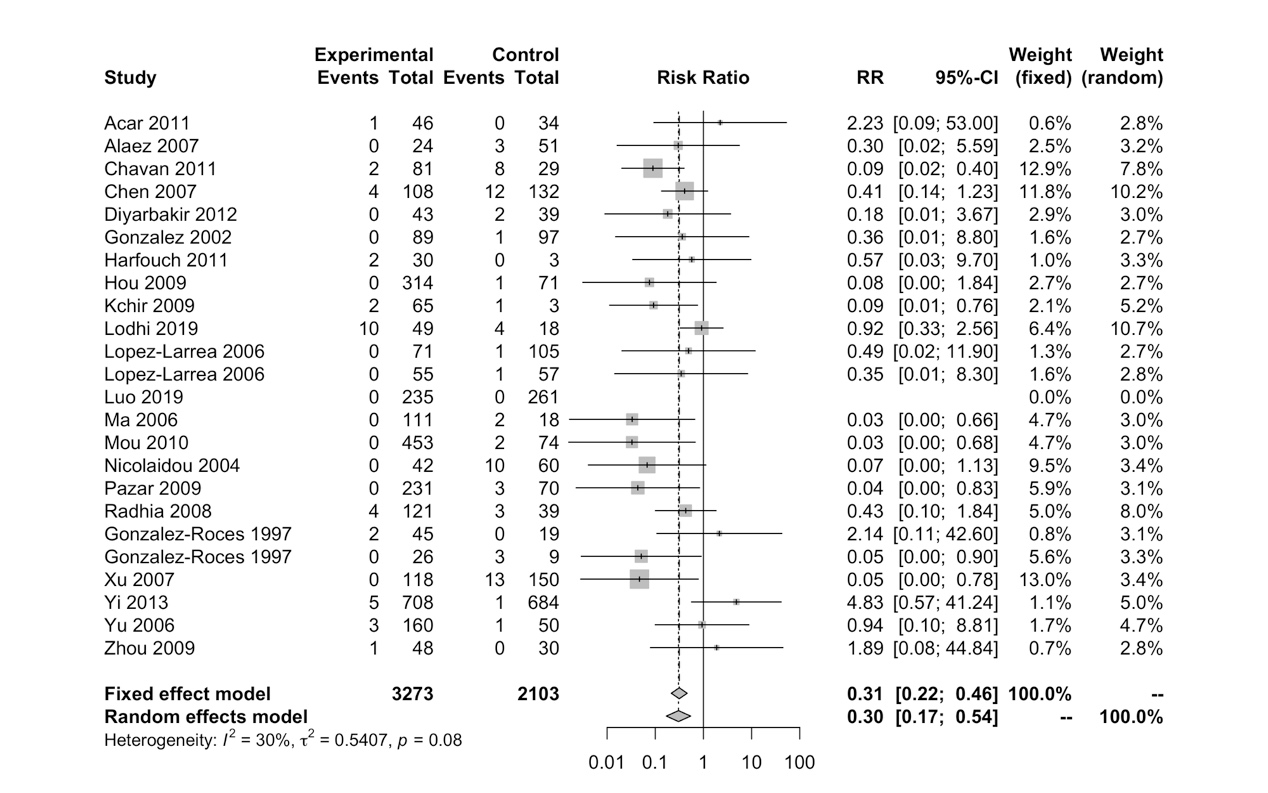


**Figure: Risk of AS in carriers of HLA-B27:07 compared with non-carriers**


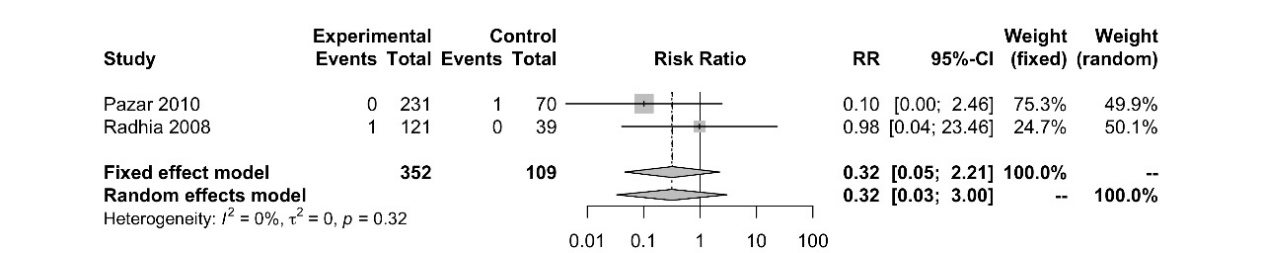


**Figure: Risk of AS in carriers of HLA-B27:09 compared with non-carriers**


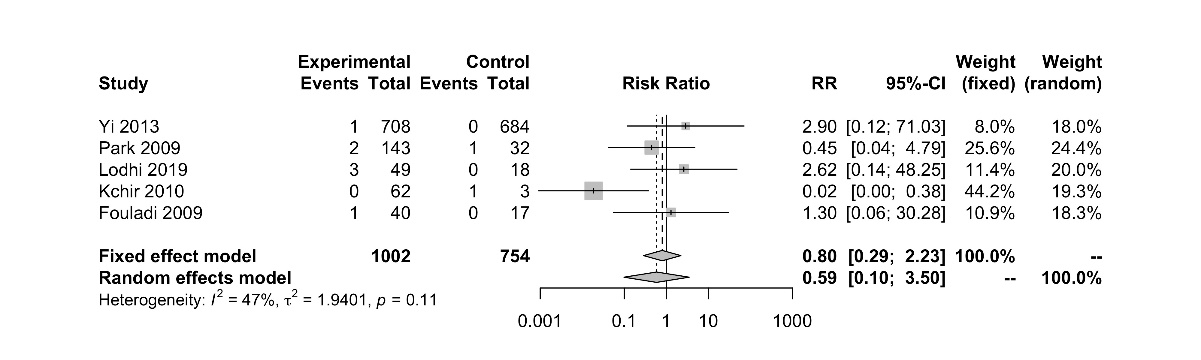


**Figure: Risk of AS in carriers of HLA-B27:10 compared with non-carriers**


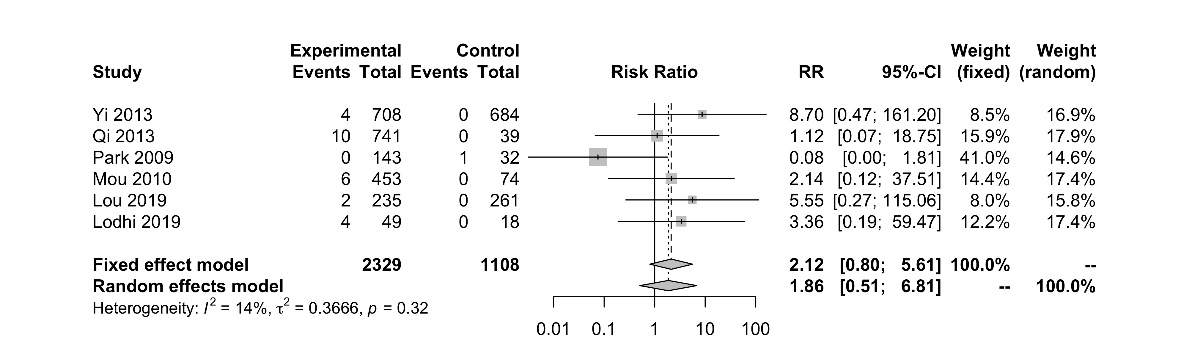


**Figure: Risk of AS in carriers of HLA-B27:15 compared with non-carriers**

**Question 3**: **Genes**

We found 34 cross-sectional studies addressing this question.

The evidence shows:

1.Genome-wide association studies have identified dozens of genetic variants associated with a risk for axSpA, including ERAP1, ERAP2, IL-23R, RUNX3, TNFRSF1A.[86]

2.MHC genes confer more significant genetic risks (20.44%) than non-MHC genes (7.38%).[87] According to our meta-analysis, HLA-DRB1, especially the allele HLA-DRB*12, as well as HLA-B60, was associated the an increased risk of axSpA.

3.It has been reported that polygenic risk scores could capture a more significant proportion of genetic risks of ankylosing spondylitis than HLA-B27 testing alone, with an AUC of 0.924, which is better than HLA-B27 (AUC=0.869), MRI (AUC=0.885) or CRP (AUC=0.700).[88] It is also a useful approach in the diagnosis of acute anterior uveitis (AAU).[89]

The quality of evidence is LOW.

**Table: Evidence profile**

| Certainty assessment | | | | | | | Summary of findings | |
| --- | --- | --- | --- | --- | --- | --- | --- | --- |
| No of participants  (studies)  Follow-up | Risk of bias | Inconsistency | Indirectness | Imprecision | Publication bias | Overall certainty of evidence | Pooled Result (95%CI) | Brief Summary |
| Question 3: | | | | | | | | |
| 34 cross-sectional studies | Not serious | Serious | Not serious | Not serious | Serious | ⨁⨁◯◯  LOW | \ | MHC genes confer more significant genetic risks than non-MHC genes. Polygenic risk scores could capture a more significant proportion of genetic risks of ankylosing spondylitis than HLA-B27 testing alone. |

**Table: Studies addressing the polygenic risk of AS**

| Study | Year | Design | Population | Result |
| --- | --- | --- | --- | --- |
| Cortes[90] | 2013 | cross-sectional | EU:9069AS,1550C  East Asia:13578AS,1567C | In total, 24.4% of the heritability of ankylosing spondylitis is now explained: 4.3% from loci  other than HLA-B and 20.1% due to HLA-B*27 itself. |
| Ellinghaus[1] | 2016 | cross-sectional | 8726AS, 34213HC | the cumulative variance increases to 27.82%, 10.88%, 12.20%, 5.48% and 7.66% (dashed horizontal lines) for ankylosing spondylitis, Crohn's disease, psoriasis, PSC and ulcerative colitis |
| Reveille[87] | 2010 | cross-sectional | 2053AS,5140HC | Both non-MHC genes previously associated with ankylosing spondylitis, ERAP1 and IL23R, were significantly associated in this data set. The most strongly associated SNPs were rs30187 (P = 2.6 × 10−11) and rs11209026 (P = 9.1 × 10−14), confirming the strong association observed for these SNPs in the initial discovery set. |
| Zhang[91] | 2014 | cross-sectional | 602AS,619HC | The study confirmed a weak association between ERAP1 (rs27434) and AS. For HLA-B*27:02 and HLA-B*27:04 positive AS patients, rs27434 and rs27582 were associated with AS. In contrast, for HLA-B27-negative and HLA-B*27:05-positive AS patients |
| Daryabor[92] | 2014 | cross-sectional | 294AS,352HC | only rs1004819 has a significant association with AS, and that the remaining four SNP alleles are not associated with AS. |
| Burton[86] | 2007 | cross-sectional | 2000AS,3000HC | IL23R may be a common susceptibility factor for the major 'seronegative' diseases. |
| Wiśniewski[93] | 2019 | cross-sectional | 180AS,506HC | the haplotype H4, containing ERAP1 SNPs associated with high enzymatic activity, together with the presence of ERAP2 expression, significantly increased the risk of AS (OR = 1.97, 95% CI = 1.21-3.21, p(corr) = 0.048). |
| Robinson[94] | 2015 | cross-sectional | 5040AS,21133HC | The associations with SNPs in ERAP1, IL23R, the intergenic regions chromosomes 2p15 and 21q22, GPR35 and IL6R were confirmed at genome-wide significance level (P<5×10−8). |
| Soomro[95] | 2022 | cross-sectional | 4072PSA,9965HC | the SNP-based heritability (h2 SNP) of PsA in the PsA-BSTOP GWAS data set of 3,609 patients and 9,192 controls. The estimated heritability of PsA in the full data set was h2 SNP = 0.63 (SD 0.04), while in analyses using non-MHC SNPs, the estimated heritability was h2 SNP= 0.61 (SD 0.04) |
| Bettencourt[96] | 2013 | cross-sectional | 200AS,559HC | The marker rs30187 revealed the strongest association level (OR = 1.5, P = 4.7 × 10−3) (Table 3). The attributable risk for rs30187 and rs22047 was 11.7% and 14.6%. A protective effect was found in three of the ERAP1 SNPs—rs17482078, rs10050860and rs2287987). Fifty-five SNPs were imputed in the ERAP1 region, in a block of ∼89 kb (Fig. 1B). Thirty-two of those SNPs had a significant association with AS. No association was observed in the TNFSF15 region in either the genotyped or in the nine imputed SNPs |
| Lin[97] | 2012 | cross-sectional | 1837AS,4231HC | extensive associations within the MHC region at 6p21, with the strongest association at rs13202464 (odds ratio (OR) = 41.6, P < 5 × 10−324). We also replicated the previously reported association with rs10865331 at 2p15 (OR = 0.79, P = 1.98 × 10−8) and found evidence supporting the reported associations of ERAP1 and IL12B . |
| Zvyagin[98] | 2010 | cross-sectional | 83AS，107HC | three allelic variants of erap1 are associated with an increased risk of AS (rs2287987 [T] (OR = 2.86), rs10050860 [C] (OR = 2.54), and rs17482078 [C] (OR = 1.91).rs30187 and rs27044 polymorphisms with AS in the Russian population lack of a significant association. |
| Zhang[91] | 2014 | cross-sectional | 602AS，619HC | ERAP1 gene polymorphisms are associated with Beijing Han AS. Specifically, only in HLA-B*27:02 and B*27:04 positive groups ERAP1 gene polymorphisms were associated with Beijing Han AS. the ERAP1 gene polymorphism is not associated with AS in B27-negative patients. |
| Zhang[99] | 2015 | cross-sectional | 679AS，628HC | the rs3212227 CC/AC genotype was more frequent among the Taiwanese patients. there was no significant differences of genotypic or allelic frequencies in mainland Han Chinese in this study. |
| Rostami[100] | 2020 | cross-sectional | 164AS, 49032HC | Prediction in a population-based setting based on all currently known AS susceptibility SNP was better than HLA-B27 carrier state alone, although the improvement was small and of uncertain clinical value. |
| Jung[101] | 2016 | cross-sectional | 861AS, 1043HC | Through logistic regression, we built the AS-GRS model consisting of 5 genetic components: HLA-B27, 3 CNV (1q32.2, 13q13.1, and 16p13.3), and 1 SNP (rs10865331). AS-GRS model showed higher specificity and accuracy than the HLA-B27–only model when the sensitivity was set to over 94%. |
| Li[88] | 2021 | cross-sectional | 15585AS, 20452HC | In people of European descent, PRS had high discriminatory capacity with area under the curve (AUC) in receiver operator characteristic analysis of 0.924. This was significantly better than for HLA-B27 testing alone (AUC=0.869), MRI (AUC=0.885) or C-reactive protein (AUC=0.700). PRS developed and validated in individuals of East Asian descent performed similarly (AUC=0.948). |
| Huang[89] | 2020 | cross-sectional | 2752AS with acute anterior uveitis, 3836 AS without | We identified one locus associated with AAU at genomewide significance: rs9378248 (P = 2.69 × 10-8, odds ratio [OR] = 0.78), lying close to HLA-B. |
| Thomas[102] | 2017 | cross-sectional | 4428axSpA, 9638HC | We first established the predictive accuracy of genetic data comparing 9,638 healthy controls and 4,428 AS cases from the homogenous International Genetics of AS (IGAS) Consortium Immunochip study which showed excellent predictive power (AUC=0.91). Genetic risk scores had lower predictive power (AUC=0.83) comparing ASAS cohort axSpA cases meeting the ASAS imaging criteria with IGAS controls. |


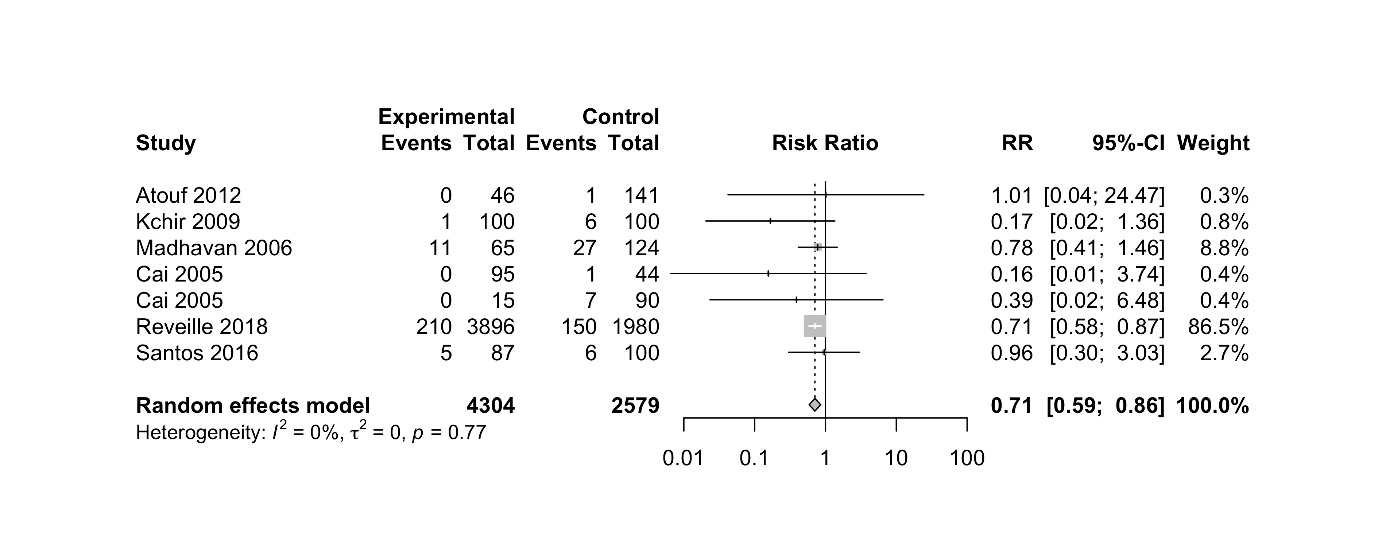
**Figure: Risk of AS in carriers of HLA-B40 compared with non-carriers**

**Figure: Risk of AS in carriers of HLA-B60 compared with non-carriers
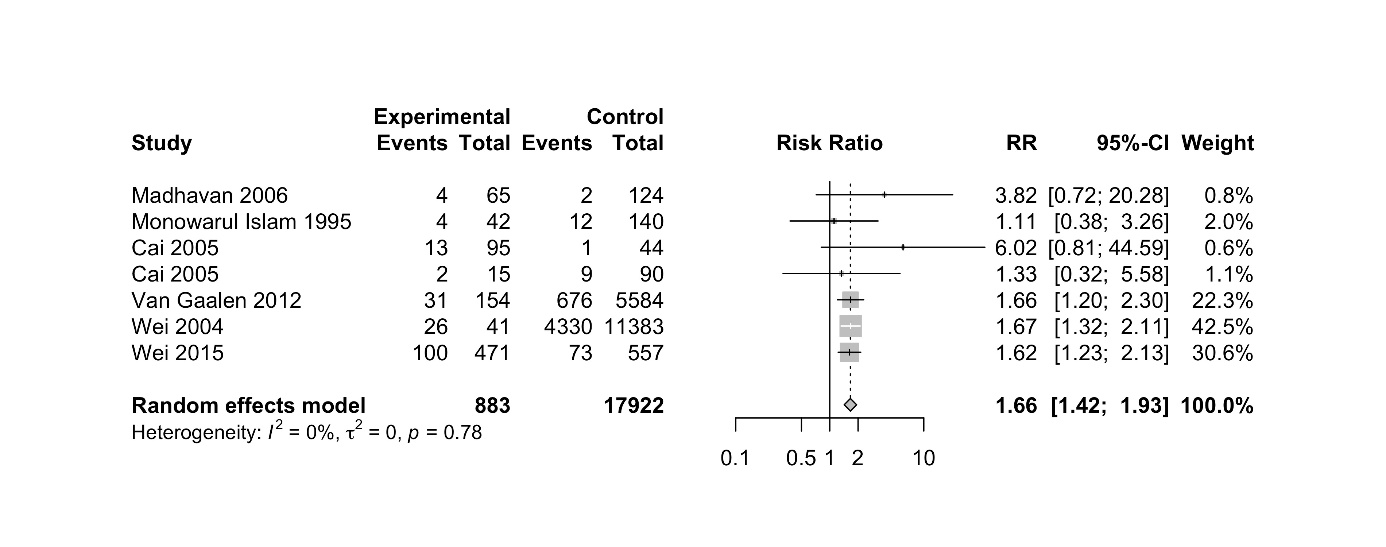
**

**Figure: Risk of AS in carriers of HLA-B61 compared with non-carriers
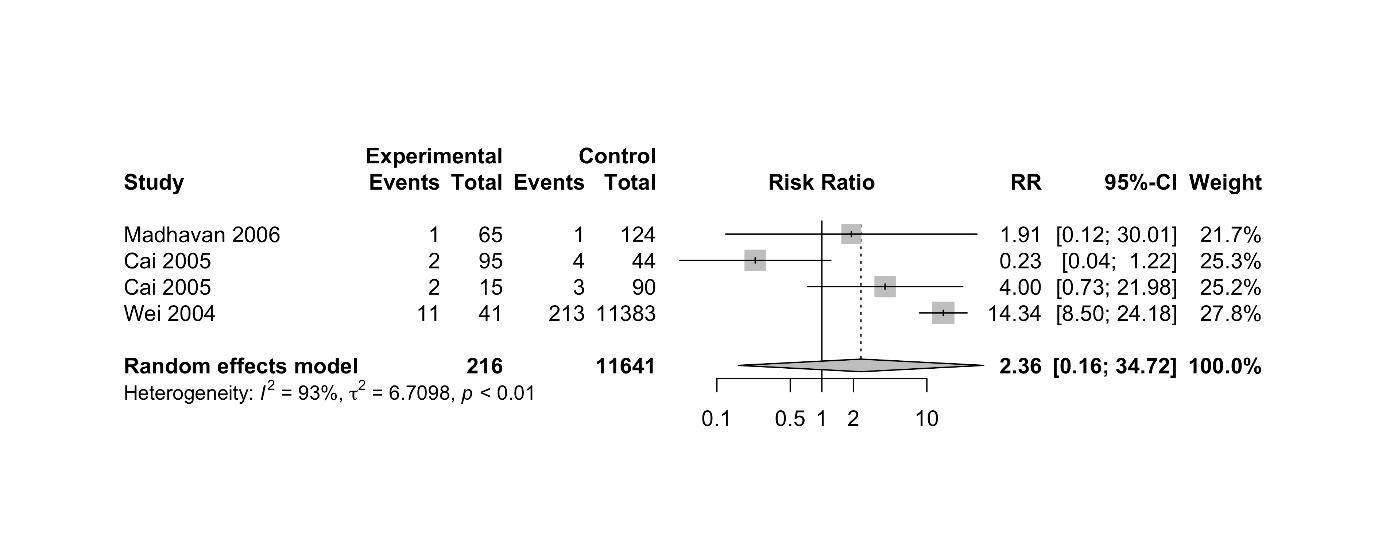
**

**Figure: Risk of AS in carriers of HLA-DRB1 compared with non-carriers
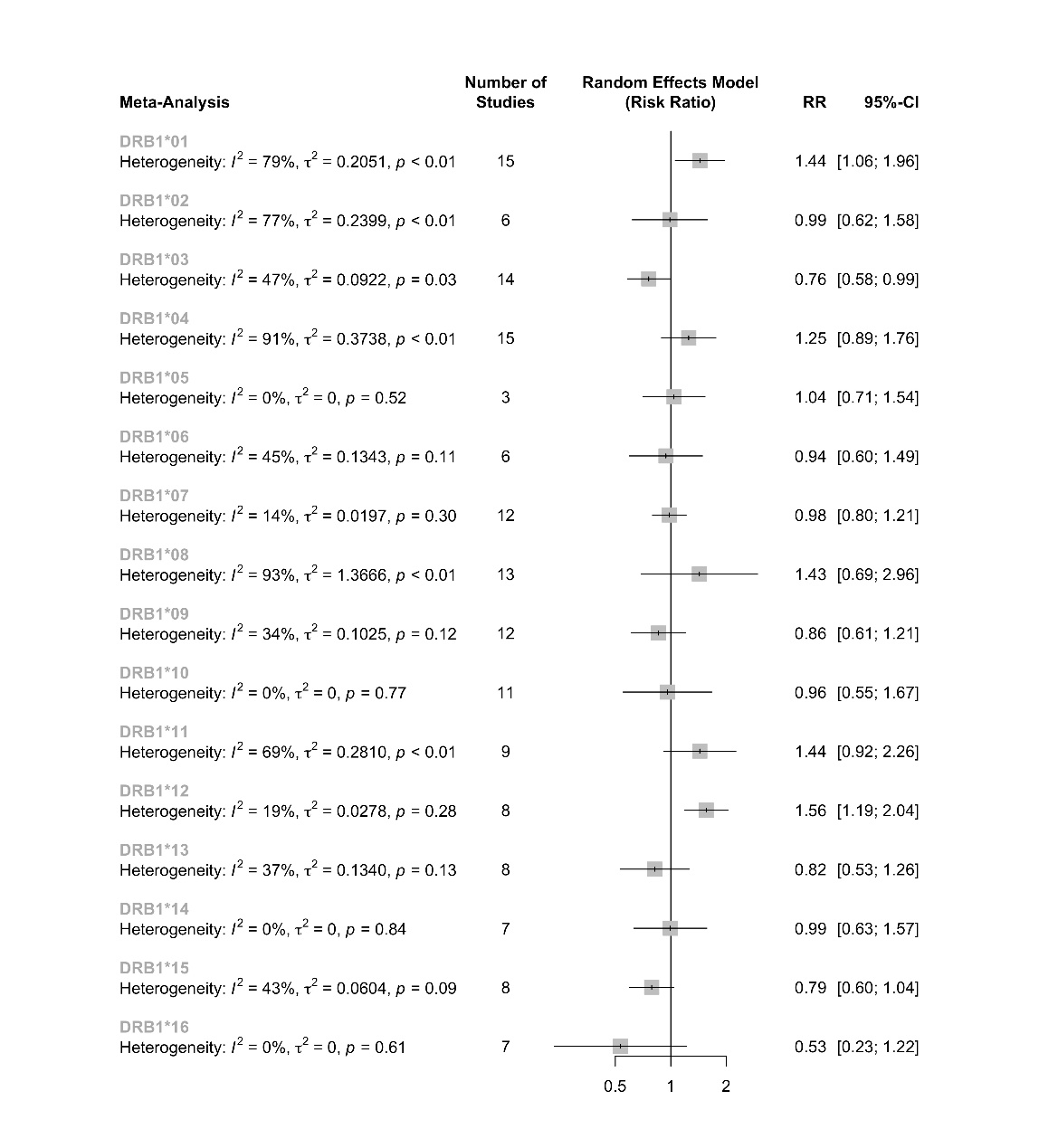
**

**Figure: Risk of AS in carriers of HLA-DRB1*01 compared with non-carriers
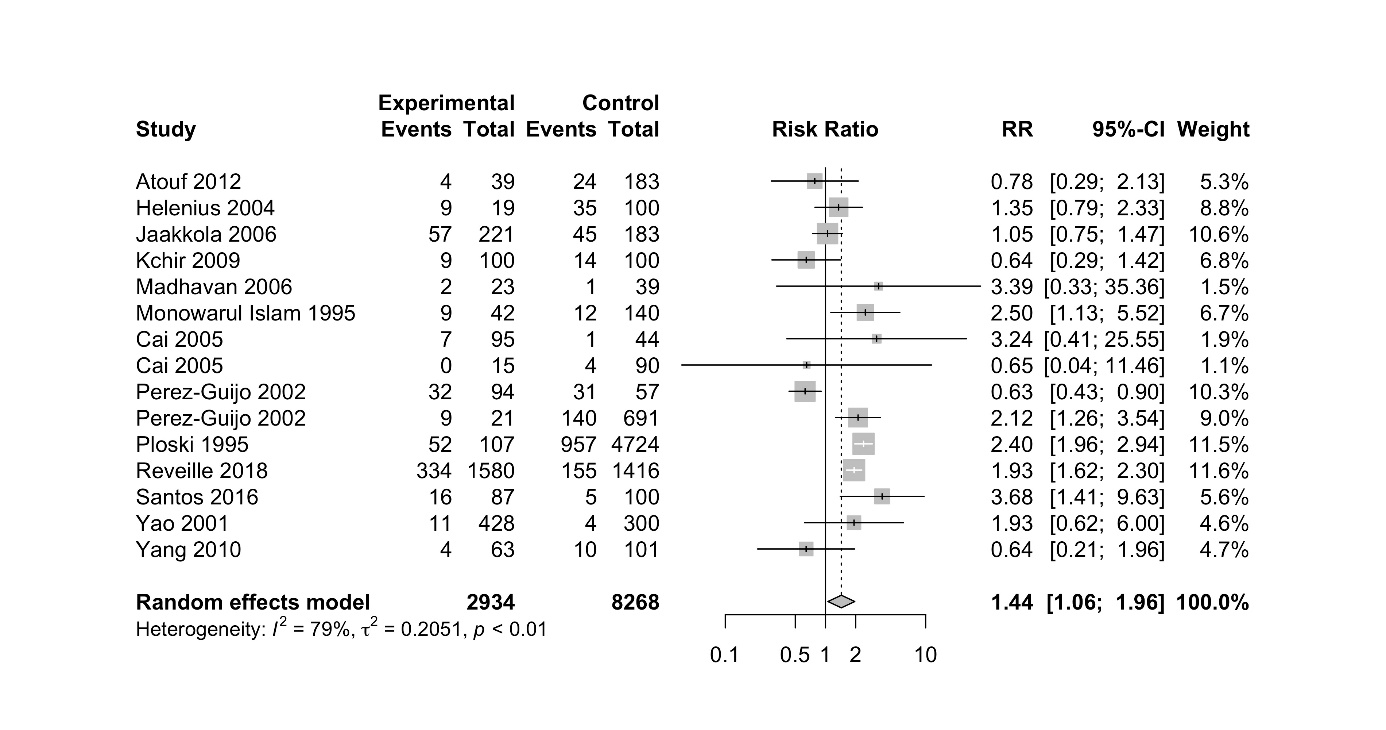
**

**Figure: Risk of AS in carriers of HLA-DRB1*02 compared with non-carriers
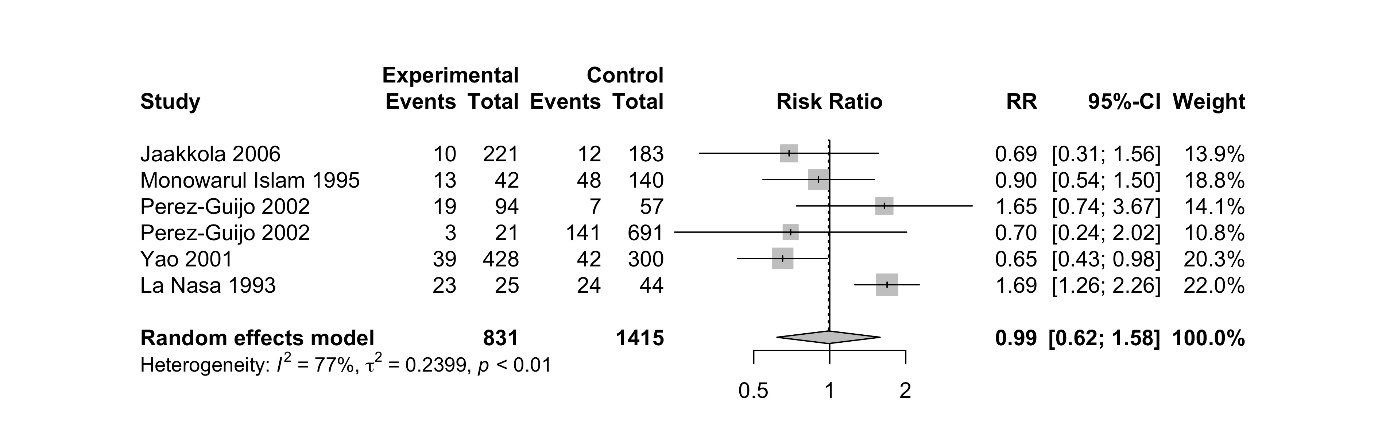
**

**Figure: Risk of AS in carriers of HLA-DRB1*03 compared with non-carriers
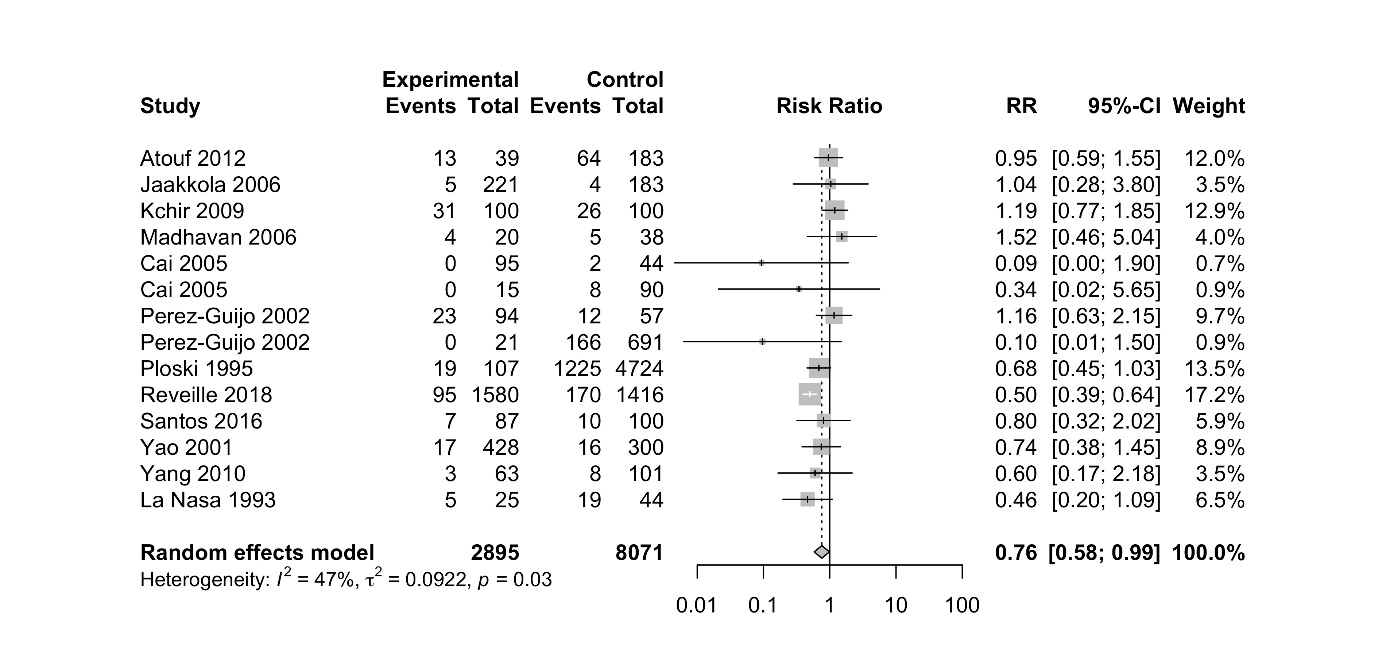
**

**Figure: Risk of AS in carriers of HLA-DRB1*04 compared with non-carriers
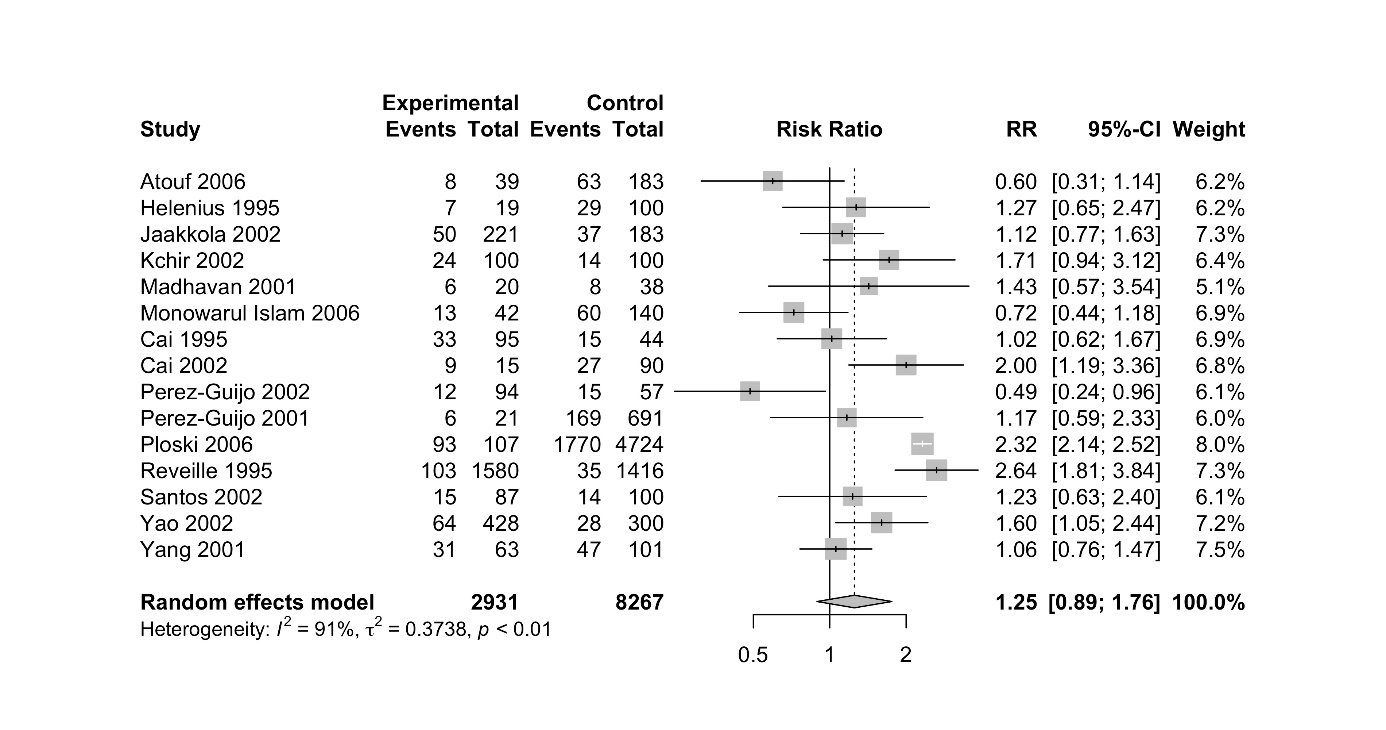
**

**Figure: Risk of AS in carriers of HLA-DRB1*05 compared with non-carriers
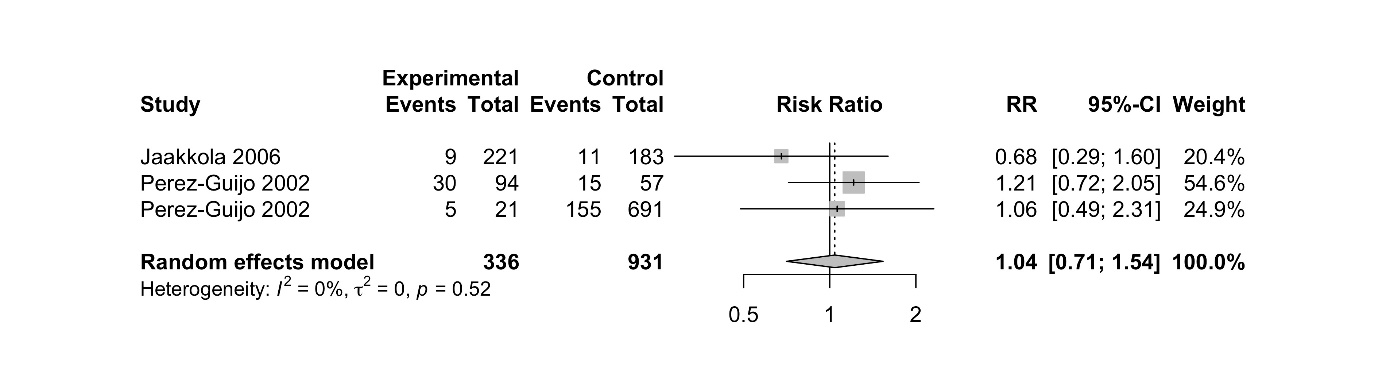
**

**Figure: Risk of AS in carriers of HLA-DRB1*06 compared with non-carriers
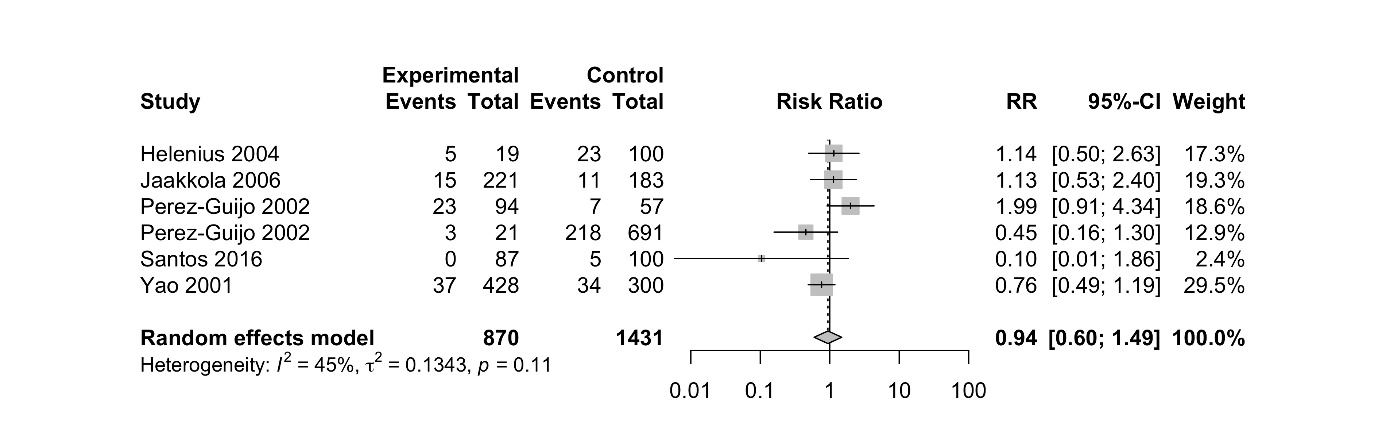
**

**Figure: Risk of AS in carriers of HLA-DRB1*07 compared with non-carriers
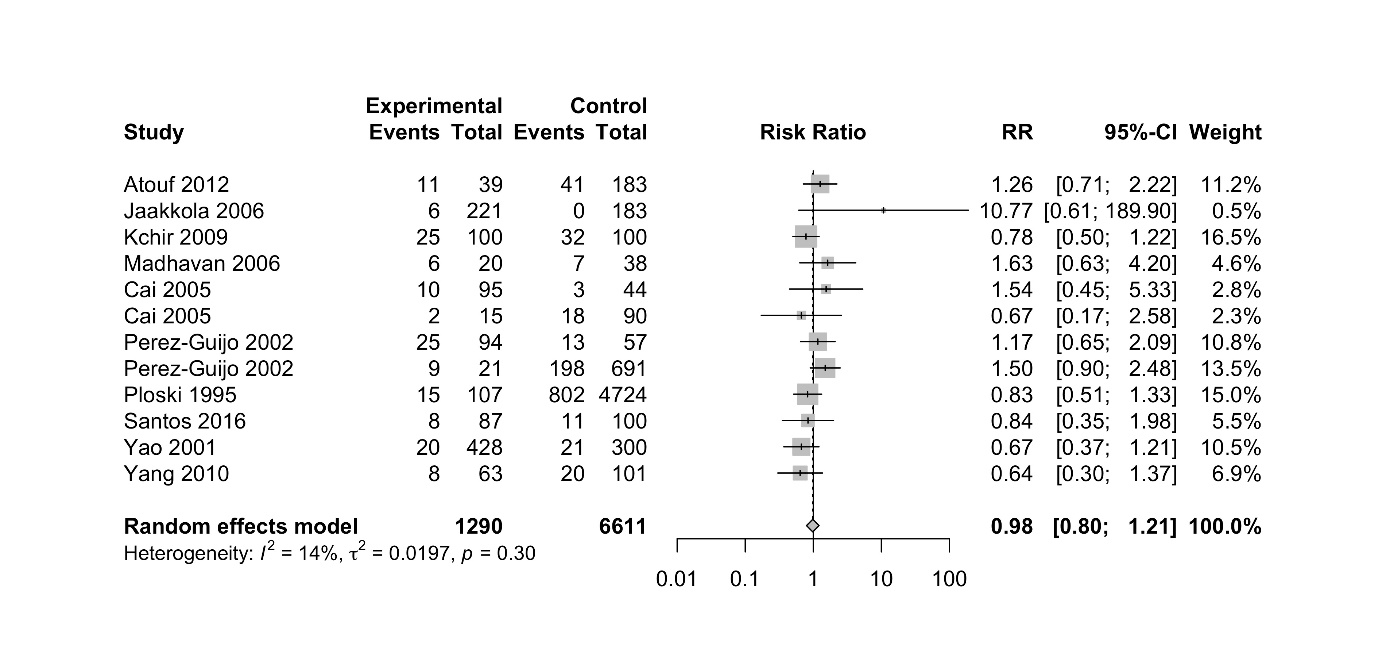
**

**
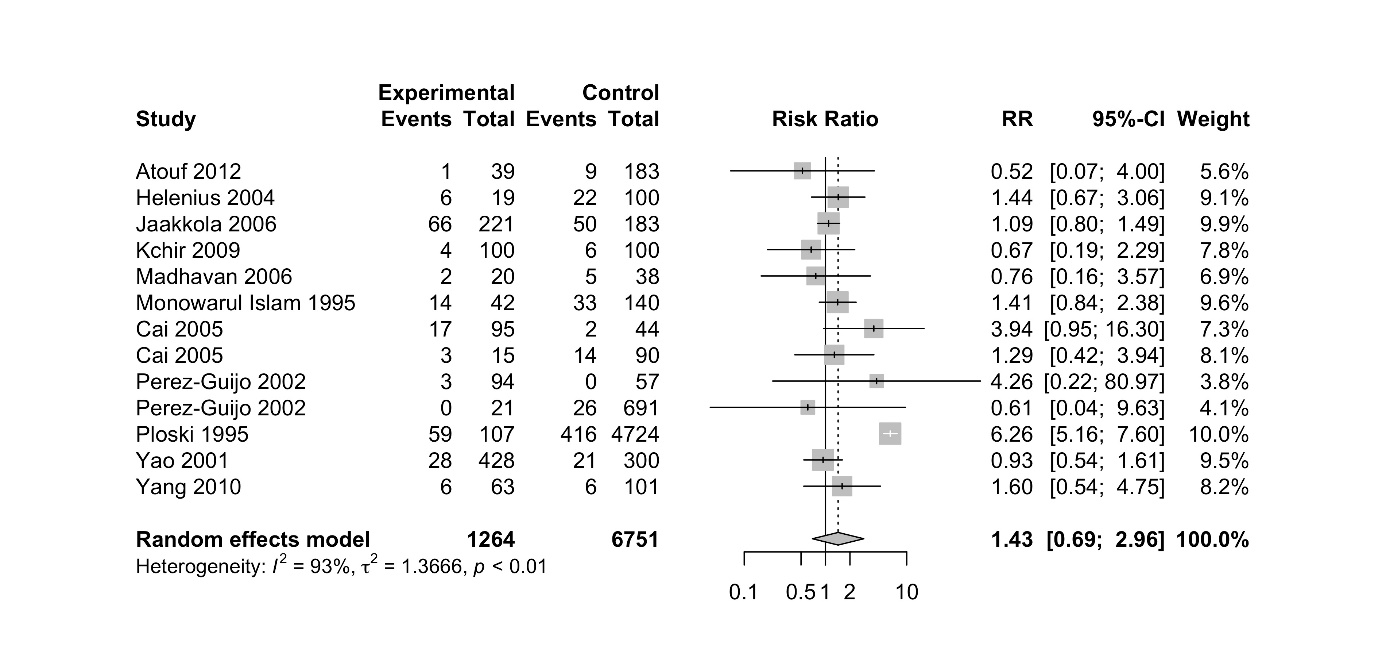
Figure: Risk of AS in carriers of HLA-DRB1*08 compared with non-carriers**

**Figure: Risk of AS in carriers of HLA-DRB1*09 compared with non-carriers
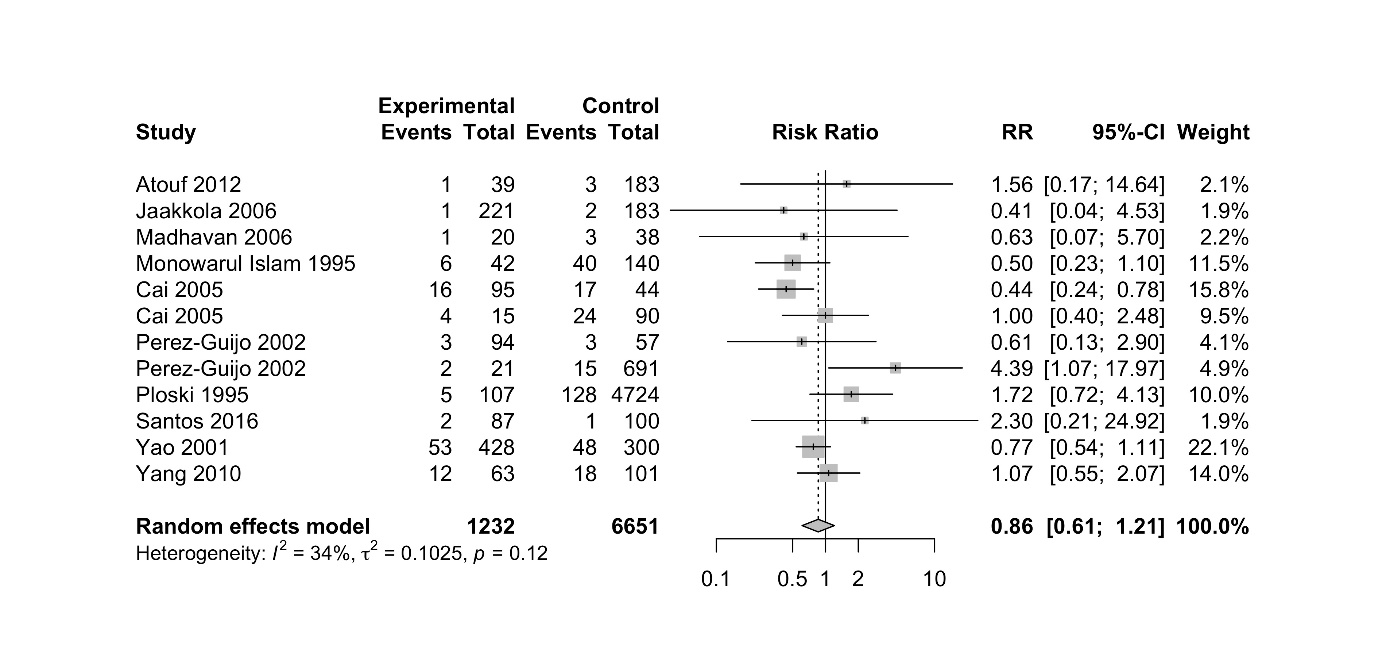
**

**Figure: Risk of AS in carriers of HLA-DRB1*10 compared with non-carriers
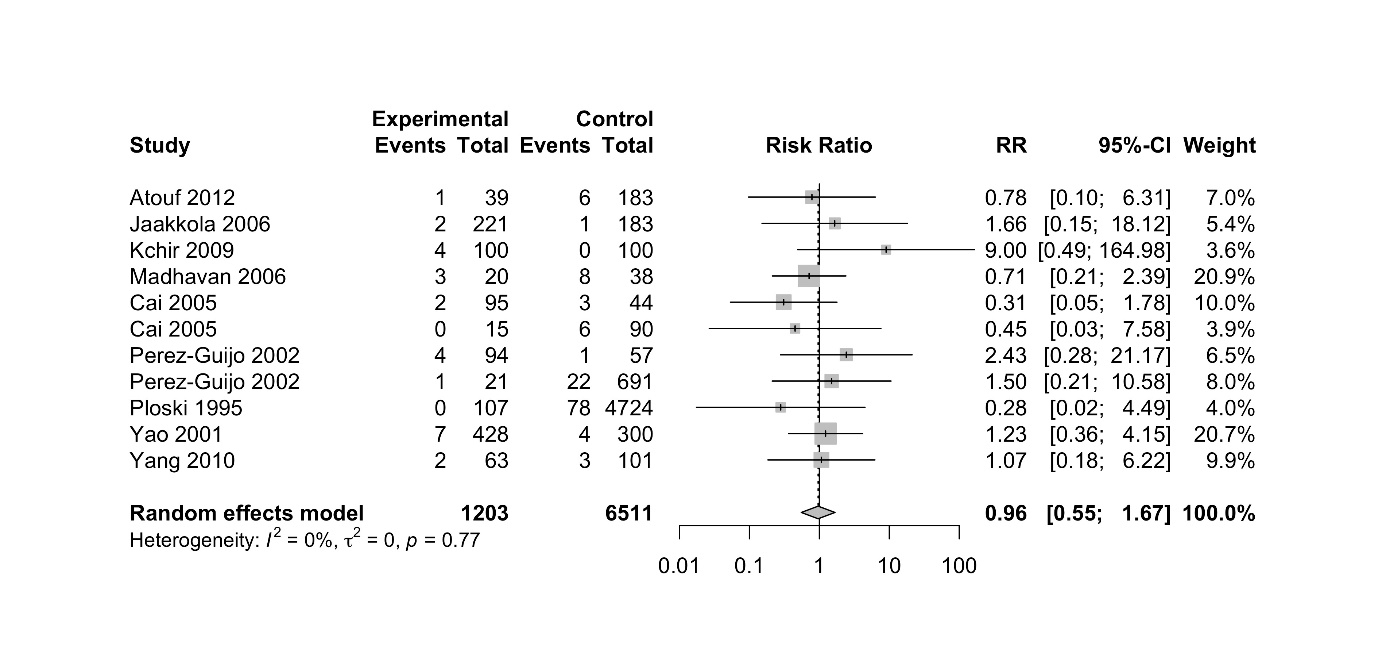
**

**Figure: Risk of AS in carriers of HLA-DRB1*11 compared with non-carriers
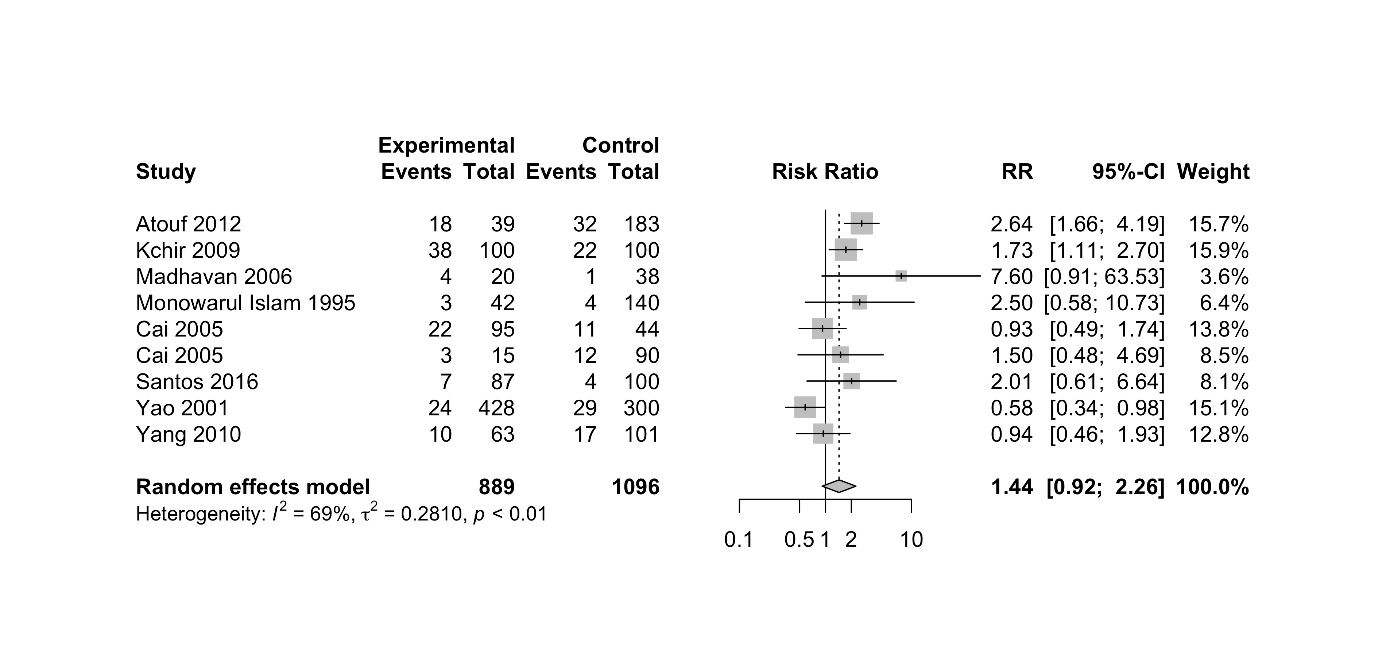
**

**Figure: Risk of AS in carriers of HLA-DRB1*12 compared with non-carriers
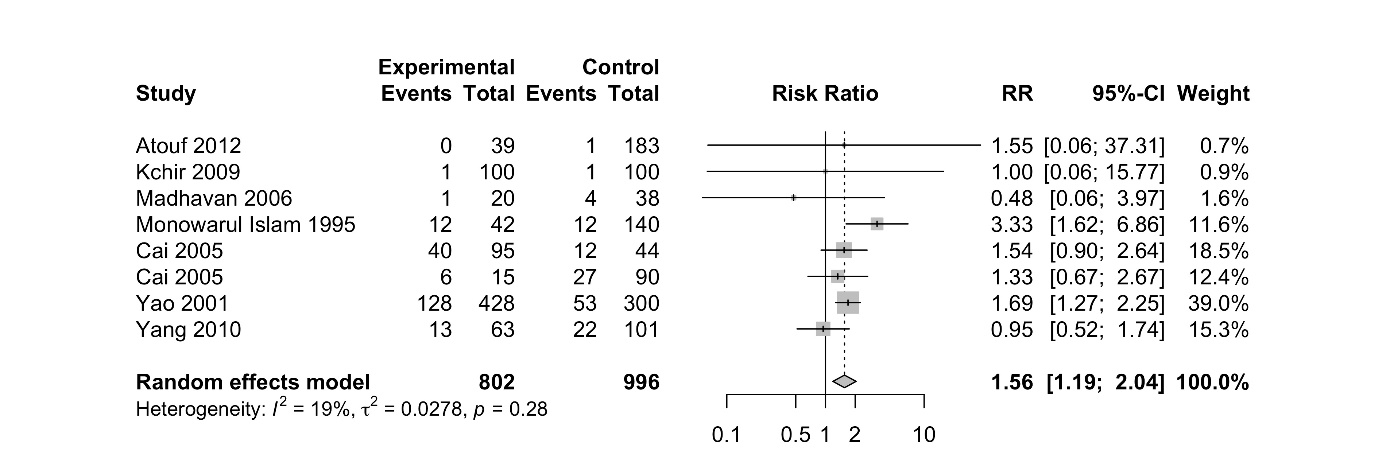
**

**Figure: Risk of AS in carriers of HLA-DRB1*13 compared with non-carriers
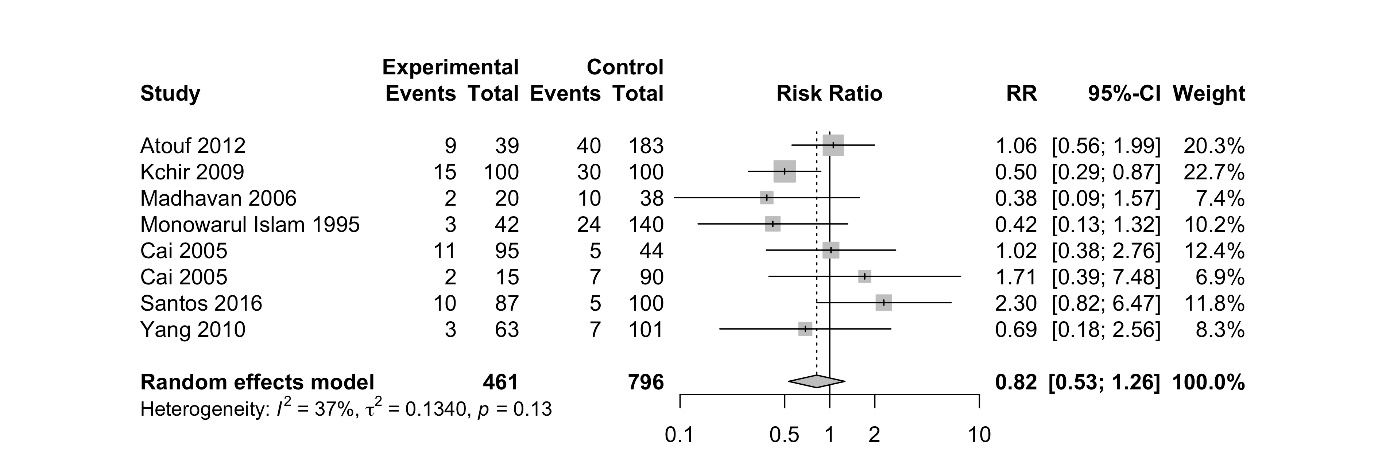
**

**Figure: Risk of AS in carriers of HLA-DRB1*14 compared with non-carriers
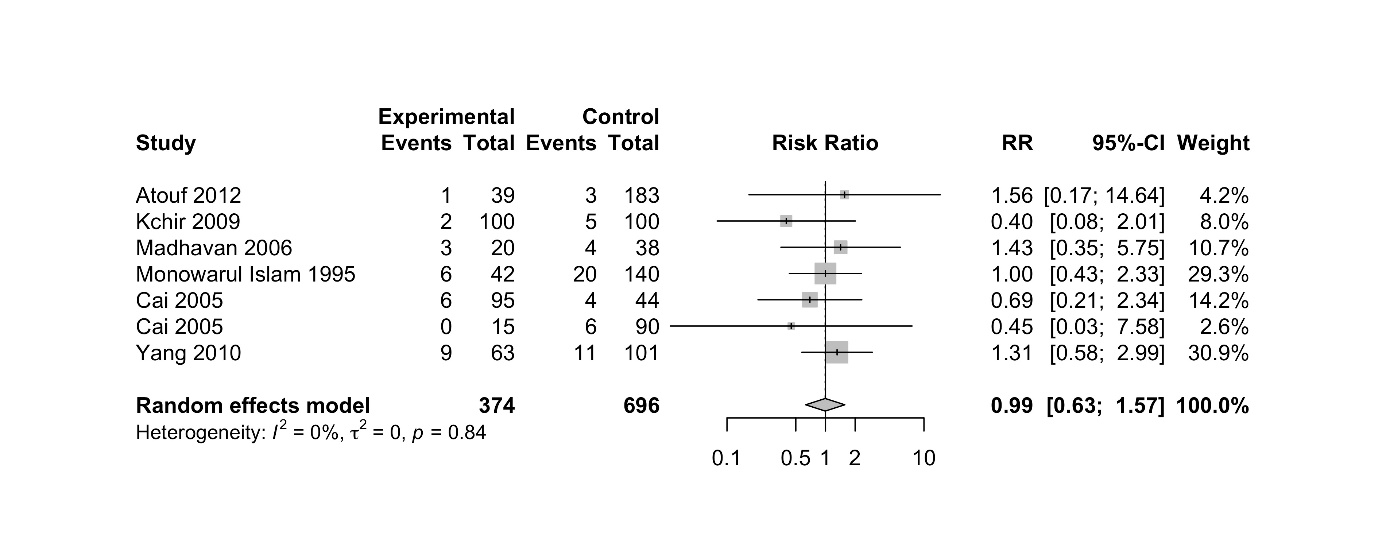
**

**Figure: Risk of AS in carriers of HLA-DRB1*15 compared with non-carriers
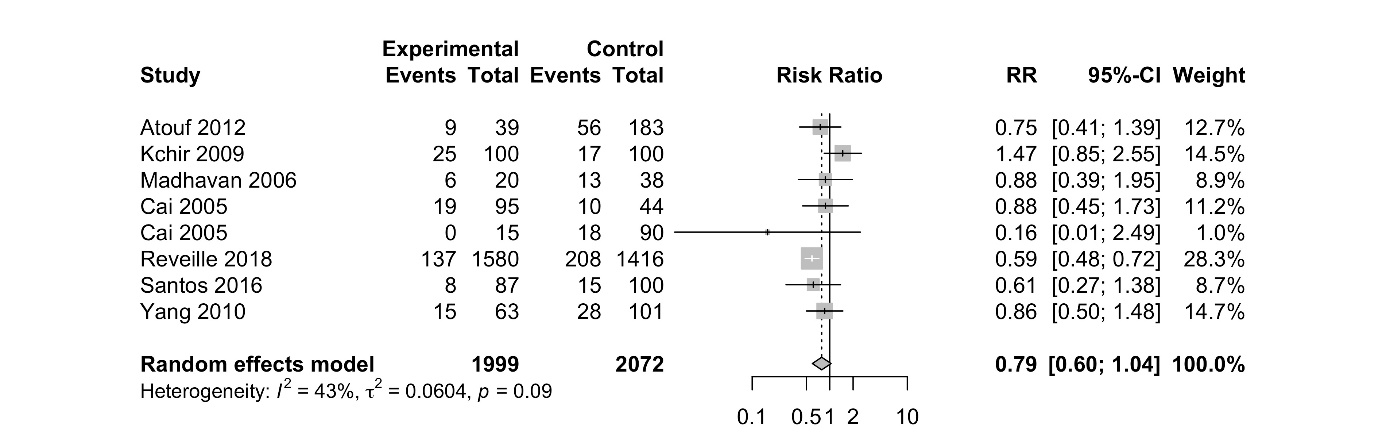
**

**Figure: Risk of AS in carriers of HLA-DRB1*16 compared with non-carriers
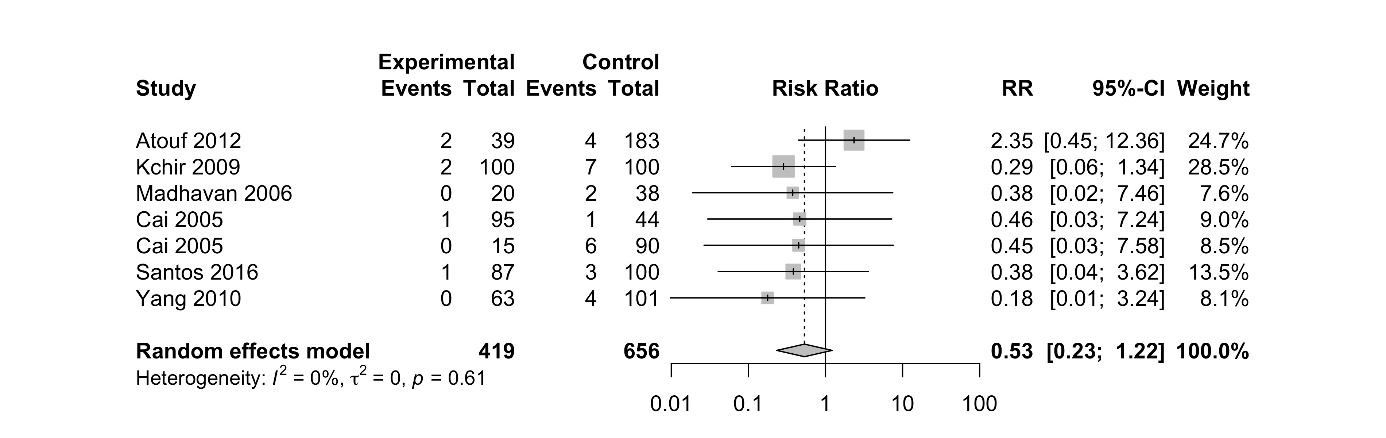
**

**Question 4**: **Antibodies**

We found 34 cross-sectional studies and 2 cohort studies addressing this question.

The evidence shows:

1.Antibodies associated with axSpA include autoantibodies and anti-pathogen antibodies. Autoantibodies include anti-CD74, anti-sclerostin and anti-noggin antibodies. Antibodies against microbial targets such as Klebsiella pneumonia, Yersinia could also be present in axSpA patients.

2.Studies investigating the diagnostic utility of anti-CD74 antibodies were highly inconsistent. Despite some reports exhibiting high specificity of anti-CD74 antibodies (95.3%) [103], SPACE cohort showed that the positive predictive value was only 58.5%, while the negative predictive value was only 59.1%.[104]

3.Sclerostin could interfere with Wnt pathway, mitigating bone formation, while noggin could target BMP signaling also leading to less bone formation. Antibodies targeting sclerostin and noggin could be associated with excessive bone formation [105], but high quality studies are warranted.

4.Antibodies targeting microbial target failed to prove diagnostic utility or correlation with disease activity in axial spondyloarthritis.

The quality of evidence is LOW.

**Table: Evidence profile**

| Certainty assessment | | | | | | | Summary of findings | |
| --- | --- | --- | --- | --- | --- | --- | --- | --- |
| No of participants  (studies)  Follow-up | Risk of bias | Inconsistency | Indirectness | Imprecision | Publication bias | Overall certainty of evidence | Pooled Result (95%CI) | Brief Summary |
| Question 4: | | | | | | | | |
| 36 studies (34 cross-sectional studies and 2 cohort studies) | Serious | Serious | Not serious | Not serious | Not serious | ⨁⨁◯◯  LOW | \ | Autoantibodies include anti-CD74, anti-sclerostin and anti-noggin antibodies. Studies investigating the diagnostic utility of anti-CD74 antibodies were highly inconsistent. Antibodies targeting microbial target failed to prove diagnostic utility or correlation with disease activity in axial spondyloarthritis. |

**Table: Studies addressing anti-CD74 antibodies as biomarkers for diagnosis**

| Study | Year | Design | Population | Result |
| --- | --- | --- | --- | --- |
| Baraliakos[106] | 2014 | cross-sectional | axSpA | AxSpA patients showed higher levels of anti-CLIP-ABs vs non-SpA.The The sensitivity of anti-CLIP-ABs and HLA-B27 for diagnosing axSpA were 85.1% and 77.8%，which means that the likelihood ratio for confirming axSpA by using anti-CLIP was even higher than HLA-B27. |
| Baerlecken[107] | 2014 | cross-sectional | axSpA | IgG antibodies against the CLIP domain of the CD74 protein were present in 145/216 (67%) of the sera obtained from patients with SpA.The presence of IgG antibodies against CLIP was 56/58 (97%) in SpA with a duration of inflammatory back pain of less than 1 year and declined after the first year. |
| Abdelaziz[108] | 2021 | cross-sectional | SpA | Anti-CD74 IgG antibodies had 80% sensitivity and 87% specificity in diagnosis of axial spondyloarthritis.The frequency of positive CD74 autoantibodies was not significantly different between those with early and late axSpA (P=0.629). |
| Do[109] | 2021 | cross-sectional | r-axSpA | The plasma level of IgA antiCD74 was significantly higher in the patients compared with the controls [12.9 (7.9–17.9) U/ml vs 10.9 (7.2–14.6) U/ml; P=0.003]., The plasma levels of IgA anti-CD74 showed significant associations with ESR and mSASSS. |
| de Winter[104] | 2018 | cross-sectional | axSpA | Anti-CD74 IgG antibodies were present in 79.7% of patients with AS vs. 43.9% of controls (p < 0.001)，but anti-CD74 IgA antibodies were present in 54.7% of the patients with axSpA and 37.0% of the patients with CBP (p < 0.001). The mean disease duration in patients with AS with or without anti-CD74 IgA antibodies did not differ (24.12 vs. 24.35 months, respectively, p = 0.931),which means that anti-CD74 IgA have no diagnostic value in early axial spondyloarthritis. |
| Riechers[110] | 2019 | cross-sectional | axSpA | The sensitivity of IgA anti- CD74 and IgG antiCD74 for identifying the 100 axSpA patients was 47% and 17%, respectively. A combination of IgA anti- CD74 and HLA–B27 results in an increasing posttest probability of 80.2% in IBP patients. |
| Hu[111] | 2020 | cross-sectional | axSpA | AxSpA patients displayed significantly higher meanconcentrationofanti-CD74 IgAantibody (25.7±39.1U/mL) in comparison to HC group (9.0±3.6U/mL; P=.000).The positivity of IgA and IgG anti-CD70 was only 23.4%,indicating that there was little clinical value of anti-CD74 autoantibodies in Chinese axSpA population. |
| Ziade[112] | 2019 | cross-sectional | axSpA | IgG4 anti-CD 74 were positive in 88% of HLA-B27 negative axSpA patients, and correlated with BASDAI.IgG4 anti-CD74 antibodies combined with HLA-B27 showed higher diagnostic value than HLA-B27 or IgG4 anti-CD74 alone for early axSpA. |
| Çolak[113] | 2021 | cross-sectional | AS | The mean levels of anti-CD74 antibodies in the AS, IBD, and control groups were 6.99±3.24 ng/mL, 6.25±3.34 ng/mL, and 7.83±4.72 ng/mL, respectively. There was no relationship between CRP,BASFI, BASDAI, BASMI, and ASDAS-CRP scores and levels of CD74 .This study could not find an association between anti-CD74 levels and SpA in Turkish patients. |
| Liu[114] | 2019 | cross-sectional | SpA | The prevalence of antiCD74 in Chinese SpA patients was 14.1%, and 2.9% in healthy controls,but the prevalence of anti-CD74 was not as high as the results from the Europe cohort.No significant correlation was found between titer of anti-CD74 and these disease activity indexes.This study indicated that anti-CD74 might not be a good biomarker for SpA diagnosis in Asian people. |

**Table: Studies addressing anti-sclerostin and anti-noggin antibodies as biomarkers for diagnosis**

| Study | Year | Design | Population | Result |
| --- | --- | --- | --- | --- |
| Tsui[105] | 2014 | cross-sectional | AS | Compared to MBP, AS patients have significantly higher levels of NOG-N54 (A) or SOST-S146 IgG.There was a modest correlation between ESR, but not CRP, and the levels of NOG-N54 and SOST-S146 IgG. |

**Table: Studies addressing ANA as a biomarker for diagnosis**

| Study | Year | Design | Population | Result |
| --- | --- | --- | --- | --- |
| Rosenberg[115] | 1979 | cross-sectional | AS | ANA were detected in the sera of 5 of 88 patients with ankylosing spondylitis (AS) and in 7 of 52 cases of psoriatic arthritis (PsA).The presence of antinuclear antibodies was not associated with clinical features or drug therapy in either AS or PsA. |

**Table: Studies addressing ANCA as a biomarker for diagnosis**

| Study | Year | Design | Population | Result |
| --- | --- | --- | --- | --- |
| de Vries[116] | 2010 | cross-sectional | AS | In the overlap group with concurrent AS and IBD, pANCA was more frequent in patients with UC as well (p = 0.024).pANCA was statistically significantly more frequent in AS with concurrent UC than in AS alone. |
| Matzkies[117] | 2012 | cross-sectional | AS | The median ANCA value was higher in AS patients than in the controls (P = 0.003). |
| Wallis[118] | 2013 | cross-sectional | AS | ANCA levels were similar in AS-IBD and AS and were significantly elevated in both these groups when compared to MBP. |

**Table: Studies addressing antibodies against microbial targets as biomarkers for diagnosis**

| Study | Year | Design | Population | Result |
| --- | --- | --- | --- | --- |
| Stone[119] | 2004 | cross-sectional | AS | There was no difference in mean stimulation indices or antibody responses between affected and unaffected family members for each of the candidate organisms including Klebsiella pneumoniae, Salmonella typhimurium, Yersinia enterocolitica and Chlamydia trachomatis. |
| Stebbings[120] | 2002 | cross-sectional | AS | Serum IgA antibodies reactive with Bacteroides vulgatus and Serum IgM antibodies reactive with Klebsiella pneumoniae were lower in concentration in AS patients compared with controls (P=0.0345;P=0.0463). |
| Tiwana[121] | 1997 | cross-sectional | active AS | A significant elevation of Ig immunoreactivity to Klebsiella pneumoniae was detected in the active AS than HC (1.13±0.04 vs 0.78 ±0.03, P<0.001),but there was no significant elevation of total Ig levels to Escherichia coli. |
| Collado[122] | 1994 | cross-sectional | AS | AS showed higher levels of anti- klebsiella IgA antibodies (IgA-Kp) than NIA and RA patients (4.7 ± 1.6 U vs 3.7 ± 1.5 U and 3.1 ± 1.4 U respectively, p = 0.001). A significant correlation between IgA-Kp and levels of CRP was observed in AS. |
| Cooper[123] | 1988 | cross-sectional | AS | Significantly raised median titres were seen in AS compared with controls (0.7 vs 0.4, p<0 0001).Anti-klebsiella IgA titres in all three states （active,probably active and inactive）were significantly raised when compared with those of the healthy controls but did not differ significantly between each group. |
| Csángó[124] | 1987 | cross-sectional | HLA(+) AS and healthy individuals | The study suggested an enhanced antibody against Chlamydia trachomatis among the HLA-B27 positive individuals whether they have AS or are healthy. |
| Kihlström[125] | 1989 | cross-sectional | AS | Compared with only 5% of blood donors, 33% of those with AS had antibodies to Chlamydia trachomatis at a titre of 1 : 128 or above(p=0.016). |
| Kumar[126] | 2014 | cross-sectional | AS | There was no correlation between the serological results of patients with ReA or uSpA(12.5% vs 13.6%). |
| Tsuchiya[127] | 1990 | cross-sectional | AS | A higher percengtage of male AS with elevated anti-YOPI P81 antibody of IgA,IgG,and IgM class patients were showed than healthy male controls. |
| Zambrano-Zaragoza[128] | 2009 | cross-sectional | AS | Twenty out of 28 AS patients (71.4%) recognized a 30-kDa band from S. typhimurium with IgG antibodies.The antibody levels against Salmonella typhimurium were higher in AS patients than in control groups (HLA-B27+ healthy relatives, unrelated healthy subjects and RA patients) for IgA and IgG. |
| de Vries[116] | 2010 | cross-sectional | AS | There were significant differences of IgA ASCA levels and IgG ASCA levels between fCAL-positive and fCAL-negative AS patients(1.9 vs 0.2,p=0.05;11.5 vs 6.3,p=0.003). |
| Andretta[129] | 2012 | cross-sectional | SpA | The study showed 18.6% (13/70) patients was positive for IgA-ASCA in the SpAgroup and 3/57 (5.2%) in the control group (P = 0.031).There was no relationship between IgA ASCA(+) and disease activity of SpA. |
| Aydin[130] | 2008 | cross-sectional | AS、uSpA | There was an overall increased prevalence of ASCA IgA in AS and uSpA compared with HCs (20.6 and 19.1% vs 5.8%, P=0.0008 and P¼0.02, respectively).ASCA-positive AS patients had higher BASRI scores [7 (2–12) vs 6 (2–12); P=0.037]. |
| Hoffman[131] | 2003 | cross-sectional | SpA | ASCA IgA, but not IgG levels, were higher in SpA than in both healthy controls.A weak, but significant correlation was found between ASCA IgA and CRP levels (r=0.307, p<0.05) and between ASCA IgA levels and ESR (r=0.332, p<0.05). |
| Mundwiler[132] | 2009 | cross-sectional | AS | ASCA IgG, ASCA IgG and IgA and anti-I2 were significantly higher in AS than in controls. |
| Rodrigues[133] | 2015 | cross-sectional | SpA | Anti-H. pylori+ serology was significantly more frequent in axial SpA patients than in those with CD (52.4 vs. 18.4 %, p < 0.001), while ASCA+ serology was significantly more frequent in CD patients than in SpA patients. |
| Torok[134] | 2004 | cross-sectional | SpA | "ASCA IgA levels but not ASCA IgG were significantly higher in patients with HLA-B27-associated SpA than in healthy controls (p<0.0001)，in particular in AS and uSpA." |
| Wallis[118] | 2013 | cross-sectional | AS | A greater proportion of AS-IBD patients had a quartile score of 4 (highest quartile) than AS patients, for IgA ASCA (36.4% vs 21.1%, P = 0.049), IgG ASCA (35.1% vs 17.9%, P = 0.016), anti-OmpC (37.7% vs 18.4%, P = 0.001) and anti-CBir1 (42.9% vs 19.7%, P = 0.003). That means AS-IBD had elevated responses when compared to AS alone for ASCA, anti-OmpC and anti-CBir. |
| Tani[135] | 1997 | cross-sectional | AS | The AS patients had significantly elevated IgA antibodies to K. pneumoniae LPS (t=2.36, P<0.05), S. enteritidis LPS (t=2.34, P<0.05) and S. typhimurium LPS (t=3.12, P<0.005) when compared to controls,but not E. coli LPS or S. flexneri LPS. |
| Dominguez-López[136] | 2002 | cross-sectional | HLA-B27+ AS | HLA-B27+ patients and healthy individuals, showed signicantly higher IgG antibody levels to the Klebsiella, Yersinia and Salmonella HSPs than HLA-B27- healthy controls. |
| Mäki-Ikola[137] | 1997 | cross-sectional | AS | AS patients had higher IgA class serum antibody concentrations against K pneumoniae when compared with the healthy controls.This increase was seen only in patients with axial type of the disease. |

**Table: Studies addressing antibodies as biomarkers for predicting radiological progression**

| Study | Year | Design | Population | Result |
| --- | --- | --- | --- | --- |
| Lee[138] | 2020 | Cohort | AS | The patients with radiographic progression showed higher baseline anti-PPM1A antibody levels than patients without radiographic progression[48.9 (38.9–84.6) vs 34.3 (21.2–50.2),p=0.005]. |
| Kim[139] | 2014 | Cohort | AS | Levels of anti-PPM1A autoantibodies were significantly higher in the AS patients than in the RA patients or the healthy controls.Levels of anti-PPM1A autoantibodies were significantly higher in the AS patients than in the RA patients or the healthy controls. |

**Question 5**: **CRP**

We found 14 case-control studies, 35 Cohort, 7 Cross-sectional studies and 2 RCT addressing this question.

The evidence shows:

1.C-reactive protein (CRP) is an acute phase reactant reflecting the inflammatory status in patients with axial spondyloarthritis. Elevated levels of CRP (≥10mg/L) could be found in approximately 60% AS patients with active disease.[140, 141] CRP is also a component of ASDAS-CRP.

2.Elevated CRP level is considered a SpA feature by the ASAS classification criteria.[3]

3.According to our meta-analysis, elevated baseline CRP level is an important predictor of radiographic progression.

4.CRP could be used an indicator to monitor therapeutic responses in patients with axSpA.

The quality of evidence is HIGH.

**Table: Evidence profile**

| Certainty assessment | | | | | | | Summary of findings | |
| --- | --- | --- | --- | --- | --- | --- | --- | --- |
| No of participants  (studies)  Follow-up | Risk of bias | Inconsistency | Indirectness | Imprecision | Publication bias | Overall certainty of evidence | Pooled Result (95%CI) | Brief Summary |
| Question 5: | | | | | | | | |
| 58 studies (14 case-control studies, 35 Cohort, 7 Cross-sectional studies and 2 RCT) | Not Serious | Not serious | Not serious | Not serious | Not serious | ⨁⨁⨁⨁  HIGH | \ | C-reactive protein (CRP) reflects the inflammatory status in patients with axial spondyloarthritis. elevated baseline CRP level is an important predictor of radiographic progression. CRP could be used an indicator to monitor therapeutic responses in patients with axSpA. |

**Table: Studies addressing the value of CRP as a biomarker for diagnosis**

| Study | Year | Design | Population | Result |
| --- | --- | --- | --- | --- |
| Akbal[142] | 2016 | case-control study | 40AS,40HC | The presence of the CRP gene CC wild haploid and C allele in patients may indicate an increased risk for AS. |
| Su[143] | 2019 | Cohort study | 129681 AS | Baseline hs-CRP was positively associated with the risk of future AS. This is the first study in a community-based cohort to demonstrate that CRP plasma concentrations predict the risk of future AS, thus providing a test that is easy to routinely perform in the clinic to assess for AS risk. |
| Kilic[144] | 2015 | cohort study | 287 axSpA | ASDAS cut-off values are quite similar between groups indicating that ASDAS-CRP works similarly well in nr-axSpA and AS. The performance of ASDAS to discriminate low and high disease activity and cut-off values are quite similar in patients with AS and non-radiographic axial SpA. |
| Li[145] | 2021 | case-control study | 53 JoAS, 53 Nr-axSpA | Peripheral involvement was prevalent in juvenile-onset nr-axSpA. IBP, buttock pain, enthesitis, elevated baseline CRP levels and SIJ-MRI positivity in patients with the disease are associated with higher risk of progression to JoAS. |
| Huang[146] | 2022 | case-control study | 297 axSpA, 71 HC | Platelet, NAR, PAR, MAR, ESR, and CRP were all positively correlated with BASDAI and BASFI (p < .05). Albumin was lower in axSpA of active group, while platelet, NAR, PAR, MAR, ESR, and CRP were higher (p <0 .05). |
| Tang[147] | 2018 | case-control study | 250AS, 250HC | There were significant differences of the level of WBC (white blood cell), Platelets, CRP (C-reactive protein) and ESR (erythrocyte sedimentation rate) between AS patients and controls (Pall<0.05). |
| Sun[148] | 2023 | cohort study | 196AS | CRP, NEU, and MONO were the independent predictive factors for the novel subtype of AS |
| Wang[149] | 2008 | case-control study | 35AS, 15HC | The levels of CRP (20.18 +/- 23.17 mg/l), PLT (259.54 +/- 102.59 x 10(9)/l) and ESR (36.86 +/- 31.23 mm/h) in AS patients were higher than those in normal controls, respectively (3.21 +/- 2.18 mg/l, P < 0.01; 197.00 +/- 55.70 x 10(9)/l, P < 0.01; 12.25 +/- 5.05 mm/h, P < 0.05). |
| Ho[150] | 2000 | case-control study | 24AS, 21HC | In clinical assessments, patients with AS had abnormally raised serum CRP (>10 mg/l) and ESR (>15 mm/1st h) levels. |
| Ozgocmen[151] | 2007 | cohort study | 27 AS | Patients with peripheral joint involvement had significantly higher ESR and CRP levels. |
| Kang[152] | 2014 | cohort study | 298 AS | Multivariate logistic regression analysis showed that previous VFs at baseline and increased CRP levels at 2 years were predictors of new VFs (odds ratio (OR) =12.8, 95% confidence interval (CI) = 3.6-45.3 and OR = 5.4, 95% CI = 1.4-15.9). Previous VFs and increased CRP levels predicted future VFs. |
| Komsalova[153] | 2020 | case-control study | 47SpA, 86 Not SpA | Factors forecasting positive diagnosis were IBP, followed by SpA features and increased CRP. |
| Nazıroğlu[154] | 2011 | case-control study | 13AS, 13HC | Erythrocyte sedimentation ratio, C-reactive protein level, Bath AS disease activity index and bath AS functional index were higher in patients with AS than in controls. |
| Zwolak[155] | 2019 | cohort study | 82AS | Currently, HLA B27 antigen and C-reactive protein are the two most commonly used biomarkers for diagnostic and disease activity monitoring purposes of axSpA and magnetic resonance is the only "imaging biomarker". |
| Sahli[156] | 2019 | cross sectional | 60 SpA | Foot involvement was correlated with C Reactive protein (p=0.043). Foot involvement and foot symptoms were seen frequently in spondyloarthritis and it is associated with late onset of the disease and with higher inflammation in blood tests. |
| Hirano[157] | 2022 | cohort study | 708 SpA | Higher CRP level, and impaired spinal mobility contribute to explaining PhGA in patients with early axial SpA, |
| Poddubnyy[158] | 2011 | cohort study | 210 axSpA | An elevated level of CRP was found to be a strong positive predictor of sacroiliitis progression. |
| Navarini[159] | 2020 | cohort study | 133 AS | Feature analysis showed that C-reactive protein (CRP) has the highest importance, while SBP and hypertension treatment have lower importance. |
| Weiss[41] | 2016 | cross-sectional | 40 JSpA, 14HC | Active sacroiliitis by MRI is common at diagnosis in juvenile SpA and is frequently asymptomatic. Children who are HLA-B27-positive and have elevated CRP levels have the highest probability of sacroiliitis. |
| Kim[160] | 2021 | cohort study | 43 axSpA | Increases in BMD in the lumbar spine were correlated with reductions in ESR (r = 0.40, P = 0.02) and C-reactive protein (CRP) (r = 0.40, P = 0.02). Increases in BMD in the total hip were correlated with reductions in CRP (r = 0.38, P = 0.03). |
| Braga[161] | 2020 | cohort study | 45 PsA | CRP levels were higher among sacroiliitis patients (p = 0.028), and time of psoriasis was positively associated with chronic lesions (p = 0.006). Sacroiliitis on MRI was highly prevalent in our sample of PsA patients. Raised CRP levels were significantly associated with sacroiliitis, and longer time of psoriasis was predictive of chronic sacroiliitis lesions. |
| Chen[162] | 2011 | cohort study | 531 AS | Patients who had longer disease duration, elevated C-reactive protein levels, advanced sacroiliitis, and radiographic hip involvement were significantly more likely to have spinal fusion (P < 0.05). Elevated C-reactive protein levels and advanced sacroiliitis were also significantly associated with the presence of spinal involvement without fusion (P < 0.05). Early disease onset and more radiographic severity in the spine and sacroiliac joints were the predictors of radiographic hip involvement (P < 0.05). |

**Table: Studies addressing the value of CRP as a biomarker for indicating disease activity**

| Study | Year | Design | Population | Result |
| --- | --- | --- | --- | --- |
| Wu[163] | 2021 | Case-control | 136AS, 63HC | Systemic immune‐inflammation index levels were higher in AS patients than in healthy controls (p < 0.001). SII levels were higher in the active group than in the remission group (p < 0.001). For patients with AS, SII correlated positively with CRP (rs = 0.483, p < 0.001), ESR (rs = 0.374, p < 0.001), and BASDAI (rs = 0.667, p < 0.001). |
| Kwan[164] | 2019 | Cross-sectional | 280 axSpA | This study supports the use of both ASDAS-CRP and BASDAI in measuring disease activity in patients with axSpA in Singapore |
| Sundaram[165] | 2020 | Cohort study | 107 AS | SASDAS-CRP showed better internal consistency than SASDAS-ESR and correlated better with ASDAS-CRP in late disease (Z = 3.04; p = 002) and those with adult onset disease (Z = 2.18; p = 0.03). SASDAS with CRP performs better than SASDAS-ESR, pending further validation. |
| Navarini[166] | 2022 | Cohort study | 295 axSpA | This study suggests that persistence of increased CRP levels and high disease activity may be considered biomarkers to identify those axSpA patients at higher risk of CVD. |
| Cowling[167] | 1980 | Cohort study | 469 AS | CRP appears to be a useful marker of disease activity in ankylosing spondylitis. |
| Bedaiwi[168] | 2021 | cross-sectional | 106 SpA | In patients with AS, CRP and age significantly impact disease activities (p<0.05). |
| Chen[169] | 2015 | Cohort study | 156 AS | ESR, CRP, and disease duration are particularly related to AS patient's poor physical mobility. Combining the usefulness of acute-phase reactants and disease duration, the values of ESR × disease duration and CRP × disease duration demonstrate better association with poor physical mobility in AS patients. |
| Benhamou[141] | 2010 | RCT | 851 axial AS | Increased CRP was frequently observed in patients with painful axial AS and was correlated both with activity and functional severity of the disease. |
| Siebuhr[170] | 2019 | case-control study | 193 axSpA, 100HC | CRPM was associated with disease activity in axSpA, and CRPM and VICM separated the axSpA groups. This study indicates that serological biomarkers may be novel biomarkers in axSpA. |
| Senna[171] | 2012 | case-control study | 40 AS, 40 HC | ESR and CRP are significantly higher in the AS patients compared with the controls (P < 0.001). AS patients with active disease had significantly higher ESR (P = 0.0151), CRP (P = 0.0124), and BASFI (P = 0.0016). |
| Toldi[172] | 2013 | case-control study | 33AS, 29HC | CRP and ESR values were higher in AS patients than in healthy individuals.  To assess the inflammatory status in AS, ESR and particularly CRP values are still more appropriate clinical markers. |
| Zwolak[155] | 2019 | cohort study | 82AS | Currently, HLA B27 antigen and C-reactive protein are the two most commonly used biomarkers for diagnostic and disease activity monitoring purposes of axSpA and magnetic resonance is the only "imaging biomarker". |
| Mlcoch[173] | 2017 | cohort study | 313 AS | We showed that BASFI and ASDAS-CRP are very strong, robust predictors of EQ-5D utilities in all regression specifications together with sex (female), invalidity, and activity impairment. |
| Seng[174] | 2018 | cross-sectional | 122 axSpA | Patients with very high disease activity had higher ESR and CRP compared to patients with inactive disease and moderate disease activity (p < 0.05). |
| Londono[175] | 2012 | case-control study | 62 SpA, 46HC | An increase in serum levels of US-CRP, IL-6, IL-1α , and LBP was correlated with factors associated with clinical activity and poor prognosis in spondyloarthritis. |
| Bansal[176] | 2017 | cohort study | 254 AS | SASDAS-ESR and SASDAS-CRP are reliable, easy-to-calculate scores for disease activity assessment in Asian Indian AS patients; which can be used in daily clinical practice. |
| Yildirim[177] | 2004 | cohort study | 20 AS | Our data suggest that CRP is a better marker of disease activity than ESR, Hp, and beta2MG. |
| Wendling[178] | 2017 | cohort study | 708 SpA | In this population suggestive of early SpA, smoking and CRP appear as major markers of disease activity in early SpA. |

**Table: Studies addressing the value of CRP as a biomarker for predicting therapeutic effect of bDMARDs**

| Study | Year | Design | Population | Result |
| --- | --- | --- | --- | --- |
| Ben-Shabat[179] | 2022 | case-control study | 5930 AS, 29018 HC | Age, male sex, mean C-reactive protein (CRP) levels and general comorbidities were predictors of mortality within the AS cohort. |
| Benhamou[141] | 2010 | RCT | 851 axial AS | Such a treatment effect was of higher magnitude in the subgroup of patients with increased CRP and usually of lower magnitude than the treatment effect of other outcome variables such as BASDAI and BASFI. Increased CRP was frequently observed in patients with painful axial AS and was correlated both with activity and functional severity of the disease. The treatment effect of NSAIDs/coxibs was relevant in the subgroup of patients with increased CRP at baseline. |
| de Vries[180] | 2009 | cohort study | 155 AS | This study demonstrates that inflammatory markers, and notably CRP and SAA, may facilitate patient selection and monitoring of efficacy of anti-TNF treatment in AS, and could be added to response criteria. |
| Iervolino[181] | 2012 | cohort study | 146 PsA | In patients with PsA, age, CRP, and BASFI at the beginning of treatment were found to be reliable predictors of MDA after 3 months of TNF-α blocker therapy. |
| Li[182] | 2020 | cross-sectional | 167 AS | Clinicians should identify high-risk patients with low chronic pain acceptance and high levels of serum CRP, and give psychological and pharmacological intervention promptly. Moreover, the combination of baseline chronic pain acceptance and serum CRP level could be used to predict the treatment response in AS patients initiating biologics treatment. |
| Sebastian[183] | 2017 | cohort study | 65 axSpA | Changes in the values of disease activity indicators (CRP, ESR) correlated with more stable response to TNFi therapy. |
| Zong[184] | 2022 | RCT | 125 axSpA | Controlling inflammation, especially reducing CRP and ASDAScrp levels, is a key factor for achieving clinical and imaging remission in patients with axSpA. |

**Table: Studies addressing the value of CRP as a biomarker for predicting radiological progression**

| Study | Year | Design | Primary diagnosis | patients number | mean age | male proportion | definition of radiological progression | follow-up time | OR (95% CI) |
| --- | --- | --- | --- | --- | --- | --- | --- | --- | --- |
| Webers[185] | 2015 | cohort | AS | 216 | 43.6 | 154/216 | mSASSS progression | 8.3y | 0.91 (0.86, 0.97) |
| Syrbe[186] | 2015 | cohort | AS | 86 | 38.1 | 56/86 | mSASSS worsening of 2 units | 2y | 4.1 (1.2, 14) |
|  |  |  |  |  |  |  | syndesmophyte formation/progression | 2y | 1.3 (0.48, 3.4) |
| Kang[187] | 2015 | cohort | axSpA | 110 | 31.6 | 83/110 | mSASSS worsening of 2 units/2y | 2y | 2.59 (1.04, 6.44) |
| Poddubnyy[188] | 2016 | cohort | axSpA | 178 | 38.1 | 89/178 | mSASSS worsening of 2 units/2y | 2y | 1.04 (0.99, 1.08) |
|  |  |  |  |  |  |  | syndesmophyte formation/progression | 2y | 1.07 (1.01, 1.13) |
| Kim[189] | 2016 | cohort | AS | 610 | 39 | 541/610 | mSASSS progression | 5y | 1.06 (0.99, 1.13) |
| Kang[190] | 2014 | cohort | AS | 67 | 35.3 | 0/67 | syndesmophyte formation | 2y | 4.7 (1.1, 21) |
| Sohn[191] | 2018 | cross-sectional | AS | 55 | 37.8 | 55/55 | syndesmophyte presence | - | 1.04 (0.56, 1.93) |
| Deminger[192] | 2018 | cohort | AS | 204 | 50 | 89/204 | mSASSS worsening of 2 units/5y | 5y | 1.06 (1.02, 1.11) |
|  |  |  |  |  |  |  | syndesmophyte formation | 5y | 1.02 (0.99, 1.06) |
| Pedersen[193] | 2018 | cohort | AS | 33 | 40.3 | 26/33 | mSASSS progression | 5y | 1 (0.98, 1.02) |
|  |  |  |  |  |  |  | spine nbf | 5y | 1 (0.98, 1.02) |
|  |  |  |  |  |  |  | progression in sij score | 5y | 1.02 (0.99, 1.04) |
|  |  |  |  |  |  |  | sij score progression >=1 | 5y | 1.02 (0.99, 1.04) |
| Jeong[194] | 2015 | cohort | AS | 47 | - | 47/47 | mSASSS worsening of 1 units>1y | - | 1.13 (0.93, 1.38) |
| Poddubnyy[195] | 2012 | cohort | axSpA | 210 | 37.1 | 107/210 | mSASSS worsening of 2 units/2y | 2y | 2.47 (1.12, 5.44) |
| Poddubnyy[158] | 2011 | cohort | axSpA | 210 | 37.1 | 107/210 | worsening of sacroiliitis by at least one grade of either the right or left sacroiliac joint | 2y | 3.23 (1.37, 7.59) |
|  |  |  | nr-axSpA | 95 | 38.7 | 32/95 | worsening of sacroiliitis by at least one grade of either the right or left sacroiliac joint | 2y | 3.65 (1.19, 11.15) |
|  |  |  | AS | 115 | 36.8 | 75/115 | worsening of sacroiliitis by at least one grade of either the right or left sacroiliac joint | 2y | 5.08 (1.02, 25.38) |
| Huerta-sil[196] | 2006 | cohort | undifferentiated spA | 50 | 26.5 | 26/50 | progression to definite radiographic sacroiliitis | 3-5y | 4.71 (0.94, 23.68) |
| Braun[197] | 2016 | cohort | AS | 356 | - | - | syndesmophyte formation | 208w | 1.155 (1.011, 1.319) |
|  |  |  |  |  |  |  | mSASSS worsening of 2 units | 208w | 1.059 (0.926, 1.212) |

**
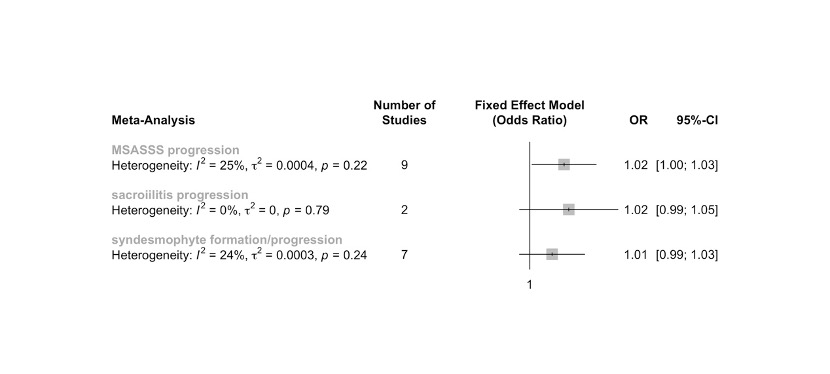
**

**Figure: Forest Plot of odds ratio: CRP and the risk of radiological progression in patients with axSpA**

**Question 6**: **ESR**

We found 5 case-control studies, 3 cross-sectional studies, 18 cohort studies and 5 clinical trials addressing this question.

The evidence shows:

1.Erythrocyte sedimentation rate (ESR) is also an indicator of inflammation. Elevated ESR could be found in approximately 70% AS patients with active disease.[180]

2.According to our meta-analysis, elevated baseline ESR is also an important predictor of radiographic progression in axSpA.

The quality of evidence is HIGH.

**Table: Evidence profile**

| Certainty assessment | | | | | | | Summary of findings | |
| --- | --- | --- | --- | --- | --- | --- | --- | --- |
| No of participants  (studies)  Follow-up | Risk of bias | Inconsistency | Indirectness | Imprecision | Publication bias | Overall certainty of evidence | Pooled Result (95%CI) | Brief Summary |
| Question 6: | | | | | | | | |
| 31 studies (5 case-control studies, 3 cross-sectional studies, 18 cohort studies and 5 clinical trials) | Not serious | Not serious | Not serious | Not serious | Not serious | ⨁⨁⨁⨁  HIGH | \ | ESR is an indicator of inflammation. Elevated baseline ESR is also an important predictor of radiographic progression in axSpA. |

**Table: Studies addressing the value of ESR as a biomarker for indicating disease activity**

| Study | Year | Design | Population | Result |
| --- | --- | --- | --- | --- |
| Wu[163] | 2021 | case-control | 136 AS, 63 HC | For patients with AS, systemic immune-inflammation index correlated positively with CRP (rs= 0.483, p < 0.001), ESR (rs= 0.374, p < 0.001), and BASDAI (rs= 0.667, p < 0.001). |
| Kim[198] | 2020 | cross-sectional | 200 AS, 130 OA | ESR correlated significantly with CRP level in AS group (p < 0.001). |
| de Vires[180] | 2009 | cohort | 155 AS | ESR, CRP, and SAA were significantly associated with the BASDAI over 3 months, and the association with ESR was the strongest. |
| Jung[199] | 2007 | case-control | 38 AS, 38 HC | The correlation between ESR and CRP levels also appeared to be significant (r = 0.703, p < 0.001). |
| Borman[200] | 2001 | case-control | 32 AS, 30 HC | The levels of ESR and BASDAI scores (p<0.05, r = 0.42), and CRP and P (p <0.05, r =0.40), correlated significantly with each other. |
| Hussein[201] | 1987 | cohort | 12 JSpA | CRP and ESR were elevated in clinically active disease in all subgroups and were significantly higher than in moderately active and inactive disease stages (p<0.001). The clinical disease activity grades showed a close relationship to grouped values of (p<0.0005) and ESR (p<0.0005). A close correlation between CRP and ESR was present in the whole group (p<0.001). |
| Sheehan[202] | 1986 | cross-sectional | 65 AS | Positive correlations were found between ESR and the acute phase proteins (APP), CRP, orosomucoid and α1AT, but none of these variables correlated with the clinical assessment of activity. |
| Cowling[167] | 1980 | case-control | 149 AS | "The differences in mean ESR value between active and inactive patients (P <0.001) and probably active and inactive patients (P <0.001) were statistically significant. Individual values of high or low ESR were closely correlated with high or low values in serum CRP irrespective of disease activities (P <0.001). |

**Table: Studies addressing the value of ESR as a biomarker for predicting therapeutic effect of bDMARDs**

| Study | Year | Design | Population | Result |
| --- | --- | --- | --- | --- |
| Wang[203] | 2022 | clinical trial | 180 AS | After treatment, the morning stiffness duration, visual analog scale score for low back pain, bath ankylosing spondylitis disease activity index and serum C-reactive protein, interleukin-6, tumor necrosis factor alpha and erythrocyte sedimentation rate levels in both groups were decreased compared with the conditions before treatment (p<0.05). |
| Alegre-Sancho[204] | 2021 | cohort | 131 axSpA, 79 PsA | In axial SpA patients treatede with golimumab as a second TNFi drug, ESR (n=119) decreased from baseline 22.4 (24.8) mm/h to 13.0 (12.2) and 10.8 (9.8) mm/h at month 3 and year 1, respectively. Similarly, in PsA patients, ESR (n=66) decreased from 18.9 (20.0) mm/h at baseline to 14.1 (17.7) and 12.9 (13.4) mm/h at month 3 and year 1, respectively. |
| Zhang[205] | 2021 | cohort | 165 SpA treated with TNFi,  74 SpA received basic treatment | For all patients receiving treatment with TNF-a inhibitors, all continuous parameters related to disease activity (i.e., CRP, ESR, BASDAI, and ASDAS) and hip joint function (i.e., Harris) were significantly ameliorated from week 12 (all p < 0.001) compared with baseline, and the significant remission levels persisted until week 52 (all p < 0.001). |
| You[206] | 2020 | clinical trial | 90 AS | After 24-week treatment, differences of ESR (P=0.005) and CRP (P=0.021) were observed among needle-knife combined with etanercept group (NKCE), needle-knife group (NK), and control groups according to three-group comparison analysis. The subsequent two-group analysis dis played that mean ESR was reduced in NKCE group compared to NK (P<.05) and control (P<.05) groups. |
| Xu[207] | 2019 | case-control | 232 AS, 314 HC | The clinical indicators of AS patients including morning stiff ness time, Bath AS function index (BASFI), Bath AS activity index (BASDAI), Visual Analogue Scale (VAS), erythrocyte sedimentation rate (ESR), and CRP were all significantly decreased after 12 weeks of etanercept treatment (P < 0.05). |
| Dong[208] | 2019 | cohort | 60 AS, 24 HC | After 12 weeks of TNFi treatment, good responders had lower baseline IL-6 level and erythrocyte sedimentation rate (ESR) than non/poor responders. The cut off value of baseline IL-6 level and ESR to predict clinical response of TNF inhibitor treatment were 9.05 pg/mL and 47.00 mm/h, respectively. Binary logistic regression found that baseline IL-6 levels and ESR had an adverse relationship with clinical response, and the combination of IL-6 level and ESR could predict clinical response more effectively. |
| Gentileschi[209] | 2018 | cohort | 21 axSpA | In axSpA treated with secukinumab, the mean ± SD ESR levels were 32.95 ± 32.04 mm/h at the start of treatment and 21.29 ± 18.82 mm/h at the 3 month follow-up visit, thus showing a statistically significant difference (P = 0.008). |
| Wei[210] | 2018 | clinical trial | 54 nr-axSpA treated with etanercept, 57 nr-axSpA treated with placebo | Statistically significant improvements from baseline to week 12 were observed in patients treated with etanercept compared with those receiving placebo for both inflammation markers (Fig. 3), hsCRP (P = 0.032), and ESR (P = 0.008). |
| Sebastian[183] | 2017 | cohort | 65 axSpA | Changes in the values of disease activity indicators (CRP, ESR) correlated with more stable response to TNFi therapy. At baseline higher levels of CRP and ESR were observed in patients with relapse of the disease at the end of TNFi treatment and with low disease activity shorter than 6 months. |
| Lubrano[211] | 2016 | cohort | 174 axSpA | Median values of ESR and CRP, continuous NSAID intake, and ASDAS-CRP value ≥ 0.8 were significantly related with both loss of remission and disease flare after adalimumab, entanercept or infliximab therapy. ESR was also significantly indicative of these events when using the cutoff value of 15 mm/h. |
| Mok[212] | 2015 | clinical trial | 20 axSpA treated with golimumab, 10 axSpA treated with pamidronate | At week 48, the BASDAI and BASFI scores, along with CRP and ESR levels, also improved significantly with golimumab treatment but not with pamidronate (P < 0.01). |
| Kneepkens[213] | 2015 | cohort | 115 AS | There was a statistically significant association of adalimumab levels with ESR in generalised estimating equation analysis. CRP, ESR and BASDAI of most patients decreased during follow-up, but in 11 patients with high antidrug antibodies titres and no detectable adalimumab levels CRP and ESR remained high during follow-up. |
| Korkosz[214] | 2013 | cohort | 40 AS | Both inflammatory markers (ESR, CRP) and clinical activity assessed with the BASDAI decreased significantly after 6 months of treatment in the TNF inhibitor-treated group (all p < 0.0001). |
| Paramarta[215] | 2012 | clinical trial | 20 SpA treated with adalimumab, 20 SpA treated with placebo | At week 12, also the improvement in the physician’s global assessment, BASDAI, ASDAS and ESR was significantly greater in the adalimumab treated patients compared with placebo (p<0.05 for all parameters). After the 12-week placebo-controlled phase all patients were treated in an open label phase with adalimumab for an add itional 12 weeks. In the original placebo group, adalimumab treatment induced a significant decrease of all clinical disease activity parameters between weeks 12 and 24 (p<0.05 for all parameters). Additionally, ESR was signifi cantly suppressed (p<0.05). |
| Sandhya[216] | 2011 | cohort | 24 SpA | In patients treated with infliximab, mean ESR at baseline, 1 month, 3 months and last follow-up were 60.37, 18.25, 27.22 and 44.68 mm/h, respectively. Statistically significant reduction in ESR was noted at 1 month (p<0.001) and 3 months (p=0.008) as compared to baseline. |
| de Vires[180] | 2009 | cohort | 155 AS | after 3 months of etanercept or infliximab treatment, ESR decreased significantly (P < 0.0001). |

**Table: Studies addressing the value of ESR as a biomarker for predicting radiological progression**

| Study | Year | Design | Primary diagnosis | patients number | mean age | male proportion | definition of radiological progression | follow-up time | OR (95% CI) |
| --- | --- | --- | --- | --- | --- | --- | --- | --- | --- |
| Kim[217] | 2018 | cohort | axSpA | 119 | 35 | 91/119 | mSASSS worsening of 2 units/2y | 2y | 1.1 (0.5, 2.4) |
| Sohn[191] | 2018 | cross-sectional | AS | 55 | 37.8 | 55/55 | syndesmophyte presence | \ | 1.01 (0.98, 1.04) |
| Deminger[192] | 2018 | cohort | AS | 204 | 50 | 89/204 | mSASSS worsening of 2 units/5y | 5y | 1.02 (0.99, 1.05) |
|  |  |  |  |  |  |  | syndesmophyte formation | 5y | 0.99 (0.95, 1.02) |
| Jeong[194] | 2015 | cohort | AS | 47 | \ | 47/47 | mSASSS worsening of 1 units>1y | \ | 1.01 (0.99, 1.02) |
| Webers[185] | 2015 | cohort | AS | 216 | 43.6 | 154/216 | mSASSS progression | 8.3y | 0.99 (0.92, 1.06) |
| Haroon[218] | 2013 | cohort | AS | 334 | 40.7 | 256/334 | mSASSS worsening of 1 units>1y | 2.87y | 1.02 (1.01, 1.04) |
| Poddubnyy[195] | 2012 | cohort | axSpA | 210 | 37.1 | 107/210 | mSASSS worsening of 2 units/2y | 2y | 4.04 (1.82, 8.97) |
| Poddubnyy[158] | 2011 | cohort | axSpA | 210 | 37.1 | 107/210 | worsening of sacroiliitis by at least one grade of either the right or left sacroiliac joint | 2y | 1.72 (0.71, 4.17) |
|  |  |  | nr-axSpA | 95 | 38.7 | 32/95 | worsening of sacroiliitis by at least one grade of either the right or left sacroiliac joint | 2y | 1.59 (0.44, 5.72) |
|  |  |  | AS | 115 | 36.8 | 75/115 | worsening of sacroiliitis by at least one grade of either the right or left sacroiliac joint | 2y | 2.12 (0.64, 9.12) |


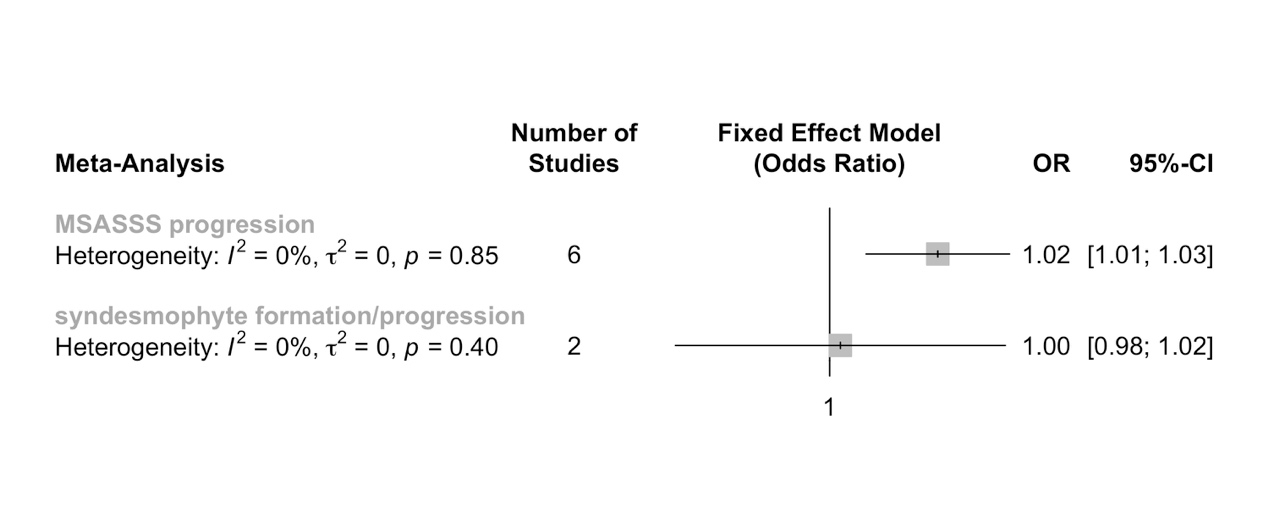


**Figure: Forest Plot of odds ratio: ESR and the risk of radiological progression in patients with axSpA**

**Question 7: SAA**

We found 3 Cohort and 5Cross-sectional studies addressing this question.

The evidence shows:

1.Serum amyloid A (SAA) is also an acute phase reactant. Multiple studies have revealed that SAA levels are significantly higher in patients with axSpA, especially those with active disease.

2.Studies exhibited moderate diagnostic utility of SAA in axSpA, with sensitivity of approximately 80% and specificity of approximately 80%.[219]

3.SAA is significantly correlated with both BASDAI and CRP, indicating that it is a robust indicator of inflammatory status.

The quality of evidence is MEDIUM.

**Table: Evidence profile**

| Certainty assessment | | | | | | | Summary of findings | |
| --- | --- | --- | --- | --- | --- | --- | --- | --- |
| No of participants  (studies)  Follow-up | Risk of bias | Inconsistency | Indirectness | Imprecision | Publication bias | Overall certainty of evidence | Pooled Result (95%CI) | Brief Summary |
| Question 7: | | | | | | | | |
| 8 studies (3 Cohort and 5Cross-sectional studies) | Not Serious | Not serious | Not serious | Not serious | Not serious | ⨁⨁⨁◯  MEDIUM | \ | SAA is significantly correlated with both BASDAI and CRP, indicating that it is a robust indicator of inflammatory status. Studies exhibited moderate diagnostic utility of SAA in axSpA. |

**Table: Studies addressing the value of SAA as a biomarker for indicating disease activity**

| Study | Year | Design | Population | Result |
| --- | --- | --- | --- | --- |
| Lange[220] | 2000 | cross-sectional | 72 AS (ESR0-20/ESR21-40/ESR>41) | The present study tested,whether serum amyloid A (SAA) could be used as amarker of inammatory disease activity in ankylosing spondylitis (AS). It was found that SAA correlates well with ESR,CRP,and BASDAI. Because of its strong correlation,SAA seems to be an additional very useful disease activity marker. |
| Hu[219] | 2021 | cross-sectional | 78 AS | Not only is SAA a reliable indicator for the presence of AS, it may also be useful for monitoring the activity of this disease |
| Liu[221] | 2020 | cross-sectional | 54 AS/28HC | A panel of the two acute phase proteins (CRP and SAA1) from two cohorts study can differentiate well among stable AS, active AS, and healthy subjects, which will be useful clinically for treatment in AS patients |
| Rademacher[222] | 2019 | cross-sectional | 117 axSpA | Repeated cross-validation analyses revealed one biomarker combination with potential added predictive value in addition to the clinical model: leptin + high molecular weight adiponectin + VEGF. |
| Londono[223] | 2012 | cross-sectional | 62 SPA | In our study population, SAA levels were higher in patients with SpA than in the controls. |

**Table: Studies addressing the value of SAA as a biomarker for predicting radiological progression**

| Study | Year | Design | Population | Result |
| --- | --- | --- | --- | --- |
| Ostensen[224] | 1985 | cross-sectional/ Cohort | 13 AS (pregnancy+ post partum) | .In RA and AS patients, SAA concentrations correlated to disease activity during and after pregnancy.We conclude that SAA is a sensitive and reliable indicator of inflammatory events both in the pregnant and non-pregnant state. |

**Table: Studies addressing the value of ESR as a biomarker for predicting therapeutic effect of bDMARDs**

| Study | Year | Design | Population | Treatment | duration | Result |
| --- | --- | --- | --- | --- | --- | --- |
| de Vries[180] | 2009 | Cohort | 155 AS | etanercept or infliximab | 3 months | SAA were significantly associated with the BASDAI over 3 months |
| van Eijk[225] | 2009 | Cohort | 92 AS | etanercept | 3 months | With anti-TNF treatment, levels of all parameters of inflammation decreased significantly, SAA was present at high levels within HDL particles from AS patients with increased CRP levels and disappeared during treatment, in parallel with declining plasma levels of SAA. |

**Question 8**: **Adipokines**

We found 20 case-control studies, 2 cross-sectional studies, 2 GO-RAISE studies, 1 epidemiological studies and 3 clinical trials addressing this question.

The evidence shows:

1. Adipokines, including leptin, adiponectin and resistin, were secreted by adipocytes and could be involved in metabolic activities and inflammation.[226]

2.Leptin is regarded as a pro-inflammatory cytokine, which could stimulate T lymphocyte proliferation and activation. Meta-analysis revealed that leptin is significantly higher in axSpA patients less than 40 years old and Asian and African patients.[226] Moreover, it has been reported that leptin is a predictor of syndesmophyte formation.[227]

3.Adiponectin is considered an anti-inflammatory cytokine. Meta-analysis showed that adiponectin is significantly higher in patients > 40 years old.[226] More importantly, the high molecular weight adiponectin (HMW-APN) is significantly associated with risks of radiographic progression. Patients with lower levels of HMW-APN is more likely to exhibit new bone formation in the spine.[227]

4.Resistin failed to show association with disease activity or radiographic progression.

The quality of evidence is LOW.

**Table: Evidence profile**

| Certainty assessment | | | | | | | Summary of findings | |
| --- | --- | --- | --- | --- | --- | --- | --- | --- |
| No of participants  (studies)  Follow-up | Risk of bias | Inconsistency | Indirectness | Imprecision | Publication bias | Overall certainty of evidence | Pooled Result (95%CI) | Brief Summary |
| Question 8: | | | | | | | | |
| 28 studies (20 case-control studies, 2 cross-sectional studies, 2 GO-RAISE studies, 1 epidemiological studies and 3 clinical trials) | Not Serious | Not serious | Not serious | Not serious | Serious | ⨁⨁◯◯  LOW | \ | Leptin, adiponectin and resistin could be involved in metabolic activities and inflammation. HMW-APN is significantly associated with risks of radiographic progression. Resistin failed to show association with disease activity or radiographic progression. |

**Table: Studies addressing the value of adipokines as biomarkers for diagnosis**

| Study | Year | Design | Population | Result |
| --- | --- | --- | --- | --- |
| Miranda-Filloy[228] | 2013 | Cross-sectional study | 29 AS | The present study shows that in non-diabetic patients with AS on treatment with infliximab adiponectin and resistin serum levels do not correlate with disease activity. Nevertheless, adiponectin concentration correlates with insulin sensitivity. This finding raises the possibility that low circulating adiponectin concentrations may be involved in the pathogenesis of the CV disease in AS. |
| Gonzalez-Lopez[229] | 2017 | case-control | 48 AS, 41 HC | Elevated leptin concentrations are associated with spinal radiographic damage in patients with AS and can serve as a biomarker. Future studies should evaluate whether leptin might be a potential target for treatments to avoid structural damage. |
| Pishgahi[230] | 2020 | case-control | 31 AS, 35 HC | The higher levels of oxidative stress and immunological inflammatory markers in AS patients with MetS provide further evidences on the oxidative stress and immunological relationship in these patients. |
| Xie[231] | 2022 | Randomization Study | 2252 AS, 227338 HC | In reverse MR analysis, there is little evidence to support the genetic causality between the risk of AS and CA levels. |
| Park[232] | 2009 | case-control | 20 AS, 20 HC | Our results shows that leptin production is increased and its stimulation of PBMCs significantly increased the production of pro-inflammatory cytokines in patients with active AS, suggesting its pro-inflammatory effect in pathogenesis of AS. |
| Kocabas[233] | 2012 | case-control | 30 AS, 30 HC | In conclusion, higher serum resistin levels in patients with AS compared to healthy subjects give clues that resistin could have a role in the pathogenesis of AS. |
| Güler[234] | 2013 | case-control | 108 AS, 65 HC | Plasma leptin was lower in AS patients compared with the control group. This is not correlated with disease activation and medical treatment utilized. |
| Kononoff[235] | 2021 | population-based longitudinal epidemiological study | 58 SpA | Metabolic syndrome was common in patients with newly diagnosed arthritides and associated with higher disease activity and increased leptin levels. Resistin responded to treatment of arthritis in RA and UA, leptin in RA, and adiponectin in SpA. |

**Table: Studies addressing the value of adipokines as biomarkers for indicating disease activity**

| Study | Year | Design | Population | Result |
| --- | --- | --- | --- | --- |
| Toussirot[236] | 2007 | case-control | 53 AS, 35 HC | nkylosing spondylitis patients had no changes in fat mass. Leptin production was reduced in contrast with normal levels of adiponectin. These adipokine results, together with high serum ghrelin levels, may influence the inflammatory response in AS. |
| Elolemy[237] | 2013 | case-control | 25 AS, 20 HC | The associations of significantly increased levels of serum leptin and IL-6 with AS disease activity parameters give clues to their role in the inflammatory process of the disease. Failure to find any correlation between high serum resistin levels and AS disease activity parameters is suggestive of its role in the pathogenesis rather than disease activity. |
| Park[238] | 2007 | case-control | 42 AS, 42 HC | Serum leptin/BMI levels were increased and significantly associated with IL-6 levels and disease activity in men with AS, suggesting a possible role for leptin in the inflammatory reactions of AS. |

**Table: Studies addressing the value of adipokines as biomarkers for predicting therapeutic effect of bDMARDs**

| Study | Year | Design | Population | Result |
| --- | --- | --- | --- | --- |
| Rademacher[239] | 2022 | cohort study | 137 AS | Baseline calprotectin and visfatin were associated with progression after 2 years. sCTX showed a positive, leptin an inverse association with 4-year progression.Independent of known risk factors, serum levels of biomarkers at baseline are able to predict radiographic spinal progression over 2 and 4 years in AS patients on TNFi therapy. |
| Czókolyová[240] | 2021 | cohort study | 17 AS | In this cohort of RA and AS patients, we longitudinally assessed the effects of one-year anti-TNF therapy on metabolic biomarkers in association with markers of disease activity, inflammation, and vascular pathophysiology. Anti-TNF therapy decreased ARE activity, MPO, adiponectin, and chemerin levels after 12 months, while lipids, PON activity, and leptin remained unchanged. Regression analyses indicated associations of IMT, PWV, and FMD indicating vascular pathophysiology with ARE, MPO, leptin, and lipids. On the other hand, these metabolic parameters were significantly associated with disease duration, CV history, CRP, obesity, PWV, and IMT. |
| Derdemezis[241] | 2011 | cohort study | 30 AS, 30 HC | Adiponectin levels were significantly higher in AS patients compared with controls. Infliximab treatment did not change serum levels of leptin and adiponectin suggesting that the anti-TNF-α treatment may not modulate significantly their levels. |
| Miranda-Filloy[242] | 2013 | cohort study | 30 AS | The present study indicates that in non-diabetic patients with AS on treatment with infliximab leptin and visfatin serum levels do not correlate with disease activity or systemic inflammation. Nevertheless, visfatin concentration correlates with insulin resistance. |
| Hulejová[243] | 2011 | cohort study | 26 AS | baseline levels of adipocytokines did not predict the change of disease activity or functional ability. Intensive physiotherapy effectively reduces all clinical measures of disease activity, but it is not associated with a significant change in acute-phase reactants or serum levels of adipocytokines. |
| Inman[244] | 2016 | GO-RAISE study | 356 AS | Extensive serum biomarker multiparametric analyses in golimumab-treated patients with ankylosing spondylitis demonstrated few correlations with disease activity or MRI changes; IL-6 weakly correlated with radiographic progression. |
| Wagner[245] | 2012 | GO-RAISE | 356 AS | Golimumab modulated acute phase reactants and inflammatory markers in patients with active AS. Specific combinations of biomarkers at baseline demonstrated a stronger prediction for clinical efficacy than CRP alone. These data provide insights into the mechanism of golimumab on inflammatory processes driving AS pathology, and may have utility in managing the treatment of patients with AS. |

**Table: Studies addressing the value of adipokines as biomarkers for predicting radiological progression**

| Study | Year | Design | Population | Result |
| --- | --- | --- | --- | --- |
| Rademacher[222] | 2019 | RCT | 117 AS | Biomarkers show potential to improve the prediction of radiographic spinal progression in axial spondyloarthritis when used in addition to the clinical parameters, though the added value seems to be rather small. |
| Sari[246] | 2007 | case-control | 28 AS, 17 HC | Chronic inflammatory condition in AS may be responsible for the reduced body fat content and lower circulating leptin concentrations. Insulin levels and insulin resistance indices seem similar in patients and controls in the absence of classic vascular risk factors. |
| Wang[247] | 2016 | case-control | 120 AS, 100 HC | Our study found that serum vaspin levels were decreased in patients with ankylosing spondylitis and were associated with FMD levels. Vaspin may serve as an independent marker for detecting early stage atherosclerosis in patients with ankylosing spondylitis. |
| Genre[248] | 2020 | case-control | 385 axSpA, 84 HC | In conclusion, in our study low omentin serum levels were associated with CV risk factors in axSpA. Furthermore, rs12409609 minor allele may be downregulating the expression of omentin. These data support a role of omentin as a CV risk biomarker in axSpA. |
| Syrbe[186] | 2015 | case-control | 86 AS, 25 HC | Serum levels of resistin and visfatin are elevated in AS patients. Elevated visfatin levels at baseline are predictive of subsequent progression of radiographic damage in AS patients. |
| Toussirot[249] | 2013 | case-control | 31 AS, 30 RA, 51 HC | The effect of the increased visceral adipose tissue on cardiovascular risk is presumably attenuated by the favorable cardiometabolic profile in women with RA, as suggested by the normal HOMA-IR and atherogenic index. |
| Kim[250] | 2012 | case-control | 72 AS, 20 HC | there was no significant association between serum leptin/BMI ratio and bone mineral density. Serum leptin levels are elevated in male AS patients with syndesmophytes and were found to be correlated with bone formation marker, suggesting a potential role of leptin in new bone formation in AS. |
| Hartl[227] | 2017 | ENRADAS trial | 120 AS | Serum leptin and HMW-APN predict protection from spinal radiographic progression in patients with AS. Women generally have higher leptin and HMW-APN serum levels that might explain why they have less structural damage in the spine as compared to male patients with AS. |
| Toussirot[251] | 2014 | case-control | 12 AS, 8 RA | Long-term TNFα inhibition in RA and AS is associated with a significant gain in fat mass, with a shift to the android (visceral) region. This fat redistribution raises questions about its influence on the cardiovascular profile of patients receiving these treatments. |
| Rueda-Gotor[252] | 2021 | Cross-sectional study | 510 axSpA | Our results revealed that vaspin is linked to CV risk factors that may influence on the atherosclerotic process in axSpA. Additionally, we disclosed that serum vaspin concentration is genetically modulated in a large cohort of patients with axSpA. |

**Question 9**: **VEGF**

We found 6 Cohort and 10 cross-sectional studies addressing this question.

The evidence shows:

1.VEGF is considered a pro-inflammatory cytokine, apart from its capacity of inducing angiogenesis.

2.Previous meta-analysis revealed that VEGF was significantly higher in axSpA patients compared with healthy controls(SMD 0.78, 95% CI [0.23-1.33]).[253] However, studies investigating VEGF association with disease activity were highly inconsistent, undermining its reliability as an indicator of inflammatory status.

3.It could not be confirmed that VEGF could predict radiographic progression.

The quality of evidence is VERY LOW.

**Table: Evidence profile**

| Certainty assessment | | | | | | | Summary of findings | |
| --- | --- | --- | --- | --- | --- | --- | --- | --- |
| No of participants  (studies)  Follow-up | Risk of bias | Inconsistency | Indirectness | Imprecision | Publication bias | Overall certainty of evidence | Pooled Result (95%CI) | Brief Summary |
| Question 9: | | | | | | | | |
| 16 studies (6 Cohort and 10 cross-sectional studies) | Serious | Serious | Not serious | Not serious | Serious | ⨁◯◯◯  VERY LOW | \ | VEGF was significantly higher in axSpA patients compared with healthy controls, but studies investigating VEGF association with disease activity were highly inconsistent. It could not be confirmed that VEGF could predict radiographic progression. |

**Table: Studies addressing the value of VEGF as biomarkers for diagnosis**

| Study | Year | Design | Population | Result |
| --- | --- | --- | --- | --- |
| Goldberger[254] | 2002 | cross-sectional | AS | Levels of VEGF were 75.3 ± 19.0 pg/ml in AS, compared to 13.8 ± 4.7 pg/ml measured in the control group (p=0.001).A significant correlation was found between VEGF of AS patients and the BASMI score (r = 0.665, p=0.013).Disease status of AS appears to be associated with elevated VEGF plasma levels. |
| Drouart[255] | 2003 | cross-sectional | SpA | Serum VEGF levels were significantly higher in SpA patients (316·4 ± 215·6 pg/ml) than in controls (217.3 ± 145.2) (p=0.003).VEGF levels correlated with disease activity indices (BASDAI: r=0.22, p=0.04; ESR: r =0.3, p=0.003; and CRP: r=0.23, p=0.02). |
| Wang[256] | 2016 | cross-sectional | AS | VEGF rs2010963 G/C genotypes and elevated plasma level in AS patients when compared with the controls.This study suggests that VEGF is involved in the immunological or inflammatory process of AS. |

**Table: Studies addressing the value of VEGF as biomarkers for indicating disease activity**

| Study | Year | Design | Population | Result |
| --- | --- | --- | --- | --- |
| Pedersen[257] | 2011 | Cohort | SpA | SpA with very high ASDAS had higher levels of CRP, IL-6 and VEGF compared with patients with moderate/ high ASDAS.Moderate/high responsiveness was seen in CRP, IL-6, VEGF, CTX-II and aggrecan in patients with major improvement of anti-TNF therapy in ASDAS and BASDAI responders. |
| Lin[258] | 2015 | cross-sectional | AS | VEGF were significantly higher in AS patients than those of healthy groups(311.6 ± 24.0 vs. 94.0 ± 8.9 pg/ml, p<0.0001).the serum concentrations of IL-27 and VEGF were not independently correlated with ESR and CRP. |
| Przepiera-Będzak[259] | 2016 | cross-sectional | AS | Serum VEGF levels were higher in AS patients than in controls [396.1 (221.6–676) vs 238.1 (209.5–420.9),p=0.02].Increased serum VEGF levels were associated with progression of the disease assessed by the BASMI. |
| Sakellariou[260] | 2017 | cross-sectional | AS | High VEGF levels significantly with ever smoking, and elevated ESR and CRP (P < 0.05). |
| Torres[261] | 2019 | cross-sectional | AS | Serum VEGF was not higher in the AS patients compared with the HC. ESR, CRP,and WBC were all positively correlated with serum levels of HGF, MMP-3, and VEGF. |

**Table: Studies addressing the value of VEGF as biomarkers for predicting therapeutic effect of bDMARDs**

| Study | Year | Design | Population | Result |
| --- | --- | --- | --- | --- |
| Appel[262] | 2008 | Cohort | SpA | VEGF correlated well to CRP, ESR and BASDAI at baseline and after 2 years.Adalimumab-treated spondyloarthritis patients had a significant decrease of VEGF (P<0.001). |
| Visvanathan[263] | 2008 | cross-sectional | AS | Significantly greater reductions in IL-6, VEGF and CRP were observed at weeks 2 and 24 in the infliximab group compared with the placebo group (p<0.001). |
| Pedersen[264] | 2010 | Cohort | SpA | Patients with SpA had compared with healthy subjects elevated VEGF [105 ng/l (22–752)] vs. [45 (12–351)].After TNF-α therapy ,VEGF levels decreased in clinical responders and persistent reductions over 3 years. |
| Tošovský[265] | 2014 | cross-sectional | AS | In patients treated with TNFα inhibitors, a significantly lower VEGF level was found when compared to untreated patients [140.3 (109.4; 262.2)] vs[261 (172.4; 396.6) pg/ml; p = 0.02)].Serum levels of VEGF correlated with serum levels of CRP (r = 0.56; p = 0.00001) and the BASDAI value (r = 0.33; p = 0.015). |

**Table: Studies addressing the value of VEGF as biomarkers for predicting radiological progression**

| Study | Year | Design | Population | Result |
| --- | --- | --- | --- | --- |
| Seo[266] | 2005 | cross-sectional | AS | In carriers of the AGG haplotype, the frequency of cervical spine involvement was significantly higher (p=0.002) and that of patients showing a BASRI score >6 was also higher (p=0.025).This study demonstrates that polymorphisms of the VEGF gene may contribute to disease severity in AS. |
| Poddubnyy[267] | 2013 | Cohort | axSpA | An elevated serum level of VEGF (>600 pg/mL) is highly specific as a predictor of radiographic spinal progression in patients with axSpA. |
| Braun[268] | 2016 | Cohort | AS | VEGF levels and the mSASSS did not significantly correlate. Logistic regression analyses showed no association between VEGF levels and an increased risk of syndesmophyte formation at weeks 104 and 208. |
| Rademacher[222] | 2019 | cross-sectional | SpA | Patients with radiographic spinal progression had significantly higher serum levels of VEGF.Biomarkers show potential to improve the prediction of radiographic spinal progression in axial spondyloarthritis when used in addition to the clinical parameters. |

**Question 10**: **Calprotectin**

We found 5 case-control studies, 1 cross-sectional study and 1 meta-analysis addressing this question.

The evidence shows:

1.Calprotectin, which could be detected in both serum and fecal sample, is known as an indicator of inflammation. Previous meta-analysis showed that both serum and fecal calprotectin were significantly elevated in SpA patients compared with healthy controls (SMD = 1.49, 95% CI = 0.91 to 2.08; SMD = 2.29, 95% CI = 0.25 to 4.33). More importantly, the correlations of calprotectin with CRP, ESR, BASDAI and BASFI were confirmed.[269]

2.Previous epidemiological study showed that 46.2% of SpA patients exhibited microscopic gut inflammation.[270] However, there is still a lack of non-invasive measure to evaluate the gut inflammation except endoscopy. We believe fecal calprotectin could provide valuable incremental information regarding the gut inflammation in axSpA patients.

The quality of evidence is MEDIUM.

**Table: Evidence profile**

| Certainty assessment | | | | | | | Summary of findings | |
| --- | --- | --- | --- | --- | --- | --- | --- | --- |
| No of participants  (studies)  Follow-up | Risk of bias | Inconsistency | Indirectness | Imprecision | Publication bias | Overall certainty of evidence | Pooled Result (95%CI) | Brief Summary |
| Question 10: | | | | | | | | |
| 7 studies (5 case-control studies, 1 cross-sectional study and 1 meta-analysis) | Not Serious | Not serious | Not serious | Not serious | Not serious | ⨁⨁⨁◯  MEDIUM | \ | Previous meta-analysis showed that both serum and fecal calprotectin were significantly elevated in SpA patients compared with healthy controls. The correlations of calprotectin with CRP, ESR, BASDAI and BASFI were confirmed.. |

**Table: Studies addressing the value of calprotectin as a biomarker of AS**

| Study | Year | Design | Population | Result |
| --- | --- | --- | --- | --- |
| Ma[269] | 2020 | Meta-analysis | - | serum and fecal calprotectin were significantly increased in spondyloarthritis patients, and associated with disease activity. Serum and fecal calprotectin were potential biomarkers for the diagnosis and disease activity of spondyloarthritis. |
| Ercalik[271] | 2021 | cross-sectional | 97AS,49Con | FC levels were not correlated with disease activity in AS. The AS group had a significantly higher FC test positivity than the RA group. In the AS group, NSAID users had significantly higher FC levels than nonusers |
| Genre[272] | 2018 | case-control | 119AS,44nr- axSpA,63HC | The serum MRP8/14 levels in patients were significantly higher than in healthy controls (p <0.0001). Patients with peripheral arthritis (n = 50) had higher levels than those with pure axial disease (n = 49) （p = 0.012）. Levels of MRP8/14 correlated with AS Disease Activity Score (DAS)-CRP (p = 0.02) and CRP (p = 0.01). Baseline levels were higher in treatment responders than in non-responders [p = 0.02]. Change in MRP8/14 levels correlated with change in BASDAI and ASDAS-CRP.MRP8/14 levels may be used as a biomarker for activity, peripheral arthritis, and response to therapy. |
| Huang[273] | 2017 | case-control | 53AS,59nr- axSpA,47HC | Serum calprotectin level was higher in AS and nr-axSpA patients than that in healthy individuals. No difference was observed in calprotectin level between AS and nr-axSpA patients. Elevated calprotectin was positively correlated with ESR, CRP, BASDAI, ASDAS as well as SPARCC scoring and had no correlation with BASFI and mSASSS in these two sub-genotypes. No correlation was observed between calprotectin and Wnt/β-catenin pathway markers. |
| Klingberg[274] | 2012 | case-control | 205AS,80HC | Levels of fecal calprotectin were associated with increasing age, disease duration, ESR, CRP, and serum calprotectin, but not with gastrointestinal symptoms. Fecal calprotectin was higher in patients using NSAIDs, salicylates, and proton pump inhibitors, but lower in patients using methotrexate and infliximab. Serum calprotectin levels were normal or low in 98% of AS patients and not different from the levels in healthy blood donors. Serum calprotectin levels were positively associated with ESR, CRP, WBC, and PLT. |
| Oktayoglu[275] | 2014 | case-control | 31AS,45HC | Mean serum level of calprotectin was significantly higher in the patients with AS compared with healthy controls (P = 0.003). Serum levels of calprotectin did not correlate with Bath AS disease activity index, AS disease activity score, Bath AS functional index, Bath AS radiology index, Bath AS metrology index, modified Schober, chest expansion, AS quality of life questionnaire, erythrocyte sedimentation rate, and C-reactive protein values (P > 0.05). |
| Olofsson[276] | 2019 | case-control | 33AS,78nr- axSpA,35HC | Elevated F-calprotectin (⩾50 mg/kg) was observed in 27% of nr-axSpA patients, 38% of AS patients and 6% of controls. F-calprotectin was significantly higher in AS vs nr-axSpA [P = 0.037], and in each axSpA subtype vs controls. |

**Question 11: non-coding RNA**

We found 2 Cohort, 25 case-control studies and 1 Cross-sectional studies addressing this question.

The evidence shows:

1.According to the systemic literature review, multiple non-coding RNAs were found to be deregulated in the serum or PBMCS of patients with ankylosing spondylitis or axSpA, including microRNA (miR-29, miR-214, miR-145 and let-7i), lncRNA (TUG1 and LINC00311) and circRNA (circPTPN22, hsa_circ_0005918). Pooled sensitivity and specificity of the miRNAs were 0.76 (0.70-0.81) and 0.80 (0.74-0.85), suggesting modest diagnostic utility.[277]

2.However, studies investigating the non-coding RNAs and disease activity or therapeutic responses were highly inconsistent, with few repetitions. Another major concern for this field of studies is the considerable amount of retracted articles, giving rise to the doubt regarding the reliability of microRNAs in the diagnosis and evaluation of axSpA.

The quality of evidence is VERY LOW.

**Table: Evidence profile**

| Certainty assessment | | | | | | | Summary of findings | |
| --- | --- | --- | --- | --- | --- | --- | --- | --- |
| No of participants  (studies)  Follow-up | Risk of bias | Inconsistency | Indirectness | Imprecision | Publication bias | Overall certainty of evidence | Pooled Result (95%CI) | Brief Summary |
| Question 11: | | | | | | | | |
| 28 studies (2 Cohort, 25 case-control studies and 1 Cross-sectional studies) | Not Serious | Not serious | Not serious | Not serious | Not serious | ⨁◯◯◯  VERY LOW | \ | multiple non-coding RNAs were found to be deregulated in the serum or PBMCS of patients with ankylosing spondylitis or axSpA, including microRNA, lncRNA and circRNA. Studies investigating the non-coding RNAs and disease activity or therapeutic responses were highly inconsistent. |

**Table: Studies addressing the value of non-coding RNA as a biomarker for diagnosis**

| Category | Study | Year | Design | Population | RNA | Result |
| --- | --- | --- | --- | --- | --- | --- |
| microRNA | Fotoh[278] | 2020 | cross-sectional study | 55 AS  55 HC | iRNA-451a, miRNA-125a | miRNA-125a and miRNA-451a as potential diagnostic biomarkers discriminating AS patients from healthy controls, ROC curve analysis was performed. Both miRNA-125a and miRNA-451a had an area under the curve (AUC) of 0.788 and 0.802, respectively, and the optimal cutoff points of them were 3.029 and 3.372, respectively. miRNA-125a had sensitivity and specificity of 70.91% and 83.64%, respectively. miRNA-451a had sensitivity and specificity of 65.45% and 78.18%, respectively |
|  | Liu[279] | 2020 | case study | 32 AS  24 HC | miR-214 | the serum level of miR-214 might be of potential diagnostic value for AS. |
|  | Ni[280] | 2020 | Case-control | 150 AS  150 HC | miR-495 | The ROC curves of miR-495 suggested that it is a very specific and sensitive biomarker for AS diagnosis. |
|  | Perez-Sanchez[281] | 2017 | Case-control | 53 AS  57 HC | miR-146a-5p, miR-125a-5p, miR-151a–3p, miR-22–3p,miR-451a | The ROC curve for the 6-miRNA signature revealed a marked diagnostic accuracy, evidenced by the AUC of 0.957 (P<0.001), which was much better than any individual miRNA in detecting AS |
|  | Tan[282] | 2021 | Case-control | 44 AS  56 HC | miR-146a, miR-125a-5p, miR-125b-5p, miR-499a, miR-155a | Logistic regression analysis with receiver operating characteristic (ROC) curves showed that combined miR-146a/miR-125a-5p/miR-125b-5p/miR-499a/miR-155a (area under curve [AUC] = 0.824, 95% confidence interval [CI] = 0.727–0.921) had high sensitivity and specificity for AS diagnosis. |
|  | Li[283] | 2017 | Case-control | 59 axSpA  39 HC | miR‐17‐5p, miR‐27a, miR‐29a, miR‐126‐3p | Elevated miR‐17‐5p, miR‐27a, miR‐29a and miR‐126‐3p expression in PBMCs of patients with axSpA, and the expression of these four miRNAs might be used as useful diagnostic markers in axSpA. |
|  | Huang[284] | 2014 | Case-control | 122 AS  122 HC | miR-21 | The expression levels of miR-21 in whole blood yielded an AUC value of 0.757 (95% CI 0.698–0.817) in distinguishing patients with AS from controls |
| lncRNA | Lan[285] | 2018 | Case-control | 82 AS  32 HC | TUG1 | AUC of TUG1 expression in serum was 0.7968 with standard error of 0.04158 and 95% confidence interval of 0.7153 to 0.8783 (p < 0.0001). In addition, AUC of TUG1 expression in biopsies for the diagnosis of AS was 0.8911 with standard error of 0.03767 and 95% confidence interval of 0.8172 to 0.9649 (p < 0.0001). |
|  | Zhong[286] | 2019 | Case-control | 80 AS  22 LBP  20 HC | LINC00311 | Area under the curve was 0.9041, with standard error of 0.02268 and 95% confidence interval of 0.8569–0.9459 (Fig. 2, p < 0.0001). Therefore, plasma LINC00311 may be a potential diagnostic marker for AS. |
|  | Wang[287] | 2022 | Case-control | 20 AS  20 HC | linc00304  linc00926  MIAT | The area under the curve (AUC) of serum linc00304 level in the diagnosis of AS was 0.687 (cutoff value: 0.413, specificity: 0.423, sensitivity: 0.900). AUC of linc00926 was 0.664 (cutoff value: 0.299, sensitivity: 0.882, specificity: 0.417). AUC of MIAT was 0.623 (cutoff value: 0.432, specificity: 0.443, sensitivity: 0.890) (all P <0.05). |
| circRNA | Wang[288] | 2021 | Case-control | 9 AS  12 HC | circPTPN22  hsa_circ_0005918 | The expression of hsa_circ_0000110 (circPTPN22) and hsa_circ_0005918 (circFCHSD2) was down-regulated. |
|  | Tang[289] | 2021 | Case-control | 60 AS  30 HC | hsa_circRNA_001544  hsa_circRNA_102532 | ROC curve analysis showed that hsa_circRNA_001544 (95% CI = 0.610–0.831, P < 0.05) and hsa_circRNA_102532 (95% CI = 0.521–0.762, P < 0.05) were statistically significant, and their area under curve (AUC) values were 0.720 and 0.642, respectively. |
|  | Luo[290] | 2020 | Case-control | 46 axSpA  46 SLE  25 HC | hsa_circ_0079787 | ROC curve analysis suggested that hsa_circ_0079787 and the combination of hsa_circ_0079787‐PLT‐MPV‐PCT had a significant diagnostic value for axSpA. |

**Table: Studies addressing the value of non-coding RNA as a biomarker for indicating disease activity**

| Category | Study | Year | Design | Population | RNA | Result |
| --- | --- | --- | --- | --- | --- | --- |
| microRNA | Qian[291] | 2016 | Case-control | 80 AS  78 HC | miR-155 | the expression level of miR-155 is suggested to be associated with disease activity and the severity of thoracolumbar kyphosis secondary to AS. |
|  | Wang[292] | 2017 | Case-control | 40 AS  40 HC | miR-31, miR-155, miR-16 | the expression levels of miR-31, miR-155, and miR-16 in PBMCs were significantly positively correlated with the ESR in new AS patients but not old AS patients. |
|  | Wang[293] | 2017 | Case-control | 41 AS  36 HC | MiRNA-199a-5p | MiRNA-199a-5p expression levels also showed significant negative correlations with the Ankylosing Spondylitis Disease Activity Score (ASDAS) and modified Stoke Ankylosing Spon dylitis Spinal Score (mSASSS) of AS patients. |
|  | Wei[294] | 2017 | Case-control | 45 AS  30 HC | miR-146a | expression level of miR‑146a in PBMC of patients with AS was positively correlated with BASDAI, ESR, CRP and duration of morning stiffness (r=0.551, P<0.01; r=0.738, P<0.01; r=0.685, P<0.01; r=0.497, P<0.01). |
|  | Yildirim[295] | 2021 | Case-control | 15 AS  9 HC | miR-145-5p | A significant association was found between miR-145-5p and BASDAI (p = 0.04941). |
|  | Zhang[296] | 2018 | Case-control | 50 AS  25 HC | miRNA16a | MiR16a expression was positively correlated with IL-4/IL-10 or disease active index, and was negatively correlated with IFN-γ and TNF-α levels (p<0.05), but not with CRP or ESR. |
|  | Fotoh[278] | 2020 | cross-sectional study | 55 AS  55 HC | miRNA-125a | miRNA-125a was positively correlated with CRP, ESR, BASDAI, BASFI, and BASMI (p < 0.001) |
|  |  |  |  |  | miRNA-451a | miRNA-451a was negatively correlated with CRP, ESR, BASDAI, BASFI, and BASMI (p < 0:001) with positive correlation with vitamin D (r = 0:471 and p < 0.001). |
|  | RAJZLEROVÁ | 2020 | case study | 19 AS | miR-145 | Patients with a more significant decrease in miR-145 levels may show further significant improvement of disease activity after 12 months. |
|  | PrajzlerovaÂ[297] | 2017 | Case-control | 68 axSpA  29 HC | miR-29a-3p, miR-146a-5p or miR-222-3p, miR-625-3p | miR-29a-3p, miR-146a-5p or miR-222-3p with an established role in extracellular matrix formation and inflammation were associated with spinal changes and/or disease activity assessed by BASDAI in AS patients |
|  | Reyes-Loyola[298] | 2019 | cross-sectional study | 15 AS  13 HC | miR-16 | Notably, plasma miR-16 levels were inversely correlated with the ASDAS-CRP score (rho -0.64, -0.87 to -0.18; P = 0.011), the BASFI score (rho -0.78, -0.93 to -0.43; P = 0.001), and serum MMP-1 levels (rho -0.59, -0.85 to -0.09; P = 0.022). |
|  | Tan[282] | 2021 | Case-control | 44 AS  56 HC | miR-125a-5p, miR-155a | Our findings suggest the association of miR-125a-5p and miR-155a with disease activity in AS patients. C-reactive protein (CRP) levels were positively correlated with the expression of miR-125a-5p (rs = 0.438, p = 0.005) and miR-155a (rs = 0.414, p = 0.006), which indicates that miR-125a-5p and miR-155a can perhaps aggravate AS-induced inflammation. |
|  | Li[283] | 2017 | Case-control | 59 axSpA  39 HC | MiR-27a | MiR‐27a was negatively correlated with Ankylosing Spondylitis Disease Activity Score as well as C‐reactive protein in patients with nr‐axSpA (r = −0.51, P < 0.01 and r = −0.42, P = 0.034 respectively). |
| lncRNA | Lan[285] | 2018 | Case-control | 82 AS  32 HC | TUG1 | Pearson correlation analysis revealed that serum levels of TUG1 were negative correlated with serum levels of CRP in ankylosing spondylitis patients (r = -0.8091, R2 = 0.6431, p < 0.0001). |
|  | Zhong[286] | 2019 | Case-control | 80 AS  22 LBP  20 HC | LINC00311 | BASDAI, ASDAS 1–4, CRP and ESR were significantly and positively correlated with plasma levels of LINC00311 (R square > 0.65, all p values < 0.0001). |
|  | Han[299] | 2022 | Case-control | 60 AS  60 HC | lncRNA‐NEF | It was observed that lncRNA‐NEF expression in synovial fluid samples was significantly correlated with ASDAS 1–4, BASDAI, and the levels of ESR and CRP, showing positive linear correlations |
|  | Wang[287] | 2022 | Case-control | 20 AS  20 HC | linc00304  linc00926  MIAT | linc00304 expression was positively correlated with bath ankylosing spondylitis disease activity index (BASDAI), bath ankylosing spondylitis functional index (BASFI), erythrocyte sedimentation rate (ESR), and c-reactive protein (CRP). linc00926 expression was only positively correlated with ESR, whereas MIAT expression was positively correlated with BASFI, ESR, and CRP. |
| circRNA | Tang[289] | 2021 | Case-control | 60 AS  30 HC | hsa_circRNA_012732 | Correlation analysis results showed that hsa_circRNA_012732 was negatively correlated with Bath Ankylosing Spondylitis Disease Activity Index (BASDAI), high-sensitivity C-reactive protein (hsCRP), and globulin (GLOB) and positively correlated with lymphocyte count (LY), mean corpusular volume, and albumin (ALB), and hsa_circRNA_008961 was negatively correlated with platelet (PLT) count. |
|  | Luo[290] | 2020 | Case-control | 46 axSpA  46 SLE  25 HC | hsa_circ_0079787 | The peripheral blood levels of hsa_circ_0079787 in patients with axSpA were negatively correlated with the Bath ankylosing Spondylitis disease activity index and positively correlated with the platelet count (PlT) and the lymphocyte-to-monocyte ratio. |

**Table: Studies addressing the value of** **non-coding RNA as a biomarker for predicting therapeutic effect of bDMARDs**

| Category | Study | Year | Design | Population | RNA | Result |
| --- | --- | --- | --- | --- | --- | --- |
| microRNA | Qian[291] | 2016 | Case-control | 80 AS  78 HC | miR-155 | the expression level of miR-155 is suggested to be associated with disease activity and the severity of thoracolumbar kyphosis secondary to AS. |
|  | Guo[300] | 2018 | Case-control | 219 AS | miR-132 | the expressions of MiR-132 and NAG-1 could serve as biological markers in the prediction of the therapeutic efficiency of NSAID treatment in AS patients. |
|  | Ciechomska[301] | 2018 | Case-control | 13 AS  12 HC | miR-5196 | miRNA-5196 which allow to predict and monitor anti-TNF-α response, would be of clinical value especially during the early phase of RA or AS development. |
|  | Lv[302] | 2014 | Case-control | 40 AS  50 HC | hsa-miR-126-3p, hsa-miR-29a | Moreover, expressions of hsa-miR-126-3p and hsa-miR-29a were dramatically upregulated after 12-weeks etanercept treatment. Fold changes were 2.20 and 3.18. |
| lncRNA | Lan[285] | 2018 | Case-control | 82 AS  32 HC | TUG1 | Patients in high expression group showed longer hospitalization time and higher rehospitalization rate. |
|  | Zhong[286] | 2019 | Case-control | 80 AS  22 LBP  20 HC | LINC00311 | Comparing to pre-treatment levels, plasma levels of LINC00311 were significantly reduced at 2 months after the initiation of treatment (p = 0.017). |
|  | Han[299] | 2022 | Case-control | 60 AS  60 HC | lncRNA‐NEF | The recurrence rate of AS was significantly lower in patients in the low lncRNA‐ NEF level group than in the high lncRNA‐NEF level group (hazard ratio = 2.266), suggesting that lncRNA‐NEF might serve as a prognostic biomarker for AS. |

**Table: Studies addressing the value of** **non-coding RNA as a biomarker for predicting radiological progression**

| Category | Study | Year | Design | Population | RNA | Result |
| --- | --- | --- | --- | --- | --- | --- |
| microRNA | Zou[303] | 2019 | Case-control | 69 AS  69 HC | miR-21 | serum miR-21 expressions were related to structural damage and radiological progression in AS, indicating that miR-21 may act as a switch between inflammation and new bone information and regulate different signal ways between lesioned enthesis and trabecular bone |
|  | Fotoh[278] | 2020 | cross-sectional study | 55 AS  55 HC | miRNA-451a, miRNA-125a | miRNA-125a had an AUC of 0.775, and the optimal cutoff point was 5.637 with sensitivity and specificity of 65.62% and 78.265, respectively. miRNA-451a had an AUC of 0.692, and the optimal cutoff point was 2.979 with sensitivity and specificity of 62.50% and 73.915%, respectively |
|  | PrajzlerovaÂ[297] | 2017 | Case-control | 68 axSpA  29 HC | miR-29a-3p, miR-146a-5p or miR-222-3p, miR-625-3p | miR-625-3p reflecting disease activity in AS with spinal involvement. |
| circRNA | Zou[304] | 2023 | Case-control | 3 AS  3 FNF | hsa_circ_0067103, hsa_circ_0004496, hsa_circ_0002649, ACTG1,  hsa_circ_0020273, hsa_circ_0005699, and hsa_circ_0048764 | hsa_circ_0067103, hsa_circ_0004496, and hsa_circ_0002649, ACTG1 were significantly upregulated, while hsa_circ_0020273, hsa_circ_0005699, and hsa_circ_0048764 were markly downregulated in AS tissue than FNF controls. |

**Question 12: Inflammatory cytokines including IL-6, IL-17 and TNF-α**

We found 29 Cohort and 31 Cross-sectional studies addressing this question.

The evidence shows:

1.Meta-analysis showed that IL-6 levels were significantly higher in patients with axSpA compared with healthy controls.[305] Multiple studies confirmed that IL-6 is associated with CRP and ESR, exhibiting its capacity as an inflammatory biomarker. Furthermore, previous reports showed that IL-6 could be a potential predictor of mSASSS score changes.[306]

2.Studies confirmed that IL-17 is significantly elevated in axSpA patients, and it is correlated with enthesitis and peripheral arthritis. One study showed that baseline IL-17 level could predict therapeutic responses of TNFi.[307]

3.TNF-α has been reported to correlate with levels of other inflammatory biomarkers, such as ESR and IL-6. [306] Evidence is still lacked regarding its capacity of predicting radiographic progression or therapeutic responses.

The quality of evidence is LOW.

**Table: Evidence profile**

| Certainty assessment | | | | | | | Summary of findings | |
| --- | --- | --- | --- | --- | --- | --- | --- | --- |
| No of participants  (studies)  Follow-up | Risk of bias | Inconsistency | Indirectness | Imprecision | Publication bias | Overall certainty of evidence | Pooled Result (95%CI) | Brief Summary |
| Question 12: | | | | | | | | |
| 50 studies (29 cohort and 31 cross-sectional studies) | Not Serious | Not serious | Not serious | Not serious | Not serious | ⨁⨁◯◯  LOW | \ | IL-6, IL-17 and TNF-α are inflammatory biomarkers, and previous studies showed that they could predict radiographic progression or therapeutic responses. |

**Table: Studies addressing the value of IL-6 as a biomarker for diagnosis**

| Study | Year | Design | Population | Result |
| --- | --- | --- | --- | --- |
| Romero-Sanchez[308] | 2011 | cross-sectional | AS | A series of serum inflammatory biomarkers from AS patients,including IL-6,IL-17 and TNF-a,were not significantly increased compared to MLB pain and HC group. |

**Table: Studies addressing the value of IL-6 as a biomarker for indicating disease activity**

| Study | Year | Design | Population | Result |
| --- | --- | --- | --- | --- |
| Gratacós[309] | 1999 | Cohort | AS | serum IL-6 levels were significantly higher in patients with active AS than in those with inactive disease ( 8.3 ± 9 pg/ml versus 2.8 +/- 5 pg/ml ，P = 0.008) |
| Bal[306] | 2007 | cross-sectional | AS | The serum levels of IL-6 were higher in AS compared to HC (p<0.05).A positive correlation was found between IL-6 and CRP. |
| Elolemy[237] | 2013 | cross-sectional | AS | IL-6 levels was correlated significantly with BASDAI, ESR and CRP in AS patients. IL-6, correlated well with disease activity parameters may play pro-inflammatory role in the disease. |
| Sharma[310] | 2014 | cross-sectional | AS | Serum IL-6 and hs-CRP levels were significantly higher in cases as compared to healthy controls. Serum IL-6 levels correlated significantly with BASDAI and BASFI. |
| Przepiera-Będzak[311] | 2015 | cross-sectional | SpA | Serum IL-6 levels were significantly higher in SpA patients than HC.There was no correlation between IL-6, VAS, BASDAI,and angiogenic cytokines in SpA patients |
| Li[312] | 2016 | cross-sectional | AS | IL-6 has been found elevated in the serum of AS patients.Exposure with IL-6 in ligament tissue induced fibroblast ossification. |
| Rabelo[313] | 2018 | cross-sectional | AS | The IL-6, TNF-α, IL-10 and IL-8 concentrations were significantly higher in AS.But only a significant correlation of mean IL-6 concentration with the BASMI was shown. |
| Park[238] | 2007 | cross-sectional | AS | IL-6 levels correlated well with BASDAI and CRP levels in the AS group at the baseline and its changes during fellowup also had significant positive correlations with them.IL-6 might be used as a marker for monitoring the disease activity of AS. |
| Park[232] | 2009 | cross-sectional | AS | IL-6 (11.1±7.7 vs 1.4 ±1.0 pg/ml, p < 0.01) were also significantly increased in PBMCs from patients with AS compared with HC.The IL-6 levels showed significant correlations with ESR, CRP levels and BASDAI. |
| Londono[223] | 2012 | cross-sectional | SpA | IL-17, IL-23, TNF-α, IL-6, IL-1α, and US-CRP levels were significantly higher in patients with SpA when compared to controls. An increase in serum levels of US-CRP, IL-6, IL-1α, and LBP was correlated with factors associated with clinical activity and poor prognosis in spondyloarthritis. |
| Mattey[314] | 2012 | Cohort | AS | Baseline IL-6 levels correlated significantly with baseline levels of CRP,but no correlation was found between IL-6 levels and the BASDAI, BASFI. |
| Taylan[315] | 2012 | cross-sectional | AS | The levels of IL-6 were similar between active and inactive patients. |
| Taylan[316] | 2012 | cross-sectional | AS | IL-6 and IL-17 were significantly increased in the AS patients group compared with healthy controls,but both of them were were similar between the groups of active and inactive,anti-TNF and conventional treatment. |
| Sveaas[317] | 2015 | cross-sectional | AS | No differences between the AS and HC groups were seen for IL-6 and IL-17a.No significant associations were seen between inflammatory markers(IL-6,IL-17a,sTNF-R1,sTNF-R2) and BASDAI and ASDAI after adjusting for personal characteristics. |
| Liu[318] | 2015 | cross-sectional | AS | BASDAI, BASFI and ASDAS scales showed high disease activity in the AS patients.The levels of plasma TNF-a, IL-6, CRP, and ESR were significantly higher in AS patients, compared with the levels in healthy controls. |
| He[319] | 2017 | Cohort | AS | Serum levels of IL6 was significantly higher in the AS patients than in the control subjects.CRP was highly associated with serum biomarker IL6. |
| Korkosz[320] | 2018 | cross-sectional | SpA | Levels of IL-6,IL-17A and TNFα were significantly elevated in axSpA patients’ sera compared to sera of healthy donors. |
| Falkenbach[321] | 1998 | Cohort | AS | IL-6 does not seem to be a reliable marker of current disease activity in AS, as it does not correlate with short-term changes in clinical parameters. |
| Falkenbach[322] | 2000 | Cohort | AS | Differences in parameters for disease activity were not related to the serum concentration of IL-6 in the 1-year followup. That means the serum concentration of IL-6 does not allow a prediction of disease progression in the subsequent year. |

**Table: Studies addressing the value of IL-6 as a biomarker for predicting therapeutic effect of bDMARDs**

| Study | Year | Design | Population | Result |
| --- | --- | --- | --- | --- |
| Dong[208] | 2019 | Cohort | AS | IL-6 level positively correlated with ESR, CRP, ASDAS-CRP, BASFI, and BASMI respectively.Low level of IL-6 and ESR at baseline predicted good clinical response of 3-month TNF inhibitor treatment. |
| Brandt[323] | 2000 | Cohort | AS | CRP,ESR,IL-6 lever decreased significantly at 4 weeks and 12 weeks treatment of infliximab,which showed that anti-TNF therapy was efficacious |
| Tarner[324] | 2009 | cross-sectional | AS | The AS patients showed a profound decrease in the levels of TNF-α, IL-1β, and IL-6 in response to treatment of mild whole-body hyperthermia. |
| Visvanathan[263] | 2008 | Cohort | AS | Significant correlations in the infliximab group were observed between early decreases in IL-6 and VEGF levels and increases in spinal BMD scores. These results suggest that changes in levels of IL-6, VEGF are associated with the changes in disease processes that occur with infliximab treatment in AS. |
| Pedersen[264] | 2010 | Cohort | SpA | After initiation of anti-TNF therapy, clinical responders decreased persistently in CRP, IL-6, VEGF, MMP-3, pain, and patient's global assessment.IL-6 may have potential value in monitoring disease activity and treatment response in patients with axial SpA treated with TNFα inhibitors. |
| Pedersen[325] | 2011 | Cohort | SpA | Patients with major improvement in ASDAS and BASDAI responders had larger percentage decreases in CRP and IL-6 after during anti-TNFα therapy. |
| Capkin[326] | 2012 | cross-sectional | AS | There was no significant correlation between IL-6 levels and BASDAI,ESR,CRP in the study.IL-6 was no significant difference among sub-groups established on the basis of medical treatments and disease activity (BASDAI ≤4 or>4). |
| Limón-Camacho[327] | 2012 | cross-sectional | AS | Serum levels of IL-6, IL-17A, TNF-α, and IL-8 were significantly higher in patients with AS compared to controls.These cytokine profiles of patients with AS taking TNF-α inhibitors were similar to those of healthy controls. |
| Wagner[245] | 2012 | Cohort | AS | IL-6 were associated with the achievement of ASAS 20 response at week 14 and also with changes from baseline to week 14 in both BASDAI and BASFI scores. |
| Xueyi[328] | 2013 | Cohort | AS | IL-6 was positively correlated with BASDAI score. Levels of IL-6 decreased significantly only in responders after anti-TNF-α therapy. |
| Schulz[329] | 2014 | Cohort | AS | The proinflammatory cytokine IL-6 tends to decrease during 12 weeks TNF-a therapy, but only reached statistical significance. |
| Inman[244] | 2016 | Cohort | AS | Strong correlations were observed between baseline IL-6 and baseline ASDAS.Baseline IL-6 and reductions at week 4 in IL-6 after golimumab-treat significantly correlated with week 14 ASspiMRI-a improvement. |
| Levitova[330] | 2016 | Cohort | axSpA | Disease activity improved after an intensive exercise programme.Serum levels of IL-6,IL-17 and TNF-a in all axSpA patients were not significantly changed after 6 months of exercise therapy. |
| Zhang[331] | 2021 | Cohort | AS | IL-6 (P = 0.004) was higher in celecoxib responders compared with non-responders.IL-6 (P = 0.019) independently predicted higher ASAS 20 response to celecoxib at W12. |
| Eggert[332] | 2007 | Cohort | AS | All AS patients showed a favourable response to infliximab therapy as assessed by BASDAI before and 12 weeks after therapy.The levels of IL-6 and CRP were reduced to those measured in the healthy controls following 2 weeks of therapy and remained at this level measured at the 12 weeks. |

**Table: Studies addressing the value of IL-6 as a biomarker for predicting radiological progression**

| Study | Year | Design | Population | Result |
| --- | --- | --- | --- | --- |
| Du[333] | 2022 | cross-sectional | AS | Serum levels of IL-6 increased gradually with sacroiliac arthritis progression.The serum levels of IL-6 may be an important indicator for disease severity evaluation of AS. |
| Korczowska[334] | 2011 | cross-sectional | AS | IL-6 which was significantly higher in AS patients with osteoporosis/ osteopenia than those without.inflammation might contribute to the accelerated bone loss in AS through stimulation of bone degradation. |
| Pedersen[325] | 2011 | Cohort | SpA | Compared to patients with no new syndesmophytes, patients with new syndesmophytes had larger percentage decreases in CRP and IL-6 levels.This supports the hypothesis of a relationship between the resolution of inflammation and new bone formation in patients with axial SpA treated with TNF inhibitors |
| Gonzalez-Lopez[229] | 2017 | cross-sectional | AS | No significant difference was observed in the serum IL-6 concentration between patients with and without syndesmophytes. |

**Table: Studies addressing the value of IL-17 as a biomarker for diagnosis**

| Study | Year | Design | Population | Result |
| --- | --- | --- | --- | --- |
| Yang[335] | 2018 | cross-sectional | AS | rs6693831 and rs1884444 in IL-23R gene and rs2275913 in IL-17A gene have genetic association with AS. |
| Aghaei[336] | 2020 | cross-sectional | AS | regarding the frequency distribution of copy numbers, less than 2 copies of IL17RA were significantly susceptible to AS |

**Table: Studies addressing the value of IL-17 as a biomarker for indicating disease activity**

| Study | Year | Design | Population | Result |
| --- | --- | --- | --- | --- |
| Taylan[316] | 2012 | cross-sectional | AS | BASFI, BASDAI, BASMI, disease duration, CRP, and being on anti-TNFa treatment did not correlate with IL-17 concentrations. |
| Chen[337] | 2012 | cross-sectional | AS | Serum IL-17 and IL-23 levels were significantly higher in AS patients than in healthy controls and the levels correlate to disease activity measured by BASDAI scores. |
| Londono[223] | 2012 | cross-sectional | SpA | IL-17, IL-23, TNF-α, IL-6, IL-1α, and US-CRP levels were significantly higher in patients with SpA when compared to controls. An increase in serum levels of US-CRP, IL-6, IL-1α, and LBP was correlated with factors associated with clinical activity and poor prognosis in spondyloarthritis. |
| Mei[338] | 2011 | cross-sectional | AS | Serum IL-17 and serum IL-23 levels were significantly increased in AS patients.No associations of serum IL-17 and IL-23 levels with CRP or ESR were found. |
| Sveaas[317] | 2015 | cross-sectional | AS | No differences between the AS and HC groups were seen for IL-6 and IL-17a.But no significant associations were seen between inflammatory markers(IL-6,IL-17a,sTNF-R1,sTNF-R2) and BASDAI and ASDAI. |
| Wendling[339] | 2008 | cross-sectional | AS | Serum IL-17 levels were significantly higher in AS patients than in controls,but it was not correlated with any of the disease related clinical variables (BASDAI, BASFI, and BAS-G) or laboratory variables (ESR, CRP, and IgA). |
| Wielińska[340] | 2021 | cross-sectional | AS | IL-17 polymorphisms have influence on AS severity and may be biomarkers of response to anti-TNF drugs. |
| Tan[341] | 2022 | cross-sectional | AS | The presence of dysregulated Th17/Th1 cell balance is closely related to AS disease activity.The static group was observed a higher IL-35 level and a lower IL-17 than the active group. |
| Jansen[342] | 2015 | cross-sectional | AS | These results demonstrate that the frequency of IL-17-producing CD4+ T cells is enhanced in the early stages of disease. |

**Table: Studies addressing the value of IL-17 as a biomarker for predicting therapeutic effect of bDMARDs**

| Study | Year | Design | Population | Result |
| --- | --- | --- | --- | --- |
| Xueyi[328] | 2013 | Cohort | AS | Frequencies of Th17,levels of IL-17, and IL-23 were positively correlated with BASDAI and BASFIscore. Serum levels of Th17-related cytokines, including IL-17 and IL-23, were significantly decreased in responders after anti-TNF-α therapy, ,but they were significantly increased in nonresponders |
| Perpétuo[343] | 2015 | Cohort | 13 | Circulating levels ofIL-17A, IL-23 and TGF-β were decreased after TNFi treatment when compared to baseline. |
| Zhang[331] | 2021 | Cohort | AS | IL-17A (P = 0.007) levels was higher in celecoxib responders compared with non-responders.Univariate logistic regression analysis revealed that IL-17A could predict higher ASAS 20 response at W12 celecoxib treatment. |
| Limón-Camacho[327] | 2012 | cross-sectional | AS | Serum IL-17A was significantly higher in patients with AS compared to controls.After taking TNF-α inhibitors,Th17 cell subsets and IL-17A profiles of patients with AS were similar to those of healthy controls. |
| Milanez[344] | 2016 | cross-sectional | AS | After 24 months of TNF blockade, active-AS responders(ASDAS-CRP <2.1) still had higher plasma levels of IL-17A compared with nonresponders (ASDAS-CRP ≥2.1) . |
| Levitova[330] | 2016 | Cohort | axSpA | Disease activity improved after an intensive exercise programme.Serum levels of IL-6,IL-17 and TNF-a in all axSpA patients were not significantly changed after 6 months of exercise therapy. |

**Table: Studies addressing the value of TNF-α as a biomarker for indicating disease activity**

| Study | Year | Design | Population | Result |
| --- | --- | --- | --- | --- |
| Bal[306] | 2007 | cross-sectional | AS | The serum levels of TNF-a were higher in AS compared to HC (p<0.05).A positive correlation was found between TNF-a and ESR. |
| Capkin[326] | 2012 | cross-sectional | AS | There was no significant correlation between TNF-a levels and BASDAI,ESR,CRP in the study. |
| Du[333] | 2022 | cross-sectional | AS | There was also a positive correlation between the serum IL-6 and TNF-α levels and the BASDAI, the progression of AS, and the CT imaging-based classification. The serum levels of IL-6 and TNF-α can function as important indicators for auxiliary diagnosis and disease activity evaluation of AS. |

**Table: Studies addressing the value of TNF-α as a biomarker for predicting therapeutic effect of bDMARDs**

| Study | Year | Design | Population | Result |
| --- | --- | --- | --- | --- |
| Xueyi[328] | 2013 | Cohort | AS | TNF-α were positively correlated with BASDAI and BASFIscore. After anti-TNF-α therapy, serum levels of TNF-α was shown to decrease significantly in both responders and non-responders,but responders decreased more obviously. |
| He[319] | 2017 | Cohort | AS | After etanercept treatment, the levels of TGF-β and TNF-α increased. |
| Wen[345] | 2017 | cross-sectional | AS | The levels of TNF-α was significantly low in the serum of patients with active AS. After NSAIDs treatment, the levels of TNF-α decreased. |
| Levitova[330] | 2016 | Cohort | axSpA | Disease activity improved after an intensive exercise programme.Serum levels of IL-6,IL-17 and TNF-a in all axSpA patients were not significantly changed after 6 months of exercise therapy. |
| Dong[346] | 2019 | Cohort | AS | At week 24, the TNF-α level of the patients signficantly decreased after anti-TNFα treatment,but was still higher than that of the healthy controls. |

**Table: Studies addressing the value of TNF-α as a biomarker for predicting radiological progression**

| Study | Year | Design | Population | Result |
| --- | --- | --- | --- | --- |
| Gonzalez-Lopez[229] | 2017 | cross-sectional | AS | SerumTNF-a concentration was significantly higher in patients than controls.No significant difference was observed in the serum TNF-a concentration between patients with and without syndesmophytes. |

**Question 13**: **Peripheral lymphocyte subsets**

We found 8 Cohort and 87 Cross-sectional studies addressing this question.

The evidence shows:

1.Flow cytometry is employed to identify the proportions of each lymphocyte subset in the serum in axSpA patients.

2.We conducted an extensive meta-analysis regarding the disequilibrium of lymphocyte subsets in peripheral blood in axSpA patients. Results revealed that the Th17 proportions in the peripheral blood is significantly higher than healthy controls (SMD=3.005, 95% CI 2.439-3.571).

3.Results also showed that CD4+CD25+CD127low/- Tregs, which possessed immunomodulatory capacities, are significantly down-regulated in axSpA (SMD=-0.71，95%CI -0.79, -0.64).

4.Th1/Th2 ratio is significantly unregulated.

The quality of evidence is MEDIUM.

**Table: Evidence profile**

| Certainty assessment | | | | | | | Summary of findings | |
| --- | --- | --- | --- | --- | --- | --- | --- | --- |
| No of participants  (studies)  Follow-up | Risk of bias | Inconsistency | Indirectness | Imprecision | Publication bias | Overall certainty of evidence | Pooled Result (95%CI) | Brief Summary |
| Question 13: | | | | | | | | |
| 95 studies (8 Cohort and 87 cross-sectional studies | Not Serious | Not serious | Not serious | Not serious | Not serious | ⨁⨁⨁◯  MEDIUM | \ | Meta-analysis revealed that the Th17 proportions in the peripheral blood is significantly higher than healthy controls, while CD4+CD25+CD127low/- Tregs are significantly down-regulated in axSpA (SMD=-0.71，95%CI -0.79, -0.64). Th1/Th2 ratio is significantly unregulated. |

**Table: Studies addressing the value of peripheral lymphocyte subsets as biomarkers of AS**

| Study | Year | Design | Population | Result |
| --- | --- | --- | --- | --- |
| An[347] | 2019 | cross-sectional | 73AS，85HC | CD4 Treg and CD8 Treg, especially CD8 Treg, are below normal levels in peripheral blood T lymphocytes of AS patients. Th17 cells are higher than normal, and the ratio of them is higher than normal. The increase in ratio of Th17/CD4 Treg involved in the occurrence of disease.  There is a negative correlation between CD4 Treg and BASDAI score, but a positive correlation between Th17/CD4 Treg and BASDAI score. |
| Appel[348] | 2011 | cross-sectional | 19 AS ，20 HC | CD4+FoxP3+ T cells (Treg) accumulates in inflamed joints.  The high frequency of CD4+FoxP3+ T cells (Treg) in pSpA might contribute to the spontaneous resolution and remitting course of arthritis in pSpA as compared to the more persistent joint inflammation in RA. |
| Bautista-Caro[349] | 2014 | cross-sectional | 25 AS ，50 HC | the absolute numbers of cTfh are decreased in AS/nb patients |
| Bidad[350] | 2013 | cross-sectional | 18 AS ，18 HC | Th17 cells plays a pathogenic role in AS. Th1 cells did not seem to contribute in the pathogenesis of this disease.  Percentages of IL-17+, IFN-γ+, FoxP3+, RORγt+, and T-bet+ CD4+T cells are not significantly different between patients with active or inactive disease |
| Brand[351] | 1997 | cross-sectional | 21 AS ，29 HC | CD4:CD8 cell ratio is higher in AS, but the difference did not reach statistical  significance. CD19- and CD8-positive cells were not significantly distinct compared to healthy controls. |
| Cai[352] | 2013 | cross-sectional | 40 AS ，20 HC | The proportion of CD4+CD25high regulatory T cells increases in positive-HLA-B27AS patients, which might contribute to the development of AS |
| Cai[353] | 2005 | cross-sectional | 30 AS ，20 HC | CD19+ B cells in AS patients are significantly increased compared with controls. (P < 0.05)  The cellular immune dysfunction in AS patients is mainly characterized by the increased expression of CD4+T cells . The expression of CD8+T cells are decreased. |
| Cao[354] | 2004 | cross-sectional | 10 AS ，29 HC | A comparison of peripheral blood frequencies of CD25brightCD4T cells(Treg) between patients and healthy subjects showed significantly lower levels in the rheumatic patients, indicating a selective recruitment of regulatory T cells in the inflamed joint. |
| Chen[355] | 2013 | cross-sectional | 61 AS ，36 HC | The ratio of CD4+CD25+FoxP3+T cell and the ratio of CD4+Th1 cell of AS group were significantly lower than that of the control group.  The imbalance of the Treg/Th17 and Th1/Th17 cell exist in AS patients and they are significantly related to disease activity. |
| Chen[356] | 2011 | cross-sectional | 23 AS ，25 HC | A higher percentage of CD4+ T cells was noted in AS patients than in healthy controls (P < 0.01), but a similar result was not observed with regard to CD4 + CD25 high + regulatory T cells, CD19+ B cells |
| Cheng[357] | 2007 | cross-sectional | 25 AS ，21 HC | There are no significant differences in the percentage of CD4+CD25high cells in peripheral blood between patients with active AS and controls.  PBMC from patients with active AS expressed reduced levels of FOXP3 mRNA which are correlated with C-reactive protein.  Treatment with etanercept or thalidomide increased CD4+CD25high cells and FOXP3 mRNA expression in the peripheral blood of AS patients |
| Dejaco[358] | 2010 | cross-sectional | 22 AS ，17 HC | Increased levels of CD3CD4CD28++− T-cells not only in the peripheral blood but also in SF in SpA patients. |
| Deng[359] | 2019 | cross-sectional | 49 AS ，100 HC | Compared with the control group, the percentage of Th1 cell subsets, the ratio of Th1/Th2 increased (P<0.05); the percentage of Th1 cell subsets, Th1/Th2 ratio in the stable group increased (P<0.05) |
| Deng[360] | 2018 | cross-sectional | 91 AS ，50 HC | Compared with the healthy control group, the CD3+CD4-CD8+T (T8 cell) , are increased in AS patients (P<0.05) , CD3+T (total T cells) , CD3+CD4+CD8-T cells (T4 cell) , T4/T8, have no statistical significance (P>0.05) .  There is no significant difference in T lymphocyte subsets and cytokines between HLA-B27 positive AS patients and HLA-B27 negative AS patients (P>0.05) . |
| Dong[361] | 2006 | cross-sectional | 30 AS ，30 HC | As compared with controls, CD3+ and CD8+ were significantly lower in patients; CD4-were significantly higher in patients (P<0.05). |
| Duan[362] | 2017 | cross-sectional | 21 AS ，16 HC | The percentage of Treg in AS group is lower than that of healthy group. The expression of PD-1 on CD8+ T cells and Tim-3 on CD4+ T cells is lower in the AS group. |
| Dulic[363] | 2017 | cohort | 7 AS ，10 HC | Decreased proportions of naïve CD4 and CD8 cells, increased frequencies of Th1 and Th17 cells and higher Th1/Th2 ratios in the long-term anti-TNF-treated patients than healthy controls, therapy-naïve and short-term anti-TNF-treated AS patients. |
| Fattahi[364] | 2018 | cross-sectional | 30 AS ，15 HC | Th17 cells is higher in AS patients than in normal controls, whereas baseline levels of Treg cells are not significantly different between AS patients and healthy controls.  The correlation analysis showed that frequencies of Th17 is positively correlated with Bath Ankylosing Spondylitis Disease Activity Index (BASDAI) and Bath Ankylosing Spondylitis Functional Index (BASFI) scores, whereas Treg cells are revealed to be negatively correlated with BASDAI and BASFI scores. |
| Forger[365] | 2009 | cohort | 15 AS ，18 HC | The longitudinal changes in the frequency of Treg cells did not differ between patients and healthy controls and therefore seemed to be a pregnancy-related phenomenon.  The relative mRNA expression of FoxP3 is lower in AS patients than in healthy controls. |
| Gao[366] | 2012 | cross-sectional | 40 AS ，37 HC | Th17 cells is higher in AS patients than in controls.  CD4+CD25+FoxP3+ Regulatory T Cells are lower in Patients With AS |
| Guo[367] | 2012 | cross-sectional | 98 AS ，76 HC | Percentage of CD4+ and CD4+/CD8+ ratio in AS group are lower, but percentage of CD8+ is higher than in healthy control group (P<0.05). |
| Hajialilo[368] | 2019 | cross-sectional | 24 AS ，35 HC | The frequency of Th17 is higher in AS patients |
| Han[369] | 2006 | cross-sectional | 69 AS ，50 HC | Compared with controls, the CD3+ T cells、CD3+CD4+ T cells and CD19+ B cells in AS patients were significantly increased(P<0.05)  The CD3+CD8+ T cells were significantly decreased(P<0.05) |
| He[370] | 2012 | cross-sectional | 32 AS ，50 HC | Compared with the control group,the absolute quantities of T lymphocyte, B lymphocyte and NK lymphocyte in AS group were significantly higher(P<0.01 or P<0.05) |
| Hu[371] | 2019 | cross-sectional | 60 AS ，40 HC | Compared with the control group, the ratio of CD8+T cells was significantly increased in patients with HLA-B27 positive spondylits (P<0.01), and the rate of CD4+T cells and NK (CD19+) cells decreased significantly(P<0.01) |
| Hu[372] | 2013 | cross-sectional | 32 AS ，30 HC | The percentage of Th1 cell in peripheral blood and ratio of Th1 / Th2 were higher in patients with AS than that of control group,there was a significant difference（P < 0.05）,the percentages of Th cell and Th2 cell were compared with the control group,there was no significant difference（P > 0.05）. |
| Huang[373] | 2009 | cross-sectional | 20 AS ，9 HC | The levels of CD4+T cells of patients were significantly higher than the normal control group （P<0.01）,the ratio between CD4+CD25+ Treg,CD4+CD25+CD127low/-Treg cells and CD4+T cells were significantly higher than the normal control group .（P<0.01） |
| Huang[374] | 1990 | cross-sectional | 9 AS ，9 HC | There was no significant difference in the percentage of PBLT4 and T8 positive cells and T4/T8 ratio between AS patients and normal controls |
| Ji[375] | 2014 | cohort | 20 AS ，20 HC | Prior to treatment, the ratio of CD4CD25CD127++low Tregs in AS patients was significantly lower compared to that in healthy controls (P<0.05) and it was significantly increased following TGT treatment (P<0.05) |
| Kenna[376] | 2012 | cross-sectional | 17 AS ，20 HC | The proportion of IL-23R-expressing T cells in the periphery was 2-fold higher in AS patients than in healthy controls, specifically driven by a 3-fold increase in IL-23R-positive γ/δ T cells in AS patients. |
| Kim[377] | 2012 | cross-sectional | 49 AS ，53 HC | percentages of peripheral blood natural killer T (NKT) cell were lower in patients than in controls |
| Klasen[378] | 2019 | cross-sectional | 14 AS ，5 HC | EP4 is significantly overexpressed in Th17 cells from patients with AS compared to Th17 cells from healthy controls.  Increased EP4 expression levels in Th17 cells from AS patients correlate with high disease activity |
| Li[379] | 2019 | cross-sectional | 64 AS ，60 HC | The Foxp3 relative expression was significantly lower than that of controls（ P < 0. 05）. The ratio of Th17 cells in AS group were significantly higher than those in control group（ P < 0. 05）.The ratio of Treg cells was significantly lower than that in control group（ P < 0. 05） |
| Li[380] | 2013 | cohort | 222 AS ，68 HC | Significantly higher baseline circulating Th17 is observed in active AS patients than in healthy controls.  Frequencies of Th17 is positively correlated with BASDAI score and BASFI score. Treg were found to be negatively correlated with BASDAI score. |
| Li[381] | 2009 | cross-sectional | 30 AS ，10 HC | Percentages of Th1 cells from intractable AS patients were higher than these of healthy volunteers. Percentage of Th2 cells were low.  Th1 cells had positive correlation with criterion（BASDAI,BASFI,BASMI, night pain,ESR and CRP），Th2 cells was negatively correlated with criterion. |
| Li[382] | 2008 | cross-sectional | 50 AS ，21 HC | The expression of CD8+CD28-T cells in peripheral blood of AS patients was significantly higher than that of normal people (P=0.020), and the expression of CD3+ and CD8+CD28+T cells in peripheral blood of AS patients was significantly lower than that of normal people (P=0.039)（P=0.038）. There was no significant difference in the expression of CD8+T cells between the two groups (P > 0.05). |
| Liao[383] | 2015 | cohort | 69 AS ，30 HC | The percentages of Tregs in PMBCs were significantly higher in AS patients than in healthy controls.  In AS patients who had poor disease functional index with higher levels of ESR and CRP were positively and significantly correlated with Tregs percentages in PMBCs. |
| Limon-Camacho[384] | 2012 | cross-sectional | 39 AS ，25 HC | The percentages of Th17 and Th1 cells in AS were higher than in healthy controls (p < 0.0001). Th17 and Th1 cell subsets in patients taking TNF-α inhibitors were lower than in those naive to such therapeutics and similar to healthy controls. |
| Lin[385] | 2008 | cohort | 66 AS ，30 HC | The CD4+T lymphocytes of AS patients were higher than those of healthy volunteers (P < 0.05), while the CD8+T lymphocytes were lower than those of healthy volunteers (P < 0.05). |
| Lin[386] | 2009 | cohort | 66 AS ，30 HC | Percentages of CD19(+) B-cells in active or peripheral joint involvement AS patients increased more obviously than those in stable or axial involvement alone AS patients (both P = 0.001), percentage of CD19(+)CD27high B-cells in AS patients with peripheral joint involvement was significantly higher than that in cases with axial involvement alone or healthy volunteers (P = 0.005 and 0.006);  The percentage of CD19(+) B-cells in AS patients was positively correlated with BASDAI scores, PGA scores |
| Liu[387] | 2017 | cross-sectional | 38 AS ，38 HC | The level of CD_4_^+^Th1 cell was lower than the control group（ P < 0. 05） |
| Liu[388] | 2016 | cross-sectional | 60 AS ，20 HC | Compared with normal control,BTLA and Treg expressing of AS patients were reduced significantly |
| Liu[389] | 2012 | cross-sectional | 60 AS ，30 HC | Compared with the healthy check-up,CD4+CD25+Treg expressing frequencies in AS patients were reduced significantly（P<0.01）Compared with pre-treatment,CD4+CD25+CD127-Treg and CD4+CD25+Treg expressing frequencies after treatment in Research group and the control group were significantly higher（P<0.01）.CD4+CD25+Treg expressing frequencies after treatment in research group had the trend of rising（P>0.05）.Compared with post-treatment in the control group,CD4+CD25+CD127-Treg expressing frequencies after treatment in Research group were significantly higher（P<0.05） |
| Liu[390] | 2010 | cross-sectional | 30 AS ，20 HC | The percentage of Th and Th2 cells in the peripheral blood was not significantly different in patients with AS than that of normal controls（P > 0.05）.The percentage of Th1 cells in the peripheral blood was significantly higher in patients of AS than that of normal controls（P < 0.01） |
| Long[391] | 2018 | cross-sectional | 65 AS ，20 HC | the percentages of cTfh cells and activated B cell subtypes are significantly increased and positively correlated with disease activity in patients with AS, and the percentage of cTfh cells is positively correlated with particular B cell subtypes. |
| Ma[392] | 2011 | cross-sectional | 36 AS ，32 HC | The percentage of CD3+CD8+ T lymphocytes in peripheral blood of patients was significantly lower than that of healthy controls（P<0.05）, CD4+/CD8+ ratio increased significantly（P<0.05）, CD3+ and CD3+CD4+T lymphocytes increased, but there was no significant difference (P > 0.05) |
| Ma[393] | 2011 | cross-sectional | 43 AS ，20 HC | The control group, the AS group total T cells (CD3 +), weak T cells (CD3 + CD8 + T cells) and total B cells (CD3 -CD19 +), total NK cells (CD3-CD16 + 56 +), activated lymphocytes (CD25 +) had significant difference |
| Ma[394] | 2004 | cross-sectional | 25 AS ，30 HC | The percentages of CD3+ and CD3+CD8+T cells were significantly lower than those in healthy control group. The percentage of CD8+HLA-DR+T cells was significantly lower than that of healthy control (P < 0.05), and CD4+HLA-DR+T was significantly higher than that of healthy control (P < 0.05). |
| Meng[395] | 2015 | cross-sectional | 42 AS ，20 HC | The proportions of helper Tc cell subsets with CD3+CD8+ receptor and Tc2 cell subsets with IL-4 cytokine secretion were higher in case group than in control group (P＜0.01).The ratio of Tc1 subgroup and Tc1/Tc2 with TNF-γ secretion was lower than that of control group (P＜0.01) |
| Mo[396] | 2019 | cross-sectional | 30 AS ，23 HC | The proportions of neutrophil absolute value, CRP, ESR, CD3+T lymphocytes, CD4+T lymphocytes, NK cells, γδT cells and MDSC in S patients were significantly higher than those in control group (P < 0.05), but there was no significant difference in the proportion of Treg. |
| Pishgahi[230] | 2020 | cross-sectional | 31 AS ，35 HC | In AS patients with MetS, higher Th17 and lower Treg frequency were observed. |
| Shan[397] | 2015 | cross-sectional | 20 AS ，10 HC | The frequency of peripheral blood FOXP3+CXCR5+CD4+TFR cells, CXCR5+CD4+TFH cells, the ratio of FOXP3+CXCR5+CD4+TFR/CXCR5+CD4+TFH cells in the AS patients were significantly higher than those in the healthy controls.  The frequency of FOXP3+CXCR5+CD4+TFR cells and the ratio of FOXP3+CXCR5+CD4+TFR/CXCR5+CD4+TFH cells significantly rose in those patients after standard treatment (P = 0.0006, P < 0.0001).  The frequency of TFR cells was negatively correlated with that of TFH cells. |
| Shen[398] | 2009 | cross-sectional | 10 AS ，16 HC | The percentages of IL-17-positive CD4+ T cells and IL-22-positive CD4+ T cells were increased in the PBMCs of both patients with AS |
| Suen[399] | 2008 | cross-sectional | 23 AS ，26 HC | No significant difference in Treg preponderance was observed between subjects with AS and healthy controls |
| Szalay[400] | 2012 | cohort | 13 AS ，9 HC | The overall prevalence of CD4+ cells within lymphocytes increased in AS, CD25 decreased.  In AS, Th1 and Th2 prevalence values increased by approximately 30 per cent,  Th17 prevalence increased, while Treg numbers were comparable to that in controls. |
| Szanto[401] | 2008 | cross-sectional | 42 AS ，52 HC | In the peripheral blood, the frequencies of CD4+ T helper and CD56+ NK cells were higher in AS compared to controls (p < 0.05).  The frequencies of Th0 and Tc0 cells were higher, while that of Tc1 cells was lowerin patients with AS versus controls (p < 0.05). |
| Thoen[402] | 1987 | cross-sectional | 31 AS ，15 HC | All the patient groups had normal proportions of T4+ and T8+ cells as well as normal T4/T8 ratios in peripheral blood.  In the synovial fluids the T4/T8 ratios were reduced in ankylosing spondylitis (p less than 0.05) |
| Toussirot[403] | 2009 | cross-sectional | 32 AS ，15 HC | No difference in CD3+ T cells was observed between SpA patients and healthy controls, while CD8+ T cells were found to be lower in SpA(P>0.05). |
| Wang[404] | 2020 | cross-sectional | 90 AS ，90 HC | The distribution ratio of Th17,ratio of Th17/Treg in peripheral blood of AS group were higher than those in control group,while the distribution ratio of Treg was lower than that in control group (p < 0.05).  AS patients with different Th17/Treg ratios had statistically significant differences in MRI sacroiliac joint lesion degree（P<0.05）.The moderate to severe proportion of high ratio group was higher than that in low ratio group（P<0.05）. |
| Wang[405] | 2018 | cross-sectional | 30 AS ，30 HC | The percentage of Th17 cells were significantly higher in the observation group than those in the control group（P<0.05）; the percentages of Th1 cells and Treg cells were significantly lower than those in the control group（P<0.05）. |
| Wang[406] | 2018 | cross-sectional | 26 AS ，26 HC | In AS patients, the frequency of CD4CD25Foxp3CD127 Treg cells was slightly increased compared to healthy controls, but the level of Foxp3 MFI in AS patient CD4CD25Foxp3CD127 Treg cells was significantly lower than that in healthy control CD4CD25Foxp3CD127 Treg cells. |
| Wang[407] | 2016 | Cross-sectional | 50 AS, 50 HC | AS patients had significantly lower Vδ1 T cell ratio in PBMC compared to controls (p<0.05), but their CD4 T cell ratio was significantly elevated (p<0.05). |
| Wang[408] | 2015 | cross-sectional | 78 AS ，30 HC | AS patients had significantly lower Vδ1 T cell ratio in PBMC compared to controls (p<0.05), but their CD4 T cell ratio was significantly elevated (p<0.05). |
| Wang[409] | 2015 | cross-sectional | 45 AS ，20 HC | Compared with the control group, the percentage of Treg cells in AS group and low-activity AS group had no significant difference（ P > 0. 05）,high-activity AS group peripheral blood Treg cells were significantly reduced（ P < 0. 01）.  The percentage of Th17 cells in AS group increased（ P <0. 01）,peripheral blood Th17 percentage of low-activity AS group and high-activity AS group were increased（ P < 0. 05）,and the ratios of Th17 / Treg in AS group, low-activity AS group and high-activity AS group were increased（ P < 0. 01）.  The ratio of Treg cells of high-activity AS group was significantly lower than that of low-activity AS group（ P < 0. 01）; the ratio of Th17 cell of high-activity AS group was significantly higher than that of low-activity AS group（ P < 0. 01）; the ratio of peripheral blood Th17 / Treg cell of high-activity AS group was significantly higher than that of low-activity AS group（ P< 0. 01）.  There were positive correlation of Bath ankylosing spondylitis disease activity index（ BASDI） with peripheral blood Th17 cell ratio and Th17 / Treg ratio of AS patients,negative correlation between BASDI and Treg cells ratio of AS patients（ P < 0. 05）. |
| Wang[410] | 2012 | cross-sectional | 60 AS ，44 HC | The percentage of Th1 and Th17 cells was found to be significantly higher in the ankylosing spondylitis groups (mild and severe) compared with the healthy individuals.  The Th1/Th2 and Th17/Treg ratios were significantly higher in patients with ankylosing spondylitis. |
| Wang[411] | 2008 | cross-sectional | 30 AS ，20 HC | The percentage of CD3+T,CD4+T,CD19+B cells,CD4+/CD8+ ratio and serum IgG,IgA,IgM,C3,C4 contents in AS group were significantly higher than those in control group (P<0.01 or P<0.05), and the percentage of CD16+56+NK cells was significantly lower than that of control group (P<0.01). The percentage of CD8+T cells was not significantly different from normal control group (P> 0.05) |
| Wei[412] | 2017 | cross-sectional | 131 AS ，127 HC | The levels of Th1/Th2 cytokines TNF-α, IL-17 and IL-10 in serum and joint fluid of AS patients were significantly higher than those in normal control group, and the levels of IL-4 were significantly lower than those in normal control group. |
| Wu[413] | 2014 | cross-sectional | 60 AS ，60 HC | The percentage of circulating CD4+CXCR5+ICOS+Tfh cells was increased inAS patients compared with HC, but the increase was not significant （P=0.137）. As AS patients were divided into active and inactive groups, the percentage of circulating CD4+CXCR5+ICOS+Tfh cells was significantly increased in active group compared with both inactive group and HC （P=0.002; P<0.001）  The percentage of circulating Tfh cells was significantly positively correlated with the score of BASDAI in AS patients （P=0.001） |
| Wu[414] | 2011 | cross-sectional | 51 AS ，49 HC | The frequencies of Treg and Fox-P3cells in AS-PBMCs decreased, while CCR4CCR6Th cells increased, compared with healthy donors.  The AS-BMSCs induced imbalance in the ratio of CCR4CCR6Th/Treg cells by reducing Treg/PBMCs and increasing CCR4CCR6Th/PBMCs, and also reduced Fox-P3cells when co-cultured with PBMCs. |
| Wu[415] | 2011 | cross-sectional | 24 AS ，30 HC | The expressions of CD19+ total B cells,CD19+CD38+ plasma blast B cells, BAFF and receptor BR3 in AS patients were significantly higher than those of healthy controls（P<0.05） |
| Xu[416] | 2019 | cross-sectional | 18 AS ，9 HC | Compared to healthy donors, the ratio of TH17/Treg was significantly higher in AS patients without NBF and lower in AS patient with NBF (both P < 0.01) in flow cytometry analysis (FCA).  TH17 significantly decreased after indirectly coculturing with Treg in FCA (P < 0.01). |
| Xu[417] | 2018 | cross-sectional | 69 AS ，22 HC | The ratio CD4+/CD8+ in AS patients was higher than that in healthy subjects（P<0.05）,the percentage of natural killer T cells was significant lower in AS patients than that in healthy subjects（P<0.05）. |
| Xu[418] | 2013 | cross-sectional | 24 AS ，22 HC | The percentage of Th1 cells and Treg cells were lower in AS activity group than in AS stable group and the healthy controls, whereas the percentage of Th17 cells was higher. The mRNA level of RORC was significantly higher in AS patients, and the levels of T-bet and FoxP3were significantly lower. The ratio of T-betmRNA/RORCmRNA and FoxP3mRNA/RORC mRNA changed significantly in AS activity group. The expression of T-bet mRNA in AS activity group was negatively correlated with RORC mRNA, and positively correlated with FoxP3mRNA, also positively correlated in T-bet mRNA, RORC mRNA, FoxP3mRNA and ASDAS. |
| Xu[419] | 2011 | cross-sectional | 78 AS ，50 HC | The percentage of CD3+, CD4+T lymphocytes and the ratio of CD4+/CD8+ in AS patients were significantly higher than those in healthy group, the difference was statistically significant (P < 0.01). There are imbalance of Th1/Th2 and Tc1/Tc2 in AS patients. |
| Xue[420] | 2015 | cross-sectional | 38 AS ，30 HC | The percentage of CD19+CD24hiCD38hiBreg in AS patients was lower than that in healthy control.  Among AS patients, higher proportion of CD19+CD24hiCD38hiBreg was observed in active patients compared with inactive patients.  The frequency of CD19+CD24hiCD27+Breg did not show significantly different between AS patients and control, the proportion of CD19+CD24hiCD27+Breg in active AS patients was lower than that in control or inactive cases.  CD19+CD24hiCD38hiBreg ratio was significantly and positively correlated with the levels of Foxp3 mRNA（ P < 0. 05）,and negatively related to TH17,RORγt mRNA, BASDAI and ASDASCRP score.  In AS group, the frequency of CD19+CD24hiCD27+Breg was positively correlated with Foxp3 mRNA（P < 0. 05）,and negatively correlated to ASDAS-CRP. No significant correlations were observed between the level of CD19+CD24hi CD27+Breg and TH17, Treg, RORγt mRNA and BASDAI. |
| Xue[421] | 2008 | cross-sectional | 89 AS ，42 HC | The CD3+ CD4+T cells and CD4+/CD8+ in AS patients significantly increased comparing with controls（P<0.05）, the CD3+ CD8+T cells significantly decreased（P<0.05）. |
| Yang[422] | 2020 | cohort | 67 AS ，50 HC | In AS patients, Th1 cells, Tfh1 cells and Tregs were lower, but Th17 cells, Tfh17 cells, and Tc cells were higher. The proportions of total B cells and class-switched B cells were increased (P < 0.05), but non-switched B cells, plasma cells, memory B cells, and immature Bregs (regulatory B cells) were lower.  After Anbainuo therapy, Tregs had increased, and the increase in Tregs was positively correlated with the decrease in CRP. |
| Yang[423] | 2018 | cross-sectional | 30 AS ，30 HC | The percentage of peripheral blood CD3+CD56+NKT cells in AS group was lower than that in control group (P < 0.05).  The expression level of CD3+CD56+NKT in AS patients was negatively correlated with ESR, hs-CRP, BASMI and BASFI (P < 0.05). |
| Yang[424] | 2017 | cross-sectional | 40 AS ，40 HC | The percentage of circulating CD4+CD25+T and CD8+CD122+T cells were significantly increased in AS patients compared with HC（P=0.001）,and the circulating CD4+CD25+T cells of AS patients with long disease duration was especially higher than that of healthy controls.  The cells count of the CD8+CD28-T cell was positively associated with the level of inflammatory level（CRP）and the score of BASFI in AS patients, |
| Yang[425] | 2016 | cross-sectional | 38 AS ，31 HC | Compared with the healthy controls, the proportion of Teff in peripheral blood of active AS patients increased significantly, while the proportion of Treg had no significant difference. |
| Yang[426] | 2007 | cross-sectional | 60 AS ，30 HC | As compared with controls, the CD3+ T cells,CD3+CD4+ T cells, and CD19+ B cells in AS patients were significant increased（P<0.05）,but the CD3+ CD8+cells were decreased（P<0.05）. |
| Ye[427] | 2013 | cross-sectional | 21 AS ，27 HC | Compared with normal controls, Foxp3+ cells in AS patients contained a higher proportion of non-Treg (P < 0.05) and a lower proportion of naïve Treg (P < 0.05). In AS patients, the frequencies of effector and naïve Treg were decreased (P < 0.05). |
| Zhang[428] | 2019 | cross-sectional | 60 AS ，30 HC | Th1 and Th17 was significantly higher in AS than healthy controls. The expression level of Th1 and Th17 cells in AS was positively correlated with VAS and BASDAI. |
| Zhang[429] | 2019 | cross-sectional | 39 AS ，41 HC | Compared with the normal control group, the absolute cell count of peripheral blood lymphocyte subsets CD3+, CD3+CD4+, CD3+CD8+, CD16+CD56+ decreased in AS group（P<0.05）, the percentage of CD3+CD4+ and CD3-CD19+ cells increased（P<0.05）, the percentage of CD3+CD8+ and CD16+CD56+ cells decreased（P<0.05）. The percentage of CD3+CD4+ cells was positively correlated with BASDAI score（P<0.05）; The absolute count and percentage of CD3+CD8+ cells were negatively correlated with BASDAI score（P<0.05）; The absolute count and percentage of CD16+CD56+ cells were negatively correlated with BASDAI score（P<0.05）. |
| Zhang[430] | 2014 | cross-sectional | 60 AS ，60 HC | The levels of CD4 + Th1 and CD4 + CD25 + Treg cell of AS group were lower than control group(P < 0. 05).  The levels of CD4 + Th17 of severe group were higher than stable disease group and moderate disease activity group. and the levels of CD4 + Th1, CD4 + CD25 + Treg cells of severe group were lower than stable disease group and moderate disease activity group (P < 0. 05). |
| Zhang[431] | 2014 | cohort | 10 AS ，10 HC | The proportion of Th17/Treg in peripheral blood of patients with ankylosing spondylitis was significantly increased |
| Zhang[432] | 2012 | cross-sectional | 32 AS ，20 HC | Th17 cells were significantly elevated in AS patients compared with OA patients and healthy controls. The percentages of Th17 cells is not correlated positively with disease activity in AS patients. |
| Zhang[433] | 2008 | cross-sectional | 78 AS ，50 HC | The content and function of CD4+ Treg in peripheral blood of AS patients were lower than those of healthy volunteers (P<0.05) |
| Zhao[434] | 2013 | cross-sectional | 21 AS ，20 HC | The percentage of Th17 cells in peripheral blood of AS patients was significantly higher than that of healthy control group (P < 0.05), and Th17 cells in peripheral blood of AS patients in active group was also significantly higher than that in stable group (P < 0.05), while the percentage of peripheral blood regulatory T cells in active group was significantly lower than that in healthy control group (P < 0.05). Th17/Treg cell ratio in AS activity group was significantly higher than that in healthy control group and AS stable group (P < 0.05). |
| Zhao[435] | 2011 | Cross-sectional | 14AS, 18 HC | The number of peripheral blood CD4+CD25highCD127low/- Treg cells in AS patients was found to be significantly lower than in healthy controls and was inversely correlated with serum IgA levels. There was no significant correlation between CD4+CD25highCD127low/- Treg cell numbers and BASDAI scores. |
| Zhao[436] | 2009 | cross-sectional | 30 AS ，30 HC | The number of peripheral blood CD4(+)CD25(high)CD127(low/-) T(reg) cells in AS patients was found to be significantly lower than in healthy controls and was inversely correlated with serum IgA levels.  There was no significant correlation between CD4(+)CD25(high)CD127(low/-) T(reg) cell numbers and BASDAI scores. |
| Zhong[437] | 2014 | cross-sectional | 78 AS ，30 HC | The level of CD4+ and the ratio of CD4+/CD8+ in AS patients were significantly lower than those in normal control group (P < 0.01). CD8+ was significantly higher than normal control group (P < 0.01).  The level of CD4+ in peripheral blood of patients with AS was negatively correlated with BASMI (P < 0.01).CD8+ was positively correlated with BASFI and BASMI (P < 0.05). The ratio of CD4+/CD8+ was negatively correlated with BASFI and BASMI (P < 0.05). |
| Zhu[438] | 2017 | cross-sectional | 42 AS ，42 HC | Compared with healthy controls, CD4+ cells were predominant in peripheral blood T cells of AS patients, and the ratio of effector T cells to naive T cells was increased. The proportion of Th1 to Tfh was significantly higher than that of healthy control group, and the proportion of Treg cells was slightly higher than that of healthy control group. |
| Zhu[439] | 2016 | cross-sectional | 30 AS ，30 HC | The number of NK cells in AS patients was lower than that in control group (P < 0.05). The level of NK cells in peripheral blood of AS patients was negatively correlated with BASFI and BASMI (P < 0.05). |
| Zhu[440] | 2000 | cross-sectional | 14 AS ，7 HC | The number of Th1 or Th2 cells in AS patients was negatively correlated with markers of inflammatory activity. |

**Figure: Forest Plot of Proportions of major lymphocyte subsets in the peripheral blood of AS patients
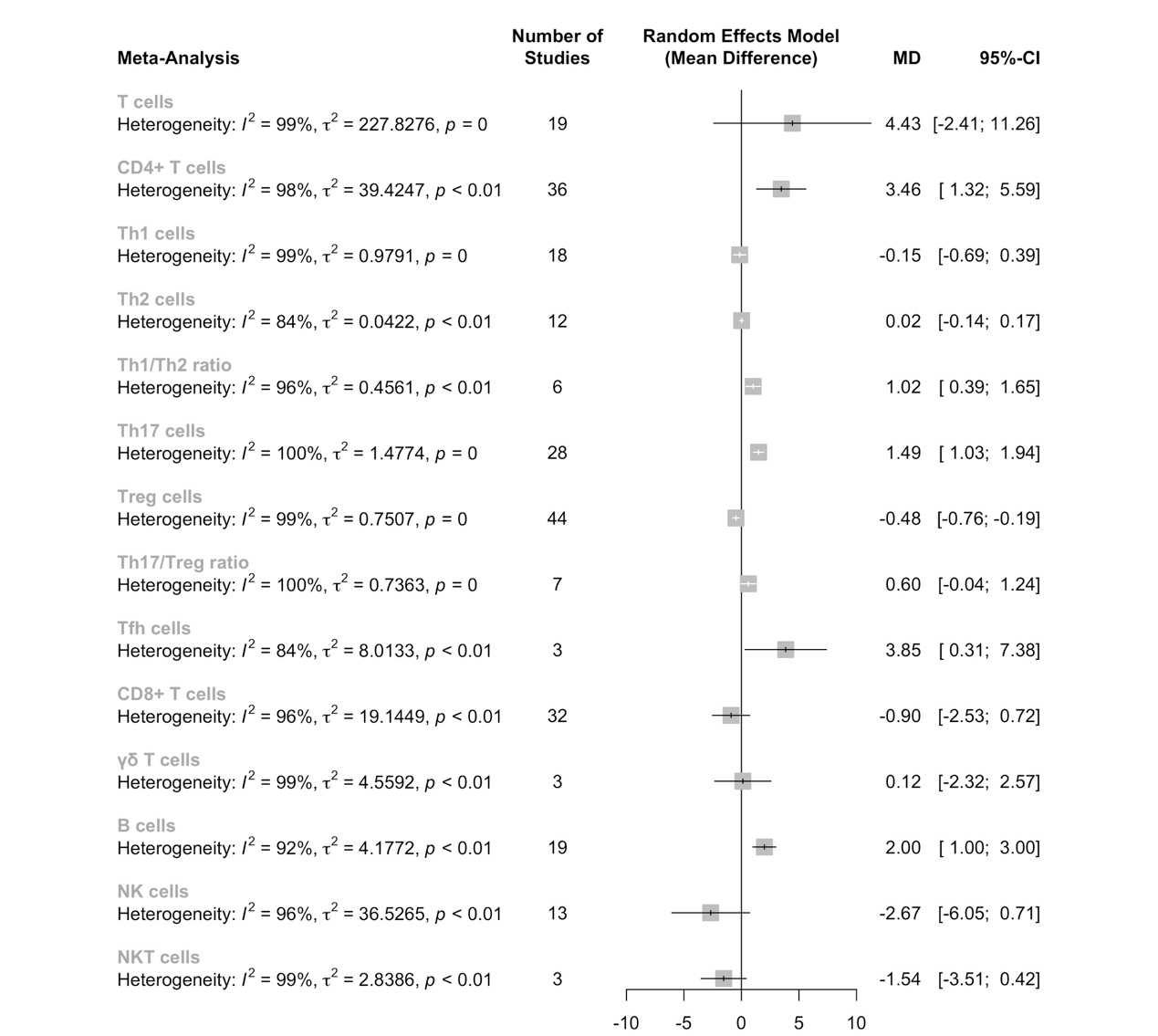
**

**Figure: Forest Plot of Proportions of Th17 cells among PBMCs, T cells and CD4+ cells
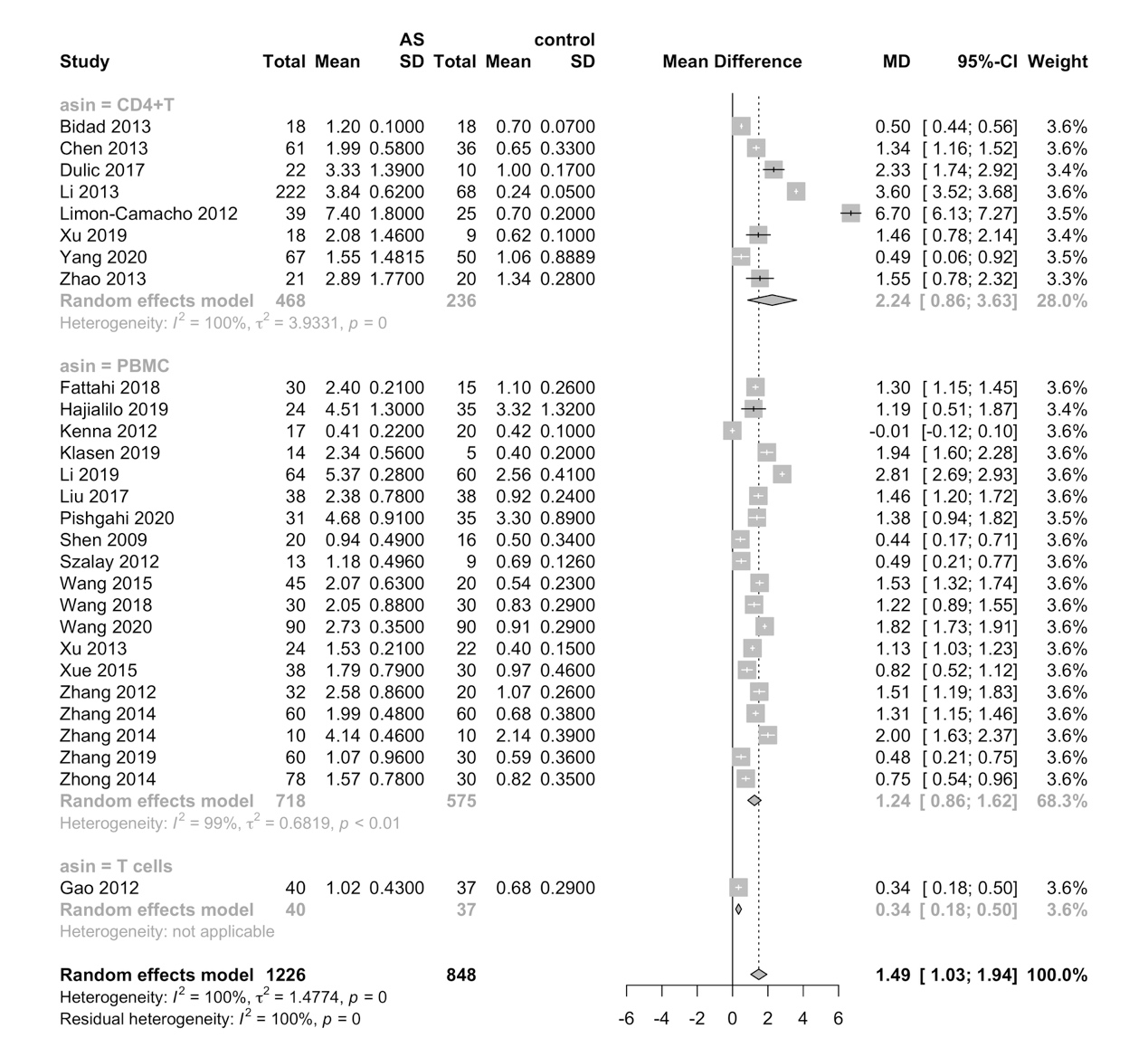
**

**
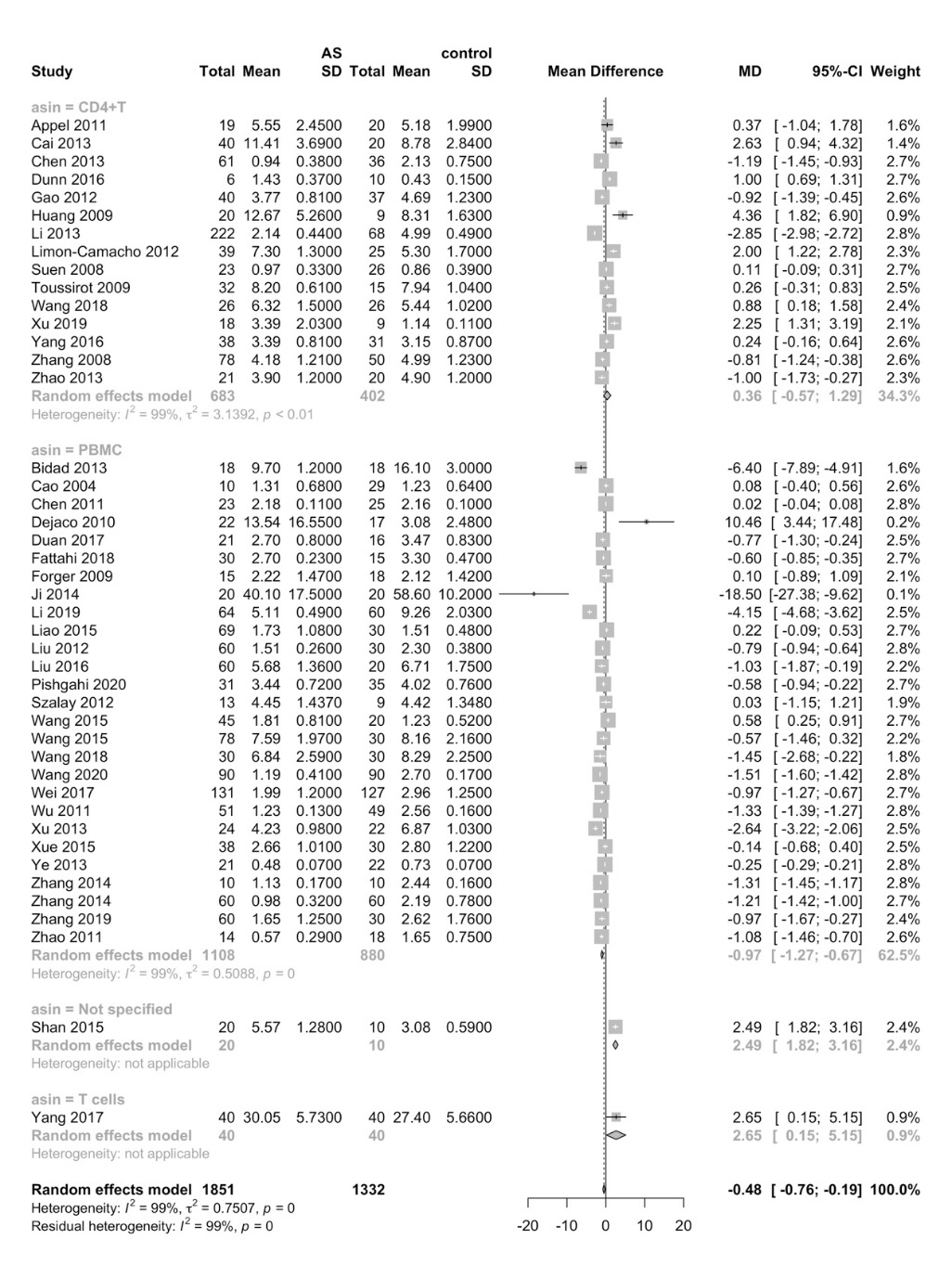
Figure: Forest Plot of Proportions of Tregs among PBMCs, T cells and CD4+ cells**

**Figure: Forest Plot of Proportions of Th1 cells among PBMCs and CD4+ cells
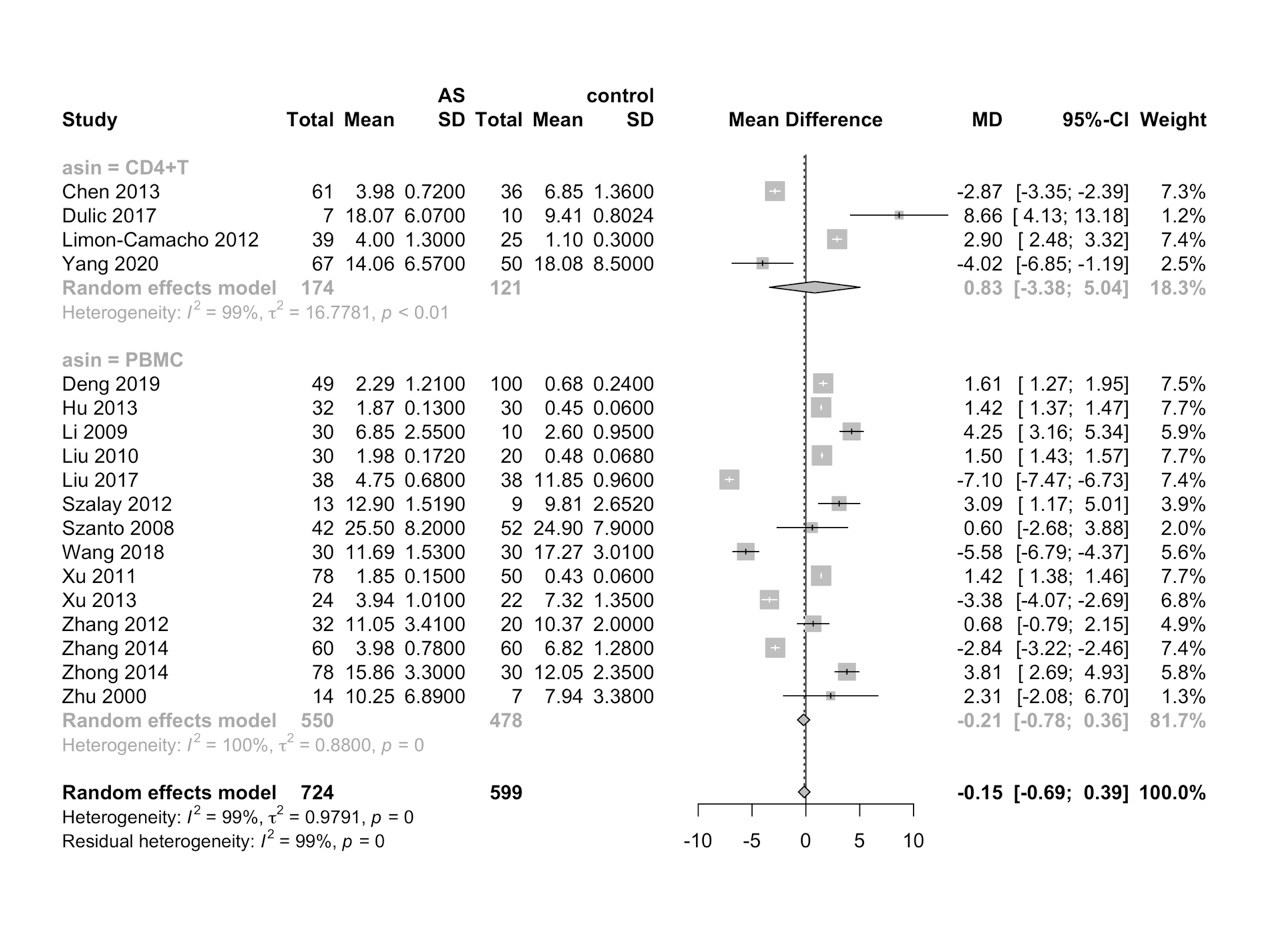
**

**Figure: Forest Plot of Proportions of Th2 cells among PBMCs and CD4+ cells
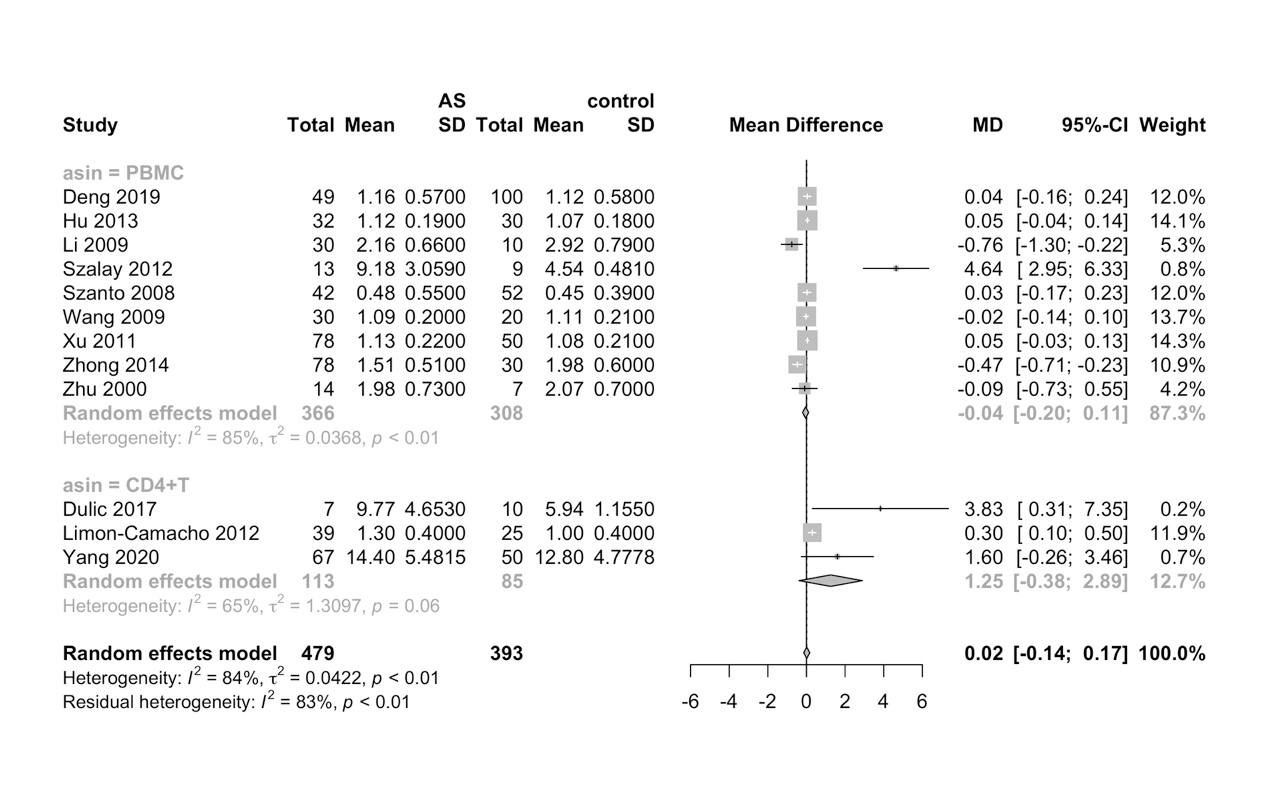
**

**Question 14**: **Bone turnover markers**

We found 14 Cohort and 45 cross-sectional studies addressing this question.

The evidence shows:

1.Axial spondyloarthritis is characterized by the disequilibrium between bone resorption and bone formation. Both osteoporosis and new bone formation are prominent features in axial spondyloarthritis. Studies have revealed that axSpA patients are at significant risks of osteoporosis.[441]

2.Bone resorption biomarkers include CTX-I, PYD and DPD. Our meta-analysis revealed that the both CTX-I level in the serum and DPD/creatinine ratio in the urine in axSpA patients are significantly higher than the healthy controls, indicating excessive bone resorption.

3.Bone formation biomarkers include PINP, BALP and osteocalcin. Our meta-analysis failed to identify an upregulation or downregulation among any of these biomarkers, which could be attributed to the high heterogeneity of the studies.

The quality of evidence is LOW.

**Table: Evidence profile**

| Certainty assessment | | | | | | | Summary of findings | |
| --- | --- | --- | --- | --- | --- | --- | --- | --- |
| No of participants  (studies)  Follow-up | Risk of bias | Inconsistency | Indirectness | Imprecision | Publication bias | Overall certainty of evidence | Pooled Result (95%CI) | Brief Summary |
| Question 14: | | | | | | | | |
| 59 studies (14 Cohort and 45 cross-sectional studies) | Serious | Not serious | Not serious | Not serious | Not serious | ⨁⨁◯◯  LOW | \ | Our meta-analysis revealed that the both CTX-I level in the serum and DPD/creatinine ratio in the urine in axSpA patients are significantly higher than the healthy controls, while it failed to identify an upregulation or downregulation among PINP, BALP and osteocalcin. |

**Table: Studies addressing the value of bone turnover markers as biomarkers for indicating disease activity**

| Study | Year | Design | Population | Result |
| --- | --- | --- | --- | --- |
| Marhoffer[442] | 1995 | cross-sectional | AS | Serum concentrations of osteocalcin and Skeletal ALP isoenzyme fraction did not differ significantly from those in healthy controls. In the AS patient group ,there was a positive correlation for urinary excretion of PYR and inflammatory disease activity (ESR) (r = 0-6, p < 0-0001) and CRP (r= 0 3, p < 0-02) . |
| Toussirot[443] | 1999 | cross-sectional | AS | A positive correlation was found between ESR and (f-Pyr+f-Dpyr) (r=0.42, P=0.018) and between ESR and f-Dpyr (r=0.49; P=0.005).Patients with raised ESR (>15 mm/h; mean:32.1±14.9 mm/h) had significantly higher f-Dpyr urinary levels than the other patients (P=0.009) .Bone impairment was more obvious in patients with raised ESR, suggesting a role for the inflammatory mediators in this bone loss. |
| Yilmaz[444] | 2000 | cross-sectional | AS | Both resorption markers were significantly higher in patients with AS (Dpyd,12.5 + 1.4; Pyd: 27.5 + 2.9) than in the control group (Dpyd: 5.7 + 0.4; Pyd: 10.5 + 1.7; p <0.001, p <0.01, respectively).Urinary Dpyd levels were affected by duration of disease, gender and ESR values of the patients whereas urinary Pyd levels were increased only in patients with an ESR>20 mm/h. Serum calcium,PTH,ALP, BALP and BGP levels of the patients and the control group were not statistically different (p >0.05). |
| Lange[445] | 2005 | cross-sectional | AS | Serum levels of 1.25 D3 and PTH were negatively correlated to disease activity (p<0.01).The excretion of urinary pyridinium cross-links was positively correlated to disease activity.The findings propose a close association of BMD, bone metabolism and inflammatory activity in AS. |
| Muntean[446] | 2011 | cross-sectional | AS | No correlation was found between OC or CTX levels and CRP levels (r=−0.03, p=0.84 and r=0.09, p=0.55, respectively) or BASDAI score (r=0.23, p=0.13 and r=−0.6, p= 0.70, respectively).But patients with active and severe AS tend to have increased levels of CTX. |
| Taylan[447] | 2012 | cross-sectional | AS | OPG showed correlations with BASDAI≥ 4 (367 vs 274pg/mL,p < 0.05; r = 0.3).Compared with conventional treatment,a decreased OPG and increased DKK-1,BAP and sRANKL/OPG were showed in patients on anti-TNF therapy(274 vs 384,p=0.04;108 vs 87,33 vs 28,1.28 vs 0.73,p=0.03) . |
| Korkosz[448] | 2013 | cross-sectional | AS | Sclerostin and OPG was significantly higher in AS patients with high disease activity in comparison to healthy subjects,but a negative correlation between sclerostin in high disease activity group (R = −0.28, P = 0.048). |
| Almodóvar[449] | 2014 | Cohort | SpA | Serum MMP-3 correlated significantly with swollen joint count (r=0.515, p=0.01) . Serum CTX-I showed a significant (p=0.01) weak correlation with swollen joint count (r=0.386) and serum CRP (r=0.361). |
| Klingberg[450] | 2014 | cross-sectional | AS | Serum levels of Wnt-3a were significantly elevated in the patients with AS and associated with higher BASMI and mSASSS. |
| Nocturne[451] | 2015 | cross-sectional | SpA | Mean DKK-1 serum levels were higher in axial SpA patients than controls (30.03± 15.5 vs. 11.6± 4.2 pmol/L; p<0.0001). DKK-1 serum levels were associated with male gender (p = 0.03), CRP level (p = 0.006), SOST serum level (p = 0.002) and presence of sacroiliitis on radiography (p = 0.05). |
| Wang[452] | 2015 | cross-sectional | AS | β-CTX levels were higher in the AS patients than in the controls (P <0.05).β-CTX were significantly higher in the group with active AS (P<0.05). |
| Zhang[453] | 2015 | cross-sectional | AS | The serum 25(OH)D in AS was significantly lower than controls (57.92± 24.42 vs 91.24± 42.02 nmol/L,p<0.01). Serum ICTP in AS was significantly higher than controls (5.72± 3.88 vs 3.69± 1.26 ug/L,p<0.01). 25(OH)D and CRP contributed independently and significantly to the serum ICTP level ( |
| Guła[454] | 2018 | cross-sectional | SpA | 25(OH)D was 24.9 ng/ml (SD 12.49) and was comparable in axSpA vs. perSpA (24 [SD 12.7] vs. 26.5 [SD 12.3], p = 0.45).There is a positive correlation between level of vitamin D and ESR in axSpA(p=0.004) |
| Descamps[455] | 2021 | Cohort | SpA | Serum BMP-7 was increased in active disease, but the increase was low with TNFi use.Serum DKK-1 decreased over 5 years with a median decrease of 4.7%(p=0.09) |
| Speden[456] | 2002 | cross-sectional | AS | Women with AS had significantly lower BALP [mean 15.0 (SD 4.8) vs 17.3 (6.2); p = 0.03, t test] and OC [mean 7.5 (SD 2.9) vs 9.6 (4.3); p = 0.02, t test] than controls.D-PYR, but not OC or BALP, correlated with CRP levels (r = 0.45, p = 0.01). |
| Torres[261] | 2019 | cross-sectional | AS | The male AS patients had higher serum levels of CTX1 and osteocalcin compared with the healthy male controls.ESR, CRP,andWBC were all positively correlated with serum levels of HGF, MMP-3, and VEGF.SerumCTX-1 and osteocalcin were not associated with any parameters reflecting disease activity. |
| Franck[457] | 2004 | cross-sectional | AS | OPG serum levels were significantly lower in patients with AS compared to controls (1.84± 1.15 vs 3.54± 2.18 pmol/l, p < 0.001). Patients with AS and osteoporosis had higher biochemical markers of bone resorption and inflammatory activity. |
| Grisar[458] | 2002 | cross-sectional | AS | the biochemical markers of bone resorption were significantly increased in patients with AS compared to controls. No significant correlation between OPG and markers of inflammation was observed. |
| Acebes[459] | 1999 | cross-sectional | AS | We found a decrease of bone mass and an increase in TPyr, FPyr, TDpyr, FDpyr, NTX and BSP in AS, but no significant differences were found in PICP, PINP and CTX. |
| Huang[460] | 2016 | cross-sectional | AS | The levels of DKK-1 was significantly higher in AS(1914.5±407.8 pg/mL)than in healthy controls (1729.1±352.9 pg/mL) (p<0.05).Correlation between ALP and bASDAI, BASFI, and ASDAI nearly achieved significance with p values of 0.055, 0.072, 0.062, respectively. |
| Borman[200] | 2001 | cross-sectional | AS | AS patients are at risk for developing osteoporosis when they have active and severe disease.AS patients compared with the control group had significantly lower BMD and significantly higher N-Telopeptide levels (p < 0.05). |

**Table: Studies addressing the value of bone turnover markers as biomarkers for predicting therapeutic effect of bDMARDs**

| Study | Year | Design | Population | Treatment | duration | Result |
| --- | --- | --- | --- | --- | --- | --- |
| Briot[461] | 2005 | Cohort | 19 SpA | TNF-i | 12months | BMD increased at the spine (5.6%, p = 0.0005) and total femur (2.6%, p = 0.01). CTX decreased from the third month (-50%, p = 0.005) up to 1 year (-30%, p = 0.012), and a trend for an increase in PINP (10%, p = 0.06) and in IGF-I (15%, p = 0.04) was seen at month 3. This study confirm that treatment with TNF-a in SpA is associated with an increase of BMD, which results from a decrease of bone resorption. |
| Appel[262] | 2008 | Cohort | 34 SpA | Adalimumab | 2 years | Adalimumab-treated SpA patients had a significant decrease of VEGF (179.8 vs 155.37 vs 120.8,P < 0.001) and MMP-3 (28.5 vs 27.1 vs 15.83,P = 0.022) after 36 to 52 weeks of therapy,but increase of BALP(8.1 vs 8.8 vs 11.6,P < 0.001).This indicate that new bone formation in AS occurs if inflammation is successfully treated. |
| Visvanathan[462] | 2009 | Cohort | 201 AS | infliximab | 102 weeks | High baseline osteocalcin levels and early increases of BAP at week 2 were consistently associated with these increases in BMD(p<0.0001;p=0.039). |
| Arends[463] | 2012 | Cohort | 111AS | TNF-i | 3 years | The bone resorption marker sCTX Z-score decreased significantly after three months (P < 0.001) and remained decreased during three years of treatment.Baseline to three months change in sCTX Z-score was significantly associated with disease activity at last follow-up.This study indicated early change in the bone resorption marker sCTX seems useful as a purely objective biomarker in the evaluation of TNFa blocking therapy in AS. |
| Kwon[464] | 2012 | Cohort | AS | TNF-i | 3 months | Serum DKK-1 levels were significantly lower in the patients with AS than in the controls,but OPG and OC were significantly higher in patients with AS(P < 0.0001 for all).Serum osteocalcin levels increased significantly after 3 months of TNF-blocking therapy (P < 0.0001),and serum osteoprotegerin levels decreased (P = 0.025). |
| Saad[465] | 2012 | Cohort | AS | TNF-i | 12 months | AS patients had significantly lower sclerostin levels (60.5± 32.7 vs. 96.7± 52.9 pmol/L, P = 0.002) compared to healthy controls.Serum levels of sclerostin gradually increased from baseline vs. 6 months vs. 12 months after TNF-α treatment (60.5± 32.7 vs. 67.1± 31.9 vs. 72.7± 32.3 pmol/L, P<0.001) . |
| Korkosz[214] | 2014 | Cohort | AS | TNF-i | 6 months | Afer 6-months TNF inhibitor-treated,Dkk-1 decreased significantly from 196.8 pg/mL to 116.3 pg/mL(p=0.02)and BMP-7 increased significantly from 1.4 pg/mL to 20.3 pg/mL(p < 0.0001). |
| De Andrade[466] | 2014 | cross-sectional | SpA | HC  AS with no medication  AS wtih NSAIDs  AS with TNF-i |  | A significant difference between the controls and the patients without medication was observed in relation to BAP(controls: 20.75±9.07 U/L vs. SpA without medication: 39.45± 13.38 U/L, p<0.0001) and OP(controls: 134.44±62.36 ρmol/L vs. SpA without medication: 247.27± 92.42 ρmol/L, p<0.0001). |
| Li[467] | 2015 | Cohort | AS | Study group:TNF-I  Control group:SSZ | 12 months | Compared with the baseline, sCTX after 1 year treatment of anti-TNF was significantly decreased in the study group (–40% at 1 yr, p < 0.0001), and BALP and PINP were increased (+45.6%, p < 0.0001; +30.8%, p < 0.0001). |
| Sharma[250] | 2017 | Cohort | AS | TNF-i | 6 months | The serum ALP level increased from a mean value of 190.3± 70.8 IU/ml at baseline to 225.4± 59.8 IU/ml at the 6-month follow-up (P = 0.006).Anti-TNF-α has a beneficial effect on bone metabolism resulting in improved bone formation. |
| Gulyás[468] | 2020 | Cohort | AS | TNF-i | 12 months | Anti-TNF therapy slowed down generalized bone loss.In AS, P1NP levels significantly increased after 12 months (56.9± 28.8 μg/l; p = 0.035) versus baseline (49.2± 18.4 μg/ l).SOST significantly increased after 12 months (81.3± 46.9 pmol/l; p = 0.034) compared with baseline (70.6± 29.0 pmol/l) |
| Braun[469] | 2021 | Cohort | AS | secukinumab | 2 years | The study showed that after 2 years of treatment with secukinumab in AS, BMD of the lumbar spine, femoral neck and total hip was stable or increased, with the most striking findings at the lumbar spine,but was not correlated with biomarker changes. |

**Table: Studies addressing the value of bone turnover markers as biomarkers for predicting radiological progression**

| Study | Year | Design | Population | Result |
| --- | --- | --- | --- | --- |
| Park[470] | 2008 | cross-sectional | AS | Urinary CTX-I and CTX-II were elevated in men with AS and correlated well with disease activity, osteoporosis of the femur neck, and radiographic damage of the spine. |
| Vosse[471] | 2008 | Cohort | AS | Urine CTX-I and CTX-II were increased in patients with AS.CTX-II is associated with radiological-damage and -progression in AS(β=0.27,p<0.03;β=0.27,p<0.05). |
| Arends[472] | 2014 | cross-sectional | AS | Patients with bridging had significantly higher sCTX and PINP Z-scores than patients with non-bridging syndesmophytes or without syndesmophytes(239.4 vs 175.8,p<0.05;0.55 vs 0.04, p<0.05).This analysis in AS patients with active disease demonstrated that higher serum levels of sCTX, and to a lesser extent PINP, are associated with the presence of complete bridging. |
| Gamez-Nava[473] | 2016 | cross-sectional | AS | This research showed that higher OC levels in AS patients with syndesmophytes, whereas both OC and CTX levels are associated with higher mSASSS, which is a reflection of severe spinal damage. |
| Sun[474] | 2019 | cross-sectional | AS | Compared to the healthy controls, the serum sclerostin increased significantly in the AS patients (106± 6.75 vs. 62.78± 6.39 pmol/l, P< 0.05).Correlation analysis revealed a negative association between serum sclerostin and mSASSS (P=0.019, r2 = 0.062).No association between the serum levels of DKK-1 or sclerostin and disease activity assessed by ASDAS (P> 0.05). |
| Iaremenko[475] | 2020 | cross-sectional | SpA | The patients with SpA had significantly lower serum levels of Dkk-1 (p < 0.001), TGF-β1 (p < 0.001), and sclerostin (p < 0.001) compared with the healthy controls.The patients with SpA had significantly lower serum levels of Dkk-1 (p < 0.001), TGF-β1 (p < 0.001), and sclerostin (p < 0.001) compared with the healthy controls |
| Liu[476] | 2021 | cross-sectional | AS | All five bone turnover markers(25(OH)VD3,TP1NP,β-CTX,OC,PTH) showed no differences between RHI-AS and WORHI-AS.25(OH)VD3 had a correlation in AS with stiffness index (rho=0.279, P=0.001),the other four bone turnover markers(TP1NP,β-CTX,OC,PTH) showed no correlation.Treatment of vitamin D deficiency may be an effective way to improve bone strength in RHI-AS patients. |
| Kim[250] | 2012 | cross-sectional | AS | BALP levels were significantly higher in patients compared with those in controls (26.2±1.6 vs. 13.9±0.8 μg/L, p<0.001).BALP was higher in AS patients with syndesmophytes than those without, but these differences did not reach statistical significance (p00.128). |

**
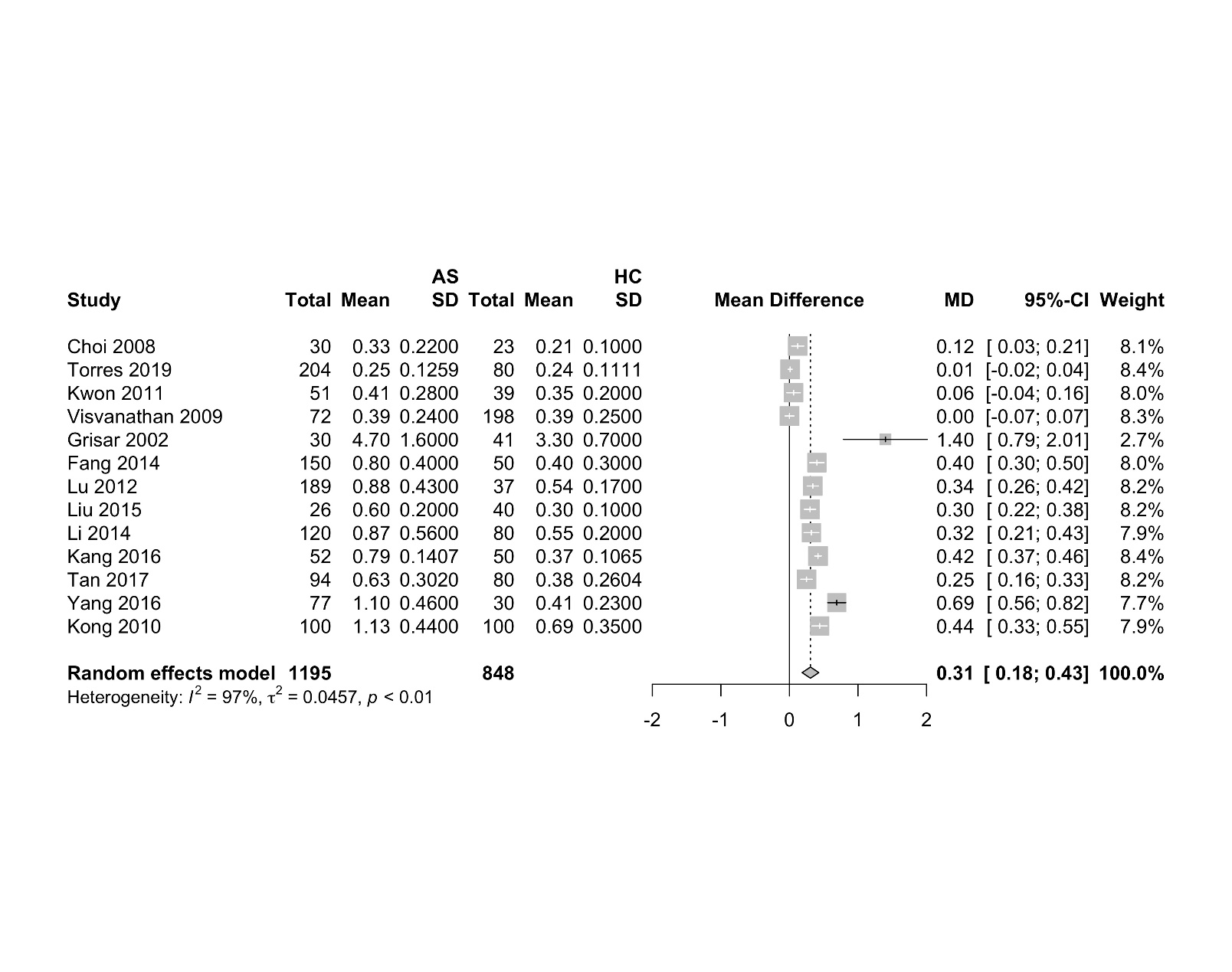
Figure: Forest Plot of CTX-I Level in the Serum in axSpA Patients and Healthy Control**

**
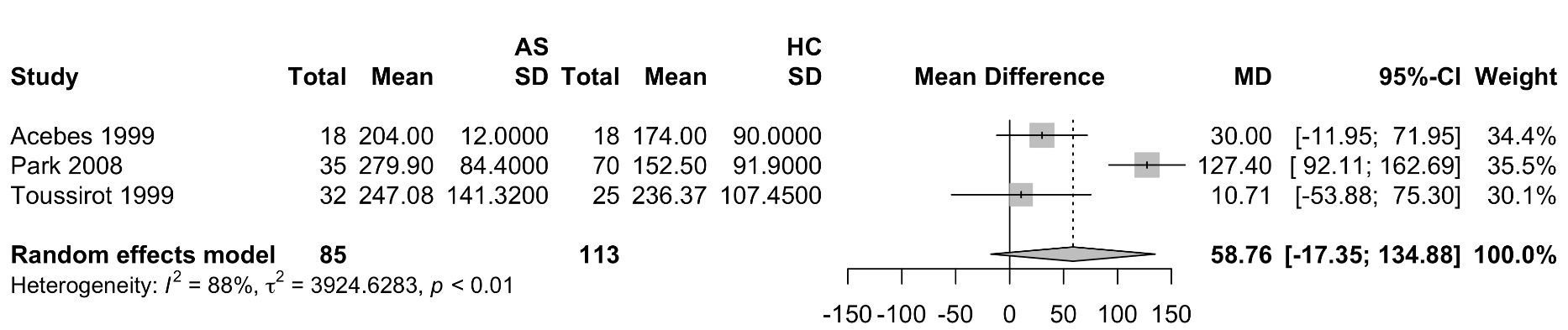
**

**Figure: Forest Plot of CTX-I Level in the Urine in axSpA Patients and Healthy Control**

**Figure: Forest Plot of BALP Level in the Serum in axSpA Patients and Healthy Contro
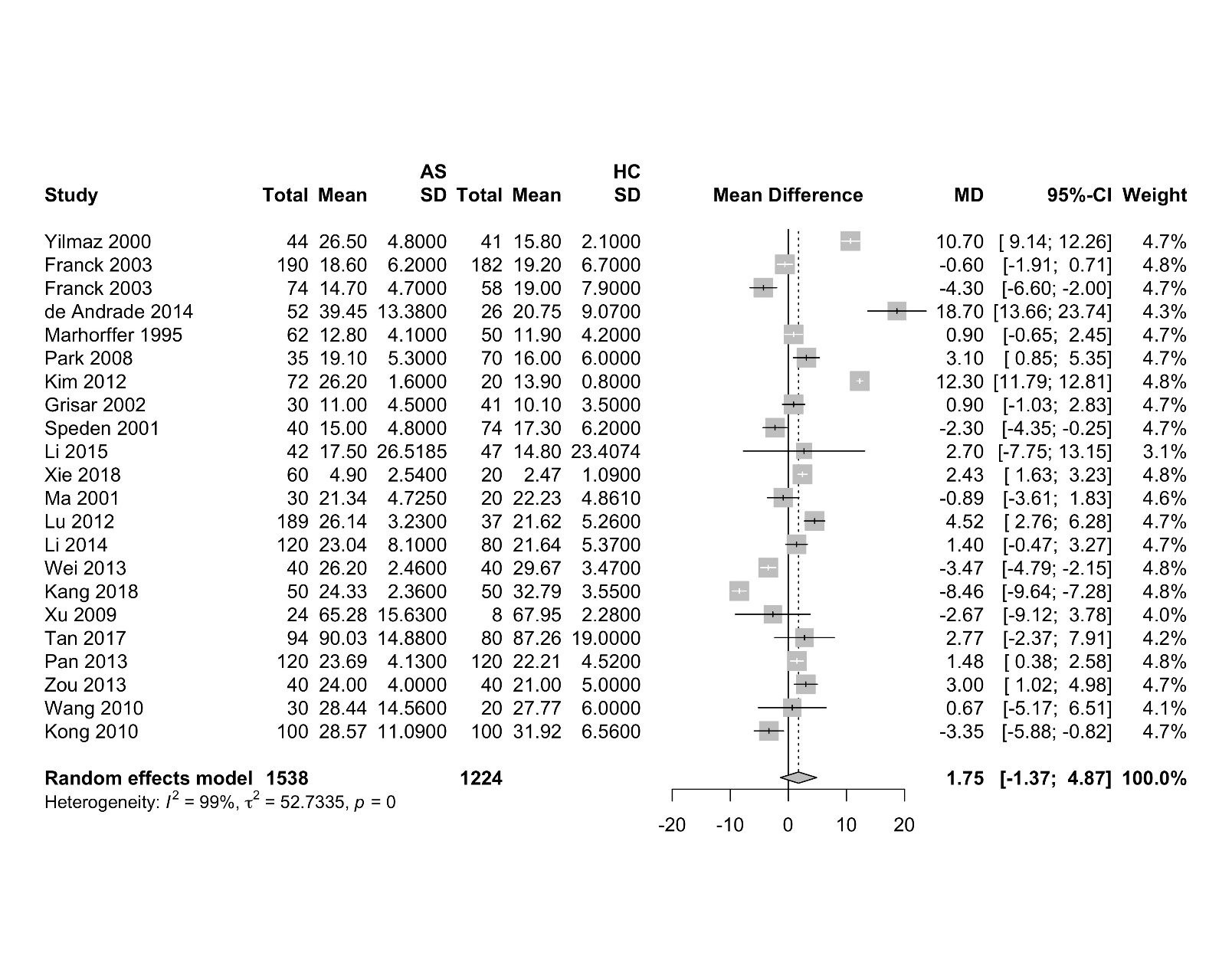
l**

**Figure: Forest Plot of DPD/creatinine ratio in the Urine in axSpA Patients and Healthy Control
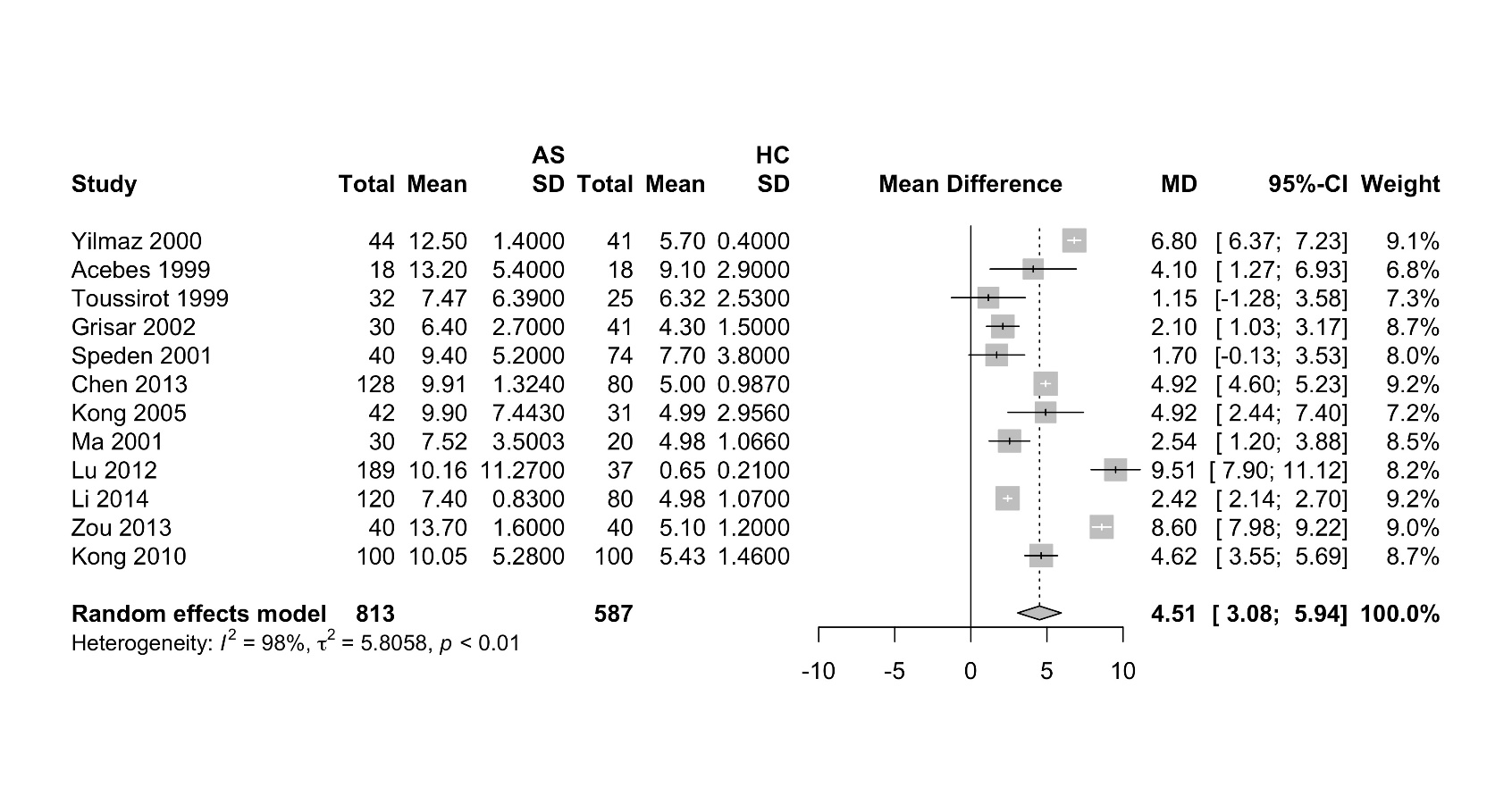
**

**
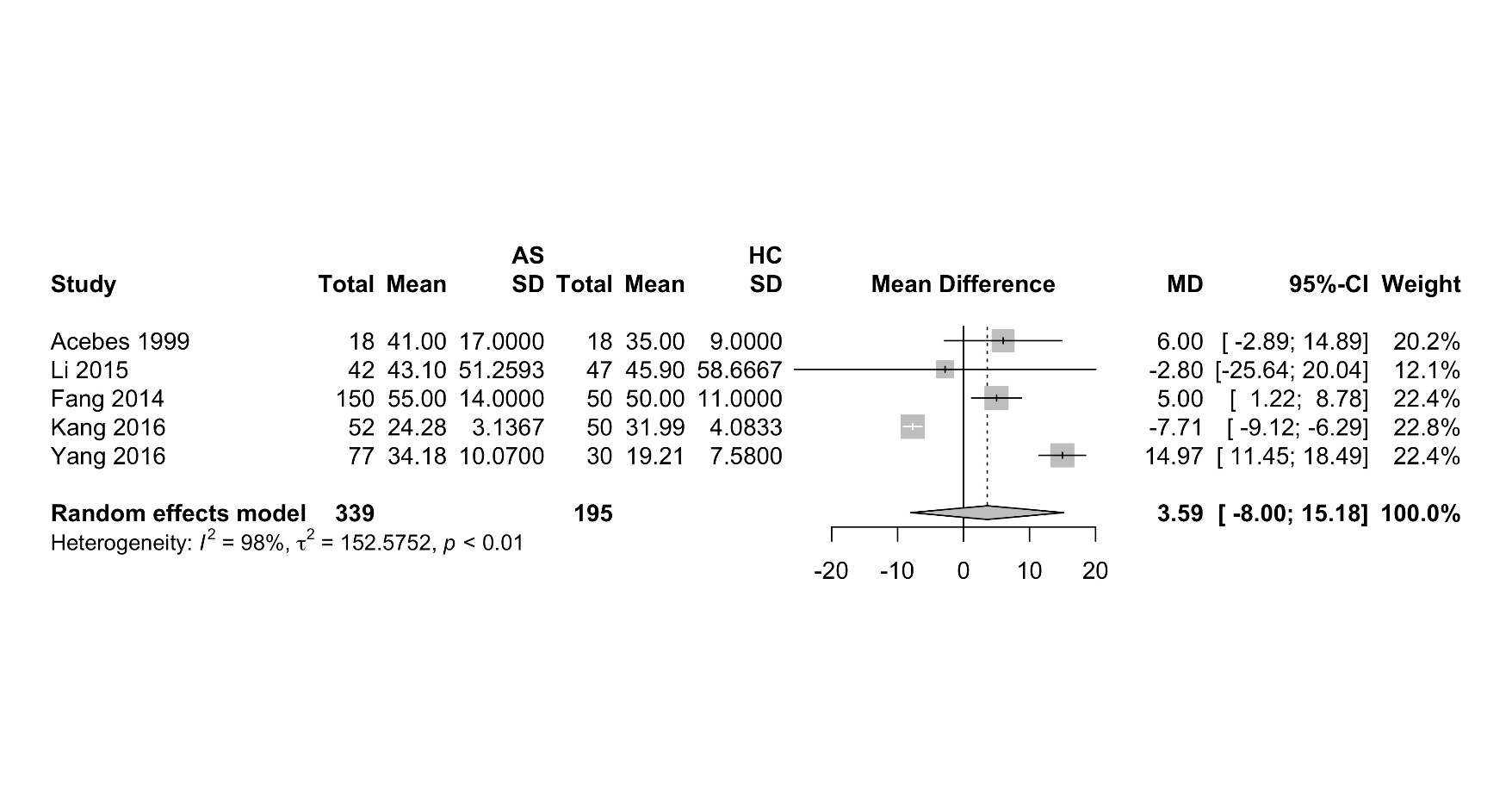
Figure: Forest Plot of PINP in the serum in axSpA Patients and Healthy Control**

**Question 15**: **C1M, C2M, C3M, C6M and VICM**

We found 5 Cohort, 3 case-control studies and 2 cross-sectional studies addressing this question.

The evidence shows:

1.C1M, C2M, C3M, C6M and VICM all belong to the group of degradation fragments of extracellular matrix, mostly generated by matrix metalloproteinase. They are believed to be indicators of connective tissue remodeling and could potentially reflect joint damage and radiographic progression.[477]

2.According to our systemic literature review, despite multiple reports revealing that such ECM fragments were elevated in patients with axSpA, these studies were mostly conducted by the same research team based in Denmark. More independent research is required to verify the results.

3.Association between ECM remodeling biomarkers and disease activity or radiographic progression is not consistent across different studies, especially after adjustments of confounding factors.

The quality of evidence is VERY LOW.

**Table: Evidence profile**

| Certainty assessment | | | | | | | Summary of findings | |
| --- | --- | --- | --- | --- | --- | --- | --- | --- |
| No of participants  (studies)  Follow-up | Risk of bias | Inconsistency | Indirectness | Imprecision | Publication bias | Overall certainty of evidence | Pooled Result (95%CI) | Brief Summary |
| Question 15: | | | | | | | | |
| 10 studies (5 Cohort, 3 case-control studies and 2 cross-sectional studies) | Serious | Serious | Not serious | Serious | Serious | ⨁◯◯◯  VERY LOW | \ | Despite multiple reports revealing that such ECM fragments were elevated in patients with axSpA, these studies were mostly conducted by the same research team based in Denmark. Association between ECM remodeling biomarkers and disease activity or radiographic progression is not consistent across different studies. |

**Table :** **Studies addressing the value of C1M, C2M, C3M, C6M or VICM as biomarkers for diagnosis**

| Study | Year | Design | Population | Result |
| --- | --- | --- | --- | --- |
| Bay-Jenson[478] | 2011 | Case-control | 40 AS patients  40 age- matched controls | The mean serum levels of C1M, C2M, C3M, C4M and C6M were significantly elevated in AS patients compared with age-matched controls. The highest diagnostic value was achieved when combining the C2M, C3M and C6M markers, AUC 87% (P < 0.0001). |
| Port[479] | 2022 | Case-control | 41 axSpA,  46 postpartum women, 25 disc herniation, 14 HC | When compared to healthy controls, patients with axSpA showed significantly higher levels of the ECM remodeling biomarkers (C1M, C4M, C6M, VICM, PRO-C4, C3M/PRO-C3; p < 0.0001, p < 0.0001, p < 0.0001, p < 0.01, p < 0.0001, p < 0.01, respectively) and significant decreased levels of type III collagen formation (PRO-C3, p ≤ 0.01). To discriminate between axSpA and healthy subjects, PRO-C4, C6M, and C1M presented the second, third, and fourth highest AUC after CRP. |
| Gudmann[480] | 2017 | Case-control | 110 axSpA  120 HC | For C1M healthy versus axSpA had an AUC value of 0.79 (CI 0.73–0.84), for C3M the AUC was close to those of C1M. Healthy versus axSpA had a value of 0.78 (0.72–0.83). C1M and C3M distinguish well between healthy and axSpA. |
| Bay-Jensen[481] | 2013 | Cohort | 201 AS  35 HC | The mean level of serum VICM at baseline was highest in the RA group (88.9 nmoles/liter [range 0.12–543]), followed by the AS group (16.4 nmoles/liter [range 0.12–123]), and was lowest in the control group (6.6 nmoles/liter [range 0.43–36.4]). In the ROC analysis, the AUC was 0.75. The highest positive likelihood ratio was calculated to be 9.8 at an optimal cutoff of 20.7 nmoles/liter. |
| Siebuhr[482] | 2019 | cross-sectional | 193 AS  100 HC | The best metabolite to differentiate between axSpA and controls was C3M (AUC 0.95; specificity 92.0, sensitivity 83.4). |

**Table : Studies addressing the value of C1M, C2M, C3M, C6M or VICM as biomarkers for indicating disease activity**

| Study | Year | Design | Population | Result |
| --- | --- | --- | --- | --- |
| Husakova[483] | 2017 | Cohort | 46 axSpA | We found no relationship of VICM, CRPM and C2M with disease duration, medication, disease activity (ASDAS-CRP, BASDAI and BASFI), all tested PROs or quality of life questionnaires. |
| Port[479] | 2022 | Case-control | 41 axSpA,  46 postpartum women, 25 disc herniation, 14 HC | ECM remodeling biomarkers are not strongly associated with clinical assessment of disease activity or severity of axSpA. |
| Gudmann[480] | 2017 | Case-control | 110 axSpA  120 HC | C3M levels were also associated with ASDAS in axSpA (r=0.28, p=0.003). C1M and C3M levels were both associated with DAS28 in axSpA (r=0.40, p<0.0001 and r=0.39, p<0.0001 respectively). |
| Bay-Jensen[481] | 2013 | Cohort | 201 AS  35 HC | Patients with VICM levels of >20.7 nmoles/ liter had significantly increased odds of having moderate disease activity (BASDAI tertile 2) (OR 7.7 [95% CI 1.7–35], P < 0.01) or high disease activity (BASDAI tertile 3) (OR 7.0 [95% CI 1.5–32], P < 0.05). Thus, at the optimal cutoff level of 20.7 nmoles/liter, VICM was associated with a higher burden of disease. |
| Husakova[484] | 2019 | cross-sectional | 193 AS  100 HC | C1M correlated with ASDAS-CRP in nr-axSpA (ρ = 0.37;p < 0.001) and AS (ρ = 0.57; p < 0.001). C1M, C3M, and C4M2 were associated with ASDAS-CRP in AS and nr-axSpA after adjustment for age, gender, and disease duration. |

**Table : Studies addressing the value of C1M, C2M, C3M, C6M or VICM as biomarkers for predicting radiological progression**

| Study | Year | Design | Population | Result |
| --- | --- | --- | --- | --- |
| Bay-Jenson[478] | 2011 | Case-control | 40 AS patients and 40 age- matched controls | The correlation with mSASSS was slightly increased using the composite index of C2M:C3M:C6M:OC. (OR=0.44, p=0.004) |
| Siebuhr[477] | 2018 | Cohort | 122 AS | Only C1M was significantly associated with mSASSS progression (β=0.01, 95% ci 0.00 to 0.03). the effect disappeared after adjustment for confounders. C5M, C6M and ViCM showed no relationship with mSASSS progression. |
| Bay-Jensen[481] | 2013 | Cohort | 201 AS  35 HC | In a logistic regression model that included VICM level of 7.2 nmoles/liter as a dichotomous independent variable, VICM was a significant predictor of 2-year progression in the mSASSS after adjustment for age, sex, disease duration, and baseline mSASSS. |
| Siebuhr[482] | 2019 | cross-sectional | 193 AS  100 HC | ASDAS-CRP correlated with CRPM and anti-MCV, but when adjusting for CRP the correlation only remained with CRPM. CRPM and VICM separated the subgroups with odds ratios of 1.19 and 1.10 adjusted for age, gender, BMI, and disease duration. VICM lost significance when adjusting for CRP. |

**Table : Studies addressing the value of C1M, C2M, C3M, C6M or VICM as biomarkers for predicting therapeutic effect of bDMARDs**

| Study | Year | Design | Population | Result |
| --- | --- | --- | --- | --- |
| Holm Nielsen[485] | 2022 | Cohort | 55 axSpA | Baseline levels of PRO‐C1, PRO‐C3, C6M, VICM, and CRP were all associated with ASDAS clinically important and major improvement after 22 weeks (ΔASDAS ≥1.1) (Mann–Whitney test, p=0.006, p=0.008, p<0.001, <0.001, <0.001, respectively), while C6M, VICM and CRP levels were associated with ASDAS clinically important and major improvement after 46 weeks (ΔASDAS ≥2.0) (p=0.002, p=0.044, and p<0.001, respectively). Baseline levels of PRO‐C4 and C6M were correlated with the total SPARCC MRI Spine and Sacroiliac Joint Inflammation score (Spearman’s Rho ρ=0.279, p=0.043 and ρ=0.496, p=0.0002, respectively). |
| Husakova[483] | 2017 | Cohort | 46 axSpA | We found that exercise therapy mainly in the nr-axSpA improves PROs, but not ECM turnover biomarkers. This indicates that exercise therapy is important for patients’ health but does not affect ECM turnover. |
| Siebhur[486] | 2016 | Cohort | 22 AS | ESR, CRP, BASDAI and C1M were decreased with treatment (p ≤ 0.04). C1M and CRP segregated patients into two populations predicting treatment efficacy. C1M and CRP were efficacy biomarkers and baseline biomarkers could select who benefited (by biomarkers) from treatment. |

**Question 16**: **Sclerostin**

We found 7 Cohort and 13 Cross-sectional studies addressing this question.

The evidence shows:

1.Sclerostin is an inhibitor of Wnt signaling pathway, which could inhibit osteoblast-induced new bone formation.

2.Despite heterogeneity of the studies investigating sclerostin levels in axSpA patients, we believed that sclerostin could be an indicator of bone formation activity, with previous reports showing that axSpA patients with lower sclerostin were more likely to exhibit radiographic progression[474, 487]. Consistent low levels of sclerostin might indicate unresolved inflammation.

3.Sclerostin is not correlated with disease activity.[474]

The quality of evidence is LOW.

**Table: Evidence profile**

| Certainty assessment | | | | | | | Summary of findings | |
| --- | --- | --- | --- | --- | --- | --- | --- | --- |
| No of participants  (studies)  Follow-up | Risk of bias | Inconsistency | Indirectness | Imprecision | Publication bias | Overall certainty of evidence | Pooled Result (95%CI) | Brief Summary |
| Question 16: | | | | | | | | |
| 20 studies (7 Cohort and 13 Cross-sectional studies) | Not Serious | Serious | Not serious | Not serious | Not serious | ⨁⨁◯◯  LOW | \ | Sclerostin could be an indicator of bone formation activity, with previous reports showing that axSpA patients with lower sclerostin were more likely to exhibit radiographic progression. .Sclerostin is not correlated with disease activity. |

**Table: Studies addressing the value of ESR as a biomarker for diagnosis**

| Study | Year | Design | Population | Result |
| --- | --- | --- | --- | --- |
| Aschermann[488] | 2016 | Germany | SpA | Sclerostin levels were significantly (P < 0.001) lower in HLA-B27+ subjects (314 ± 21 pg/mL) ,not only in SpA ,compared to HLA-B27 negative controls (492 ± 30 pg/mL). |

**Table: Studies addressing the value of sclerostin as a biomarker for indicating disease activity**

| Study | Year | Design | Population | Result |
| --- | --- | --- | --- | --- |
| Saad[465] | 2012 | Cohort | AS | AS patients with lower sclerostin serum levels had an increased risk of high CRP at 12 months than those with higher sclerostin values.Persistent low sclerostin serum levels are associated with continuous inflammation in AS patients under anti-TNF therapy |
| Taylan[447] | 2012 | cross-sectional | AS | The level of sclerostin was not different in AS patients from the healthy controls. In a subgroup analysis,level of sclerostin was also not difference between patients with active disease and the inactive group. |
| Korkosz[448] | 2013 | cross-sectional | AS | Sclerostin serum level was significantly higher in AS patients with high disease activity in comparison to healthy subjects. |
| Klingberg[450] | 2014 | cross-sectional | AS | The patients with AS had significantly lower levels of sclerostin (p = 0.014) compared with the controls. High CRP was associated with low sclerostin (p = 0.003). |
| Ustun[489] | 2014 | cross-sectional | AS | Serum sclerostin levels were significantly lower in the patients with AS than in the healthy controls (P = 0.037).But there were not different in AS patients with and without TNF-a treatment or those with active disease and inactive disease. |
| Rossini[490] | 2016 | cross-sectional | AS | Serum sclerostin was significantly lower in AS as compared to healthy controls (25.2±9.4 vs 38.0±17.2 pmol/L) and it significantly correlated with age. |
| Sakellariou[260] | 2017 | cross-sectional | AS | There was no significant difference in serum sclerostin between AS patients and controls. |
| Solmaz[491] | 2018 | cross-sectional | AS | Sclerostin levels was significantly lower in patients with very high disease activity compared with those with ASDAS-CRP scores of ≤3.5(p=0.016 ). |
| Iaremenko[475] | 2020 | cross-sectional | SpA | The sclerostin serum level had a weak negative correlation with the active inflammatory MRI SIJ lesions. |

**Table: Studies addressing the value of sclerostin as a biomarker for predicting therapeutic effect of bDMARDs**

| Study | Year | Design | Population | Result |
| --- | --- | --- | --- | --- |
| Pathan[492] | 2012 | Cohort | AS | Plasma sclerostin was statistically significant decreases in in the apremilast-treated responder of AS. |
| Atas[493] | 2022 | Cohort | axSpA | Sclerostin level increased significantly after anti-TNF treatment from 312.4 (140.8–412.7) to 405.1 (276.3–452.5) pg/ml (p = 0.018). |
| Guo[494] | 2023 | Cohort | AS | The levels of sclerostin were more significantly increased in the radiographic improvement group compared with those in the radiographic nonimprovement group after recieving 12-week treatment of imrecoxib or celecoxib. |
| Özdemirel[495] | 2023 | Cohort | AS | Higher serum sclerostin was shoed in AS patients than those of control group but was not affected after anti-TNF-a treatment. |

**Table: Studies addressing the value of sclerostin as a biomarker for predicting radiological progression**

| Study | Year | Design | Population | Result |
| --- | --- | --- | --- | --- |
| Appel[487] | 2008 | cross-sectional | AS | Serum levels of sclerostin were significantly lower in patients with AS than in healthy individuals.A low serum level of sclerostin in AS is linked to increased structural damage. |
| Tuylu[496] | 2014 | cross-sectional | AS | The levels of sclerostin was not different among the syndesmophyte-positive and -negative patients and healthy control groups (p>0.05). |
| Perrotta[497] | 2018 | cross-sectional | AS | Serum sclerostin levels were significantly higher in healthy controls (18.04 (13.6–24) pg/ml) than in AS patients (6.46 (4.5–11.1) pg/ml; P value < 0.01).However,no significant correlations were found between serum sclerostin levels and radiographic damage and between serum sclerostin levels and clinical indices of activity. |
| Rademacher[222] | 2019 | cross-sectional | SpA | There was not difference in serum sclerostin between patients with radiographic spinal progression and those without. |
| Sun[474] | 2019 | cross-sectional | AS | The correlation analysis revealed a negative association between serum sclerostin and mSASSS. |
| Rademacher[239] | 2022 | Cohort | AS | The change of sclerostin level after 3 months of TNFi therapy was positively associated with syndesmophyte progression at 2 years and sclerostin change after 2 years of therapy with syndesmophyte progression at 4 years. |

**Question 17**: **DKK-1**

We found 1 RCT, 1 meta-analysis, 10 case-control studies and 8 cross-sectional studies addressing this question.

The evidence shows:

1.Dickkopf-1 (DKK-1) is another inhibitor of the Wnt/β-catenin signaling pathway, which could competitively combine with LRP5/6, thus limiting the combination between Wnt and LRP5/6, ultimately inhibiting new bone formation.[498]

2.According to meta-analysis, DKK-1 levels were significantly lower in axSpA patients with elevated CRP levels and high mSASSS scores, indicating a negative correlation between DKK-1 and disease activity as well as radiographic progression. Lower levels of DKK-1 could be interpreted as higher risks for radiographic progression.[498]

2.Current studies suggested that treatment had a minimal effect on DKK-1 levels.

The quality of evidence is LOW.

**Table: Evidence profile**

| Certainty assessment | | | | | | | Summary of findings | |
| --- | --- | --- | --- | --- | --- | --- | --- | --- |
| No of participants  (studies)  Follow-up | Risk of bias | Inconsistency | Indirectness | Imprecision | Publication bias | Overall certainty of evidence | Pooled Result (95%CI) | Brief Summary |
| Question 17: | | | | | | | | |
| 20 studies (1 RCT, 1 meta-analysis, 10 case-control studies and 8 cross-sectional studies) | Not Serious | Not serious | Not serious | Not serious | Not serious | ⨁⨁◯◯  LOW | \ | According to meta-analysis, DKK-1 levels were significantly lower in axSpA patients with elevated CRP levels and high mSASSS scores, indicating a negative correlation between DKK-1 and disease activity as well as radiographic progression. |

**Table: Studies addressing the value of DKK-1 as a biomarker for diagnosis**

| Study | Year | Design | Population | Result |
| --- | --- | --- | --- | --- |
| Kwon[464] | 2012 | cross-sectional | 56AS,40HC | Serum levels of DKK-1 were significantly lower in the AS patients than in the controls (P < 0.0001). |
| Fassio[499] | 2017 | cross-sectional | 28PSA,43HC | Baseline mean Dkk-1 serum levels for the PsA arm were significantly lower than in the HC (p<0.05). |
| Niu[500] | 2017 | Cass-oontrol | China | serum levels of Dkk-1 levels were lower in AS patients than those in the controls. |
| Liao[501] | 2017 | Case-control | China | Dkk-1 was significantly lower in AS patients than in controls. |
| Bai[502] | 2017 | Case-control | China | The levels of DKK1 in experimental group were ( 0．72 ± 0．15) ng /ml, which in control group were ( 0．12 ± 0．03 ) ng /ml，respectively，( P＜ 0．05) ． |
| Rossini[490] | 2916 | Case comtrol | Italy | DKK1 serum levels were also significantly lower in AS patients than in healthy controls (23.3 ± 13.1 vs 29.8 ± 15.9 pmol/L, (p=0.009) |
| Huang[460] | 2016 | Case-control | China | The level of Dickkopf-1 was significantly higher in AS patients (1914.5±407.8 pg/mL) than in healthy controls (1729.1±352.9 pg/mL) (p<0.05). |
| Ustun[489] | 2014 | Cross-sectional | Turkey | Serum Dkk-1 levels were lower (P > 0.05) in the AS patients than in the controls. |
| Tuylu[496] | 2014 | Case-control | Turkey | dickkopf-1 was significantly higher in patients with ankylosing spondylitis compared with controls |
| Kwon[464] | 2012 | Case-control | Korea | Serum levels of DKK-1 were signiWcantly lower in the AS patients than in the controls (P < 0.0001). |
| Kim[377] | 2012 | Case-control | Korea | mean serum DKK-1 level (S.E.M) in AS patients was 341.8±55.1 pg/ml, which was similar to the serum levels of controls (mean±S.E.M. 239.6±32.4 pg/ml). |
| Daoussiss[503] | 2010 | Case control | Greece | Serum Dkk-1 levels were significantly increased in patients with AS (mean SEM 2,730±135.1 pg/ml) as compared with normal subjects (P=0.040), patients with RA (P=0.020), and patients with PsA (P=0.049). |
| Iaremenko[475] | 2020 | cross-sectional | Ukraine | Serum levels of Dkk-1 were signifcantly lower in SpA patients compared to healthy controls. |
| Nocturne[451] | 2015 | cross-sectional | France | Mean DKK-1 serum levels were higher in axial SpA patients than controls (30.03 ± 15.5 vs. 11.6 ± 4.2 pmol/L; p<0.0001). |
| Özdemirel[495] | 2023 | cross-sectional | Turkey | Serum DKK-1 levels in the AS group before anti-TNF-a treatment were significantly higher compared to the control group (p<0.01). |
| Papagoras[504] | 2022 | Case-control | Greece | At baseline, r-axSpA patients had significantly lower mean serum levels of Dkk-1 (1228 vs 3052pg/ml, p=0.001), compared to controls. |
| Atas[493] | 2022 | Case-control | Turkey | At baseline, axSpA patients had significantly lower median (IQR) DKK1 levels, 446.7 (356.9–529.3) vs. 1088.7 (951.7–1244.4) pg/ml, p < 0.001. |

**Table: Studies addressing the value of DKK-1 as a biomarker for indicating disease activity**

| Study | Year | Design | Population | Result |
| --- | --- | --- | --- | --- |
| Sakellariou[260] | 2017 | cross-sectional | 57AS,34HC | Dkk-1 levels were significantly (P <0.05) higher in AS patients with elevated ESR and CRP and no syndesmophytes, and were significantly (P <0.001) correlated with sclerostin levels (r = 0.592). In multivariate regression analyses, high Dkk-1 levels were significantly (P ≤ 0.001) associated with elevated ESR and CRP, no syndesmophytes and high sclerostin levels. |
| Liao[501] | 2017 | Case-control | China | The BMP-7/Dkk-1 ratio correlated significantly with sacroiliitis severity, Bath Ankylosing Spondylitis Radiology Index (BASRI)-total, modified Stoke Ankylosing Spondylitis Spinal Score, and disease duration. |
| Rossini[490] | 2916 | Case comtrol | Italy | DKK1 were inversely correlated to lumbar spine Z-score BMD and positively with BASFI, BASMI score, and mSASSS. A significant correlation was found between DKK1 serum levels and CRP (r = 0.240, p = 0.043). |
| Huang[460] | 2016 | Case-control | China | There was no correlation between high Dickkopf-1 level and any of the clinical parameters contributing to inflammation or bone formation. |
| Ustun[489] | 2014 | Cross-sectional | Turkey | There wasn’t a correlation between serum Dkk-1 levels, and disease activity indices (P > 0.05). BASRI scores did not correlate with serum Dkk-1 levels(P > 0.05). |
| Korkosz[448] | 2013 | Case-control | Poland | Dkk-1 level was significantly lower in high activity group compared to low activity group and healthy controls. There were no significant correlations between remodeling molecules, inflammatory markers, BASDAI and mSASSS (including lack of correlation between CRP and sclerostin) except negative correlation between sclerostin and Dkk-1 in high disease activity group (R = −0.28, P = 0.048). |
| Iaremenko[475] | 2020 | cross-sectional | Ukraine | The serum levels of Dkk-1 positively correlated with CRP. Dkk-1 had a signifcant negative correlation with Danish score. |
| Papagoras[504] | 2022 | Case-control | Greece | no correlations were identified between bone markers (Dkk-1) and disease activity indices. |

**Table: Studies addressing the value of DKK-1 as a biomarker for predicting radiological progression**

| Study | Year | Design | Population | Result |
| --- | --- | --- | --- | --- |
| Liao[501] | 2017 | Case-control | China | BMP-2/Dkk-1 was significantly correlated with disease duration. |
| Rossini[490] | 2916 | Case comtrol | Italy | Patients with one or more prevalent vertebral fractures had significantly higher DKK1 levels |
| Rubio Vargas[505] | 2017 | cross-sectional | Spain | Serum levels of DKK-1 were significantly higher in patients with early axSpA compared with established axSpA (22.1 ± 12.6 vs 16.4 ± 10.7PM; p = 0.04). Among all tested variables, only symptom duration was significantly and inversely correlated with DKK-1 serum levels (beta: −0.041; p = 0.01). |

**Table: Studies addressing the value of DKK-1 as a biomarker for predicting therapeutic effect of bDMARDs**

| Study | Year | Design | Population | Result |
| --- | --- | --- | --- | --- |
| Kwon[464] | 2012 | cross-sectional | 56AS,40HC | Serum DKK-1 level was lower in patients with AS than in healthy controls and did not change after 3 months of anti-TNF-α therapy in the AS patients despite the marked improvement in BASDAI scores. |
| Fassio[499] | 2017 | cross-sectional | 28PSA,43HC | Dkk-1 increased at Month 6 during the treatment with secukinumab (p<0.05 vs. baseline). When the PsA arm was compared to the HC, the difference between the serum levels of Dkk-1 lost significance at Month 6. |
| Liao[501] | 2017 | Case-control | China | Dkk-1 was higher in AS patients who received non-steroidal anti-inflammatory drugs (NSAIDs) regularly in the past year (p = 0.001). |
| Ustun[489] | 2014 | Cross-sectional | Turkey | Dkk-1 levels were similar in the patients that did and didn’t receive anti-TNF-a treatment, and in the patients with active and inactive disease (P > 0.05). |
| Kwon[464] | 2012 | Case-control | Korea | Serum levels of DKK-1 were not changed after the 3-month anti-TNF- therapy. |
| Guo[494] | 2023 | RCT | China | The levels of DKK-1, and RUNX2 were increased after the treatment of COX-2 inhibitors |
| Papagoras[504] | 2022 | Case-control | Greece | Anti-TNF treatment did not affect Dkk-1 levels. |
| Atas[493] | 2022 | Case-control | Turkey | DKK-1 levels increased significantly after anti-TNF treatment from 446.7 (356.9–529.3) to 881.3 (663.1–972.2) pg/ml (p < 0.001) |

**Question 18: OPG/RANKL/RANK**

We found 6 Cohort and 17 Cross-sectional studies addressing this question.

The evidence shows:

1.Although many studies reported that the RANKL, RANK and OPG levels were altered in patients with axSpA, results were highly inconsistent and exhibited significant heterogeneity.

2.More than half of the studies could not establish a correlation between RANKL, RANK or OPG and indices of disease activity. There is not sufficient evidence to confirm RANKL/RANK/OPG as an indicator of disease activity.

3.Only one study investigated the association between soluble RANKL and syndesmophyte formation.[506] There is not enough evidence to confirm the association between RANKL/RANK/OPG and radiographic progression.

The quality of evidence is VERY LOW.

**Table: Evidence profile**

| Certainty assessment | | | | | | | Summary of findings | |
| --- | --- | --- | --- | --- | --- | --- | --- | --- |
| No of participants  (studies)  Follow-up | Risk of bias | Inconsistency | Indirectness | Imprecision | Publication bias | Overall certainty of evidence | Pooled Result (95%CI) | Brief Summary |
| Question 18: | | | | | | | | |
| 23 studies (6 Cohort and 17 Cross-sectional studies) | Serious | Serious | Not serious | Not serious | Serious | ⨁◯◯◯  VERY LOW | \ | RANKL, RANK and OPG levels may alter in patients with axSpA. There is not sufficient evidence to confirm RANKL/RANK/OPG as an indicator of disease activity. There is not enough evidence to confirm the association between RANKL/RANK/OPG and radiographic progression. |

**Table: Studies addressing the value of OPG/RANKL/RANK as biomarkers for diagnosis**

| Study | Year | Design | Population | Result |
| --- | --- | --- | --- | --- |
| Beyazal[507] | 2016 | Case-control | 60 AS  50 HC | OPG levels were significantly higher in the AS patients than in the controls (106.7±50.9 vs. 58.1±12.7 pg/mL; P < 0.001). |
| Caparbo[508] | 2018 | Case-control | 85 AS  59 HC | A lower RANKL/OPG ratio was found in AS patients compared to the control group (0.05 ± 0.03 vs. 0.07 ± 0.07, p = 0.046) (Fig. 2), but no difference was observed in RANKL serum levels between the two groups, respectively (0.19 ± 0.11 vs. 0.23 ± 0.13, p = 0.066) and OPG serum levels (5.20 ± 3.18 vs. 4.34 ± 1.52, p = 0.199). |
| Chen[509] | 2010 | Case-control | 42 AS  26 HC | Serum levels of sRANKL [mean (SD), 4.75 (1.88) vs. 3.70 (1.14) pmol/l, p = 0.015] and OPG [mean (SD), 5.18 (1.19) vs. 4.52 (0.85) pmol/l, p = 0.026] were significantly higher in the 42 AS patients than the 26 healthy controls. |
| Dhir[510] | 2013 | Case-control | 85 AS  20 HC | Patients had higher mean (±SD) OPG level (649.7 ± 286.8, 389.3 ± 244.8 pg/mL, 𝑃 < 0.001). However, there was no difference in sRANKL (349.2 ± 872.0, 554.7 ± 1850.1, 𝑃 = ns). |
| Franck[457] | 2004 | Case-control | 264 AS  240 HC | OPG serum levels were significantly lower in patients with AS compared to controls (1.84 ± 1.15 vs 3.54 ± 2.18 pmol/l, p < 0.001). |
| Genre[511] | 2018 | Case-control | 163 axSpA  63 HC | Patients displayed higher OPG but lower SCL levels than controls (p=0.02 and 0.001, respectively). |
| Hou[512] | 2018 | Case-control | 40 AS  40 HC | The level of serum RANKL was significantly higher in AS patients than those in controls (3.136±0.2015 pmol/L vs 2.550±0.1983 pmol/L, p=0.0417) |
| Jadon[513] | 2017 | Case-control | 157 AS  50 HC | OPG concentrations were statistically no different in HC compared with any disease group. OPG concentrations were significantly lower (OR 0.20 per ng/mL increase; p = 0.02) in patients with axial arthritis (PsSpA and AS combined) than in those without (pPsA). |
| Kim[514] | 2006 | Case-control | 60 AS | Serum levels of sRANKL in AS patients were significantly higher than those in normal controls (1187.4±419.0pg/ml vs 301.0±110.3pg/ml, P=0.045). However, serum levels of OPG in AS patients were not different from those in normal controls (928.4±61.7pg/ml vs 1158.2±266.1pg/ml, P>0.05). In particular, the ratio sRANKL to OPG was clearly higher in AS than in controls (1.49±0.5 vs 0.34±0.1, P=0.033). |
| Klingberg[450] | 2014 | Case-control | 204 AS  80 HC | The patients with AS had significantly higher serum levels of Wnt-3a (p < 0.001), lower serum levels of sclerostin (p = 0.014), lower serum levels of sRANKL (p = 0.047), and lower sRANKL/OPG ratio (p = 0.022) compared with the healthy controls. |
| Korkosz[320] | 2018 | Case-control | 27 axSpA  23 HC | Sera levels of RANKL in axSpA patients appeared significantly lower (P = 0.016; median values: 8.5 vs. 272 pg/ml). OPG levels in axSpA did not differ from those in healthy blood donors (P = 0.18). |
| Kwon[464] | 2012 | Cohort | 56 AS  40 HC | Serum osteoprotegerin levels were significantly higher in patients with AS than in the controls (3.5±0.5 vs 2.0±1.0). |
| Liu[515] | 2021 | Cohort | 23 AS  15 HC | RANKL/OPG was significantly increased in AS patients (n = 23) compared with normal controls (n = 15) (p < 0.05). RANKL was increased and OPG was decreased in AS patients compared with healthy subjects, without statistical significance. |
| Mou[516] | 2015 | Case-control | 68 juvenile-onset AS  32 HC | The levels of sRANKL in the juvenile-onset ankylosing spondylitis group were significantly higher than those in the control group (790.20 vs 452.13, p=0.008), and OPG was slightly higher in juvenile-onset ankylosing spondylitis (82.62 vs 58.16, p=0.070). |
| Niu[500] | 2017 | Case-control | 6 AS  9 HC | Serum levels of OPG (*p < 0.05) were lower in the patients with AS than in the controls (13.4±2.3 vs 26.1±15.3). |
| Stupphann[517] | 2008 | Case-control | 21 AS  15 HC | Patients with AS showed mean serum OPG levels of 3.3 ± 0.4 pmol/l (normal range from 1.2 to 6.6 pmol/l) and soluble RANKL 0.1 ± 0.0 pmol/l (normal range from 0.0 to 2.7 pmol/l) which were both within the normal range. |
| Sveaas[317] | 2015 | Case-control | 143 AS  124 HC | Analysis of covariance (ANCOVA) demonstrated elevated plasma levels of OPG (2.3, 95% CI 2.1–2.4 vs. 2.0, 95% CI 1.9–2.2 ng/mL, p 1⁄4 0.02) in AS patients vs. controls. |
| Taylan[447] | 2012 | Cohort | 55 AS  33 HC | The levels of sRANKL were similar between the patients and controls (p > 0.05). OPG levels were significantly lower in the AS group [339 (52–1118) vs. 527 (16–1030) pg/ mL, p<0.05]. |

**Table: Studies addressing the value of OPG/RANKL/RANK as biomarkers for indicating disease activity**

| Study | Year | Design | Population | Result |
| --- | --- | --- | --- | --- |
| Beyazal[507] | 2016 | Case-control | 60 AS  50 HC | Statistically significant correlation was observed between serum OPG levels and ESR, BASFI, or ASDAS (r = 0.275, P = 0.033; r = 0.292, P = 0.024; r = 0.272, P = 0.036, respectively). |
| Chen[509] | 2010 | Case-control | 42 AS  26 HC | Serum OPG levels correlated significantly with ESR (r = 0.417, p = 0.007), CRP (r = 0.524, p < 0.001), tragus-to-wall distance (r = 0.556, p < 0.001), fingertip-to-floor distance (r = 0.423, p = 0.007), and occiput-to-wall distance (r = 0.465, p = 0.002) and correlated inversely with modified Schober index (r = −0.525, p = 0.001), cervical rotation (r = −0.403, p = 0.022), lateral lumbar flexion (r = −0.587, p < 0.001), and chest expansion (r = −0.553, p < 0.001). |
| Dhir[510] | 2013 | Case-control | 85 AS  20 HC | There was no correlation between BASDAI and levels of OPG (𝑟 = −0.05), MMP-3 (𝑟 = −0.04), or TIMP- 1 (−0.01, 𝑃 = 𝑛𝑠). There was also no correlation between ESR and OPG (𝑟 = −0.12) or MMP-3 (𝑟 = 0.09, 𝑃 = 𝑛𝑠). |
| Genre[518] | 2014 | Cohort | 30 AS | We found a positive correlation between OPG levels and markers of disease activity such as BASDAI and VAS spinal pain (r=0.497, p=0.01; r=0.390; p=0.04, respectively). Nevertheless, no differences in OPG concentration were observed when patients with a history of anterior uveitis, presence of syndesmophytes, hip involvement or synovitis in other peripheral joints and peripheral enthesitis were compared with the remaining patients who did not exhibit these clinical characteristics |
| Grisar[458] | 2002 | Case-control | 30 AS  41 HC | The serum levels of OPG were increased in patients with AS (44.3 ± 19.7 ng/ml; p = 0.046) compared to controls (35.2 ± 10.0 ng/ml). No significant correlation between OPG and markers of inflammation was observed (data not shown). |
| Guo[494] | 2023 | Cohort | 116 axSpA | We did not observe any significant differences in the levels of OPG compared with those in patients with active disease (P > 0.05). |
| Kim[514] | 2006 | Case-control | 60 AS | There was no correlation between sRANKL:OPG ratio and clinical data, such as CRP, ESR, HLA-B27 and AS disease activity indexes, disease duration or BMD. |
| Klingberg[450] | 2014 | Case-control | 204 AS  80 HC | No significant correlations were found between the biomarkers analyzed and BASDAI, ASDAS-CRP, BAS-G, ESR, or hemoglobin. |
| Mou[516] | 2015 | Case-control | 68 juvenile-onset AS  32 HC | There was a positive association between sRANKL and MMP-3 but a negative association between sRANKL and disease duration. And OPG had no association with other indicators. |
| Sveaas[317] | 2015 | Case-control | 143 AS  124 HC | ASDAS was significantly associated with OPG (p=0.01). After adjustments for gender, age, BMI, and smoking status, the association between ASDAS and OPG was maintained, but only borderline statistically (p = 0.05). BASDAI was significantly associated with OPG (p = 0.03) in the univariate analyses but was not associated with any of the inflammatory biomarkers measured after adjustments for gender, age, BMI, and smoking status. |

**Table: Studies addressing the value of OPG/RANKL/RANK as biomarkers for predicting radiological progression**

| Study | Year | Design | Population | Result |
| --- | --- | --- | --- | --- |
| Liu[515] | 2021 | Cohort | 23 AS  15 HC | OPG was negatively correlated with T-scores of femoral neck (p < 0.05). However, no significant correlation with BMP-2, BMP-6, BMP-7, DKK-1, OPN, PDGF-BB, TGF-β3, and RANKL was found (Table S3). |
| Stupphann[517] | 2008 | Case-control | 21 AS  15 HC | Serum OPG levels were negatively correlated with lumbar spine BMD values, measured by QCT (r = -0.571; P = 0.025) and—although statistically not significant—they also tended to be negatively correlated with all other BMD measurements. |
| Wang[506] | 2019 | Case-control | 22 AS  22 HC | AS patients with syndesmophyte formation had significantly lower sRANKL serum levels (N =22, mean±SEM=0.54573±0.14767) than did those without syndesmophyte formation (N = 22; mean ± SEM = 1.2488 ± 0.24056; p = 0.0054). |

**Table: Studies addressing the value of OPG/RANKL/RANK as biomarkers for predicting therapeutic effect of bDMARDs**

| Study | Year | Design | Population | Result |
| --- | --- | --- | --- | --- |
| Genre[518] | 2014 | Cohort | 30 AS | An infliximab infusion did not lead to a significant reduction in OPG levels. |
| Guo[494] | 2023 | Cohort | 116 axSpA | We found that the levels of VEGF and β-catenin were decreased, whereas those of sclerostin, OPG, noggin, DKK-1, and RUNX2 were increased. |
| Liu[515] | 2021 | Cohort | 23 AS  15 HC | Following 6 months of TNF-α inhibitor treatment, 12 patients showed significantly decreased (p < 0.05) RANKL and RANKL/OPG. |
| Woo[519] | 2007 | Cohort | 26 AS | There were no statistically significant differences between the baseline and 12-week values of RANKL and OPG. |

**Question 19**: **MMP3**

We found 9 Cohort and 3 cross-sectional studies and 8 case-control studies addressing this question.

The evidence shows:

1.Literature review showed that serum matrix metalloproteinase 3 (MMP3) is significantly higher in patients with radiographic axial spondyloarthritis, and it seemed to be associated with articular destruction.[520]

2.Levels of MMP3 could potentially reflect disease activity and therapeutic response to TNFi, but there is significant inconsistency between studies, hampering the reliability of this biomarker. The literature review team decided that more high-quality studies were required to increase the certainty of evidence.

The quality of evidence is VERY LOW.

**Table: Evidence profile**

| Certainty assessment | | | | | | | Summary of findings | |
| --- | --- | --- | --- | --- | --- | --- | --- | --- |
| No of participants  (studies)  Follow-up | Risk of bias | Inconsistency | Indirectness | Imprecision | Publication bias | Overall certainty of evidence | Pooled Result (95%CI) | Brief Summary |
| Question 19: | | | | | | | | |
| 20 studies (9 Cohort and 3 cross-sectional studies and 8 case-control studies) | Serious | Serious | Not serious | Not serious | Serious | ⨁◯◯◯  VERY LOW | \ | Serum MMP3 is significantly higher in patients with radiographic axial spondyloarthritis, and Levels of MMP3 could potentially reflect disease activity and therapeutic response to TNFi. |

**Table: Studies addressing the value of MMP-3 as a biomarker for indicating disease activity**

| Study | Year | Design | Population | Result |
| --- | --- | --- | --- | --- |
| Lorenzin[521] | 2019 | cross-sectional | 75 AS | Correlations were found between MMP3 and ESR, hsCRP and mSASSS SIJ scores. |
| Torres[261] | 2019 | case-control | 204 AS, 80 HC | ESR, CRP, and WBC were all positively correlated with serum levels of MMP-3. MMP-3 were positively correlated with swollen joints count. |
| He[319] | 2017 | case-control | 24 AS, 10 HC | CRP was positively correlated with serum MMP3 and IL6. serum MMP3 level was found to have a positive correlation with the MRI score of SIJ and CRP. |
| Mou[516] | 2015 | case-control | 56 JAS, 32 HC | Serum levels of MMP-3 showed positive correlation with BASDAI and BASFI (r = 0.590 and 0.542, respectively, p < 0.01). |
| Almodovar[449] | 2014 | cross-sectional | 60 early SpA | Serum levels of MMP-3 showed no correlation with BASDAI, ASDAS or BASFI. The presence of sacroiliitis on MRI was unrelated with MMP-3. |
| Ramonda[522] | 2013 | cohort | 43 PsA | A correlation was found between MMP3 and hs-CRP (r = 0.45, P = 0.0005). |
| Soliman[523] | 2012 | case-control | 30 AS, 10 HC | High MMP3 was significantly associated with high BASDAI among AS patients (P = 0.046). Patients with elevated MMP-3 had statistically higher BASMI (P = 0.013) and BASFI (P = 0.053) than patients with low MMP-3. As regards the association between the MRI activity and MMP-3 levels, there was no statistically significant relation. Bone marrow edema was detected in 52.63% of AS with high MMP-3 levels compared to 47.37% of patients with low MMP-3 (P = 0.919). |
| Mattey[314] | 2012 | cross-sectional | 157 AS | MMP-3 levels were correlated with CRP levels but not with the BASDAI or BASFI. |
| Ardend[524] | 2011 | cohort | 92 AS | At baseline, no statistically significant correlations were found between serum MMP-3 levels and clinical assessments of disease activity or physical function in male patients. In female patients, baseline serum MMP-3 levels correlated positively with baseline CRP and ASDAS scores (p < 0.05). |
| Wendling[525] | 2008 | case-control | 23 AS, 21 HC | MMP-3 is significantly increased in patients with active AS but fails to correlate significantly with ESR, CRP, or BASDAI. |
| Appel[262] | 2008 | cohort | 71 AS | MMP-3 correlated well to CRP at baseline (r = 0.291, P = 0.014) but not to CRP after 2 years (r = 0.112, P > 0.05). MMP-3 did not correlate to ESR at baseline or after 2 years (r = 0.011, P > 0.05 and r = -0.139, P > 0.05) or to BASDAI (r = -0.039, P > 0.05 and r = -0.062, P > 0.05). |
| Maksymowych[526] | 2008 | cohort | 82 AS | At 12 and 24 weeks, significant reductions in MMP-3 was observed for adalimumab versus placebo (p<0.001). Significant baseline correlations were noted between CRP and MMP-3 (r=0.45) (p<0.001). Changes in MMP-3 at 12 weeks correlated significantly with changes in BASDAI (r=0.33), and CRP (r=0.43) (p<0.005). |
| Chen[527] | 2006 | case-control | 42 AS, 20 HC | Within AS patients, MMP-3 levels were higher in patients with high disease activity compared with those with low disease activity, and correlated significantly with BASDAI (r = 0.366, P = 0.017) and functional indices (r = 0.344, P = 0.026). The correlation with BASDAI was stable in a 1-yr follow-up (r = 0.464, P = 0.095) and reproducible with two different enzyme-linked immunosorbent assays. Using receiver operating characteristic plots to analyse the two cohorts, MMP-3 was more accurate than ESR and CRP in detecting AS patients with high disease activity (P = 0.01 and P = 0.009, respectively). |
| Yang[528] | 2004 | case-control | 41 AS, 28 HC | In the group of AS patients not treated with biologics, MMP-3 correlated with the Bath Ankylosing Spondylitis Disease Activity Index (BASDAI) values. Logistic regression analysis showed that MMP-3 values were high in those with severely active disease. |

**Table: Studies addressing the value of MMP-3 as a biomarker for predicting therapeutic effect of bDMARDs**

| Study | Year | Design | Population | Result |
| --- | --- | --- | --- | --- |
| Kaaij[529] | 2020 | cohort | 20 pSpA | Serum levels of MMP-3 significantly decreased after secukinumab therapy (P < 0.0001). |
| Torres[261] | 2019 | case-control | 204 AS, 80 HC | Serum MMP-3 was not associated with use of glucocorticoids or TNFi. |
| Turina[530] | 2014 | case-control | 78 SpA, 20 HC | Infliximab treatment for 2 weeks did not decrease serum level of MMP-3 significantly (P = 0.063). But a significant decrease in serum levels of MMP-3 was observed after etanercept treatment for 4 weeks (P = 0.045). |
| He[319] | 2017 | case-control | 24 AS, 10 HC | Serum levels of MMP3 was significantly higher in AS patients at baseline. After etanercept treatment, the levels of MMP3 decreased significantly﻿. |
| Ramonda[522] | 2013 | cohort | 43 PsA | MMP3 levels were significantly lower after 6 months of anti-TNF-α therapy (P < 0.0001), and remained low after 12 months (P < 0.0001) and 24 months (P < 0.0001). |
| Chandran[531] | 2013 | cohort | 40 PsA | After a mean treatment duration of 11 months with TNFi (etanercept 28 patients, adalimumab 6, golimumab 4, infliximab 2), 29 patients were classified as TNFi responders. Baseline level of MMP-3 was independently associated with responder status (OR 1.067 for each 1-unit increase, p = 0.045). A reduction in MMP-3 levels with therapy increased the odds of achieving response (OR 1.213 for each 1-unit change, p = 0.030). |
| Pedersen[325] | 2011 | cohort | 60 SpA, 333 HC | During treatment with TNF-α inhibitors, there were significant decreases from week 2 to week 46 in CRP (P < 0.002) and MMP-3 (P < 0.03). |
| Ardend[524] | 2011 | cohort | 92 AS | After 3 and 12 months of etanercept treatment, changes in serum MMP-3 levels correlated positively with changes in BASDAI, ESR, CRP, ASDAS, physician GDA, and patient GDA scores in male patients (p < 0.05). In female patients, there were significant positive correlations between changes in serum MMP-3 levels and changes in physician GDA scores after 3 months as well as changes in BASDAI, CRP, ASDAS, and BASFI scores after 12 months (p < 0.05). |
| Wendling[525] | 2008 | case-control | 23 AS, 21 HC | Thirteen patients were evaluated 10 weeks into TNF-α antagonist therapy (adalimumab, n=7; etanercept, n=4; or infliximab, n=2). Serum MMP-3 decreased significantly (P=0.04); significant decreases were also noted for the ESR, CRP, and BASDAI. |
| Appel[262] | 2008 | cohort | 34 SpA | MMP-3 were 28.0 ± 28.5 ng/mL before adalimumab therapy and decreased significantly to 23.5 ± 27.1 ng/mL after 12 weeks and to 19.02 ± 15.83 ng/mL after 36 to 52 weeks of therapy (P = 0.022). |
| Maksymowych[526] | 2008 | cohort | 82 AS | At 12 and 24 weeks, significant reduction in MMP-3 was observed for adalimumab versus placebo (p<0.001). Significant baseline correlations were noted between CRP and MMP-3 (r=0.45) (p<0.001). Changes in MMP-3 at 12 weeks correlated significantly with changes in BASDAI (r=0.33), and CRP (r=0.43) (p<0.005). |
| Woo[519] | 2007 | cohort | 26 AS | Serum levels of MMP-3 were significantly decreased after 12 weeks of etanercept treatment (p < 0.05). Change of MMP-3 had a high correlation coefficient with changes of CRP and ESR upon etanercept treatment (CRP, r = 0.446, p = 0.022; ESR, r = 0.449, p = 0.021). |
| Yang[528] | 2004 | case-control | 41 AS, 28 HC | Infusions of infliximab in AS patients led to a significant decrease in the values of the BASDAI as well as the serum MMP-3. |

**Table: Studies addressing the value of MMP-3 as a biomarker for predicting radiological progression**

| Study | Year | Design | Population | Result |
| --- | --- | --- | --- | --- |
| Pedersen[325] | 2011 | cohort | 60 SpA, 333 HC | No associations were found between radiographic progression and pretreatment levels of serum MMP-3 or CRP. |
| Maksymowych[532] | 2007 | cohort | 97 AS | After adjustment for sex, age, disease duration, C-reactive protein level, and baseline mSASSS, only MMP-3 was significantly associated with 2-year progression (beta = 0.29, P = 0.004). Logistic regression analysis revealed MMP-3 (cutoff 68 ng/ml; odds ratio 9.4 [95% confidence interval 1.6-56]) and baseline mSASSS (cutoff 10 mSASSS units; odds ratio 18.6 [95% confidence interval 2.5-138]) as the only independent predictors of 2-year progression (cutoff 3 mSASSS units; model R(2) = 50%). MMP-3 was primarily contributory in patients who already had substantial baseline damage (>10 mSASSS units). |

**Question 20**: **BMP2**

We found 1 Cohort and 6 case-control studies addressing this question.

The evidence shows:

1.BMP-2 could induce the differentiation of osteoblast and chondroblast by activating Smad signals, facilitating new bone formation.

2.Previous meta-analysis confirmed that BMP2 levels were significantly elevated in axSpA patients.[533] However, no reliable study could confirm that it is a predictor of radiographic progression, and the majority of studies could not identify a correlation between BMP-2 and disease activity.

The quality of evidence is VERY LOW.

**Table: Evidence profile**

| Certainty assessment | | | | | | | Summary of findings | |
| --- | --- | --- | --- | --- | --- | --- | --- | --- |
| No of participants  (studies)  Follow-up | Risk of bias | Inconsistency | Indirectness | Imprecision | Publication bias | Overall certainty of evidence | Pooled Result (95%CI) | Brief Summary |
| Question 20: | | | | | | | | |
| 7 studies (1 cohort and 6 case-control studies) | Not Serious | Serious | Not serious | Not serious | Serious | ⨁◯◯◯  VERY LOW | / | Previous meta-analysis confirmed that BMP2 levels were significantly elevated in axSpA patients. However, no reliable study could confirm that it is a predictor of radiographic progression, and the majority of studies could not identify a correlation between BMP-2 and disease activity. |

**Table: Studies addressing the value of BMP-2 as a biomarker for indicating disease activity**

| Study | Year | Design | Population | Result |
| --- | --- | --- | --- | --- |
| Guo[494] | 2023 | cohort | 116 axSpA | Taking a BASDAI score of ≥4 as the standard for high disease activity, we identifed 60 cases with high disease activity at the time of enrollment, 39 cases with disease activity in remission after 12 weeks of treatment, and 21 cases without remission. No signifcant diference in the BMP-2 levels was found in patients with disease remission compared with those in patients with active disease (P > 0.05). |
| Ozdemirel[534] | 2022 | case-control | 100 AS, 102 HC | In the examination performed by classifying AS patients as active and inactive disease according to BASDAI values, no significant difference was found between BMP-2 levels and disease activity (p>0.05). Similarly, no significant difference was found between disease activity and BMP-2 levels in AS patients according to ASDAS-CRP (p>0.05). No significant correlation was found between ESR and CRP levels and BMP-2 and BMP-4 levels in RA and AS patients. |
| Liao[501] | 2017 | case-control | 72 AS, 30 HC | The largest AUCs correlating with BASDAI, BASFI, and BASG were those of BMP-2/Dkk-1, BMP-2, and BMP-2 [AUC = 0.868 (p < 0.001),0.723 (p = 0.026), and 0.810 (p = 0.013), respectively]. The AUCs of BMP-2 and BMP-2/Dkk-1 were significantly larger than those of ESR and CRP. The AUCs suggested that BMP-2/Dkk-1 and serum BMP-2 are good indicators to predict disease activity, functional index, and patient global assessment in AS patients. |
| Tosovsky[265] | 2014 | case-control | 21 AS patients treated with TNFi and 42 untreated | A mild correlation between serum levels of BMP-2 and CRP (r = 0.28; p = 0.036) and even no correlation between BMP-2 and BASDAI (r = 0.056, p = 0.68) were found. |
| Park[535] | 2008 | case-control | 40 AS, 40 HC | In AS patients, BMP-2 levels showed significant correlations with BASDAI (p < 0.05). During the follow-up period, The changes in BMP-2 levels (p < 0.05) correlated well with the changes of BASDAI. |

**Table: Studies addressing the value of BMP-2 as a biomarker for predicting therapeutic effect of bDMARDs**

| Study | Year | Design | Population | Result |
| --- | --- | --- | --- | --- |
| Ozdemirel[495] | 2023 | case-control | 53 AS, 50 HC | When BMP-2 levels were compared before and after anti-TNF-a treatment for 40 patients, no statistical difference was observed (p>0.05). |
| Tosovsky[265] | 2014 | case-control | 21 AS patients treated with TNFi and 42 untreated | In patients treated with TNFα inhibitors, no difference was found between the BMP-2 levels in comparison with the untreated patients (254.8 (230.1; 267.3) vs. 261.1 (248.6; 273.5) pg/ml; p = 0.24). |

**Table: Studies addressing the value of BMP-2 as a biomarker for predicting radiological progression**

| Study | Year | Design | Population | Result |
| --- | --- | --- | --- | --- |
| Guo[494] | 2023 | cohort | 116 axSpA | Based on the diference in changes in SPARCC scores (dSPARCC) before and after treatment, we dichotomized patients into the radiographic improvement (dSPARCC <-1.0) and radiographic nonimprovement (dSPARCC >-1.0) groups. No signifcant diference in the BMP-2 levels was found in the radiographic improvement group compared with those in the radiagraphic nonimprovement group (P > 0.05). |
| Chen[536] | 2010 | case-control | 120 AS, 40 HC | Patients with spinal fusion had higher levels of BMP-2 than either controls (98.0 ± 188.5 vs 49.4 ± 20.3 pg/ml; p < 0.001) or patients without spinal fusion (98.0 ± 188.5 vs 86.6 ± 136.5 pg/ml; p <0.001), but there was no difference between the latter 2 groups. BMP-2 and BMP-4 levels had a significant correlation with spinal radiograph scores, especially for BASRI of the lumbar spine (r = 0.356 and 0.348, respectively, p < 0.001). Serum BMP levels also correlated significantly with the number of fused vertebrae. |

**Question 21: TNC**

We found 4 case-control studies addressing this question.

The evidence shows:

1.Tenascin-C is believed to be a pro-inflammatory extracellular matrix glycoprotein, secreted by myeloid cells in response to stimulation such as mechanical strain or pathogens.[537]

2.Only four studies were included in our systemic literature review. These studies consistently reported that TNC was elevated in the serum of axSpA patients. However, most of these studies did not identify a correlation between serum TNC levels and disease activity or radiographic progression.

3.TNC is a relatively new biomarker identified in the pathogenesis of axSpA, with the first report published in 2018.[538] Given the limited number of related studies, the literature review team decided not to formulate a recommendation for or against this biomarker yet, but this biomarker shall be revisited in a couple of years.

The quality of evidence is LOW.

**Table: Evidence profile**

| Certainty assessment | | | | | | | Summary of findings | |
| --- | --- | --- | --- | --- | --- | --- | --- | --- |
| No of participants  (studies)  Follow-up | Risk of bias | Inconsistency | Indirectness | Imprecision | Publication bias | Overall certainty of evidence | Pooled Result (95%CI) | Brief Summary |
| Question 21: | | | | | | | | |
| 4 studies (4 case-control studies) | Serious | Not serious | Not serious | Not serious | Not serious | ⨁⨁◯◯  LOW | \ | These studies consistently reported that TNC was elevated in the serum of axSpA patients. However, most of these studies did not identify a correlation between serum TNC levels and disease activity or radiographic progression. |

**Table: Studies addressing the value of TNC as a biomarker for diagnosis**

| Author | Year | Design | Population | Result |
| --- | --- | --- | --- | --- |
| Gupta[538] | 2018 | case-control | 36 AS  39 HC | Median serum tenascin C levels were higher in AS [578.5 ng/ml] as compared to healthy controls [32.88 ng/ml, p < 0.0001]；On ROC analysis for active (PhGA ≥ 6) vs. inactive (PhGA ≤ 4) disease, tenascin-C (AUC = 0.60) performed as well as CRP (AUC = 0.65) and ESR (AUC = 0.73). |

**Table: Studies addressing the value of TNC as a biomarker for indicating disease activity**

| Author | Year | Design | Population | Result |
| --- | --- | --- | --- | --- |
| Bubová[537] | 2020 | case-control | 45 axSpA  20 HC | TNC levels did not correlate with disease activity measures (serum CRP or BASDAI). Nevertheless, the weak correlation of TNC levels with different disease stages (r=0.25, p=0.025) was found, with the highest levels in patients with syndesmophytes. |
| Al-Hindawi[539] | 2023 | case-control | 74 axSpA  28 HC | The serum TN-C concentration was significantly increased in patients, especially those on non-TNFi (79 ± 9.6 pg/mL) compared to those on TNFi (69.1 ± 3.2 pg/mL) and control (53 ± 3.9 pg/mL), where (p = 0.003) between patients and controls, while no significant differences (p = 0.21) were found between TNFi and non-TNFi. |
| Hulejova[540] | 2019 | case-control | 61 AS  20 HC | TNC serum levels were elevated in axSpA patients (535.3 (457.7- 677.2) ng/mL) compared to HC (432.1 (329.1-565.9) ng/mL, p= 0.007). TNC serum levels did not correlate with disease activity biomarkers (serum CRP or BASDAI) in patients with axSpA. Although we have not observed correlation between TNC and mSASSS radiographic score, weak correlation with disease subsets was found (r=0.25, p=0.025). |

**Table: Studies addressing the value of TNC as a biomarker for predicting therapeutic effect of bDMARDs**

| Study | Year | Design | Population | Result |
| --- | --- | --- | --- | --- |
| Al-Hindawi[539] | 2023 | case-control | 74 axSpA  28 HC | The serum TN-C concentration was significantly increased in patients, especially those on non-TNFi (79 ± 9.6 pg/mL) compared to those on TNFi (69.1 ± 3.2 pg/mL) and control (53 ± 3.9 pg/mL), where (p = 0.003) between patients and controls, while no significant differences (p = 0.21) were found between TNFi and non-TNFi. |
| Gupta[538] | 2018 | case-control | 36 AS  39 HC | Median serum tenascin C levels were higher in AS [578.5 ng/ml] as compared to healthy controls [32.88 ng/ml, p < 0.0001]；On ROC analysis for active (PhGA ≥ 6) vs. inactive (PhGA ≤ 4) disease, tenascin-C (AUC = 0.60) performed as well as CRP (AUC = 0.65) and ESR (AUC = 0.73). Tenascin C fell levels with treatment [n = 11, 630.8 ng/ml to 376.4 ng/ml p = 0.0006] in treatment responders but not in non-responders [n = 11, 562.3 to 445.6, p = 0.33]. |

**Question 22: Gut microbiota**

We found 6 Cohort and 18 case-control studies addressing this question.

The evidence shows:

1.Regarding the diversity of gut microbiota in axSpA, most of the studies failed to identify changes of a-diversity in axSpA, but a majority of the studies recognized a significant change of β-diversity, indicating a different microbial composition in the gut.[541]

2.Taxonomically, multiple organisms were found to be altered in the gut microbiota in axSpA. Dialister, actinobacteria and clostridium were consistently found to be elevated, while bacteroides seemed to be reduced.

3.Regarding the diagnostic utility of the gut microbiota, different researcher devised different diagnostic panels, with AUC up to 0.95.[542] However, none of these panels were validated by subsequent research.

4.Gut microbiota seemed to be restored after effective treatment.[543] However, correlation between indices of disease activity and gut microbiota was inconsistent and required further studies.

5.Given the current evidence, we decided not to formulate a recommendation for or against the panels of gut microbiota, unless further evidence is put forth and substantiate the robustness of gut microbiota.

The quality of evidence is LOW.

**Table: Evidence profile**

| Certainty assessment | | | | | | | Summary of findings | |
| --- | --- | --- | --- | --- | --- | --- | --- | --- |
| No of participants  (studies)  Follow-up | Risk of bias | Inconsistency | Indirectness | Imprecision | Publication bias | Overall certainty of evidence | Pooled Result (95%CI) | Brief Summary |
| Question 22: | | | | | | | | |
| 24 studies (6 Cohort and 18 case-control studies) | Not serious | Serious | Not serious | Not serious | Serious | ⨁⨁◯◯  LOW | \ | Regarding the diagnostic utility of the gut microbiota, different researcher devised different diagnostic panels, with AUC up to 0.95. Gut microbiota seemed to be restored after effective treatment. However, correlation between indices of disease activity and gut microbiota was inconsistent and required further studies. |

**Table: Studies addressing the value of intestinal flora as a biomarker for diagnosis**

| Study | Year | Design | Sample | Population | Result |
| --- | --- | --- | --- | --- | --- |
| Berlinberg[544] | 2021 | Case-control | Feces | 21 axSpA  27 CD  12 CD-axSpA  24 HC | Alpha diversity analyses of richness and evenness as assessed by Observed, Chao1, Shannon, and Simpson indices were not different between the four subject groups. Beta diversity was assessed by PCoA on the basis of axSpA vs HC using Bray- Curtis dissimilarity and PERMANOVA, and found to have no separation. |
| Breban[545] | 2017 | Case-control | Feces | 87 SpA  28 RA  69 HC | Dysbiosis was evidenced in SpA and RA, as compared with HCs, and was disease specific. A restriction of microbiota biodiversity was detected in both disease groups. The most striking change was a twofold to threefold increased abundance of Ruminococcus gnavus in SpA, as compared with both RA and HCs that was significant in both studies. |
| Cardoneanu[546] | 2020 | Case-control | Feces | 28 AS  20 CD  27 UC  17 IBD+AS  32 HC | Concerning AS patients, significant correlations were observed only for the Bifidobacterium species, significantly increased in the axial form compared to peripheral disease (p=0.035). |
| Cardoneanu[547] | 2021 | Case-control | Feces | 28 AS  32 HC | In cases with AS, a significantly decreased level of C. leptum was observed, associated with an increased level of E. coli. The other analyzed microbial populations did not show significant statistical differences with the control arm. The group of cases with AS also showed a decreased microbial diversity than the control group, but without any statistical value. |
| Costello[548] | 2015 | Case-control | Terminal ileal biopsy | 9 AS  9 HC | Our results show the terminal ileal microbial communities of patients with AS differ significantly (P<0.001) from HC, driven by higher abundance of five families of bacteria Lachnospiraceae (P=0.001), Veillonellaceae (P=0.01), Prevotellaceae (P=0.004), Porphyromonadaceae (P=0.001), and Bacteroidaceae (P=0.001); two of which, Lachnospiracecae, and Prevotellaceace, have been strongly associated with colitis and CD. |
| Chen[542] | 2019 | Case-control | Feces | 41 AS  19 HC | Our results showed that fecal microbial communities in patients with AS differ significantly from those in HCs, driven by a higher abundance of 7 genera (Prevotella_9, Dialister, Comamonas, Collinsella, Streptococcus, Alloprevotella and Prevotella_2) and a lower abundance of 4 genera (Eubacterium_ruminantium_ group, Ruminococcus_gnavus_group, Lachnospira and Bacteroides). In addition, pAS patients were more enriched in Comamonas, Streptococcus and Collinsella, while axAS patients were more enriched in Prevotella_2. An 8 genera-based model showed high accuracy for distinguishing AS patients from HCs with an area under the curve (AUC) up to 0.950. |
| Dai[549] | 2022 | Cohort | Feces | 24 AS  11 HC | Significantly different microbial compositions were observed in samples from ankylosing spondylitis patients compared with healthy controls, characterized by a lower abundance of short-chain fatty acid (SCFA)- producing bacteria. |
| Huang[550] | 2019 | Cohort | Feces | 29 AS  37 HC | Four species were enriched in the patients with AS: Flavonifractor plautii, Oscillibacter, Parabacteroides distasonis and Bacteroides nordii (P<0.05) |
| Klingberg[551] | 2019 | Case-control | Feces | 150 AS  18 HC  17 HC | Compared with HC, fecal microbiota in AS was characterized by a higher abundance of Proteobacteria, Enterobacteriaceae, Bacilli, Streptococcus species, and Actinobacteria, but lower abundance of Bacteroides and Lachnospiraceae. Further, fecal microbiota composition differed between patients with normal (≤ 50 mg/kg, n = 57) and increased (≥ 200 mg/kg, n = 36) fecal calprotectin. |
| Li[552] | 2023 | Case-control | Feces | 193 AS  59 HC | AS patients revealed a significant decrease in gut viral richness and a considerable alteration of the overall viral structure. At the family level, AS patients had an increased abundance of Gratiaviridae and Quimbyviridae and a decreased abundance of Drexlerviridae and Schitoviridae. We trained classification models based on gut viral signatures to discriminate AS patients from healthy controls, with an optimal area under the receiver operator characteristic curve (AUC) up to 0.936, suggesting the clinical potential of the gut virome for diagnosing AS. |
| Liu[553] | 2020 | Case-control | Feces | 10 AS  12 HC | The two research groups did not differ significantly regarding alpha diversity. By comparison to HCs, AS cases displayed a lower relative level of Bacteroidetes (P < 0:05), but a higher level of Firmicutes and Verrucomicrobia (P < 0.05). |
| Min[554] | 2023 | Case-control | Feces | 33 axSpA  20 HC | axSpA patients were found to have decreased a-diversity compared to HCs, indicating that axSpA patients have less diverse microbiomes. In particular, at the species level, Bacteroides and Streptococcus were more abundant in axSpA patients than in HCs, whereas Faecalibacterium (F). prausnitzii, a butyrate-producing bacteria, was more abundant in HCs. |
| Sternes[555] | 2022 | Case-control | Feces +  Intestinal biopsy | 185 AS  59 IBD  105 HC | Focusing on the Australian cohort, AS, AS‐IBD and IBD patients differed from one another and from healthy controls in both alpha and beta diversity. AS patients with and without clinical IBD could be distinguished from one another with moderate accuracy using stool microbiome (AUC=0.754). |
| Thompson[556] | 2023 | Case-control | Feces | 67 axSpA  119 RA  35 PsA  54 NIJP  165 HC | We identified several taxa associated with inflammation, diagnosis, and anemia or more disease-specific markers of inflammation (e.g., BASDAI or DAS28-CRP) that paralleled changes previously observed in dysbiotic individuals with IBD, including the clades Streptococcus sp., Escherichia coli, and R. gnavus. |
| Wen[557] | 2017 | Case-control | Feces | 97 AS  114 HC | The ankylosing spondylitis patients demonstrated increases in the abundance of Prevotella melaninogenica, Prevotella copri, and Prevotella sp. C561 and decreases in Bacteroides spp. It is noteworthy that the Bifidobacterium genus, which is commonly used in probiotics, accumulated in the ankylosing spondylitis patients. Diagnostic algorithms were established using a subset of these gut microbial biomarkers. From the ROC curves, we found that the gene markers (AUC = 96.64% in the validation cohort) were better than the sequenced reference genome markers (AUC = 93.55% in the validation cohort). |
| Zhang[558] | 2019 | Case-control | Feces | 103 AS  104 HC | Alpha diversity was not significant difference in AS compared with HCs. For the community structure, Bacteroidetes was the most represented class. Megamonas, Dorea, and Blautia were significantly greater in AS than in HCs, whereas the abundance of Lachnospira, Ruminococcus, and Clostridium_XlVb was significantly lower in AS than in HCs. |
| Zhang[559] | 2020 | Case-control | Feces | 20 AS  20 HC | The increased relative abundance of microbiota in AS nonsmokers was g_Comamonas and g_Desulfovibrio, while that in AS smokers was g_Actinomyces, g_Collinsella, g_Lachnospiraceae_UCG-008, and g_Paraprevotella. The relative abundance of gut microbiota showed dynamic variation. |
| Zhou[560] | 2019 | Case-control | Feces | 85 AS  62 HC | We identified AS-enriched species including Bacteroides coprophilus, Parabacteroides distasonis, Eubacterium siraeum, Acidaminococcus fermentans and Prevotella copri. The area under the receiver operating curve (AUC) was 89.7% (CI 83.7%–95.6%) and 82.6% (CI 68.5%–96.6%) in training and testing group, respectively. |

**Table: Studies addressing the value of intestinal flora as a biomarker for indicating disease activity**

| Study | Year | Design | Sample | Population | Result |
| --- | --- | --- | --- | --- | --- |
| Berland[561] | 2023 | Case-control | Feces | 102 SpA  63 HC | The restriction of microbiota diversity was detected in patients with the most active disease, and the abundance of several bacterial species was correlated with Bath Ankylosing Spondylitis Disease Activity Index score. We highlighted a decreased abundance of several species of bacteria in SpA patients, especially those bacteria belonging to the Clostridiales order. Among the few species of bacteria showing increased abundance, Ruminococcus gnavus was one of the top differentiating species. |
| Breban[545] | 2017 | Case-control | Feces | 87 SpA  28 RA  69 HC | There was a striking correlation between R. gnavus abundance and BASDAI in the subgroup of patients having IBD history (p<0.005, r=0.77) but not in the others. |
| Cardoneanu[546] | 2020 | Case-control | Feces | 28 AS  20 CD  27 UC  17 IBD+AS  32 HC | In the group of AS cases, no significant correlations were observed between BASDAI and BASFI and the bacterial groups. BASDAI score was inversely correlated with the total bacterial group (p=0.010, r=-0.606). In addition, the BASFI score correlated with all bacteria (p=0.001, r=-0.764), F. prausnitzii (p=0.010, r = 0.606), Bifidobacterium (p=0.016, r = 0.575), Lactobacillus (p=0,001, r=0,843) and E. coli (p=0.016, r = 0.575). |
| Cardoneanu[547] | 2021 | Case-control | Feces | 28 AS  32 HC | ESR and CRP were inversely correlated with the level of Bacteroides and directly proportional to C. coccoides and C. leptum. Using the Spearman correlations, we aimed to ascertain whether there were correlations between the disease activity quantified by BASDAI and BASFI scores and the bacterial populations. We did not find statistically significant data between these scores and the bacteria species. |
| Dai[549] | 2022 | Cohort | Feces | 24 AS  11 HC | SCFA-producing bacteria described above (i.e. Megamonas and Lachnoclostridium genera), which were restored in post-treatment group, were significantly negatively correlated with the BASDAI score. The abundance of Megamonas may have a stronger effect than that of Lachnoclostridium, consistent with their association with the CRP score. Intriguingly, Haemophilus was positively associated with disease activity. |
| Klingberg[551] | 2019 | Case-control | Feces | 150 AS  18 HC  17 HC | No association was found between the fecal microbiota composition and HLAB27 status, disease activity, function, or medication. |
| Liu[553] | 2020 | Case-control | Feces | 10 AS  12 HC | At the phylum level, Firmicutes and Verrucomicrobia had a positive association with ESR and CRP, while Bacteroidetes exhibited an inverse correlation with ESR and CRP. Meanwhile, in terms of genus, Bacteroides had a positive association with ESR and CRP, whereas Ruminococcus and Parasutterella had an inverse correlation with ESR and CRP, and Helicobacter also displayed an inverse correlation with CRP. |
| Sternes[555] | 2022 | Case-control | Feces +  Intestinal biopsy | 185 AS  59 IBD  105 HC | Microbiome composi‐ tion was correlated with disease activity measured by BASDAI and faecal calprotectin (FCP) levels. Enrichment of potentially pathogenic Streptococcus was noted in AS, AS‐IBD and IBD patients. Furthermore, enrichment of another potentially pathogenic genus, Haemophilus, was observed in AS, AS‐IBD, IBD, AS patients with increased BASDAI, and IBD patients with faecal calprotectin >100 μg/mg. |
| Tito[562] | 2016 | Case-control | ileal and colonic biopsies | 27 SpA  15 HC | We revealed a positive correlation between the abundance of the genus Dialister and the Ankylosing Spondylitis Disease Activity Score (ASDAS) (correlation coefficient rho = 0.62, FDR-corrected q-value < 0.01). This finding was further supported by the low frequency of Dialister observed in non-inflamed SpA samples and healthy controls. |
| You[563] | 2021 | Case-control | Feces | 40 AS  20 HC | The relative abundances of opportunistic pathogens Ruminococcus_2, Ruminococcaceae_ UCG_014, especially, Clostridium_sensu_stricto_1 increased notably in the high score ASDAS group. It is the first time proving that Clostridium_sensu_stricto_1 is related to AS with higher ASDAS scores. |
| Zhang[558] | 2019 | Cohort | Feces | 103 AS  104 HC | BASDAI, BASFI and ASDAS-CRP were not correlated with bacterium at the phylum level (P > 0.05). At the class level, Bacilli, belongs to Firmicutes, was positively correlated with BASDAI (r = 0.2705, P = 0.0140). |
| Zhang[564] | 2020 | Cohort | Feces | 78 AS  19 HC | BASDAI positively correlated with the relative abundance of g_Escherichina-Shigella and g_Klebsiella, but negatively correlated with f_Lachnospiraraceae at baseline. (r=0.544, P=0.013, r=0.509, P=0.022 and r=-0.577, P=0.008, respectively). |
| Zhou[560] | 2019 | Case-control | Feces | 85 AS  62 HC | The relative abundance of AS-enriched Acidaminococcus fermentans VR4_DSM_20731 was positively related to BASDAI, while AS-enriched Parabacteroides distasonis ATCC_8503 was negatively related to them. |

**Table: Studies addressing the value of intestinal flora as a biomarker for predicting radiological progression**

| Study | Year | Design | Sample | Population | Result |
| --- | --- | --- | --- | --- | --- |
| Cardoneanu[547] | 2021 | Case-control | Feces | 28 AS  32 HC | No correlations were found between the degree of radiological sacroiliitis and bacterial groups (P=0.053, Kruskal‐Wallis test) or between BMI and bacterial populations (P=0.366). |

**Table: Studies addressing the value of intestinal flora as a biomarker for predicting therapeutic effect of bDMARDs**

| Study | Year | Design | Sample | Population | Result |
| --- | --- | --- | --- | --- | --- |
| Berland[561] | 2023 | Case-control | Feces | 102 SpA  63 HC | A negative correlation between BASDAI score and MSP richness was observed (Spearman’s rho = –0.32; P = 0.007) for patients receiving treatment with at least 1 drug, and this negative correlation remained significant for patients not receiving treatment (Spearman’s rho = –0.35; P = 0.049). Overall, these results suggest that the SpA microbiome signature exists independently of the treatment patients receive. |
| Chen[565] | 2021 | Cohort | Feces | 30 AS  24 HC | The microbiome was restored remarkably after 6 months of adalimumab therapy in AS patients. We then compared the baseline gut microbiome of 22 adalimumab responders with 8 non-responders, a higher abundance of Comamonas was revealed in the latter, although no statistical difference was found after adjusting for the false discovery rate. |
| Dai[549] | 2022 | Cohort | Feces | 24 AS  11 HC | All patients exhibited a positive response after anti-TNF-α treatment, accompanied by  a trend of restoration in the microbiota compositions and functional profile of ankylosing spondylitis patients to healthy controls. In particular, the abundance of SCFA-producing bacteria (e.g. Megamonsa and Lachnoclostridium) was significantly lower in ankylosing spondylitis patients than in healthy controls and restored after anti-TNF-α treatment. |
| Huang[550] | 2019 | Cohort | Feces | 29 AS  37 HC | Only F. plautii was found to be significantly changed. No additional species were found in the HC vs. Chinese herbal medicine (CHM) analysis, which indicated a beneficial effect of CHM in removing the other three strains. F. plautii was found to be significantly increased in the comparison between the HC and WM (NSAIDs) groups, along with four other species (Clostridium bolteae, Clostridiales bacterium 1_7_47FAA, C. asparagiforme and C. hathewayi). |
| Klingberg[551] | 2019 | Case-control | Feces | 150 AS  18 HC  17 HC | No association was found between the fecal microbiota composition and HLAB27 status, disease activity, function, or medication. |
| Yin[566] | 2019 | Case-control cohort | Feces | 127 AS  123 HC | TnFi therapy largely restored the perturbed microbiome observed in untreated as cases to that of healthy controls, including several important bacterial species that have been previously associated with as and other related diseases. TnFi therapy of patients with as was also associated with a reduction of potentially arthritogenic bacterial peptides, relative to untreated patients. |
| Zhang[559] | 2020 | Cohort | Feces | 20 AS  20 HC | The improvement rate of ASDAS in AS nonsmokers was higher than that in AS smokers (2.297 vs 1.736) after anti-TNF-α treatment. The β-diversity of gut microbiota in AS smokers was lower than that in AS nonsmokers and improved with treatment. |
| Zhang[564] | 2020 | Cohort | Feces | 78 AS  19 HC | We found that the relative abundance of microbiota in AS patients treated with anti-TNF-α differed at various time points and distinguished 4 groups: the higher and lower than healthy control (HC) level groups throughout the study and the unchanged and restored to HC levels groups. The characteristic increases of microbes in AS patients were f_Prevotellaceae and f_Actinomycetaceae. In HC, the characteristic increase was f_Lachnospiraceae. The beta-diversity of microbiota in AS at baseline was lower than HC at the same level (P<0.01) and restored to normal values one month after treatment. |

**Question 23: Metabonomics signature**

We found 3 cohort and 16 case-control studies addressing this question.

The evidence shows:

1.Different samples could be used in the metabolomics analysis, including serum, urine and even fecal samples. Metabolomics studies in axSpA patients have identified significant alterations in the metabolism of amino acids, fatty acids, glucose and choline.

2.It was consistently reported that amino acids, notably tryptophan, proline and glutamic acid, were altered in axSpA. Analysis also showed active metabolism of glucose and fatty acids, which could be subsequent to the inflammatory status of axSpA.

3.Different researchers put forth diagnostic panels comprised of different metabolites, with AUC as high as 0.998. Many of these metabolites were also associated with disease activity.[567] However, none of these panels were repeatedly verified by subsequent studies.

4.Based on the current studies, we decided that there was not enough evidence to endorse a single panel for diagnosis or evaluation of disease activity, but it shall be revisited once more evidence is accumulated.

The quality of evidence is LOW.

**Table: Evidence profile**

| Certainty assessment | | | | | | | Summary of findings | |
| --- | --- | --- | --- | --- | --- | --- | --- | --- |
| No of participants  (studies)  Follow-up | Risk of bias | Inconsistency | Indirectness | Imprecision | Publication bias | Overall certainty of evidence | Pooled Result (95%CI) | Brief Summary |
| Question 23: | | | | | | | | |
| 19 studies (3 cohort and 16 case-control studies) | Not Serious | Not serious | Not serious | Not serious | Not serious | ⨁⨁◯◯  LOW | \ | It was consistently reported that amino acids, notably tryptophan, proline and glutamic acid, were altered in axSpA. Analysis also showed active metabolism of glucose and fatty acids, which could be subsequent to the inflammatory status of axSpA. Many of these metabolites were also associated with disease activity. |

**Table: Studies addressing the value of metabolomics for diagnosis**

| Study | Year | Design | Sample | Population | Result |
| --- | --- | --- | --- | --- | --- |
| Berlinberg[544] | 2021 | Case-control | Colon biopsy sample | 21 axSpA  27 CD  12 CD- axSpA  24 HC | We identified significant alterations in tryptophan pathway metabolites, including an expansion of indole-3-acetate (IAA) in axSpA and CD-axSpA compared to HC and CD and indole-3-acetaldehyde (I3Ald) in axSpA and CD-axSpA but not CD compared to HC, suggesting possible specificity to the development of axSpA. |
| Chen[568] | 2014 | Case-control | Serum | 33 AS  33 HC | The results showed that significant differences in most of the FFA (C12:0, C16:0, C16:1, C18:3, C20:4, C20:5, C22:5 and C22:6) and EFA (C12:0, C16:1, C18:0, C18:1, C18:2, C18:3, C20:4 and C22:6) concentrations were found between the AS patients and healthy controls (p < 0.05). FFAs C20:4, C12:0, C18:3 and EFAs C22:6, C12:0 were confirmed as potential biomarkers to identify AS patients and healthy controls. (VIP>1.5) |
| Dogan[569] | 2022 | Case-control | Serum | 18 AS  20 HC | Down-regulations were observed in phosphatidylcholine (PC) (16:0/0:0), beta-D-Fructose, stearic acid, trimipramine N-Oxide and muconic acid, and up-regulation were detected in PC (18:2/0:0), 3-Methylindole, palmitic acid (PA), alpha-Tocotrienol, and beta-D-glucopyranoside in active AS patients compared to the healthy control subjects. |
| Fischer[570] | 2011 | Case-control | Serum | 18 AS  9 HC | One molecular feature identified as a Vitamin D3 metabolite—(23S,25R)-25-hydroxyvitamin D3 26,23-peroxylactone—was down-regulated in AS. The ratio of this vitamin D metabolite versus vitamin D binding protein serum levels was also altered in AS compared with controls. |
| Gao[571] | 2009 | Case-control | Serum | 15 AS  24 HC | AS patients presented elevated plasma concentrations of proline, glucose, phosphate, urea, glycerol, phenylalanine and homocysteine but reduced levels of phosphocholines, tryptophan and a bipeptide – phenylalanyl-phenylalanine. |
| He[572] | 2019 | Case-control | Feces | 49 AS  38 HC | Male-specific fecal signatures in AS patients were steroid compounds, including cholestan-3-ol, tocopherol, stigmastan-3,5-diene, cholest-3-ene, cholest-4-en-6-one and 1-heptatriacotanol. Female-specific fecal signatures were ergost-5-en-3-ol, acetate and D-myo-Inositol. |
| Jiang[573] | 2013 | Case-control | Serum | 27 AS  33 GA  27 OA  27 RA  60 HC | Differentially expressed metabolites in AS and GA include dihydrothymine, alloxanoic acid, uric acid, 5-oxoproline, valine, creatine, arabitol, succinic acid, taurine, sucrose, lysine, citrulline, sarcrosine, valine, malic acid, alanine and cysteine. The area under the curve (AUC) reached 0.88 with a sensitivity of 79% and specificity of 85%. |
| Li[574] | 2022 | Case-control | Serum | 20 AS  20 HC | A diagnostic model comprising nine metabolites (cysteinylglycine disulfide, choline, N6, N6, N6-trimethyllysine, histidine, sphingosine, fibrinopeptide A, glycerol 3-phosphate, 1-linoleoyl-GPA (18:2), and fibrinopeptide A (3–16)) was generated using LASSO regression, capable of distinguishing HCs from AS with a high AUC of 1. |
| Lv[575] | 2021 | Case-control | Saliva | 37 AS  41 HC | With an area under the curve (AUC) threshold of 0.8, 9 potential biomarkers were observed; these included one serum cytokine (IL-6, AUC=0.84) and eight salivary metabolites: 5-aminovaleric acid (AUC=0.89), D-rhamnose (AUC = 0.85), glycine (AUC = 0.84), L-ornithine (AUC = 0.82), L-lysine (AUC = 0.82), L-alanine (AUC = 0.81), D-galactose (AUC = 0.80), and L-proline (AUC = 0.80). Among these metabolites, the combination of 5-aminovaleric acid and phoshorylethanolamine produced an AUC of 0.928 |
| Onmaz[576] | 2021 | Case-control | Serum | 85 AS  50 HC | Serum tryptophan, kynurenic acid, 3-hydroxykynurenine levels were significantly decreased (p < 0.05) in both AS groups compared to the control group, while the levels of kynurenine, quinolinic acid, CRP, ESR, and IL-6 were higher (p < 0.05). |
| Ou[567] | 2021 | Cohort | Serum | 32 AS  40 HC | We generated a diagnostic panel comprising five metabolites (L-glutamate, arachidonic acid, L-phenylalanine, PC (18:1(9Z)/18:1(9Z)), 1- palmitoylglycerol), capable of distinguishing HCs from AS with a high AUC of 0.998, (95%CI: 0.992–1.000). |
| Shao[577] | 2016 | Case-control | Feces | 40 AS  35 RA  34 HC | Significant differences in the fecal metabolic profiles could distinguish AS/RA patients from healthy controls but could not distinguish between AS and RA patients. The significantly decreased metabolites in AS/RA patients were butyrate, propionate, methionine, and hypoxanthine. Significantly increased metabolites in AS/RA patients were taurine, methanol, fumarate, and tryptophan. |
| Stoll[578] | 2016 | Case-control | Feces | 14 ERA  9 HC;  10 ERA  10 HC | The pathway for tryptophan metabolism was represented among both the positively and negative charged ions as being decreased in JIA/ ERA patients. Some of the other findings, although not internally validated, confirm previous reports in animal models or patients with IBD, such as decreased butyrate and compounds associated with purine metabolism. |
| Wang[579] | 2016 | Case-control | Plasma, urine and ligament tissue | 44 AS  44 HC | On the basis of correlation coefficients, VIP values and P values of the metabolites obtained from the multivariate analysis, we selected 20 metabolites from plasma (n = 13), urine (n = 7) and tissue (n = 2) as potential biomarkers for AS. These metabolites were associated mainly with metabolic pathways such as fat metabolism, intestinal microbial metabolism, glucose metabolism and choline metabolism, as well as probably with immune regulation. |
| Zhou[580] | 2020 | Case-control | Serum | 30 AS  32 RA  30 HC | A total of 29 amino acids and biogenic amines were detected in all participants by UPLC-TQ-MS. It showed significant amino acid differences between the AS/RA patients and control subjects. Additionally, 4-hydroxy-L-proline, alanine, γ- aminobutyric acid, glutamine, and taurine were identified as candidate markers shared by AS/RA groups. Especially, lysine and glycine were special markers for AS and RA, respectively. |
| Du[581] | 2023 | Case-control | Serum  Urine | 36 AS  186 RA  84 SLE  39 SS  12 SSc  6 CTD  186 HC | The AS vs. SS vs. SLE vs. RA vs. HC model was assessed. RF achieved superior results in both urine and serum samples with an AUC of 0.998 and accuracy of 96.1%, and an AUC of 0.974 and accuracy of 83.3%, respectively. In the fusion model, NN exhibited superior classification results with an AUC of 0.999 and accuracy of 97.2%. |

**Table: Studies addressing the value of metabolomics for indicating disease activity**

| Study | Year | Design | Sample | Population | Result |
| --- | --- | --- | --- | --- | --- |
| Dogan[569] | 2022 | Case-control | Serum | 18 AS  20 HC | CRP was statistically significant correlated with PC (16:0/0:0) (p = 0.035, r =0.498) and BASFI was statistically significant correlated with PC (18:2/0:0) (p = 0.048, r = 0.471). |
| Onmaz[582] | 2021 | Cohort | Serum | 60 AS  60 HC | It was found that total methylated arginine load significantly increased in patients with AS (p < 0.001), and the Arg/ADMA ratio was positively correlated with HDL levels and negatively correlated with glucose, ESR, total cholesterol, triglyceride, and LDL levels. |
| Shao[577] | 2016 | Case-control | Feces | 40 AS  35 RA  34 HC | Proline, a-ketoisocaproate, asparagine and inosine had a remarkable correlation with ESR, isoleucine, choline, and methanol; UDP-glucose and tryptophan were remarkably correlated with CRP. |
| Zhou[580] | 2020 | Case-control | Serum | 30 AS  32 RA  30 HC | The generalized linear regression analysis showed that tyrosine, serine, lysine, proline, γ- aminobutyric acid, alanine, and acetyl-carnitine were related to the AS activity (P < 0.05). Furthermore, based on VIF < 10, the results finally presented that lysine, proline, serine, and alanine were related to disease activity (BASDAI, ARDAS-CRP, and BASFI) of AS. |

**Table: Studies addressing the value of metabolomics for predicting radiological progression**

| Study | Year | Design | Sample | Population | Result |
| --- | --- | --- | --- | --- | --- |
| Zhang[583] | 2023 | Case-control | Serum | 18 AS  20 HC | Serum hypoxanthine and xanthine levels were significantly increased in patients with AS, suggesting that purine metabolites may be involved in the radiological progression of AS. Purine metabolites, along with PTHrP derived from SHP2-deficient chondrocytes, accelerated the growth of chondrocytes and ectopic new bone formation through PKA/CREB signaling. |

**Table: Studies addressing the value of metabolomics for predicting therapeutic effect of bDMARDs**

| Study | Year | Design | Sample | Population | Result |
| --- | --- | --- | --- | --- | --- |
| Bogunia-Kubik[584] | 2021 | Cohort | Serum | 29 AS  26 RA  23 PsA | ANOVA or Kruskal–Wallis tests identified seven metabolites, and two unknown signals were determined to be significant for the comparison of RA, AS, and PsA after 6M. The AS entity exhibited the most prominent increase in the levels of ethanol and glutamate. Moreover, acetate and sn-3GP exhibited differentiating possibilities only between AS and PsA diseases. |
| Onmaz[576] | 2021 | Case-control | Serum | 85 AS  50 HC | The Kynurenine/Tryptophan ratio and CRP levels of the conventional therapy and anti-TNF therapy group were significantly lower than the newly diagnosed AS patients (p < 0.05). |
| Onmaz[582] | 2021 | Cohort | Serum | 60 AS  60 HC | Serum ADMA, SDMA, total methylated arginine load, and CRP levels were lower (p < 0.05) in the TNF-α group compared to the conventional treatment group. |
| Ou[567] | 2021 | Cohort | Serum | 32 AS  40 HC | We aimed to identify potential metabolite biomarkers to predict response to TNF inhibitors. The patients were divided into TNF inhibitors sensitive (n=21) and resistant (n=11) groups according to the Assessment of Spondylarthritis international Society (ASAS) Response Criteria for a 20% improvement (ASAS20). However, the PCA did not show discrimination among the two groups and the OPLS-DA models were overfitted. The results suggested that serum metabolomics might not be able to predict responses to TNF inhibitors in AS patients. |

**Question 24: NSAIDs-related genes**

We found 14 case-control studies addressing this question.

The evidence shows:

1.The gene CYP2C9 is involved in the metabolism of multiple NSAIDs, including celecoxib, meloxicam and diclofenac. Mutations of CYP2C9 include CYP2C9*2 and CYP2C9*3, which are associated with reduced rate of metabolism of such NSAIDs.[585]

2.Our meta-analysis confirmed that the mutations CYP2C9*2 and CYP2C9*3 are associated with an increased risk of gastrointestinal adverse effects (OR=1.35, 95% CI [1.06, 1.72]), especially upper gastrointestinal bleeding.

3.The systemic literature review also turned up other genes that might be connected to deregulated metabolism of NSAIDs, such as PTGS2 with encodes COX-2.[586] However, studies failed to prove its association with NSAIDs safety or efficacy.

The quality of evidence is LOW.

**Table: Evidence profile**

| Certainty assessment | | | | | | | Summary of findings | |
| --- | --- | --- | --- | --- | --- | --- | --- | --- |
| No of participants  (studies)  Follow-up | Risk of bias | Inconsistency | Indirectness | Imprecision | Publication bias | Overall certainty of evidence | Pooled Result (95%CI) | Brief Summary |
| Question 24: | | | | | | | | |
| 14 studies (14 case-control studies) | Not serious | Not serious | Serious | Serious | Not serious | ⨁⨁◯◯  LOW | \ | the mutations CYP2C9*2 and CYP2C9*3 are associated with an increased risk of gastrointestinal adverse effects (OR=1.35, 95% CI [1.06, 1.72]), especially upper gastrointestinal bleeding. The systemic literature review also turned up other genes that might be connected to deregulated metabolism of NSAIDs, such as PTGS2 with encodes COX-2. |

**Table: Studies addressing the risk of gastrointestinal adverse reactions after taking NSAIDs in CYP2C9*2 or CYP2C9*3 carrier**

| Study | Year | Design | Diagnosis of cases | Ethnicity | Number of case (CYP2C9*2 or CYP2C9*3 carrier) | Number of gastrointestinal adverse reactions in cases | Number of control (CYP2C9*1/*1) | Number of gastrointestinal adverse reactions in controls |
| --- | --- | --- | --- | --- | --- | --- | --- | --- |
| Forgerini[587] | 2023 | Case-control | UGIB | Latin American | 28 | 11 | 50 | 20 |
| Figueiras[585] | 2016 | Case-control | UGIB | European | 577 | 217 | 1343 | 530 |
| Ishihara[588] | 2014 | Case-control | Small intestinal injury | Asian | 7 | 2 | 422 | 18 |
| Musumba[589] | 2012 | Case-control | Peptic ulcer | European | 485 | 170 | 123 | 39 |
| Carbonell[590] | 2010 | Case-control | UGIB | European | 57 | 33 | 263 | 100 |
| Blanco[591] | 2008 | Case-control | UGIB | European | 50 | 36 | 66 | 30 |
| Ma[592] | 2008 | Cross-sectional | Gastropathy | Asian | 52 | 1 | 57 | 0 |
| Pilotto[593] | 2007 | Case-control | UGIB | European | 26 | 18 | 52 | 15 |
| Vonkeman[594] | 2006 | Case-control | Peptic ulcer | European | 26 | 9 | 87 | 31 |
| Martinez[595] | 2004 | Case-control | UGIB | European | 94 | 53 | 124 | 51 |
| Martin[596] | 2001 | Case-control | Peptic ulcer | European | 23 | 7 | 32 | 13 |


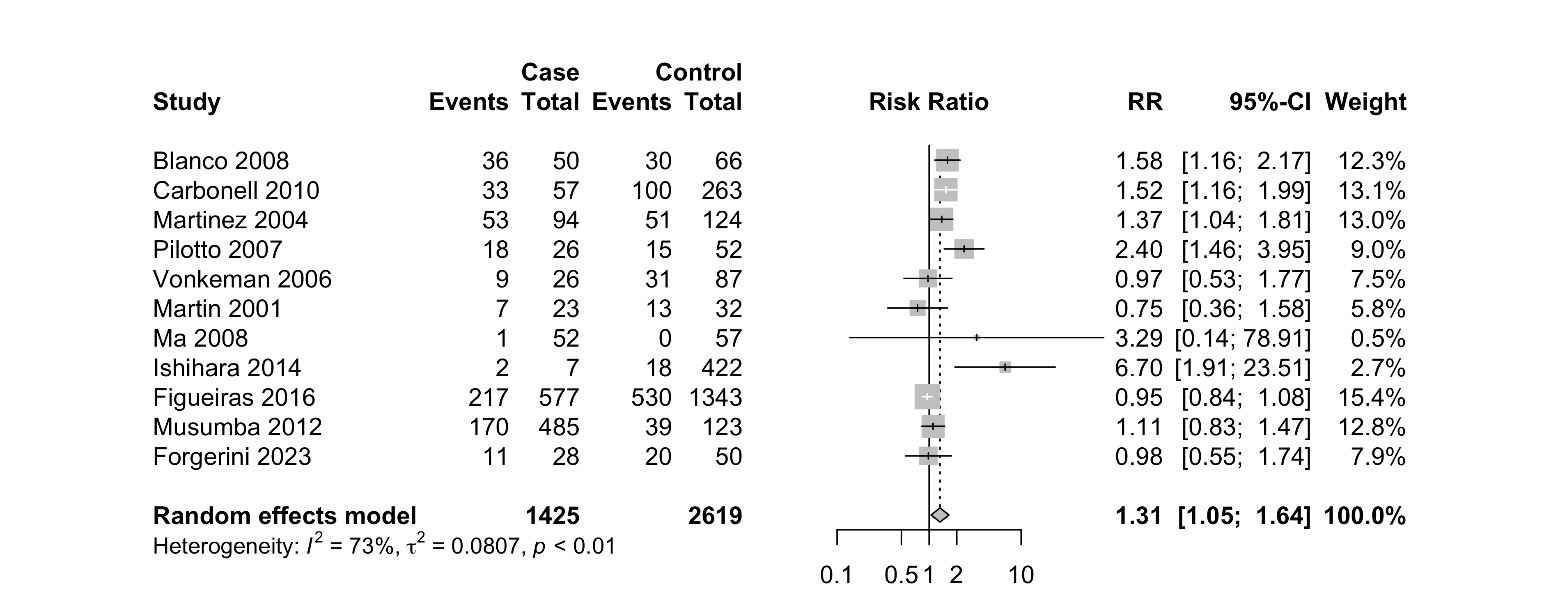
**Figure: Forest Plot: the risk of gastrointestinal adverse reactions after taking NSAIDs in CYP2C9*2 or CYP2C9*3 carrier**


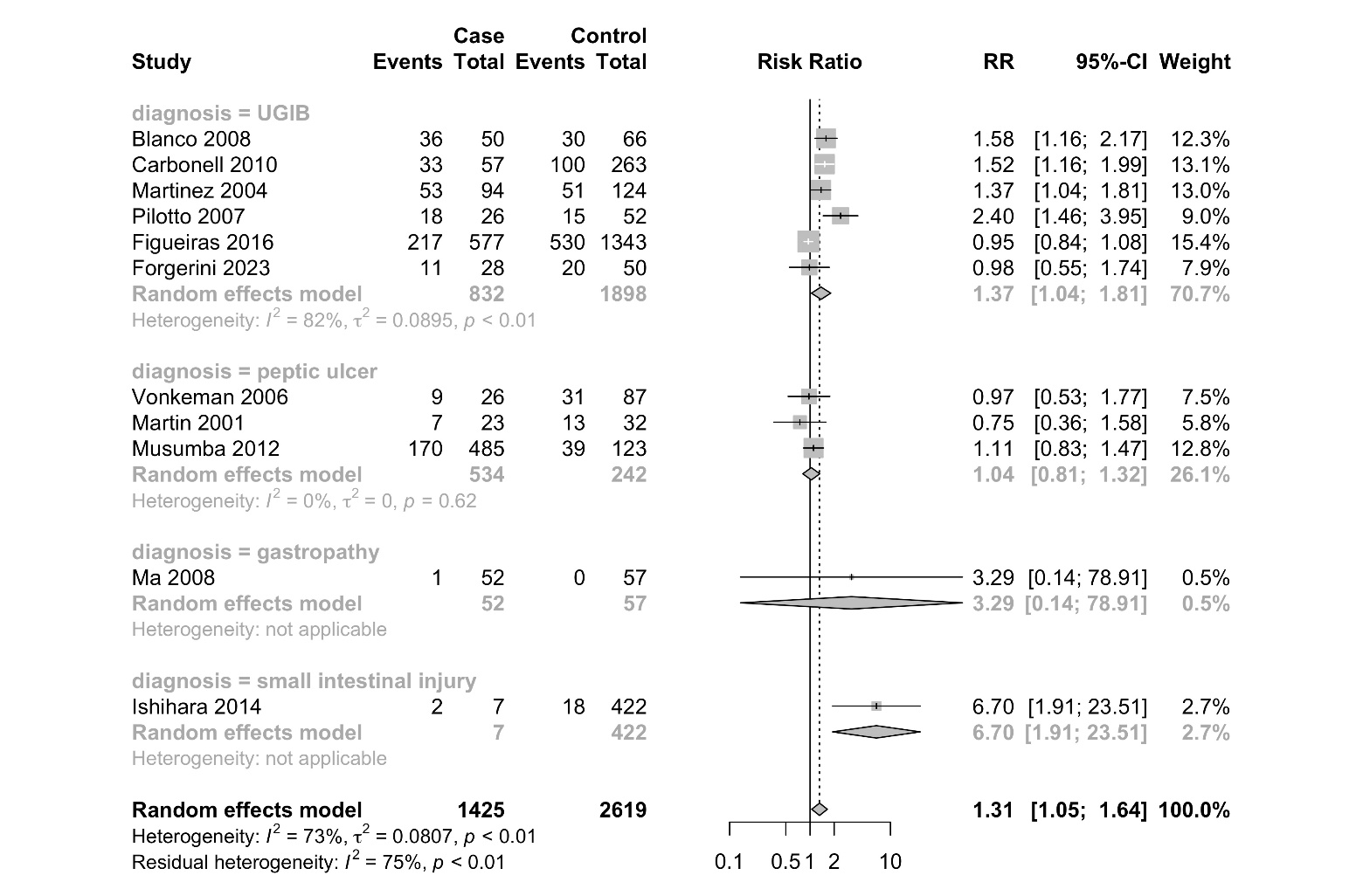
**Figure: Forest Plot: the risk of different gastrointestinal adverse reactions after taking NSAIDs in CYP2C9*2 or CYP2C9*3 carrier**


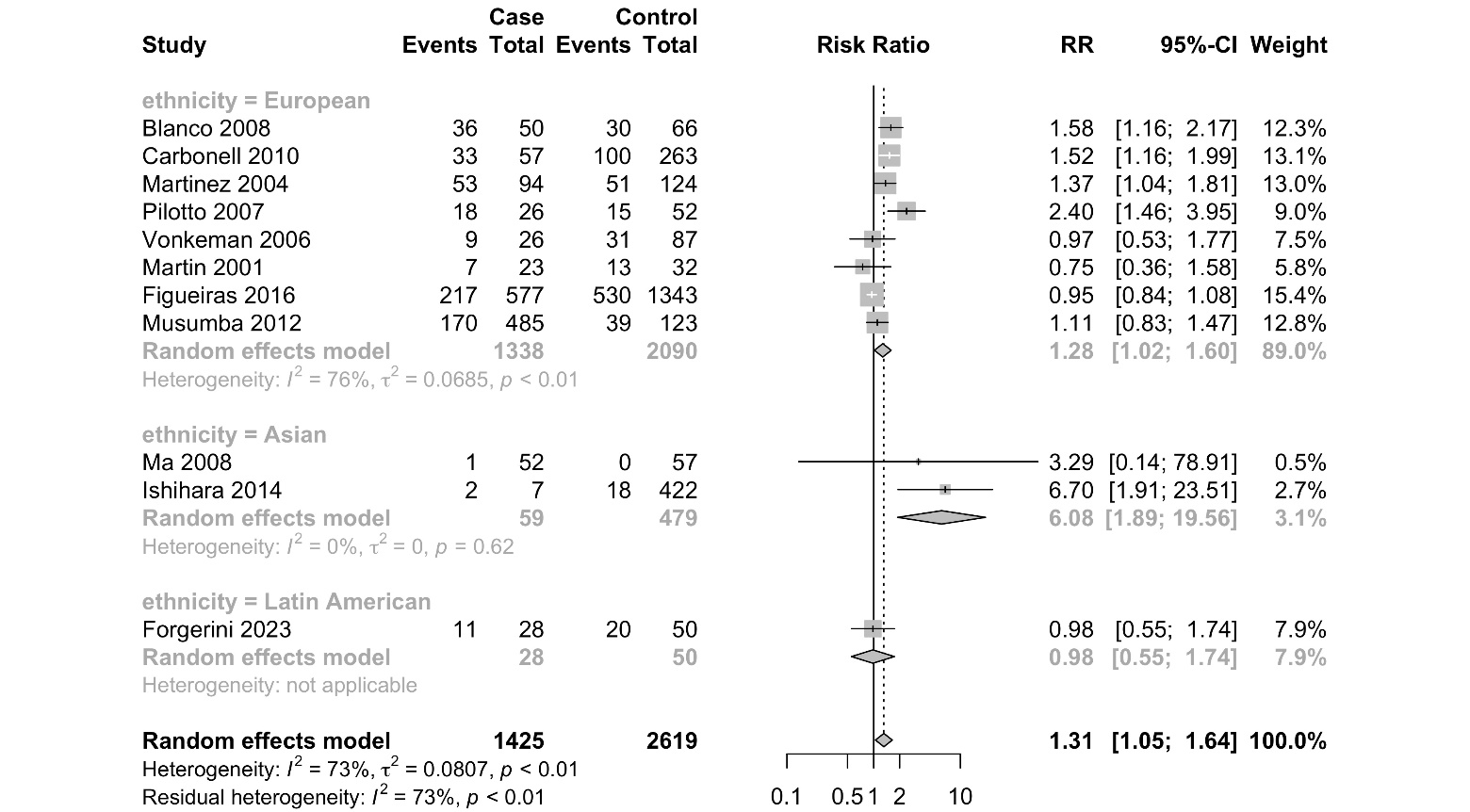


**Figure: Forest Plot: the risk of gastrointestinal adverse reactions after taking NSAIDs in CYP2C9*2 or CYP2C9*3 carrier of different ethnicity**

**Table: Studies addressing the risk of gastrointestinal adverse reactions after taking NSAIDs in CYP2C9*2 carrier**

| Study | Year | Design | Diagnosis of cases | Ethnicity | Number of case (CYP2C9*2 carrier) | Number of gastrointestinal adverse reactions in cases | Number of control (CYP2C9*1/*1) | Number of gastrointestinal adverse reactions in controls |
| --- | --- | --- | --- | --- | --- | --- | --- | --- |
| Forgerini[587] | 2023 | Case-control | UGIB | Latin American | 28 | 8 | 50 | 8 |
| Figueiras[585] | 2016 | Case-control | UGIB | European | 577 | 129 | 1343 | 353 |
| Ishihara[588] | 2014 | Case-control | Small intestinal injury | Asian | 7 | 0 | 422 | 0 |
| Musumba[589] | 2012 | Case-control | Peptic ulcer | European | 485 | 108 | 123 | 24 |
| Carbonell[590] | 2010 | Case-control | UGIB | European | 57 | 14 | 263 | 58 |
| Blanco[591] | 2008 | Case-control | UGIB | European | 50 | 24 | 66 | 17 |
| Ma[592] | 2008 | Cross-sectional | Gastropathy | Asian | 52 | 1 | 57 | 0 |
| Pilotto[593] | 2007 | Case-control | UGIB | European | 26 | 18 | 52 | 10 |
| Vonkeman[594] | 2006 | Case-control | Peptic ulcer | European | 26 | 7 | 87 | 17 |
| Martinez[595] | 2004 | Case-control | UGIB | European | 94 | 38 | 124 | 32 |
| Martin[596] | 2001 | Case-control | Peptic ulcer | European | 23 | 4 | 32 | 9 |


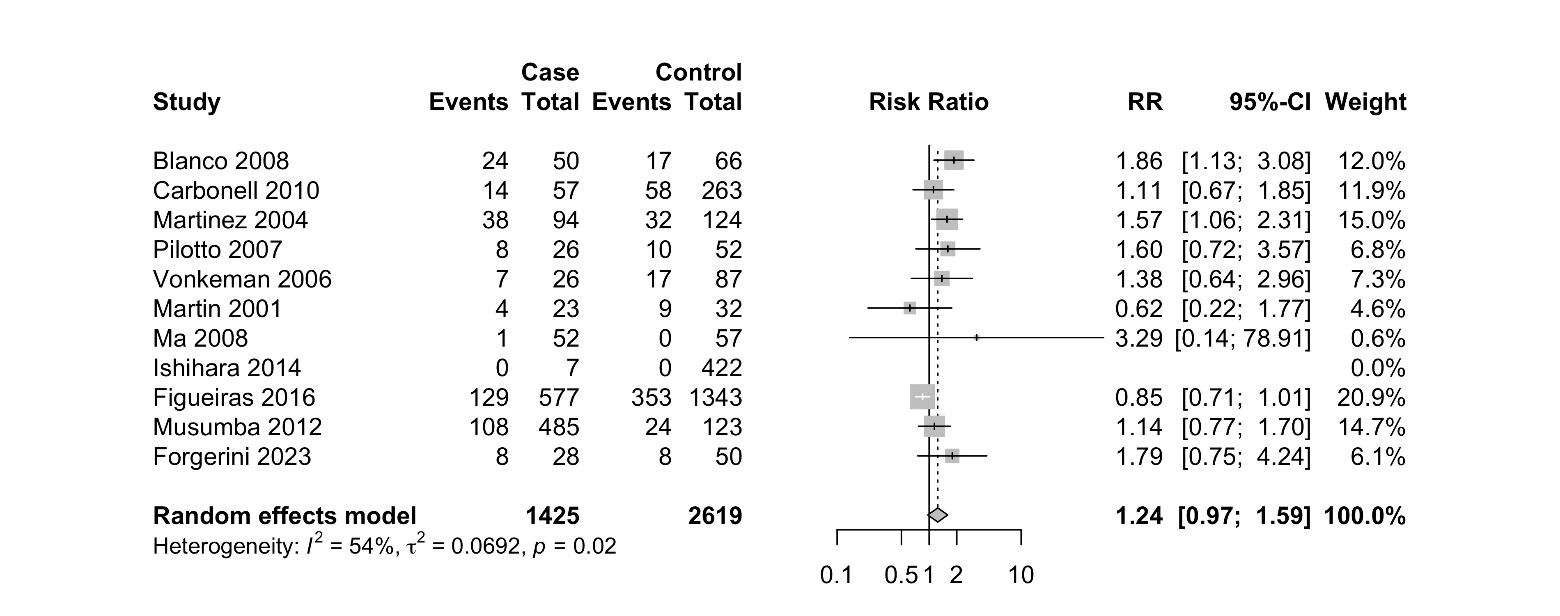


**Figure: Forest Plot: the risk of gastrointestinal adverse reactions after taking NSAIDs in CYP2C9*2 carrier**

**
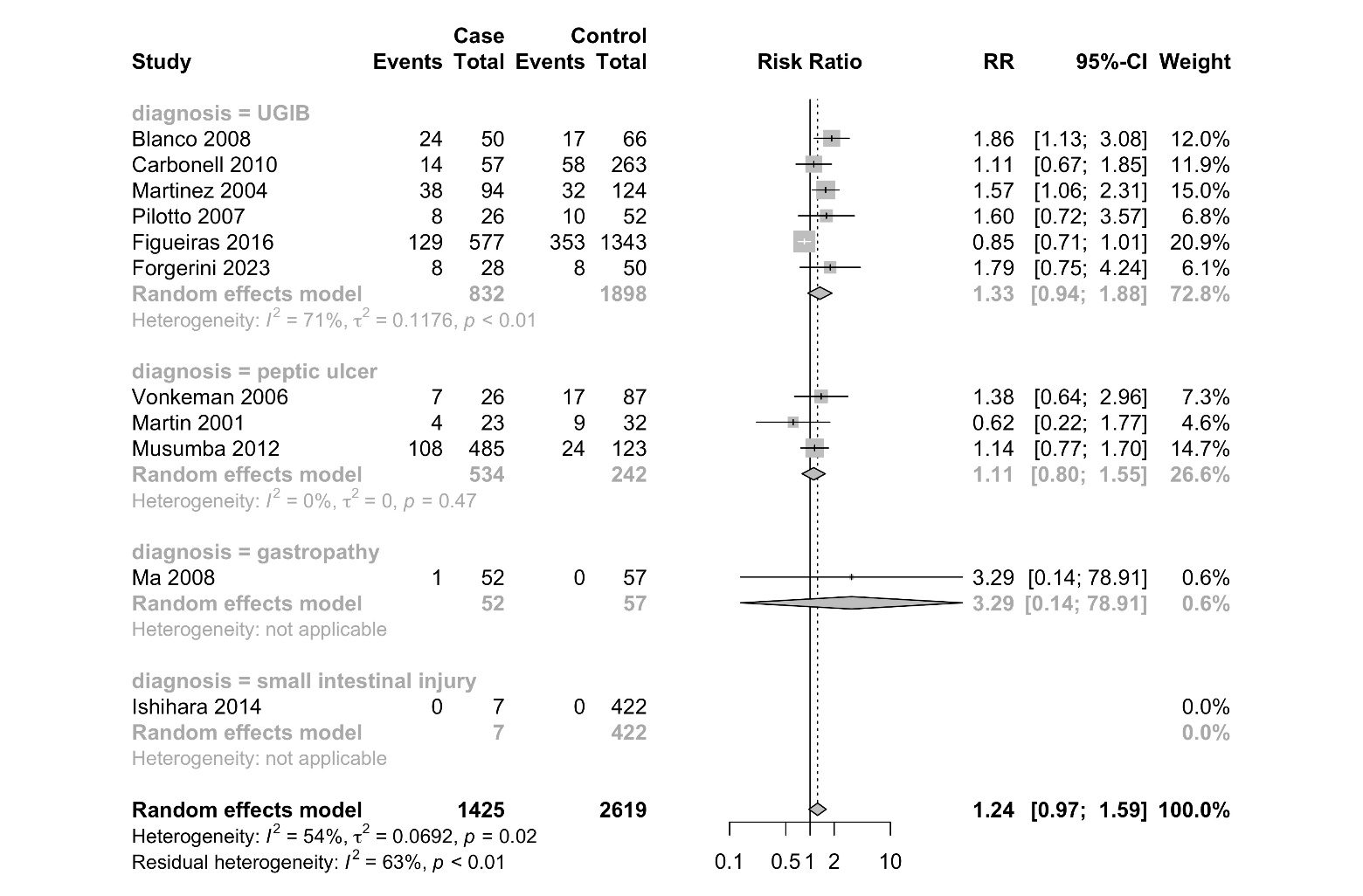
Figure: Forest Plot: the risk of different gastrointestinal adverse reactions after taking NSAIDs in CYP2C9*2 carrier**

**
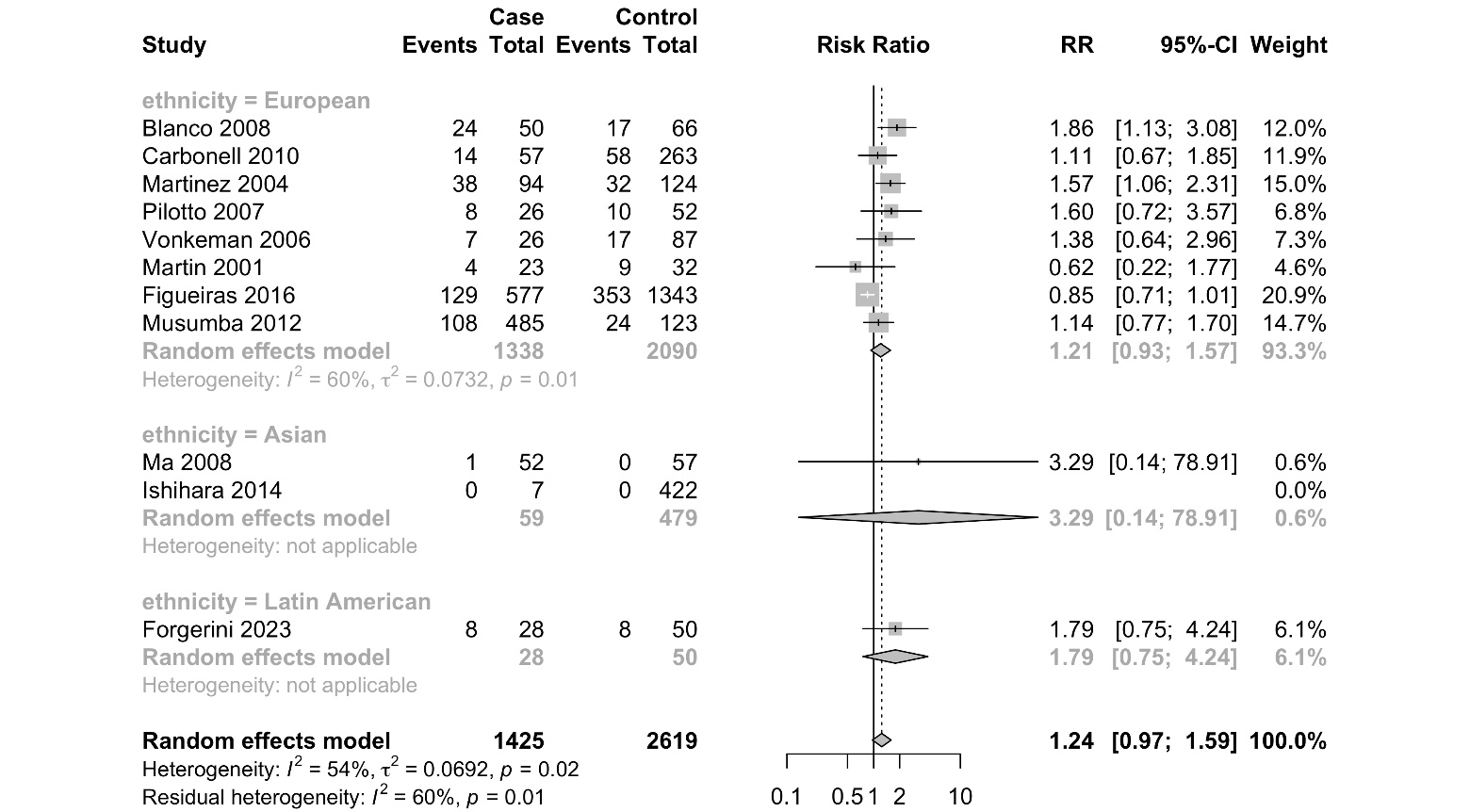
**

**Figure: Forest Plot: the risk of gastrointestinal adverse reactions after taking NSAIDs in CYP2C9*2 carrier of different ethnicity**

**Table: Studies addressing the risk of gastrointestinal adverse reactions after taking NSAIDs in CYP2C9*3 carrier**

| Study | Year | Design | Diagnosis of cases | Ethnicity | Number of case (CYP2C9*3 carrier) | Number of gastrointestinal adverse reactions in cases | Number of control (CYP2C9*1/*1) | Number of gastrointestinal adverse reactions in controls |
| --- | --- | --- | --- | --- | --- | --- | --- | --- |
| Forgerini[587] | 2023 | Case-control | UGIB | Latin American | 28 | 3 | 50 | 12 |
| Figueiras[585] | 2016 | Case-control | UGIB | European | 577 | 88 | 1343 | 177 |
| Ishihara[588] | 2014 | Case-control | Small intestinal injury | Asian | 7 | 2 | 422 | 18 |
| Musumba[589] | 2012 | Case-control | Peptic ulcer | European | 485 | 62 | 123 | 15 |
| Carbonell[590] | 2010 | Case-control | UGIB | European | 57 | 19 | 263 | 42 |
| Blanco[591] | 2008 | Case-control | UGIB | European | 50 | 12 | 66 | 13 |
| Ma[592] | 2008 | Cross-sectional | Gastropathy | Asian | 52 | 0 | 57 | 0 |
| Pilotto[593] | 2007 | Case-control | UGIB | European | 26 | 10 | 52 | 5 |
| Vonkeman[594] | 2006 | Case-control | Peptic ulcer | European | 26 | 2 | 87 | 14 |
| Martinez[595] | 2004 | Case-control | UGIB | European | 94 | 15 | 124 | 19 |
| Martin[596] | 2001 | Case-control | Peptic ulcer | European | 23 | 3 | 32 | 4 |


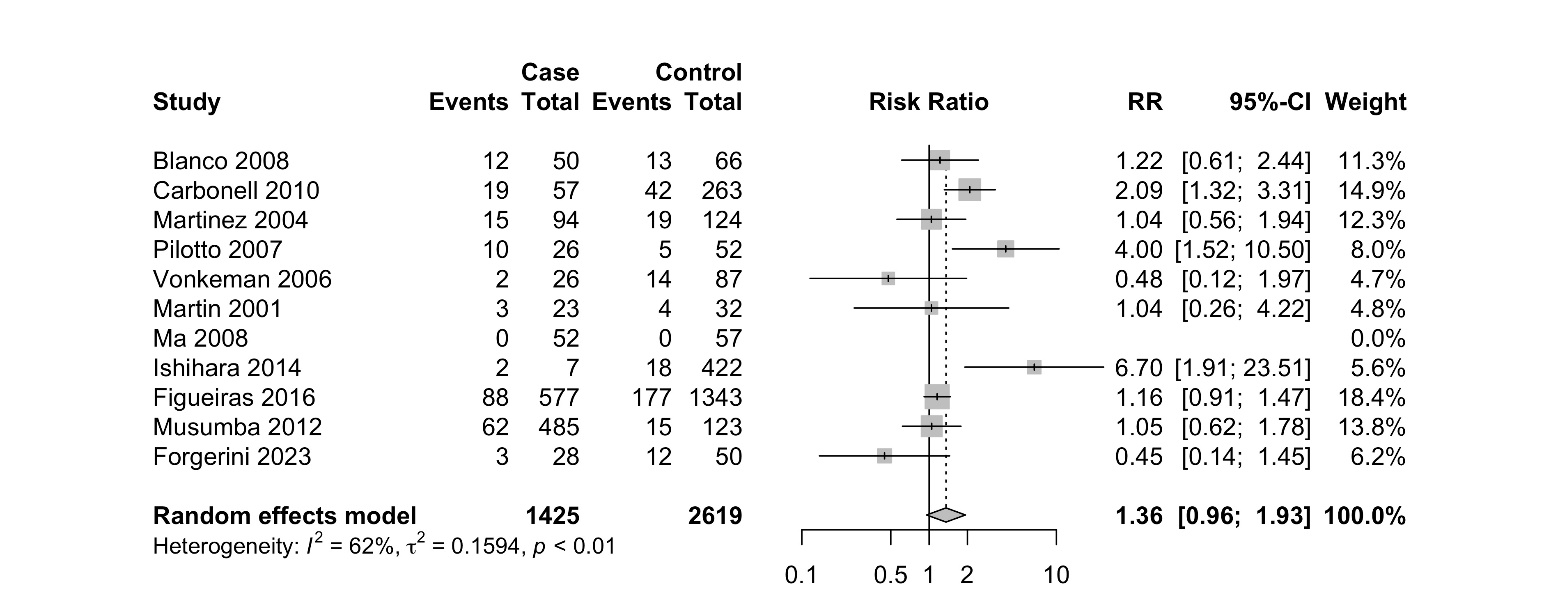
**Figure: Forest Plot: the risk of gastrointestinal adverse reactions after taking NSAIDs in CYP2C9*3 carrier**


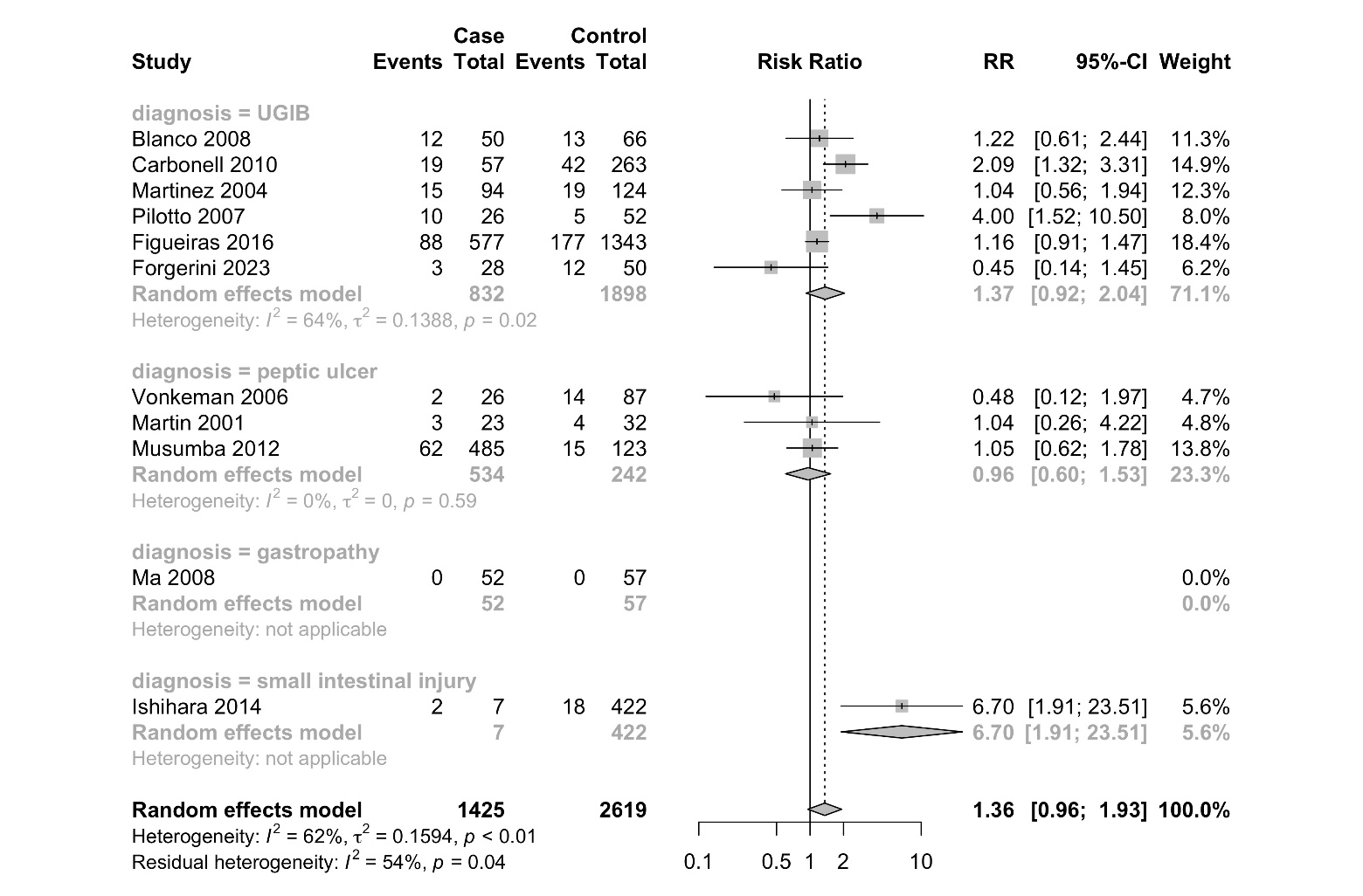
**Figure: Forest Plot: the risk of different gastrointestinal adverse reactions after taking NSAIDs in CYP2C9*3 carrier**


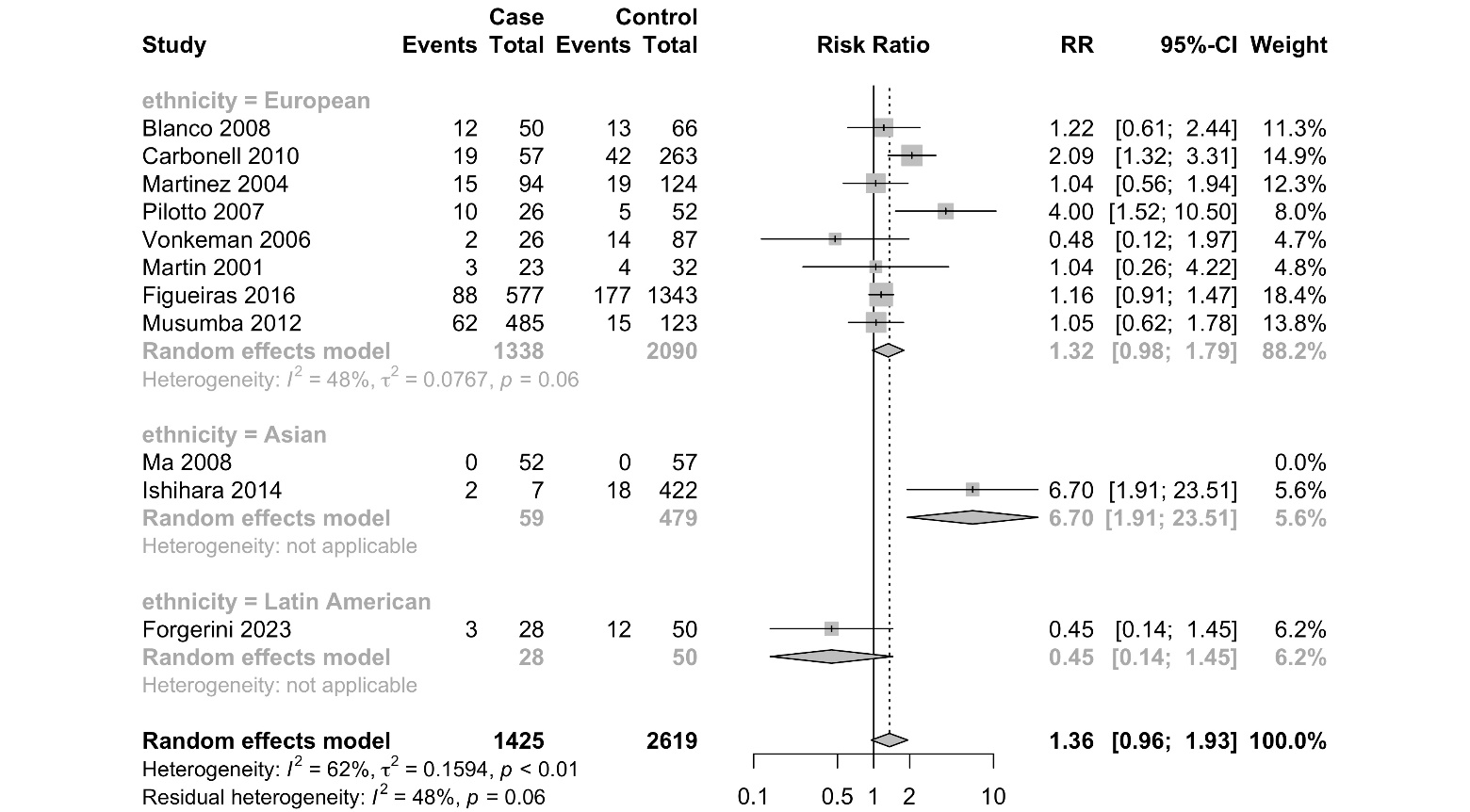
**Figure: Forest Plot: the risk of gastrointestinal adverse reactions after taking NSAIDs in CYP2C9*3 carrier of different ethnicity**

**Table: Studies addressing other genetic polymorphisms associated with gastrointestinal adverse reactions after taking NSAIDs**

| Study | Year | Design | Population | Result |
| --- | --- | --- | --- | --- |
| Forgerini[587] | 2023 | Case-control | 200 patients with gastrointestinal bleeding and 706 HC | For defined daily dose of NSAIDs upward of 0.50, a higher risk of UGIB was identified in carriers of the variant homozygous genotype (TT) of VKORC1 (OR: 38,850, 95% CI: 2.70–556.00) compared to carriers of the heterozygous genotype (CT) (OR: 6.025, 95% CI: 2.40–15.12). |
| Forgerini[597] | 2021 | Case-control | 200 patients with gastrointestinal bleeding and 706 HC | Considering the PTGS1 gene, in NSAIDs users, the four variants evaluated were associated with the risk of UGIB (rs1330344, rs3842787, rs10306114, and rs5788), and this risk ranged from 2.71 in carriers of the CA + AA genotypes vs. CC of rs5788 (CI 95%: 1.251–5.88) to 5.69 in carriers of the AG + GG genotypes vs. AA of rs10306114 (CI 95%: 1.46–22.07). |
| Groza[598] | 2017 | Case-control | 163 patients with gastrointestinal bleeding and 178 without | In subjects with NSAIDs/aspirin therapy and genotype VKORC1 -1639 G>A AA there was a two times higher chance of UGIB compared to those under NSAIDs/aspirin therapy alone (OR 7.6 vs. 3.6, p<0.001). |
| Blanco[591] | 2008 | Case-control | 134 patients with gastrointestinal bleeding and 177 without | Among gastrointestinal bleeding patients receiving NSAID that are CYP2C8/9 substrates the frequencies for CYP2C8*3 carriers of variant alleles versus control patients were 0.50 vs. 0.23 (OR; 95% CI=3.4; 1.5-7.5; P=0.002). The frequencies for carriers of the CYP2C8*3+CYP2C9*2 genotype versus control patients were 0.40 vs. 0.15 (OR; 95% CI=3.7; 1.6-8.9; P=0.003). |
| Skarke[599] | 2006 | Case-control | 10 healthy -765GG genotype carrier and 10 healthy -765CC genotype carrier | The PTGS2 -765G>C single-nucleotide polymorphism does not modulate COX-2 inhibitory effects of celecoxib as assessed by an ex vivo whole blood assay. |

**Question 25: SSZ-related genes**

We found 9 Cohort study addressing this question.

The evidence shows:

1.Most of sulfasalazine is hydrolyzed in the colon into 5-aminosalicylic acid and sulfapyridine, and the latter is absorbed into blood and metabolized in the liver by N-acetyltransferase 2 (NAT2). Individuals carrying the wild type gene of NAT2, namely NAT2*4 could be categorized as fast acetylator, while those carrying the mutated genes NAT2*5, 6, 7 could be categorized as slow acetylator. [600]

2.Our meta-analysis confirmed that the slow acetylators carrying the mutated genes NAT2*5, 6 were at a significantly higher risk of dose-dependent adverse events, such as nausea, vomiting, dizziness, but slow acetylation was not associated with hypersensitivity-related adverse events, such as skin rash or granulocytopenia. Interestingly, mutations of NAT2 are very prevalent across the general population (~50%).[601]

3.Apart from NAT2, ABCG2 is another gene reported to be associated with the safety and efficacy of SSZ. However, studies investigating ABCG2 were limited, and more research is needed to confirm its association with safety and efficacy of SSZ.[602]

The quality of evidence is LOW.

**Table: Evidence profile**

| Certainty assessment | | | | | | | Summary of findings | |
| --- | --- | --- | --- | --- | --- | --- | --- | --- |
| No of participants  (studies)  Follow-up | Risk of bias | Inconsistency | Indirectness | Imprecision | Publication bias | Overall certainty of evidence | Pooled Result (95%CI) | Brief Summary |
| Question 25 : | | | | | | | | |
| 9 studies (9 Cohort studies) | Serious | Not Serious | Not serious | Not serious | Not serious | ⨁⨁◯◯  LOW | \ | The slow acetylators carrying the mutated genes NAT2*5, 6 were at a significantly higher risk of dose-dependent adverse events, such as nausea, vomiting, dizziness, but slow acetylation was not associated with hypersensitivity-related adverse events, such as skin rash or granulocytopenia. Interestingly, mutations of NAT2 are very prevalent across the general population (~50%). |

**Table: Studies addressing the value of SSZ-related genes as a biomarker of AS**

| Study | Year | Design | Population | Result |
| --- | --- | --- | --- | --- |
| Ricart[603] | 2002 | Retrospective cohort study | 64 UC | The toxicity rates among the 64 patients treated with sulfasalazine were 34% (12 of 35) for slow acetylators and 45% (13 of 29) for rapid acetylators (odds ratio 0.6, 95% CI 0.2–1.8, p 0.65).  NAT1 and NAT2 genotypes did not predict response to mesalamine or sulfasalazine, or toxicity to sulfasalazine |
| Chen[604] | 2007 | Prospective  cohort study | 68 IBD/ 109 HC | Those with the slow acetylator genotypes experienced more SP dose-related adverse effects than those with the fast acetylator genotypes (36% versus 8%, OR of 0.17, 95% CI 0.039 to 0.749; P=0.019).NAT2 slow acetylator genotypes were significantly associated with SP dose-related  adverse effects of SASP in the treatment of IBD. |
| Hou[605] | 2014 | Prospective  cohort study | 266 AS/ 280 HC | The prevalence of SSZ-induced ADRs and drug withdrawal was 9.4% and 7.1%, respectively. The  frequencies of overall ADRs, dose-related ADRs, and termination of drug treatment because of intolerance were higher in the NAT2 slow acetylator genotype carriers than in the fast-type carriers and in those with co-existence of NAT1 and NAT2 slow acetylator genotypes. Furthermore, the ADRs emerged earlier in the AS cases carrying both NAT1 and NAT2 slow acetylator genotypes. |
| Tanaka[606] | 2002 | Retrospective cohort study | 144 RA | Sixteen patients (11.1%) had experienced adverse effects from SSZ, the most common being allergic reactions including rash and fever. The slow acetylators who had no NAT2*4 haplotype had experienced adverse effects more frequently (62.5%) than the fast acetylators who had at  least one NAT2*4 haplotype (8.1%) (p < 0.001, OR 7.73, 95% CI 3.54–16.86). In 25% of the slow acetylators, the adverse effects were so severe that they were hospitalized. |
| Sabbagh[607] | 1997 | Prospective  cohort study | 11 CDLE | SA seem to be more prone to toxic events.These findings strongly suggest that the genetic polymorphism of NAT2 is responsible for differences in the response to sulfasalazine in patients with CDLE. Therefore, candidates for sulfasalazine therapy should be genotyped to identify those patients who might benefit from the drug. |
| Tanigawara[608] | 2002 | Retrospective cohort study | 13 IBD/8 HC | In patients with IBD, skin rash was seen in 3 of 6 Rapid Types and 1 of 6 Intermediate Types, consistent with the concept that hypersensitive reactions are independent of serum SP concentrations. In contrast, SASP dosing-related acute pancreatitis was found in the Slow Type patient. |
| Kumagai[609] | 2004 | Retrospective cohort study | 96 RA/180 HC | There was no clear difference in the genotype frequencies between RA patients and healthy subjects. NAT2 genotypes significantly affected both the plasma concentration ratios of SP to AcSP (SP/AcSP) and the efficacy of SASP (p < 0.05). Adverse reactions to SASP were found in 26 (27.1%) out of 96 patients,  and there was no difference among the three genotype groups. |
| Taniguchi[610] | 2007 | Retrospective cohort study | 186 RA | Patients without the wild-type haplotype at NAT2 were more likely to suffer from overall adverse events[n= 186, P = 0.001, relative risk (RR) 3.31, 95% confidence interval (CI) 1.76–6.22] and severe adverse events (P = 0.015, RR 24.6, 95% CI 2.37–254.53) by sulfasalazine. Patients with the T allele at C677T in MTHFR were more susceptible to overall adverse events (n= 156, P = 0.003;RR 2.4, 95% CI 1.29–4.55) while patients with the C allele at A1298C were less likely to be treated with a higher dose ( > 6 mg/week) of methotrexate in one year of treatment (n= 159, P = 0.008, RR 1.84, 95% CI 1.12–3.01). In all three association studies, the results were essentially the same as previously reported. |
| Wiese et al.  [611] | 2014 | Prospective  cohort study | 229 RA | After correction for conventional variables, toxicity in 229 participants was influenced by NAT2 phenotype (hazard ratio ¼ 1.74 (95% confidence interval (CI) 1.01–3.21), P ¼ 0.044) and remission in 141 participants was associated with ABCG2 genotype (odds ratio ¼ 3.34 (95% CI 1.18–9.50), P ¼ 0.024). In our sample of early RA patients who were primarily treated with a combination of DMARDs, common variants in genes that encode NAT2 and ABCG2 were associated respectively with toxicity and response to SSA. |


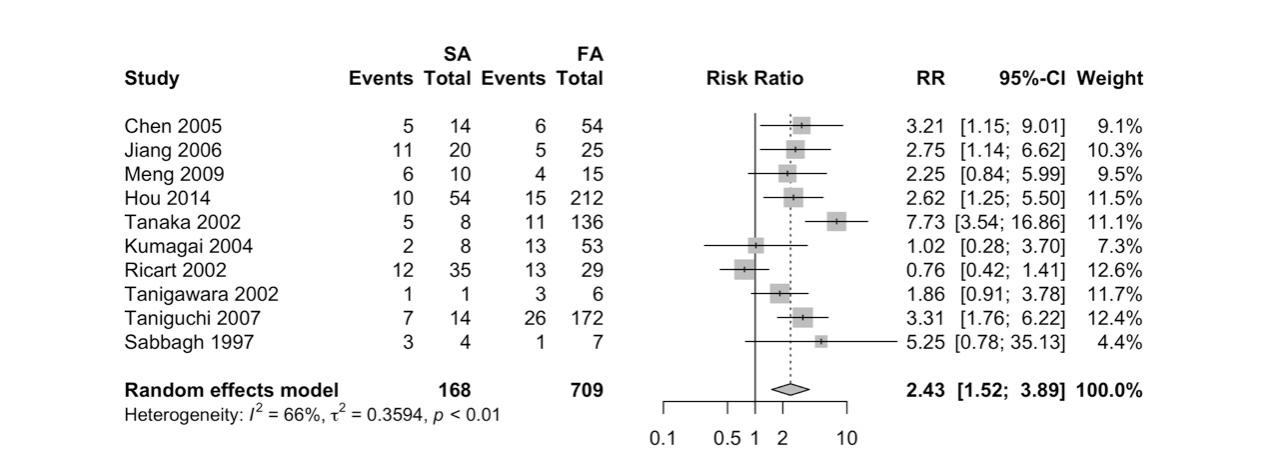


**Figure: Forest Plot of comparison:The risk of adverse reactions in slow and rapid acetylator status after taking SASP**


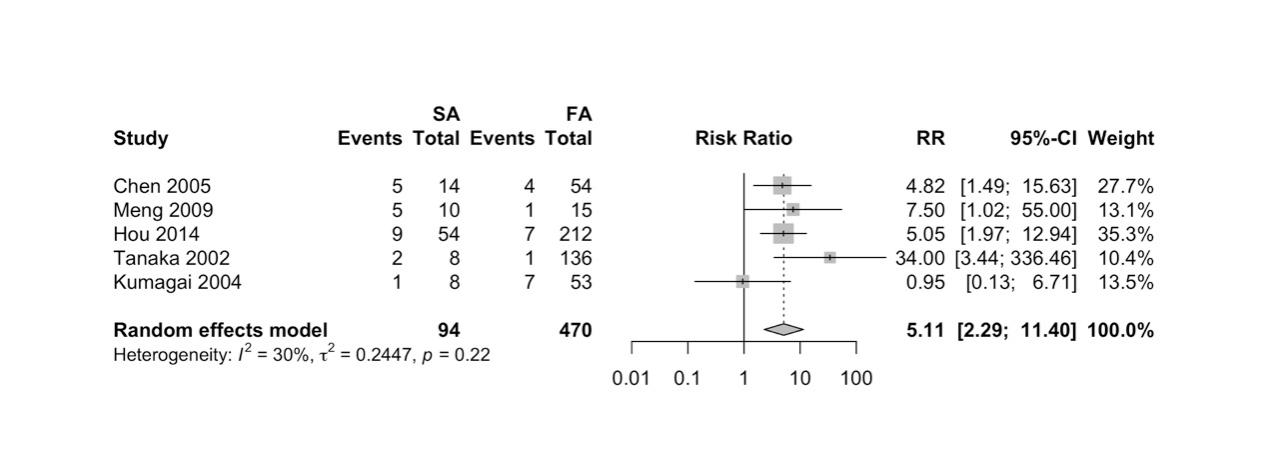


**Figure: Forest Plot of comparison: The risk of dose-dependent adverse reactions in slow and rapid acetylator status after taking SASP**


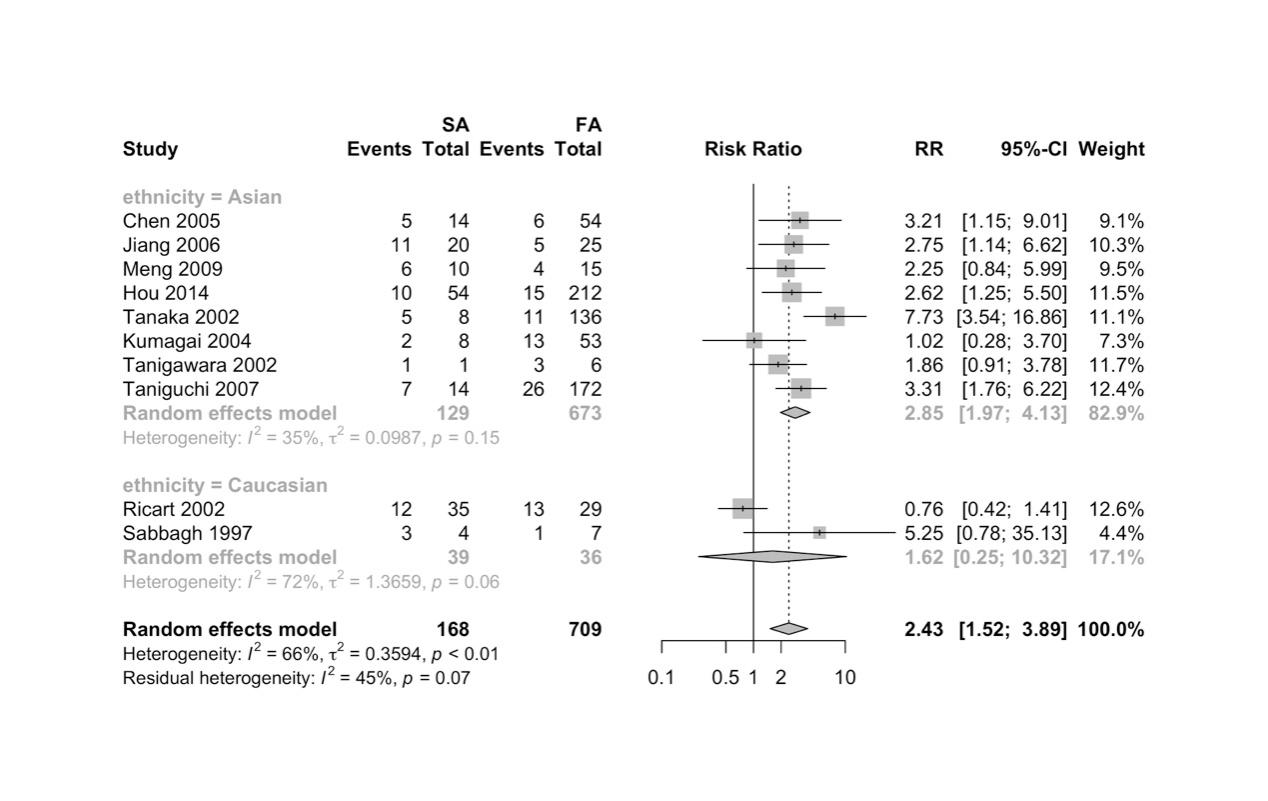


**Figure: Forest Plot of comparison:The risk of adverse reactions in slow and rapid acetylators of different races after taking SASP.**

**Question 26: Anti-drug antibodies**

We found 17 Cohort and 3 Cross-sectional studies addressing this question.

The evidence shows:

1.The incidence rates of anti-drug antibodies (ADAbs) vary across different TNF-a inhibitors. Research reported that the incidence rates of ADAbs infliximab and adalimumab were from 7% to 47.3%, while certolizumab pegol was reported to be 6.9%. ADAbs were very rarely detected in patients treated with etanecept.[612]

2.Our meta-analysis revealed that ADAbs were significantly associated with injection site skin reactions, lower drug concentrations. Administration of methotrexate could lower the occurrence rates of ADAbs.

3.IL-17 inhibitors generally exhibited good immunogenicity. The incidence rate of ADAbs in secukinumab was less than 1%. For ixekizumab, the general incidence rate of ADAbs was 9-19.4%, yet such ADAbs could not predict treatment outcomes. It is currently believed that ADAbs to secukinumab, ixekizumab and bimekizumab were not associated with adverse events.[613] Based on the evidence above, we considered that it is not necessary to examine ADAbs in patients treated with IL-17 inhibitors.

The quality of evidence is MEDIUM.

**Table: Evidence profile**

| Certainty assessment | | | | | | | Summary of findings | |
| --- | --- | --- | --- | --- | --- | --- | --- | --- |
| No of participants  (studies)  Follow-up | Risk of bias | Inconsistency | Indirectness | Imprecision | Publication bias | Overall certainty of evidence | Pooled Result (95%CI) | Brief Summary |
| Question 26: | | | | | | | | |
| 20 studies (17 Cohort and 3 Cross-sectional studies) | Not Serious | Not serious | Not serious | Not serious | Not serious | ⨁⨁⨁◯  MEDIUM | \ | The incidence rates of ADAbs infliximab and adalimumab were from 7% to 47.3%, while certolizumab pegol was reported to be 6.9%. The incidence rate of ADAbs in secukinumab was less than 1%. For ixekizumab, the general incidence rate of ADAbs was 9-19.4%. ADAbs were significantly associated with injection site skin reactions, lower drug concentrations. |

**Table: Studies addressing the detected rate of anti-drug antibodies in patients with AS**

| Study | Year | Design | Patients diagnosis | Patient number | ADAbs(+) | Positive rate (%) | Result |
| --- | --- | --- | --- | --- | --- | --- | --- |
| Ducourau[614] | 2011 | Cohort | SpA | 91 | 14 | 15.4 | In patients with SpA, antibodies towards infliximab were detected in 15%(14/91) of them. |
| Plasencia[615] | 2013 | Cohort | SpA | 42 | 11 | 26.2 | ADA were detected in 26.2%(11/42) of all SpA patients |
| Paramarta[616] | 2014 | Cohort | SpA | 26 | 6 | 23.1 | At the end of the treatment period, 6 (23.1%) of 26 patients tested positive for serum antiadalimumab ADAbs. |
| Park[617] | 2017 | Cohort | AS | 174 | 44 | 25.3 | At week 102, the proportions of patients with ADAs in the maintenance and switch groups were 23.3%(21/90) and 27.4%(23/84), respectively. |
| Gehin[618] | 2019 | Cohort | SpA | 116 | 6 | 5.2 | After 3 months of treatment, 6 of 116 (5.2%) aSpA patients were ADAb positive. |
| Ducourau[619] | 2020 | Cohort | SpA | 107 | 39 | 36.4 | ADA were detected at W26 in 39/107 (36.4%) patients. |
| Park[620] | 2013 | Cohort | AS | 250 | 57 | 22.8 | Antibodies to infliximab with active AS patients were detected in 27.4% (n=32) and 22.5% (n=25) of patients for CT-P13 and INX, respectively, at week 30. |
| Kneepkens[621] | 2015 | Cohort | AS | 115 | 31 | 27.0 | 13(11.3%) patients had detectable ADAb at week 12 after start of treatment and 31 (27.0%) at week 24. |
| Jung[622] | 2014 | Cohort | AS | 177 | 18 | 10.2 | ADAs were above the cut-off level in 18 (10.2%) patients with AS. |
| Hoxha[623] | 2016 | Cohort | AS | 22 | 4 | 18.2 | Anti-ADA antibodies were positive in4/22 (18.2%) with AS. |
| de Vries[624] | 2007 | Cohort | AS | 38 | 11 | 28.9 | After 54 weeks, 11/38 (29%) patients showed undetectable serum trough infliximab levels and detectable anti-infliximab. |
| Braun[625] | 2008 | Cohort | AS | 239 | 23 | 9.6 | Through week 102,the incidence of antibodies to infliximab was 23/239 (9.6%). |
| Arends[626] | 2010 | Cohort | AS | 60 | 10 | 16.7 | Anti-IFX, anti-ETA, and anti-ADA antibodies were induced in 4/20(20%), 0/20(0%）, and 6/20(30%）of AS patients, respectively. |
| Su[627] | 2020 | Cohort | AS | 641 | 115 | 17.9 | The number (proportion) of patients at week 24 was 72/412 (17.5%) in the HS016 group and 43/229 (18.8%) in the adalimumab group |
| Mahmoud[628] | 2020 | Cross-sectional | SpA | 71 | 21 | 29.6 | ADA was positive in 54%(12/71) for INF, 33% (9/71)for ADL and 0%(0/71) for ETA with a significant difference(p<0.0001). |
| de Vries[629] | 2009 | Cohort | AS | 53 | 0 | 0.0 | Antibodies against etanercept were not detected in all patients with any of the assays after 6 month treatment. |
| Balsa[630] | 2018 | Cross-sectional | SpA | 293 | 70 | 23.9 | ADA were found in 70/ 293 (23.8%) SpA patients; 35/293 (11.9%) against INF and 35/293 (11.9%) against ADL; none against ETN. |
| Arstikyte[631] | 2015 | Cross-sectional | SpA | 81 | 6 | 7.4 | Anti-ADA Ab,Anti-ETA Ab and anti-INF Ab were respectively detected in 0/81(0%) ,0/81(0%) and 6/81(18.2%) in SpA patients. |
| Plasencia[632] | 2012 | Cohort | SpA | 94 | 24 | 25.5 | ATI were detected in 24/94 (25.5%) patients. |
| Méric[633] | 2011 | Cohort | SpA | 32 | 6 | 18.8 | 6/32(18.8%) patients were detected as being positive for ATI. |

**Table: Studies addressing the effect of anti-drug antibodies on bDMARDs therapeutic efficacy in patients with AS**

| Study | Year | Design | Population | Result |
| --- | --- | --- | --- | --- |
| Ducourau[614] | 2011 | Cohort | SpA | AIT positive patients in SpA had lower infliximab concentration and shorter time of infliximab maintenance than AIT negetive patients. |
| Plasencia[615] | 2013 | Cohort | SpA | At 6 months after switching to the second anti-TNF drugs, patients who had developed ADA against the first anti-TNF drug had lower disease activity, as measured by the ASDAS than those without ADA(1.62 ± 0.93 with ADA vs. 2.79 ± 1.01 without ADA, p = 0.002). |
| Paramarta[616] | 2014 | Cohort | SpA | ADAbs at the end of treatment and at follow-up were not different between responders and nonresponders. |
| Park[617] | 2017 | Cohort | AS | There were significantly different between the proportion of ADA-negative and positive patients achieving ASAS20 in the maintenance group at 54 week and switch groups at week 102(75.7% vs 50%,p=0.04;84.6% vs 62.5%,p=0.04). |
| Gehin[618] | 2019 | Cohort | SpA | ADAb-positive patients had significantly lower CZP levels than ADAb-negative patients[1.0 (0.2–6.8) vs. 34.4 (21.2–44.7) mg/L ,P<0.01].Among ADAb negative patients with response data, 129/234 (55%) were responders,but only 1 out of 11 (9%) ADAb-positive patients was classified as a responder. |
| Ducourau[619] | 2020 | Cohort | SpA | MTX treatment was associated with higher adalimumab concentration at any time point (p<0.05).The clinical response was only poorer for the ADA- high than ADA- low and ADA- negative patients at W26 followup.The median survival of adalimumab in ADA- positive patients was 56.9 weeks (5.0–212.1), compared with 98.6 weeks (4.3–236.0) in ADA- negative patients (p=0.015). |
| Park[620] | 2013 | Cohort | AS | ADA-positive patients had a less robust ASAS20 response. |
| Kneepkens[621] | 2015 | Cohort | AS | Adalimumab levels (mg/L) were significantly different for patients without and with ADAb (12.7 (IQR 8.2–18.0) vs 1.2 (IQR 0.0–2.0), respectively; p<0.001) .At 24 weeks of treatment, ESR was significantly higher in ADAb-positive patients. |
| Jung[622] | 2014 | Cohort | AS | ADAs positive was highest in AS patients treated with INF, followed by patients treated with ADL and those treated with ETN. |
| Hoxha[623] | 2016 | Cohort | AS | Lack of response and/or loss of efficacy were observed in 8/22 (36.4%) with AS. |
| de Vries[624] | 2007 | Cohort | AS | 9% (1 of 11) patients with detectable anti-infliximab was classified as a responder at week 54, compared with 74% (20 of 27) of patients without anti-infliximab.Serum trough infliximab levels were significantly (p,0.0001) lower in patients with (mean 0.02 mg/l) than in those without (12.7 mg/l) anti-infliximab. |
| Arends[626] | 2010 | Cohort | AS | Patients with anti-IFX or anti-ADA antibodies had significantly lower serum TNF-α blocker levels compared to patients without these antibodies. Furthermore, significant negative correlations were found between serum TNF-α blocker levels and assessments of disease activity. |
| Su[627] | 2020 | Cohort | AS | There was no difference in clinical response, PK parameters, and TEAEs in the NAbpositive adalimumab and HS016 groups. |
| Mahmoud[628] | 2020 | Cross-sectional | SpA | The clinical activity and functional impact (BASDAI≥4, BASFI) were significantly higher in ADA positive group (57% vs 32%,p=0.04;4.9±2.4 vs 2.05±2.6,p=0.015). |
| Plasencia[632] | 2012 | Cohort | SpA | The patients with ATI had significantly higher clinical activity (as measured by the ASDAS) at 6 months (2.55±0.89 vs 1.79±1.04, p=0.038), 1 year (1.95±0.67 vs 1.67±0.71, p=0.042) and >4 years (2.52±0.99 vs 1.53±0.81, p=0.024) of follow-up. |
| Arstikyte[631] | 2015 | Cross-sectional | SpA | Patients with RA and SpA not responding to treatment had statistically significant higher anti-ADA (𝑃 < 0.0001) and anti-INF Ab (𝑃 < 0.0001) concentrations,but the same data separately in RA and SpA patients results did not differ statistically significant between responders and nonresponders. |

**Table: Studies addressing the effect of anti-drug antibodies on bDMARDs therapeutic safety in patients with AS**

| Study | Year | Design | Population | Result |
| --- | --- | --- | --- | --- |
| Ducourau[614] | 2011 | Cohort | SpA | Among ATI positive patients, 11 (52%) had at least one infusion-related reaction,as compared with only 1 (1%) in the ATI negetive group.Eighteen (86%) of the ATIpos patients and forty-one (47%) of the ATIneg patients discontinued infliximab during follow-up. |
| Park[617] | 2017 | Cohort | AS | There was not different in rate of Infusion-related reactions by ADA status. |
| Gehin[618] | 2019 | Cohort | SpA | Eight patients experienced one or more injection-site reaction, and all of these were ADAb negative at 3 months. |
| Ducourau[619] | 2020 | Cohort | SpA | Six patients (11%) in the MTX- group showed injection skin reactions as compared with one (2%) in the MTX+ group." |
| Park[620] | 2013 | Cohort | AS | The proportions of infusion-related reactions in CT-P13 and INX groups were 3.1% (n=1) vs 11.1% (n=3) for ADA-positive group and 3.4% (n=3) and 2.2% (n=2) for ADA-negative group, respectively. |
| Braun[625] | 2008 | Cohort | AS | Through week 102, patients positive for antibodies to infliximab had the highest occurrence[14/23 (60.9%] of infusion reactions. |
| Arstikyte[631] | 2015 | Cross-sectional | SpA | Patients with anti-INF Ab have higher odds to have infusion related reaction [OR 5.88 ,(95% CI 1.04–33.28)]. |
| Plasencia[632] | 2012 | Cohort | SpA | Eleven patients (12%) developed infusion-related reactions, and of these, ATI were present in eight patients (73%). |

**Table: Studies addressing the effect of MTX or DMARDs therapy on anti-drug antibodies developing in patients with AS**

| Study | Year | Design | Population | Result |
| --- | --- | --- | --- | --- |
| Ducourau[614] | 2011 | Cohort | SpA | For SpA patients, concomitant MTX treatment was lower for ATI positive than for ATI negetive patients (0 (0%) of 14 vs. 25 (32%) of 77, respectively; P = 0.03). |
| Plasencia[615] | 2013 | Cohort | SpA | For SpA patients, those without MTX treatment had higher rate for ADA positive than for ADA negetive patients,but there was no significant difference.(8 (25.8%) of 31 vs. 1 (9.1%) of 11, respectively; P = 0.498). |
| Paramarta[616] | 2014 | Cohort | SpA | There was no statistical difference in antiadalimumab ADAb positivity between patients taking DMARDs (5 (33.3%) of 15 patients,) vs. patients not taking DMARDs (1, (9.1%) of 11 patients) (P= 0.147). |
| Ducourau[619] | 2020 | Cohort | SpA | The proportion of ADA positive in the MTX+ group was lower than in the MTX− group (13/52 (25%) vs 26/55 (47.3%),p=0.03). |
| Balsa[630] | 2018 | Cross-sectional | SpA | Overall, a significantly lower proportion of patients receiving concomitant DMARDs [61/369 (16.5%)] vs those receiving anti-TNF monotherapy [53/201 (26.4%)] tested positive for ADA (P= 0.004). |
| Arstikyte[631] | 2015 | Cross-sectional | SpA | Negative correlation between MTX use and presence of anti-drug Ab in ADA patients,but in INF patients group correlation was not found. |
| Plasencia[632] | 2012 | Cohort | SpA | ATI development occurred more frequently in the patients not receiving methotrexate (20/58 (34.5%) vs 4/36 (11.1%), p=0.011). |

**REFERENCE**

1. Ellinghaus D, Jostins L, Spain SL, Cortes A, Bethune J, Han B, et al. Analysis of five chronic inflammatory diseases identifies 27 new associations and highlights disease-specific patterns at shared loci. Nat Genet. 2016 May; 48(5):510-518.

2. Lin H, Gong YZ. Association of HLA-B27 with ankylosing spondylitis and clinical features of the HLA-B27-associated ankylosing spondylitis: a meta-analysis. Rheumatol Int. 2017 Aug; 37(8):1267-1280.

3. Rudwaleit M, van der Heijde D, Landewé R, Listing J, Akkoc N, Brandt J, et al. The development of Assessment of SpondyloArthritis international Society classification criteria for axial spondyloarthritis (part II): validation and final selection. Ann Rheum Dis. 2009 Jun; 68(6):777-783.

4. Bennett AN, McGonagle D, O'Connor P, Hensor EM, Sivera F, Coates LC, et al. Severity of baseline magnetic resonance imaging-evident sacroiliitis and HLA-B27 status in early inflammatory back pain predict radiographically evident ankylosing spondylitis at eight years. Arthritis Rheum. 2008 Nov; 58(11):3413-3418.

5. Vastesaeger N, van der Heijde D, Inman RD, Wang Y, Deodhar A, Hsu B, et al. Predicting the outcome of ankylosing spondylitis therapy. Ann Rheum Dis. 2011 Jun; 70(6):973-981.

6. Rudwaleit M, Listing J, Brandt J, Braun J, Sieper J. Prediction of a major clinical response (BASDAI 50) to tumour necrosis factor alpha blockers in ankylosing spondylitis. Ann Rheum Dis. 2004 Jun; 63(6):665-670.

7. Jajić I. The role of HLA-B27 in the diagnosis of low back pain. Acta Orthop Scand. 1979 Aug; 50(4):411-413.

8. Sadowska-Wróblewska M, Filipowicz A, Garwolinska H, Michalski J, Rusiniak B, Wróblewska T. Clinical symptoms and signs useful in the early diagnosis of ankylosing spondylitis. Clin Rheumatol. 1983 Mar; 2(1):37-43.

9. Deesomchok U, Tumrasvin T. Clinical study of Thai patients with ankylosing spondylitis. Clin Rheumatol. 1985 Mar; 4(1):76-82.

10. Sampaio-Barros PD, Bertolo MB, Kraemer MHS, Marques-Neto JF, Samara AM. Undifferentiated spondyloarthropathies: A 2-year follow-up study. Clinical Rheumatology. 2001; 20(3):201-206.

11. Brandt HC, Spiller I, Song IH, Vahldiek JL, Rudwaleit M, Sieper J. Performance of referral recommendations in patients with chronic back pain and suspected axial spondyloarthritis. Ann Rheum Dis. 2007 Nov; 66(11):1479-1484.

12. Dincer U, Cakar E, Kiralp MZ, Dursun H. Diagnosis delay in patients with ankylosing spondylitis: possible reasons and proposals for new diagnostic criteria. Clin Rheumatol. 2008 Apr; 27(4):457-462.

13. Nazarinia MA, Ghaffarpasand F, Heiran HR, Habibagahi Z. Pattern of ankylosing spondylitis in an Iranian population of 98 patients. Mod Rheumatol. 2009; 19(3):309-315.

14. Liao ZT, Pan YF, Huang JL, Huang F, Chi WJ, Zhang KX, et al. An epidemiological survey of low back pain and axial spondyloarthritis in a Chinese Han population. Scand J Rheumatol. 2009 Nov-Dec; 38(6):455-459.

15. Aggarwal R, Malaviya AN. Clinical characteristics of patients with ankylosing spondylitis in India. Clin Rheumatol. 2009 Oct; 28(10):1199-1205.

16. Liu X, Li YR, Hu LH, Zhou ZM, Chen FH, Ning Y, et al. High frequencies of HLA-B27 in Chinese patients with suspected of ankylosing spondylitis. Rheumatol Int. 2010 Aug; 30(10):1305-1309.

17. van Onna M, Jurik AG, van der Heijde D, van Tubergen A, Heuft-Dorenbosch L, Landewé R. HLA-B27 and gender independently determine the likelihood of a positive MRI of the sacroiliac joints in patients with early inflammatory back pain: a 2-year MRI follow-up study. Ann Rheum Dis. 2011 Nov; 70(11):1981-1985.

18. Liao Z, Lin Z, Xu M, Hu Z, Li T, Wei Q, et al. Clinical features of axial undifferentiated spondyloarthritis (USpA) in China: HLA-B27 is more useful for classification than MRI of the sacroiliac joint. Scand J Rheumatol. 2011 Nov; 40(6):439-443.

19. Chung HY, Machado P, van der Heijde D, D'Agostino MA, Dougados M. HLA-B27 positive patients differ from HLA-B27 negative patients in clinical presentation and imaging: results from the DESIR cohort of patients with recent onset axial spondyloarthritis. Ann Rheum Dis. 2011 Nov; 70(11):1930-1936.

20. De Carvalho HMS, Bortoluzzo AB, Gonçalves CR, Da Silva JAB, Ximenes AC, Bértolo MB, et al. Gender characterization in a large series of Brazilian patients with spondyloarthritis. Clinical Rheumatology. 2012; 31(4):687-695.

21. van den Berg R, de Hooge M, van Gaalen F, Reijnierse M, Huizinga T, van der Heijde D. Percentage of patients with spondyloarthritis in patients referred because of chronic back pain and performance of classification criteria: experience from the Spondyloarthritis Caught Early (SPACE) cohort. Rheumatology (Oxford). 2013 Aug; 52(8):1492-1499.

22. Qi J, Li Q, Lin Z, Liao Z, Wei Q, Cao S, et al. Higher risk of uveitis and dactylitis and older age of onset among ankylosing spondylitis patients with HLA-B*2705 than patients with HLA-B*2704 in the Chinese population. Tissue Antigens. 2013 Dec; 82(6):380-386.

23. Peláez-Ballestas I, Navarro-Zarza JE, Julian B, Lopez A, Flores-Camacho R, Casasola-Vargas JC, et al. A community-based study on the prevalence of spondyloarthritis and inflammatory back pain in Mexicans. J Clin Rheumatol. 2013 Mar; 19(2):57-61.

24. Tomero E, Mulero J, de Miguel E, Fernández-Espartero C, Gobbo M, Descalzo MA, et al. Performance of the Assessment of Spondyloarthritis International Society criteria for the classification of spondyloarthritis in early spondyloarthritis clinics participating in the ESPERANZA programme. Rheumatology (Oxford). 2014 Feb; 53(2):353-360.

25. Kassimos DG, Vassilakos J, Magiorkinis G, Garyfallos A. Prevalence and clinical manifestations of ankylosing spondylitis in young Greek males. Clin Rheumatol. 2014 Sep; 33(9):1303-1306.

26. Nakashima Y, Ohishi M, Okazaki K, Fukushi JI, Oyamada A, Hara D, et al. Delayed diagnosis of ankylosing spondylitis in a Japanese population. Modern Rheumatology. 2016; 26(3):421-425.

27. Burgos-Vargas R, Wei JCC, Rahman MU, Akkoc N, Haq SA, Hammoudeh M, et al. The prevalence and clinical characteristics of nonradiographic axial spondyloarthritis among patients with inflammatory back pain in rheumatology practices: A multinational, multicenter study. Arthritis Research and Therapy. 2016; 18(1).

28. Bautista-Molano W, Landewé RB, Londoño J, Romero-Sanchez C, Valle-Oñate R, van der Heijde D. Analysis and performance of various classification criteria sets in a Colombian cohort of patients with spondyloarthritis. Clin Rheumatol. 2016 Jul; 35(7):1759-1767.

29. Bandinelli F, Salvadorini G, Delle Sedie A, Riente L, Bombardieri S, Matucci-Cerinic M. Impact of gender, work, and clinical presentation on diagnostic delay in Italian patients with primary ankylosing spondylitis. Clin Rheumatol. 2016 Feb; 35(2):473-478.

30. Arnbak B, Grethe Jurik A, Hørslev-Petersen K, Hendricks O, Hermansen LT, Loft AG, et al. Associations Between Spondyloarthritis Features and Magnetic Resonance Imaging Findings: A Cross-Sectional Analysis of 1,020 Patients With Persistent Low Back Pain. Arthritis Rheumatol. 2016 Apr; 68(4):892-900.

31. Fırat SN, Yazıcı A, Yılmazer B, Coşan F, Savlı H, Cefle A. Low frequency of HLA-B27 in ankylosing spondylitis and its relationship with clinical findings in patients from Turkey. Eur J Rheumatol. 2017 Dec; 4(4):268-271.

32. Ez-Zaitouni Z, Bakker PAC, van Lunteren M, Berg IJ, Landewé R, van Oosterhout M, et al. Presence of multiple spondyloarthritis (SpA) features is important but not sufficient for a diagnosis of axial spondyloarthritis: data from the SPondyloArthritis Caught Early (SPACE) cohort. Ann Rheum Dis. 2017 Jun; 76(6):1086-1092.

33. Tong F, Lv Q, Li A, Fang L, Luo Z, Feng J, et al. An epidemiological study of the prevalence rate of inflammatory back pain and axial spondyloarthritis in a university in the south of China. Clin Rheumatol. 2018 Nov; 37(11):3087-3091.

34. Endo Y, Fujikawa K, Koga T, Mizokami A, Mine M, Tsukada T, et al. Characteristics of late-onset spondyloarthritis in Japan: A retrospective cohort study. Medicine (Baltimore). 2019 Feb; 98(7):e14431.

35. Passalent L, Sundararajan K, Perruccio AV, Hawke C, Coyte PC, Bombardier C, et al. Bridging the Gap Between Symptom Onset and Diagnosis in Axial Spondyloarthritis. Arthritis Care Res (Hoboken). 2022 Jun; 74(6):997-1005.

36. Edara M, Bhatt V, Zanwar A, Koneru K, Patel AM, Jawade P. A study of clinical, radiological features and HLA-B27 serology of axial spondyloarthropathy with comparison of radiographic and non-radiographic disease. J Family Med Prim Care. 2022 Aug; 11(8):4417-4423.

37. Chung HY, Huang JX, Lee KH, Tsang HHL, Lau CS, Chan SCW. MRI lesions in SpA: a comparison with noninflammatory back pain using propensity score adjustment method. Therapeutic Advances in Musculoskeletal Disease. 2022; 14.

38. Puhakka KB, Jurik AG, Schiottz-Christensen B, Hansen GV, Egund N, Christiansen JV, et al. Magnetic resonance imaging of sacroiliitis in early seronegative spondylarthropathy. Abnormalities correlated to clinical and laboratory findings. Rheumatology (Oxford). 2004 Feb; 43(2):234-237.

39. Ma HJ, Yin QF, Hu FP, Guo MH, Liu XD, Liu Y, et al. Different clinical features in patients with ankylosing spondylitis from southern and northern China. Int J Rheum Dis. 2012 Apr; 15(2):154-162.

40. Londono J, Romero-Sanchez MC, Torres VG, Bautista WA, Fernandez DJ, Quiroga JA, et al. The association between serum levels of potential biomarkers with the presence of factors related to the clinical activity and poor prognosis in spondyloarthritis. Revista Brasileira de Reumatologia. 2012; 52(4):536-544.

41. Weiss PF, Xiao R, Biko DM, Chauvin NA. Assessment of Sacroiliitis at Diagnosis of Juvenile Spondyloarthritis by Radiography, Magnetic Resonance Imaging, and Clinical Examination. Arthritis Care Res (Hoboken). 2016 Feb; 68(2):187-194.

42. Baraliakos X, Szumski A, Koenig A, Jones H. C-reactive protein as a predictor of treatment response in patients with ankylosing spondylitis. Clinical and experimental rheumatology. 2014; 32(5):787.

43. Al-Saleh JA, Saab MA, Negm A, Balushi F, Namas R, Ziade N. Predictors of not Achieving Remission or Low Disease Activity in Axial Spondyloarthritis Patients from Middle Eastern Countries: A Prospective, Multicenter, Real-world Study. Oman Medical Journal. 2022; 37(3).

44. Huerta-Sil G, Casasola-Vargas JC, Londoño JD, Rivas-Ruíz R, Chávez J, Pacheco-Tena C, et al. Low grade radiographic sacroiliitis as prognostic factor in patients with undifferentiated spondyloarthritis fulfilling diagnostic criteria for ankylosing spondylitis throughout follow up. Ann Rheum Dis. 2006 May; 65(5):642-646.

45. Sampaio-Barros PD, Bortoluzzo AB, Conde RA, Costallat LT, Samara AM, Bértolo MB. Undifferentiated spondyloarthritis: a longterm followup. J Rheumatol. 2010 Jun; 37(6):1195-1199.

46. Bakker PAC, Ramiro S, Ez-Zaitouni Z, van Lunteren M, Berg IJ, Landewé R, et al. Is it Useful to Repeat Magnetic Resonance Imaging of the Sacroiliac Joints After Three Months or One Year in the Diagnosis of Patients With Chronic Back Pain and Suspected Axial Spondyloarthritis? Arthritis Rheumatol. 2019 Mar; 71(3):382-391.

47. Lorenzin M, Ortolan A, Felicetti M, Vio S, Favero M, Polito P, et al. Spine and Sacroiliac Joints Lesions on Magnetic Resonance Imaging in Early Axial-Spondyloarthritis During 24-Months Follow-Up (Italian Arm of SPACE Study). Front Immunol. 2020; 11:936.

48. Yi L, Wang J, Guo X, Espitia MG, Chen E, Assassi S, et al. Profiling of hla-B alleles for association studies with ankylosing spondylitis in the chinese population. Open Rheumatol J. 2013; 7:51-54.

49. Siala M, Mahfoudh N, Gdoura R, Younes M, Fourati H, Kammoun A, et al. Distribution of HLA-B27 and its alleles in patients with reactive arthritis and with ankylosing spondylitis in Tunisia. Rheumatol Int. 2009 Aug; 29(10):1193-1196.

50. Ren EC, Koh WH, Sim D, Boey ML, Wee GB, Chan SH. Possible protective role of HLA-B*2706 for ankylosing spondylitis. Tissue Antigens. 1997 Jan; 49(1):67-69.

51. Pazar B, Safrany E, Gergely P, Szanto S, Szekanecz Z, Poor G. Association of ARTS1 gene polymorphisms with ankylosing spondylitis in the Hungarian population: the rs27044 variant is associated with HLA-B*2705 subtype in Hungarian patients with ankylosing spondylitis. J Rheumatol. 2010 Feb; 37(2):379-384.

52. Park SH, Kim J, Kim SG, Kim SK, Chung WT, Choe JY. Human leucocyte antigen-B27 subtypes in Korean patients with ankylosing spondylitis: higher B*2705 in the patient group. Int J Rheum Dis. 2009 Apr; 12(1):34-38.

53. Mou Y, Wu Z, Gu J, Liao Z, Lin Z, Wei Q, et al. HLA-B27 polymorphism in patients with juvenile and adult-onset ankylosing spondylitis in Southern China. Tissue Antigens. 2010 Jan; 75(1):56-60.

54. Ma HJ, Hu FP. Diversity of human leukocyte antigen-B27 alleles in Han population of Hunan province, southern China. Tissue Antigens. 2006 Aug; 68(2):163-166.

55. Luo F, Zhao Z, Zhang J, Leng J. Comparison of HLA-B*27 subtypes between Chinese patients with ankylosing spondylitis and non-ankylosing spondylitis carriers. J Int Med Res. 2019 Jul; 47(7):3171-3178.

56. Lopez-Larrea C, Gonzalez-Roces S, Pena M, Dominguez O, Coto E, Alvarez V, et al. Characterization of B27 haplotypes by oligotyping and genomic sequencing in the Mexican Mestizo population with ankylosing spondylitis: juvenile and adult onset. Hum Immunol. 1995 Jul; 43(3):174-180.

57. Lodhi NA, Bashir MM, Tipu HN, Hussain M. Distribution of HLA-B*27 Subtypes in Patients with Ankylosing Spondylitis in Local Population. J Coll Physicians Surg Pak. 2019 May; 29(5):418-421.

58. Liu X, Hu LH, Li YR, Chen FH, Ning Y, Yao QF. The association of HLA-B*27 subtypes with ankylosing spondylitis in Wuhan population of China. Rheumatol Int. 2010 Mar; 30(5):587-590.

59. Kchir MM, Hamdi W, Laadhar L, Kochbati S, Kaffel D, Saadellaoui K, et al. HLA-B, DR and DQ antigens polymorphism in Tunisian patients with ankylosing spondylitis (a case-control study). Rheumatol Int. 2010 May; 30(7):933-939.

60. Hou TY, Chen HC, Chen CH, Chang DM, Liu FC, Lai JH. Usefulness of human leucocyte antigen-B27 subtypes in predicting ankylosing spondylitis: Taiwan experience. Intern Med J. 2007 Nov; 37(11):749-752.

61. Harfouch EI, Al-Cheikh SA. HLA-B27 and its subtypes in Syrian patients with ankylosing spondylitis. Saudi Med J. 2011 Apr; 32(4):364-368.

62. Gonzalez-Roces S, Alvarez MV, Gonzalez S, Dieye A, Makni H, Woodfield DG, et al. HLA-B27 polymorphism and worldwide susceptibility to ankylosing spondylitis. Tissue Antigens. 1997 Feb; 49(2):116-123.

63. Diyarbakir E, Eyerci N, Melikoglu M, Topcu A, Pirim I. HLA B27 subtype distribution among patients with ankylosing spondylitis in eastern Turkey. Genet Test Mol Biomarkers. 2012 May; 16(5):456-458.

64. Cipriani A, Rivera S, Hassanhi M, Marquez G, Hernandez R, Villalobos C, et al. HLA-B27 subtypes determination in patients with ankylosing spondylitis from Zulia, Venezuela. Hum Immunol. 2003 Jul; 64(7):745-749.

65. Chou CT, Chen JM, Hsu CM, Chen SJ. HLA-B27 and its subtypes in 4 Taiwanese Aborigine tribes: a comparison to Han Chinese patients with ankylosing spondylitis. J Rheumatol. 2003 Feb; 30(2):321-325.

66. Chavan H, Samant R, Deshpande A, Mankeshwar R. Correlation of HLA B27 subtypes with clinical features of ankylosing spondylitis. Int J Rheum Dis. 2011 Oct; 14(4):369-374.

67. Birinci A, Bilgici A, Kuru O, Durupinar B. HLA-B27 polymorphism in Turkish patients with ankylosing spondylitis. Rheumatol Int. 2006 Feb; 26(4):285-287.

68. Ben Radhia K, Ayed-Jendoubi S, Sfar I, Ben Romdhane T, Makhlouf M, Gorgi Y, et al. Distribution of HLA-B*27 subtypes in Tunisians and their association with ankylosing spondylitis. Joint Bone Spine. 2008 Mar; 75(2):172-175.

69. Alaez C, Gazit E, Ibarrola B, Yaron M, Livneh A, Avishai O, et al. Distribution of HLA-B27 subtypes in ankylosing spondylitis in an Israeli population. Arch Med Res. 2007 May; 38(4):452-455.

70. Acar M, Cora T, Tunc R, Acar H. HLA-B27 subtypes in Turkish patients with ankylosing spondylitis and healthy controls. Rheumatol Int. 2012 Oct; 32(10):3103-3105.

71. Varnavidou-Nicolaidou A, Karpasitou K, Georgiou D, Stylianou G, Kokkofitou A, Michalis C, et al. HLA-B27 in the Greek Cypriot population: distribution of subtypes in patients with ankylosing spondylitis and other HLA-B27-related diseases. The possible protective role of B*2707. Hum Immunol. 2004 Dec; 65(12):1451-1454.

72. Lopez-Larrea C, Blanco-Gelaz MA, Torre-Alonso JC, Bruges Armas J, Suarez-Alvarez B, Pruneda L, et al. Contribution of KIR3DL1/3DS1 to ankylosing spondylitis in human leukocyte antigen-B27 Caucasian populations. Arthritis Res Ther. 2006; 8(4):R101.

73. Gonzalez S, Garcia-Fernandez S, Martinez-Borra J, Blanco-Gelaz MA, Rodrigo L, Sanchez del Rio J, et al. High variability of HLA-B27 alleles in ankylosing spondylitis and related spondyloarthropathies in the population of northern Spain. Hum Immunol. 2002 Aug; 63(8):673-676.

74. Diaz-Pena R, Vidal-Castineira JR, Lopez-Vazquez A, Lopez-Larrea C. HLA-B*40:01 Is Associated with Ankylosing Spondylitis in HLA-B27-positive Populations. J Rheumatol. 2016 Jun; 43(6):1255-1256.

75. Chen IH, Yang KL, Lee A, Huang HH, Lin PY, Lee TD. Low frequency of HLA-B*2706 in Taiwanese patients with ankylosing spondylitis. Eur J Immunogenet. 2002 Oct; 29(5):435-438.

76. Yamaguchi A, Ogawa A, Tsuchiya N, Shiota M, Mitsui H, Tokunaga K, et al. HLA-B27 subtypes in Japanese with seronegative spondyloarthropathies and healthy controls. J Rheumatol. 1996 Jul; 23(7):1189-1193.

77. Wu X, Wu J, Li X, Wei Q, Lv Q, Zhang P, et al. The Clinical Characteristics of Other HLA-B Types in Chinese Ankylosing Spondylitis Patients. Front Med (Lausanne). 2020; 7:568790.

78. Van Gaalen FA. Does HLA-B*2706 protect against ankylosing spondylitis? A meta-analysis. Int J Rheum Dis. 2012 Feb; 15(1):8-12.

79. Oguz FS, Ocal L, Diler AS, Ozkul H, Asicioglu F, Kasapoglu E, et al. HLA B-27 subtypes in Turkish patients with spondyloarthropathy and healthy controls. Dis Markers. 2004; 20(6):309-312.

80. Mou Y, Zhang P, Li Q, Lin Z, Liao Z, Wei Q, et al. Clinical Features in Juvenile-Onset Ankylosing Spondylitis Patients Carrying Different B27 Subtypes. Biomed Res Int. 2015; 2015:594878.

81. Lopez-Larrea C, Sujirachato K, Mehra NK, Chiewsilp P, Isarangkura D, Kanga U, et al. HLA-B27 subtypes in Asian patients with ankylosing spondylitis. Evidence for new associations. Tissue Antigens. 1995 Mar; 45(3):169-176.

82. Kanga U, Mehra NK, Larrea CL, Lardy NM, Kumar A, Feltkamp TE. Seronegative spondyloarthropathies and HLA-B27 subtyes: a study in Asian Indians. Clin Rheumatol. 1996 Jan; 15 Suppl 1:13-18.

83. Fouladi S, Adib M, Salehi M, Karimzadeh H, Bakhshiani Z, Ostadi V. Distribution of HLA-B*27 alleles in patients with ankylosing spondylitis in Iran. Iran J Immunol. 2009 Mar; 6(1):49-54.

84. Liu Y, Jiang L, Cai Q, Danoy P, Barnardo MC, Brown MA, et al. Predominant association of HLA-B*2704 with ankylosing spondylitis in Chinese Han patients. Tissue Antigens. 2010 Jan; 75(1):61-64.

85. Yang T, Duan Z, Wu S, Liu S, Zeng Z, Li G, et al. Association of HLA-B27 genetic polymorphisms with ankylosing spondylitis susceptibility worldwide: a meta-analysis. Mod Rheumatol. 2014 Jan; 24(1):150-161.

86. Wellcome Trust Case Control C, Australo-Anglo-American Spondylitis C, Burton PR, Clayton DG, Cardon LR, Craddock N, et al. Association scan of 14,500 nonsynonymous SNPs in four diseases identifies autoimmunity variants. Nat Genet. 2007 Nov; 39(11):1329-1337.

87. Australo-Anglo-American Spondyloarthritis C, Reveille JD, Sims AM, Danoy P, Evans DM, Leo P, et al. Genome-wide association study of ankylosing spondylitis identifies non-MHC susceptibility loci. Nat Genet. 2010 Feb; 42(2):123-127.

88. Li Z, Wu X, Leo PJ, De Guzman E, Akkoc N, Breban M, et al. Polygenic Risk Scores have high diagnostic capacity in ankylosing spondylitis. Ann Rheum Dis. 2021 Sep; 80(9):1168-1174.

89. Huang XF, Li Z, De Guzman E, Robinson P, Gensler L, Ward MM, et al. Genomewide Association Study of Acute Anterior Uveitis Identifies New Susceptibility Loci. Invest Ophthalmol Vis Sci. 2020 Jun 3; 61(6):3.

90. International Genetics of Ankylosing Spondylitis C, Cortes A, Hadler J, Pointon JP, Robinson PC, Karaderi T, et al. Identification of multiple risk variants for ankylosing spondylitis through high-density genotyping of immune-related loci. Nat Genet. 2013 Jul; 45(7):730-738.

91. Zhang Z, Dai D, Yu K, Yuan F, Jin J, Ding L, et al. Association of HLA-B27 and ERAP1 with ankylosing spondylitis susceptibility in Beijing Han Chinese. Tissue Antigens. 2014 May; 83(5):324-329.

92. Daryabor G, Mahmoudi M, Jamshidi A, Nourijelyani K, Amirzargar A, Ahmadzadeh N, et al. Determination of IL-23 receptor gene polymorphism in Iranian patients with ankylosing spondylitis. Eur Cytokine Netw. 2014 Mar 1; 25(1):24-29.

93. Wisniewski A, Kasprzyk S, Majorczyk E, Nowak I, Wilczynska K, Chlebicki A, et al. ERAP1-ERAP2 haplotypes are associated with ankylosing spondylitis in Polish patients. Hum Immunol. 2019 May; 80(5):339-343.

94. Robinson PC, Leo PJ, Pointon JJ, Harris J, Cremin K, Bradbury LA, et al. Exome-wide study of ankylosing spondylitis demonstrates additional shared genetic background with inflammatory bowel disease. NPJ Genom Med. 2016; 1:16008.

95. Soomro M, Stadler M, Dand N, Bluett J, Jadon D, Jalali-Najafabadi F, et al. Comparative Genetic Analysis of Psoriatic Arthritis and Psoriasis for the Discovery of Genetic Risk Factors and Risk Prediction Modeling. Arthritis Rheumatol. 2022 Sep; 74(9):1535-1543.

96. Bettencourt BF, Rocha FL, Alves H, Amorim R, Caetano-Lopes J, Vieira-Sousa E, et al. Protective effect of an ERAP1 haplotype in ankylosing spondylitis: investigating non-MHC genes in HLA-B27-positive individuals. Rheumatology (Oxford). 2013 Dec; 52(12):2168-2176.

97. Lin Z, Bei JX, Shen M, Li Q, Liao Z, Zhang Y, et al. A genome-wide association study in Han Chinese identifies new susceptibility loci for ankylosing spondylitis. Nat Genet. 2011 Dec 4; 44(1):73-77.

98. Zvyagin IV, Dorodnykh VY, Mamedov IZ, Staroverov DB, Bochkova AG, Rebrikov DV, et al. Association of ERAP1 Allelic Variants with Risk of Ankylosing Spondylitis. Acta Naturae. 2010 Jul; 2(3):72-77.

99. Zhang L, Fan D, Liu L, Yang T, Ding N, Hu Y, et al. Association Study of IL-12B Polymorphisms Susceptibility with Ankylosing Spondylitis in Mainland Han Population. PLoS One. 2015; 10(6):e0130982.

100. Rostami S, Hoff M, Brown MA, Hveem K, Holmen OL, Fritsche LG, et al. Prediction of Ankylosing Spondylitis in the HUNT Study by a Genetic Risk Score Combining 110 Single-nucleotide Polymorphisms of Genome-wide Significance. J Rheumatol. 2020 Feb; 47(2):204-210.

101. Jung SH, Cho SM, Yim SH, Kim SH, Park HC, Cho ML, et al. Developing a Risk-scoring Model for Ankylosing Spondylitis Based on a Combination of HLA-B27, Single-nucleotide Polymorphism, and Copy Number Variant Markers. J Rheumatol. 2016 Dec; 43(12):2136-2141.

102. Thomas GP, Willner D, Robinson PC, Cortes A, Duan R, Rudwaleit M, et al. Genetic diagnostic profiling in axial spondyloarthritis: a real world study. Clin Exp Rheumatol. 2017 Mar-Apr; 35(2):229-233.

103. Riechers E, Baerlecken N, Baraliakos X, Achilles-Mehr Bakhsh K, Aries P, Bannert B, et al. Sensitivity and Specificity of Autoantibodies Against CD74 in Nonradiographic Axial Spondyloarthritis. Arthritis Rheumatol. 2019 May; 71(5):729-735.

104. de Winter JJ, van de Sande MG, Baerlecken N, Berg I, Ramonda R, van der Heijde D, et al. Anti-CD74 antibodies have no diagnostic value in early axial spondyloarthritis: data from the spondyloarthritis caught early (SPACE) cohort. Arthritis Res Ther. 2018 Mar 1; 20(1):38.

105. Tsui FW, Tsui HW, Las Heras F, Pritzker KP, Inman RD. Serum levels of novel noggin and sclerostin-immune complexes are elevated in ankylosing spondylitis. Ann Rheum Dis. 2014 Oct; 73(10):1873-1879.

106. Baraliakos X, Baerlecken N, Witte T, Heldmann F, Braun J. High prevalence of anti-CD74 antibodies specific for the HLA class II-associated invariant chain peptide (CLIP) in patients with axial spondyloarthritis. Ann Rheum Dis. 2014 Jun; 73(6):1079-1082.

107. Baerlecken NT, Nothdorft S, Stummvoll GH, Sieper J, Rudwaleit M, Reuter S, et al. Autoantibodies against CD74 in spondyloarthritis. Ann Rheum Dis. 2014 Jun; 73(6):1211-1214.

108. Abdelaziz MM, Gamal RM, Ismail NM, Lafy RA, Hetta HF. Diagnostic value of anti-CD74 antibodies in early and late axial spondyloarthritis and its relationship to disease activity. Rheumatology (Oxford). 2021 Jan 5; 60(1):263-268.

109. Do L, Granasen G, Hellman U, Lejon K, Geijer M, Baraliakos X, et al. Anti-CD74 IgA autoantibodies in radiographic axial spondyloarthritis: a longitudinal Swedish study. Rheumatology (Oxford). 2021 Sep 1; 60(9):4085-4093.

110. Riechers E, Baerlecken N, Baraliakos X, Achilles-Mehr Bakhsh K, Aries P, Bannert B, et al. Sensitivity and Specificity of Autoantibodies Against CD74 in Nonradiographic Axial Spondyloarthritis. Arthritis Rheumatol. 2019 May; 71(5):729-735.

111. Hu CJ, Li MT, Li X, Peng LY, Zhang SZ, Leng XM, et al. CD74 auto-antibodies display little clinical value in Chinese Han population with axial spondyloarthritis. Medicine (Baltimore). 2020 Dec 11; 99(50):e23433.

112. Ziade NR, Mallak I, Merheb G, Ghorra P, Baerlecken N, Witte T, et al. Added Value of Anti-CD74 Autoantibodies in Axial SpondyloArthritis in a Population With Low HLA-B27 Prevalence. Front Immunol. 2019; 10:574.

113. Colak S, Turgay TM, Kucuksahin O, Duman MT, Cetinkaya H, Toruner M. The association of anti-CD74 antibody with spondyloarthropathies. Eur J Rheumatol. 2021 Oct; 8(4):207-210.

114. Liu Y, Liao X, Shi G. Autoantibodies in Spondyloarthritis, Focusing on Anti-CD74 Antibodies. Front Immunol. 2019; 10:5.

115. Rosenberg JN, Johnson GD, Holborow EJ. Antinuclear antibodies in ankylosing spondylitis, psoriatic arthritis, and psoriasis. Ann Rheum Dis. 1979 Dec; 38(6):526-528.

116. de Vries M, van der Horst-Bruinsma I, van Hoogstraten I, van Bodegraven A, von Blomberg BM, Ratnawati H, et al. pANCA, ASCA, and OmpC antibodies in patients with ankylosing spondylitis without inflammatory bowel disease. J Rheumatol. 2010 Nov; 37(11):2340-2344.

117. Matzkies FG, Targan SR, Berel D, Landers CJ, Reveille JD, McGovern DP, et al. Markers of intestinal inflammation in patients with ankylosing spondylitis: a pilot study. Arthritis Res Ther. 2012 Nov 29; 14(6):R261.

118. Wallis D, Asaduzzaman A, Weisman M, Haroon N, Anton A, McGovern D, et al. Elevated serum anti-flagellin antibodies implicate subclinical bowel inflammation in ankylosing spondylitis: an observational study. Arthritis Research & Therapy. 2013; 15(5).

119. Stone MA, Payne U, Schentag C, Rahman P, Pacheco-Tena C, Inman RD. Comparative immune responses to candidate arthritogenic bacteria do not confirm a dominant role for Klebsiella pneumonia in the pathogenesis of familial ankylosing spondylitis. Rheumatology (Oxford). 2004 Feb; 43(2):148-155.

120. Stebbings S, Munro K, Simon MA, Tannock G, Highton J, Harmsen H, et al. Comparison of the faecal microflora of patients with ankylosing spondylitis and controls using molecular methods of analysis. Rheumatology (Oxford). 2002 Dec; 41(12):1395-1401.

121. Tiwana H, Wilson C, Walmsley RS, Wakefield AJ, Smith MS, Cox NL, et al. Antibody responses to gut bacteria in ankylosing spondylitis, rheumatoid arthritis, Crohn's disease and ulcerative colitis. Rheumatol Int. 1997; 17(1):11-16.

122. Collado A, Gratacos J, Ebringer A, Rashid T, Marti A, Sanmarti R, et al. Serum IgA anti-Klebsiella antibodies in ankylosing spondylitis patients from Catalonia. Scand J Rheumatol. 1994; 23(3):119-123.

123. Cooper R, Fraser SM, Sturrock RD, Gemmell CG. Raised titres of anti-klebsiella IgA in ankylosing spondylitis, rheumatoid arthritis, and inflammatory bowel disease. Br Med J (Clin Res Ed). 1988 May 21; 296(6634):1432-1434.

124. Csango PA, Upsahl MT, Romberg O, Kornstad L, Sarov I. Chlamydia trachomatis serology in ankylosing spondylitis. Clin Rheumatol. 1987 Sep; 6(3):384-390.

125. Kihlstrom E, Gronberg A, Bengtsson A. Immunoblot analysis of antibody response to Chlamydia trachomatis in patients with reactive arthritis and ankylosing spondylitis. Scand J Rheumatol. 1989; 18(6):377-383.

126. Kumar P, Bhakuni DS, Rastogi S. Diagnosis of Chlamydia trachomatis in patients with reactive arthritis and undifferentiated spondyloarthropathy. J Infect Dev Ctries. 2014 May 14; 8(5):648-654.

127. Tsuchiya N, Husby G, Williams RC, Jr. Antibodies to the peptide from the plasmid-coded Yersinia outer membrane protein (YOP1) in patients with ankylosing spondylitis. Clin Exp Immunol. 1990 Dec; 82(3):493-498.

128. Zambrano-Zaragoza JF, de Jesus Duran-Avelar M, Rodriguez-Ocampo AN, Garcia-Latorre E, Burgos-Vargas R, Dominguez-Lopez ML, et al. The 30-kDa band from Salmonella typhimurium: IgM, IgA and IgG antibody response in patients with ankylosing spondylitis. Rheumatology (Oxford). 2009 Jul; 48(7):748-754.

129. Andretta MA, Vieira TD, Nishiara R, Skare TL. Anti-Saccharomyces cerevisiae (ASCA) and anti-endomysial antibodies in spondyloarthritis. Rheumatol Int. 2012 Feb; 32(2):551-554.

130. Aydin SZ, Atagunduz P, Temel M, Bicakcigil M, Tasan D, Direskeneli H. Anti-Saccharomyces cerevisiae antibodies (ASCA) in spondyloarthropathies: a reassessment. Rheumatology (Oxford). 2008 Feb; 47(2):142-144.

131. Hoffman IE, Demetter P, Peeters M, De Vos M, Mielants H, Veys EM, et al. Anti-saccharomyces cerevisiae IgA antibodies are raised in ankylosing spondylitis and undifferentiated spondyloarthropathy. Ann Rheum Dis. 2003 May; 62(5):455-459.

132. Mundwiler ML, Mei L, Landers CJ, Reveille JD, Targan S, Weisman MH. Inflammatory bowel disease serologies in ankylosing spondylitis patients: a pilot study. Arthritis Res Ther. 2009; 11(6):R177.

133. Rodrigues IK, Andrigueti M, de Oliveira Gil ID, de Lucca Schiavon L, de Andrade KR, Pereira IA, et al. An investigation into the relationship between anti-Helicobacter pylori and anti-Saccharomyces cerevisiae antibodies in patients with axial spondyloarthritis and Crohn disease. Rheumatol Int. 2015 Feb; 35(2):359-366.

134. Torok HP, Glas J, Gruber R, Brumberger V, Strasser C, Kellner H, et al. Inflammatory bowel disease-specific autoantibodies in HLA-B27-associated spondyloarthropathies: increased prevalence of ASCA and pANCA. Digestion. 2004; 70(1):49-54.

135. Tani Y, Sato H, Tanaka N, Hukuda S. Antibodies against bacterial lipopolysaccharides in Japanese patients with ankylosing spondylitis. Br J Rheumatol. 1997 Apr; 36(4):491-493.

136. Dominguez-Lopez ML, Burgos-Vargas R, Galicia-Serrano H, Bonilla-Sanchez MT, Rangel-Acosta HH, Cancino-Diaz ME, et al. IgG antibodies to enterobacteria 60 kDa heat shock proteins in the sera of HLA-B27 positive ankylosing spondylitis patients. Scand J Rheumatol. 2002; 31(5):260-265.

137. Maki-Ikola O, Leirisalo-Repo M, Turunen U, Granfors K. Association of gut inflammation with increased serum IgA class Klebsiella antibody concentrations in patients with axial ankylosing spondylitis (AS): implication for different aetiopathogenetic mechanisms for axial and peripheral AS? Ann Rheum Dis. 1997 Mar; 56(3):180-183.

138. Lee JS, Lee EJ, Lee JH, Hong SC, Lee CK, Yoo B, et al. Autoantibodies against Protein Phosphatase Magnesium-Dependent 1A as a Biomarker for Predicting Radiographic Progression in Ankylosing Spondylitis Treated with Anti-Tumor Necrosis Factor Agents. J Clin Med. 2020 Dec 7; 9(12).

139. Kim YG, Sohn DH, Zhao X, Sokolove J, Lindstrom TM, Yoo B, et al. Role of protein phosphatase magnesium-dependent 1A and anti-protein phosphatase magnesium-dependent 1A autoantibodies in ankylosing spondylitis. Arthritis Rheumatol. 2014 Oct; 66(10):2793-2803.

140. Dougados M, Gueguen A, Nakache JP, Velicitat P, Zeidler H, Veys E, et al. Clinical relevance of C-reactive protein in axial involvement of ankylosing spondylitis. J Rheumatol. 1999 Apr; 26(4):971-974.

141. Benhamou M, Gossec L, Dougados M. Clinical relevance of C-reactive protein in ankylosing spondylitis and evaluation of the NSAIDs/coxibs' treatment effect on C-reactive protein. Rheumatology (Oxford). 2010 Mar; 49(3):536-541.

142. Akbal A, Resorlu H, Gokmen F, Savas Y, Zateri C, Sargin B, et al. The relationship between C-reactive protein rs3091244 polymorphism and ankylosing spondylitis. Int J Rheum Dis. 2016 Jan; 19(1):43-48.

143. Su J, Cui L, Yang W, Shi H, Jin C, Shu R, et al. Baseline high-sensitivity C-reactive protein predicts the risk of incident ankylosing spondylitis: Results of a community-based prospective study. PLoS One. 2019; 14(2):e0211946.

144. Kilic E, Kilic G, Akgul O, Ozgocmen S. Discriminant validity of the Ankylosing Spondylitis Disease Activity Score (ASDAS) in patients with non-radiographic axial spondyloarthritis and ankylosing spondylitis: a cohort study. Rheumatol Int. 2015 Jun; 35(6):981-989.

145. Li HG, Wang DM, Shen FC, Huang SX, Hou ZD, Lin L, et al. Risk factors for progression of juvenile-onset non-radiographic axial spondyloarthritis to juvenile-onset ankylosing spondylitis: A nested case-control study. RMD Open. 2021; 7(3).

146. Huang Y, Deng W, Pan X, Liu M, Zhong Z, Huang Q, et al. The relationship between platelet to albumin ratio and disease activity in axial spondyloarthritis patients. Mod Rheumatol. 2022 Aug 20; 32(5):974-979.

147. Tang Y, Yang P, Wang F, Xu H, Zong SY. Association of polymorphisms in ERAP1 and risk of ankylosing spondylitis in a Chinese population. Gene. 2018 Mar 10; 646:8-11.

148. Sun X, Zhou C, Zhu J, Wu S, Liang T, Jiang J, et al. Identification of clinical heterogeneity and construction of a novel subtype predictive model in patients with ankylosing spondylitis: An unsupervised machine learning study. Int Immunopharmacol. 2023 Apr; 117:109879.

149. Wang F, Yan CG, Xiang HY, Xing T, Wang NS. The significance of platelet activation in ankylosing spondylitis. Clin Rheumatol. 2008 Jun; 27(6):767-769.

150. Ho KJ, Chen PQ, Chang CY, Lu FJ. The oxidative metabolism of circulating phagocytes in ankylosing spondylitis: determination by whole blood chemiluminescence. Ann Rheum Dis. 2000 May; 59(5):338-341.

151. Ozgocmen S, Godekmerdan A, Ozkurt-Zengin F. Acute-phase response, clinical measures and disease activity in ankylosing spondylitis. Joint Bone Spine. 2007 May; 74(3):249-253.

152. Kang KY, Kim IJ, Jung SM, Kwok SK, Ju JH, Park KS, et al. Incidence and predictors of morphometric vertebral fractures in patients with ankylosing spondylitis. Arthritis Res Ther. 2014 Jun 16; 16(3):R124.

153. Komsalova LY, Martinez Salinas MP, Jimenez JFG. Predictive values of inflammatory back pain, positive HLA B27 antigen and acute and chronic magnetic resonance changes in early diagnosis of Spondyloarthritis. A study of 133 patients. PLoS One. 2020; 15(12):e0244184.

154. Naziroglu M, Akkus S, Celik H. Levels of lipid peroxidation and antioxidant vitamins in plasma and erythrocytes of patients with ankylosing spondylitis. Clin Biochem. 2011 Dec; 44(17-18):1412-1415.

155. Zwolak R, Suszek D, Graca A, Mazurek M, Majdan M. Reasons for diagnostic delays of axial spondyloarthritis. Wiad Lek. 2019; 72(9 cz 1):1607-1610.

156. Sahli H, Bachali A, Tekaya R, Mahmoud I, Sedki Y, Saidane O, et al. Involvement of foot in patients with spondyloarthritis: Prevalence and clinical features. Foot Ankle Surg. 2019 Apr; 25(2):226-230.

157. Hirano F, Landewe RBM, van Gaalen FA, van der Heijde D, Gaujoux-Viala C, Ramiro S. Determinants of the Physician Global Assessment of Disease Activity and Influence of Contextual Factors in Early Axial Spondyloarthritis. Arthritis Care Res (Hoboken). 2022 Feb; 74(2):268-273.

158. Poddubnyy D, Rudwaleit M, Haibel H, Listing J, Marker-Hermann E, Zeidler H, et al. Rates and predictors of radiographic sacroiliitis progression over 2 years in patients with axial spondyloarthritis. Ann Rheum Dis. 2011 Aug; 70(8):1369-1374.

159. Navarini L, Caso F, Costa L, Currado D, Stola L, Perrotta F, et al. Cardiovascular Risk Prediction in Ankylosing Spondylitis: From Traditional Scores to Machine Learning Assessment. Rheumatol Ther. 2020 Dec; 7(4):867-882.

160. Kim SH, Kim KH, Kim MY, Hong YS, Kang KY. A 2-year longitudinal study of bone health in adolescent patients with axial spondyloarthritis. Arch Osteoporos. 2021 Jan 8; 16(1):12.

161. Braga MV, de Oliveira SC, Vasconcelos AHC, Lopes JR, de Macedo Filho CL, Ramos LMA, et al. Prevalence of sacroiliitis and acute and structural changes on MRI in patients with psoriatic arthritis. Sci Rep. 2020 Jul 14; 10(1):11580.

162. Chen HA, Chen CH, Liao HT, Lin YJ, Chen PC, Chen WS, et al. Factors associated with radiographic spinal involvement and hip involvement in ankylosing spondylitis. Semin Arthritis Rheum. 2011 Jun; 40(6):552-558.

163. Wu J, Yan L, Chai K. Systemic immune-inflammation index is associated with disease activity in patients with ankylosing spondylitis. J Clin Lab Anal. 2021 Sep; 35(9):e23964.

164. Kwan YH, Tan JJ, Phang JK, Fong W, Lim KK, Koh HL, et al. Validity and reliability of the Ankylosing Spondylitis Disease Activity Score with C-reactive protein (ASDAS-CRP) and Bath Ankylosing Spondylitis Disease Activity Index (BASDAI) in patients with axial spondyloarthritis (axSpA) in Singapore. Int J Rheum Dis. 2019 Dec; 22(12):2206-2212.

165. Sundaram TG, Muhammed H, Aggarwal A, Gupta L. A prospective study of novel disease activity indices for ankylosing spondylitis. Rheumatol Int. 2020 Nov; 40(11):1843-1849.

166. Navarini L, Currado D, Marino A, Di Donato S, Biaggi A, Caso F, et al. Persistence of C-reactive protein increased levels and high disease activity are predictors of cardiovascular disease in patients with axial spondyloarthritis. Sci Rep. 2022 May 7; 12(1):7498.

167. Cowling P, Ebringer R, Cawdell D, Ishii M, Ebringer A. C-reactive protein, ESR, and klebsiella in ankylosing spondylitis. Ann Rheum Dis. 1980 Feb; 39(1):45-49.

168. Bedaiwi MK, AlRasheed RF, Bin Zuair A, Alqurtas EM, Baeshen MO, Omair MA. A cross-sectional study on clinical characteristics of Saudi axial spondylarthritis: preliminary results. Eur Rev Med Pharmacol Sci. 2021 Aug; 25(16):5241-5247.

169. Chen CH, Chen HA, Liao HT, Liu CH, Tsai CY, Chou CT. The clinical usefulness of ESR, CRP, and disease duration in ankylosing spondylitis: the product of these acute-phase reactants and disease duration is associated with patient's poor physical mobility. Rheumatol Int. 2015 Jul; 35(7):1263-1267.

170. Siebuhr AS, Husakova M, Forejtova S, Zegzulkova K, Tomcik M, Urbanova M, et al. Metabolites of C-reactive protein and vimentin are associated with disease activity of axial spondyloarthritis. Clin Exp Rheumatol. 2019 May-Jun; 37(3):358-366.

171. Senna MK, Olama SM, El-Arman M. Serum melatonin level in ankylosing spondylitis: is it increased in active disease? Rheumatol Int. 2012 Nov; 32(11):3429-3433.

172. Toldi G, Szalay B, Beko G, Kovacs L, Vasarhelyi B, Balog A. Plasma soluble urokinase plasminogen activator receptor (suPAR) levels in ankylosing spondylitis. Joint Bone Spine. 2013 Jan; 80(1):96-98.

173. Mlcoch T, Sedova L, Stolfa J, Urbanova M, Suchy D, Smrzova A, et al. Mapping the relationship between clinical and quality-of-life outcomes in patients with ankylosing spondylitis. Expert Rev Pharmacoecon Outcomes Res. 2017 Apr; 17(2):203-211.

174. Seng JJB, Kwan YH, Low LL, Thumboo J, Fong WSW. Role of neutrophil to lymphocyte ratio (NLR), platelet to lymphocyte ratio (PLR) and mean platelet volume (MPV) in assessing disease control in Asian patients with axial spondyloarthritis. Biomarkers. 2018 May-Jun; 23(4):335-338.

175. Londono J, Romero-Sanchez MC, Torres VG, Bautista WA, Fernandez DJ, Quiroga JdA, et al. The association between serum levels of potential biomarkers with the presence of factors related to the clinical activity and poor prognosis in spondyloarthritis. Rev Bras Reumatol. 2012 Aug; 52(4):536-544.

176. Bansal N, Duggal L, Jain N. Validity of Simplified Ankylosing Spondylitis Disease Activity Scores (SASDAS) in Indian Ankylosing Spondylitis Patients. J Clin Diagn Res. 2017 Sep; 11(9):OC06-OC09.

177. Yildirim K, Erdal A, Karatay S, Melikoglu MA, Ugur M, Senel K. Relationship between some acute phase reactants and the Bath Ankylosing Spondylitis Disease Activity Index in patients with ankylosing spondylitis. South Med J. 2004 Apr; 97(4):350-353.

178. Wendling D, Guillot X, Gossec L, Prati C, Saraux A, Dougados M. Remission is related to CRP and smoking in early axial spondyloarthritis. The DESIR cohort. Joint Bone Spine. 2017 Jul; 84(4):473-476.

179. Ben-Shabat N, Shabat A, Watad A, Kridin K, Bragazzi NL, McGonagle D, et al. Mortality in Ankylosing Spondylitis According to Treatment: A Nationwide Retrospective Cohort Study of 5,900 Patients From Israel. Arthritis Care Res (Hoboken). 2022 Oct; 74(10):1614-1622.

180. de Vries MK, van Eijk IC, van der Horst-Bruinsma IE, Peters MJ, Nurmohamed MT, Dijkmans BA, et al. Erythrocyte sedimentation rate, C-reactive protein level, and serum amyloid a protein for patient selection and monitoring of anti-tumor necrosis factor treatment in ankylosing spondylitis. Arthritis Rheum. 2009 Nov 15; 61(11):1484-1490.

181. Iervolino S, Di Minno MN, Peluso R, Lofrano M, Russolillo A, Di Minno G, et al. Predictors of early minimal disease activity in patients with psoriatic arthritis treated with tumor necrosis factor-alpha blockers. J Rheumatol. 2012 Mar; 39(3):568-573.

182. Li T, Liu Y, Sheng R, Yin J, Wu X, Xu H. Correlation Between Chronic Pain Acceptance and Clinical Variables in Ankylosing Spondylitis and Its Prediction Role for Biologics Treatment. Frontiers in Medicine. 2020; 7.

183. Sebastian A, Wojtala P, Lubinski L, Mimier M, Chlebicki A, Wiland P. Disease activity in axial spondyloarthritis after discontinuation of TNF inhibitors therapy. Reumatologia. 2017; 55(4):157-162.

184. Zong HX, Xu SQ, Wang JX, Chu YR, Chen KM, Wang C, et al. Presence of subclinical inflammation in axial spondyloarthritis patients with NSAID/anti-TNF-alpha drug-induced clinical remission. Clin Rheumatol. 2022 May; 41(5):1403-1412.

185. Webers C, Essers I, Ramiro S, Stolwijk C, Landewe R, van der Heijde D, et al. Gender-attributable differences in outcome of ankylosing spondylitis: long-term results from the Outcome in Ankylosing Spondylitis International Study. Rheumatology (Oxford). 2016 Mar; 55(3):419-428.

186. Syrbe U, Callhoff J, Conrad K, Poddubnyy D, Haibel H, Junker S, et al. Serum adipokine levels in patients with ankylosing spondylitis and their relationship to clinical parameters and radiographic spinal progression. Arthritis Rheumatol. 2015 Mar; 67(3):678-685.

187. Kang KY, Kim IJ, Yoon MA, Hong YS, Park SH, Ju JH. Fat Metaplasia on Sacroiliac Joint Magnetic Resonance Imaging at Baseline Is Associated with Spinal Radiographic Progression in Patients with Axial Spondyloarthritis. PLoS One. 2015; 10(8):e0135206.

188. Poddubnyy D, Protopopov M, Haibel H, Braun J, Rudwaleit M, Sieper J. High disease activity according to the Ankylosing Spondylitis Disease Activity Score is associated with accelerated radiographic spinal progression in patients with early axial spondyloarthritis: results from the GErman SPondyloarthritis Inception Cohort. Ann Rheum Dis. 2016 Dec; 75(12):2114-2118.

189. Kim TJ, Shin JH, Kim S, Sung IH, Lee S, Song Y, et al. Radiographic progression in patients with ankylosing spondylitis according to tumor necrosis factor blocker exposure: Observation Study of Korean Spondyloarthropathy Registry (OSKAR) data. Joint Bone Spine. 2016 Oct; 83(5):569-572.

190. Kang KY, Kwok SK, Ju JH, Park KS, Park SH, Hong YS. The predictors of development of new syndesmophytes in female patients with ankylosing spondylitis. Scand J Rheumatol. 2015; 44(2):125-128.

191. Sohn DH, Jeong H, Roh JS, Lee HN, Kim E, Koh JH, et al. Serum CCL11 level is associated with radiographic spinal damage in patients with ankylosing spondylitis. Rheumatol Int. 2018 Aug; 38(8):1455-1464.

192. Deminger A, Klingberg E, Geijer M, Gothlin J, Hedberg M, Rehnberg E, et al. A five-year prospective study of spinal radiographic progression and its predictors in men and women with ankylosing spondylitis. Arthritis Res Ther. 2018 Aug 3; 20(1):162.

193. Pedersen SJ, Weber U, Said-Nahal R, Sorensen IJ, Loft AG, Kollerup G, et al. Structural progression rate decreases over time on serial radiography and magnetic resonance imaging of sacroiliac joints and spine in a five-year follow-up study of patients with ankylosing spondylitis treated with tumour necrosis factor inhibitor. Scand J Rheumatol. 2019 May; 48(3):185-197.

194. Jeong H, Bea EK, Lee J, Koh EM, Cha HS. Body mass index and estrogen predict radiographic progression in the spine in ankylosing spondylitis. Joint Bone Spine. 2015 Dec; 82(6):473-474.

195. Poddubnyy D, Haibel H, Listing J, Marker-Hermann E, Zeidler H, Braun J, et al. Baseline radiographic damage, elevated acute-phase reactant levels, and cigarette smoking status predict spinal radiographic progression in early axial spondylarthritis. Arthritis Rheum. 2012 May; 64(5):1388-1398.

196. Huerta-Sil G, Casasola-Vargas JC, Londono JD, Rivas-Ruiz R, Chavez J, Pacheco-Tena C, et al. Low grade radiographic sacroiliitis as prognostic factor in patients with undifferentiated spondyloarthritis fulfilling diagnostic criteria for ankylosing spondylitis throughout follow up. Ann Rheum Dis. 2006 May; 65(5):642-646.

197. Braun J, Baraliakos X, Hermann KG, Xu S, Hsu B. Serum C-reactive Protein Levels Demonstrate Predictive Value for Radiographic and Magnetic Resonance Imaging Outcomes in Patients with Active Ankylosing Spondylitis Treated with Golimumab. J Rheumatol. 2016 Sep; 43(9):1704-1712.

198. Kim MJ, Lee EB, Song YW, Park JK. Profile of common inflammatory markers in treatment-naive patients with systemic rheumatic diseases. Clin Rheumatol. 2020 Oct; 39(10):2899-2906.

199. Jung SY, Park MC, Park YB, Lee SK. Serum amyloid a as a useful indicator of disease activity in patients with ankylosing spondylitis. Yonsei Med J. 2007 Apr 30; 48(2):218-224.

200. Borman P, Bodur H, Bingol N, Bingol S, Bostan EE. Bone mineral density and bone turnover markers in a group of male ankylosing spondylitis patients: relationship to disease activity. J Clin Rheumatol. 2001 Oct; 7(5):315-321.

201. Hussein A, Stein J, Ehrich JH. C-reactive protein in the assessment of disease activity in juvenile rheumatoid arthritis and juvenile spondyloarthritis. Scand J Rheumatol. 1987; 16(2):101-105.

202. Sheehan NJ, Slavin BM, Donovan MP, Mount JN, Mathews JA. Lack of correlation between clinical disease activity and erythrocyte sedimentation rate, acute phase proteins or protease inhibitors in ankylosing spondylitis. Br J Rheumatol. 1986 May; 25(2):171-174.

203. Wang Z, Hu Y, Long HB. To Compare the Early Efficacy of Infliximab and Adalimumab for the Treatment of Ankylosing Spondylitis and their Impacts on Inflammatory Markers. Indian Journal of Pharmaceutical Sciences. 2022; 84(S1).

204. Alegre-Sancho JJ, Juanola X, Rodriguez-Heredia JM, Manero J, Villa-Blanco I, Laiz A, et al. Effectiveness and persistence of golimumab as a second biological drug in patients with spondyloarthritis: A retrospective study. Medicine (Baltimore). 2021 Apr 2; 100(13):e25223.

205. Zhang K, Zheng Y, Han Q, Liu Y, Wang W, Ding J, et al. The Clinical and MRI Effect of TNF-alpha Inhibitors in Spondyloarthritis Patients With Hip Involvement: A Real-World Observational Clinical Study. Front Immunol. 2021; 12:740980.

206. You Y, Cai M, Lin J, Liu L, Chen C, Wang Y, et al. Efficacy of needle-knife combined with etanercept treatment regarding disease activity and hip joint function in ankylosing spondylitis patients with hip joint involvement: A randomized controlled study. Medicine (Baltimore). 2020 May; 99(19):e20019.

207. Xu Y, Jiang W, Zhang H. Association between C-reactive protein gene variant and treatment efficacy of etanercept in ankylosing spondylitis patients receiving hip arthroplasty. J Clin Lab Anal. 2020 Aug; 34(8):e23343.

208. Dong Y, Guo J, Bi L. Baseline Interleukin-6 and Erythrocyte Sedimentation Rate Can Predict Clinical Response of TNF Inhibitor Treatment in Patients with Ankylosing Spondylitis. Ann Clin Lab Sci. 2019 Sep; 49(5):611-618.

209. Gentileschi S, Vitale A, Rigante D, Lopalco G, Emmi G, Orlando I, et al. Prompt Clinical Response to Secukinumab in Patients with Axial Spondyloarthritis: Real Life Observational Data from Three Italian Referral Centers. Isr Med Assoc J. 2018 Jul; 20(7):438-441.

210. Wei JC, Tsai WC, Citera G, Kotak S, Llamado L. Efficacy and safety of etanercept in patients from Latin America, Central Europe and Asia with early non-radiographic axial spondyloarthritis. Int J Rheum Dis. 2018 Jul; 21(7):1443-1451.

211. Lubrano E, Massimo Perrotta F, Manara M, D'Angelo S, Addimanda O, Ramonda R, et al. Predictors of Loss of Remission and Disease Flares in Patients with Axial Spondyloarthritis Receiving Antitumor Necrosis Factor Treatment: A Retrospective Study. J Rheumatol. 2016 Aug; 43(8):1541-1546.

212. Mok CC, Li OC, Chan KL, Ho LY, Hui PK. Effect of golimumab and pamidronate on clinical efficacy and MRI inflammation in axial spondyloarthritis: a 48-week open randomized trial. Scand J Rheumatol. 2015; 44(6):480-486.

213. Kneepkens EL, Wei JC, Nurmohamed MT, Yeo KJ, Chen CY, van der Horst-Bruinsma IE, et al. Immunogenicity, adalimumab levels and clinical response in ankylosing spondylitis patients during 24 weeks of follow-up. Ann Rheum Dis. 2015 Feb; 74(2):396-401.

214. Korkosz M, Gasowski J, Leszczynski P, Pawlak-Bus K, Jeka S, Siedlar M, et al. Effect of tumour necrosis factor-alpha inhibitor on serum level of dickkopf-1 protein and bone morphogenetic protein-7 in ankylosing spondylitis patients with high disease activity. Scand J Rheumatol. 2014; 43(1):43-48.

215. Paramarta JE, De Rycke L, Heijda TF, Ambarus CA, Vos K, Dinant HJ, et al. Efficacy and safety of adalimumab for the treatment of peripheral arthritis in spondyloarthritis patients without ankylosing spondylitis or psoriatic arthritis. Ann Rheum Dis. 2013 Nov; 72(11):1793-1799.

216. Sandhya P, Danda D, Mathew J, Gattani A. Outcome of patients with seronegative spondyloarthritis continuing sulphasalazine and methotrexate after a short course of infliximab therapy--experience from a tertiary care teaching hospital in South India. Clin Rheumatol. 2011 Jul; 30(7):997-1001.

217. Kim HR, Hong YS, Park SH, Ju JH, Kang KY. Low bone mineral density predicts the formation of new syndesmophytes in patients with axial spondyloarthritis. Arthritis Res Ther. 2018 Oct 16; 20(1):231.

218. Haroon N, Maksymowych WP, Rahman P, Tsui FW, O'Shea FD, Inman RD. Radiographic severity of ankylosing spondylitis is associated with polymorphism of the large multifunctional peptidase 2 gene in the Spondyloarthritis Research Consortium of Canada cohort. Arthritis Rheum. 2012 Apr; 64(4):1119-1126.

219. Hu QL, Fu S, Huang R, Zhang L, Wu LF, Lv YJ. The Value of Serum Amyloid A in the Diagnosis and Management of Ankylosing Spondylitis. Int J Gen Med. 2021; 14:2715-2719.

220. Lange U, Boss B, Teichmann J, Klor HU, Neeck G. Serum amyloid A--an indicator of inflammation in ankylosing spondylitis. Rheumatol Int. 2000; 19(4):119-122.

221. Liu S, Ji W, Lu J, Tang X, Guo Y, Ji M, et al. Discovery of Potential Serum Protein Biomarkers in Ankylosing Spondylitis Using Tandem Mass Tag-Based Quantitative Proteomics. J Proteome Res. 2020 Feb 7; 19(2):864-872.

222. Rademacher J, Tietz LM, Le L, Hartl A, Hermann KA, Sieper J, et al. Added value of biomarkers compared with clinical parameters for the prediction of radiographic spinal progression in axial spondyloarthritis. Rheumatology (Oxford). 2019 Sep 1; 58(9):1556-1564.

223. Londono J, Romero-Sanchez MC, Torres VG, Bautista WA, Fernandez DJ, Quiroga Jde A, et al. The association between serum levels of potential biomarkers with the presence of factors related to the clinical activity and poor prognosis in spondyloarthritis. Rev Bras Reumatol. 2012 Aug; 52(4):536-544.

224. Ostensen M, Marhaug G, Husby G. Amyloid-related serum protein (SAA) during and after pregnancy in healthy women and women with rheumatic disease. Acta Pathol Microbiol Immunol Scand C. 1985 Feb; 93(1):1-5.

225. van Eijk IC, de Vries MK, Levels JH, Peters MJ, Huizer EE, Dijkmans BA, et al. Improvement of lipid profile is accompanied by atheroprotective alterations in high-density lipoprotein composition upon tumor necrosis factor blockade: a prospective cohort study in ankylosing spondylitis. Arthritis Rheum. 2009 May; 60(5):1324-1330.

226. Yang J, Zhang X, Ma Y, Wu M, Hu X, Han R, et al. Serum levels of leptin, adiponectin and resistin in patients with ankylosing spondylitis: A systematic review and meta-analysis. Int Immunopharmacol. 2017 Nov; 52:310-317.

227. Hartl A, Sieper J, Syrbe U, Listing J, Hermann KG, Rudwaleit M, et al. Serum levels of leptin and high molecular weight adiponectin are inversely associated with radiographic spinal progression in patients with ankylosing spondylitis: results from the ENRADAS trial. Arthritis Res Ther. 2017 Jun 15; 19(1):140.

228. Miranda-Filloy JA, López-Mejias R, Genre F, Carnero-López B, Ochoa R, Diaz de Terán T, et al. Adiponectin and resistin serum levels in non-diabetic ankylosing spondylitis patients undergoing TNF-α antagonist therapy. Clin Exp Rheumatol. 2013 May-Jun; 31(3):365-371.

229. Gonzalez-Lopez L, Fajardo-Robledo NS, Miriam Saldana-Cruz A, Moreno-Sandoval IV, Bonilla-Lara D, Zavaleta-Muniz S, et al. Association of adipokines, interleukin-6, and tumor necrosis factor-alpha concentrations with clinical characteristics and presence of spinal syndesmophytes in patients with ankylosing spondylitis: A cross-sectional study. J Int Med Res. 2017 Jun; 45(3):1024-1035.

230. Pishgahi A, Abolhasan R, Danaii S, Amanifar B, Soltani-Zangbar MS, Zamani M, et al. Immunological and oxidative stress biomarkers in Ankylosing Spondylitis patients with or without metabolic syndrome. Cytokine. 2020 Apr; 128:155002.

231. Xie J, Yang M, Yu H, Xu K, Wan X, Wang J, et al. No Evidence to Support a Causal Relationship between Circulating Adiponectin Levels and Ankylosing Spondylitis: A Bidirectional Two-Sample Mendelian Randomization Study. Genes (Basel). 2022 Dec 2; 13(12).

232. Park MC, Chung SJ, Park YB, Lee SK. Pro-inflammatory effect of leptin on peripheral blood mononuclear cells of patients with ankylosing spondylitis. Joint Bone Spine. 2009 Mar; 76(2):170-175.

233. Kocabas H, Kocabas V, Buyukbas S, Melikoglu MA, Sezer I, Butun B. The serum levels of resistin in ankylosing spondylitis patients: a pilot study. Rheumatol Int. 2012 Mar; 32(3):699-702.

234. Güler M, Çapkin E, Karkucak M, Aydin T, Türkyilmaz AK, Karaca A, et al. Plasma leptin status and the relationship between different medical treatments used in ankylosing spondylitis. Nobel Medicus. 2013; 9(2):109-113.

235. Kononoff A, Vuolteenaho K, Hamalainen M, Kautiainen H, Elfving P, Savolainen E, et al. Metabolic Syndrome, Disease Activity, and Adipokines in Patients With Newly Diagnosed Inflammatory Joint Diseases. J Clin Rheumatol. 2021 Dec 1; 27(8):e349-e356.

236. Toussirot E, Streit G, Nguyen NU, Dumoulin G, Le Huede G, Saas P, et al. Adipose tissue, serum adipokines, and ghrelin in patients with ankylosing spondylitis. Metabolism. 2007 Oct; 56(10):1383-1389.

237. Elolemy GG, Ganeb SS, Ghanima ATA, Abdelgwad ER. Influence of adipocytokines and IL-6 on ankylosing spondylitis disease activity and functional status. The Egyptian Rheumatologist. 2013; 35(2):65-70.

238. Park MC, Lee SW, Choi ST, Park YB, Lee SK. Serum leptin levels correlate with interleukin-6 levels and disease activity in patients with ankylosing spondylitis. Scand J Rheumatol. 2007 Mar-Apr; 36(2):101-106.

239. Rademacher J, Siderius M, Gellert L, Wink FR, Verba M, Maas F, et al. Baseline serum biomarkers of inflammation, bone turnover and adipokines predict spinal radiographic progression in ankylosing spondylitis patients on TNF inhibitor therapy. Semin Arthritis Rheum. 2022 Apr; 53:151974.

240. Czokolyova M, Pusztai A, Vegh E, Horvath A, Szentpeteri A, Hamar A, et al. Changes of Metabolic Biomarker Levels upon One-Year Anti-TNF-alpha Therapy in Rheumatoid Arthritis and Ankylosing Spondylitis: Associations with Vascular Pathophysiology. Biomolecules. 2021 Oct 18; 11(10).

241. Derdemezis CS, Filippatos TD, Voulgari PV, Tselepis AD, Drosos AA, Kiortsis DN. Leptin and adiponectin levels in patients with ankylosing spondylitis. The effect of infliximab treatment. Clin Exp Rheumatol. 2010 Nov-Dec; 28(6):880-883.

242. Miranda-Filloy JA, López-Mejias R, Genre F, Carnero-López B, Ochoa R, Diaz de Terán T, et al. Leptin and visfatin serum levels in non-diabetic ankylosing spondylitis patients undergoing TNF-α antagonist therapy. Clin Exp Rheumatol. 2013 Jul-Aug; 31(4):538-545.

243. Hulejova H, Levitova A, Kuklova M, Stochl J, Haluzik M, Pavelka K, et al. No effect of physiotherapy on the serum levels of adipocytokines in patients with ankylosing spondylitis. Clin Rheumatol. 2012 Jan; 31(1):67-71.

244. Inman RD, Baraliakos X, Hermann KA, Braun J, Deodhar A, van der Heijde D, et al. Serum biomarkers and changes in clinical/MRI evidence of golimumab-treated patients with ankylosing spondylitis: results of the randomized, placebo-controlled GO-RAISE study. Arthritis Res Ther. 2016 Dec 28; 18(1):304.

245. Wagner C, Visvanathan S, Braun J, van der Heijde D, Deodhar A, Hsu B, et al. Serum markers associated with clinical improvement in patients with ankylosing spondylitis treated with golimumab. Ann Rheum Dis. 2012 May; 71(5):674-680.

246. Sari I, Demir T, Kozaci LD, Akar S, Kavak T, Birlik M, et al. Body composition, insulin, and leptin levels in patients with ankylosing spondylitis. Clin Rheumatol. 2007 Sep; 26(9):1427-1432.

247. Wang HH, Wang QF. Low vaspin levels are related to endothelial dysfunction in patients with ankylosing spondylitis. Braz J Med Biol Res. 2016 Jul 4; 49(7).

248. Genre F, Rueda-Gotor J, Remuzgo-Martinez S, Pulito-Cueto V, Corrales A, Mijares V, et al. Omentin: a biomarker of cardiovascular risk in individuals with axial spondyloarthritis. Sci Rep. 2020 Jun 15; 10(1):9636.

249. Toussirot E, Grandclement E, Gaugler B, Michel F, Wendling D, Saas P, et al. Serum adipokines and adipose tissue distribution in rheumatoid arthritis and ankylosing spondylitis. A comparative study. Front Immunol. 2013; 4:453.

250. Kim KJ, Kim JY, Park SJ, Yoon H, Yoon CH, Kim WU, et al. Serum leptin levels are associated with the presence of syndesmophytes in male patients with ankylosing spondylitis. Clin Rheumatol. 2012 Aug; 31(8):1231-1238.

251. Toussirot E, Mourot L, Dehecq B, Wendling D, Grandclement E, Dumoulin G, et al. TNFalpha blockade for inflammatory rheumatic diseases is associated with a significant gain in android fat mass and has varying effects on adipokines: a 2-year prospective study. Eur J Nutr. 2014 Apr; 53(3):951-961.

252. Rueda-Gotor J, Lopez-Mejias R, Remuzgo-Martinez S, Pulito-Cueto V, Corrales A, Lera-Gomez L, et al. Vaspin in atherosclerotic disease and cardiovascular risk in axial spondyloarthritis: a genetic and serological study. Arthritis Res Ther. 2021 Apr 13; 23(1):111.

253. Zhan H, Li H, Liu C, Cheng L, Yan S, Li Y. Association of Circulating Vascular Endothelial Growth Factor Levels With Autoimmune Diseases: A Systematic Review and Meta-Analysis. Frontiers in Immunology. 2021; 12.

254. Goldberger C, Dulak J, Duftner C, Weidinger F, Falkenbach A, Schirmer M. Vascular endothelial growth factor (VEGF) in ankylosing spondylitis--a pilot study. Wien Med Wochenschr. 2002; 152(9-10):223-225.

255. Drouart M, Saas P, Billot M, Cedoz JP, Tiberghien P, Wendling D, et al. High serum vascular endothelial growth factor correlates with disease activity of spondylarthropathies. Clin Exp Immunol. 2003 Apr; 132(1):158-162.

256. Wang M, Zhou X, Zhang H, Liu R, Xu N. Associations of the VEGF level, VEGF rs2010963 G/C gene polymorphism and ankylosing spondylitis risk in a Chinese Han population. Immunol Lett. 2016 Nov; 179:56-60.

257. Pedersen SJ, Sorensen IJ, Garnero P, Johansen JS, Madsen OR, Tvede N, et al. ASDAS, BASDAI and different treatment responses and their relation to biomarkers of inflammation, cartilage and bone turnover in patients with axial spondyloarthritis treated with TNFalpha inhibitors. Ann Rheum Dis. 2011 Aug; 70(8):1375-1381.

258. Lin TT, Lu J, Qi CY, Yuan L, Li XL, Xia LP, et al. Elevated serum level of IL-27 and VEGF in patients with ankylosing spondylitis and associate with disease activity. Clin Exp Med. 2015 May; 15(2):227-231.

259. Przepiera-Bedzak H, Fischer K, Brzosko M. Serum VEGF, EGF, basic FGF, and acidic FGF levels and their association with disease activity and extra‑articular symptoms in ankylosing spondylitis. Pol Arch Med Wewn. 2016 Mar 24; 126(4):290-292.

260. Sakellariou GT, Iliopoulos A, Konsta M, Kenanidis E, Potoupnis M, Tsiridis E, et al. Serum levels of Dkk-1, sclerostin and VEGF in patients with ankylosing spondylitis and their association with smoking, and clinical, inflammatory and radiographic parameters. Joint Bone Spine. 2017 May; 84(3):309-315.

261. Torres L, Klingberg E, Nurkkala M, Carlsten H, Forsblad-d'Elia H. Hepatocyte growth factor is a potential biomarker for osteoproliferation and osteoporosis in ankylosing spondylitis. Osteoporos Int. 2019 Feb; 30(2):441-449.

262. Appel H, Janssen L, Listing J, Heydrich R, Rudwaleit M, Sieper J. Serum levels of biomarkers of bone and cartilage destruction and new bone formation in different cohorts of patients with axial spondyloarthritis with and without tumor necrosis factor-alpha blocker treatment. Arthritis Res Ther. 2008; 10(5):R125.

263. Visvanathan S, Wagner C, Marini JC, Baker D, Gathany T, Han J, et al. Inflammatory biomarkers, disease activity and spinal disease measures in patients with ankylosing spondylitis after treatment with infliximab. Ann Rheum Dis. 2008 Apr; 67(4):511-517.

264. Pedersen SJ, Hetland ML, Sorensen IJ, Ostergaard M, Nielsen HJ, Johansen JS. Circulating levels of interleukin-6, vascular endothelial growth factor, YKL-40, matrix metalloproteinase-3, and total aggrecan in spondyloarthritis patients during 3 years of treatment with TNFalpha inhibitors. Clin Rheumatol. 2010 Nov; 29(11):1301-1309.

265. Tosovsky M, Bradna P, Andrys C, Andrysova K, Cermakova E, Soukup T. The VEGF and BMP-2 levels in patients with ankylosing spondylitis and the relationship to treatment with tumour necrosis factor alpha inhibitors. Acta Medica (Hradec Kralove). 2014; 57(2):56-61.

266. Seo JS, Lee SS, Kim SI, Ryu WH, Sa KH, Kim SU, et al. Influence of VEGF gene polymorphisms on the severity of ankylosing spondylitis. Rheumatology (Oxford). 2005 Oct; 44(10):1299-1302.

267. Poddubnyy D, Conrad K, Haibel H, Syrbe U, Appel H, Braun J, et al. Elevated serum level of the vascular endothelial growth factor predicts radiographic spinal progression in patients with axial spondyloarthritis. Ann Rheum Dis. 2014 Dec; 73(12):2137-2143.

268. Braun J, Baraliakos X, Hermann KG, Xu S, Hsu B. Serum Vascular Endothelial Growth Factor Levels Lack Predictive Value in Patients with Active Ankylosing Spondylitis Treated with Golimumab. J Rheumatol. 2016 May; 43(5):901-906.

269. Ma Y, Fan D, Xu S, Deng J, Gao X, Guan S, et al. Calprotectin in spondyloarthritis: A systematic review and meta-analysis. Int Immunopharmacol. 2020 Nov; 88:106948.

270. Van Praet L, Van den Bosch FE, Jacques P, Carron P, Jans L, Colman R, et al. Microscopic gut inflammation in axial spondyloarthritis: a multiparametric predictive model. Ann Rheum Dis. 2013 Mar; 72(3):414-417.

271. Ercalik C, Baskaya MC, Ozdem S, Butun B. Investigation of asymptomatic intestinal inflammation in ankylosing spondylitis by fecal calprotectin. Arab J Gastroenterol. 2021 Dec; 22(4):272-277.

272. Genre F, Rueda-Gotor J, Remuzgo-Martinez S, Corrales A, Mijares V, Exposito R, et al. Association of circulating calprotectin with lipid profile in axial spondyloarthritis. Sci Rep. 2018 Sep 13; 8(1):13728.

273. Huang J, Yin Z, Song G, Cui S, Jiang J, Zhang L. Discriminating Value of Calprotectin in Disease Activity and Progression of Nonradiographic Axial Spondyloarthritis and Ankylosing Spondylitis. Dis Markers. 2017; 2017:7574147.

274. Klingberg E, Carlsten H, Hilme E, Hedberg M, Forsblad-d'Elia H. Calprotectin in ankylosing spondylitis--frequently elevated in feces, but normal in serum. Scand J Gastroenterol. 2012 Apr; 47(4):435-444.

275. Oktayoglu P, Bozkurt M, Mete N, Caglayan M, Em S, Nas K. Elevated serum levels of calprotectin (myeloid-related protein 8/14) in patients with ankylosing spondylitis and its association with disease activity and quality of life. J Investig Med. 2014 Aug; 62(6):880-884.

276. Olofsson T, Lindqvist E, Mogard E, Andreasson K, Marsal J, Geijer M, et al. Elevated faecal calprotectin is linked to worse disease status in axial spondyloarthritis: results from the SPARTAKUS cohort. Rheumatology (Oxford). 2019 Jul 1; 58(7):1176-1187.

277. Li J, Xie X, Liu W, Gu F, Zhang K, Su Z, et al. MicroRNAs as Biomarkers for the Diagnosis of Ankylosing Spondylitis: A Systematic Review and Meta-Analysis. Front Med (Lausanne). 2021; 8:701789.

278. Fotoh DS, Noreldin RI, Rizk MS, Elsabaawy MM, Esaily HA. miRNA-451a and miRNA-125a Expression Levels in Ankylosing Spondylitis: Impact on Disease Diagnosis, Prognosis, and Outcomes. J Immunol Res. 2020; 2020:2180913.

279. Liu Z, Huang F, Luo G, Wang Y, Du R, Sun W, et al. miR-214 stimulated by IL-17A regulates bone loss in patients with ankylosing spondylitis. Rheumatology (Oxford). 2020 May 1; 59(5):1159-1169.

280. Ni WJ, Leng XM. Down-regulated miR-495 can target programmed cell death 10 in ankylosing spondylitis. Mol Med. 2020 May 25; 26(1):50.

281. Perez-Sanchez C, Font-Ugalde P, Ruiz-Limon P, Lopez-Pedrera C, Castro-Villegas MC, Abalos-Aguilera MC, et al. Circulating microRNAs as potential biomarkers of disease activity and structural damage in ankylosing spondylitis patients. Hum Mol Genet. 2018 Mar 1; 27(5):875-890.

282. Tan H, Ren R, Zhang J, Huang Z, Niu Q, Yang B. Analysis of inflammation-related microRNA expression in patients with ankylosing spondylitis. Immunol Res. 2022 Feb; 70(1):23-32.

283. Li X, Lv Q, Tu L, Zhao M, Zhang P, Li Q, et al. Aberrant expression of microRNAs in peripheral blood mononuclear cells as candidate biomarkers in patients with axial spondyloarthritis. Int J Rheum Dis. 2019 Jul; 22(7):1188-1195.

284. Huang CH, Wei JC, Chang WC, Chiou SY, Chou CH, Lin YJ, et al. Higher expression of whole blood microRNA-21 in patients with ankylosing spondylitis associated with programmed cell death 4 mRNA expression and collagen cross-linked C-telopeptide concentration. J Rheumatol. 2014 Jun; 41(6):1104-1111.

285. Lan X, Ma H, Zhang Z, Ye D, Min J, Cai F, et al. Downregulation of lncRNA TUG1 is involved in ankylosing spondylitis and is related to disease activity and course of treatment. Biosci Trends. 2018 Sep 19; 12(4):389-394.

286. Zhong H, Zhong M. LINC00311 is overexpressed in ankylosing spondylitis and predict treatment outcomes and recurrence. BMC Musculoskelet Disord. 2019 Jun 7; 20(1):278.

287. Wang JX, Zhao X, Xu SQ. Screening Key lncRNAs of Ankylosing Spondylitis Using Bioinformatics Analysis. J Inflamm Res. 2022; 15:6087-6096.

288. Wang T, Meng S, Chen P, Wei L, Liu C, Tang D, et al. Comprehensive analysis of differentially expressed mRNA and circRNA in Ankylosing spondylitis patients' platelets. Exp Cell Res. 2021 Dec 1; 409(1):112895.

289. Tang YP, Zhang QB, Dai F, Liao X, Dong ZR, Yi T, et al. Circular RNAs in peripheral blood mononuclear cells from ankylosing spondylitis. Chin Med J (Engl). 2021 Oct 19; 134(21):2573-2582.

290. Luo Q, Fu B, Zhang L, Guo Y, Huang Z, Li J. Expression and clinical significance of circular RNA hsa_circ_0079787 in the peripheral blood of patients with axial spondyloarthritis. Mol Med Rep. 2020 Nov; 22(5):4197-4206.

291. Qian BP, Ji ML, Qiu Y, Wang B, Yu Y, Shi W, et al. Identification of Serum miR-146a and miR-155 as Novel Noninvasive Complementary Biomarkers for Ankylosing Spondylitis. Spine (Phila Pa 1976). 2016 May; 41(9):735-742.

292. Wang M, Wang L, Zhang X, Yang X, Li X, Xia Q, et al. Overexpression of miR-31 in Peripheral Blood Mononuclear Cells (PBMC) from Patients with Ankylosing Spondylitis. Med Sci Monit. 2017 Nov 18; 23:5488-5494.

293. Wang Y, Luo J, Wang X, Yang B, Cui L. MicroRNA-199a-5p Induced Autophagy and Inhibits the Pathogenesis of Ankylosing Spondylitis by Modulating the mTOR Signaling via Directly Targeting Ras Homolog Enriched in Brain (Rheb). Cell Physiol Biochem. 2017; 42(6):2481-2491.

294. Wei C, Zhang H, Wei C, Mao Y. Correlation of the expression of miR-146a in peripheral blood mononuclear cells of patients with ankylosing spondylitis and inflammatory factors. Exp Ther Med. 2017 Nov; 14(5):5027-5031.

295. Yildirim T, Yesilada E, Eren F, Apaydin H, Gulbay G. Assessment of plasma microRNA potentials as a non-invasive biomarker in patients with axial spondyloarthropathy. Eur Rev Med Pharmacol Sci. 2021 Jan; 25(2):620-625.

296. Zhang CL, Li YC, Wu JW, Zhu BL. Expression and function of peripheral blood miRNA16a in patients with ankylosing spondylitis. Eur Rev Med Pharmacol Sci. 2018 Aug; 22(16):5106-5113.

297. Prajzlerova K, Grobelna K, Husakova M, Forejtova S, Jungel A, Gay S, et al. Association between circulating miRNAs and spinal involvement in patients with axial spondyloarthritis. PLoS One. 2017; 12(9):e0185323.

298. Reyes-Loyola P, Rodriguez-Henriquez P, Ballinas-Verdugo MA, Amezcua-Castillo LM, Juarez-Vicuna Y, Jimenez-Rojas V, et al. Plasma let-7i, miR-16, and miR-221 levels as candidate biomarkers for the assessment of ankylosing spondylitis in Mexican patients naive to anti-TNF therapy. Clin Rheumatol. 2019 May; 38(5):1367-1373.

299. Han D, Ouyang G, Pan P, Yuan Y. Upregulated lncRNA-NEF predicts recurrence and poor treatment outcomes of ankylosing spondylitis. Immun Inflamm Dis. 2022 Aug; 10(8):e627.

300. Guo TM, Yan Y, Cao WN, Liu Q, Zhu HY, Yang L, et al. Predictive value of microRNA-132 and its target gene NAG-1 in evaluating therapeutic efficacy of non-steroidal anti-inflammatory drugs treatment in patients with ankylosing spondylitis. Clin Rheumatol. 2018 May; 37(5):1281-1293.

301. Ciechomska M, Bonek K, Merdas M, Zarecki P, Swierkot J, Gluszko P, et al. Changes in MiRNA-5196 Expression as a Potential Biomarker of Anti-TNF-alpha Therapy in Rheumatoid Arthritis and Ankylosing Spondylitis Patients. Arch Immunol Ther Exp (Warsz). 2018 Oct; 66(5):389-397.

302. Lv Q, Li Q, Zhang P, Jiang Y, Wang X, Wei Q, et al. Disorders of MicroRNAs in Peripheral Blood Mononuclear Cells: As Novel Biomarkers of Ankylosing Spondylitis and Provocative Therapeutic Targets. Biomed Res Int. 2015; 2015:504208.

303. Zou YC, Gao YP, Yin HD, Liu G. Serum miR-21 expression correlates with radiographic progression but also low bone mineral density in patients with ankylosing spondylitis: a cross-sectional study. Innate Immun. 2019 Jul; 25(5):314-321.

304. Zou YC, Wu J, Zhao C, Luo ZR. Analysis of circular RNA expression profile of pathological bone formation in ankylosing spondylitis. Int J Rheum Dis. 2023 Jul; 26(7):1403-1406.

305. Liu W, Wu YH, Zhang L, Liu XY, Xue B, Wang Y, et al. Elevated serum levels of IL-6 and IL-17 may associate with the development of ankylosing spondylitis. Int J Clin Exp Med. 2015; 8(10):17362-17376.

306. Bal A, Unlu E, Bahar G, Aydog E, Eksioglu E, Yorgancioglu R. Comparison of serum IL-1 beta, sIL-2R, IL-6, and TNF-alpha levels with disease activity parameters in ankylosing spondylitis. Clin Rheumatol. 2007 Feb; 26(2):211-215.

307. Peng F, Chen F, Wen H, Bai J, Tian Y. Measurement of pre-treatment inflammatory cytokine levels is valuable for prediction of treatment efficacy to tumor necrosis factor inhibitor in axial spondyloarthritis patients. International Journal of Rheumatic Diseases. 2022; 25(8):844-850.

308. Romero-Sanchez C, Tsou HK, Jan MS, Wong RH, Chang IC, Londono J, et al. Serum monocyte chemotactic protein-1 concentrations distinguish patients with ankylosing spondylitis from patients with mechanical low back pain. J Spinal Disord Tech. 2011 May; 24(3):202-207.

309. Gratacos J, Collado A, Pons F, Osaba M, Sanmarti R, Roque M, et al. Significant loss of bone mass in patients with early, active ankylosing spondylitis: a followup study. Arthritis Rheum. 1999 Nov; 42(11):2319-2324.

310. Sharma SK, Ahmad S, Sharma SK. Serum IL-6 level as a marker of disease activity in ankylosing spondylitis patients with pure axial involvement. Indian Journal of Rheumatology. 2014; 9(3):115-119.

311. Przepiera-Bedzak H, Fischer K, Brzosko M. Serum IL-6 and IL-23 Levels and Their Correlation with Angiogenic Cytokines and Disease Activity in Ankylosing Spondylitis, Psoriatic Arthritis, and SAPHO Syndrome. Mediators Inflamm. 2015; 2015:785705.

312. Li DH, He CR, Liu FP, Li J, Gao JW, Li Y, et al. Annexin A2, up-regulated by IL-6, promotes the ossification of ligament fibroblasts from ankylosing spondylitis patients. Biomed Pharmacother. 2016 Dec; 84:674-679.

313. Rabelo CF, Baptista TSA, Petersen LE, Bauer ME, Keiserman MW, Staub HL. Serum IL-6 correlates with axial mobility index (Bath Ankylosing Spondylitis Metrology Index) in Brazilian patients with ankylosing spondylitis. Open Access Rheumatol. 2018; 10:21-25.

314. Mattey DL, Packham JC, Nixon NB, Coates L, Creamer P, Hailwood S, et al. Association of cytokine and matrix metalloproteinase profiles with disease activity and function in ankylosing spondylitis. Arthritis Res Ther. 2012 May 28; 14(3):R127.

315. Taylan A, Sari I, Kozaci DL, Yildiz Y, Bilge S, Coker I, et al. Evaluation of various endothelial biomarkers in ankylosing spondylitis. Clin Rheumatol. 2012 Jan; 31(1):23-28.

316. Taylan A, Sari I, Kozaci DL, Yuksel A, Bilge S, Yildiz Y, et al. Evaluation of the T helper 17 axis in ankylosing spondylitis. Rheumatol Int. 2012 Aug; 32(8):2511-2515.

317. Sveaas SH, Berg IJ, Provan SA, Semb AG, Olsen IC, Ueland T, et al. Circulating levels of inflammatory cytokines and cytokine receptors in patients with ankylosing spondylitis: a cross-sectional comparative study. Scand J Rheumatol. 2015; 44(2):118-124.

318. Liu R, Yue Z, Peng X, Wang X, Feng Z, Wan L. Association Between Intercellular Adhesion Molecule-1, -2, -3 Plasma Levels and Disease Activity of Ankylosing Spondylitis in the Chinese Han Population. Spine (Phila Pa 1976). 2016 May; 41(10):E618-624.

319. He D, Zhu Q, Zhou Q, Qi Q, Sun H, Zachariah LM, et al. Correlation of serum MMP3 and other biomarkers with clinical outcomes in patients with ankylosing spondylitis: a pilot study. Clin Rheumatol. 2017 Aug; 36(8):1819-1826.

320. Korkosz M, Czepiel M, Gula Z, Stec M, Weglarczyk K, Rutkowska-Zapala M, et al. Sera of patients with axial spondyloarthritis (axSpA) enhance osteoclastogenic potential of monocytes isolated from healthy individuals. BMC Musculoskelet Disord. 2018 Dec 6; 19(1):434.

321. Falkenbach A, Herold M. In ankylosing spondylitis serum interleukin-6 correlates with the degree of mobility restriction, but not with short-term changes in the variables for mobility. Rheumatol Int. 1998; 18(3):103-106.

322. Falkenbach A, Herold M, Wigand R. Interleukin-6 serum concentration in ankylosing spondylitis: a reliable predictor of disease progression in the subsequent year? Rheumatol Int. 2000; 19(4):149-151.

323. Brandt J, Haibel H, Cornely D, Golder W, Gonzalez J, Reddig J, et al. Successful treatment of active ankylosing spondylitis with the anti-tumor necrosis factor alpha monoclonal antibody infliximab. Arthritis Rheum. 2000 Jun; 43(6):1346-1352.

324. Tarner IH, Muller-Ladner U, Uhlemann C, Lange U. The effect of mild whole-body hyperthermia on systemic levels of TNF-alpha, IL-1beta, and IL-6 in patients with ankylosing spondylitis. Clin Rheumatol. 2009 Apr; 28(4):397-402.

325. Pedersen SJ, Sorensen IJ, Lambert RG, Hermann KG, Garnero P, Johansen JS, et al. Radiographic progression is associated with resolution of systemic inflammation in patients with axial spondylarthritis treated with tumor necrosis factor alpha inhibitors: a study of radiographic progression, inflammation on magnetic resonance imaging, and circulating biomarkers of inflammation, angiogenesis, and cartilage and bone turnover. Arthritis Rheum. 2011 Dec; 63(12):3789-3800.

326. Capkin E, Karkucak M, Akyuz A, Alver A, Turkyilmaz AK, Zengin E. The relationship between plasma homocysteine level and different treatment modalities in patients with ankylosing spondylitis. Rheumatol Int. 2012 Aug; 32(8):2349-2353.

327. Limon-Camacho L, Vargas-Rojas MI, Vazquez-Mellado J, Casasola-Vargas J, Moctezuma JF, Burgos-Vargas R, et al. In vivo peripheral blood proinflammatory T cells in patients with ankylosing spondylitis. J Rheumatol. 2012 Apr; 39(4):830-835.

328. Xueyi L, Lina C, Zhenbiao W, Qing H, Qiang L, Zhu P. Levels of circulating Th17 cells and regulatory T cells in ankylosing spondylitis patients with an inadequate response to anti-TNF-alpha therapy. J Clin Immunol. 2013 Jan; 33(1):151-161.

329. Schulz M, Dotzlaw H, Neeck G. Ankylosing spondylitis and rheumatoid arthritis: serum levels of TNF-alpha and Its soluble receptors during the course of therapy with etanercept and infliximab. Biomed Res Int. 2014; 2014:675108.

330. Levitova A, Hulejova H, Spiritovic M, Pavelka K, Senolt L, Husakova M. Clinical improvement and reduction in serum calprotectin levels after an intensive exercise programme for patients with ankylosing spondylitis and non-radiographic axial spondyloarthritis. Arthritis Res Ther. 2016 Nov 25; 18(1):275.

331. Zhang Y, Ning C, Zhou H, Yan Y, Liu F, Huang Y. Interleukin-1beta, interleukin-6, and interleukin-17A as indicators reflecting clinical response to celecoxib in ankylosing spondylitis patients. Ir J Med Sci. 2021 May; 190(2):631-638.

332. Eggert M, Seeck U, Semmler M, Maass U, Dietmann S, Schulz M, et al. An evaluation of anti-TNF-alpha-therapy in patients with ankylosing spondylitis: imbalanced activation of NF kappa B subunits in lymphocytes and modulation of serum cortisol concentration. Rheumatol Int. 2007 Jul; 27(9):841-846.

333. Du J, Sun J, Wen Z, Wu Z, Li Q, Xia Y, et al. Serum IL-6 and TNF-alpha Levels Are Correlated with Disease Severity in Patients with Ankylosing Spondylitis. Lab Med. 2022 Mar 7; 53(2):149-155.

334. Korczowska I, Przepiera-Bedzak H, Brzosko M, Lacki JK, Trefler J, Hrycaj P. Bone tissue metabolism in men with ankylosing spondylitis. Adv Med Sci. 2011; 56(2):264-269.

335. Yang B, Xu Y, Liu X, Huang Z, Wang L. IL-23R and IL-17A polymorphisms correlate with susceptibility of ankylosing spondylitis in a Southwest Chinese population. Oncotarget. 2017 Sep 19; 8(41):70310-70316.

336. Aghaei H, Farhadi E, Akhtari M, Shahba S, Mostafaei S, Jamshidi A, et al. Copy number variation of IL17RA gene and its association with the ankylosing spondylitis risk in Iranian patients: a case-control study. BMC Med Genet. 2020 Jul 10; 21(1):147.

337. Chen WS, Chang YS, Lin KC, Lai CC, Wang SH, Hsiao KH, et al. Association of serum interleukin-17 and interleukin-23 levels with disease activity in Chinese patients with ankylosing spondylitis. J Chin Med Assoc. 2012 Jul; 75(7):303-308.

338. Mei Y, Pan F, Gao J, Ge R, Duan Z, Zeng Z, et al. Increased serum IL-17 and IL-23 in the patient with ankylosing spondylitis. Clin Rheumatol. 2011 Feb; 30(2):269-273.

339. Wendling D, Cedoz JP, Racadot E, Dumoulin G. Serum IL-17, BMP-7, and bone turnover markers in patients with ankylosing spondylitis. Joint Bone Spine. 2007 May; 74(3):304-305.

340. Wielinska J, Swierkot J, Kolossa K, Bugaj B, Chaszczewska-Markowska M, Jeka S, et al. Polymorphisms within Genes Coding for IL-17A and F and Their Receptor as Clinical Hallmarks in Ankylosing Spondylitis. Mediators Inflamm. 2021; 2021:3125922.

341. Tan H, Huang S, Wang T. Clinical Significance of Peripheral Blood Th1 and Th17 Cell Content and Serum IL-35 and IL-17 Expression in Patients with Ankylosing Spondylitis. Evid Based Complement Alternat Med. 2022; 2022:6540557.

342. Jansen DT, Hameetman M, van Bergen J, Huizinga TW, van der Heijde D, Toes RE, et al. IL-17-producing CD4+ T cells are increased in early, active axial spondyloarthritis including patients without imaging abnormalities. Rheumatology (Oxford). 2015 Apr; 54(4):728-735.

343. Perpetuo IP, Raposeiro R, Caetano-Lopes J, Vieira-Sousa E, Campanilho-Marques R, Ponte C, et al. Effect of Tumor Necrosis Factor Inhibitor Therapy on Osteoclasts Precursors in Ankylosing Spondylitis. PLoS One. 2015; 10(12):e0144655.

344. Milanez FM, Saad CG, Viana VT, Moraes JC, Perico GV, Sampaio-Barros PD, et al. IL-23/Th17 axis is not influenced by TNF-blocking agents in ankylosing spondylitis patients. Arthritis Res Ther. 2016 Feb 24; 18:52.

345. Wen JT, Zhang DH, Fang PF, Li MH, Wang RJ, Li SH. Role of Th1/Th2 cytokines in the diagnosis and prognostic evaluation of ankylosing spondylitis. Genet Mol Res. 2017 Mar 16; 16(1).

346. Dong Y, Li P, Xu T, Bi L. Effective serum level of etanercept biosimilar and effect of antidrug antibodies on drug levels and clinical efficacy in Chinese patients with ankylosing spondylitis. Clin Rheumatol. 2019 Jun; 38(6):1587-1594.

347. An H, Li X, Li F, Gao C, Li X, Luo J. The absolute counts of peripheral T lymphocyte subsets in patient with ankylosing spondylitis and the effect of low-dose interleukin-2. Medicine (Baltimore). 2019 Apr; 98(15):e15094.

348. Appel H, Wu P, Scheer R, Kedor C, Sawitzki B, Thiel A, et al. Synovial and peripheral blood CD4+FoxP3+ T cells in spondyloarthritis. J Rheumatol. 2011 Nov; 38(11):2445-2451.

349. Bautista-Caro MB, Arroyo-Villa I, Castillo-Gallego C, de Miguel E, Peiteado D, Plasencia-Rodríguez C, et al. Decreased frequencies of circulating follicular helper T cell counterparts and plasmablasts in ankylosing spondylitis patients Naïve for TNF blockers. PLoS One. 2014; 9(9):e107086.

350. Bidad K, Salehi E, Jamshidi A, Saboor-Yaraghi AA, Oraei M, Meysamie A, et al. Effect of all-transretinoic acid on Th17 and T regulatory cell subsets in patients with ankylosing spondylitis. J Rheumatol. 2013 Apr; 40(4):476-483.

351. Brand JM, Neustock P, Kruse A, Alvarez-Ossorio L, Schnabel A, Kirchner H. Stimulation of whole blood cultures in patients with ankylosing spondylitis by a mitogen derived from Mycoplasma arthritidis (MAS) and other mitogens. Rheumatol Int. 1997; 16(5):207-211.

352. Cai CS, Xiao P. [Expression of regulatory T cells in the peripheral blood of patients with ankylosing spondylitis.]. Journal of Chinese Practical Diagnosis and Therapy. 2013; 27(12):1192-1194.

353. Cai PW, Lin Y, Dou M, Chen JH, Lin Y. [Expression of CD40-CD40L on peripheral blood lymphocytes of patients with Ankylosing spondylitis.]. Immunological Journal. 2005; 21(06):507-508+513.

354. Cao D, van Vollenhoven R, Klareskog L, Trollmo C, Malmstrom V. CD25brightCD4+ regulatory T cells are enriched in inflamed joints of patients with chronic rheumatic disease. Arthritis Res Ther. 2004; 6(4):R335-346.

355. Chen SZ, Bai JP, Xie YH, You YQ, Su ML, Xu XX, et al. [Expression of transcription factor Th17，Treg and Th1 in peripheral blood from patients with ankylosing spondylitis and its correlation with disease activity.]. Chinese Journal of Immunology. 2013; 29(08):834-838+847.

356. Chen MH, Chen WS, Lee HT, Tsai CY, Chou CT. Inverse correlation of programmed death 1 (PD-1) expression in T cells to the spinal radiologic changes in Taiwanese patients with ankylosing spondylitis. Clin Rheumatol. 2011 Sep; 30(9):1181-1187.

357. Cheng F. [CD4+CD25+regulatory T cells in peripheral blood of patients with ankylosing spondylitis.] [doctoral thesis]: The Second Military Medical University; 2007.

358. Dejaco C, Duftner C, Klauser A, Schirmer M. Altered T-cell subtypes in spondyloarthritis, rheumatoid arthritis and polymyalgia rheumatica. Rheumatol Int. 2010 Jan; 30(3):297-303.

359. Deng L, Chen YP, Sun YL. [Expression of miR⁃138 in peripheral blood mononuclear cells of patients with ankylosing spondylitis and its relationship with Th1/Th2 imbalance.]. Journal of Tropical Medicine. 2019; 19(08):1008-1011+1051.

360. Deng JH, Li ZQ, Zhang R, Li YM. [The Correlation Ananlysis of HLA-B27 Expression and Lymphocytes Subsets, Cytokines in the Patients with Ankylosing Spondylitis.]. International Journal of Laboratory Medicine. 2018; 39(16):1976-1979.

361. Dong Q, Yang DR, Liu H. [The Test of Immune Function and Hemorheologic Changes in HLA-B27 Positive Patients with Ankylosing Spondylitis.]. Chinese Journal of Hemorheology. 2006; 16(02):273-274+324.

362. Duan Z, Gui Y, Li C, Lin J, Gober HJ, Qin J, et al. The immune dysfunction in ankylosing spondylitis patients. Biosci Trends. 2017 Mar 22; 11(1):69-76.

363. Dulic S, Vasarhelyi Z, Bajnok A, Szalay B, Toldi G, Kovacs L, et al. The Impact of Anti-TNF Therapy on CD4+ and CD8+ Cell Subsets in Ankylosing Spondylitis. Pathobiology. 2018; 85(3):201-210.

364. Fattahi MJ, Ahmadi H, Jafarnezhad-Ansariha F, Mortazavi-Jahromi SS, Rehm BHA, Cuzzocrea S, et al. Oral administration effects of beta-d-mannuronic acid (M2000) on Th17 and regulatory T cells in patients with ankylosing spondylitis. Biomed Pharmacother. 2018 Apr; 100:495-500.

365. Förger F, Villiger PM, Ostensen M. Pregnancy in patients with ankylosing spondylitis: do regulatory T cells play a role? Arthritis Rheum. 2009 Feb 15; 61(2):279-283.

366. Gao Y, Song Y, Fan YX, Chen M, Xiao N, Pan LZ, et al. [The alteration of TH17cells and CD4+CD25+FoxP3+ regulatory T cell in patients with ankylosing spondylitis.]. Chinese Journal of Microbiology and Immunology. 2012; 32(04):318-322.

367. Guo L, Hou Q, Kou R. [Application of Combined Detection of T Lymphocyte Subsets and Ferritin in Patients with Ankylosing Spondylitis.]. International Journal of Laboratory Medicine. 2012; 33(12):1436-1437.

368. Hajialilo M, Dolati S, Abdolmohammadi-Vahid S, Ahmadi M, Kamrani A, Eghbal-Fard S, et al. Nanocurcumin: A novel strategy in treating ankylosing spondylitis by modulating Th17 cells frequency and function. J Cell Biochem. 2019 Feb 25; 120(07):12027-12038.

369. Han YX, Zhang SH, Wu JB. [Expressions of B7 and CD28 in Peripheral Blood Lymphocytes of Patients with Ankylosing Spondylitis and their Significance.]. Journal of Wenzhou Medical University. 2006; 36(04):356-358.

370. He YH, Wu XM, Wu LJ. [Analysis of Lymphocyte Subsets in Patients with Ankylosing Spondylitis.]. International Journal of Laboratory Medicine. 2012; 33(02):141-142+145.

371. Hu W, Wang ML, Qiu W, Chen SM, Liu DS. [Preliminary study on immunological indicators of patients with ankylosing spondylitis in suqian.]. Contemporary Medicine. 2019; 25(10):1-3.

372. Hu B, Cheng J, Liu SQ. [Analysis of T cell subsets in peripheral blood in patients with ankylosing spondylitis.]. Modern Medical Journal. 2013; 41(08):543-545.

373. Huang H, Wang YD, Sun YY, Sui WG. [Expression regulatory T cells in peripheral blood of ankylosing spondyligis patients.]. China Tropical Medicine. 2009; 9(10):1992-1993.

374. Huang F, Cai XH, Shi GY, Chen XM, Cheng QL, Dong K, et al. [A study of cellular immune function in patients with ankylosing spondylitis.]. Academic Journal of Chinese PLA Medical School. 1991; 12(02):102-105.

375. Ji W, Li H, Gao F, Chen Y, Zhong L, Wang D. Effects of Tripterygium glycosides on interleukin-17 and CD4(+)CD25(+)CD127(low) regulatory T-cell expression in the peripheral blood of patients with ankylosing spondylitis. Biomed Rep. 2014 Jul; 2(4):517-520.

376. Kenna TJ, Davidson SI, Duan R, Bradbury LA, McFarlane J, Smith M, et al. Enrichment of circulating interleukin-17-secreting interleukin-23 receptor-positive γ/δ T cells in patients with active ankylosing spondylitis. Arthritis Rheum. 2012 May; 64(5):1420-1429.

377. Kim TJ, Lee SJ, Cho YN, Park SC, Jin HM, Kim MJ, et al. Immune cells and bone formation in ankylosing spondylitis. Clin Exp Rheumatol. 2012 Jul-Aug; 30(4):469-475.

378. Klasen C, Meyer A, Wittekind PS, Waque I, Nabhani S, Kofler DM. Prostaglandin receptor EP4 expression by Th17 cells is associated with high disease activity in ankylosing spondylitis. Arthritis Res Ther. 2019 Jun 28; 21(1):159.

379. Li WQ. [Expression of miＲ-155 in peripheral blood of patients with ankylosing spondylitis and its relationship with Th17 /Treg balance.]. Journal of Shanxi Medical University. 2019; 50(02):235-240.

380. Xueyi L, Lina C, Zhenbiao W, Qing H, Qiang L, Zhu P. Levels of circulating Th17 cells and regulatory T cells in ankylosing spondylitis patients with an inadequate response to anti-TNF-α therapy. J Clin Immunol. 2013 Jan; 33(1):151-161.

381. Li JX, Zhang LY, Huo YH, Li XF. [Effect of Methylprednisolone on the Th1/Th2 Balance and Cytokines in Patients with Refractong Ankylosing Spondylitis.]. Chinese Journal of Allergy and Clinical Immunology. 2009; 3(01):28-33.

382. Li HX, Sun GR, Cao YX, Wang JB. [Expression and significance of CD8+CD28-T cells in the peripheral blood of patients with AS.]. Chinese Journal of Rheumatology. 2008; 12(05):333-335+361.

383. Liao HT, Lin YF, Tsai CY, Chou CT. Regulatory T cells in ankylosing spondylitis and the response after adalimumab treatment. Joint Bone Spine. 2015 Dec; 82(6):423-427.

384. Limón-Camacho L, Vargas-Rojas MI, Vázquez-Mellado J, Casasola-Vargas J, Moctezuma JF, Burgos-Vargas R, et al. In vivo peripheral blood proinflammatory T cells in patients with ankylosing spondylitis. J Rheumatol. 2012 Apr; 39(4):830-835.

385. Lin Q, Lin ZM, Gu JR, Huang F, Li TW, Wei QJ, et al. [Changes of T lymphocyte subsets and expression of costimulatory molecule CD154 on T-cells in peripheral blood from patients with ankylosing spondylitis.]. Chinese Journal of Rheumatology. 2008; 12(05):309-313.

386. Lin Q, Gu JR, Li TW, Zhang FC, Lin ZM, Liao ZT, et al. Value of the peripheral blood B-cells subsets in patients with ankylosing spondylitis. Chin Med J (Engl). 2009 Aug 5; 122(15):1784-1789.

387. Liu EC, Feng YX. [Relationship of the Balance between Leptin and Th17 and Th1 with Ankylosing Spondycitis Patients.]. Medical Recapitulate. 2017; 23(01):187-189.

388. Liu L, Liu J, Wan L. [The changes of platelet parameters，BTLA and Treg in peripheral blood in patients with ankylosing spondylitis.]. Chinese Journal of Clinical Healthcare. 2016; 19(01):8-11.

389. Liu J, Wang SH, Wan L, Zhang JS, Yang J, Zong RK, et al. [Changes of regulatory T cells in peripheral blood in ankylosing spondylitis patients and the influence of chinese medicine spleen-strengthening unit therapy.]. Chinese Journal of Clinical Healthcare. 2012; 15(01):1-4+113.

390. Liu XC, Wang JX, Wei P. [The Function of Helper T Lymphocytes in Ankylosing Spondylitis.]. Tianjin Medical Journal. 2010; 38(12):1047-1049.

391. Long S, Ma L, Wang D, Shang X. High frequency of circulating follicular helper T cells is correlated with B cell subtypes in patients with ankylosing spondylitis. Exp Ther Med. 2018 May; 15(5):4578-4586.

392. Ma XH, Zhang X, Zhang N, Zhao Y, Song LT. [Detection of lymphocyte subsets in peripheral blood in patients with ankylosing spondylitis and its clinical meaning.]. Chinese Journal of Laboratory Diagnosis. 2011; 15(10):1765-1766.

393. Ma L, Zhang Y, Wang ZQ, Gu J. [A study of subsets and activation of lymphocytes in patients with ankylosing spondylitis.]. Journal of Clinical Research. 2011; 28(10):1963-1964.

394. Ma L, Yang J, Li H. [Study of the activated state of TH1/TH2 cytokines on ankylosing spondylitis.]. Chinese Journal of Immunology. 2004; 20(08):572-574.

395. Meng JH, Wei P, Chen HY, Wang JX, Xie JL, Zhang Y. [Change of cytotoxic T-lymphocytes in patients with ankylosing spondylitis.]. Journal of Hebei Medical University. 2015; 36(05):543-546.

396. Mo JF, Shan DP, Bao Y, Ye Q, Yan WH. [Proportion of several subsets of immune cells and expression of CXCR6 on CD3+ , CD4+ and γδT cells in peripheral blood of patients with ankylosing spondylitis.]. Current Immunology. 2019; 39(03):217-221.

397. Shan Y, Qi C, Zhao J, Liu Y, Gao H, Zhao D, et al. Higher frequency of peripheral blood follicular regulatory T cells in patients with new onset ankylosing spondylitis. Clin Exp Pharmacol Physiol. 2015 Feb; 42(2):154-161.

398. Shen H, Goodall JC, Hill Gaston JS. Frequency and phenotype of peripheral blood Th17 cells in ankylosing spondylitis and rheumatoid arthritis. Arthritis Rheum. 2009 Jun; 60(6):1647-1656.

399. Suen JL, Li HT, Jong YJ, Chiang BL, Yen JH. Altered homeostasis of CD4(+) FoxP3(+) regulatory T-cell subpopulations in systemic lupus erythematosus. Immunology. 2009 Jun; 127(2):196-205.

400. Szalay B, Meszaros G, Cseh A, Acs L, Deak M, Kovacs L, et al. Adaptive immunity in ankylosing spondylitis: phenotype and functional alterations of T-cells before and during infliximab therapy. Clin Dev Immunol. 2012; 2012:808724.

401. Szántó S, Aleksza M, Mihály E, Lakos G, Szabó Z, Végvári A, et al. Intracytoplasmic cytokine expression and T cell subset distribution in the peripheral blood of patients with ankylosing spondylitis. J Rheumatol. 2008 Dec; 35(12):2372-2375.

402. Thoen J, Førre O, Waalen K, Pahle J. Phenotypes and spontaneous cell cytotoxicity of mononuclear cells from patients with seronegative spondyloarthropathies: ankylosing spondylitis, psoriatic arthropathy and pauciarticular juvenile chronic arthritis--analysis of mononuclear cells from peripheral blood, synovial fluid and synovial membranes. Clin Rheumatol. 1988 Mar; 7(1):95-106.

403. Toussirot E, Saas P, Deschamps M, Pouthier F, Perrot L, Perruche S, et al. Increased production of soluble CTLA-4 in patients with spondylarthropathies correlates with disease activity. Arthritis Res Ther. 2009; 11(4):R101.

404. Wang YF, Wang M, Song AF. [Evaluation value of peripheral Th17/Treg balance in patients with ankylosing spondylitis.]. International Journal of Laboratory Medicine. 2020; 41(07):842-845.

405. Wang CL, Li KZ, Cui W. [Study on expression ofTh1, Th17, Treg cells and related cytokines in ankylosing spondylitis patients.]. Chronic Pathematology Journal. 2018; 19(09):1154-1156+1160.

406. Wang M, Liu C, Bond A, Yang J, Zhou X, Wang J, et al. Dysfunction of regulatory T cells in patients with ankylosing spondylitis is associated with a loss of Tim-3. International Immunopharmacology. 2018; 59:53-60.

407. Wang H, Sun N, Li K, Tian J, Li J. Assay of Peripheral Regulatory Vδ1 T Cells in Ankylosing Spondylitis and its Significance. Med Sci Monit. 2016 Sep 6; 22:3163-3168.

408. Wang ZL, Zhong NF, Ma L. [A Study on the Clinical Value and Correlation of Treg and Th17 Cells among Different Active Stages of Ankylosing Spondylitis.]. Journal of Guizhou Medical University. 2015; 40(01):68-71+75.

409. Wang C, Liao Q, Hu Y, Zhong D. T lymphocyte subset imbalances in patients contribute to ankylosing spondylitis. Exp Ther Med. 2015 Jan; 9(1):250-256.

410. Wang YF, Xu LH, Jiang LX, Qi CP, Wang Y. [Clinical Significance of Detecting Immune Functions on Patients with Ankylosing Spondylitis.]. Journal of Modern Laboratory Medicine. 2012; 27(06):132-134.

411. Wang JX, Wei P, Meng JH, Liu XC, Liu YJ, Gu G, et al. [Expression and significance of Thl／Th2 cytokines in ankylosing spondylitis.]. Clinical Medicine of China. 2008; 24(10):989-990.

412. Wei YY, Han ZJ, Huang HY, Du W, Ren TL, Gao MZ. [Analysis of Treg cells and lymphocyte subgroup in 131 patients with ankylosing spondylitis.]. China Medical Herald. 2017; 14(28):46-48.

413. Wu SS. [Association of Follicular Helper T Cells and Ankylosing Spondylitis.] [master’s thesis]: Anhui Medical University; 2014.

414. Wu Y, Ren M, Yang R, Liang X, Ma Y, Tang Y, et al. Reduced immunomodulation potential of bone marrow-derived mesenchymal stem cells induced CCR4+CCR6+ Th/Treg cell subset imbalance in ankylosing spondylitis. Arthritis Res Ther. 2011 Feb 21; 13(1):R29.

415. Wu HK, Zhou L, Zhang LZ, Zhong RQ. [The expression research of B lymphocyte subsets，B-cell activating factor and its receptor BR3 in peripheral blood from patients with ankylosing spondylitis.]. Laboratory Medicine. 2011; 26(12):818-822.

416. Xu F, Guanghao C, Liang Y, Jun W, Wei W, Baorong H. Treg-promoted New Bone Formation Through Suppressing TH17 by Secreting Interleukin-10 in Ankylosing Spondylitis. Spine (Phila Pa 1976). 2019 Dec 1; 44(23):E1349-E1355.

417. Xu WL, Luo Y, Li K, Liao CZ, Lin YH, Zhang HD. [Eepression and significance of T lymphocyte subgroup and natural killer T cells in peripheral blood of ankylosing spondylitis patients.]. Laboratory Medicine and Clinic. 2018; 15(02):192-194.

418. Xu XX. [The differentiation of Th1/Th17/Treg cells and their expression of associated transcription factors and cytokines in patients with ankylosing spondylitis.] [master’s thesis]: Fujian Medical University; 2013.

419. Xu XF, Jiang LH, Gao WH, Tao L, Huang LJ, Xu QB. [Detection of HLA-B27 and T lymphocyte subsets in patients with ankylosing spondylitis and its meaning.]. Laboratory Medicine and Clinic. 2011; 8(19):2366-2368.
[truncated: 50,448 more chars]
