# Supplementary material for: A guideline on biomarkers in the diagnosis and evaluation in axial spondyloarthritis
Source: Front Immunol. 2024 Oct 30;15:1394148. doi: 10.3389/fimmu.2024.1394148 (PMC11557325; doi:10.3389/fimmu.2024.1394148)
Supplement: Supplementary file 7 [file Table7.doc]

**SUPPLEMENTARY APPENDIX 7: Guideline project plan**

Spondyloarthritis (SpA) is a group of inflammatory diseases. Ankylosing spondylitis is the main clinical type of spondyloarthritis. It is characterized by sacroiliac arthritis and tenosynovitis and mainly involves the central axis. Chronic inflammatory diseases of joints, the prevalence of AS and SpA is between 0.1% and 1.4%, which causes a major burden on the social economy.

In recent years, the International Rheumatology Alliance organizations such as The Assessments in Ankylosing Spondylitis International Society (ASAS), EULAR and ACR have separately developed AS expert recommendation guidelines, emphasizing that patients should be A detailed and comprehensive assessment not only includes the safety of medication, but the assessment of disease activity status is also crucial to the choice of medication. ASAS recommended ASDAS and other series of indicators jointly evaluated by patients and doctors provide patients with a valuable means for evaluating disease activity, treatment effects and various disease indexes.

Regarding the etiology, clinical manifestations, diagnosis, patient education, rehabilitation, medication, and follow-up of AS, although there have been new studies and discoveries of biomarkers at various levels in recent years, there are still subjective aspects in clinical practice. Especially in clinical practice, how to fully understand these biomarkers and apply them in a standardized, standardized, and rational manner needs urgent improvement. From the perspective of individualized and precise diagnosis and treatment, it is necessary to study and formulate guidelines for AS biomarkers.

This guideline study was proposed by Professor Gu Jieruo. It will build a methodology based on the guideline, follow the steps of the "WHO Guidelines Development Manual", and make recommendations based on the GRADE classification based on evidence-based diagnosis and treatment issues affecting clinical decision-making.

1. Method for developing guidelines of ankylosing spondylitis biomarkers

This guideline study was proposed by Professor Gu Jieruo. It will build a methodology based on the guideline, follow the steps of the "WHO Guidelines Development Manual", and make recommendations based on the GRADE classification for diagnosis and treatment issues that affect clinical decision-making.

2. Working Group on Guidelines of Ankylosing Spondylitis Biomarkers

According to the requirements and needs of the guideline work, a guideline guide group, guideline development group, guideline secretarial group, guideline consensus group, and guideline external review group are established. The groups are as follows, and regular work meetings are held to ensure the smooth development of guideline development.

1.2.2.1. Guide Steering Group

The steering group consists of 5 members, including 3 rheumatology clinicians and 2 evidence-based medicine experts. Its main responsibilities are as follows: ①Determine the scope of the guide; ②Select members of the guideline development group, secretarial group, consensus group and external review team; ③Organize guideline development meeting; ④Review selected questions and outcome indicators; ⑤Supervise literature search, Production of systematic reviews; ⑥ Review of declaration of interests; ⑦ Approval of the release of recommendations and guidelines.

2.2. Guidelines Development Group

It consists of 15 members, including clinical experts, patients, evidence-based medicine experts, statistics experts, health economists, and ethics experts in different disciplines. Its main responsibilities are as follows: ① Formulate a guide plan; ② Determine the issues to be included in the guide and determine the importance of the outcome indicators; ③ Instruct the secretary group to conduct investigations and systematic reviews; ④ Handle external audit opinions; ⑤ Write the full text of the guide.

2.3. Guide Secretary Group

It consists of 5 members, including 1 evidence-based medicine expert and 4 rheumatologists who have worked in the rheumatology and immunology department for more than ten years. Its main responsibilities are as follows: ①Search the literature and understand the relevant questions to be investigated; ②Carry out questionnaire survey and screen the core questions of the guidelines; ③Formulate a systematic review; ④Meeting records.

2.4. Guidelines Consensus Group

It consists of 10 members, mainly rheumatologists who have worked in the Rheumatology Department for more than ten years. Its main responsibility is to form recommendations.

2.5. Guide external review team

It is composed of 7 members and is a guide stakeholder, including medical workers, pharmacists, and patients. Its main responsibilities are as follows: ①Evaluate the scope of the guideline and the selected questions; ②Evaluate the choice of clinical outcome; ③Review the draft guideline.

3. Declaration of interests

All members participating in the preparation of the guide sign a declaration of interest form, manage conflicts of interest according to the norms, and present them in the attachment of the guide to reduce the risk of bias caused by conflicts of interest. The declaration of interests of all members will be presented as an attachment in the final guidance document.

4. Guidelines for ankylosing spondylitis biomarkers

The guideline team performs the following tasks: selecting clinical issues, question classification, and statistical analysis, and in turn performing the Delphi method to determine the clinical issues to be covered by the guideline.

5. Evidence retrieval

The system searches according to the selected questions. Chinese databases include CNKI, WanFang Data, CBM, and English databases include Pubmed, Embase, the Cochrane Library, Clinical trails.gov and other databases. Select keywords according to specific problems, and use a combination of subject words and free words to search. Evidence of selected clinical problems are supported by evidence-based medicine through meta-analysis/systematic review.

6. Evaluation of evidence quality

The GRADE classification was used to evaluate the quality of evidence for clinical problems selected by the ankylosing spondylitis biomarker guidelines.

7. The preliminary recommendations of the Working Group on the Guidelines for the Formation of Ankylosing Spondylitis Biomarkers

The guideline group uses the Delphi method to grade the strength of the preliminary recommendations.

8. External review of guideline recommendations and formation of guideline

The guidelines are formed in accordance with the requirements of the Reporting Items for Practice Guidelines in Healthcare (RIGHT), and are submitted to the guideline steering group for discussion, voting, and correction to determine the final guideline.

9. Publication and dissemination of guidelines

The full guideline will be published in 2021 and will be introduced on academic conferences and websites related to rheumatic immune diseases.
